# A Qualitative Study of Environmental Factors Important for Physical Activity in Rural Adults

**DOI:** 10.1371/journal.pone.0140659

**Published:** 2015-11-10

**Authors:** Verity Cleland, Clarissa Hughes, Lukar Thornton, Alison Venn, Kathryn Squibb, Kylie Ball

**Affiliations:** 1 Menzies Institute for Medical Research, University of Tasmania, Hobart, Tasmania, Australia; 2 Centre for Rural Health, University of Tasmania, Hobart, Tasmania, Australia; 3 Centre for Physical Activity and Nutrition Research, School of Exercise and Nutrition, Sciences, Deakin University, Burwood, Victoria, Australia; University of Edinburgh, UNITED KINGDOM

## Abstract

**Purpose:**

Despite increasing evidence that the physical environment impacts on physical activity among urban-dwellers, little attention has been devoted to understanding this relationship in rural populations. Work in this area is further hindered by a lack of environmental measures specifically designed for rural settings. This qualitative study aimed to explore the salience of urban physical activity environment constructs among rural adults.

**Methods:**

In 2011, 49 rural men and women from three distinct areas (coastal, animal-based farming, forestry/plant-based farming) of rural Tasmania, Australia, were purposively recruited to participate in semi-structured interviews. Interviews explored features of the built and social environment commonly examined in studies of urban adults, including functional characteristics (eg, lighting, footpaths, roads/verges), road and personal safety, availability and accessibility of places to be active, destinations, and aesthetics. Interviews were recorded, transcribed verbatim and analysed using a content-thematic approach using QSR NVivo software.

**Findings:**

While some urban environmental constructs were salient to these rural adults, such as availability of and accessibility to places to be active, some constructs were operationalised differently, such as road safety (where large trucks and winding roads rather than traffic density was of concern), or were not considered relevant (eg, personal safety related to crime, availability of walkable destinations, aesthetics).

**Conclusions:**

The measurement of the physical environment in rural populations may require reconsideration and/or modification to ensure salience and appropriate quantification of associations with physical activity in future studies.

## Introduction

Physical inactivity is one of the major modifiable risk factors for cardiovascular diseases, the leading cause of death in most Westernised countries [1, 2], and is the fourth leading contributing factor to death worldwide [3]. In upper-middle- and high-income countries, the proportion of adults not meeting physical activity guidelines is estimated at 40–45%, [4] and is even higher in certain population groups, such as those living in rural areas [5–11]. Higher rates of physical inactivity observed in rural areas may contribute to explaining the lower life expectancies and poorer health outcomes of rural residents [5, 12]. Understanding the factors that influence physical activity behaviour among rural adults is therefore an essential requirement for developing and implementing effective preventive programs and policies.

Social-ecological theories highlight the concurrent role of individual, social and environmental factors in influencing behaviour [13]. A review of 11 review papers investigating the physical environment and physical activity concluded that there are reasonably consistent associations between physical activity participation and access to physical activity facilities, convenient and proximate access to destinations, high residential density, land use, urban “walkability” scores, perceived safety, exercise equipment, and footpaths (and that these associations were relatively consistent between studies of total physical activity and of walking) [14]. A key limitation of these existing studies is that they have been mostly limited to urban or suburban populations. Consequently, many of the factors identified may have limited transferability to rural populations (e.g. urban walkability scores, high residential density, and convenient and proximate access to destinations). Despite this, most studies assessing associations between environments and health behaviours in rural communities have done so using environment exposure measures best applied to urban settings (e.g. [9, 15–24]). This approach largely ignores the unique geographies of rural environments, which are characterised by large open spaces, low population densities, fewer facilities for activity, limited public transport, and large distances required to travel to places to be active [25]. As there is currently little evidence to guide the selection of appropriate measures of the physical activity environment in rural populations, this study used a qualitative approach to explore the salience of urban physical activity environment constructs among rural adults. To this end, in this paper we report the findings of the expectant (anticipated) themes related to the key environmental constructs explored.

## Materials and Methods

This study was conducted during 2011, and followed the ‘Consolidated criteria for reporting qualitative research (COREQ)’ guidelines [26].

### Ethics Statement

Approval to conduct the study was granted by the Tasmanian Social Sciences Human Research Ethics Committee, and informed written consent was obtained from all participants.

### Participants

The Australian Standard Geographical Classification Remoteness Structure system [27] was used to classify all postcodes of Tasmania, Australia, as Major Urban, Inner Regional, Outer Regional, Remote and Very Remote. Two Outer Regional areas and one Remote area were purposefully selected to provide representation from across the state (north, central and south) and diversity in area types (eg, small coastal area, animal-based farming, and fruit-growing). The demographic profile of these areas is represented in Table 1.

**Table 1:** Characteristics of the 3 purposefully selected study areas.

Characteristic	Area 1	Area 2	Area 3
Location	North	Central	South
Region type	Coastal	Central highlands/ lakes district	Forest/channel
Industry	Vegetable production	Mixed agriculture and tourism	Forestry/Apple- and fruit-growing
Remoteness Area^a^	Outer Regional	Outer Regional	Remote
Population^a^	10,323	991	1584
Area (sq km)	131.8	336.3	3578.3
Population/sq km	78.3	2.9	0.4
IRSAD^a^	914.4	865.8	885.9
IRSD^a^	942.0	908.8	905.1
Distance from State capital (Hobart) (km)	312.9	79.5	58.5
Number of Participants (male/female)	25 (10/15)	14 (4/10)	11 (2/9)

IRSAD: Index of Relative Socioeconomic Advantage and Disadvantage (where 1000 represents the national average). IRSD: Index of Relative Socioeconomic Disadvantage (where 1000 represents the national average)/

^a^Based on Australian Bureau of Statistics data from the 2006 Population Census

Purposive sampling techniques were employed to recruit 34 women and 16 men aged 18–55 years from the 3 regions. Uneven numbers of women and men were included as a result of study resourcing: a greater amount of funding was obtained for interviews with women than for interviews with men. Recruitment strategies included advertisements in local newspapers, features on local radio, posters in key neighbourhood locations (such as libraries, community houses, neighbourhood centres) and through networks of key community contacts. Snowball sampling, whereby volunteering participants refer other eligible people to the project, was also employed.

### Measures

Interview: A semi-structured interview schedule was developed to explore issues related to physical activity and the physical environment. Physical activity was defined as ‘any activity that lasts for at least 10 minutes and causes your body to work harder than normal—so your heart rate might go up, you might breathe a little heavier like huffing and puffing, and you might “warm up” a bit’. Through an open-ended questioning format, participants were asked to describe the intensity, duration and frequency of physical activity in the past two weeks at work (occupational), to get from place to place (transport), around the house/yard (domestic), and during leisure/discretionary/spare time (leisure).

Features of the built and social environment commonly examined in studies of urban adults [28] were explored, including functional characteristics (eg, lighting, footpaths, roads/verges), road and personal safety, availability and accessibility of places to be active, destinations, and aesthetics. Participants described these features, and whether they impacted on their physical activity. They were also asked whether there was anything else related to where they lived, their environment, or their physical activity that was not covered in the interview. The interview schedule was pilot-tested with two adults recruited via convenience from rural areas that were not targeted in this study, with minimal adjustments required.

Survey: Participants completed a brief demographic questionnaire including date of birth, language spoken at home, country of birth, highest qualification (and partner’s if applicable), employment status (and partner’s if applicable), marital status, number of children, access to a motor vehicle, injury/illness/disability that limits activity, height and weight. Body mass index (kg/m^2^) was calculated and weight status classified using standard definitions of overweight (25-<30kg/m^2^) and obesity (≥30kg/m^2^).

### Data Collection

Forty-five of the interviews were conducted by one female interviewer with qualifications in social work, women’s studies and environmental health. Four interviews were conducted by the lead author (female), a postdoctoral research fellow with qualifications in health promotion and epidemiology. Participants had no prior relationship with interviewers or the authors, and the goals of the research were clearly explained to participants prior to interview. Both interviewers followed the same semi-structured interview schedule. Interviews were conducted in community houses (n = 13), community health services (n = 5), community centres (n = 6), local council offices (n = 4), a participant’s home (n = 1), participants’ workplaces (n = 3), at the university (n = 7) or via telephone (n = 11). Interview length ranged from 27 to 62 minutes and lasted on average 43 minutes. Participants were provided the opportunity to review their transcript.

### Data Analyses

Interviews were digitally recorded, transcribed verbatim and imported into NVivo software (QSR, version 8.0), which was used to assist in coding and analysing transcripts (see S1–S5 Files). Data analysis involved one author (VC) reading and re-reading transcripts, listening to digital recordings, and thematic analysis of transcripts to identify common and contrasting ideas, supplemented by interpretive content analysis. Regular discussion and refinement of the themes occurred amongst the researchers and the interviewer/s. For this analysis, transcribed interviews were coded according to expectant themes (functional characteristics, road and personal safety, availability and accessibility, destinations, and aesthetics). Interpretive content analysis involves ‘counting’ the frequency of particular phrases or types of behaviour and provides numeric data that is useful in summarising findings. A second author (KS) verified coding of the expectant themes in a subsample of interviews (n = 10; 20%); any discrepancies were discussed until consensus was achieved, and deferment to a third author was not required.

## Results

### Participant characteristics

Most participants were born in Australia, had medium/high levels of education, were employed, and were married/living as married (Table 2). More than half had children living in the household, all had access to a motor vehicle, very few had an injury, illness or disability that prevented activity, and around a third of women and two thirds of men were classified as overweight. Participants were provided with the opportunity to raise any other issues, but none were raised, and no participants took the opportunity to review their transcript.

**Table 2:** Characteristics of study participants.

Characteristic		Women (n = 34)	Men (n = 15)
Age, *mean (min*, *max)*		43 (26, 55)	47 (34, 59)
Born in Australia, *n (%)*		29 (85)	13 ^a^ (93)
Education, *n (%)*	Low (Year 12 or less)	5 (15)	1 (7)
	Medium (Trade/ apprenticeship/ certificate/ diploma)	11 (32)	6 (40)
	High (University)	18 (53)	8 (53)
Employment Status, *n (%)*	Full-time work	12 (35)	14 (93)
	Part-time work	12 (35)	0 (0)
	Unemployed	2 (6)	0 (0)
	Keeping house	6 (18)	0 (0)
	Full-time study	2 (6)	0 (0)
	Retired	0 (0)	1 (7)
Marital Status, *n (%)*	Married/Living as married	30 (88)	12 (80)^a^
	Previously married	4 (12)	1 (7)
	Never married	0 (0)	1 (7)
Children in household, *n (%)*	None	21 (62)	9 (60)
	One or more	13 (38)	6 (40)
Children in household age (years), *Min*, *max*		0.6, 26	1.5, 22
Access to motor vehicle, *n (%)*		34 (100)	15 (100)
Has injury/illness/disability preventing activity, *n(%)*		1 (3)	1 (6)
Height (cm), *mean (min*, *max)*		169 (153, 184)	181 (162, 196)
Weight (kg), *mean (min*, *max)*		67 (52, 96)	91 (69, 123)
Body mass index≥25kg/m^2^, *n (%)*		10 (33)^b^	10 (67)

^a^ Data missing for 2 participants (denominator = 14)

^b^ Four female participants declined to provide height and/or weight data (denominator = 30)

### Key themes

#### Road safety

Road safety was considered an issue by about two thirds of participants, similar to the urban literature. However, one of the most common reasons for poor road safety was the high number of trucks on local roads, which combined with narrow and winding roads was a concern for both men and women in all areas.

*…once you go up on the main road you wouldn’t walk there for quids*…*it’s narrow and it’s tight…And we’ve had two or three deaths on it*. *And there’s trucks and there’s log trucks—and so you can’t really walk along it too much*. [Male, Area 3]

*‘But the road… I walked it once and I was terrified*. *Because it’s sort of a windy road*. *It’s narrow and you get log trucks and you get all sorts of…heavy farm equipment and there’s no place for you to get off the road*.*’* [Female, Area 1]

Other road safety issues identified less frequently were similar to those seen in the urban literature, including the speed limits, visibility at night, and tourist traffic. A fifth of participants had mixed feelings about road safety “*There are some places that are really safe…there are places where like I said before you have to walk on the road”* [Male, Area 2]. The remaining participants felt road safety was not an issue “*you can walk up the middle of the road*, *there’s very little traffic…most people walk right down the middle of the road when they go for a walk*. *…a lot of the places there aren’t actually footpaths*, *but you can walk on the road and feel safe*.*”* [Female, Area 2], with the majority of these participants being from the more populace coastal area (Area 1), or living in or close to the main township in their area, suggesting that town size and proximity to town centres may be an important consideration.

Three-quarters of the participants who indicated that road safety was an issue in their area also indicated that road safety impacted on their physical activity. Some participants felt that road safety did not impact on their physical activity directly, but impacted on where or how they undertook their physical activity “*That depends where I go”* [Male, Area 2]; “*No…it’s how and when I do it*” [Female, Area 2].

#### Personal safety

Unlike studies of urban populations, personal safety relating to crime and violence was not considered an issue for the overwhelming majority of participants “*You see the Police around a lot*, *so they’re a very sort of physical presence there*… *you just know that they’re around*…*it’s not a town that feels unsafe*” [Female, Area 1]. The 2 participants reporting that it was an issue both lived in a rural township (Area 1) and both had concerns related to alcohol. One also had security concerns related to the running of his business.

Similar to observations in urban samples, many participants (12 participants) indicated that there was no street lighting in their area (across all three areas), with about a third mentioning that there was adequate street lighting in their nearest township. Some participants felt that poor lighting impacted on their physical activity participation “*Lighting is a big thing for me where I live because you just can’t go anywhere after dark”* [Female, Area 2], but for others, this was considered a positive aspect of living in a rural area “*I like the fact that there aren’t that many street lights because we get really good stars at night”* [Female, Area 3]. Others felt that to have lighting in their area was an unrealistic expectation “*there aren’t enough houses up there to do that*, *to warrant the cost*” [Female, Area 3].

A number of other uniquely rural safety issues were raised, including injury risks related to uneven surfaces in paddocks or on tracks (8 participants; least common in Area 1; 17% of comments vs. 43% and 40% in Areas 2 and 3), a fear of snakes in summer (7 participants; most common in Area 2; 43% of comments vs. 17% and 20% in Areas 1 and 3, respectively), and concerns about strangers loitering in remote areas (4 participants, all from Area 1). Six participants (all female) commented that they would always carry a mobile phone with them when walking because of their isolation.

*…if I have to walk across a paddock and it’s wet and it’s windy or something*, *if a branch fell on me or if I slipped in the wet mud and fell over and did my leg or something*, *then I’d carry the phone as a backup communication* [Female, Area 3].

More than half of participants indicated that issues relating to personal safety did not impact on their physical activity, but of those that did, all were female. Personal safety related to knowing other people in the community appeared to impact on physical activity for a small number of participants, both in positive “*Most people know people so you feel safe out walking”* [Female, Area 2] and negative “…*with walking in the town I wouldn’t feel overly safe*. *I don’t know anyone in the community to sort of make sure that you know it’s all OK*…” [Female, Area 2] ways.

#### Functional characteristics

Although a number of functional characteristics were mentioned by participants, including connectivity of streets, shops and services, and public transport, the most commonly discussed features were footpaths, tracks and roads, and lighting.

Eleven participants indicated that their neighbourhood had footpaths, and that they were satisfied with these. All of these participants except one were from the more populated coastal area (Area 1) where a new walking/cycling track had recently been built; a number mentioned this new track and had favourable attitudes towards it “…*they’ve built a lovely walk along the beachfront…nicely removed from the edge of the road and wide enough…groups could pass each other*” [Male, Area 1]. About half (n = 22) of the participants indicated that footpaths were present in their area, but there were barriers to their use (least common in Area 1; 44% of comments vs. 88% and 100% in Areas 2 and 3, respectively). For instance, 13 participants indicated that footpaths were only present in the central part or main street of their closest town, nine commented on issues related to continuity or path length, and six felt that the surfaces of the footpaths were poor. A small number of participants also commented on footpath accessibility issues in their area for residents with prams (strollers/pushchairs) or those with mobility impairments who needed to use a wheelchair/motorised scooter.

While for some people walking or cycling on the road where paths or tracks were not available was an option, for many it was not.

*The edges*, *like a lot of those sorts of road*, *the edges are gravel and they tend to be a bit sloped*, *so you lose your footing and they’re not good for bikes…And also there’s electric fences so you don’t want to get too close to the edge* [Female, Area 1]

Some participants expressed that the presence, absence or condition of footpaths impacted on their physical activity.

…*having access to that track is really important*. *If it wasn’t there you’d be walking through thick bush and steep hills and it wouldn’t be as enticing* [Male, Area 1]

…*I would probably walk with the children to the sports centre more if there was a really good footpath*. [Female, Area 3]

…*if there were footpaths I’m sure I would*, *or room for pedestrians then I’m sure I would exercise more*. [Female, Area 1]

For others however, the presence, absence or condition of footpaths appeared to have limited impact on their physical activity.

*The inland part doesn’t have a footpath*, *but that doesn’t stop me from walking there with the dog and so on* [Female, Area 1]

*There’s not…a lot of the places there aren’t actually footpaths*, *but you can walk on the road and feel safe*. [Female, Area 2]

#### Availability and accessibility

All participants were able to identify places to be active in their community, including sports/recreational facilities (n = 41), parks/ovals (n = 28), natural amenities (eg, beaches, rivers, national parks; n = 28; most common in Area 1: 68% vs. 43% and 18% in Areas 2 and 3 respectively), and walking or cycling tracks (n = 20; most common in Area 1: 60% vs. 21% and 18% in Areas 2 and 3 respectively). Despite the availability of places to be active, many participants indicated they were not accessible or did not fit with their activity preferences.

*Oh there’s a(n) Aquatic Centre…but that’s only open in the summer months*, *because it’s an open pool*, *it’s not closed in*. [Male, Area 1]

…*the local town has got a cricket oval*, *and that’s about it*. *And I’m not a cricketer* [Male, Area 2]

‘*There’s a football club but I don’t play football*.’ [Male, Area 1]

*There’s the golf course*, *if you’re into golf*, *which I’m not* [Female, Area 2]

Irrespective of area or gender, a large proportion of participants (81%) felt that having places to be active impacted on their physical activity. Those perceiving more places to be active generally believed that this impacted positively on their physical activity, while those perceiving fewer places to be active commonly felt that this negatively impacted on their physical activity.

#### Destinations

While around 20% of participants indicated that there were no destinations that they could walk or ride to from their home, the remainder felt that there was at least one destination within walking or cycling distance from their home. While destinations commonly highlighted in the urban literature were mentioned (eg, shops, schools, cafes), other destinations more commonly associated with rural life included bushwalking trails, nearby townships and beaches. Despite many of the participants identifying at least one destination, more than half highlighted barriers to walking or cycling to these destinations (no differences across the three areas), such as the terrain, the long distances to reach the destination, road safety concerns, lack of time and the time of year (ie, season).

Of those who indicated that it was relatively easy to walk or cycle to destinations, approximately half agreed that this impacted on their physical activity (n = 11) while the other half felt that it did not (n = 8). In contrast, of those who indicated it was difficult to walk or cycle to destinations (n = 16), the large majority felt that this impacted on their physical activity levels (n = 13).

…*if the bike track went past our house I’m sure we’d use it all the time*. *But it’s just that you’ve got to load the bikes in the car and pack it all up and go*. [Male, Area 1]

*I think if there were shops closer*, *yes we would walk*. [Female, Area 3]

#### Aesthetics

There was little variation in perceptions of the aesthetic qualities of the physical environment. Although two participants felt that their environment was not aesthetically pleasing (eg, “*I’m bored with it*. *It doesn’t change and it’s every day”* [Male, Area 1] and eight had mixed feelings eg, “…*if you talk about [township]*, *oh it’s a bit of a hole*…*where if you talk about the farm*, *it’s beautiful*” [Male, Area 3], the overwhelming majority of participants felt that their local environment was aesthetically pleasing.

*As you hit [township]*, *it becomes lush*. *Green*, *trees*, *you know*. *And it’s magical*. *It’s just phenomenal*. *And the changing of the seasons*, *all the flowering trees*, *the flowering plums and the plum trees*, *the cherry trees*. *And the hawks and the crows*. [Male, Area 3]

Although two thirds of participants agreed that aesthetics influenced their physical activity levels eg, “*It calls you out to go and visit”* [Female, Area 2], approximately one quarter of participants felt that aesthetics did not “*But no*, *it wouldn’t make any difference*, *I don’t think*’ [Male, Area 1] and a small number had mixed feelings eg, “*Ah*, *that’s a bit of a combination isn’t it*? *I mean it’s nice to be out in a lovely setting*, *but at the end of the day I think you’ve got to have a health consciousness really*, *to be there mentally*, *and to value it”*. [Male, Area 1].

## Discussion

The aim of this study was explore the salience to rural adults of physical environmental constructs commonly used to measure physical activity in urban populations. The findings suggest that while some urban constructs are appropriate and relevant, many require reconsideration and modification to ensure relevance to rural populations. As observed among urban populations, availability and accessibility to places to be active (eg, recreational facilities) and functional characteristics such as footpaths were important considerations. However, the constructs of personal safety related to crime, the availability of walkable destinations (eg, shops, schools, parks) and aesthetics had limited influence on the physical activity of rural adults in this study. Issues related to road safety were important but operationalized differently to urban populations, further highlighting the need for modification of measures used in this population group. These differences have implications for the measurement of built and social environments in rural populations which are summarised with suggested modifications in Table 3.

**Table 3:** Suggested modifications to physical activity environments measures for use with rural populations.

Construct	Issue/s	Recommendation
Road safety	*‘Local streets’ not salient term	*Replace with term ‘local roads’
	*Emergent themes around large trucks & narrow/windy roads	*Include items such as ‘Large trucks are common on our local roads’ & ‘Narrow or winding roads make it unsafe to walk/cycle in my local area’
Personal safety	*Emergent theme around animal threats to safety	*Include item such as ‘Dogs/ snakes are a concern in my local area’
	*Emergent theme around surfaces and footing	*Include item such as ‘Uneven surfaces in paddocks &/or on tracks make walking unsafe’
Functional design	*Not just presence of footpaths—emergent theme about barriers to use of footpaths	*Include items such as ‘Footpaths in my local area are continuous’ or ‘Footpaths in my local area are well-maintained’
	*Emergent theme around lack of shoulders/footpaths on main roads	*Include item such as ‘There are shoulders /footpaths on most main roads in my local area’
Availability & accessibility	*Not just availability—accessibility & relevance to personal PA preferences important	*Include items such as ‘There are facilities available in my local area that provide activity options that I am interested in’
	*Weather/time of year a consideration	*Ask about accessibility separately for warmer and cooler months/seasons
Destinations	*Few destinations in walking/cycling distance	*Ask about availability (yes/no) rather than time or distance to destinations
	*Barriers to accessing destinations identified	*Assess barriers to walking/cycling to destination such as ‘The local terrain impacts on my ability to access destinations within walking/cycling distance’
Aesthetics	*Lack of variation in response	*Consider exclusion of measures of aesthetics /greater exploration where heterogeneity present

Road safety concerns were most strongly related to large trucks and winding roads with narrow or no shoulders, rather than high traffic density (ie, heavy or light), which has previously been negatively associated with physical activity [29]. A plausible explanation for this difference in findings is that the rural areas included in the previous review were predominantly North American; the rural areas included in this Australian study may have been less likely to be main thoroughfares between urban and rural locations. Road safety may be under-appreciated in rural areas because of the general assumption that roads carry less traffic. However, it is likely to be the case (as identified by some participants) that traffic along these roads travels at high speeds and in many cases comprises heavy vehicles. The narrowness of the roads and lack of a separate walking or cycling path increases the risk to pedestrians and cyclists. In rural areas where such conditions exist, it is unlikely that people will engage in active transport to destinations or engage in recreational exercise along these routes if it means risking their safety.

As observed in urban samples, the importance of functional characteristics (such as footpaths) was a relevant construct for rural residents. Interestingly, only four out of nine studies included in a review of the built environment in rural settings found positive associations between sidewalks or shoulders and physical activity [29]. The findings from the current study highlight the importance of assessing factors that hinder footpath usage in rural areas, such as continuity, length and poor surfaces, and strongly relate to the issues of road safety, described above. Availability and accessibility of places to be active (eg, recreational facilities) was also a relevant construct, with the rural adults in this study identifying numerous places to be active were available. However, as seen in studies of urban adults, accessibility was an issue for many (eg, opening hours, winter closures), as was a lack of facilities relevant to personal interests.

Although positive associations between low crime and physical activity have been observed in rural studies [29], in the current study there was a lack of concern about personal safety related to crime, which had little impact on physical activity. This may be related to Tasmania’s crime rates which are generally lower than the national average in Australia [30]. However, personal safety issues related to uneven surfaces on tracks and in paddocks and environmental hazards (eg, falling branches, snakes) were evident, particularly among women, and require specific consideration in rural samples.

While some participants mentioned destinations commonly described in the urban literature (eg, services, schools, shops) as being within walking/cycling distance, more common destinations included bushwalking trails, nearby townships, or beaches/rivers, which should be included in future studies examining this construct. Also important to assess are the barriers to using active forms of transport to get to these destinations, such as terrain, long distances, road safety concerns, and the impact of poor weather. Mixed findings have been observed in studies examining associations between ‘walkable destinations’ and physical activity [29]. These mixed findings may be due to a lack of consideration of destinations that are relevant to rural residents, such as those described in the current study (eg, bushwalking trails, nearby townships, beaches/rivers), which should be given attention in future studies of rural residents.

Nearly all participants in the current study felt that their area was aesthetically pleasing. Despite this, most felt that the aesthetic appeal of their area did not result in increases in physical activity. This is in contrast to the findings from other rural (quantitative) studies [29] which have consistently identified positive relationships between aesthetics and different types of physical activity, including total physical activity [16, 31], walking [32], and leisure-time activity [23]. This finding is difficult to interpret in this study because of the lack of heterogeneity in responses, so it is not clear whether those in a less attractive rural setting would do less or more physical activity. The impact of aesthetics on levels of physical activity amongst rural residents requires further exploration.

A number of differences in the key themes were noted according to area. For example, road safety was not considered an issue in Area 1 but was in Areas 2 and 3; personal safety, including ‘hoons’ and strangers, were only of concern for residents in Area 1 while footing was less of a concern in Area 1; and compared to Areas 2 and 3, those living in Area 1 were more commonly satisfied with the footpaths in their area, and more commonly reported access to the natural environment and to cycling and walking tracks. These differences may attributable to differences in the ‘rurality’ and/or profiles of areas. For example, the population density of Area 1 was much higher (78.3/km^2^) than of Areas 2 (2.9/km^2^) and 3 (0.4/ km^2^), Area 1 is further from the Tasmanian capital city, Hobart (312.9km), than Areas 2 (79.5km) and 3 (58.5km), and Area 1 is less disadvantaged (Index of Relative Socioeconomic Advantage and Disadvantage, IRSAD 914.4) than Areas 2 (IRSAD 865.8) and 3 (IRSAD 885.9). Of interest in terms of transferability of these findings to other countries, the population density of Area 1 is similar to that seen in rural areas of Europe (mean: 63/km^2^) [33] and the United States of America (USA) (mean: 65/km^2^) [34].

Limited qualitative research has examined environmental influences on physical activity among rural adults. However, some similarities and differences have been noted in other studies. One focus group study of 19 adults in a rural Midwestern county in the USA identified accessibility, destinations and sidewalks as important influences on physical activity in rural adults [35], which is similar to the findings for accessibility and footpaths, but not destinations (which did not seem impact on physical activity), described in the current study. A qualitative study of senior citizens in Oklahoma, USA, identified lack of indoor physical activity opportunities as a major barrier to physical activity, which did not emerge as a theme in the current study, possibly due to more extreme climatic conditions in Oklahoma compared to Tasmania [36].

This study had some limitations. It was limited to one state of Australia, but did include three distinct regions representing diverse land uses (eg, animal-based farming, plant-based farming, and forestry), landscapes (eg, inland, coastal, forest), and population densities. There may have been other environmental features of more concern to rural residents that were not explored; participants were provided with the opportunity to raise any other issues, but no other relevant issues were raised. The sample varied from the general population on some characteristics (education levels were higher, marriage was more common and fewer women were classified overweight or obese in this study), but were very similar in other areas (median age, proportion Australian-born, proportion of men classified as overweight or obese). Because of the cross-sectional nature of the study, it is possible that more active participants had greater awareness of their environment because of greater exposure; that is, people who are more commonly utilising places to be active may be more aware of what is available. Both a possible limitation and strength is that we did not focus on specific types or domains of physical activity (e.g. leisure, transport, walking) in relation to the environmental constructs explored, as this was an exploratory study: this lack of specificity may have diluted responses, or potentially strengthened the findings by allowing participants to describe physical activity through their own interpretation and experience.

Key strengths include this being the first study to use a qualitative approach to understand the salience to rural adults of commonly used constructs of the built and social environment, and how the built and social environment influences physical activity among rural adults. Definitions of rurality also vary internationally, but this study allows insights into the impact of the local environment on rural residents’ physical activity behaviour. As with much research related to environments and health, some of the results may be specific to the context explored and therefore we advocate for further work across and Australia and internationally to help confirm key factors most salient for rural populations.

Despite some limitations, the findings of this study have implications for research, practice and policy. Researchers examining the effects of the built and social environment in rural areas could consider modifying instruments, and testing the validity of these modifications in larger more representative samples using quantitative techniques, to ensure that the environmental factors acting as barriers or facilitators to physical activity among rural residents are thoroughly captured. If these findings are confirmed in larger, quantitative studies, there are possible implications for policy and practice, such as local councils considering safety audits of the main travel routes in and around their town and assess if separate paths are required to ensure safe travel, and ensuring that existing paths are well-maintained and provide linkages with commonly-accessed destinations or facilities. Again, if findings are confirmed through quantitative research, local councils, service providers and recreational facilities could work towards tailoring their physical activity programs to suit local needs, and towards ensuring that facilities are available at suitable times of the day and throughout the year.

In conclusion, this study, which aimed to explore the salience of urban physical activity environment constructs among rural adults, has identified a number of areas where current instruments could be improved upon to better reflect the nature of rural life. Although not conclusive due to the qualitative and cross-sectional nature of this study, consideration of these factors in future research and practice is warranted.

## Supporting Information

S1 File Interview transcripts 1–10. (PDF)

S2 File Interview transcripts 11–20. (PDF)

S3 File Interview transcripts 21–30. (PDF)

S4 File Interview transcripts 31–40. (PDF)

S5 File Interview transcripts 41–49. (PDF)
