# Supplementary material for: A Qualitative Study of Environmental Factors Important for Physical Activity in Rural Adults
Source: PLoS One. 2015 Nov 10;10(11):e0140659. doi: 10.1371/journal.pone.0140659 (PMC4640845; doi:10.1371/journal.pone.0140659)
Supplement: S1 File — (PDF) [file pone.0140659.s001.pdf]

Date: 10 October 2011

Duration: 37 minutes, 27 seconds

Interviewer: [interviewer]

Transciber: [transcriber]

So I'll just start with talking a bit about the place where you live and just asking you a bit about what it's like to live where you do, and your likes and your dislikes.

Ah, yeah that's very open isn't it?

Mm. So are you living in [town] or ..

Yeah, I live in, live in [town]. Yeah, I mean I really love where I live.

Yeah. And um, how long have you lived there?

Ah the house we're in at the moment, we've been there for about four going on five years. I've lived in the [x] area, municipality area my entire life. Or married life anyway, adult life. So I suppose for 25 odd years, 30 years maybe.

Yep. And you mentioned that you lived in [town] itself. Is that a house on a suburban block?

Yes, it is. Yes.

OK. And um your reasons for living there? You are saying that you've grown up there, but your reasons for living in [town] itself?

Um, look I guess it's all related to family. You know, family and friends, work colleagues. I mean when I first got posted [job], I was posted to [town]. I guess those early years you do make friendship circles. I've never really had a desire to leave. And I guess once your kids come along you just want to be in the one spot. And fortunately with employment I was lucky to stay in the one spot so I didn't have to move.

Yeah, that's lovely. And often people do say that, about they find a place and it's good to just be, be able to live your life there. And especially with family and stuff coming, it's good.

Yeah, yeah.

Now in this research we've been doing in urban areas - as I was saying most of the research has been done in urban areas - people often refer to their local neighbourhood and I was interested in understanding what the term neighbourhood means to men who live in rural areas. And indeed if it's relevant. So what does the word "neighbourhood" mean to you?

Neigbourhood. Ah, well I guess it's like a um, a smaller section of where you do live. If you know what I mean. Like within [town] itself, I guess your neighbourhood is your direct ah

neighbouring areas and streets that are kind of around you, which are probably the areas that you are more likely to know people or you are more familiar with, so I guess it becomes your neighbourhood.

So if you were going to put a distance around that, how far would that be? A kilometre or less?

Oh, I guess that whole area would be maybe what, maybe like a 3km, 2km radius.

Yep. And how far do you actually live out of the centre itself? In [town]?

Probably less than 2km. Walking distance. Yeah.

And what about the word "community" what does that mean to you?

Ah, community I think is the greater um, the greater neighbourhood I suppose. A combination of all the neighbourhoods in the [area]. That is our community that we live in I guess. But it depends on what sense you are talking about too. I mean I think you know it's your local community when you are talking about what it is you are doing for your local charity or your local issue going on in your town, that's your community. But like when we are in [continent], like [state] feels like your community. You know if a bloke in [state] does something, well then that is part of your community then isn't it? So when you're home your community is, it's all in context I suppose.

That's a really important point, it is very much really community is more related to the context. Yeah. Yep. So for you, for your community now, if you needed to put a distance around that, what would it be?

Ah, yeah, OK, so generally the municipal area I would say. Um, not sure how big ours is. But I guess it would be, you know I consider our rural component of [town] part of the community. And some of those would be you know three quarters of an hour drive away, of 30 km. That's a fair bit of travelling. You know there might be 40 or 50 km away. Still part of our community.

Yep. Great. Now I'm going to talk a bit about physical activity now. And as I said that is a very broad term and it changes throughout your life. So what does the term physical activity mean for you?

Um, I guess anything that um creates an increase in the heartbeat per minute. Apart from that related to adrenalin I guess, instead of sitting still and not doing anything. So I guess it has got to be, it's got to have movement associated with an increased heart beat.

Yeah. Yeah. Now that's really clear. You've actually linked it to movement and sort of heartbeat, heart rate as well. Which is, um that's very clear. That's quite specific. Um, do you consider yourself to be physically active?

Yes, I would. Yes.

And why would you say that?

Ah, because I do something that fits the definition on a daily basis. Something that increases the heart rate on a daily basis.

Great. And I'll go into that a bit more about what to do in your life to be physically active. But just to finish this bit, is um, where does physical activity fit into your life? Is it of no interest, something that's a priority, or a high priority for instance?

Ah yeah, OK. It's probably not a high priority but it's a you know, it would be, it's a routine. So I guess a routine is like, yeah it's not a priority but it's just something you do. You make time for. Yeah, don't know what, something in there.

Yeah, that's clear. That's clear. So in our research when we think about physical activity, we think of any activity that lasts for at least ten minutes, the evidence of health benefit. And causes actually you were saying, your heart rate to go up, your body is working a bit harder. Um, or it might be you are breathing a little heavier or you might warm up a bit. So thinking about this type of physical activity over the last two weeks – and I'll go through different parts of your life, not to segment your life but just to make sure that we cover the different parts of your life. OK?

Yep.

So at work, have you been active at work for at least ten minutes at a time in the last two weeks.

At work, no.

Uh hum, great. And could you tell me a bit about why that is?

Ah, well I guess it's you know if you can call walking from your office to a pigeon hole, it doesn't fit my definition of what exercise I do.

So it's more the nature of your work.

The nature of the work doesn't provide for that sort of thing.

Yep. And if you had wanted to, is there anything that would have helped you be more active at work?

Ah, if I wanted to, well I do, I mean I'd like to be. But I mean it's just not possible to do that. Um, you have roles and responsibilities. And you can't, you can't do those in an active way, you have to do them in a passive way and there is not the time to get away from those to be active.

Yep. Thank you for that. That's really clear. And I think that it's really a theme that is coming out about work these days and the nature of work. That a lot of people have you know, have that sort of work that you are not able to be active, not only for the nature of the work but the fact of time and things like that, um doesn't fit. OK. So in the past two weeks have walked or cycled for at least ten minutes of the time to get to or from a place?

Ah, well I have. I've walked and cycled. But when you say to get to or from a place, um not really. It's actually leaving home and getting back to home. It's not a mode of transport which I think is what your question might be.

Um, yes, though as we go I'm not too fussed about where we fit things that you do in your life, it's more about covering the things that you do. So I'm happy if you want to talk about it here or we can talk about on another part of this.

Yeah, yeah. Follow through with the routine you've got there.

OK. So um how do you want to do it? Do you want to say that you have walked or cycled ten minutes?

Well yeah I have, I have yeah. And can you tell me about what you did? In the last two weeks?

Ah I walked mainly. So ah walked a couple of kilometres exercising dogs. OK. And do you cycle as well? Did you say?

Yes, yeah I do. Yep. Yep.

OK. Now thinking about those two activities can you have a think about the intensity of the activities. Were they steady, moderate or vigorous?

Ah, I'd go moderate.

OK. And how long would you have done those over the last two weeks?

Um, each time or cumulatively over the last two weeks.

Ah just an overall estimate for how much you would have done.

On a daily basis?

Yeah, that's fine.

Ah, yeah at least half an hour a day. On just about every day I would say.

Yep. And when would you have done that? Would that before or after work and obviously the weekends as well?

Yeah, yeah, no after work. Evening time.

Yep. And where would you have done it?

Um, from home. So, is that what you mean?

Yep, no that's fine. And if you were on any, using any sort of walking paths or roads or footpaths.

Oh yeah, there's a cycle path that we go along and the rest of the time it's usually on the beach.

Yep. So would you walk more often than cycle. When we are thinking about those two activities?

Yes.

So, would walking be more likely every day?

Yes, it would yes.

And cycling how often would that be?

Ah maybe once every couple of weeks.

OK. And OK, so if it was every couple of weeks how long would the duration be of cycling.

Oh probably about an hour.

Yep. OK. Great. And um, OK, so thinking about where you cycled and where you walked, OK, why did you do it there? What attracted you to the place?

What attracted me to do it? Oh well, certainly with the cycling, the cycling path has made it way more attractive. It's not something I've done for many years. So that has certainly helped.

*Mm. Are you talking about the one that's um, between [*town*] or ..*

Yep. That's correct.

It's a real favourite, it seems to be. Everyone seems to be talking about it that I've talked to. It's great. And people are looking forward to more.

Absolutely. Yeah, we need more of it.

Yeah. OK. And were you alone or with others in these two activities that you did?

Ah, in the last two weeks I was probably with others most times. Yep.

And the walking? You were saying that was more on the beach?

Yeah, yeah on the beach. So that is often, I suppose I leave by myself and come back with others. So it's a bit of both.

Yeah, it's a great beach.

Yes.

It's sort of like when I've been up there it's been um, it's one of those things; you can just wander along the beach and really appreciate it. And you could be anywhere. You know, you are not in the middle of a town or anything, it's just beautiful.

No, no, that's right. Yes. It sort of has a very natural feel to it, which is really good, but you are only a kilometre out of the GPO.

Yeah, it's incredible, that you can just walk, to the beach. Can you walk to the beach from where you are?

Ah, we're lucky. We're just across the road from the beach, so..

Oh! Yes.

You don't really have to walk anywhere to get to the beach.

Oh that's great. Um, and thinking of those two activities, is this what you usually do or is it one off?

Yeah I usually do that, yeah.

Yeah? And does this change depending on the season or time of year?

Ah, no it doesn't. No. I make a point of doing it, even when you don't feel like doing it.

And if you'd wanted to is there anything that would have helped you walk or cycle more to and from places?

I guess as you were saying before, if there were cycle tracks, more of them, we would do it more often.

Yep. Yep. Thank you. Now in the past two weeks have you been active around the house or yard? That is inside or outside?

Yes. I have, yes.

Yep. And can you tell me about what you did?

Um, the gardening.

Uh hm. Especially this time of the year. It's starting to really happen isn't it?

Yeah, that's right. Yes.

So gardening.

Oh and the [16:38] as well. Do some bashing every now and then.

Yep. And so thinking about those activities, what would you say the intensity of them was? Steady, moderate or vigorous?

Oh, what was the first one?

Ah, steady. Or moderate.

Yeah, yeah, steady.

Steady?

Yep.

And how long over the last couple of weeks would you have done those activities?

Um in the last couple of weeks, um, I guess mainly weekends and they would be yeah, I suppose three or four hours.

And the frequency. How often – is usually on the weekend. So it would be what? Once a week? Or twice a week?

Oh as in every weekend or not every weekend?

Mm.

Ah, I'd do that sort of thing most weekends.

OK. Yep. And um, why did you do it at that time?

One would be weather, and two would be necessity, um, three I suppose would be interest.

Yep. And would you have done those activities by yourself or with others?

Um, probably on my own I would think. Yes.

And is that what you usually do? Or is it one off?

Is it what I usually do?

Yep.

Yeah.

And does it change depending on the season or the time of the year?

Um, it does yeah. I spend more time out in the garden when it gets to this time of the year.

Yeah. Everything in the garden is starting to go berserk.

Absolutely.

And if you'd wanted to is there anything that would have helped you be more active around home? Either inside or outside.

Ah, no I don't think so.

Yep, fine. Now, I'll just ask you if there is anything else we haven't covered. And have you been physically active for at least ten minutes during your leisure or spare time? So that's just um, asking you is there anything we haven't covered?

Oh, anything else that I've done that's ah, active?

Yeah.

No probably not.

OK. Now the last part of this is physical environment. Now we've talked to you a little bit about where you live and your activity, but we're also interested in talking to you about the physical environment, where you live and work and play. So when I talk about the physical environment I mean all the physical things that surround you, the roads, the trees, shops, traffic. Those sorts of things. And some of these things are things that people in urban areas have said are important for their physical activity. So we are interested in finding out whether they are relevant too for rural people. So can you tell me a bit about how easy or difficult it is to be physically active in your area.

Oh I would say it's very easy.

Yeah, yeah. [town] seems to be a very sort of um walkable and um has got great facilities. Natural and otherwise, yes, yes. Um, OK. So I'm just going to go through a couple of areas just to ask you about. The first one is availability and accessibility of places to be active. If I were to ask you about places to be active in your area, what sorts of things would you tell me about? Now, you've already told me about the beach and the cycling track. But this is a chance, you may not use these facilities, just to tell me a bit about them. There's a whole range, I presume, of sporting facilities.

Yes, there's parklands, there's um, there's walking paths. I mean you know you talk about the cycling paths to [town]. Before that even went through, that side, there was walking paths that sort of run through the parklands, or meander through the parklands. There's some physical fitness equipment that has sort of been put in there. Not that I use it, but there is gear and I do see people using it. The path sort of goes up alongside the beach near the river, it's a very pleasant – and they are extending those now with the new bridge that has gone through as well. So you can walk right round the river and the beach as well.

Well that's good they've got some facilities in the park as well. So you've got a bit of choice in terms of walking tracks and if you want to use the equipment as well.

Yeah.

So that's good. OK. So availability. There is definitely places to be active in your area. It sounds like [town] is pretty well appointed in that way. Um, and access – are places convenient? Are they easy to access. And that's in terms of cost and opening hours as well.

Yeah, they are. Yeah, not a problem.

So thinking about having places to be active, does having places to be active influence whether you are active or not do you think?

Oh I would say definitely.

Yep, yep. And if you wanted to be more active, are there things or places that would help if they were available or more accessible?

Well, I don't know if they could be more accessible. So yeah, I mean I guess for other people, the municipality that would [23:06].

Mm. And it would be sort of keeping up and obviously as you are saying, the interest is that they're, you know, that that is there and that is what they are doing. So it's just doing, they will be doing more of that. So in terms of walking tracks and cycle tracks.

Yeah, yeah that's right. I think it's all about proximity really. You know if it's a car drive to get to one of those things, well then it makes it more difficult to access. So it probably depends what part of the municipality you live in. But certainly in our case it's just not an issue. Like access is very simple and it's 24/7.

Well it's interesting. I've talked to people who are a bit further out. You know like they may live in [village] or [town] or you know, that sort of thing. But what people do is you know, they just put the kids and the bikes in the back of the car and head off down here to the bike track.

Yeah, yeah, it's good.

OK. Destinations. If I were to ask you about places you could walk or cycle to from your home, what sorts of things would you tell me about?

Ah, I would tell you about coffee shops.

Yeah, sounds good.

That's pretty high up on the weekend walks.

Absolutely.

Maybe the Sunday breakfast, um venue is always good. Um, family will often cycle to [town] and visit, because we know people out there. Ah, our friends, that is the other destination I suppose. Yeah, what else is a destination? Um –

Things like your workplace or shops or things like that or services?

Oh to actually walk to those sort of things you mean? Yeah, we'd walk into town and do whatever. Or sometimes cycle as opposed to run up and down in the car.

Mm. So there's a whole range of destinations and ah, yeah. So thinking about that, does having destinations influence whether you are active or not?

Ah, yeah, I think it does. I think it does. I mean it -

It certainly helps.

Well yeah it does. I mean it's interesting, like we will walk on a Sunday into town to get coffee or breakfast on a Sunday morning, and I know a lot of people from [town] will come through the cycle path to do the same. But nobody goes from [town] on the cycle path to [town] to have breakfast or coffee because there isn't any. Or if there is it's not very good. So it's one way traffic, because they like that kind of – you know, I'm talking about people my age too and the people we hang around with. Which is a very limited number I suppose in the overall context, but that attraction is, you know that sort of café dining is tragic. It's only a one way street at the moment.

*Oh well that is interesting too. But yeah, and certainly other people have actually said that. It certainly helps to have you sort of destinations and things like that – good coffee spots and breakfast spots are certainly on the agenda. Though I did have someone who said to me that what they do in the summer is they ride from [town] to [*town*] to the strawberry farm. Because they've got ice creams there.*

Yeah, that's a good idea.

So um, if you wanted to be more active would it help if there were more destinations within easy walking or riding distance?

Oh yeah, for sure. I'm not really sure what sort of destinations they would be but you know if there are other things of interest that were around that are in proximity then yeah.

But definitely good coffee would be high on the agenda.

Yeah, that's right.

Yes. I must admit I like quality coffee too. Makes all the difference. Yeah, we'll go for – like I live down in [city] but we sort of live around the corner from [reserve] and stuff like that. On Saturday if we go for a walk around the summit and back, we'll go down and have a coffee afterwards. Absolutely.

Yeah, which part of [city] is [reserve] in?

Pardon.

Is that, that's in the city is it, [reserve]?

Yeah. Yeah. Um, it's on the other side of the rivulet.

Ah, right.

So West [city].

Is it [x] Coffee Lounge in North [city]?

Oh yeah, yeah. Yep. You recommend those?

Yeah they're good.

OK. Good. Yeah. But you can never be too spoilt for good coffee. OK. Now looking at neighbourhood function and design, if I were to ask you about the built or manmade features of your area, what sorts of things might you tell me about and this is where we get into footpaths, lighting, whether the streets connect and how walkable it is. And I think generally we are talking about [town] being pretty um, pretty well designed that way.

Yeah, it is really. The lighting, I mean you know of a night time we don't walk around the beach way, because of the lighting we'd walk up in the town way. Um, so I guess that is always, but you know, how do you justify spending millions of dollars on lighting just in case someone wants to walk in the dark. Um, so yeah lighting is always one of those things I suppose. And the other problem is, and I guess you get it everywhere, is um you know putting paths where it is kind of sheltered as well, not being exposed. Um, yeah. Like I know some of the national parks over at Port [x] way, the path out to [29: 42] national park, I think it is, you know it's only about 2 metres back off the beach but it's a natural sand dune. It sits underneath the sand dune. So you can go there on a howling day and you can still walk and it is quite pleasant. Um, as opposed to, I don't know whether [city] is on your interview list or not, but –

No, no. It's just outside our list actually.

Ah right. But [city], they've put a boardwalk that sort of sits right out on the coast on the edge of the beach. Which is lovely on a nice day, but –

But a lot of the time you get that wind.

Oh you just cop it like nothing else. So yeah.

So do you think [city] has been well designed that way?

Yeah, reasonably well. I mean it's just got a, the coast line is exposed anyway. So you can't do anything about that, other than put in more trees I suppose. But they are all along the

inland side and there is a bit of a small dune most of the way along it I suppose, so you can get out of the wind slightly. But it is, um yeah, a bit exposed. But that's the – do you lose the views for putting in the trees or do you get the shelter or do you stick with the view. You know, so – I'm being pedantic now.

So do you think the built features in your area influence whether you are physically active or not?

For sure. Yeah.

Yeah. And if you wanted to be more active, are there any built features in your area that would help?

If I wanted to be more active?

Mm.

Are there any features that would help?

Mm. We've talked about extending the cycleways a bit.

Yeah, well that's right. Yeah, that could happen, I suppose. Yeah, but certainly catered well for everything else I think.

OK. Now the other side is the natural environment. If I was to ask you about the aesthetic qualities or the attractiveness of your area, what sorts of things would you tell me about? Now I think you have told me about the beach, and the –

The beaches yes. And the river way is ah, you know, quite spectacular as well. Um, yeah I mean we use it for skiing over the summer as well, waterskiing. Um, yeah, so it's, you know, it's a really good asset I think that is probably a little bit under rated, is the actual river itself in its use and that sort of thing. I mean they sail on it, all the sailing clubs there and there's a rowing club that access it as well. So yeah, it's a real feature of the town. It offers, you know, a number of sports for different interests.

And do you think your local environment is aesthetically pleasing?

Yeah. For sure.

And do the aesthetics of your area influence whether you are physically active or not?

Undoubtedly, yeah.

And if you wanted to be more active, are there features of your area that might help if they were more pleasing or attractive?

Ah, are there features that would help us to be more active? Well that's sort of like that last question and I suppose that having you know, an extended cycleway or even the walking

path they have now, there's still options to link them to go further south, inland. They've got some beautiful parks further back, walkways, but there is no reason why they couldn't link them as well, incorporating the river. So there's heaps of scope to actually extend the paths within [town] as well. And that concept of connecting the coastal communities.

Yep. That's a really important point actually. It's a really good to make. Um, now one last area, safety. If I were to ask you about safety in your area what sorts of things would you tell me about?

About safety and exercise? Um, well I don't know because it's um, I couldn't say there was anything unsafe.

Mm. It's more about, [town] is a pretty safe community, it's more about traffic things.

Yeah, right. Well where they have got the walking path, the traffic is negligible, like you know it's really quiet. Um, so there's not a safety issue at all, you know like the cycling, you know the access to that path. I mean you hardly park your car before you get to the cycling path. I think there is a road you've got to cross at one stage that maybe has five cars on it a day. It really is very safe. So yeah I couldn't say that there was anything there that they could do to make it safer than it is. Or that would make me think differently.

OK. So does road safety influence whether you are physically active or not?

Well it would, I would say. Yeah, for sure, road safety would. And I'd probably ride more on roads if I felt safe. But I don't. So I just stick to the cycle paths.

OK. No, that's a good point. That's a very good point. And does personal safety influence whether you are physically active or not?

Ah yeah it would, yes. Sure. Um, yeah. I wouldn't do it if I didn't feel safe.

Yep. And if you wanted to be more active, are there personal or road safety issues that if addressed might help you be more active?

No. No, I don't –

OK. So –

So you are asking me if there is more, if anything safety wise, if there was a change, it would make me more active?

Yeah.

No, I don't think there would be.

That's fine. So just before we finish, is there anything else that you'd like to tell me about where you live, your physical environment or your activity that we haven't covered today.

Ah, no I don't think so. Oh probably one thing I should mention is that they do have a designated dog walking beach. Um, and you know because we walk during winter, really the only people we see walking generally are people walking their dogs. Um, so I think if that policy wasn't in place and they said no, there is no dog walking on beaches, I think you would certainly cut back a lot of the, the people that do access a lot of the walking facilities because it's a dog friendly town. Or designates areas that are for dogs. I think that's the important one to ah, give them a pat on the back for, and to look at other communities, it does encourage people to use those facilities.

Yes, and indeed people have actually said in the study that having a dog actually really helps them be physically active on a regular basis. So, but you have to have the facilities to do that.

Yes that's right. You've got to have the facilities then to be able to go and walk your dog somewhere and know it's OK – other than on the side of the road or a footpath you know. Nice to be able to go through the parks or through the beaches. Um, and that you know, if you can't do that well then it sort of makes that even harder to take the old pooch for a walk.

Absolutely. Absolutely. OK, look –

AUDIO RECORDING ENDS

Date: 3 October 2011

Duration: 43 minutes, 44 seconds

Interviewer: [interviewer]

Transcriber: [transcriber]

OK. So I thought we made start by just talking a bit about the place in which you live. Um, so could you tell me what it's like to live where you do? Your likes and your dislikes.

Ah, OK. Well we live on a bush block um in the rural part of this area.

OK. So what part – you're not in [town] itself?

It's [suburb], so about 10 ks as the crow flies. Um, so there's a lot of trees, we have um some beautiful rich soil. We live right on the River [x], so we have access to the river. Um, what more info do you …

Um, well you were saying that you were living on a bush block, so what size is..

Oh we have 50 acres.

Wow. That's great.

So yeah, it's a lot of land. Or there is enough to keep us busy. So yeah, some of the things that keep me busy on that land are chopping wood, we have storms every now and then which create hazards. So tidying up after storms and that sort of thing. Um, was there any specific things that you needed…

No, because we'll go through it later anyway. That's absolutely fine. And the length of time you've been living there?

Ah, it's been about twenty-five months. Yes, just over two years.

OK. And your reasons for living there.

Ah, we had lived in suburbia, um and we just had a birth of our son and we wanted to bring him up in an area where it was a bit more um, opportunity for him to actually be around nature and outside of a town, and perhaps better opportunities for him to learn what we think is important in life.

That's important. It's important for children to have that contact.

Yeah, and we thought living – my wife is from [city] and I was from just out of [city], that um a more rural setting would be ideal for us to just slow down and have access to things that you don't normally have in the towns.

So what is the distance to your nearest town, that's [town] is it?

The nearest biggest town is [town]. There are a couple of very small towns, [town] and [town] would have probably a couple of hundred, sorry less than that. Um, [town] might have 50 or so houses. [town] might have about the same, spread out over a bigger area. Very small towns.

And so how far are you did you say, from [town]?

Ah, from [town] we are 15 minutes, from [town] to our house. So it's about um 12ks. Yeah.

OK. So as I was saying in the research that has been done, it's mainly been looked at urban areas, and people often refer to their local neighbourhood. And I'm interested in understanding what the term "neighbourhood" means for men who live in rural areas. And indeed, if it's relevant. So what does the word "neighbourhood" mean for you?

Ah, yeah I guess it is different living in the country. For me, for us, we live on a road which is about 5ks long. And we are at the end of the road. So I guess for us our immediate neighbours would be what we would consider our neighbourhood. So there is probably about 15 houses or blocks in between the [x] Rd and our place. And we know or have introduced ourselves to everyone. And since we've been there there's been a few natural, not disasters, but weather events, that have needed people to get together and help each other. So I guess for us that's what we would call our neighbourhood, would be our immediate neighbours.

And if you had to put a distance on that what sort of distance would it be?

Ah, yeah it would be probably about 5k radius maybe. Yep.

OK. And what about the word "community"? What does that mean to you and is that relevant?

Ah, yeah it is relevant. I would again say it's probably a bigger area. We see ourselves also as part of the [town] community. Which even though it is at more of a distance from us, we still, we use the services in the town, more so than we would in [town] or [town]. So I would say that community for us includes a much bigger area. And yeah we would associate ourselves with being in the [town] community rather than perhaps a smaller area inland.

So that distance would be..

That would probably be within 30ks of where we are. Yeah.

OK. Now I want to talk a bit about physical activity. And what does the term "physical activity" mean for you? It's a very broad term as we've said.

Ah, probably something that would include exercise, which could include working. Physical activity would be you are exercising at the same time and working. I'm not too sure. I haven't really thought about it to be honest.

Well that's really, thank you for being honest. Because I think that's important too. Because you know, we often don't think about things like that. We just sort of get on with our life and do things. So that's really important to know too. It's like this study we are really interested in looking at a range of what, not only what it means and what it is, because there is a whole range. And people are sometimes more active in their lives and sometimes less. And it's really important, in fact more important to talk to people who maybe are less active than, at this time of their life, for instance. Because that's when you start to find out about things – people sort of, yeah. So, do you consider yourself physically active?

I do. Yes. Yes. Definitely.

And why would you say that?

Um, I would probably say most of the time, well not most, but some of the time during work hours I sit in front of the desk. But as soon as I get home I'm active with my son, with the jobs that I need to do at home to provide a comfortable living for our family. Um, and yeah on weekends definitely active with the family. And again trying to make sure that we have enough wood for the fireplace, and the garden is nice and tidy. Or just recreational activity on the river. So yeah, I'm definitely active. I would consider myself active anyway.

And with 50 acres there's always something.

Yeah, there's always something to do when you have land, yes.

OK. And where does physical activity fit into your life? Like is it of no interest, a priority, a high priority? Where do you put it at the present moment in your life?

Ah to be honest it's something that happens without me necessarily planning it. In my day to day life I need to be active to be able to, like a say, to have the lifestyle that we need. So, our house is not like a normal house where you can just ah, switch on the gas or switch on a powerpoint. We chose to have an environmentally house, which sometimes creates more physical activity than people might realise.

Are you on mains power?

No. We're not on mains power. So you know I have to maintain the generator or the batteries. For our cooking, or sorry, our heating is all wood, so I have to chop ten tonnes or so of wood each year to make sure that we've got heating for our family. So I don't

necessarily do that because it's physical activity. It's just part of my day to day life that we need that to survive I guess, or to live our lifestyle. So it's important, but it's not planned as such. It's just part of our day to day..

And that's what people are actually saying. It's part of my day to day. A lot of people are saying that no I don't plan it, it's just get on with it and do it.

Yeah, yeah that's right.

Which is quite a different notion to like you know sometimes oh, when people talk about physical activity they, you know, when you read about things, they sort of think it's about well there's a particular thing that we do, and we do it once a week and we do it once a week by going somewhere to do it. Gym is the classic thing. You know. And what we're finding is that you know, people have quite a different notion of what physical activity is. It's really about their day to day life.

Yeah. I guess there's the day to day essentials. And there's the recreational or, you know the things we choose to do. The physical activity on top of those.

Yeah, that's a really interesting way of putting it too.

Because I look at um, like I would like to, or I have looked into and hope to continue doing martial arts here in town. But because my day to day life is busy enough, um it's hard to either physically, be physically in the shape where I can drive into town and do another physical activity. Ah, which is like I say, a chosen or an activity on top of what I already would do. So it's finding the time and the balance sometimes.

I'd like to look a bit more at those activities. And um, in our research when we think of physical activity, we think of any activity that lasts for at least ten minutes. That's the evidence of health benefit. And it causes your body to work harder than normal. Heart rate may be up, you notice, or you might feel warmer or you might start feeling like a bit of huffing and puffing. So thinking about this type of activity, um and I'd like you to think back over the last two weeks. And what' I'll do is just go through different parts of your life, not to segment life, but just to make sure that we don't sort of miss things out. So we'll go back and talk a bit more about the activities that you've talked about. Now first of all, in the past two weeks have you been active at all at work for at least ten minutes at a time?

Yes, I have. Yes.

OK. Can you tell me about what you did?

Um, I go and talk to a lot of land owners about um NRM activities and how they might be able to make use of our funding opportunities for them. So we have quite a few programs at the moment that I'm overseeing to be encouraging land owners to do shelter belts or fence off and replant water courses. So I've been out talking to land owners and walking paddocks and advising them on those sorts of projects. And again that's part of my normal day to day work. It's not every day. But definitely every week I'm out walking around the bush or paddocks.

OK. Now, just thinking about that activity over the last two weeks, could you tell me the intensity of that activity? Would it be steady, moderate or vigorous?

Ah it would be steady and moderate.

Yeah, depending on the terrain I would imagine.

Yeah, we have some pretty hilly terrain in this region. So ah yeah, there wouldn't be too much vigorous.

And the duration? How long would you have done this activity?

Ah in one visit?

Mm.

Normally we can be on site for about two hours. So probably an hour, hour and a half of walking around paddocks and talking.

And the frequency? How often would that have happened in the last two week?

Ah, in the last two weeks it's been almost daily. Except for weekends. So this is a particularly busy period for us.

Mm. Because spring would be very busy.

Yeah, spring there's lots of weeds emerging. We need to get all of our orders in for trees. And make sure landowners understand the full process of our funding. So yes. So daily I would say.

That would be for shelter belts also, as well as water courses.

Yes. Getting it ready for next years planting.

Yes. It's a busy time. And when would you do that activity? You've said it is during the week, is it mainly during the day?

Yes. During work hours. So generally 9 till 5.

And is this what you usually do, or is it one off? Now, you've said that you do usually do this.

I usually do it, but not

Not as often.

Yeah. Not as often.

So does it change depending on the season or the time of year?

Ah, yes.

So spring is a very busy one.

Spring, yes.

OK. And if you had wanted to, is there anything that would have helped you be more active at work? It sounds like ..

Ah, not this last few weeks, no.

OK. That's fine. Now, in the past two weeks have you walked or cycled for at least ten minutes at a time to get to or from places?

Yes. Yes I have.

Can you tell me about what you did?

Yeah. We, I took my son for a walk a couple of times. And we have a few tracks on our property. So we like to just go and walk down to the river. Or just go and make sure that the tracks, there's no trees down or that sort of thing. Yeah.

And what would be the intensity of that exercise? Would that be steady or moderate or vigorous? I guess with a two year old it would not be moderate or vigorous.

No, it depends. Sometimes, my son, I still need to carry him on my shoulders. So for me that can be moderate, but mostly steady.

Yep. And duration? How long would you have done that over the last two weeks?

Ah, I would say an hour at a time. And probably four times, I'd say.

And when did you do that?

It's on weekends. Yes.

And where? You've said your property. And why did you do it there?

Ah, because that was easy. It was there. It's safe. It's easily accessible.

Sounds like a beautiful spot for a walk too.

Yeah, that's right. It's aesthetically pleasing.

Yep. Yep. And you've said that you've done, you've walked with your son. And is that what you usually do, or is it one off?

Ah yes, we usually do, do the walk. Yes.

And does it change depending on the season or the time of the year?

Ah yes. There's not so much walking in winter. But pretty well every other season, yeah we definitely like to get our son out and about. It might change to the beach in summer. But yes, we do.

And in winter it may not be quite as often, but if there is a sunny day or you are out and about.

Yeah, whenever we get an opportunity, we definitely rug up. Yep.

And if you wanted to is there anything that would have helped you walk to and from places more often?

Ah, I don't think so. I think it's always there. And it's up to us, if the weather is right and we feel like doing it.

It's in overall balance in what you do in your life in terms of how much time you've got anyway. Now, in the past two weeks have you been active around the house or yard? So, at home inside or outside?

Ah yes.

And we were talking about that. Yes. So can you tell me about what you did?

Ah, well we are always looking after our son. So he's at the stage now where he's running around. So we like to let him run around the house, outside on the grass, and play chasie, or play with the kite or um, yeah do that sort of fun stuff. We also, I do a fair bit of gardening and look after the vegies.

So you've got a vegie garden as well that you are looking after. Yep. That's fairly constant and hard work.

Yeah, it can be. It's a different sort of physical activity I guess. You can pace yourself.

And the other one you were mentioning was the wood cutting as well.

Yeah, the wood cutting is just a constant, when you live in the bush. You always have to try and get enough timber for the few years coming.

Hm. So it's always searching for..

Yeah, there's always trees that are down. And cutting them and stacking them. So yes, I have definitely been doing a lot of that.

So thinking of those activities. Um, those three activities that you've mentioned, the intensity of them, would they be steady, moderate or vigorous?

Ah, with playing with my son it's probably steady. And the same with the vegie garden. But the wood chopping and stacking is vigorous. Yes.

Mm. Oh it's hard yakka. And duration? How long?

Um, in one sitting?

For those activities? For each of them is probably the easiest way.

Over the two weeks?

Yeah.

Um, probably playing with my son would be ah, I don't know, ten hours maybe over the last two weeks.

And how often?

Ah, that would be after work and on weekends.

Yep.

And before work. Um, gardening is generally on weekends. And that would probably a couple of hours, maybe half a day in total. And the wood stacking and cutting and carting would be probably two days, so sixteen hours maybe.

There's a lot of work to it.

Yeah, it is. When you work full time it can be a bit challenging.

And you've said that with the activities, when you've done them, um with your son, it's over the week and the weekend, the garden is more the weekend, and wood –

That would be the weekend as well. Sometimes it's after work, if we've run out of wood for the fire. But generally I prefer it to be on weekends.

Why did you do it at that time? That's the time that you have available.

Yeah, that's right.

OK. Especially when you are working full time, it's very hard juggling everything.

Yeah, it can be.

Is that what you usually do?

Yes.

And does it change depending on the season or time of the year?

Um, no. All of those are constant.

Yep. And if you'd wanted to, is there anything that would have helped you be more active around the home, either inside or outside?

Um, not working full time.

Yep. More time, yes.

That would be the only thing.

Now have you been physically active for at least ten minutes during your leisure, or spare time? And this is really a category for if there is anything else we've missed.

Um, I guess I'm not too sure, what else would be included in that, but um..

It's just if there is anything else that you've done that we haven't covered really.

Ah probably not. No, I don't think so.

Great. Good. Now I want to talk a bit, the last part is talking a bit about physical environments. And that is looking at the physical environments where we live, work and play. So, and when I talk about the physical environment, it could mean a whole range of things. Any of the physical things that surround you. It could be roads, trees, houses, shops, traffic. And some of these things are, people in urban areas have said may actually be relevant and important for their physical activity. So we wanted to check with people living in rural areas, whether they, and see whether they're relevant for you. Now, just first up, can you tell me a bit about how easy or difficult it is to be physically active in your area?

Ah, well I think it's quite easy for us to be physically active. Especially with a young boy and having access to yeah, roads and property and ah..

And you're close to the river.

The river. Yeah, if this was summer we could be out there swimming and canoeing or kayaking. So yeah, I guess we're lucky that we have access to things that people in the suburbs may not, do not have. Yeah.

And also you've got access to things that are immediately there for you. Um, in some rural areas people have to literally get in their car to ..

Yeah, that's right.

But it would be good to be living on land. People will say that they will, you know, be active on their property. Not only walking but there's always things to do on it.

Yes. There is. And that was part of why we bought the property, was to have all of that. We've been in suburbia. We know there are benefits and pros and cons to everything. And we know what suburbia offered us. And, yeah, we're lucky.

And um, OK. So I'm going to go through some categories now of the physical environment. And the first one is availability and accessibility of places to be active. And so if I was to ask you about places to be active in your area, what sort of things would you tell me about? And this may not just be what you actually use yourself, but being aware of what's around.

Ah well, I know there are quite a lot of walking tracks in the neighbourhood or the community. Is that the scale that you want me to consider?

Yeah, yeah. And that's what, around [town] area?

Yeah. Just across the river from us there's the big [x] mountain range. There's walking tracks all through there. There's also um mountain bike tracks. People can go on tracks to find fishing spots, um lots of horse riding.

And you've already mentioned the river with kayaking.

Yeah, river kayaking, canoeing, swimming …

It's sort of got everything this area, really.

Yeah, it has. We think it's got everything that we need. So ah, and then yeah you've got all the services in town. Ah, the gyms and the martial arts and dancing, or um yeah swimming pools, heated pools. And all the sporting..

So there's a range of choice.

Yeah, there's I would say more choice here than there was in the suburbia where we were. Yeah.

And um, accessibility. Um, are things easy to access? Like um, particularly for services in Ulverstone? In terms of the cost or opening hours?

Ah, yes I think that accessibility is fair and yeah, I wouldn't have thought it would be a burden or a constraint to doing exercise.

And does having places to be active influence whether you are active or not?

Ah, I think it does. Yeah, I think in our situation just having those um physical things close by. You've always got that option. If um, you're bored and you're sitting there and it's a beautiful day, we are often just "come on let's go for a walk" or "let's go down to the river" or yeah. One day when we have kayaks and some other things for the river then, yeah there's no excuse really. Especially if you are fit and healthy.

And if you wanted to be more active are there things or places that would help if they were available or more accessible?

Um, not that I can think of.

Hm. Because you've actually said that there is quite a bit available and accessible to you. Now, the next one is destinations. So if I were to ask you about places that you could walk to from your home, what sorts of things would you tell me about? And you've actually talked

about the property, and there is a walking track over the river. Ah, any other things for a local that you can walk to?

Ah there's parks, it would be a long walk but you could walk. Perhaps not.

What that's a few kilometres away is it?

Yeah, about 6k. So it's a long walk. You could drive.

So you could get there, but maybe getting back..

That's right.

So it's more easy to drive on that one. Yep. And does having destinations influence whether you are active or not?

Um, I think it does, yeah. Yes.

And if you wanted to be more active, would it help if there were more destinations within easy walking?

Um, I don't know actually. Most people would probably drive to a destination, and then do their activity. So ..

That seems to be the balance in a rural setting. Like you have got your property you are on and then subsequent to that, it's really getting in a car and doing some other things.

Yep.

OK. Now neighbourhood function and design. If I were to ask you about the built or the man made features, the non natural features of your area, what sort of things might you be able to tell me about?

Ah, what area again?

Your local. Your immediate area.

OK, my immediate area. Um,

Do you have footpaths and …

No. We have tracks, dirt roads, and that's probably about it.

Yep. And um, are they walkable? Those roads?

Yes, yes they are. Yes.

OK. So there's not too much traffic on them.

No, there's not. No, no there's not. Not like other areas of [country].

Yep. And do they connect together? Like, in some places in rural areas there's one major road and there's really not very much that you can walk. So people like have to, if they want to walk they've got to walk up and down this main road. Whereas some areas, other areas,

are well serviced in terms of they connect to all sorts of different pathways so you've got a bit of a choice of where you walk.

Yeah, yeah. We know all our neighbours pretty well. So we all would allow any of them to walk on our property, and they would allow us to do the same. So um, …

That's really nice local people support that way.

Yeah, I think we are, people that live in the bush generally, are like minded people and to be able to do that is, yeah it is a bit of a luxury.

Yes, that provides more choice.

It does, yes.

OK. And do the built features of your area influence whether you're physically active or not?

Ah, yeah I guess they do. I guess having access to that track is really important. If it wasn't there you'd be walking through thick bush and steep hills and it wouldn't be as enticing.

Yes.

Absolutely. And if you wanted to be more active are there any um, built features in your area that would help?

Ah, no I don't think so.

OK. Now the other side of it is the natural environment, aesthetics. If I were to ask you about the aesthetic qualities or the attractiveness of the area, what sorts of things would you tell me about?

Um, well we think it's nice.

You've got the bush, you are overlooking the [x] range, you can see there.

You can see a bit of the [x] range. Ah, compared to some of the places we've lived ah it's very beautiful. So yeah again, that's why we chose the area. Um, I don't know what to say. It is very beautiful. There are definitely, it's very aesthetically pleasing walking on the track.

Yeah. And do you think the aesthetics of the area influence whether you are physically active or not?

Um, yes I think it does. Yes.

Certainly one of the reasons you picked to be where you are. Yeah.

Yep.

And if you wanted to be more active are there features of your area that might help if they were more aesthetically pleasing?

That's possible, of course.

It sounds like it's pretty gorgeous as it is.

Yeah, I guess I'm thinking of when we were living in town, it was in a low socio-economic area and we would go for a walk after work with our dog. And yeah, there are days when you come across people or cars or whatever and it is a bit of a um, disincentive to go walking. So in that sense comparing that to the bush setting, um I'd walk in the bush anyday. That's the context that I'm thinking of.

So it really does help to have such a beautiful spot to be in. Yep. And that's um, so that's thank you for clarifying that. Because I think it's important, the difference. It's often, it's um I've found that people often who are living in rural areas find it um an interesting question to ask. Because what happens is it's a given for your choice to live rurally. It's part of that, that's it's beautiful. So to then start to think about it, it's something a bit different again.

Yeah. And some people have probably lived either where they are now or nearby for generations. And so for someone that has come into this area and seen [city] and big cities, yeah it's a different appeal.

It is. It is. And that's certainly another thing that has come out of this study, is that um it's interesting talking to particularly people up here in the [region]. A lot of people have actually come from elsewhere, and chosen to live here. So hm, for the very sorts of reasons. Yeah, more so than, like there are three study areas we're looking at. One is [region] and the other is down at [town]. So yeah, but it is more, yeah quite clear up this way. OK, one last area is safety. If I were to ask you about safety in your area what things would you tell me about?

Um, yeah I think generally it's pretty safe where we are. Occasionally you get people that come down in cars that like to explore new roads. And but apart from that it's pretty safe.

Exploring or hooning?

Yeah, oh trying to, who knows what they want to do? They get gently persuaded to move on.

So does personal safety influence whether you are physically active or not?

Ah I don't, ah where we are I don't think so.

So you are saying you are feeling quite safe here.

Yeah, I know when we were in the town, that did, definitely did. But not where we are now, no.

And when you are talking about the town, is that the town here or the town ..

No, in [state], [x] Bridge.

I used to work in that rural area, [x] Hills, [x] Bridge and so, yes, I know [x] Bridge. Um, so and does road safety influence whether you are physically active or not?

Ah it's definitely something to consider. Although it's a one way road, I guess and we are at the end, you never know who might be coming in to say g'day or if the neighbours are on the track. So yeah, you definitely you have to be aware that potentially someone might be driving up. Especially with our son. So that would probably be the only real safety concern.

If you were walking with him.

Yeah walking. Or, if when he's ready to ride a bike. If we are on the track.

Yes, because it would be ideal, the dirt road, wouldn't it?

Yeah. So people coming the other way, yeah would always be something that we have to consider.

That's a really important point. And if you wanted to be more active are there personal or road safety issues that if addressed might help you be more active?

No, I don't think so.

OK. Well that's it. But before we finish is there anything else you'd like to tell me about where you live, physical activity or the environment that you live in that we haven't covered today?

I don't think so. I think that's pretty well everything.

Great. Thank you.

AUDIO RECORDING ENDS

Interviewer: So first of all I was wanting to talk a bit about the place where you live.

Respondent: Yeah.

Interviewer: So I'm interested in actually sort of asking you to tell me just what it's like living where you do. Your likes and your dislikes. So you're living in...

Respondent: [town] .

Interviewer: [town].

Respondent: Yes.

Interviewer: OK.

Respondent: Yeah.

Interviewer: Yeah. So... and so if you could tell me a bit about [town]. and...

Respondent: [town]. [town]'s good. I mean [town]'s quite small but it's got a kind of concentration of about maybe 60 houses max, close to... there's just a shop, there's nothing else there. There's a bit of a marina with boats. So lots of people that live there have boats.

Interviewer: Yeah.

Respondent: And lots of people that live there are old. Lots and lots of retired people like ex yachties live there. It's kind of a mix between them and kind of your traditional Tasmanian kind of rural folk who are like tenth generation.

Interviewer: Yeah, lived there years. Yes.

Respondent: Yeah.

Interviewer: Yes.

Respondent: So there's a mix of that. There's a few horsey people, a bit of small scaling farming but no massive farms. And also the big thing there is the [x], there's the port there. And [workplace] have... so there's a lot of port action and kind of [x] farming kind of.

Interviewer: So do you live actually in the area?

Respondent: I'm in actual town.

Interviewer: Town itself. So...

Respondent: Yeah. So...

Interviewer: ... you're on a... in a house and house block?

Respondent: Yeah. Yeah.

Interviewer: Yeah.

Respondent: So we have a really small old house, kind of shack and just a kind of... just slightly larger than average kind of... I think its 11,000 metre square block. But then... so I've got neighbours on two sides but then on all of the other side's there's empty land. There's like one... so there are houses around but I can't see my neighbours' houses, if that makes sense. And we're off a dirt road so...

Interviewer: Oh OK.

Respondent: ... we don't... and we don't have any through traffic.

Interviewer: Aah.

Respondent: So we don't actually have any cars go past.

Interviewer: So a nice sort of spot.

Respondent: So it's kind of mixed. While it is kind of suburbanesque in the fact that it's like a suburban block it's actually quite rural and we don't have traffic, which is a really big thing.

Interviewer: Yeah, you're often on a highway.

Respondent: Yeah.

Interviewer: And the length of time you've been living there?

Respondent: Four years.

Interviewer: OK. And the reasons for living there?

Respondent: Because we want. We wanted to live outside [city].

Interviewer: Yeah.

Respondent: And we got... it was just the cheapest house because we were students; it was the cheapest house that we could afford. It was really, really cheap and it was liveable. We wanted within like a 40 minute drive of [school]. So we just met that, that's the criteria that we basically... we knew the area we wanted to go but it wasn't necessarily because we love [town] or we love the house, it was because we didn't have very much money, so yeah.

Interviewer: No. Yeah. And it came together.

Respondent: Yeah.

Interviewer: OK. And so now...distance to your nearest town, like you're just out of [town]...

Respondent: Yeah.

Interviewer: Yeah. You're in [town] but just... so how far are you from, like you mentioned the shop?

Respondent: The shop is walking distance.

Interviewer: Yeah.

Respondent: It's only a few hundred metres.

Interviewer: Yeah.

Respondent: It's... it might be 500 metres max. So yeah, we can walk there with the kids easily and back again, so...

Interviewer: Yeah. And then your...

Respondent: ... and that's where we get our mail and yeah.

Interviewer: Ah right.

Respondent: Milk, yeah.

Interviewer: Yeah. But then the next closest centre would be [town] wouldn't it?

Respondent: [town]. [town]'s only a few minutes away. Yeah.

Interviewer: Yeah. OK. So in research with women living in urban areas, people often refer to neighbourhood.

Respondent: Yeah.

Interviewer: And we're interested in understanding whether that's... what that means living in rural areas and whether it's relevant or not?

Respondent: Oh yeah.

Interviewer: So if... so what does the word neighbourhood mean for you?

Respondent: Oh quite a bit in [town] actually.

Interviewer: Yeah.

Respondent: I think, especially having kids and because there are so many retired people it means that people are at their houses a lot.

Interviewer: Yes.

Respondent: So people don't go off to work every day. People are there. And when I walk around with my children I see people all the time. I know all my neighbours and I know most of the people that don't work, I know them all like to talk to and just see. And some of them I'm very good friends with. And they're all older so... and having kids and small children as well, it's really good, it's... yeah, it's fantastic way to be because there's... they're all really interesting people in different ways. And while it doesn't have anything... it doesn't have people my own age, we don't socialise a lot because we've got small kids anyway, but it's quite a rich environment to live in. Yeah. So.

Interviewer: So if I was to ask you to put a dis... your distance around that neighbourhood what would you say?

Respondent: It would probably only be like a one, one and a half K radius. It wouldn't be huge.

Interviewer: Would that be at [town] itself?

Respondent: Yeah.

Interviewer: Yeah.

Respondent: Yeah.

Interviewer: Yeah.

Respondent: I mean my daughter goes to school in [town], so we're part of that community as well because we do a lot over there. But that's kind of different to neighbourhood.

Interviewer: Yeah. Well that was the other question I was going to ask you about is community.

Respondent: Yeah.

Interviewer: And what that might mean for you.

Respondent: Yeah.

Interviewer: And again whether that's relevant.

Respondent: Community I'd say is more [town].

Interviewer: Yeah.

Respondent: Yeah. I would put myself in terms of community in with kind of not [town] but [town] and [town]. Definitely. And even then [town] and you know what I mean, just that sense that we're not [town] you know.

Interviewer: Yeah. So that's got a broader kilometre. So distance wise that would be what? About ten K radius?

Respondent: Ten... ten K yeah. I'd say ten.

Interviewer: Yeah. And... I mean if you needed to define community what would it be?

Respondent: See neighbourhoods people I suppose that share the same geographic closeness.

Interviewer: Yeah.

Respondent: And seen on a daily basis. Whereas community would be more people who have... live in the same environment... kind of environmental area.

Interviewer: Yeah.

Respondent: And use the same services. So the same schools, the same shops. So yeah.

Interviewer: Yeah. That's really clear. Now I want to talk a bit about physical activity.

Respondent: Yeah.

Interviewer: And what that means for you. And I've already said it's a very broad thing that's why we ask people individually what it is to them. So what does the term physical activity mean for you?

Respondent: Both focused exercise, like I'm going to exercise for my health. But also physical activity, anything where I use my body, which includes picking up the kids, cleaning, gardening, walking.

Interviewer: Your day to day, what you do?

Respondent: Yeah.

Interviewer: Yeah. And do you consider yourself physically active?

Respondent: Only recently, yeah. Yeah. I'm more physically active I think this year than I have been for the last... since I started having kids. Yeah.

Interviewer: Yeah. That obviously makes a difference.

Respondent: Yeah.

Interviewer: But I'll ask you a bit about why... what's the difference now.

Respondent: Yeah. The difference... I mean I was very physically active and very physically fit in the past, having incred... and so... and then having children and studying and I think and just... there's just no time. So my physical... there's no space, there's just having kids. Being pregnant, breastfeeding, and looking after kids. So outside of that, and there's just no space whatsoever for physical activity. But now that they're older...

Interviewer: Yeah, you're starting to...

Respondent: ... can, yeah. It's finding the space to do more physical activity. Yeah.

Interviewer: Including that they're at a walkable age so you're walking around them…

Respondent: Yeah. I always walked with them.

Interviewer: Yeah.

Respondent: Always walked with them. Always walked with them. But even things like doing gardening. Like previously I could really commit to doing large chunks of gardening because of the distraction of everything and the kids. Yeah. So now I'm more active in the yard as well as choosing to do physical. Yeah.

Interviewer: OK. And where does physical activity fit in to your life? Is it of interest to you, no interest, a priority or?

Respondent: Yeah. Yeah. It's of a lot of interest to... I like being physically fit.

Interviewer: OK.

Respondent: It's, yeah, and healthy. I'm very healthy so fitness is part of that. Yeah.

Interviewer: Yeah. Great. OK. Now I'm just going to go on and talk a bit more about that. In our research when we think about physical activity we think of any activity that lasts for at least ten minutes, the evidence of health benefit is that one, and causes your body to work harder, you're starting to feel warmer, handy in winter, and heart rate up, a bit of huffing and puffing. So thinking about this type of physical activity during the past two weeks, I'll just go through some parts of your life here.

Respondent: Yeah.

Interviewer: Not to segment your life, but just make sure that we cover them basically.

Respondent: Yeah.

Interviewer: Now are you currently working at all?

Respondent: Yeah.

Interviewer: Yes.

Respondent: Part... a few days a week.

Interviewer: OK. So thinking about the last two weeks, have you been active at all at work for at least ten minutes at a time?

Respondent: Yeah. Often I walk around a lot and I use the stairs at the [workplace]. So there are periods when I'm active for the ten minute mark in terms of walking around and using the stairs. Yeah.

Interviewer: OK. And how would you define the intensity of that exercise? Would it be steady or moderate or vigorous?

Respondent: Oh just steady.

Interviewer: Yeah.

Respondent: Yeah.

Interviewer: OK. And the duration you've already said is at least ten minutes.

Respondent: Yeah.

Interviewer: And how often would that be in the last two weeks, two days a week that you're there?

Respondent: Two days a week. Only two days a week realistically for about ten minutes of like walking, not standing. Probably I think about three times a day.

Interviewer: Yeah.

Respondent: Each day.

Interviewer: Yeah.

Respondent: Twice a day. Yeah. Twice a week. Yeah.

Interviewer: And OK, so is that what you usually do or is it a one off?

Respondent: Usual. Yeah.

Interviewer: And does it change with the time of the year or the season?

Respondent: Yeah. If it's good weather, like if it's warm I'll walk... drop my son off at child care at the [child care centre] and walk in to work and then back from work, which I see as part of my working day.

Interviewer: Yeah.

Respondent: So I do that when the weathers a bit better, I don't do it in winter.

Interviewer: Yeah.

Respondent: It's just... it's, yeah.

Interviewer: Yeah. Fair enough. And how long does that take you?

Respondent: Half an hour.

Interviewer: Oh OK. Yeah, that's a nice walk in the morning. Yeah.

Respondent: Yeah.

Interviewer: OK. And is that just to drop him off or you do it the other end of the day as well?

Respondent: Yeah, generally if I'm going to do it I'll do it... depends, changes from day to day depending on how late or early we're running. But yeah, I... it's a constant thing is that we drive in and go to [school] and then from there I either catch a bus or walk.

Interviewer: OK.

Respondent: Yeah. So in summer I do at least one way.

Interviewer: Yeah.

Respondent: Walking, so yeah, and autumn generally. Yeah.

Interviewer: Yeah. OK. In the past two weeks have you walked or cycled for at least ten minutes at a time to get to and from places?

Respondent: Yeah.

Interviewer: OK. So that's walking or cycling?

Respondent: Walking.

Interviewer: OK. Could you tell me about what you did?

Respondent: OK. Different walkings. Walking in the last two weeks... walking to the shop takes more than ten minutes, but again that's... if I do it myself it takes me five minutes.

Interviewer: Yes.

Respondent: If I have the children it takes me longer so that's a bit... it's really only five minutes' walk but it's ten minutes when you drag the kids. But having said that I'm generally carrying, you know what I mean. At some stage during that I carry as well so it turns into a more physically... and there's a hill as well. There's a hill where we walk.

Interviewer: That helps too.

Respondent: So I have had my heart rate raised walking up the hill. And also in the last two weeks it's been school holidays so I've done more walking round town down to the sports centre which is probably about a 15 minute walk there and back, two or three times with the kids as well. Yeah.

Interviewer: So would you describe that intensity as steady, moderate or vigorous?

Respondent: Just steady.

Interviewer: OK. And the duration, you've let me know about it's at least ten minutes...

Respondent: Ten minutes to the shop.

Interviewer: ... to the shop. And about 15 to 20...

Respondent: To the sports... yeah, to 20 to the sports centre.

Interviewer: Yeah. And then the frequency of that.

Respondent: Frequency. In the last two weeks probably three times to the sports centre.

Interviewer: Yeah.

Respondent: But then probably about eight...

Interviewer: Yeah. So that's there and back?

Respondent: ... to the shop. Yeah.

Interviewer: Yeah. OK. And when did you do it?

Respondent: Different days.

Interviewer: Yeah. And why did you do it at that time?

Respondent: Because I could. Because, for the kids. Yeah. For the kids, mainly for entertainment for them and fits in with their day. Yeah.

Interviewer: OK. And OK. So... and you've told me where which means sort of coming down your dirt road, going along a main road for a little while.

Respondent: Yeah. There's always a little bit of main road action involved in that. Yeah.

Interviewer: Yeah. OK. And why did you do it there?

Respondent: To get the mail and then also to take the kids to the pool.

Interviewer: Yeah.

Respondent: And...

Interviewer: They've got a great pool.

Respondent: They do.

Interviewer: Yes.

Respondent: It’s fantastic.

Interviewer: So I dropped in and met [woman] and I was impressed.

Respondent: Oh you met [woman]?

Interviewer: Yes.

Respondent: [woman]’s great.

Interviewer: Yeah.

Respondent: Yeah.

Interviewer: ‘Cause a lot of women we’re talking to from the [town], [town] area all mention the pool. It’s... yeah, particularly if they’ve got kids too. Yeah, it’s great.

Respondent: Yeah.

Interviewer: And OK, why did you walk there? What attracted you to the place?

Respondent: Yeah.

Interviewer: Yeah. And you’ve already let me know that it’s with your kids. And is that something you usually do?

Respondent: Yeah.

Interviewer: OK. And does it change depending on the season or the time of the year?

Respondent: Not really. I tend to drag them no matter what.

Interviewer: Yes, especially down there, you never know when you're going to get the showers do you?

Respondent: When the fronts it's really easy just to tie them in between the rain because even if... and I just have them dressed. So it stops raining, we go now. And then you just wait for that gap you know.

Interviewer: Yeah. You're an expert.

Respondent: Yeah. Yeah, it's always going to come.

Interviewer: Yeah.

Respondent: Yeah.

Interviewer: And that's nicely thing about it it's always getting passed.

Respondent: Yeah.

Interviewer: OK. And if you'd wanted to, is there anything that would have helped you use more... use active forms of transport, like walking or cycling?

Respondent: I mean I use it as much as I can. I'm kind of restrained. We only have one car and it comes in to town, so yeah, I'm kind of restrained by walking, so it tends to be what I choose to do. Yeah.

Interviewer: Yeah. OK.

Respondent: Yeah.

Interviewer: Right. And at work, is there anything that could have helped you be more active at work?

Respondent: No.

Interviewer: OK. Now in the past two weeks have you been active around the house or yard?

Respondent: Yeah.

Interviewer: So at home inside or outside. Yeah. For at least ten minutes. Can you tell me about what you did?

Respondent: Gardening, weeding, digging over beds, mulching, which is dragging lots of hay, bales of hay. Yeah. Wood, moving tons of wood. Yeah. Probably be two tons of wood, well only half of it. I moved about one ton of wood in the last...

Interviewer: That's hefty enough.

Respondent: Yeah. Couple of weeks. Yeah.

Interviewer: And so thinking about those activities, the gardening and the wood carrying as well as obviously the housework stuff that gets done anyway, could you tell me the intensity of those activities?

Respondent: Wood moving, I mean moderate to strenuous, depending on how fast I choose to go, but yeah, it's hard work. Moving hay bales around tends to be only short spurts but still that's quite...

Interviewer: It's hefty isn't it?

Respondent: Yeah, it it hefty. Digging.

Interviewer: Yeah.

Respondent: Yeah, that's fairly extreme as well. Especially when the soils are so wet in clay, it's hard work yeah. And you get a sweat up and it's hard soil. Yeah.

Interviewer: And housework. What would you call the intensity of that?

Respondent: Oh steady.

Interviewer: Yeah.

Respondent: Yeah.

Interviewer: Yeah. And the duration of those activities, how long would you have done them for?

Respondent: Well housework probably be about an hour a day. And wood is probably half a day's work, so that's at four hours. Digging probably only about... oh actually probably an hour, an hour and a half. Mulching stuff probably about four hours work overall. Yeah, it feels like more but it's not. Yeah.

Interviewer: And how often would you have done those activities?

Respondent: One off for all of those that I've listed other than housework. Housework daily.

Interviewer: Yeah.

Respondent: Yeah.

Interviewer: And when did you do those activities?

Respondent: Tuesday again.

Interviewer: And why did you do them at this time?

Respondent: ‘Cause you need the sunlight.

Interviewer: Yes.

Respondent: Yes.

Interviewer: And the light.

Respondent: Yeah.

Interviewer: Particularly at this time of the year here. Yeah. OK. And who with?

Respondent: With my husband. Yeah. Yeah.

Interviewer: OK. And is that what you usually do or is it one off?

Respondent: Wood I only do it two or three times a year.

Interviewer: Yeah.

Respondent: So that was more one offish.

Interviewer: Yeah.

Respondent: And haybales I tend to do that about once a month. Yeah.

Interviewer: It sounds like you have a great garden.

Respondent: We do. We've got a mass... yeah, we grow all our own fruit and vegetables so, yeah.

Interviewer: Oh great.

Respondent: Yeah. Huge investment of time, but it's good.

Interviewer: Yeah. And does this change doing these activities depending on the season or time of year?

Respondent: Wood yes.

Interviewer: Yes.

Respondent: Even though we plan for it to be...

Interviewer: Uh-huh.

Respondent: ... an activity that gets done before winter, generally happens in winter. But all the others are...

Interviewer: Yeah, gardening's...

Respondent: ... all year.

Interviewer: ... yeah, with that sort of... you'd garden, you wouldn't be doing things all year.

Respondent: Yeah.

Interviewer: OK. And if you'd wanted to, is there anything that would have helped you be more active around the home?

Respondent: Better weather.

Interviewer: Fair enough.

Respondent: Warmer weather always means more active. Yeah.

Interviewer: Yeah. OK. Now just to check that we've covered everything, have you been physically active for at least ten minutes during your leisure or spare time? So this sort of catches up with anything that we haven't talked about.

Respondent: Yeah. Yeah, definitely.

Interviewer: OK.

Respondent: Yeah.

Interviewer: Could you tell me about what you did?

Respondent: OK. In the last two weeks?

Interviewer: Uh-huh.

Respondent: I go down to the sports centre on Monday and Wednesday nights, I've started doing that this year for a one hour like boot camp.

Interviewer: Oh.

Respondent: Which is just awesome, it's great. And I only go there because, this will answer one of your other questions, one of the... my neighbours started going last year, and got incredibly fit and lost a lot of weight and she takes me and it's after the kids go to bed. So I have the kids in bed and then I do that. I have someone pick me up and take me and then do that then for the hour.

Interviewer: Yeah. So does that help having somebody to do with it you?

Respondent: Absolutely. I wouldn't do it otherwise. And that... that's just basic yeah. And for her as well it works because it means...

Interviewer: It becomes a routine for both of you?

Respondent: Yes. Yeah.

Interviewer: OK. So tell me about what you did and its intensity?

Respondent: One hour of intensive cardio vascular workout and weights.

Interviewer: OK.

Respondent: Yeah.

Interviewer: And that's one hour...

Respondent: Twice a week.

Interviewer: Twice a week.

Respondent: Yeah.

Interviewer: OK. And that's in the evening. And why did you do it at that time?

Respondent: 'Cause it was the only time I have. Yeah. Free time. And because it's scheduled on at that time at the sports centre as well because that's when the class is.

Interviewer: Yeah. And I presume your partner then sort of he's...

Respondent: He's at home.

Interviewer: ... home looking after the kids. Yeah.

Respondent: Yeah.

Interviewer: Yeah. And OK, so what attracted you to the sports centre to do those activities?

Respondent: The fact that it was on and it was on then. Yeah. Yeah. The timing.

Interviewer: And OK. And the questions asking with... I know that you drive... get a lift there.

Respondent: Yeah.

Interviewer: But the question is why, what was that method of transport chosen, i.e. public versus public transport.

Respondent: Yeah, because there is no public transport.

Interviewer: Or because there is none.

Respondent: Yeah.

Interviewer: Yeah.

Respondent: Although I do catch public transport down there. I mean there are six or so buses a day.

Interviewer: Oh.

Respondent: Yeah. So I do, I do actually rely on public transport at times.

Interviewer: Yeah. But not necessarily to get through... around [town]...

Respondent: Not at night. There's nothing.

Interviewer: ... [town]

Respondent: No, not around [town].

Interviewer: No, and not at night.

Respondent: Yeah.

Interviewer: Yeah. OK. OK. So is this what you usually do?

Respondent: Yeah.

Interviewer: Yeah. And does it change depending on the season or time of year?

Respondent: No.

Interviewer: OK. And if you wanted to, is there anything that would have helped you be more active in your leisure or spare time?

Respondent: Yeah, I think... I mean it's... yeah we should be noted that when you've got small kids that leisure or spare time is... it doesn't exist. It just doesn't exist. And we do as a family go down to the pool.

Interviewer: Yeah.

Respondent: The spare time together. I don't necessarily get exercise when I'm at the pool.

Interviewer: No it's very different.

Respondent: No. It's exercising them you know, and we go to the park and that exercises them, I exercise the children. So yeah, it's a fine line there but it's not really exercise.

Interviewer: Yeah.

Respondent: Yeah.

Interviewer: Yeah. I understand. OK. Now I want to talk a bit about physical environment.

Respondent: Yeah.

Interviewer: So it's the last part of it. So we've talked about where you live and your physical activity. Physical environments are the environments that you live work and play in. And when we talk about them we're talking about all the physical things that surround you, the roads, the trees, the streets...

Respondent: Uh-huh.

Interviewer: ... traffic. And some of these things are things that people in rural, well in urban areas have said do affect them actually... now we're talking to people in rural areas they say it affects them too in terms of their physical activity. So I wanted to ask whether they're relevant for you too?

Respondent: Yeah.

Interviewer: Yeah. So can you tell me a bit about how easy or difficult it is to be physically active in your area?

Respondent: I don't think it's really that difficult 'cause there's always a place to walk. There's hills.

Interviewer: Yeah.

Respondent: And...

Interviewer: So there's access to areas to walk through your area?

Respondent: Yeah. And you know, they've just closed off [x] Road which doesn't sound like much, but in terms of getting exercise, I mean it's that what was once a busy road now has maybe ten cars that live on it. So you know... and so... and there are dirt roads that we can walk up that we have in the past but we don't with the children. And then if I was really determined to exercise that I could just walk up, I could do 15, 20 minutes in the morning and it would be done. Like if it was... I was that way inclined that it's always, yeah.

Interviewer: OK. And what makes it easy? Just having sort of areas that are accessible?

Respondent: Yeah, and no traffic I think. Low traffic areas, 'cause down there it's just the highway and I'd say one of the biggest impediments to not doing more is the

fact that I have to walk on the highway to get to the sports centre and it's not good.

Interviewer: No.

Respondent: It's logging trucks and yeah.

Interviewer: Yes. And do you have footpaths and...

Respondent: No footpaths. No footpaths along the highway.

Interviewer: Oh not in... OK.

Respondent: No. And I mean just small things, like my children, to teach them how to ride a bike I have to go elsewhere because there aren't enough paths and there's no straight flat roads.

Interviewer: No.

Respondent: There's no...

Interviewer: Yeah. 'Cause you live on a dirt track but then it's got a gradient as well.

Respondent: Yeah. It is. Yeah. Everything's got a gradient and there is no flat road.

Interviewer: The only flat road's the highway?

Respondent: Anywhere in [town] except the highway. Yeah. Yeah.

Interviewer: Yeah. OK. Now I'm going to talk about a number of... a few features of the fiscal environment. The first one's availability and accessibility of places to be

active. So if I was to ask you about places to be active in your area, what sorts of things would you tell me about?

Respondent: The sports centre. And also the park down at [x] Point.

Interviewer: Yeah.

Respondent: Yeah.

Interviewer: Yeah. That's a nice park, it's got a...

Respondent: Yeah, it is. They're really done... put a lot of work in to it, so it's quite nice. Yeah. And one of our neighbours just up the street has a tennis court that we're allowed to use, that we don't use much at the moment because it's winter and it's been wet and horrible and icy, yeah. Yeah. But that's available to us to use.

Interviewer: Yeah.

Respondent: And there's a lot of park as well just across the creek, so... just a really small one. Yeah. There's quite a bit really.

Interviewer: Yeah.

Respondent: Yeah.

Interviewer: Yeah. And you were saying also just the low traffic areas as well.

Respondent: Low traffic areas. Definitely.

Interviewer: Yeah.

Respondent: Yeah.

Interviewer: OK. So there are availability of places to be active. And access, are the places convenient, are they easy to access either cost, opening hours or actual being able to get to them with the traffic?

Respondent: Yeah cost is pretty good. Cost is pretty good. Opening hours for the sports centre are all over the place, which is hard, but I know them... they're trying to pitch to their markets and you know, it's quite difficult sometimes.

Interviewer: Yeah I noticed they sort of... I was trying to contact [woman] last...

Respondent: A couple of hours in the morning and then...

Interviewer: Yeah. I know. I was... I had to time it to pop in.

Respondent: 'Cause they do all the oldies stuff in the morning.

Interviewer: Oh OK.

Respondent: And then all the kids stuff in the afternoon.

Interviewer: And then the adult type stuff at night.

Respondent: At night.

Interviewer: Yeah. Yeah.

Respondent: So, but it's... I mean the pool closes at seven which is a bit of a problem. I'd use the pool a lot more; I'm a bit of a swimmer. But it's not... it's just not feasible for me at all, the hours that it's open. But they can't keep the pool open for me if you know what I'm saying, it's yeah. It's one of those things.

But having said that they're doing surveys at the moment and I don't doubt that if we lobby hard enough they would do it once a week for you because that's the kind of place, you know what I mean, it's...

Interviewer: Yeah.

Respondent: Yeah. It's good for that.

Interviewer: Yeah. OK. OK. And like parks you were talking about. Are they easy to access?

Respondent: Yeah.

Interviewer: Other than getting over the main road?

Respondent: Over the highway, yeah.

Interviewer: Yes.

Respondent: But even that's not a really busy highway.

Interviewer: No.

Respondent: Yeah, you never really have to wait to cross it. It's just that it's got the logging trucks on it which are quite big.

Interviewer: Yeah. And there's not much room both sides in terms of...

Respondent: Yeah. There's no room. Yeah.

Interviewer: Yeah.

Respondent: There are patches that are good but then there are thing patches as well. Yeah.

Interviewer: OK. And if you wanted to be more active... sorry... so does having places to be active influence whether you're active or not?

Respondent: Absolutely. Yeah.

Interviewer: Yeah. And if you wanted to be more active are there things or places that would help if they were available or more accessible? You mentioned the swimming would be very nice.

Respondent: Yeah. Opening hours. Yeah. Opening hours for those. But outside of that I'd say mainly depending on the weather which can't be done much about. Yeah.

Interviewer: Actually it's nice that you've got a venue like that in [town].

Respondent: It's fantastic isn't it? I know. It's a really good place. Yeah.

Interviewer: Yeah. OK. Destinations. If I were to ask you about places that you could walk to from your home, what sorts of things would you tell me about?

Respondent: The parks. [x] Point. The Jetty which is also good. It's fun. And the sports centre. Yeah.

Interviewer: Now the jetty's separate from the park is it?

Respondent: Yeah.

Interviewer: It's further over?

Respondent: It's just... yeah, the parks next to the jetty, like they're not far from each other.

Interviewer: Yeah.

Respondent: But the jetty I'd say is definitely a different experience. Yeah. We don't always do both like if you go to the jetty, you don't go to the park. You do one or the other.

Interviewer: Yeah.

Respondent: Yeah.

Interviewer: OK. And you've already mentioned neighbours and other people's places that you walk past and post in [town]. So does having destinations influence whether you're active or not?

Respondent: Oh completely.

Interviewer: Yeah.

Respondent: Yeah. Absolutely.

Interviewer: It's good to have someone... where that you could actually go to.

Respondent: Yeah, definitely. Definitely.

Interviewer: And if you wanted to be more active, would it help if there were more destinations within easy walking distance?

Respondent: Oh you know, what else could there be? I... like I say the, yeah. I think we're spoilt realistically within walking distance from my house to have what I have anyway, for a city let alone a country town.

Interviewer: Yeah.

Respondent: Yeah.

Interviewer: OK. Now neighbourhood design which is the manmade built features.

Respondent: Yes.

Interviewer: The non natural features.

Respondent: Yes.

Interviewer: Now if I were to ask you about those, what sorts of things would you tell me about?

Respondent: Oh it's just haphazard. There's no planning. There are no street signs. It's old... it's old school, there's... they've just built... I think the first footpaths got into the new area; they're subdividing a large area, which does have footpaths which is very exciting for [town]. But most of the kids... it's not in town, there's creeks through things and it's just poorly planned and poorly designed and horrible to walk around. There are no footpaths, there are no, you know, it's, yeah.

Interviewer: So what... so even with the shops there's not a footpath in that section either?

Respondent: No.

Interviewer: Yeah. So it's just sort of on the... what's it, you walk on the...

Respondent: It's a bit of gravel.

Interviewer: ... dirt verge of which some of it... and I've noticed as you said...

Respondent: It's pretty thin.

Interviewer: ... when I went down there, it's narrow. Yeah.

Respondent: Yeah.

Interviewer: What about the slope? Like women say, another one from [town] said it gets a bit tricky sometimes because she walks in to [town] and it's the cambers as well.

Respondent: Oh it's not really cambers on most of ours. There's a couple of spots where there's a drain and they're a bit dodgy, but yeah, the cambers not that bad.

Interviewer: And I presume with the dirt roads you obviously... you obviously don't have a footpath but do you have an area that you can walk safely along them?

Respondent: Well no-ones on ours at all. The one I go to...

Interviewer: Oh OK.

Respondent: ... and I... we generally walk down this dirt road and you have to cross a creek and they... like the kids use it and a few people in their ATV's and it's really muddy and stuff. That's fun for the kids you know.

Interviewer: Ideal really for them.

Respondent: Yeah. And there's pot holes in the dirt road and they're full of mud and so it's good. And there's only three or four other houses in this particular road and so... who have kids so it's not really a massive issue at all, the dirt roads. It's more the footpaths on the heavy traffic roads.

Interviewer: Yes. Yes. And what about lighting?

Respondent: Yeah, there's quite a few now.

Interviewer: You've got some street lighting along there?

Respondent: Yeah, there is. The street lights tend to be on the intersections...

Interviewer: OK.

Respondent: ... of wherever one street hits another.

Interviewer: OK.

Respondent: So it means I can see six from my house. And I notice there's a few more going up in the new area. So I mean it's dark out at night but there's none... there's one on the corner of my street up the road. There's none outside my house but realistically it's... I like the fact that there aren't that many street lights because we get really good stars at night. Very aware that the more light there is the more you lose the starscape.

Interviewer: Yes. And I think it's interesting with walk ability; generally would you do physical activity like walking at night?

Respondent: If I didn't have the kids. I used to work a lot at nights when I was younger and so I'm used to being out at nights. And I have conversations with my husband on how weird it is to be out at night now because it's not something we're

used to whereas before I was really comfortable about being out at night because I was out every night. So it's quite different not to be out at night. But that's mainly because I have children asleep and when they're kids you don't go out at night when the kids are sleeping.

Interviewer: And the other one is for walk ability, do you have streets that connect with each other rather than sort of like one main highway and that's it, you can actually sort of follow some roads around?

Respondent: Yeah, you can.

Interviewer: But, yeah.

Respondent: Yeah there are. There's more than just the highway.

Interviewer: Yeah.

Respondent: Yeah.

Interviewer: And if... now do built features of your neighbourhood, or lack of them, actually influence whether you're physically active or not?

Respondent: Influence the kids, some of the activities I do with the kids, but I just get out there and do it anyway. Yeah. Most... just like we'd, yeah, just do it anyway.

Interviewer: It would be nice but...

Respondent: It would be great.

Interviewer: ... it doesn't stop you?

Respondent: It would be great. I would probably walk with the children to the sports centre more if there was a really good footpath.

Interviewer: Yeah.

Respondent: But I just do it anyway. Yeah.

Interviewer: OK. And that's the story we're getting from women who live in rural areas. It'd be nice but you just get out and do it.

Respondent: And also... I mean it's, yeah... aren't many streetlights and so you realise that part of the things like that is that I don't want to live somewhere where there are a lot of street lights and footpaths aren't important to me, otherwise I wouldn't live there. There are other things that are more important. And the second you start bringing all of that other stuff in, no longer is it the place is it.

Interviewer: Yes. Yes.

Respondent: Yeah.

Interviewer: And if you wanted to be more active, are there any built features in your neighbourhood that would help?

Respondent: I mean yeah, there are street lights if I wanted to go out at nights. My husband has just started needing to become more active and he's started jogging at night. So it's possible. There are lights; there are enough street lights for a night jog to happen.

Interviewer: Yeah.

Respondent: Yeah. So.

Interviewer: OK. Now the other side of it is the natural features of your environment, the aesthetics which we were just starting to talk about. If you were... if I were to ask you about the aesthetic qualities or the attractiveness of your area, what sorts of things would you tell me about?

Respondent: Oh you know, beautiful hills, a combination of bush and grape fields and it's so green at the moment because it's been so wet. You know the river, the estuary, it's just fantastic. Stunning place. Our view is just all... we look West straight at the mountains you know, and that's... it's just stunning. Like we spend a lot of time out there in summer when you've got these great mountains. In winter it's... you get the snow, these phenomenally magnificent views of the snow you know, it's picturesque, just stunningly beautiful place to live. But part of that is because there are... it's not full of buildings and roads and street lights.

Interviewer: Yeah.

Respondent: And when I look out I see houses, but yeah, they're old houses. I've seen maybe ten houses built in the time... the four years that we've been there as well. Like it's... but I also see hay sheds and cows and my children pass the time sometimes when... watching the cows and the bulls and the cow hides.

Interviewer: It's great for the kids isn't it?

Respondent: And that's normal. It's good. Yeah.

Interviewer: OK. And do you think your local environment is aesthetically pleasing and...

Respondent: Yeah, definitely.

Interviewer: ... attractive near your home? Yeah.

Respondent: Yeah.

Interviewer: And do the aesthetics of your area influence whether you're physically active or not?

Respondent: Yeah. Yeah. Definitely. If I was somewhere that was ugly outside or I didn't feel comfortable in, yeah, I wouldn't want to be. But at the moment like in summer you just want to be outside, summer nights I want to be outside. It just drags you out you know what I mean, the house because you're in... and it's kind of like a natural room the way the town sits as well. Like when I'm outside, and we've got outside tables and chairs, it actually feels... the way the hills are and look feels like expand room walls. It's just beautiful. It's... you get a sense that you're not exposed because you've got these wonderful hills around you and it's... so we spend a lot of time outside because of the natural environment.

Interviewer: And if you wanted to be more active, are there features of your area that might help if they were more aesthetically pleasing or attractive?

Respondent: I mean the highway isn't that aesthetically pleasing. I'm just trying to think.

Interviewer: The natural environment.

Respondent: The natural... I'm thinking about the natural environment. Yeah. Around the river, we'd probably spend more time down the river if it was different.

Interviewer: Yeah.

Respondent: And that's a natural environment. And partly it's because it's either exposed, too close to the highway or cut off by fences or cleared.

Interviewer: Yes.

Respondent: So we'd probably spend a lot more time on the river I think if it was...

Interviewer: A bit more accessible in that way.

Respondent: Accessible and just kind of... yeah.

Interviewer: 'Cause there's little spots but the rest of it is either very close to the highway or it's owned privately isn't it?

Respondent: Yeah.

Interviewer: Yeah.

Respondent: Yes. So we'd probably spend... there's a bit down near the yacht club, there's a bit around there that's quite nice that we go to sometimes. But I think if.... yeah, there was more trees and stuff we'd definitely spend more time down by the water. Yeah.

Interviewer: 'Cause it draws you down there doesn't it?

Respondent: Yeah. Yeah. We like the water, yeah. We've got a couple of kayaks which of course we haven't used in the last two weeks, but...

Interviewer: Yeah. But it's something you would...

Respondent: Yeah. Yeah. We'd take the kids out.

Interviewer: ... use the kayaks. Great.

Respondent: Yeah.

Interviewer: Yeah. 'Cause it's sort of an ideal activity to have down there?

Respondent: Oh yeah. Yeah. 'Cause you can't swim in the water. Oh God, it's too cold.

Interviewer: Ahh.

Respondent: We go down the beach. We go down to [x] Beach quite a bit in summer, but this summer of course was just terrible for it.

Interviewer: Where do you go?

Respondent: [x] Beach.

Interviewer: Where's that?

Respondent: Between [town] and kind of [town] on the coastline.

Interviewer: Oh I think that was down that way, yeah.

Respondent: It's stunning. Best beach. Oh look, we've travelled Australia and it's one of the finest beaches in Australia.

Interviewer: Oh OK.

Respondent: It's just... it's just stunning. Yeah. So it's still a 15 minute crappy drive along winding things, you know you're car sick getting there, but it's just so worth it. It's just such a beautiful environment. So yeah.

Interviewer: But if you think well you know, 15 minutes elsewhere and...

Respondent: Oh absolutely. It's nothing.

Interviewer: In Australia’s...

Respondent: Nothing. And you have it to yourself generally, or with just a couple of other people and it’s just stunning. Yeah.

Interviewer: Yeah.

Respondent: Beautiful place. Yeah. And we also go round a bit to the [town] kind of side of the river along those bays there, like, what is it? [x] Point, desolated... around there.

Interviewer: Yeah, that’d be a... yeah.

Respondent: We take the kids over there and spend a bit of time over there and fossil hunting and stuff. So there’s really... I mean, and part of it is because of the natural beauty.

Interviewer: Yes.

Respondent: And it just drags you out to do that.

Interviewer: Yeah.

Respondent: If it was the city we would not be doing that.

Interviewer: No, no. It’s not in the last two... couple of weeks but just following up the kayaking which is usually in summer I gather.

Respondent: Yes.

Interviewer: Yeah.

Respondent: And autumn.

Interviewer: And autumn. So how often would you go out kayaking?

Respondent: Again it's so weather dependent.

Interviewer: Yeah.

Respondent: It's so weather dependent.

Interviewer: Yeah. And you would only really have the weekends to do that.

Respondent: Weekends generally. So...

Interviewer: Unless it's a very long summer evening and the holidays or something.

Respondent: Absolutely. So yeah, it varies. Again I couldn't say and not on a weekly basis.

Interviewer: Yeah. Sort of once a month.

Respondent: About fortnightly.

Interviewer: Oh OK. Once a fortnight.

Respondent: I try to get out there and do it.

Interviewer: Yeah. Cools you down there doesn't it that river? OK.

Respondent: Yeah.

Interviewer: Now the last area's safety. And if I were to ask you about safety in your area, what sorts of things would you tell me about?

Respondent: When I think about safety generally I think about human threats, I don't think about physical safety.

Interviewer: It's a pretty safe community in that way.

Respondent: Yeah. But I mean even... I know people in town who are the only ones who let's say... I mean we're [occupation] so you know what I mean, I look at people and people's behaviour. And there are only two or three people in the whole community that I've come across who I would be a little bit concerned with with the behaviour, so I assess those risks and I know who they are, where they live, I know what's going on. So I mean I feel quite safe because I know who the major risks are. And I don't find them to be a big risk. And because you're enmeshed in a neighbourhood and community you know everybody. So... which means that part of my... I suppose the big thing then would be the safety, for instance getting to... at night time getting to the sports centre. I wouldn't walk that at night because I have to walk along the highway.

Interviewer: Yeah.

Respondent: But I would walk my own streets at night. If I didn't have to cross the highway it wouldn't be an issue.

Interviewer: And that's more an issue of road safety isn't it?

Respondent: Road safety. And also...

Interviewer: And sort of...

Respondent: Oh because I'm a [occupation]... I've been working on the [workplace], I know, I know that there are people down that way who are not mentally well and I know that there's a lot of, kind of, you know what I mean? There's a small core of people who pick people up from the side of the road you know, that that happens and, yeah. And there's a lot of trucks and that's dangerous. So yeah. So night time I wouldn't walk to the sports centre. But having said that, in summer, in the twilight I do.

Interviewer: Yeah.

Respondent: Yeah.

Interviewer: It's light enough.

Respondent: Yeah. And because there's no... there's a long stretch with no houses and that kind of thing, you have stretches. But outside of that I walk around [town] no problems.

Interviewer: Yeah.

Respondent: 'Cause...yeah.

Interviewer: Yeah. Yeah. Fair enough. And did... so does personal safety influence whether you're physically active or not?

Respondent: Not really, no.

Interviewer: No.

Respondent: No.

Interviewer: Yeah. If it did at all it would be at night.

Respondent: At night, yeah. If for instance my friend is not going to pick me up to take me to the sports centre I generally don't go, even though my husband probably could drive me, you know what I mean? I just, yeah, because I... mainly because I would not walk it myself and the effort that would need to go in to getting me there with the kids and everything is just... outweighs the benefit.

Interviewer: Yeah.

Respondent: Yeah.

Interviewer: And does road safety influence whether you're physically active or not?

Respondent: Yeah. Yeah. Definitely. Knowing the difference now that I do walk to the top of the hill occasionally because there is no traffic on it. And walking to friends houses that live on that road is now... with the children I never used to before because there was so much traffic I do now. So... and not walking on the highway.

Interviewer: But that's the road that has been cut off?

Respondent: Yeah.

Interviewer: Yeah.

Respondent: Lots of people complained about it but it's been fantastic.

Interviewer: Yeah. And then you were going to say about the highway [indistinct 45:39].

Respondent: Highway getting to the sports centre. Yeah.

Interviewer: Yeah.

Respondent: Would be the other big one. So it does definitely impact.

Interviewer: Is there times when it's busier than others?

Respondent: Day time. Yeah.

Interviewer: Yeah. Any time during the day…

Respondent: It's really quiet at night.

Interviewer: Yeah.

Respondent: Which is another issue.

Interviewer: Yeah.

Respondent: You know, because the busier road is at night but realistically in terms of personal safety because there are more people, so.

Interviewer: And I gather it would be the logging trucks that would go during the... they would be on the road in the day time anyway.

Respondent: Day time. Yeah.

Interviewer: OK. So if you wanted to be more active, are there personal or road safety issues that if they were addressed could help you be more active?

Respondent: Oh absolutely. Like a by-pass.

Interviewer: Yeah. Yes.

Respondent: Oh 'cause I was a bike rider for many years, that's what I did.

Interviewer: Oh yeah.

Respondent: And I've been hit by cars on several occasions, so it's a massive thing. And I could easily ride to [town] but I don't. I can put the car, the kids on the back of the bike and ride to [town] and I don't because it's just not safe.

Interviewer: No.

Respondent: You can't ride it. Can't do it.

Interviewer: No. When I moved to a rural area I gave up riding.

Respondent: Bike riding. Yeah. Oh we can't ride anywhere, it's just...

Interviewer: Because it's just too dangerous.

Respondent: ... dangerous.

Interviewer: Yeah.

Respondent: Yeah.

Interviewer: Yeah.

Respondent: Not going to happen.

Interviewer: No.

Respondent: No.

Interviewer: No. And unless they sort of look at another alternative those... that roads quite narrow and twisty.

Respondent: Oh it's such a bad road. And the speed limit. And when people ride their bicycles on it, it's just dangerous for people, only because the reaction of the the drivers to a bicycle it just dangerous with logging trucks, just is dangerous. It's a dangerous road. Some crazy stuff goes on all the time; it's a very dangerous road.

Interviewer: So the only way you'd cycle then is to actually put the bikes in the back of the car.

Respondent: Yeah.

Interviewer: And drive to a place.

Respondent: Yeah. We go to [town] or [town] . Yeah.

Interviewer: OK.

Respondent: Long distances to ride bikes, to find somewhere...

Interviewer: OK. So where in [town] would you happily ride?

Respondent: Down around the river.

Interviewer: OK.

Respondent: So...

Interviewer: So there's some dirt roads.

Respondent: ... the kids can ride on the track, but then there's... down on the river there's long stretches where generally there aren't cars and things that we can ride.

Interviewer: Yeah. So off the main road?

Respondent: But it's not pleasurable. It's not my kind of riding, but I do it with the kids.

Interviewer: Yeah.

Respondent: Yeah.

Interviewer: Yeah. And [town] would be the same would it? In terms of the areas.

Respondent: Yeah, the kids park, the really big park down there.

Interviewer: Oh yeah.

Respondent: Yeah. And take the kids there. It's got a special kids riding area.

Interviewer: Yeah.

Respondent: Yeah.

Interviewer: So it's... I've been talking... been talking with people up in [town] as well.

Respondent: Yeah.

Interviewer: And not only in [town] but the surrounds so that... in the more rural areas around, and they were saying oh we've got this bike track, a walking track from [town] to [x] Beach.

Respondent: Wow. Awesome isn't it?

Interviewer: Uh-huh.

Respondent: I mean and I've lived in other rural areas that haven't had high traffic and I used to ride my bike everywhere because there wasn't the high... there wasn't just one road, it was on the mainland, so it's very different communities and road system.

Interviewer: Yeah, it is different. Yeah.

Respondent: A different topography...

Interviewer: Where it's much more likely to be a... one main road system here.

Respondent: Yeah.

Interviewer: I've noticed that, not only where you live but [region] is the same. Yeah. Which is the other study area.

Respondent: Whereas I used to really enjoy bike riding a lot in the country, but down here it's just not going to be, it's not... the consideration's not going to happen.

Interviewer: No. OK.

Respondent: Which is really disappointing 'cause I love it. Yeah.

Interviewer: Absolutely. Oh that's really important. So just before we finish, is there anything else that you'd like to say today about where you live, your physical activity or the physical environment that we haven't covered?

Respondent: No, I think that's pretty much it. Yeah.

Interviewer: Thank you very much.

Respondent: That’s OK.

Date: 4 October 2011

Duration: 48 minutes, 41 seconds

Interviewer: [interviewer]

Transcriber: [transcriber]

So we are talking a bit about what it was like to live around [town], your likes and your dislikes.

[town] is a great little town, but like all country towns we are very very restricted by what we've got, what we can do. Um, it's really really hard to deal with council all the time, especially when – I work with two areas, youth and the aged, and youth is a real forgotten problem in the area. And of course what is happening now, because of the unemployment we are getting more and more people finding it hard. Um, through the Community Centre we have now a food pantry, which we supply food through Centrelink. And we are handing out more and more and more. You know there is people struggling. We are getting requests daily for help that we can't even do, you know. And that's the negative to the town. The positive to the town is that there is a good community feel in the town. Ah, we've got the best group of heart walkers in [state] and the second best in [country].

Yes, so I hear. Yeah. Which is very impressive.

Yeah. Um, so we've got, there is a lot of good in the town, but there's a lot of negatives. And with the forest industry like it is now, we just don't know where the negatives are going to go.

No, what's going to happen.

And it's something that as working with the Community Centre down there, I think we are going to have more and more problems. And working with older people is going to become more interesting too. Because it's going to get harder for them to operate. I mean even having walking places, people don't – and you know there is a lot of people who are not exercising because there is nowhere for them to walk. The roads up our way, we live 4 ks out of town, the roads are narrow, we've got [1:43] on the road and nowhere to walk along them.

Yeah, because you were saying that you live on the [1:50] road and

And it's narrow.

It's quite sort of narrow.

It's tight, we've got [1.53] running up and down.

Yeah. And you live on how many acres did you say?

We live on three quarters of an acre.

Three quarters of an acre.

Off on a side road, on a dirt road. And it's really good. It's a great area to be. But, yeah..

OK. And your reasons for living there. You were saying that you were [state] but lived on the mainland for a while. Why did you pick [town] when you came back?

Well [town] [state] is my home. Um when we came down because my Dad was ill we found this block of land that we liked. It was a creek, permanent creek. It's in a nice area. It's quiet. Um, well we like our isolation a little bit. We've always lived in areas where we had people around us, and this a tree change I guess. We've now, I mean we've got neighbours around us, but they are not looking in on our property so – we'll class it as a five acre farm set.

OK. Now, as I was saying most of this research has been done with people in urban areas and um, they often refer to their local neighbourhood. And I'm interested in understanding what the term 'neighbourhood' means for you, living in a rural area? And indeed if it is actually relevant?

It is.

What does it mean to you?

Well because we live in a rural town, everyone knows everybody. When we first moved into the town and knew no-one, "oh you're the people that bought such and such place" you know like. And they refer to someone who is probably died ten year ago and but they still refer to their house. And um, this sort of, this is the energy. So once you know where you are and what you are capable of, you are accepted in the town. Ah, probably takes 30 years to become a local. But you are accepted in the town for what you are.

How long have you been living there then?

We're back there – we've been back there eight years now. Seven, eight years. We came down for my Dad. He got sick, he died, we stayed. And um, we still have a lot of contacts and friends on the mainland which we stay in touch with. We still do the [4:14] backwards and forwards. But when you come over the saddle and you're heading towards [town] and you are looking down to the see the trees and then you hit [town] and you follow the river down, you know you're home.

Yeah.

Yes. It's the peace, it's the serentity, it's – yeah.

So OK, with the neighbourhood, is there a distance around neighbourhood? Like is it sort of the immediate people or is it the whole of [town]? What would neighbourhood be for you?

Within the [region] you've got communities. [town] is a community.

That's the other question I was going to ask you.

[town] is a community. [town] is a community. And it's really really hard to get any interaction between communities a lot of the time. The issue that I have as someone who works with the youth, is that there might be a lot of stuff happening in [town] but people don't want to travel to it. They want to just be able to slip out for five minutes to do it. You know? Um, transport is a big issue down there. I guess we've got all the rural problems as far as you know. Um, we are lucky that [town] has got two community cars, we've got the Metro [5:25] bus now which is in our system. And we've got, there's a bit around, but it's still an issue.

So what does the term "community" mean for you then?

It's working with the people in the community. It's working to achieve things that we need in the community.

So that would be all of [town] and the [town] area.

[town], [town], even to [town] Um, just that –

So if you put a distance around that –

Probably 8 ks.

8 ks.

8 ks either side of [town].

Now is "neighbourhood" different?

No.

No, it's the same. So it would be the same area.

I guess it's, if you go and live in say [suburb], but at [suburub] you've got [suburb] – and they're different neighbourhoods. But with us, it's just because we've got 15, 1600 people down there in that area, in the whole of the [region], um round [town]. But basically you've got 15 – that's your community. That's your neighbourhood. That's the people you live with.

Yeah. OK. That's great. Now I want to talk a bit about physical activity. And as I was saying, it's very broad. So what does the term "physical activity" mean for you?

I am still fairly active. Touch wood. Um, I do a lot of stuff. I'm a motor sport fanatic. And I'm a fire rescue trainer and marshall. So I spend a fair bit of time on [raceway] at weekends and operate out there. Because I won't spend a lot of time on my feet anymore because of arthritis and things like that. But I am very, very active.

So that restricts you a bit, the arthritis?

Um, I find it really really hard sometimes down at home. We're working in an area that's wet and grassy. And sometimes you are walking through the top paddock and you got to come back and my legs start to ache. And it's a case of take your time. And I'm a [x]-year old and I've had arthritis since I was 30. So you've sort of got to be very careful about what you do, you know? Several years of riding around in trucks with horrible seats and yeah [7:36]. So you are restricted as to what you can do but you need to have the ability to be able to drive and do things yourself. You need, it's really really hard. Like we've got a fairly good bus service from [city] to [town]. But we live 4ks from [town]. So you catch a bus to [town], well you still have to get to the bus stop. And that's the sort of stuff you've got to look at. Whereas in the city the bus probably goes door to door. Or two streets from where you live. And that's the biggest problem with the bus service is people have to get in their car to go and catch the bus, so why bother?

Yes, it's that stuff of having to get into the car to do things. So physical activity is about doing things?

Yes. That's it.

Do you see yourself as physically active?

I do.

Yep. And tell me how, why you would say that?

I don't actually work with any of the organised groups as far as activities and things like that. Except for the Community Centre do a lot of stuff. But I do a fair bit of walking in the respect that I'm at the tracks on the weekend, I'm moving around all the time. Um, at home there's a fair bit of walking to do to feed the chooks, to do the stuff.

And that's the thing about arthritis isn't it? It's about movement isn't it?

That's it. But when it nails me, that's the hard bit, you know? Ah and it's only the fact that we live [hot water bed 8:55] and keep the chooks the movin, and like you know like but…

So you've really worked on how you can um, best manage your arthritis?

If they ever put that heat rub in a 44 gallon drum I reckon I'd buy one.

Yes. And where does physical activity fit into your life? Is it of no interest, or is it a priority, is it a high priority?

Wherever I can I like to be active. Um, if my body tells me you need to sleep, then you need to sleep. If I don't feel like going and doing something, then I don't. Um, I won't work full time anymore because of that reason. Because ideally I want to do it at my leisure. But if I don't feel like doing it, I'll lock the door and I'll stay at home.

Yeah. So um, just one of the things with the research, is we often look at physical activity is any activity that lasts for at least ten minutes. That's the evidence of health benefit effect. And it causes your body to work harder than normal, so your heart rate might be up. You might breathe a little heavier, you might feel a bit warmer. Um, so thinking about that sort of physical activity over the last couple of weeks – I'll just go over just some parts of your life. Um, and not to segment your life but just make sure that we cover everything. Now as you were saying, you'd been retired, you are not employed at work, but you do do quite a bit of work by the sounds of it.

I work with the [x] organisations very heavily.

Yes. So thinking of those, the things you've done in that area over the last two weeks, um have you been active in that work?

Yes I have. I've done a bus trip for the [x] association. And that involves, you know when you load them on the bus, you help them up the stairs, you come at the other end, you take the step, you get them down, you walk with them. A lot of them are on frames, so you physically, you've got to work with them all the time, or on walking sticks. We actually did a trip to [x] Bay on the ferries, and we had to walk a distance up to the restaurant and that.

Yes. It's quite a bit of a steep ramp I've heard.

Dealing with the youth, when we have our, I had a youth night down at the [11:41], and I turned out um, we did chicken kebabs and fairy floss. So I had the fairy floss machine and we were running that all night. You know, two or three hours of solid movement all the time.

Absolutely. Absolutely.

And the legs start to scream about half way through the night. Even though the kids are helping you've still got to be on your toes, because you're the supervisor for the evening. So yeah, so yeah I'm active all the time.

OK. So thinking about those couple of activities, those two examples, but um can you give some idea about the intensity of that exercise? Is it steady, moderate, or vigorous?

Um, steady to moderate most of the time. OK. Occasionally vigorous.

And how long would those activities be done for?

Oh, probably two or three hours.

At a time?

Yeah. Um, even when I at the race track on the weekends, we sit in the truck for a few hours and then we get out and we might have a vehicle recovery.   So it's half hour of hook up, get them into the pits, undo them, go back, sit down again. So yeah, you're looking at, you're looking at one to four hours of fairly [12:48] activity.

OK. And the stuff at the weekend is more in your leisure time?

Well it's all leisure time these days really.

Oh, OK. Well we'll pick that up as we're talking. And how often would you have done those activities? You were saying they were three to four hours at a time. In the last two weeks?

Oh, lately, lately it's been oh, two or three days, every week. Um, even, even to the extent of doing, doing meetings. Yeah, because you've physically got to organise – I organise meetings and so you've got to go in, you've got to set up tables and chairs. So there's exercise there all the time.

Yep. Yeah. Now, and what time of the day? Is that a particular time of the day you would have been …

Yes. Usually from midday till about four or five.

But you were also saying there are some evenings.

Yep. And sometimes I like to, if I've got the flexibility of doing things, I would do them in the morning sometimes, because the arthritis can [13:58] I got up three flights of stairs to you this morning.   But that wouldn't have happened at four o'clock this afternoon.

No. Is this what you usually do or is it one off? The activities you've described…

Constantly.

Yes. And does it change depending on the season or the time of the year?

Winter it probably slows down a little bit.

Yeah. But not much.

Because the weather, it slows down what we can do. Um, you wont take people out on the bus on a really wet day because it's too hard to get them from [14:33] and if you do they're

slipping and sliding. Um this time of the year it comes in really heavy, and we are starting to feel it over the last month, it's becoming constantly go, go, go. I was sitting at home the other day, a nice quiet day about to watch sport on tele – and the alarm system went off at the Community Centre and I was the first called.

Oh no. That's the thing isn't it?

Straight away, snap – into the van, down to the Centre, check that it was all OK. And it, an hour later you come back home. But you know, that's just constant, so yeah.

OK. In the past two weeks have you walked or cycled for at least ten minutes at a time to get to or from places?

Cycling no. Walking yes.

Yes. OK. Can you tell me about what you did?

Um, yeah. Um, I spent three hours the other day walking around the town, talking to people. I've got a – my friend is organising a dance and I've been out putting up posters, talking to suppliers, chasing bits and pieces. I had about three hours the day before yesterday. A couple of days before that I had a couple of hours in the town chasing round doing stuff So each day I'm doing a couple of hours, yes.

So you'd walk around the town for activities? I presume because you are 4k out you probably get in your car to …

Oh I drive myself yeah. I might get to town [16:02] – When I was at the Grand Prix this year I was a little bit sore one of the days and the sector master said to me "are you going to be able to get to that fire over there" and I said "yeah, but you might have to carry me back" sort of thing.

So thinking about those two activities you've talked about?   What would be the intensity? Would it be steady exercise?

Yeah, steady, yep.

And the duration? Just a bit of a guess?

Couple of hours.

Yeah. And the frequency in the last couple of weeks? How often might you have done that?

Too often. As I said, the last month and this month and November are my busy times.

Yeah, they're really, really busy.

It's constant. Um, whereas in winter I might only go out twice a week. OK? One, because it's too wet, one because I'm not feeling up to it – the arthritis isn't letting me. So and like

when the sun comes out and the lawn is dry, I spend three hours on the ride on mower. Now that's not walking, but it's moving, bouncing.

That's the next thing I'm going to ask you about. But um, just to finish this one off, um how often would you have done these activities of walking around the town in the last two weeks?

Oh twice a week, sometimes three times, depending what I've got to do.

Yep, yep. Um. OK. And would you be doing that alone or with others?

Well alone. But in conjunction with others. I don't walk with an organised walking group or – because it's something that I can't plan on, because if the walking day is a day when I don't feel like it then it doesn't happen. And I have three or four other friends that are like that. They're not into walking groups for that exact reason. That, as much as they love to walk, they have to do it when they are able.

Able to. Well that's a really important point. And if you wanted to is there anything that would have helped you um walk more often to or from places?

Yeah, probably my attitude.

How do you mean?

Well even though I'm active, I consider myself lazy. I won't physically walk unless I've got something to do. Um, because I think –

That's a really good point actually. I think it really helps when you do have something to do.

Yeah. If I've got an objective or a goal, I'll do it. But if it's just I'm going to go for a walk because I've got to go for a walk, no.

No, it's not – no. Especially when you are that far out of town, you don't sort of just, and the roads are narrow you just don't get out and walk on the road.

Um, when I'm at the track now, some of the [18:54] I'm doing, I actually run the [18:57]course, and I'm in at town two stories up. And it's all stairs. So I'm up and down the stairs probably half a dozen times a day. So even then I'm getting my exercise. When I'm [19:08] I'll walk the length of the pits.

Fair enough. And um, OK, so in the past two weeks have you been active around the house or the yard, at home inside or outside for a least ten minutes of the time.

Oh yeah, I've mowed some lawns out the front with the hand mower. I've built a couple of fences. I've got a very sick dog and she's old and she's about to die soon. And we just don't want her to stray onto the road. So we put a little fence up for her. And um, I feed the

chooks every day of course, and wander around and talk to me babies – I've got three geese on nests at the moment. So you get in to check them and make sure that they are OK, because they settle into their little holes and don't come out once they're on the nest. So you got to make sure they've got food, you know. So you know, walking around. And because I'm walking on mud at the moment, it's pretty um horrific.

Yeah, it's not much fun is it? So looking at those activities, um the intensity of the exercise, would it be steady, moderate …

Oh, all moderate.

Moderate? OK.

Yeah. Don't do anything at high speed anymore, unless it's - the other day when I was chasing the geese because he was attacking the chooks, and then I fell over in the mud.

You don't need that at all? And so how long would you have done those activities for? Because some, you do daily don't you?

Oh to feed the chooks and the animals is an hour a day in the afternoon. Um, and then in the morning you go down – it takes a couple of hours a week to maintain the animals and look after them.

Yep, with all the activities. OK. And um, what time of day would you have done those activities?

Well usually the early morning run is about 8 o'clock. Ah, the afternoon we'd feed them about 4 o'clock.

OK.

It's nicely spread out.

Yep. And ah, who would you have done those activities with? By yourself?

Me and the animals.

The dog helps out.

No, the dog can't walk – she's old, and she's got a heart condition. Usually me big black cat runs down the back with me. We've got a seven year old big black cat that runs everywhere we go,

And OK, so this is what you usually do, and does it change depending on the season?

That's something you've got to do every day. Um, the only thing that changes with the season is probably the extra activities that you get up to. Like, there's no way known in the

middle of winter we jump in the van and go for a drive and go for a walk along the beach, but we might do that in summer sort of thing.

Yeah. And if you wanted to is there anything that would have helped being more active at home?

Again I'll say me. Um, there's always things to do. It's not like when you are living in suburbia – there's always things to do you know. There's always repairs. There's always stuff to be done. I've got three quarters of an acre to mow regularly. I'm helping everyone in the community, a lot of the time, even in winter, you know? I've got a little old lady who lately I've taught to use a computer.

Oh good on you.

So every now and then she rings me up –"oh I've got some trouble, somethings gone wrong". So, and again that's in the car, drive up, walk to her –

So there isn't anything really, I mean because there's always things you do anyway.

And most of the guys that I talk to down there, because we have a little meeting at the [x] Church, once a month, called our secret men's business. It's just a group of guys getting together for lunch and a chat. And 90% of the guys down there, of different ages, but they're all fairly active still. You know, and a lot of them are looking after older, oldish wives. A couple of their wives have died. But they're all still active. They're all still doing things. They're all out cutting fire wood, they're all you know, yeah.

OK. And um, OK have you been physically active for at least ten minutes during your leisure or spare time? So that's anything else we haven't covered.

Well that's, my leisure time is my motor sport time and that's always active. Because you have to be, it's a physical activity – and I do it for that reason. I've been doing it for 35 years. And I know it, I'm comfortable with it, I know my limitations. I went to [country] last year with motor sport. Went over to the Grand Prix and actually helped set up the track over there. And that was long days. I worked from 7 in the morning until 7 at night. Um, and we were setting up the track, training [nationality] – and it was pretty full on days.

Yes, quite exciting too.

I missed out this year. I was disappointed.

Oh. So, thinking about those activities, can you tell me about the intensity of them?

Sometimes very intense.

So they might be quite moderate to vigorous?

It's an emergency situation. You have a guy go into a wall in a racing car at speed – you work hard. Um, if nothing happens, well we might have a check, we might have to do a pit load a few times. There's always some activity. But if we have something goes [24:38] – it's an hour.

So thinking of the last two weeks, um how long would you have done that activity for?

Um, within the last two weeks I did a three day meeting and we run it Friday, Saturday and Sunday. And um, that was full on. We had a heap of cars from the mainland, and we had to run it all.

So that's what, in the last two weeks?

Yes.

OK.

And again this weekend, and then two weeks after that. And then two weeks after that.

It's fairly regular then isn't it?

Yep.

And you do that with others.

Yes, I'm part of the [x] Club and the [x] club [25:23]

And is this what you usually do, or is it one off? Yes it is. And ..

35 years.

And it doesn't change depending on the season or time of year?

We have a little bit of lay time over the winter. There'll be still some small event, you know? And then Christmas through till the end of January beginning of February we have a little bit of time. But it's there basically all the time. When I was in [city] it was 52 weekends a year, or 50 weekends a year. And I was working up at to five days a week at the race track.

OK. So I went to talk about – the last bit of it – is your physical environment. So when we talk about physical environment, it's the environment you live, work and play in. And when and um, I mean things, physical things that surround you. So it could be trees, roads, houses, shops, anything like that. And some of these things are things that people in urban areas have said might be important to their activity. So we're interested in finding out whether that's the case rurally as well. Um, can you tell me about how easy or difficult it is to be physically active in your area? And why that might be the case.

It's probably a little bit difficult. Um, because of the restriction of access. Um, we've got some footpaths in the town. But there's a lot of areas that don't have footpaths. Ah, to

actually um, to actually get around our area is fairly hard. I mean um, and I've said before, to walk from our place um it's all right on the dirt road, but once you go up on the main road you wouldn't walk there for quids. You know?

No. It's a main, narrow, road.

It's runs out to the airwalk and it's narrow and it's tight.

Yeah it's busy.

And we've had two or three deaths on it. And there's trucks and there's log trucks – and so you can't really walk along it too much. Um, so we're restricted in that if you've got to walk somewhere, you can't walk too far. Or you've got to walk on the back roads, which the dirt roads have got a [27:34]

Yeah, so there are other dirt roads, OK.

Yeah the dirt roads you've got a [27:39], so when you're walking, you are walking unevenly.

Yeah, which is very – it's very difficult to walk that way. And it's also very uncomfortable.

Yeah, um normally if we walk we drive down to the local regatta grounds and we walk through the grass there and wander around there a bit. Um, they're trying to get more walkways in and they are slowly and surely putting in walkways for people. But again it's all budgeting.

That's right. So um we're going to go through a few areas. First of all, is availability and accessibility of places to be active. So if were to ask you about places to be active in your area what sorts of things would you tell me about?

Um, well probably there is the [x] Sports Centre, it has got a swimming pool there.

That's only about 6k away.

The problem is that it is booked out to groups so much of the time, it's really really hard for someone to just walk in and have a swim. I've got a friend who needs therapy, but he likes to do it on his own. He can't get a spot there. He's got to go to [city] to do it.

Now I've heard that too. A few other people have said about, it's a bit tricky around the availability. It's a great resource, but availability is a problem. And I've also heard the comment that if you are actually needing to do um certain exercises, whether they be aqua aerobics or whatever, you actually need a warmer pool. It's a bit cold.

There is guy that does aqua aerobics Mondays and Thursday mornings at 8.30. And he has an hour – he does that twice a week. The schools use it a fair bit.

So it's not really available in the daytime.

The big issue is that, to me, I've come from seeing a lot of other stuff in other places, and your sports centre should be open from early in the morning until late at night, to give everyone access to what they want.

And it's not is it? It's closed.

Yes. And then over five weeks over Christmas it was closed. Now,

If people have a bit more flexibility in time with the kids in school –

My kids – my youth group, they have to travel to the Centre to get a swim, but they can't always get in because of activities. Now there's no swimming pool down there, that they can just access. There used to be an outdoor one, but they filled it in.   The nearest one is [town]. Or the river. Which is dam cold on a good day. But I guess the problem is that even with um the restaurants and everything down there – but they are all open in that little time frame, 9 till 5 or whatever it is. There are some exceptions, don't get me wrong, but instead of planning, especially in summer with the tourism trade, to be open from early till late – they don't all have to be, they can work it between them surely – there's just not the access there that people need, no. Um, to get a coffee at 7 o'clock in the morning is just not on. I mean people go to work at 6 o'clock down there. And then to get a coffee at 7 o'clock at night! No hope in hell, you know. And when you got tourists that come from the mainland that are used to that sort of access, it restricts them.

Especially if they've been out and doing something. They come, [town] is the centre, they come back into.

And unfortunately, that's a big problem in our area. And it's something that needs to be addressed. And how, I don't know. The Council don't seem to want to know about it. It's actually a real big issue. So as far as facilities, it's really really hard. At the Community Centre now we have yoga, we have um, at the Community Hall they have tai chi at the moment. Which are all organised operations. But there is a lot of people don't like organised things. They like to do their own thing at their own speed. And that's where the problem is.

Yeah. OK. No, that's well put. And um what about things like walking tracks and parks and things like that?

We've got a really really nice park in the centre of [town], the [x] Park.

It's quite a big one isn't it?

Yeah, and we've got a platypus walk and there's good footpaths. Ah, there's a BBQ area in there and it's used fairly heavily. And there is a sort of a skate park, an old one in there which the kids want upgrading. We are fighting at the moment to get that done. Um but apart from that area there's not really anything else. I mean even down at the picnic, at the regatta grounds, there's parks there, um but there's nowhere that's even for people to walk. You know, there's no prepared tracks, or there is nothing – like especially older people who need that area to walk in that is comfortable. There's potholes and you know.

Yep. So do you think having or not having places to be active in influences whether you are active or not?

To a degree. Yeah. Um, I think there is a – there's actually a good walking track at [town], ah halfway through [town] and at [town]. And it's done on crown land around [x] Bay. So the local, a little group of people got together and they just made this walking track – and it's only about a kilometre through the bush along the waterfront. And it gets used fairly extensively. People park their cars and they walk along it, they walk back, you know. It's only a kilometre but people use it. And it's nice.

Because people would use it because it's easy.

So the more we can get the more they'll use. Um, I actually went to a meeting for the Council, meeting all the new Councillors last night. I was very disappointed there was only six Councillors turned up. And three of them aren't in yet. Um, and I just find that there seems to be a really lethargic laid back "oh I'll get in again". Anyway that's beside the point.

So you actually see that if you wanted to be more active there are things that would help, like more walking tracks. And also the Sports Centre.

That's a really good one in [town].

Oh is it?

Yeah, it runs around the back of the flood plain. Runs from the foreshore right round the back of the town. They put it in. And it's a good one. And they put bridges in and that. So they are doing it but slowly.

Yeah, yeah. So it's keep up the good work, more of it. And also the Sport Centre, um that whole..

The Sports Centre is a sore point.

I gathered it might be. I popped in to talk with [manager] at one stage about advertising the study. But yeah, I gather that it's been tricky.

Five weeks over Christmas they shut down. Just when the kids need it you know.

And this is Council isn't it?

Yeah. [manager] is the manager of it. And I think she's on wages with Council and I think that' s just – we'd like to see it set up so it's open every day. I mean I'm chasing a youth drop in centre right now. If we could use that, that would be fantastic. Because it's so important. But again because it's a Council place, it's all under so many restrictions. Just recently we had to do a funding issue to get money to help them out, because their Council bloke is not allowed to get funding, accept their own funding. I don't know.

Now destinations. If I wanted to ask you about places to walk from your home, what sorts of things would you tell me about? Now, obviously to get into [town] you go by car. And you actually say, you wouldn't walk on the dirt road because of the [35:46] of it, and you wouldn't walk on the main road. So um, would you actually –

You've got to park in the town to walk around. You've got to –

Yeah. But there is nothing you can actually walk to from your home?

No. Not safely. I mean I'll walk up the dirt road obviously. But if you've got um, a lady I care for, she's got a bad knee. And she's had an Achilles tendon before. And she can only walk a short distance. But quite often she'll go for a walk because she gets frustrated. And I've got to go and pick her up in the vehicle because she just can't get back again.

So does not having destinations influence whether you are active or not? If there's nothing you can actually walk to?

Probably not so much. Because –

You get in your car.

You get in your car and go to a spot and go for a walk. And it doesn't – I mean there is no way known I could walk to [town] and back. Although some people do. But just not having a footpath to walk on I guess is you know, that's probably the biggest obstacle. And there's a lot of people wont walk because there isn't – I mean even if they go and put a footpath down the side – so there is a gap between it and the road. You know, something like that.

If you were wanting to be more active would it help if there were more destinations within easy walking distance?

Well not from where we are, no. We're in the bush. I mean to build, to do anything active you've got to go into an area that is designed to do it. And there's no way known, I mean

we've got 11 houses in our street of 2km. I mean you know, what you can do? The funding's just not there to do that sort of stuff.

Yeah.

But footpaths would be nice.

Yeah, with obviously with the main roads being so narrow.

And with the kids riding pushbikes on the road – you know if you had a combination walk and cycle path they could ride their bushbikes on it without getting run over.

OK. How about neighbourhood design? If I were to ask you about the built or the man-made features of your area, what sorts of things would you tell me about? We've already talked about footpaths. Um, lighting? Whether the streets connect, whether you can actually walk them.

In the town you can. Not out our way.

And there wouldn't be any lighting out your way either?

Oh no.

How about in the town?

Oh there's lighting in the town and it's not bad lighting in the town, in some areas.

In some areas. Yeah, I've heard that too. Yep.

But there's other areas that aren't – basically well enough or not at all. And there's a lot of streets in [town], and I'm talking in the town, they haven't got footpaths. Again, people are walking on the roads. And that's slowly going, but not quick enough.

Not quick enough, no. And the other side of it is the aesthetics, the natural environment. Um, if I was to ask you about the attractiveness of your area?

Oh, magic. Magic.

It's beautiful.

Oh, I get to leave [suburb], as I do a fair bit. And it's green at [suburb]. You come over there into [village] and it's brown most of the year. You come into [suburb], it's still brown. You drive through [city], you get to [town], as soon as you get to [town] it turns green again. As you hit [town], it becomes lush. Green, trees, you know. And it's magical. It's just phenomenal. And the changing of the seasons, all the flowering trees, the flowering plums and the plum trees, the cherry trees. And the hawks and the crows. I mean everything in nature. Coming up this morning, coming up near [community centre] and there's a pair of swans with five babies walking across the lawns. And I'm a sucker for that sort of thing. So

that's the side of it that I like. You know like we've got on our little block, we have native hens coming to nest every year. We've had the same set of plovers there for seven years that don't let anyone else on the site. They're used to us, they don't swoop at us. Um, we've got ducks coming in every day. And they're the same ducks. We get to, we've got to know them. And that's what we like about the area. It's the magic of having that around you. But at five o'clock in the morning when they cows start and the sheep start…

Yeah. So do the aesthetics of your area influence whether you are active, physically active or not?

No, not greatly.

It's a hard question because in rural areas you actually pick to live rurally because of the environment.

I guess if I was younger and more fit and I could walk up the mountain, or I could, you know then it would be great. But because I'm older, I'm restricted as to where I can go and what I can do. Um, we've got a road that goes on forever that has been gated off to stop cars going through. And it's a dirt road. And you can walk for miles in the bush. But it's the physical ability to do it.

That's the dirt road that you have – you can actually..

Yeah, you can go right through. It's a gated road. And it becomes a private, or a forestry road past a certain point. But you can actually walk right through the back of it. And there is a lot of walking that you can do. But mine is the ability to do it. And as you get older, being able to do it gets harder and harder. We've got an old fella in our street who is 66 now, and [name] has lived in that street all his life. And he can tell about the tree that used to be in the corner of such and such and the house that was underneath it when it fell over. And he's in the archives down there. But even [name] now at 66 he is not as active. He can't walk where he used to. He's still pretty – touch wood, but he can't walk to the peak and he can't, you know. So the situation is there but as you get older you know, you can't. And there is not the infrastructure to jump on a bus and go somewhere and then – like that's probably the biggest killer is there's no bus. So to get from there to [town] you've got to have transport, your own transport. Um, and you can jump on a bus to go to [city] and come back. One of my friends is involved with [organisation] and they've just done a big thing on [state] Link buses. There was a lot of things came out that was very very interesting – but there is still shortfalls. There was a lot of positives, like, now we've got wheel chair

access buses. But there is still so many shortfalls in the system. And there's no way to beat it because of being in that rural area. And they've got to run a business.

So if you wanted to be more active are there features in your area that might help if they were more aesthetically pleasing? Or no, because they are beautiful as it is.

No, we've got – we're not at [suburb] I guess. We're not in a situation where the vandal – we get some vandalism, but since the youth have been involved we've lost most of our vandalism. So we've turned that around. Um, we've still got a few problems in the area because we've got a low income housing area in the middle of the town and that does create some problems. But, it's not major any more.

OK. One last area is safety. If I was to ask you about safety in the area what sorts of things would you tell me about? And you've just talked to me about um, the traffic, um in that you wouldn't go walking on the main road. So does um, does road safety influence whether you are physically active or not? It only does from the point of view that you can't walk …

Well you get run over if you are in the wrong spot.

Yes, exactly.

We've got school zones for the kids, we've got footpaths in the town in most places. Um, it's as probably well set up as any country town. But the issue is, as with most rural areas, is access – it's not like the city where it's compact and concrete if you like. We choose to live in spread out area. So I guess the bottom line is to get the quality of life that we want, we've got to give up certain things. I mean I guess that is the bottom line to the whole deal. But in return for what we give up we feel we get more than what ..you know, it's a balancing act.

Yeah. OK. So does personal safety influence whether you are physically active or not?

No. Not for me.

It's a safe community in that way?

Yes, if you work around things.

I guess it's more about um,

Planning your activity.

Planning your activity and you manage your arthritis in that way.

You've got to plan your activities and you can't be just you know, like if you want to be in [town] we've got the Walking Group as I've already told you. You know, we've got martial arts, we've got a swimming pool. It's there, but it's just not there to – ah, it's not there for the individual. It's there for the organised groups, which seems to be a big thing nowadays.

Yeah. So if you want to be part of an organised group.

I know my partner and she doesn't-

She doesn't always want to be.

She doesn't want to be a part of a group. And she wants to do it her pace and her speed. And that's the sort of problem that you have. Whereas if she was living in [city], you've got the acquatic centre, and do it basically any day she wanted she can slip into her bathers, go and sit in the pool. Or she could go down to [suburb] to the hydrotherapy pool or you know. But that's the sort of stuff that we haven't got. Yeah, and the other thing that worries me about the town is, you were talking about safety before, is we are [46:26] down here. And we've got a really good place in the town, but unfortunately the area is so big, the kids all know when we leave town and then they can do what they want.

It is a hard one isn't it? OK. Well that's it for today but if there's um anything else that, um about where you live or physical activity or physical environment that we haven't covered today, is there anything?

Probably one of the biggest things that we've had in [town] is that we've now got our Men's Shed operating. And they are hoping to get more of the old guys in town involved, only if they can get a coffee. In fact, the guys in [suburb] reckon that's their biggest bill. But we've just got to convince the people that haven't had it for 50-60 years that it is there for their use. And that's been a little bit hard. But the same with the Community Centre. Twelve months ago we had no one. Right now it's full, it's working, it's flat out all the time. So that is just a time thing. And you can set all this stuff up, you can put it all together. But it is then changing the thought cycle of the locals, to actually put it into the place. And that's probably the hardest thing. There's a lot of old families down there that have had nothing for years and they don't' expect it anymore. And it's the people that are moving in from the mainland that have had everything, that want more and I guess that's the bottom line. I think that's probably the difference.

So it's an interesting town in that way.

Oh very interesting. It's probably half and half now. It's got to the stage where there's old people dying and there's a lot people moving out, there's a lot of new people moving in. The new people moving in stay a couple of years and then they get out again. We've got problems with schools down there, and education. Past Year 10, they've got to come up to [x] College. We've been trying to work out how we can get some education for Year 11 and

12. And the kids are out of home at 6 or 7 o'clock in the morning and not getting home until 6 or 7 at night. And this might be a day where they have only got 2 classes, but they've got to be there all day. So we are trying to get round that at the moment, that's another one of our challenges.

That's important.

Yeah. But lots of stuff, but we live in a rural area because we want to.

Exactly. So thank you very very much for that.

AUDIO RECORDING ENDS

Date:	11 October 2011
Duration:	49 minutes, 32 seconds
Interviewer:	[interviewer]
Transcriber:	[transcriber]

OK. I thought we'd start with just talking a bit about the place in which you live. Yeah? So if you'd like to tell me about what it's like to live where you do? Your likes and your dislikes?

Yep. Yeah well I live in [town]. I feel very privileged to live here, because being close to both sea and to the bush. But I also like to have people around me. So having an urban environment around me, I still enjoy that. But being able to just walk down the street along the beach front and walk to church and walk to school and all those sorts of things, I think are a real privilege. So yeah, I find it very enjoyable.

It's a very beautiful spot where you are living. I was up there a couple of weeks ago and through the course of the study, have been up a couple of times. And it's really quite unique in that way isn't it?

Yes. Sometimes we are a little bit limited to some of our services perhaps. But I don't find what, my father I suppose drummed into me a bit that you can't always – like he lived on top of a hill and he'd say "you can't have a view without some wind". And the same is, you know it's lovely to have our quieter lifestyle, but you can't have that whilst being surrounded by services and activities. So there's that trade off. But I think it's a pretty good balance where we are.

That's good to hear. Now, do you actually live in the actual sort of centre of [town] itself?

Yep. Yep. Pretty well.

OK. And what's the property type that you live on? Is it a standard house and block?

Yes. I've got, we are buying our house which I'm renovating at the moment. And it's on a good size block. I've probably only got one direct boundary sharing neighbour, because I'm next to a laneway. And next to a church yard on one side of me. So I don't feel like I'm surrounded by neighbours. As I said, because I'm at the end of the street, I've still got people around me which I enjoy.

Great. And what's the length of time that you have been living there?

Ah, in that address, um 13 years?

And overall in the area?

In [town], I've been here 17 years.

Yeah, so you know it well. And your reasons for living there?

Um, yeah well I ended up marrying a [town] girl. And I had come back to [state] and found a new group of people that I was friendly with around [town] and yeah eventually met a girl and decided to get married and stay in the area. Bit of an old style way of doing things I suppose. It's worked well for us.

Yeah. Absolutely. And it's interesting, we've been talking obviously to quite a few people from [town] and [town], and not only in this study which is talking to men, but earlier in the year we were talking with women. And a lot of people have chosen to either come back and actually choose to live in the [town] area, or have come from somewhere else. And have chosen it. So it's a bit of a uniqueness of that part of the North West in that way.

Well I grew up in [village].

Oh well not far.

Yeah. And I'd been away and working in different areas including [city] [state] not long before I came back here. And I just found that I really did enjoy the smaller town, the community around that.

Mm. And we'll go on to talk a bit about that. But just to finish this bit off, um, what's the distance that you live from the sort of centre of [town]?

From the centre of [town] it would be about 800 metres. Under a kilometre, that's for sure.

So quite close. Yeah. So in research with men living in urban areas, which is what most of the research has been about, people often refer to their local neighbourhood. And I was interested in understanding what the term neighbourhood means to men who live in rural areas. And indeed if it is a relevant term. So what does the word neighbourhood mean to you?

Ah, yeah it probably would encompass in my mind [town], the township. Probably much the same kind of notion as community.

Yes, that's what I was actually going to ask you also, about the word community and what it means to you.

Actually, yes, but community does mean more to me. Because I work in the school environment, community – and I'm studying to be a youth worker and I've been working as a chaplain – community does involve for me like the school, the people around me and also some of the people supplying services into those areas. So it does have a – you know your service groups, you think of the Lions Clubs and the churches. And it has a wider, or a wider scope. It's still basically the area, but it has a wider, people who actually serve into the community, not just being in it.

So the difference would be –they are both relevant – but the difference would be neighbourhood would be more the geographic area.

Yeah, the physical.

Yeah, the physical geographic area. So if you needed to put a distance on that, in terms of kilometres, how big would that area be? [town] itself.

I hadn't actually thought of that before. It's probably about a 5 kilometre radius. There is parts, some older parts of town that used to actually go by the same name. There's one area on maps known as [town] town, which is the top of the hill. And that certainly is generally referred to as [town], but that's a bit further away. But certainly you feel as much as if you are in [town] as anywhere else. But yeah, probably about 5 kilometres I'd say. I might push that at times, because we've got bits of [town] that head off into the bush a bit. So there's probably some fingers that go out further than that. But the main, the main part of it would probably be within about a 5 kilometre spread.

OK. Now I want to talk a bit about physical activity, and as I said it's a very broad term, and it's different for each individual. So what does the term physical activity mean for you?

Um, not being on my couch. Physical activity to me is quite often just, yeah, being outside, even being outside of the office here where I try not to spend too much time. Ah, yeah just being involved.

Yep. And do you consider yourself to be physically active?

Ah, I do. Yes, I'm quite busy and I do spend a lot of time out of the house, out of the office. I don't do a lot of planned exercise as such. But I find myself quite busy. And you know, I

renovate and I walk and do lots of activity as such, but not a lot of planned activity I suppose. Or planned exercise.

Well that's a really important, and again sort of starts to talk about that broader notion in your life of physical activity, rather than you know planned activity. OK. And where does physical activity fit into your life? Is it of no interest? A priority? Or a high priority?

I think starting to mature a bit in one's years, I have a bit more of an interest in looking after my health and well being. Ah, and so it does have a priority. I am not though, I wouldn't put physical activity like in front of relationships. Like to me that probably would have a higher – so by meaning that, there are times where I might not do something physical because I wanted to spend time with someone; someone in my family, someone that I work with or whatever. Um, but it certainly has a high priority and I'm constantly aware of the need for it and to both fulfil my duties in life but also to keep well.

Yeah. Thank you. So in our research when we think about physical activity, we think about any activity that lasts for at least ten minutes – that's the evidence of health benefit effect – and causes your body to work harder than normal. So your heart rate might go up a bit, you might breathe a little heavier you know. And you might feel like you're warming up a bit too. So thinking about this type of physical activity during the last two weeks, I'll just go through different parts of your life, not to segment your life, but just to make sure that we cover things. So in the past two weeks have been active at all at work for at least ten minutes at a time?

Absolutely.

Yeah. So thinking about those activities, which obviously your work is based on because it's about that contact with the youth – actually you work both in the [x] and the [x] don't you?

I do. Yes.

But thinking about those activities, maybe it's the sort of walking around ones as well as being part of physical activity with sport, thinking about those maybe as two activities, what would be the intensity of those activities? Would it be steady, moderate or vigorous?

Oh it um, yeah probably not much more than a um, yeah the intensity is probably reasonably low.

So it would be pretty steady.

Yes.

But some of the sport playing I presume would be a bit more moderate from time to time?

Yes, I try to. But having said that, I still don't mind a bit of a run around with a few lads. Sometimes I'll do, like in the last um week I would have taken a group down into the bush, some young fellas. We were clearing a bit of a trail to um, yeah just to make an area that's a bit nice for people to walk through. And um, so it's not that – if I was working myself I would probably be working a bit harder. But you are working continuously, but like I say, it's probably at a lower sort of rate.

So just thinking about those activities, how long would you have done them at any one time in the last couple of weeks?

Oh at any one time, probably twenty minutes?

OK. And how often might you have done them?

Most days I would be having a couple of sessions like that I suppose. A couple of times where I'm working pretty solidly. I know one time last week – you know these vary too, but I run a couple of breakfast clubs, and on one of those, like just not having any volunteers bar one on one day, so you spend about three quarters of an hour running, or longer by the time I clean up, probably well over an hour at a pretty steady pace. So yeah, that can vary.

So in that sense your work – I mean a lot of people's work is more sitting around, but your work actually does incorporate physical activity with the job.

Yep.

And when would you normally do these activities? During the day?

Yep.

During the week?

Yes.

And is this what you usually do or is it one off?

No, no. I'm doing various types of things constantly. Like next week, this is a bit unusual, I'm going off to [state] with a school group. Um, and so we'll be on the go from early in the morning till you know a reasonable time at night time. And sometimes it sounds a bit glamorous, but I can tell you it will certainly be –

It's hard work.

Well it will be a good work out as well, the time spent with some young people.

Yeah, yeah. And does this change depending on the season or time of the year?

Ah yes. Certainly. Certainly does. It changes all the time anyway.

Yes, because of the activities are different, yep.

There are different activities, yes. Like sometimes I will take a group of young people down to do some bowling with residents of the old people's home, like that. So there's still a degree of activity over an extended period of time, but that's quite a lot lower.

So you are saying, does it change with the seasons?

Yeah, that as well. Sometimes I'll you know, like in summer I might walk a …. and we'll do some gardening somewhere. So but, generally speaking it's not that much more different. It seems that the [workplace] in winter doesn't seem to get out as much. Whereas in um I think the [workplace] is probably just as physical through the winter as it is. Ah, we find more things to do inside if it's um wet. Like we probably do more running around in halls and buildings than we would in the [workplace].

That's an interesting comment. In terms of you know that whole comment about you know that as adults our notion about physical activity starts really young. And you know, how that changes through our life.

Yeah, I certainly do notice that here, that if I don't come inside and look for them, I struggle to find them. I've got to remember to come inside and look for them.

Mm. Now in the last two weeks have you walked or cycled for at least ten minutes at a time to get to or from places?

Ah, walking and cycling. I haven't done much cycling, I must admit. I'm missing that. I do like to do a cycling. My walking to get somewhere. I do a lot of walking that would be under 10 minutes. Ah, like I walk to and from the church on a Sunday, which we would do quite frequently. Um, and to and from the shop. But none of these would be over 10 minutes, no. I do quite a few of them I suppose.

OK. Yeah, no well that's the thing that's important to balance out is that you do them quite frequently. But what would be the intensity of that exercise? Would it be steady or moderate or vigorous?

Oh probably steady again.

Mm. And how often would you do that?

Um, three or four times a week, every other day. But like I say, during the day like today, I would have you know, my recesses go for at least half an hour – I'll spend probably 50% of that walking around the [workplace]. I would do that every [break]… And my lunch hour I would spend 20 minutes or more walking around. Like I said, none of these, you are not going belting fast because you are talking to people or moving through groups and things.

It’s something that I do every day in my working life, just in those breaks, let alone any other activities. I’m not saying it’s some great regimen but yeah it certainly tires one by the end of the day.

Yes. Now those activities, would you be doing that by yourself or with others? The ones you’ve just described.

Ah, most of it, most of it by myself. Moving between groups, um but yes sometimes I, quite often, have a young friend with me, walking with me.

And is this what you usually do or is it one off?

Oh absolutely what I normally do. Yep.

And does it change depending on the season or the time of the year?

Not really. Not those activities, no.

And if you’d wanted to is there anything that would have helped you be, walk more between places, to and from places?

Yeah, I’d like to be doing more walking or bike riding to [workplace]. But I find myself continually having to take lap tops home, have food to bring into [workplace], have activities that we run on different days that we’ve got to – I would like to be walking or riding more to and from school, but some of those other necessities make it difficult.

Yeah. No, that’s a really good point.

I’d like to – because I have Monday, Tuesday that’s [workplace] and then I go to the [workplace] Wednesday, Thursday, and then I’m back to the [workplace] Friday – but the days that I’m there for at least two days in a row, I’d like to try to make those perhaps that I can, I’m not bringing too much in and out. Because there are some activities like the breakfast club and things, it’s hard to not have a vehicle. And also sometimes I, through the course of the day, sometimes I’m doing activities outside of school or I’m wanting to meet up with someone. Or I might be taking a group somewhere and I might use my own vehicle.

Yeah. So you need that flexibility.

Yeah.

Now in the past two weeks have you been active around the house or yard? So at home, inside or outside for at least ten minutes at a time?

Ah, yeah. Absolutely.

Can you tell me about what you did?

Mowed the lawns in the last two weeks, which is a record. Been avoiding them a bit, but um did some lawn mowing. I'm also renovating my house, which I've been doing –

Yes, well that's an activity in itself isn't it?

Yes. Though I've been probably a little bit quieter this last couple of weeks, because it's difficult coming back into the school term to find the time. But I still would have, oh the last couple of weeks, I would still have I don't know, oh several hours of work that I would have done outside. And like I said, the last, oh I suppose most of the time I've been here we've been doing some form of renovation. It's just a bit more official at the moment. I've pulled all the weatherboards off, replaced all the windows and I've done up all the kitchen. I'm making a room up in my attic and putting stairs in. So lots of things.

Yeah. So thinking about those activites, the outside and the inside and the renovation, um what would be the intensity of those activities? Would they be steady, moderate or vigorous?

I would probably put those moderate. Because being a – I've worked as a tradesperson, as a fitter and machinist – and um, I suppose I'm more used to working at a bit steadier pace than what my other activities around school would be. So when I do work I like to keep going at a bit stronger pace. So probably class that as moderate.

And how long would you have done those activities at a time?

Um, oh two to three hour bursts I suppose. Probably three hours.

And in the last two weeks how often would you have done them? You've got the lawns, so that was twice wasn't it?

Ah yep.

And the renovation?

Yeah, probably about three efforts there.

Yep. Yep. And when did you do these activities?

Ah, mainly around weekends. So Friday nights, Saturday, Sunday. Yeah, probably mainly around those times. Um, that will change now with the daylight savings and weather. I will start doing more of that through the week nights as well. When I have the opportunity to.

And would you do those activities by yourself or with others?

Ah, a bit of both. I'm quite fortunate. I have some good helpers around town who quite often volunteer themselves. One chap in particular who will come over especially to help

me do some of these tasks. So pretty fortunate. And also my wife will be helping us out as well.

Yep, yep. And is this what you usually do or is it one off?

Ah, I suppose it's pretty usual. When I'm not doing that, I've got a brother in law who has just bought a house. I'm sure we'll be doing more of that there. And um, we've done a lot of work for our church. And we've just bought a nice new big old warehousey type building in the middle of [town] that we'll be doing a lot of building work to. I know like this weekend we've got, oh not this weekend – yeah, no Friday week we've got um a night time working bee and then a Saturday one. We've got a couple of big pushes there to get a lot of work done. So yes it is something that I would probably do.

And does it change depending on the season or the time of year?

Ah yes. I would do less of it through the middle of winter. But probably a little bit less through the winter.

Yes. And as you said um, you'd start to do more now that you've got a bit more light during the week too.

Yes. It does make you feel like wanting to be outside doing some of those things a bit more.

Yeah. And if you'd wanted to, is there anything that would have helped you be more active around the home, either inside or outside? Sounds like you are pretty busy anyway, but if there is anything…

Yeah, I would probably um, I try to change my lifestyle a bit to be a little bit quieter, and I've ended up probably just as, probably as busy as I ever was. So I need to keep watching that I don't have too many things in my life. So that's something that I'm continually looking at so that I'm a little bit freer to do some of these things.

Yep. And have you been physically active for at least ten minutes during your leisure or spare time? And that's really picking up anything that we've missed out so far.

Well I like the concept of leisure and spare time, apart from what we've talked about on a regular basis – probably not.

You mentioned cycling though.

I yes, I'd like to be – but the way I would ride my push bike more would be going to places, a bit like school or to my church or to a meeting even, or something like that around. They're the sorts of …

So it would be with a purpose, which is –

And I think that's a bit more of a rural sort of, people are more inclined to be riding because they have got to go somewhere or walking because they've got to – you do see a lot of people walking around the town here, who just like walking. That has certainly become more apparent over the years. Ah, there's a lot of us that probably think that it needs to have a purpose behind it. So for me to go for a ride on your bike to get some milk rather than hop in the car or something like that, they're the sorts of things that I would like to be doing more of.

That's a really important point, how people view that. And quite a few people have said that to us, that yeah you often sort of think of a purpose for you know, doing this activity. So yeah, so it does make a lot of sense. OK. Um, now the last part of this is the physical environment. And so we've talked a bit about where you live and your activity. Now I want to talk about the physical environment where you live and work and play. And when I talk about physical environment I mean all the physical things that surround you. The roads, the trees, the houses, the shops, traffic lights, etc. Although you don't have any of those in [town] but probably a bit of traffic.

We do have a set of traffic lights. A set. And it's in a stone's throw of my house. Things still beep at us till 10 o'clock at night. If you are trying to have an early night or whatever. So yes, we do have a set.

Fair enough. And some of these things are things that people living in urban areas have said may be important for their physical activity. So I was wanting to ask if they're relevant to you. So first of all, can you tell me a bit about how easy or difficult it is to be physically active in your area? And why?

Um, not at all difficult. As I say, I live in a part of town that I find especially beautiful, because I'm looking straight at the water and it's flat. Which I, I like flat. And so I can step outside my door and within about five minutes be at my church or down at the supermarket or within two minutes I could be on a beach. So having those things around me I, and I also like – haven't done a lot in the last couple of years - but I do enjoy some bushwalking, and we have got the [x] Range five minutes in the car behind us. And I do a bit of walking around there with school groups and things. But I haven't done a lot of formal bushwalking in that area. So yeah, not at all difficult. Not at all difficult.

And so I'll just go through a couple of areas. Um, availability and accessibility y of places to be active. If I were to ask you about places to be active in your area what sorts of things

would you tell me about? It's more a general comment, it's may be things that you yourself do, but it may not be.

Yep. We've um, one thing that springs to mind; like in the [x] school where I work here now we've ah, part of our building the education revolution or whatever it was, the Rudd money, we've um put a nice new gymnasium at the school here. So we've got a lovely modern gymnasium facility which is available to staff and to the public to some extent. So that's something that's available. And I think having fairly quiet roads and decent parks. Certainly the parks and the beach, I know that it's good to take the kids down and muck around in those areas. So those, I suppose just having decent paths and roadways around you do make it.

And you've talked about the bush in the [x] Range close to you too. OK. So thinking about that, are these places convenient? Are they easy to acces? Like the cost or the operating hours?

Oh absolutely, that's a big factor for us. Single, probably lower income. And so for us, most of these things are no cost. So for me the idea of paying to exercise is a bit foreign. In as much as to be doing it without, so even the idea of the gym work is a little bit ah, I'm a little bit slow to grasp. Ah, because I tend to find myself things to do rather than spend that time in a gymnasium.

Mm. That's a very good point you've made actually, yeah. And yeah, in terms of people's access to physical activity, yes, that's important. So does having places to be active influence whether you are active or not?

I believe so. One thing, I thought you were going to ask if there were things that could make it easier or we'd like to see. I know we do miss – it's lovely going to [city] to perhaps, to visit the aquatic centre. A good aquatic centre would be magnificent. That's probably the main thing that we miss, and that I look forward to to having trips to [state], or to the cities or whatever to go and visit a nice pool is something that I do certainly look forward to.

Thank you for that. I was actually going to ask you about if you had some suggestions, so thank you.

That's probably the main thing. Well we've got a nice athletics track here in [town] too. And you know we have oh, forgotten what it was called now, but like the Cancer Council we have like 12 hours or so where they walk around a track and raise money and awareness around cancer and research and stuff, which I like to get involved with. So we've got

opportunities like that which is, you know you would be less likely to do some of those activities without having a nice athletics venue. That's a good facility in our area.

Yep. And certainly a few people have mentioned the aquatic centre idea for the area would be really good. So gives all round, full year round access.

Yep. We tend to visit the pools and things. I had a swim in the – I'm quite reticent to swim in the ocean even though I've got a diving, a scuba diver – I've done quite a bit of. But it's not quite as enticing, but I'd say yeah, a covered all year round pool would go extremely well here.

Another area is destinations, which you've actually brought up a bit already. If I were to ask you about places you could walk or cycle to from your home, what sorts of things would you tell me about? You've actually told me a lot about the schools, the services, the shops, the recreational facilities, friends, are all within walking distance.

Yeah that's right. Absolutely.

Anything that we've missed out?

Um, well I have friends that will cycle to other towns. Like they will go to [city] from [town]. One of them would regularly do that for his work. I just saw while we've been talking, there's a fellow at the school here who has brought his pushbike out of an office to ride back to Port [x]. So it's surprising the amount of people that do go quite a few places. But no, I suppose it's pretty general, most areas of our life around here could be serviced on a bike or by walking.

I guess the benefit that you've got too, which people have commented on is that because you're not on the main road, you actually can sort of cycle to various locations without having to deal with the main road. Except I think, um a little, there's a bit of main road people have commented on going the [city] way around [39:50?] Creek?

Yes, if you went to [city] you'd have to access the highway, but that's not too bad through that section.

Mm. And the other way you can get to [town], although the road is a little narrow on the coast I've heard. But also there's that um, people have been commenting on that cycle walk track that takes you to [town].

Yeah, which I've heard is magnificent. Yes. There is um, and I lived for some time in [city] and was a big fan, they had some beautiful cycleways there. Like from the pool right round to, or down town to the river. And you can go for miles on cycleways. And I think where

appropriate they certainly do encourage more people to get out where you can cycle. I do have friends who have spoken about that one at [town] and it sounds quite interesting.

And that's what I was going to ask you: does having destinations influence whether you are active or not?

Destinations?

Ah hm.

Yep.

Yep. And if you wanted to be more active, would it help if there were more destinations within easy walking or riding distance? And I think you've been actually saying that [town] is very well placed and that it is quite accessible.

Oh yeah, absolutely. Well another one, I mean I've, I'm in my 40s, but I have been known to go down the shop on a skateboard or something around the street too. Because it's nice and flat. My, ah I've got some primary aged boys who will be on scooters and it's not unknown for me to have a flashback to my youth and grab a skateboard or something.

Sounds great. Now we are actually going to take up, the next question is around walkability. It's about neighbourhood function and design. So if I were to ask you about the built or man-made features of your area, what sorts of things would you tell me about? One of the things you have actually said is that [town] is very walkable. It's flat. And it's obviously got walkways.

Yep. With the last couple of years they've built a lovely walk along the beach front. So very close to my house, I'd say within probably two minutes I can get down towards the beach and ah it's quite a lovely four or five hundred metre stretch along the coastline that is nicely removed from the edge of the road and wide enough that you know groups could pass each other. It probably goes longer than that, I don't know, but it'll join from one beach right along the main street. And those areas certainly do make a – and there's been, I think there's been a fair bit of footpath that has been repaired or dug up. And they've also been fixing up curbs so that you can get on and off them, like on a pushbike or a scooter or something. You know, they make them cuttings. Some of our older style footpaths are a bit sudden, they don't have that sort of easability to get anything wheeled on or off. You know, scooters, wheelchairs or whatever. So they've been doing a fair bit of that type of work, that we've been pleased to see. So those types of things are making it easier.

So do the built features of your area influence whether you are physically active or not?

Ah, certainly help. Yep.

And if you wanted to be more active are there any built features in your area that would help? Now we've obviously named the aquatic centre would be one.

Absolutely.

Is there any others?

Um, I think the idea of bikeways or thinking of areas that can be better connected with um, yeah with biking and walking tracks is good. We've got some, actually one area that I hadn't thought about until we were talking – behind the schools here there is a section of bush with a lot of tracks that we like to mountain bike and that in. And the kids will grab BMXs and I will be chasing them around on a mountain bike or whatever. So it's good having some areas like that as well where we can – that's virtually next door to the school.

So you've got a bit of a choice in terms of a range of activities for people.

Yes. And so um, some of those type of areas and it's been up in the [x] Range, some of those tracks and things could do with a bit of maintenance. A lot of them are, a lot of the walking tracks in our [range] area aren't as well maintained as they could be.

Yep. And the other side of it is the natural environment. If I was to ask you about the aesthetic qualities or the attractiveness of your area, what sorts of things would you tell me about? And you've actually told me about it's a pretty beautiful spot being close to the coast and also the ranges.

Yes. Lovely beaches, the [x] Ranges, and our bushland. Yep. Just the general coastline along here. Like you were talking about the narrow road. Like along our old coast road there. And ah we still like to scrabble round a few rocks and things like that on the coastline.

So do you think the aesthetics of your area influence whether you are physically active or not?

Oh absolutely. Yep.

Yes, if you've got beautiful environment then it really helps, wanting to be out in it and get out in it and do something, doesn't it?

Yep. I think I'd say sometimes we need a little bit more, like some better managed walking and mountain biking tracks in this beautiful [x] Range. It would encourage I think a lot more activity.

Yes. So for specific sort of targeting for different activities.

Because I've walked right through this area. Like there is a [town] to [x] Mountain track –

Yes and it not only encourages people, it looks after the bush itself too. OK.

The tracks that we do have can be quite hard going.

Yep. So those are some features that might help. The attractiveness, but also it sort of fits in with the built environment too. Now one last one is safety. So if I were to ask you about safety in your area, what sorts of things would you tell me about?

Um, I'd tell you I feel very safe. In both physical safety as in not too worried about being run over or knocked over um most of the time, to not really feeling a threat of violence I suppose. Um, so yep, yeah quite comfortable as far as safety goes.

Yep. So that with road safety, does it influence whether you are physically active or not? And it's a safe enough area.

Not a concern. Not an excuse I can use I'm afraid.

No. And the same with personal safety. So if you wanted to be more active – just asking the question – are there any personal or road safety issues that if addressed might help you be more active?

Ah around safety. Not in particular.

Yep, that's great. Well that's the end. Um, but before we finish is there anything else that you'd like to tell me about where you live or your physical environment and your activity that we haven't covered today?

Um, no, no I think that's it. You get probably a bit of a general idea that um, you know certainly for my life it's more about keeping busy rather than targeting particular pursuits. And ah, yeah but being aware, looking for opportunities where I can walk or be active rather than always using, using the car or whatever.

Thank you.

AUDIO RECORDING ENDS

Interviewer: So I thought we might start by talking a bit about the place where you live and what it's like. So can you tell me about what it's like to live around here, your likes your dislikes?

Respondent: OK. So we live in [town] which is nice, we live on a property. We're quite lucky in the fact that where our house is located we're not very far out of town so it's nice to have the bonus things like water and garbage and you can walk into town, it's a very short trip. It's cold, that's one of my dislikes, I don't particularly like winter. It's a nice community, it's a nice group of

people that we have support and friendship with which is nice. What else do I like? I like living in the country, I like being out of the city but I like the fact that we're only an hour away so it's accessible, you can go down for a day trip you don't have to plan to go for weeks or anything so that's a good thing. That's probably it? Is there anything else?

Interviewer: The property you're on... is that OK? Your partner is...?

Respondent: Oh yep.

Interviewer: I just thought I'd check, it's really hard when you hear a baby crying. The property you're on how big is that?

Respondent: So it's about 5000 acres here with some bush and other bits.

Interviewer: A big one. And the length of time you've been here?

Respondent: So I moved here five years ago. I moved here from [city] but I grew up in the country so it's like coming back.

Interviewer: And your reasons for living here?

Respondent: My husband because we met and he runs the farm here so yeah.

Interviewer: It's the family farm here.

Respondent: So it wasn't really an option for him to move anywhere else so I moved here.

Interviewer: Now you mentioned that you can just walk into [town]. How far would it be just in distance?

Respondent: It's probably 500 metres to the edge of town.

Interviewer: In research with women living in urban areas, people often talk about their local neighbourhood and I'm interested in understanding the term neighbourhood and its relevance in country areas. So if I was going to... I'll ask you what the word neighbourhood actually means to you and is it relevant?

Respondent: Probably the better definition that I have of neighbourhood at this point in time is very different to the neighbourhood I had when I was in the city. In terms of I know my neighbours, I will pop in and have a cup of tea, you know their children sometimes you know their parents and in terms of neighbourhood I guess [town] is a really funny community and you can be quite segregated and so I think there's a really good farming community in terms of so the people who I socialise and I guess would call friends and

could call on if I needed something would be those who are also farming people. And there just seems to be a bit of division between farming and townsfolk so to speak.

Interviewer: So the people who live in the town but don't necessarily do farming...?

Respondent: Yeah which is quite interesting and I really noticed it more so when [x] and I went into a thing at a local school and nobody that I really knew were there. There were a few people that I knew that worked at the shop and that sort of thing and I said 'Hello' but nothing more, they don't try and strike up a friendship or sort of, it's quite interesting the incident... you think that sometimes those things don't exist anymore but it really does.

Interviewer: So neighbourhood for you would be? What would it mean? Seeing as you've just said to me it's more of the farming area so?

Respondent: It's important to me. It's something that...

Interviewer: If you need to put a distance on it what would it be?

Respondent: Oh a distance, it's kilometres and kilometres.

Interviewer: So 50 kilometres?

Respondent: Sometimes some of our neighbourhood that I... it's more of a district I guess that I refer to rather than a neighbourhood.

Interviewer: And a farming district?

Respondent: Yeah. And so for us here probably our local [town] neighbourhood would probably be the furthest property away would be 25 kilometres so it's probably where we would sit.

Interviewer: And I want to ask you about the word community and what that might mean to you? Is that different?

Respondent: I guess they have similarities but they're also different. And I guess something that springs to mind when we talk about community is I think about the whole of [town] and I think about recently there was a tragedy in the town...

Interviewer: Yes I remember that.

Respondent: And everything was put down and everyone came together and it didn't matter where you lived, who you were everyone had their little bit to help

and so that sort of... I think community is probably broader than neighbourhood for me.

Interviewer: Yes and if you needed to put a distance around that? It's more about... so if you needed to put a distance what would community be?

Respondent: So I guess when I think about neighbourhood I think about lots of people around the actual township of [town].

Interviewer: The farming communities around.

Respondent: So then when I think about community I think about the people in the town and the people surrounding.

Interviewer: The drawing together is that people will come from the same area, where as neighbourhood for you is the area but it's also the interest in terms of farming?

Respondent: Yes.

Interviewer: That's fine. Now I want to talk a bit about physical activity and what does the word physical activity mean for you?

Respondent: So it means anything that gets your body moving so it can be simple things like I guess for me living on a farm it might be getting out and opening gates for my husband when we go and move sheep or it could be me actually going for a walk or doing something else that's actual physical activity so it's part of a broad term.

Interviewer: And it's something you do in your day to day life in that way?

Respondent: Yes.

Interviewer: And do you consider yourself physically active?

Respondent: I do. I am physically active but I'm certainly not a fitness freak. It's something that is in the forefront of my mind to be physically active.

Interviewer: Why?

Respondent: I think health is an important thing and you need to be proactive in your health and being a [occupation] I know about the things that can go wrong when you don't look after your body so making sure that you have a holistic approach, so physical activity is one component of trying to maintain your health...

Interviewer: Where does physical activity fit into your life? Is it an interest, a priority, a high priority?

Respondent: It's a priority but it's one of those priorities that tend to get put off if something else comes up.

Interviewer: I guess particularly now with family it's sort of...

Respondent: Definitely and also having family means that I'm not working at the moment so I'm here all the time whereas I used to be going to town for work...

Interviewer: Oh right so you were working in town?

Respondent: Yes which meant that I had access to go to a gym class after work or to go for a swim or things like that? It was just easier. I can still do those things but it takes a lot more planning.

Interviewer: OK. So in our research when we think about physical activity we think of any activity that lasts for at least ten minutes that is the evidence of the health benefit as you know. And it causes the body to work harder than normal, it might be the heart rate's a bit up or you might find you feel warmer, great for winter and there might be a bit of huffing and puffing so thinking about that type of physical activity during the last two weeks. I'll just go through parts of your life, not to segment them but to mainly make sure that we collect all the activity that you do.

Respondent: OK.

Interviewer: I'll just check with you, you were just saying that you weren't currently working and obviously haven't in the last couple of weeks so we'll leave that one.

Respondent: I am working in the office just sort of, not my usual profession.

Interviewer: OK well that's fine. Let's talk about that then.

Respondent: Sorry.

Interviewer: That's absolutely fine. That's why I'm checking with you. Do you know we... what do they call it rich lives; we're all full of different things that we do. So thinking about that, in the past two weeks have you been active at all at work for at least ten minutes at a time?

Respondent: No.

Interviewer: And can you tell me why not?

Respondent: Because the work that I'm currently doing involves me doing the office work for the farm which is a job where you sit at a chair at a desk working on the computer or getting up to do a bit of filing. It's not physical.

Interviewer: And if you wanted to is there anything that would have helped you be more active at your work?

Respondent: No.

Interviewer: In the past two weeks have you walked or cycled for at least ten minutes at a time to get to or from places?

Respondent: Yes.

Interviewer: Can you tell me about what you did?

Respondent: If I need something from the shop I will walk into town to get that rather than driving. And when I was in town last week I needed something from the shop and I also wanted to go for a walk.

Interviewer: Thinking about those activities could you tell me the intensity of them, whether they're steady, moderate or vigorous?

Respondent: Probably vary between steady and moderate.

Interviewer: The duration? How long might they have taken?

Respondent: Between 20 minutes and an hour.

Interviewer: The frequency? Did you say it was a couple of times?

Respondent: Yes.

Interviewer: OK. When did you do it? During the day?

Respondent: Yes, during the day usually the afternoon.

Interviewer: During the week or weekends?

Respondent: During the week.

Interviewer: And you've told me about where. And why did you do it there?

Respondent: So both times it was needing things, so needed something from the shop so may as well walk.

Interviewer: Where you alone or with others?

Respondent: With [x] my son, if he counts?

Interviewer: Absolutely. It's a great name. Is this what you usually do or is this one off?

Respondent: No it's what I usually try and do.

Interviewer: And does this change depending on the season or the time of the year?

Respondent: No but it does depend on the weather in terms of if it's raining I won't go.

Interviewer: Fair enough. If you wanted to, is there anything that would have helped you walk more often?

Respondent: So in terms of?

Interviewer: To and from places.

Respondent: To and from places? No not really.

Interviewer: So in the past two weeks have you been active around the house or yard, so at home inside or outside for at least ten minutes?

Respondent: Yes a bit of cleaning here and there. Some gardening but that's about all.

Interviewer: On the property? Gates and things like this?

Respondent: No not in the past two weeks.

Interviewer: And the intensity of those activities? Steady, moderate and vigorous.

Respondent: Steady.

Interviewer: OK, duration?

Respondent: Only quick things so probably ten, 15 minutes.

Interviewer: The frequency? How often would you have done that?

Respondent: I think I would do something every day. There would be ten minutes work.

Interviewer: So that would be inside and would that be outside? The outside activity as well?

Respondent: Yes so things like hanging out the washing. So between them both it would be probably more inside than outside.

Interviewer: At this time of the year. And so when did you do it?

Respondent: Usually in the morning or the afternoon.

Interviewer: And why?

Respondent: Pretty much to get the jobs done. To get chores ticked off the list.

Interviewer: Did you do it by yourself or with others?

Respondent: Usually by myself but sometimes a bit of gardening [son] will come out and sit in the pram.

Interviewer: I was going to say you probably wouldn't get some help there. Is this what you usually do or is it one off?

Respondent: No it's pretty standard how our weeks go.

Interviewer: And does it change depending on the seasonal time of year?

Respondent: I guess it does so in winter you are less inclined to go outside and do things.

Interviewer: Yes there's less to do in the garden as well.

Respondent: Exactly, we don't have much of a garden but a little bit to keep me busy.

Interviewer: So if you wanted to, is there anything that would have helped you be more active around the house or yard?

Respondent: Probably [son] sleeping more or being away with someone else at the time. It does impact on what you can do when.

Interviewer: So if you had more time or... at his age you have to arrange out past a point, arrange his care. Have you been physically active for at least 10 minutes during your leisure or spare time?

Respondent: Yes.

Interviewer: Can you tell me about what you did?

Respondent: I am to walk every day, doesn't always happen.

Interviewer: I remember when I rang, you were out walking.

Respondent: Yes, so probably in the last two weeks I've probably walked ten out of the 14 days. And I've been for a swim, two swims actually if we are talking about two weeks. So that's...

Interviewer: So where do you get to go for a swim?

Respondent: I go to [city]. So what we try and do is have one night a week in [city] so I have access to a pool or a gym to go and do something.

Interviewer: That's a really good idea. So thinking of those activities could you describe the intensity of them?

Respondent: Probably moderate for all of them.

Interviewer: For how long would you have done those activities?

Respondent: I usually walk between 30 to 40 minutes and my swims are about 30.

Interviewer: And you've told me its two swims and nine walks?

Respondent: Nine walks.

Interviewer: OK, so when? What time of the day would you have done this activity?

Respondent: So swimming I usually go seven o'clock in the morning and my husband has [son] and walking we usually go at around, depends when [son] wakes up but usually three or three thirty. It's earlier this week.

Interviewer: Yes because of the light. And why did you do that? Obviously you just said about [son]'s care?

Respondent: Sorry? Why did I walk or why did I...?

Interviewer: Yes, the two activities why you picked to do them at that time?

Respondent: OK. Walking is something that [son] likes playing in the pram and I like getting out and getting fresh air. Yes, swimming is... I had some care so [x] was there to get to watch him and...

Interviewer: Where did you do those activities? You've told me about the swimming? Now walking, you usually walk from home?

Respondent: Yes.

Interviewer: And it's around your local area? Your favourite little walking tracks?

Respondent: Yes. We have a few walking tracks, depending on the time frame and so I'm not a big walker on the farm. I usually head into town and walk around town.

Interviewer: And I just asked how you, what method of transport you've got to do these activities? Obviously you're walking from here and going to [city] would be by public...?

Respondent: Car.

Interviewer: Yeah car. And who with?

Respondent: So swimming by myself and walking with [son] usually.

Interviewer: And this is what you usually do? Or is it one off?

Respondent: No it's what I usually do.

Interviewer: Yes. And does it change during the season? Depending on the season or the time of the year?

Respondent: Yes I guess timing is the big thing so when there was daylight in the morning I would go early before [husband] went to work so I could go for a walk by myself. And then during summer when the days are longer we go a bit later in the afternoon. And we don't go when it's raining and cold and windy and horrible. Any excuse to stay indoors.

Interviewer: Fair enough. If you wanted to, is there anything that would have helped you be more active in your leisure or spare time?

Respondent: I guess for me living here, there isn't anything to do other than what you decide to do for yourself. So there isn't a pool that I can go swimming, there

isn't a gym class I can go to. Because my times, [husband] finishes work at five thirty so I could go to something at six or after. Whereas it's just not on offer so that impacts me because I would do something like that. Other things that... I guess the weather, lately in the last couple of weeks haven't been particularly nice in terms of rain, wind and that sort of thing so that definitely impacts. Yeah they're probably the two main things.

Interviewer: Now going onto the last part of it which is physical environments and actually picking up on some of the points you were starting to talk about availabilities and things. So we've talked about where you live and your physical activity, now I want to talk about physical environment where you work, live and play and when we talk about physical environments we're actually meaning all the things that surround you. So the trees, the hills, the roads, lighting, traffic, parks all of those sorts of things. I want to just go and talk about several aspects of it but the reason why we're asking is that people in urban areas have often said that these sorts of things are relevant to their physical activity. So first of all can you tell me a bit about how easy or difficult it is to be physically active in your area?

Respondent: It's easy if you're willing to be self motivated and self directed and do it. I guess it's like anything, if you want to do something then you can do it.

Interviewer: And what makes it easy?

Respondent: I guess it's about being a priority and it's something to me, I want to lose weight and I want to be healthier so it's an important thing to do for that aspect so that makes it easy or easier.

Interviewer: And as you were saying with your walking, you're able to just literally leave the front door and walk. Now the first area I'll ask you about is availability and accessibility which we've started touching on, of places to be active. So if I were to ask you about places to be active in your area, what sorts of things would you tell me about?

Respondent: The obvious one that we have is wide open spaces and have plenty of places that I could walk so I can walk into town or I could walk on the farm; I could go up the hills or anywhere so we have that available. There's a local pool that it's...

Interviewer: Yes I saw that one as I came in.

Respondent: It's only open during summer understandably because it's outdoors and not heated particularly well and to be honest I've never been there to actually swim but it's a small pool and it's more for little kids to go and have a play as opposed to an adult trying to do laps. So in terms of availability that's all that I know that is available.

Interviewer: In your community? And other than that it's literally going down to [city] or is there anything else around you?

Respondent: Well not really, I think there are some things at [town] and [town] and that but I'm not involved in them.

Interviewer: At that stage you might as well be in [city].

Respondent: Exactly, go to [city].

Interviewer: So really there isn't much availability in places to be active and likewise the access, there not easy to access if they're not there.

Respondent: Exactly.

Interviewer: So does not having places to be active influence whether you're active or not?

Respondent: It does to a degree because I get bored walking. I like it, it's good to get outside and to get fresh air but day after day it does get a bit... and I'm like 'Oh where will we go today?', yes I'd like to do something else and that's why I've started swimming which has been in the last month just to mix it up a bit because I just get bored and as much as I like it and for me [son] is wonderful to walk with but you know there's no conversation or anything so you're there by yourself. Sometimes I take my iPod but you're there by yourself.

Interviewer: And if you wanted to be more active are there things or places that would help if they were available or more accessible?

Respondent: Definitely. I think that and it's something who, I have a friend who has recently moved to the country and she is a personal trainer and I've been trying to talk to her to get her to come in and do some classes in [town] because there are lots of people who I know would like to do it but just are like me, can't access it and don't necessarily have somewhere in town where

they can go and stay, so that helps me so I can go down and stay the night and [husband] can come in.

Interviewer: Is there that facility in [town], a community hall or something that...?

Respondent: Yeah. Definitely there's community hall, the school gym I'm sure would be available.

Interviewer: Yes because my memory was that the school is actually quite an active part of the community or interested in being.

Respondent: Yes and I think it depends on what stage of your life that you are in, whether you are in that school community or not and I guess I'm probably on the fringe of it because I do go to leap frogs which is for little babies. But yeah, not really into the school community.

Interviewer: But those are the two facilities, I think I was being told about the health services over in other places of the [region] like [town] but here in [town] the sort of community facilities are the hall and the school basically.

Respondent: Yeah so basically and the pool which is cold and there is a recreational ground but that's for the guys to play football and I don't think it really gets used for anything else.

Interviewer: Fair enough. So it would certainly help if there were some things that were available.

Respondent: Yes and I guess I think that there's so much evidence about wellness programs and things like that really benefitting communities and people personally and I think it would be great if the council was proactive in terms of that and could organise something.

Interviewer: It's been one of the things that women have actually said all over, is it would be something that they could go to in their local hall and half of it would be they would support it because it's part of being part of the community and things and being social.

Respondent: Exactly. Yeah that's exactly right.

Interviewer: Yes so it is important. And it's interesting how often at the present moment it isn't available.

Respondent: Yeah definitely.

Interviewer: Particularly in the smaller communities.

Respondent: Where I could think of ten people off the top of my head who I know would go and support those classes which I think is enough to do something, it's not like you've got two people who are going to... and I know there used to be tai-chi classes that were held at the hall but they don't happen anymore but I know a lot of people went to those just for that exact thing. It's not only that you're getting some physical activity you're getting that social interaction.

Interviewer: I want to ask you about destinations. If I were to ask you about places you could walk from your home what sorts of things could you tell me about?

Respondent: To a destination?

Interviewer: To a destination.

Respondent: OK so I could walk and see [husband]'s parents and take [son] to see his grandparents. He is starting to crawl so sorry he's having a few bangs. There is a cafe so we would walk into the cafe and have a coffee, I'll walk to the shop and get the newspaper or get some milk or things like that. I don't really walk and see people because they're a bit too far...

Interviewer: You're a bit more get in the car and drive to see friends?

Respondent: Yes. We might walk to the park if we have older children here that want to do something but they're probably the main things.

Interviewer: Does having destinations influence whether you're active or not?

Respondent: Yes I like destinations. I like to have a reason that I'm going to do what I'm doing.

Interviewer: If you wanted to be more active would it help if there were more destinations within an easy walking distance?

Respondent: It would but I don't really know what destinations I'm looking for.

Interviewer: Yes I understand what you mean.

Respondent: I don't know what else the community could offer to give...

Interviewer: I think one thing that came up in [town]; someone was saying that there is public access to the river? But they were saying that if that was slightly better that that would offer some options?

Respondent: OK I guess and I do walk down there but haven't lately because it's been flooded and that's one of my things with it but it's a beautiful spot to walk even though the bridge is a bit rickety when you have to cross over the river

but it's been flooded and as soon as we have any rain it sort of floods and you can't go there. There's Mt [x] I guess, that's a destination which is a hill but I don't tend to go there. Not that big a fan of hills.

Interviewer: Now just wanted to ask you about neighbourhood design. The man made features of built features of your environment. And if I was going to ask you about those what sorts of things would you tell me about?

Respondent: So are you talking about footpaths and that sort of things?

Interviewer: Yes. Footpaths, lighting, roads...

Respondent: So I guess in terms if I start out of our front door we walk onto a road that I thought that when we first moved here was not busy but it's actually quite a busy road and the point where we go from the speed limit change so people who are coming out of town, you have your end 60 sign and then it goes quite fast and we're also at the point where you hit the 80 sign so one thing that impacts on me is that I don't like walking there that much, and I have to but because cars are going so fast and I worry that the pram and a lot of people don't slow down and a lot of people don't move over to the side or...

Interviewer: So are you able to walk on the road or is it on the side of the road that you walk?

Respondent: I usually walk on the road because there isn't a footpath, there isn't... there's a little bit of gravel and then there's grass but the grass is not cut, it's long and it's not a place where you can walk. So I do it but I don't like it, it's my one bit of walk that's... and then when I get into [town] I usually walk on the road or the street because the footpaths are so hit and miss so you have them...

Interruption

So we tend to, because I have the pram a lot of the footpaths have got boy metal so they're not, or they're grass and so they're... and you're walking on the footpath and it will stop so there isn't a footpath and you think well I won't start..

Interviewer: It's very difficult with a pram.

Respondent: It is so it's much easier just to walk on the road, so we tend to do that. In terms of lighting, I guess when we are coming into winter I was walking at

seven-ish and there's very minimal street lighting and I stopped going and especially because of this bit of road there's nothing which is understandable...

Interviewer: So as soon as you leave [town] it's...

Respondent: There's nothing.

Interviewer: Town limits yes.

Respondent: What else is there?

Interviewer: I guess the other ones to is street, what they call... do streets connect together because often...

Respondent: Yes.

Interviewer: They do in [town] because in other places they don't which makes it really, you've got one major highway and that's...

Respondent: No they do connect together and there are a good few routes that we can take which is good.

Interviewer: And generally footpaths once you get in to [town]?

Respondent: No.

Interviewer: No? You've got the same sort of problem as of pushing pram...

Respondent: Yes so usually we're on the road or the street because you get on a footpath and then they stop and they don't start again or they're only on one side of the road and you're going the other way and it's usually not overly busy so it doesn't matter too much.

Interviewer: So do the man made features or built features in your area influence whether you are physically active or not?

Respondent: They don't but they...

Interviewer: They don't help.

Respondent: They don't help and there are certain streets that I don't walk down because I think we'll they don't have footpaths, it's too annoying I'm not going down there or I avoid walking along the main street which is the highway because of the traffic and that sort of thing.

Interviewer: And if you wanted to be more active are there any built features in your area that would help?

Respondent: I think that the footpaths and maybe having a walking track somewhere or something that you could actually go and you'd know that you can do a good walk.

Interviewer: Do you have any parks here that they could link a walking track with? I know you've got the little one coming out but that's...

Respondent: Other than down by the river and Mt [x] there isn't really a great deal but there is land, like we surround the town and I would be happy to donate some, my husband might not be but to offer a little bit of land that a proper park can be put on so people have got somewhere to go.

Interviewer: OK yes because how it was described to me was [town] is that the farms have been planned, designed as the farms are all quite close so they're around that perimeter of [town] so it makes sense if a group of people want or thought that was good idea.

Respondent: Yes definitely. There is a local walking group that I haven't been involved in, it's linked in with the heart foundation but I'm not sure where they go and how they feel about, whether they've found good places to go or walk.

Interviewer: Yes well I actually talked with [x] and [x] at the community health service in [town] because they run the walking groups and I think [x] comes over here because I said to her, well you have all of these things that we're talking about they had to work through and they said they did have, they do have difficulty working through but they've sort of tried to work out a route where it's quieter.

Respondent: Right.

Interviewer: Yes, so we're coming up to people noticing where the pickle is.

Respondent: And I have no problems with trucks or anything like that at all but this road there are trucks, whether they are carrying grain or whatever and the road isn't very wide so they don't have a great deal...

Interviewer: I noticed that, as soon as I left the town it's actually quite narrow...

Respondent: And it's the same at the opposite ends, so [x] Road is the same when you head out towards [town] so the same thing if you're walking on there then you've got...

Interviewer: OK. Now the other side of is the natural features of the environment. If I were to ask you about the aesthetic qualities or the attractiveness of your area, what sorts of things would you tell me about?

Respondent: That I'd probably take it for granted that you live here and so you're used to seeing the trees and the beautiful hills...

Interviewer: This vista.

Respondent: Exactly. You are very used to it and it certainly not something that impacts on my physical activity.

Interviewer: Do you think your local environment is aesthetically pleasing? Attractive near your home?

Respondent: Yes I think so.

Interviewer: Do the aesthetics of your area influence whether you're physically active or not?

Respondent: No I don't think so. Not for me.

Interviewer: And other than you enjoy walking. If you wanted to be more active are there features of your area that might help if they were more aesthetically pleasing?

Respondent: I don't think so, no.

Interviewer: The last area's about safety. If I were to ask you about safety in your area what sorts of things would you talk about?

Respondent: I guess the thing that I've highlighted is traffic and the speed of traffic for where we're located. In terms of safety, my personal safety and [son]'s footpaths they're hard to get up on so there are a curb and there's no where unless you go in on a driveway there's nowhere to actually get up on that footpath so things like that is quite dangerous.

Interviewer: Yes not particularly well designed.

Respondent: No. So they're probably the two things.

Interviewer: So does personal safety influence whether you're physically active or not?

Respondent: No but it impacts how I do my physical activity and when.

Interviewer: And does road safety influence whether you're physical active or not?

Respondent: No but again it's how and when I do it.

Interviewer: And if you wanted to be more active, are there personal or road safety issues that if addressed might help you be more active?

Respondent: So one would be speed limit, so that and it's actually been enforced and I know that there's a big uproar about it at the moment but this end 60 signs what does that mean? So for people I think they interpret it that you can do whatever you like, so that would be the big thing.

Interviewer: And just about trying to do some thinking about these sides of the roads and access to the footpaths as well.

Respondent: Yes.

Interviewer: OK. So before we finish up is there anything else that you'd like to tell me about, where you live, your activity, and the environment that we haven't covered today?

Respondent: No I guess my one thing that I did say before was that I would really like it if the council was proactive in terms of putting together a wellness program and some sort of physical activity options for people because I think, I don't know if you've been in and around [town] but obesity is a huge thing right from children at school to right through to adults and I think that the council has responsibility for the health of the people who live in the community and to help them. So I guess if something came out of it that meant that we actually had some opportunities to do exercise and also socialise and I guess mental health is another big thing that in rural and remote communities and I think that physical activity is shown to help decrease those things and to get people out and socialising in their community. So I think that would be a really good thing and if there was some footpaths done and a walking track and some sort of thing then that would be absolutely wonderful.

Date: 18 October 2011

Duration: 30 minutes, 32 seconds

Interviewer: [interviewer]

Transcriber: [transcriber]

I thought I would start by talking a bit about where you live. So can you tell me a bit about what it's like to live where you do? Your likes and your dislikes?

Oh actually it's a –well this year it's a beautiful place to live. Because it's not a drought. But ah, we've got eight and a half ks of [x] River frontage and it's a beautiful spot. It's not that far from town, which is a lot or substantial towns and [city]. Which is very different to where I used to live on the mainland. It is a yeah, everything is a lot closer. The biggest problem we had when we first came over was um, I suppose, to be accepted. Like accepted socially and things like that. And that was pretty hard. Um, because most, we are probably the only corporate place in the [region]. And a lot of the neighbours are sort of fourth and fifth generation. And yeah, it was a bit hard. But we're all good now.

Oh great. So you live at um, is it [town]? Between [town] and [town]?

Yeah, between [town] and [town]. About nearly halfway between [town] and [town], which is um oh it's a lovely spot.

It's a sheep property is it?

Yeah, sheep and irrigation. Well there's sheep and crops. But it's mainly a fat lamb base ah, set up.

And how big is the property?

5,000 acres.

That's a nice size. And what is the length of time you've been living there?

Oh I've been here for just on three years.

And what are your reasons for living there?

Ah poverty. Ha! Um, well there was a drought. We had ten years of drought. I had a business over on the mainland. And then I was managing properties and the drought just continued. And um, and then my position at a management role in a [x] cattle, had a live export [x] cattle, ah disappeared because we had no water and we couldn't do anything. So they just had to downsize. I went back contracting and then this position came up. And we took it.

Mm. And on reflection, it sounds like you are pretty happy about being where you are now?

Oh yes, but we didn't really have too many choices. And luckily my wife – I was about 50, nearly 50 – and um, if we didn't make a move then – like I was back shearing at that stage. If we didn't make a move – well I had three kids in [school], so I had to, I had to keep putting some money in. Um, yeah so now we're settled. You know, we're here, [name] my wife, she [job] at a [workplace] in town. Or she runs the office at a [workplace] in town. And I run this.

OK.

So then all the kids have grown up, or are growing up; one in [state], one in [town] and one in [city].

Mm. So what's the distance from your nearest town? What would be your nearest township?

A town? Well you wouldn't really call [town] a town. Ah, [town], I think it's about 25 kilometres.

OK. So in our research with men, as I was saying most of it has been done in urban areas and cities, people refer to their local neighbourhood. And I was interested in understanding what the term neighbourhood means to men who live in a rural areas, and indeed whether it's relevant or not. So what does the term neighbourhood mean to you?

Well it's ah, yeah well that'd be [town] and the people, the next door neighbours, property owners and that sort of thing. Yeah, like this is the sort of a neighbourhood I'm in. We um, yeah we spread a bit further now. Like I go down and play tennis at the [place] on a Tuesday night with one, two, about four or five other guys in the [region] here on properties. Plus a lot of guys from down in town, like accountants and the valuers and real estate agents and stock and station agents. Which is a fantastic thing, to get off the place.

Yeah. So just thinking about neighbourhood, and you were saying about [town] – how, if you needed to put a distance on it, what sort of distance would it be?

Oh the actual neighbourhood? To go and visit people – like to [town] is about 6 kilometres.

OK. And what about the word 'community', what does that mean to you?

Ah the word community, well I'm ah, I've got three guys, I've only got one other full time guy and then two casuals here. And the word community - and then my contact with those guys is pretty much daily. But when you start saying word 'community' I'd be including my stock and station agent, merchandise man that comes and sees me, um and we also contract out to the guys that do grow seed cabbages – and yeah they're the sort of guys I have contact with on a sort of daily or you know, every second or third day.

Yeah, so it would be a community of interest.

You're not isolated. There's not the isolation that we used to have.

Yeah. And so if you needed to put a distance around that, that sounds like it might be a bit bigger distance than [town]. Um, what sort of distance might you put on it?

Oh you could put 20 ks on that. Yeah 20, well see they are sort of [suburb] type guys. But then I don't travel to them, they travel to me. But yeah, they come from sort of [suburb] and yeah [town], better say about 35 or 40 ks I suppose.

Yep. Now we're going to talk a bit about physical activity. And um as I was saying it's a very broad term in what it means to each individual. So what's the word physical activity mean to you?

Oh just in my job entails a fair bit of that. There's walking – and I do most of the sheep. Um, with the dogs and I get out and walk a fair bit. And I'm quite conscious of it, because I'm not a little bloke. I'm um now I'm 6 foot 2" and 110 kilos, so I've got to keep reasonably active. And that was one thing that did fall off a bit when I first got here. Um, was I was just that flat out trying to get everything else sorted out, that sort of went by the by. And I wasn't involved in any sports or anything like that. And um yeah so then I sort of realised – I have always been pretty fit, and played tennis and all those sorts of things. Um, but I didn't do that for probably the first two years I was here. And that can get you down. If you're not, you know you know you are not getting off the place and you're not physically doing what you usually do, it does ah, yeah it does run you down.

Yes. But as you say, it does depend on the time, you know you needed to put a lot of effort into the property. And that's what happens.

Yeah. Just one moment [name], please [brief discussion with another person] – sorry about that.

That's OK. Do you consider yourself to be physically active?

Oh yes, yeah.

Yep. And why is that?

Say again?

And why is that?

Oh well I walk. And I walk behind the sheep every day. I play tennis once a week, I ah, yeah there's no days when I'm not sort of breaking into a bit of a sweat.

Yeah. And so where does physical activity fit in your life? Is it of no interest? A priority? A high priority?

I'd nearly say priority to high priority. It's something I try and endeavour to do, more so recently. If I go work down the paddock, working with my dogs I'll get out and walk instead

of just sitting in the ute, talking them out the window. So that's, and those sort of things. I battle a bit coming from the flat country, getting up some of these hills with my old knees.

Yes, they're pretty hilly aren't they? OK. So we're going to go into a bit more detail of that. So in our research when we think about physical activity we think about any activity that lasts for at least ten minutes. That's the evidence of health benefit effect. And causes your body to work harder than normal. So you might notice your heart rate go up, you might breathe a little heavier or you might feel warmer, particularly with those hills. So just thinking about that type of activity over the last two weeks. Now, I'm going to go through parts of your life not to segment your life, just to make sure that we cover everything. Now in the past two weeks have you been active at all at work for at least ten minutes at a time? And you've been telling me yes.

Yes.

Yeah. And that's been walking around the paddocks and various different –

Walking around the paddocks, shearing a few sheep that are stray. Then we've been band marking. And then it's up and down the 9:45 doing wetting and drying the ewes, which is physically, seeing if they've got milk or not, drenching them, jetting them, so that's pretty yeah, pretty full on.

It's a busy time isn't it?

Yeah, so up and back and up and back and all that.

OK. Now thinking about those activities, what you think they, the intensity of them were? Would they be steady, moderate or vigorous?

Um, moderate mostly. Ah, yep.

Ok. And how long would you have done that at a time?

Well, when we get that in that's about four hours, four or five hours every day, except for the Saturday and Sunday.

And is this what you usually do? Or is it one off?

No this is what we usually – well this is only at this time of the year we do that. But we are quite often, we are always doing something.

Yes, it would be other activities that you were doing.

Yeah there's always sheep work and there's irrigators and there's pipe work and a lot of fencing. Because we put our fences up and down a lot and there's irrigators.

And so does it change depending on the season or time of year?

Yes. Definitely.

Yeah, so the activities do. But overall you are still busy during that time.

Winter is harder down here. The winter is a bit longer and colder and that's probably it limits a fair bit of the physical activity.

And um, if you wanted to is there anything that would have helped you be more active at work now? You obviously are very active, but I just needed to ask the question.

Probably a couple of new knees'd be good.

OK. Now in the past two weeks have you walked or cycled for at least ten minutes of time to get to places.

Yeah. I haven't cycled. The country is not, it's good in one direction and not in the other here. I can get down the hills but I can't get back up them. But no, walking. I can't run. I do not run. My knees are beyond that. But I can cycle and I can walk. That's not a problem. And I do walk quite a bit.

OK. So thinking about those activities, um can you tell me about what the intensity of them would be, would they be steady, moderate or vigorous?

Oh it's probably moderate, I'd say. That all depends on the terrain too. Like if you're just cruising down the, cruising along behind a mob of sheep on the flat, that's not a big deal. But if you start hitting a 40 degree grade, ah that's a totally different story altogether. Yeah, that's when you really know you are getting a bit of a workout.

Mm. And the duration of those activities?

Oh it's usually whenever I start that, it's usually an hour. So it's an hour in those set ups and that can be a couple of times a day.

OK. And where would you have done those activities?

On the farm.

Yep. OK. And were you alone or with others?

Usually alone with my dogs.

Yep. And is this what you usually do or was it once off?

This is usually what I do. Whenever I'm doing sheep movements um, I'm not a great one to go for a walk just for the sake of going for a walk. If I can fit it in with something else I'm doing, that's better.

And does it change depending on the season or the time of year?

Yes, we don't do as much of that in the winter. Just the weather and the conditions just don't allow it.

So if you had wanted to is there anything that would have helped you um, be able to walk more to and from places?

Ah, not really here. No, just flatter ground

Yeah, yep. OK. Now in the past two weeks have you been active around the house? So inside or outside? Now, you probably need to think about outside as around the home.

Yeah, well I've got 80 little ram lambs out the back here that are my little pets at the moment. So I cart grain and hand feed all those each day. And the gardening, I don't do that much of. It's mainly, it's a very big garden and I get the guys – it's mainly just mowing. Mowing and whipper snipping. So I usually don't get a chance to do that recently. I used to when I first got here but I just haven't got the time now. But I wouldn't call that strenuous exercise.

OK. Well thinking about the activities you were talking about, the feeding and the lambs and things. What would be the intensity of it? Would it be steady, moderate or vigorous?

Oh steady. There's a bit of lifting in it. Like you are lifting sort of 25 kilo buckets and moving them around and that sort of thing. But it's not a um, you know you don't go breaking your neck to try and do it. You just sort of poke along and get it done.

Yeah. And the duration? How long would you have done that?

Oh it only takes me about 25-30 minutes twice a day.

And when would you have done that? Would that be before the other farm work and after it?

Yeah, first thing in the morning and last thing at night.

Yeah, yep. And would you have done that by yourself or with others?

Yeah, just about really most things I do are by myself.

Yeah. And is it what you usually do, or is it once off?

Oh yeah, we are always, there is usually something like that.

Yeah, around the house to do. And does it change depending on the season and time of year?

Yes, yes. Winters there is not much happening around the house. In the house there is, but not around it.

And if you wanted to, is there anything that would have helped you be more active around the house or the yard?

No, not really.

No, you're busy enough.

I have an exercise bike but that's about it. That's the only thing that I jump on every now and then when I feel a bit guilty for not doing much in the winter.

Right. Now, have you been physically active for at least ten minutes during your leisure or spare time? And this picks up the other activities, like you were saying about the tennis?

Yep. Yep.

Um, now any other activities other than the tennis?

No, tennis is the only one I can get. See there's not too many facilities in that local area or neighbourhood I'm talking about. Even for tennis courts. Or things like that. So [town] there is. But we've actually just made up a thing with the guys there at the [place]. So I'm just doing that.

So you go down to the [place].

Yeah. Tuesday night we go down. It ends up a late night. But it's um, it's good.

OK. Now what would be the intensity of that exercise?

Ah it's full on. That's bloody – when we're playing there's no prisoners. Yes.

Yep. And how long would you play at a time?

We play for about three hours. Probably in that three hours there is probably two hours of really full on.

And that's every week?

Yeah.

And that's of an evening is it?

Yeah.

OK. Um, and OK, now that's usually with others. And is it what you usually do or is it once off?

Now it's all year round that one. And it's weather dependent though. Like if it's a filthy day we don't go.

Fair comment. And if you wanted to is there anything that would have helped you be more active in your leisure or spare time?

Well just yeah, probably some more facilities like that closer to where I live.

Yes. Yes.

You know, if [town] had a tennis court we could probably have another night or a social night, you know during the week, and things like that. That'd be handy.

No, that's really important. And we are actually going to go on to talk about that. The last area to talk about is the physical environments. And so we've talked about where you live and your physical activity. And now I just want to talk about the physical environment where you live and work and play. And when we talk about physical environment we mean all the physical things that surround you. It might be the roads, the trees, traffic, whatever. Um, and some of these things are things that people living in urban areas have said may be important for their physical activity. So I wanted to ask whether they are relevant to you.

Right.

So first of all can you just tell me about how easy or difficult it is to be physically active in your area?

Oh it's quite easy to be physically active on the farm and also as I said with going down to town for sport. But it would be, it would be – we've got, the local town has got a cricket oval, and that's about it. And I'm not a cricketer.

Yes. And that – OK, so I'm going to go through a couple of areas and availability and accessibility is the first one. And this is what you've been talking a bit about. If I were to ask you about places to be active in your area, what sorts of things would you tell me about? Well the cricket oval.

There's a cricket oval there. There's no, there's not a football club that's, oh there used to be, but there's not much of that. Actually, the fire brigade does a bit. They've got the young kids and they do a bit for them. But um, I'm a bit past fire brigade. And my kids are on the mainland. And I haven't got time to be doing, to be getting involved in that sort of stuff anyway. But yeah no, if we had a couple more like tennis courts or even a um, somewhere to hit a ball up against or something like that. I notice that down at [x] school they've got plenty of tarmac area but no actually lines marked or tennis nets or you know like, anyway. But the kids obviously get out there and play other stuff. But I haven't actually seen them.

So there's a lack of availability, but also access – are places easy to access?

Yeah there is. Unless it's um, in the winter time you also remember in the winter time unless it's a facility that's under cover, a lot of the time you are not going to get a chance to

use it. Ah, but at least if it's there. If it is there it would be used. We are actually fixing our tennis –we've got a tennis court here and so that's the next priority, to get it fixed up.

Yeah. So does having – well in this case, not having places - to be active, influence whether you are active or not?

Oh it does to a certain extent yeah. Like I've made a commitment now that I go Tuesday nights, whatever pretty much, down to that. But if I had facilities just down the hill here, like ah yeah that'd definitely, would be more inclined to be doing something.

Yep. And if you wanted to be more more active are there things or places that would help if they were available or more accessible? Like you've said a tennis court but are there any other particular things?

Ah not really. I can do the rest, like a lot of the other stuff, like I can go fishing here. But that's sort of um, that's a pastime, it's not really, you know you're not knocking yourself out when you're fishing. Um, yeah there's fishing here. You can do shooting, there's another one. That's actually – go walking - I don't do it, I get guys on here that do it. But walking with the dogs and that, that's another one I could probably do. And that's fairly physical. Keeping up with the beagles.

Would you do that on the property?

Yes. On the property.

There isn't really anywhere to really walk, other than – off the property is there?

No, you can't really. Unless I go down to town. Like go down to [town]. There's a nice walk, there's nice things there. But that's probably, if I went down there, I haven't actually done it yet but if I went down there I'd probably take my bike. Because it's actually flat enough to go for a ride.

That's true, there's the cycleway down there too.

Yeah. Tell me why they don't make a cycleway out of that old railway line all the way up to Mount [x].

Yeah. That would be good.

It would. Yeah, that would be fantastic.

OK. We'll take that on board. Now destinations. We've been talking a bit about that. If I were to ask you about places you could walk or cycle to from your home, what sort of things would you tell me about?

Ah well I could cycle down to [town]. I'd struggle to get back. It's a bit steep.

Yeah, how far is it into [town]?

About six kilometres.

Yep.

Yeah I could do that. But just, yeah I have got an issue with my knees. So I've just got to um –

Yeah, it's not really very good for your knees trying to get back.

And it's too – I'm alright on the flat, I'm alright up to about 5 degrees, but once it's above that I'm in a bit of trouble.

Right. Alright, so does having or not having destinations influence whether you are active or not?

Ah, yes. I suppose. It's yeah, well it's mainly the topography of the place. Like if it was flat you'd be riding down to there all the time. You can sort of get there in one direction but you can't or I can't get back.

And if you wanted to be more active, um would it help if there were more destinations within easy walking or riding distance?

Oh not really, no. I don't think that would be, no that wouldn't make too much distance to me.

Yep. Yep. OK. And just a couple of more areas: neighbourhood function and design. If I were to ask you about the built or man-made features of your area, what sort of things would you tell me about? That's the sorts of things about roads and footpaths and things like that.

Oh the roads. Well we're all pretty good up here I think. Um,

You're on a dirt road yourself?

No, no we're on the highway.

Oh Ok.

So just off the highway and just a gravel road up to the house, it's only about 800 metres. Yeah, so we don't have any issues with that. You can't really walk or cycle on the, well beginners, you wouldn't get on that [x] Highway with the log trucks and everything. That's one limiting factor for that. There'd be no way you could fit a cycleway on it anyway, on that road. So that's why, as I said if I was going to go for a ride, I'd throw the bike in the ute and go down to [town] or somewhere a bit flatter that I could – you know, a cycleway that I could have a bit of a go on, than trying to do it up here.

Fair enough. That's a good comment. And if you wanted to be more active are there any built features in your area that would help?

Oh yeah, if they just put a bit more of a sporting centre at [town] or [town], that'd um, yeah just get the community a bit more involved. There doesn't seem to be much community involvement in any sort of organised sport in the [region], to my, what I can see.

Yeah, now a few people have commented on that. Because we are, at this stage we're talking to men who are living in rural areas but earlier in the year we were talking with women. And that was a comment that came out quite loud and clear. So it's interesting. It's good that you've um been talking about that. It just does seem to be a bit of a lack around the whole [region].

Yeah.

Yep. Um, OK. The other side of it is aesthetics. If I were to talk about the aesthetic qualities or the natural environment, the attractiveness of your area, what sorts of things would you tell me about?

Oh, beautiful pastures, the river, the trees, my animals. No, we tick all the boxes there.

It's beautiful. So what river are you..

The [river].

You have actually got the [river] going round there?

We've got eight and a half kilometres of [river] river frontage.

Wow. Beautiful.

It's massive.

Beautiful. That would be tempting to wander down to fish in it.

Oh it is. And you can actually, if you get in the river, you can get on a little punt and go for a fish or whatever. But you can get in there and you could be anywhere in the world. You get in there when it's nice and quiet and with the cliffs all around you and it's lovely.

Yeah. So you've got a really beautiful spot actually on your property.

Oh several. Several really nice ones.

Yeah. So do the aesthetics of your area influence whether you are physically active or not?

Yeah, we do. Yeah, I just get out and do it. Like it's my workplace as well. So it's um – yeah I walk as I said. I just do the walking thing like I said, and I try and blend that in with the other.

And if you wanted to be more active are there any features in your area that might help if they were more pleasing or attractive?

Oh no, no.

It doesn't sound like it.

You don't get it any nicer.

It's pretty beautiful. Now the last area is safety. If I were to ask you about safety in your area what sorts of things would you tell me about?

Oh just the highway is the one that um if that was um yeah – it's the one thing that where our turn off is is right on the top of a hill on a bend, so that scares me, and the big log trucks on that. And the rest of it, the other limiting, there is not too much other safety, like on the properties safety is not an issue. It's just the outside influences that are – like that.

Yep, it's more the road safety. When you are wanting to cycle.

Yeah, that's the main one that springs to mind here.

So does road safety influence whether you are physically active or not?

Oh not really. Not in that regard, no. Because as I said, the country here, like my front drive is too bloody steep for me to get up on a bike. I can get down it no worries, but I can't get back up. I could I suppose, but I'd have to walk it, walk the bloody bike up.

Yeah. And does personal safety influence whether you are physically active or not?

No, not really. Not really.

So if you wanted to be more active are there any personal or road safety issues that if they were addressed might help you be more active?

No, not look obviously –

Because we've talked about the road being too narrow.

Yeah, now look it's a reasonable road but it's just got that big you know log truck traffic and everything. It's just not a friendly, not a place for bicycles. Or not for me anyway.

No, I've had that comment from a couple of others as well. They just don't find it very friendly. They find another place to ride as you've done. So that's the last the question. So I just need to check if there is anything else you want to tell me about where you live, the environment or your physical activity that we haven't covered today?

No. That's about all I do. Most of it is involved with what I do on the farm. And the other is um, yeah I just do the tennis thing and probably ah, increase that. It'll probably be you know

two nights a week, one night in [town] and one night down in town or something like that. But that's about yeah. And I think you've pretty much covered it.

AUDIO RECORDING ENDS

Date:	3 October 2010
Duration:	59 minutes, 24 seconds
Interviewer:	[interviewer]
Transcriber:	[transcriber]

So I thought we might start by talking about the place where you live. So can you tell me what it's like to live where you do and your likes and your dislikes?

Yep. Sure can. I live about 11 kms out of [town], at [town]. I've got a 25 acre hobby farm, mostly bush, and a few acres of land. A northerly aspect, beautiful, been there for 16, 17 years. Um, we moved up there when all the boys were home. And now they've all shifted. To be honest with you we're getting to the stage now where we're looking to get out of it because of the amount of upkeep to the place.

It's a lot of work isn't it?

A lot of work. I'm getting to that age now when I want to spend more time out in the boat, out in the camper, building this and doing that. But I find every time we have a storm, I got trees coming down over the fences. I've got a huge place to mow, I've got whipper snipping, I've got sheep to shear and all sorts, water pumps to fix.

So just trying to do this is like having a second job.

It's been fantastic. Absolutely love it. We just in the process of putting it on the market now for that reason. And the fact that I love, well I'm 53 now. And my place is on a side of a hill, so everything you do is hard yakka. Probably good for you, but hard, hard yakka. And we'd like to move probably down to town and get something with less maintenance, and enjoy life also with that sort of thing.

So that would be more round [town].

[town], anywhere but preferably flat.

Flatter.

Flatter. Because I love push bikie riding and that sort of thing. And where I am, because I'm up a hill, wonderful coming down, not much fun going up.

And that's when you have the home run.

But yeah, it's beautiful.

OK. And what were your reasons for living there? Was it ..

Ah mainly for the, always wanted a few acres out of town. Well it was 16 years ago, I was mid 30s and I lived on a small place in town, that was very congested and three boys that couldn't ride their motor bikes or anything like that. So this place come along and we bought it. And we've had a ball up there. But the boys have all left now. They all say "oh Dad, you can't sell it, it's too good". I say "well you come up and maintain it. Come up and give me a hand to maintain it". I'll miss it. I know I'll miss it. Love the place, but you know I've got to be sensible about it.

It's for a time isn't it?

That's right.

And you enjoy it for the time that it is.

Indeed. Indeed. And there's wood to cut too. And a lot of it I do enjoy doing, but um yes it's taking too much of me life just maintaining it. Every weekend I've got work, lots of work to do. And I work here, not that I work particularly hard, this is office, but when I get home I'm sort of you know, got to do this and fix that.

Well that's the thing with land, it never stops. There's always something to do.

Yeah. So there's a water pump too. That's a big problem, you know lots of people say oh your rates and tax are cheap up there, you know you've got no sewerage, you've got a septic tank and no water. And I said but yeah, I'm up for pumps every two or three years.

Yeah, you've got to maintain it.

And the septic tank wants maintaining. And you know and then you don't get a lot of less in your rates and taxes for living up there. I think now we're paying $1,000 a year when I think here they are paying about $1500. If I work out me pump and all that sort of thing and the fuel to run it and …

Let alone the wear and tear on you.

Yes. Sure. Sure. So. But ah, yeah no, it's good.

And um what's the distance from where you are to [town]?

11 km.

11 km.

Exactly.

OK. Now in the research as I was saying, um it's mainly been done in urban areas and people often refer to their local neighbourhood. And we are interested in talking to rural people about whether that's, you know what neighbourhood means to them? And indeed if it's relevant. So what does the word neighbourhood mean to you?
To me it always means me direct neighbours, the people that I know within close proximity which rurally I suppose, I'd have four up there that are neighbours. Well I mean the closest one would be 150 metres away. And they get further away from that.
Yes. So what sort of distance would that be?
Um, well I'd say the first one, from house to house is about 150 metres, the second one is about 250 metres and the third would be about 400 metres. But I actually go past their driveways to get to my place. So they're my, mine's a no through road.
..you actually go past, so you'd be…
It's a no through road and the very end of the road is my property. So there's three, four, different houses I go past to get to my property. Oh, one is a driveway that leads up into the bush. I don't know where they are.
So it is relevant to you in terms of you've got neighbours.
Yeah, there's only one that we really get on with. The others are as green as green can be. And we fell out big time with them when we first went up. It was really bad. Because they were sort of my age then when I came up, and all their kids had left and what have you. And we went up there with little boys with motor bikes and all the rest of it. It was like "oh, shock, horror, the noise you're making" and you know, we had the police up there and all this carry on. We had to look into the environmental act and see how far you were allowed to ride a motor bike within close proximity of somebody else's property. And oh..
Oh that would have been really hard.
Yeah. So there's only one that we get on well. And they're the same as us, they got kids and a few motor bikes.
Yeah. It always helps when you've got the same as somebody else and interests too. And what about the word community. Does that, what does that mean to you?
When I think of community, I think of direct community up there. I think of the [town] community and the [town] community hall which is up there. But to honest with you there's no, like unlike [town] and a few other places, they have like a monthly meeting at the hall. [town] don't, they keep to themselves sort of. The Hall ..

So there's a building there.

There's a building there but it's never used. Never used. It's maintained by workgroups once a year sort of thing. And I think there is a badminton club that hire it for about $1.00 a year just to use it like. But no, there's no community as such. There's a few people in [town] that I know, but no-one sort of gets together as such.

OK. So if you need to put a distance around that in terms of community, what would it be?

Oh it'd be 5k square.

Yes, so [town] itself.

But see even [town], you'd have about 5 or 6 houses next to one another and that is [town]and all the rest of them are all over the place. You know they're just like commissioned homes, but like old brick houses that were built there. And that is the township of [town]. And we're all spread all out and about around it.

OK. Now I want to talk about physical activity. And um as I was saying it's a very broad term. It means different things to different people at different times of your life. So what does the term physical activity mean for you?

Any kind of exercise, I guess. I mean my physical activity, other than what I do up and around the house, and I don't do as much as what I should. Um, I tend, I go to the gym twice a week. That's how I keep fit. I walk every day before coming to work. I go down the beach and I walk 2km.

Wow.

Yeah, but it's not enough. I know it's not enough. Um, I tried to actually do more walking around here. You know because if there is a job happening, if I got to and visit a job and it's 300 metres over there, I walk over there. But nine times out of ten I got to take the car. And like when I'm on holidays which I am now, I took the dog yesterday and I rode from [town] to [village] along the footpath, and loved it. That's one of the reasons I want to come down town. Because I can do more of that, what I can't up there. I'm stuck on the side of a hill. So yeah. Any kind of exercise, is physical activity. The gym is really hard. We're in a group, oh most of us are 50 and over, include the instructor. Because he is doing it all the time, he is superfit. And when he trains hard it's really hard for us to keep up. And we do. And I'm absolutely exhausted. In fact I've been going for five years and someone said the other day, does it ever get any easier. I said "it never has. There has not been one time that I've

walked out of this gym after and said gee that was an easy night". He's ruthless. He's really really hard.

He really knows what he's …

Actually he's an ex Council employee that went and done a degree at university. And got his whatever it is, to run gyms. And he's a return to work, he return to work people who are crook and sick and all the rest of it. And he's a hard case. He's good. He's fair, but yeah. And that can be, come the middle of winter and you get home from here, and you get the fire going and the house nice and warm and you watch the news, and you think "oh gee, I wouldn't mind the some tea. But I can't. I'm hungry, I got the gym in another half hour."

Oh so you go home and then you come back down for the gym.

And that can be hard some days. Bloody hell. I don't want to go. But if I don't, I've only got to miss one day and I'll suffer the next day more. And then twice a week. If I miss the Monday, I'll go Thursday and it's double hard. It truly. And I've got a back problem. I've had a back problem for years. I'm a [job] by trade and that really, when I do that it's no fun at all. It's a week of misery sort of thing.

Yeah. So keeping up your exercise actually helps that?

It does help it. But I'm beginning to think now I've got to be really careful. Because there's a few exercises I do that can aggravate it. And when I do it, it might be just "oh shit, I've aggravated it". And the next day, I'm flat out. I've had it.

So getting to know what you can and cannot do. And then finding out I guess what other things can substitute. I've got a back condition too.

I think most of us have.

But you've got a very good reason for it.

Yeah, stupid bugger. If I knew what I knew now – the things I used to do, you know. Oh dear oh dear.

Do you consider yourself physically active?

I consider myself physically active. But I'd like to be more physically active.

Yes. Because there's a combination of all the activities you've talked about, walking, the gym, but also all the work that you do on the property.

Yes. It's my job too. Like most of it, more than two-thirds of the day is sit, and doing audits, there's no real physical activity in it. I try to. I'll go for a walk at dinner time sometimes as well but it is just difficult to do.

And where does physical activity fit into your life? Is it of no interest, or a priority or a high priority?

It's a priority. It truly is. I got a wife that doesn't and I'm always onto her to do more. She's on her feet all day. We had this pedometer challenge here a few weeks ago. We all had one of them and I was trying to get me 10,000 steps a day and I was struggling to do it but I was. And she was doing 15,000. I couldn't explain to her, "but you are standing and walking in a shop all day" I said, "you're not". You know she gets home and her feet are sore and her legs are sore. I said "but you're not" – she thinks she's doing the steps and she is, but they're not good steps, you know, they're not. She needs to stride out more and get the heart rate going a bit. She just sort of stands around. But anyway.

Mm. It's hard when ..

You women like to try and tell us..

Well often you get home really tired when you've got that sort of a job.

I know. She is. She's very tired.

So in our research, when we think about physical activity, we think of any activity that lasts for at least ten minutes, for your evidence of health benefit effect. And causes your parts body to work harder. So that's heart rate up. Might be huffing and puffing a bit. Um, temperature a bit warmer. OK. So thinking about that type of physical activity over the last two weeks OK, I'm going to ask you about different of your life. Not to segment your life, but just to make sure we can collect all the activity you're doing. So um, in the past two weeks have you been active at all at work for at least ten minutes of the time?

I couldn't say it would be at work. But it would be dinner hour, and pior to work. Definitely.

OK. Certainly your dinner hour. And I don't mind where we talk about your walk before. We can do it under this or we can do it under another..

Well I start work here at, supposed to be here at 7.30. I'm at the beach at 7 o'clock every morning. And I walk striding out for half the hour, 25 minutes, because it takes me 5 minutes to get here. So it's 25 minutes and that's walking hard. Really going for it. And then I get here and there is probably not a lot here that happens. I mean I don't do, I mean even if I go out for a job, there wouldn't be 10 minutes of walking around that I do at the job. I get out of the car, I walk over to the guys and ask a few questions, do an audit, get in a bus and travel off again. There's not a lot at work, no.

So unless you get to go for a walk in your dinner time..

Sometimes dinner time I go for a walk, if time permits. Or you know if I sneak me lunch while I'm working, before I have dinner and then go for a walk. I often do that. Or if I want to get a salad roll I walk over to the shop and back which is, it would be just 10 minutes there and back.

Yeah. So thinking about those activities, there is three of them you've named. Um, what would be the intensity of those, steady, moderate or vigorous?

It would be, moderate.

The walking out on the beach would be.

Moderate.

And the other activities?

Ah, yeah, I'd put moderate. Because what I do, when and if I do at dinner time, I don't just lull about. That's my time, I go pretty hard at it.

And the duration? How long?

Well all up then I suppose – are we talking just during the work hours?

Yep.

Not till I get home after?

No. We'll talk about that.

Then it would be three quarters of an hour a day.

OK. Yep. Yep.

That'd be it.

And half an hour would be the walk in the morning.

Yep. You know the walk in the morning, and probably quarter of an hour around here. So that's quarters of an hour all up.

And is that what you usually do?

Yeah.

And does it change depending on the season or the time of year?

Yeah. If it's pouring down with rain I wont go. I don't go, nah I can't get wet and then come here all sopping wet. Um, but I don't care if it's blowing a gale or it's freezing cold, I'll go. In fact I rather like it. I prefer it when it's cold than I do when it's stinking hot in the middle of summer. Because I'm sweating and I come back with the air conditioner on ..

Yeah, that's not much fun. OK. And if you wanted to is there anything that would have been more active at work? Now I know before we started you started talking about that there were things happening in [workplace].

There is, like I say. But I find it hard to support too. Because I do gym twice a week. At me own gym. And I feel like because I've been there five years I wouldn't like to, even though this other gym is free.

Oh so [workplace] is offering gym. What else are they ..

Tai Chi. I suppose I should. I just can't see any sense in –

Are they offering that in work hours?

No, after hours.

After hours. OK. And do they have a concession price to employees?

It's free to employees.

It's free? Oh, wow.

Free to employees. And there's free membership to the [gym]. Which is a gym over the West. And they've got swimming twice a week, just about to start up through the summer months. And that's for [workplace] employees, their wives and even grandkids and kids or something.

Really?

At the [town] swimming pool. And they've got an instructor there who used to be a [workplace] employee who just does it for the love of it. So it's actually, and I should go to that too. But it's time.

It's time. Yeah, well that's what it comes down to.

I do two nights a week as it is.

And thank you for being really honest. Because it is really, you know they are really valuable comments. Because one we are talking about, you know everybody seems to be talking about this. Is the sit down at work stuff. But also people are really busy at work. It's really hard to then sort of look at other activities. And some people aren't activity people or work activity people. And then there is all the other stuff you have got to do in your life. Yeah.

So there's two nights a week, probably an hour and a half each night for the gym. If I start going to another two for the swimming and the tai chi, well there's another. And because my wife doesn't support it, well I'm not home much either. You know, I come home, I'm

gone.   Well that's not real good for marriage either. If she could support it enough to come with me, but she's not into it.

OK. So how long has [workplace] been offering this?

Oh about two years.

Oh really.

Lifestyle, health and wellbeing program. Yeah it was a lot of [colleagues] – big business in [state], they tend to run with these sort of things, and try to get other businesses to support it as well. Every year we have a corporate challenge. That's playing cricket on the beach and basketball. And they try and get all the other [business] and [business]. And they'll get a team of eight. And we'll go down there and play them. It's pretty good, pretty good. You know, it's well run and it's a bit of a contest and there's a BBQ afterwards and a few prizes. And they do that every year. So, but I think [workplace] look at uphold that sort of thing and try and get the community involved because we are a [workplace]. And it's pretty good. As I say $15,000. It's a fair budget every year just for that.

Absolutely. No, that's very good.

In fact when it was put – they are trying to spend some money at the moment and they said we was down at [workplace] a few months ago, and they just had a push bike in there. Two push bikes in their office. And I said "what are these for?" And they said well we do a lot of interaction between the works depot and their admin. So the guys, instead of taking the car to take the mail, they go on the push bike to take the mail. And so we've actually come up with that idea here too.   Thinking about doing that ourselves.

Oh OK. Because I was going to ask um you know, like coming back to yourself, like OK is there anything that would help you personally, to be more active at work? It's obviously something you've been thinking about.

I would. I would. But I still, once again it would probably only be for dinner time. I would probably jump on the bike and whip down to the main street. And I couldn't see meself. Because I've got to take a helmet with me, me folder with me, my steel cap boots. And then I got to get meself on a push bike.

That's fairly tricky. So it's the nature of your work. Yeah. OK. And um so in the past two weeks have you walked or cycled for at least ten minutes to get to or from places?

Um, no. I've done it, I mean I cycled yesterday. But it's not for a reason.

Not for going to and from places.

Because I can't from home.

Yep. Yep.

Oh it was fine years ago. I used to ride to work and back everyday. From [town]. And I loved it. But then I was hot and sweaty when I got here. You know, I've got to live in these clothes all day. By the time I got home I was absolutely buggered, because it's up hill all the way to [town]. And I can't do that anymore. But I tried. And it was good. I enjoyed it but it was just too much.

Too much yeah. And if you had wanted to is there anything that would have helped you, walk or cycle to and from places? Other than not living where you are.

That's what I say. If I was on the flat, I would. I would definitely do it everyday. I would love to do it. But [town] is a constant grade up hill all the way. So you get little exercise on the way down. You're just free on the way down. But absolutely buggered by the time you get home.

It's not the time of the day when you want to do that.

No.

But, you do.

Yeah, yeah.

In the past two weeks have you been active around the house or yard? So at home, either inside or outside for at least ten minutes?

Yep, mowing the lawns, chasing sheep, cutting wood. And all those type of things. Just general maintenance round the place.

So thinking about those activities, um what would be the intensity of them? Would they be steady, moderate or vigorous?

Ah, they'd be moderate. They'd be moderate. I mean past when I pulling out fence posts yesterday – what a shit of a job that was, it was hard work you know.

That's a bit more than moderate.

It was hard, but then you know pushing and all the mowing, well it's not really vigorous but it's on the verge of being.

Depends on the season?

Yeah, well that's right.

This sort of season it gets a bit hard.

And then like well you know, walk down the pump. Well that's down a steep hill, down to the creek and back. Well that is bloody, you know, I got to take a couple of spells to get back sort of thing. That is very vigorous work.

So what would be the duration of those activities?

Oh, on a day to day basis – like you say it depends on the year. Because until daylight savings, by the time I got home I've had half an hour, it's dark. Or it's so bloody cold. You know. It's freezing, you get the fire going and get inside. But then this time of year, I got a couple of hours when I get home and l love it.

Then you tend to do that.

Yes.

So on the land, when you come home that's what you do, yeah

Yeah. I can't stand coming home on a good day and going in and putting the telly on and watching something. Can't stand it. Even if there's nothing to do, I'll go out and find something to do just to get out there.

So you'd normally do um this time of the year, an hour an hour and a half at night. And then um, weekends.

Yeah. Pretty well all weekend. Just all weekend. But it's only moderate. You know it's nothing, you know, it's not really hard physical stuff. Just moderate type of exercise.

OK. So that would be every day.

Yep. Pretty well.

Yep. And would you have done that by yourself or with others?

By meself.

And is that what you usually do, or is it one off?

No, it's what I normally do.

And does it change depending on the season or the time of the year?

Definitely.

We've already talked about that. So, the more light the more you do. And if you'd wanted to, is there anything that would have helped you be more active around, inside or outside? It sounds like you are pretty well active as it is.

Yeah.

Now the other part is, have you been physically active for at least ten minutes during your leisure or spare time? And those are the sorts of things we were picking up about the gym,

and the cycling. Are there any other things? We've already talked about the beach walks in the morning.
No, I mean when I get time I take the boat out, but there's not really sort of much physical activity in that. I got a motor cycle, I love riding me bike. There's nothing much in that either but I love doing it. And that's about it. We've got an old vintage car, we are in the [x] car club. I used to use that a lot, but we don't anymore because I just don't seem to get the time for it anymore. So I need to get rid of that actually. So, no, not a great deal. It's not as if I go kicking a football or playing some kind of sport. I don't play any sport anymore. I used to for years, but I don't anymore. Too old. Pull up too bruised. I had a young bloke up last week, because I got a lot of trees down and I don't need the wood. Had a young bloke up, about 23, and he said "oh I'll take your wood". I said, "you bring up your chainsaw and you help yourself to it". Anyway, I could hear this chop, chop, chop, chop, chop. I went down and had a look, he was like a bloody thrashing machine. And I thought I remember being able to do that. Now if I've got to cut a metre of wood, it will take me – I cut wood from time to time, periodically throughout the year - it will take me hours to cut a metre of wood now. Two or three swings of the axe. So I go, "oh Jesus, that's playing up my back" or "I'm out of breath, I have to have a spell, sit down, you know." When I got up there fifteen years ago, I was like a thrashing machine. I remember cutting bundles of stuff all over the place. And used to enjoy it, used to love it. And I still like it but it just knocks me out. I just can't do what I used to do.
Well thinking about those couple of main activities you were talking about, which is um, well we were talking about cycling and the gym. And do you want to include any of the others? The motor bike and the others?
Oh not really the motor bike, there's no real exercise…
OK. Well if we focus on those two, um the intensity of them, steady, moderate or vigorous?
The gym is definitely vigorous. It's full on. Ah, and the cycling or the walking is just moderate.
Yep. Yep.
You know, I don't um, when I get on me bike I don't just sort of trickle around, I keep going, but I'm not, I don't exhaust meself. In fact I can't, that's why I go to the gym. Because I cannot – my brother will train by himself, run 10 km every morning and can do it. I can't find the drive. But if someone is there watching me, in a group exercise, I'll do it.

Oh that's an important point. It does really help some people.

Oh it does. Like I think oh it costs me $15 a week. I could be doing this at home but I know I wouldn't do it. So I've got to go to the gym.

No, you don't get the same effect.

That's right. And hence that's why you see all that gym equipment on the tip. All these people go and buy it. Oh I can do that at home!

That's right. But thinking about those activities, um how long would you have done them in the last couple of weeks at a time?

Well, um the gym twice a week every week.

How long is that for?

Well it's two hours.

OK. Two hours per session?

No, an hour session.

So two hours a week. Yes.

Two hours of that. And daily I do well particularly over the holidays I was an hour and a half on the bike yesterday. But normally, three quarters of an hour a day. With me walking and the little bit I get around here.

And how often would you usually get to bike ride? Sounds like it would be more on the weekends now.

On the weekends, the weekends. But there might be more opportunity now that the weather is coming up good.

More opportunity to put it in the car and bring it down.

That's what I do. I put it in the back of the ute. And bring it down. Park down at the wharf and go to [village]. And the dog runs. I've got a whippet dog that runs alongside me too.

That's great. I wouldn't train my dog for that.

Oh he's good. Well a whippet.

He just goes for ever.

He is just bounding along. And I'm going quite hard and bloody hell, he's running backwards and forwards and out and still keeping up.

Is this on the tracks along the…

Yeah. Through the parks and on the tracks. It says in the parks that they should be leashed but he's as harmless as can be. It's not as if he's a dog that'd attack anyone. He's terrified of other dogs actually. But he's a good runner and he'll be chasing a rabbit every now and then when he sees one. And yeah, it's good. But I actually pulled up, I went yesterday, and I actually pulled up sore when I got back in my lower back. I'm alright now, but I actually got off the bike and walked for a good way because it was playing up me back, yesterday. That's me biggest fear in life that me back is going to fail me where I can't do even that. That's my biggest fear. And I know it is gradually getting worse as I'm getting older. I was talking to someone the other day and I "where's your wife, I haven't seen her" and he said "her back is that bad now, she's more or less just compelled to being inside. And she can have an operation he said, but the doctors said you keep taking your painkillers as long as you can because if you have the op. there is only a 15% success rate." And I thought "oh Jesus I'd hate to be like that". You know if you can't do a thing, can't get out of your chair or anything.

So you try and um…

Oh I try and strengthen it yeah.

Very wise.

That's the best thing you can do for a back injury. People say, when you've done it though, a lot of people say "oh the best thing to do is to keep moving around". Well if you've really done it bad, you can't. It just makes it worse. You've got to just sit there and rest for a day or two, until you start getting a bit of movement around you and bring it on.

And I think you know when people have those sorts of chronic injuries they know their body the best and what needs to happen. And I always find that when you get into that spasm, it's the muscles around it that spasm and whatever, you are trying to stop that happening. Because that makes it four times worse.

Oh I was on the floor one day there, really bad, two or three years ago and I was on me stomach on the floor and I got a spasm and it just, I've never felt pain like it. And it hit me about three or four times. I just couldn't stop it. And me wife was like "What's wrong? What's wrong?" And ,oh, just terrible, just terrible. And frightening. And I thought there's nothing I did to bring it on, I was just sitting there. Oh God. Yes.

Mm. It's often the most innocent things. I mean I often say you know if you had a good story to go with it and like you are on the top of a mountain and um you know the Himalayas

or something, that makes a good story. But it's often not that that happens, it's a little turn. But anyway we try and do what we can and keep as well as we can.

For sure.

And um, OK. So you've talked about um when you do those activities, are mainly sort of the weekends or before work. Um, and you've said about where and why. Often come down here. And um, would you do that um with others or by yourself.

By meself.

Other than the dog of course.

Yep. By meself.

Yep. And is this what you usually do or is it a one off?

Sorry.

Is it what you usually do, the walking and the cycling?

Yes.

And does it change depending on the season? Now you've already said with the walking if it's raining you don't.

Only if it's pouring down. If it's raining -

There wouldn't be too many when it's really pouring here.

No, normally it's good. A couple of times I've got down the beach and I've looked over and it's looking pretty black over there and I've taken a risk and get half way over. And I literally run back to the car.

Fair enough. And if you wanted to, is there anything that would have helped you be more active in your leisure and spare time? Now you've mentioned apart from having more time, and that's one the things that you are looking for.

If me wife would be more involved then I would do more. I know that for a fact. If she would, if she would, um she keeps saying she will but it just doesn't happen. Because then you are doing it together. You are still together, but if I keep going and doing these things, it's – she's not doing it. And of course the place suffers for it too. Because I'm spending more time when there's things to do around the house, so yeah, I want to do more. In fact…are you a local?

No. I live in [city].

Ah, right.

In fact I originally come from [state], so I've only been living here but I chose to come to [town] to [state] to live because of, it's just wonderful. We live in [town], right round the corner from [reserve] and the Rivulet, we've got a [dog breed] dog, so every morning you are walking. And then you just walk down into the city.

It is nice. I like [town]. I hate [city], but I like [town]. Um, there's a group of guys that get together every Saturday morning. You see them if you go down the shop to get the paper and the main street now, they are all older than me, they're in their sixties most of them. And they got all their riding gear on. Their helmets. And they sit outside the coffee shop with their bloody $6,000 push bikes. And they all have a coffee and then they ride. There's two groups and one is almost semi-professional and the other groups are just amateurs. And they will just take off and wind about the canyon which is a hell of a bloody ride. You know, up the [36:39] canyon and the other guys will probably go along to [village]. And then they will come back. And I thought "geez I'd like to do that, just to get in." And me brother is doing it and he says that it's a good bunch, a good bunch, you know.

So this is some of, if you moved down here, gives you more options to..

Oh I could still do it while I'm up there as well. But it's just time factor. [name] me brother has just started it. And he says it's bloody good. And I said "the only thing is those bloody bikes that they ride, you know the handle bars like that". I said "my back wouldn't last". He said "you need to get a, what's called a cross-trainer, which is like a racing push bike with the handle bars further up and you actually lean on them". And he said "most of them have got them if you have a look". He said "I've got a spare one of those, so I'm actually just going to see if I can handle that".

Because I find like when I used to ride one of – I've got a more upright bike – but when I used to ride one of those, I'd tend to ride on the top of the handle bars anyway, rather than go down.

No that's right, but even that, that is still falling a bit. Because I mean the bike I've got is only a cheapie mountain bike. But because I hurt me back last week and hurt it again yesterday, so but I like the idea of it. I don't mind pushing myself, no. And it's beautiful here on the [region]. They ride along to [town] along the coast road and that.

*Yeah, no I've heard people say when I was doing the interviews earlier in the year, you know with all the women up here and I was talking about the bike track to [*town*]. And also the road along to [town]. It's just a beautiful…*

Oh the big picture is from what I've heard from Council is to run a bike track from [town] to [city].

That would be something.

That's going to be fantastic. Well you've got one in [city].

Yeah.

My brother said he used to ride to work every day on it and he loved it.

Oh it's great.

But this would be great too. I mean [town] has got a good one now from here to [town]. It really is good. And it doesn't go through any built up areas, it's along the beach and bush and that. Beautiful. I love it.

Yeah, everyone was talking about it. So I had a bit of time one time I was up here. I was up here for a couple of days and I had a bit of time so I actually went and had a look. And it was great. And was thinking you know, yeah lots of people would use it. And even people who were sort of living out of [town] used to say that come the weekend they would put the bikes and the kids in the car and go down there.

We got the new bridge now as you probably saw. Well the bike track is going to link up to that and go under the bridge and then over west and follow the coast around.

Great. That would be fabulous.

It is. It is. And that's another reason why I want to come to town. It would be there, right at me doorstep. You know.

Oh I wish you the best with that one. It sounds a really good move. To be able, yeah it's a different activity, different time in your life.

Well it's not strenuous. And when you are older you know you don't, I love to jog and all the rest of it but I feel like me knees and me hips and everything, I can't do that. Well I do, I do on the treadmill but I can't – treadmill is a lot different from the concrete.

Yeah. Absolutely. OK. So the last area is about the physical environment, the environment that you live, work and play in. And when we talk about physical environment, it's all the physical things that surround you. It might be the roads, the trees, shops, traffic, houses, whatever, parks. And some of these things people living in urban areas have said that they might be important for their physical activity. So we are wanting to check out whether that's um, works for rural people as well. And whether it's relevant to you. So first of all, can you

tell me a bit about how easy or difficult it is to be physically active in your area. And you've already touched on that. It's pretty difficult.

It is.

Because of the slopes and the gradient.

All my physical activity up there is work related. I don't do it to keep fit. Well, ..

No, And it's hard yakka too. It's the slope.

Yeah, well it's not that I've chosen to do it, it's that I've got to do it. When anything I do down here, I've chosen to do it.

So really, yes exactly. And then you need to get in the car to get down here to do that.

Yeah. That's right.

OK. Now I'll just check out a couple of areas. Availability and accessibility of places to be active. If I were to ask you about places to be active in your area, what sorts of things would you tell me about?

Up home?

Yes. As opposed to down here. Really what you were saying then is that you come down here.

Yep.

And here has got a range of recreation facilities like you do things in the gym and you've already talked about the Council, there's an indoor swimming pool, parks, and cycle ways.

Look I walk down the beach and I take the dog down the beach, on the dog beach. Yeah, I sometimes walk out to the breakwater with the dog.

Which bit is the dog beach here?

Ah it's on the far eastern side of [x] [42:07] Creek, right out to [x] Point.

So it's the one from the eastern side, and it's from where water street comes down.

Yeah.

Yeah. I think I know where.

It's right along there. It's out near [x] Point where an old fishing track was. So from [town] right out to there. Well right on the beach here and out to the breakwater there. It's all beach.

Oh OK. And then there's a section that isn't, and there's a section further on.

That's right. That's the bike track. That is the bike track. See how it goes all the way to [town]?

Yep, yep. OK. Now, so there's plenty of places that are available to be active down here. And accessibility, are they convenient like in terms of opening hours and cost and things like that?

Oh yes. Oh yeah. Yeah it's not a problem.

OK. So does having places to be active influence whether you are active or not?

Definitely. Definitely. Because I choose, on that basis when I go. So yeah. I'm not into bushwalking so much. There's lots of people go bushwalking, and I've got a lot of bush up there. But I've never been much for bushwalking. I just like easy going.

Yeah, well the wonderful thing about here is you've got all the choices.

Yeah, that's right.

And if you wanted to be more active are there things or places that would help, if they were available or more accessible?

What sort of things are we looking at there?

What you've been talking about is more you being able to move to a place so you can take up those possibilities. But is there anything here?

No.

Somebody has mentioned that it would be nice to have, like if there was an indoor pool was

Open more? If we had a proper swimming pool, like something like [city] has got. I mean our pool is only a school pool.

That's what I was saying actually.

Yeah, a swimming pool would be good. Um, I know I've got access to the squash court. I used to do that a bit. But that's too hard. You know I can aggravate my back doing squash.

It's hard on the body.

Yeah it is. It's a bit like netball. It's all joints. You know, stomping.

You don't bounce as much.

I played soccer for 25 years and I love that. And I know there is a group of guys, over 50s who have got a sort of a fun roster and I've been asked to go and play that. But I'm too [45:11] of me back, again. You know if I went over bad or kicked or something and wasn't quite braced up or something happened. Because I'm wearing it the whole time. I can feel it now. Sitting here now I can feel it.

Yeah. OK. Another area is destinations. If I were to ask you about places you could walk or cycle to from your home, what sorts of things would you tell me about? You were saying you can ride down to [town], but really what you were more likely to do, there isn't really a ..
If I ride down, I'd love to ride down, I'd ride down every day, but I got to get back.
Yep that's right. So there isn't around your area, but there's plenty more if you're down here. And then there are destinations.
Even if it was flat up at [town]. So if there was an incline and then up there was tablelands, it wouldn't worry me. I'd go up there. But there's not. It just keeps going up and up an up until you get right up to [46:21] and you've got the mountains after that. There's no flat. There is down your way, [city] way, you've got lots of flat areas, the [region] and all that sort of thing. But this is all hills.
So, does having, or not having, destinations influence whether you are active or not?
Well, yeah. It does.
Because not having them up there, having them down here, influences, it does. Yeah. OK. And if you wanted to be more active would it have helped if there were more destinations within an easy walking or riding distance?
Definitely.
And that's why you're planning to move. OK. Neighbourhood function and design. Now if I were to ask you about the built features of your area, what sorts of things would you tell me about? I'm talking about sort of footpaths and lighting and walkability, and those sorts of things?
Here or up there?
Well you can pick.
Well up there, all I've got like I say, because I'm on the end of the road the only place I can really walk other than through bush, is the unsealed road in my property. Whenever I go out, I've got to come back again. That's boring. It's the same thing everyday, it's dusty, it's not good underfeet. You know ankles, you can roll the ankles or whatever. There's twigs and stuff on the side of the road. Then a car goes by and you get covered in dust. So it really is boring.
It's not great incentive. Yeah.
I just get bored of it. I can do the same thing down here and walk that beach road. I've been doing it for 4 or 5 years and I don't get bored of it. But I do up there. And I don't know

why. I don't know if it's just because the sea's there, there's more to see, or what. But I get –

No, I understand that. I've lived in areas where you know you've only got one main access, and it's just -

You come home on that road and then you get out of your car, and you've got to walk back that way. I said to [name], I said, "even if I could leave me car down the bottom of the letter box, which is probably 2ks away, and walk home, that'd be alright. And then possibly walk down the next morning to get me car, but then there's a security thing too. The car is on the side of the bloody road.

Yeah, yeah, no you can't do that. No you wouldn't want to go on the main road by the sound of it.

No, no.

OK.   So um, and there wouldn't be footpaths and lighting up your way?

No.

And there is a point about what we call street connectivity.   It's that it's a lot more interesting. And a lot of people have commented. They live in an area where there is a main road and that's it, it's really difficult and not very interesting looking. If you can find places where you can get to it's a lot more interesting.

That's right. I'm quite happy. Sometimes I've even just walked through streets. Just through the suburbs. Just walk through the streets.

Whereas down here there is always something interesting.

Sometimes I knock off near dinner time and then walk over [business] down, cross the train bridge, over the [suburb], cross the road bridge, up through [x] Park and back. It's half an hour. It's good.

It's very walkable. Like all the comments I've heard, [town] of all the places would be – definitely in the study areas – [town] is very walkable.

It's a nice, definitely a nice town because it's got very little industry here and you are in between [city] and [city].

Even better. And a lot of people have said that they've actually chosen to live here for what this spot has to offer. So do the built features of your area influence whether you are physically active or not? So you've been actually saying yes they do, up in [town]. And they do down here, but they are very different.

That’s right. Totally different.

One discourages you.

Exactly.

And if you wanted to be more active are there any built features in your area that would help?

No, only the fact that once that footpath, the keep progressing the footpath. I’d go to [city], I’d ride to [city]. Not a problem. That’d be great. Because it’s flat all the way and you know exactly how far, you can set your agenda to it. It’s not long enough.

No, that would be great. They’ll get there, but it sounds like there’s not only Council support for it, but there is so many people who are interested.

Sure.

It would be great.

Yeah.

Now the other side of that is the aesthetic side, the natural part of the environment. If I was to ask you about the aesthetic qualities or the attractiveness of your area, what sort of things would you tell me about?

Once you get down here at town, um I just think it’s being in among people, people and their dogs.   People stop you and talk about your dog and their dog and what have you. Every morning there is three or four guys that I see every morning doing the same sort of thing as me. Most of them are retired. I say “how you going?” “yeah, good”, “how’s the weather?” “Yeah good”. In fact if I go there and they’re not there, I’m like “oh I wonder where he is? I didn’t see him today”. There’s girls, a couple girls that are joggers – I don’t know how they do it, just running and running and running – every morning and they are up at about 5 o’clock and they are still running. And where I walk is just a part of the course that they still run. And I say g’day to them every morning. And when I’m up country I see nothing of that. It’s just bush. I might see a wallaby. You know there is just nothing to keep me going at all. Down here, I see the sea. I’ve got a boat and I know the sea is calm, I might go and take the boat out later on today.   So it’s all those sort of things. There’s a shop there, I might get the papers while I’m down there. But up here it’s just the bush, the bush, the bush. There’s nothing that changes.

And that’s an important point. Like coming back to what you were saying, you meet people and people have just, one of the things people have been saying is you know – um, one

woman was actually saying I choose to go on walks and part of that is to say hello to people. And I think that is an important point about meeting people. And with dogs, you will always meet people. It was funny last night I talk a walk down the beach from where I was staying and the first thing I clapped eyes on when I got the beach was this [dog breed] and of course I'm feeling a bit deprived of my [dog breed]. And you always start talking.

You have a conversation.

Absolutely.   So do you think the natural environment is aesthetically attractive, near your home?

No. Because I'm bored with it. It doesn't change and it's every day.

But down here it's something quite different.

The only time I go for a walk on my property is if I have got to go and do something.

And ah, do the aesthetics of your area influence whether you are physically active or not?

Yes, we've been saying that it does.

Definitely.

And if you wanted to be more active are there features of your area that might help if they were more aesthetically pleasing?

Well, there's nothing up home. I can't – well what more is there aesthetically pleasing that I can do up home?

Yeah, yeah. Whereas down here it is aesthetically pleasing.

That's right. Yeah.

One last area, safety. If I were to ask you about safety in your area, what sorts of things would you tell me about? You've been talking a bit about that.

Up home?

Mm.

Only underfoot, being steep and undulating and that sort of thing. And because I'm walking home down Seaview and me foot tend to…

And even your dirt road not very..

That's right. Not real good underfoot. Dust and um, yeah. I remember when I went up there, and old fella that I boarded off, he was in his 70s and he had a walking stick. Not a stick, just a pole and everywhere he went he had this pole. Because he was unsteady on his feet going down his property. And I thought you know, another ten years I'll be probably like that too.   I don't think of it now, but yeah.

So does personal safety influence whether you are physically active or not?

Not at all. I go for a walk of a night. I know they talk about thugs and all the rest of it and I know it happens, but I'm still of the opinion I can look after myself pretty well. I haven't been approached or..

It's a pretty safe area, [town].

Pretty safe.

And does road safety influence whether you are physically active or not?

Not at all. Not at all. No, not in town anyway. We don't have the amount of vehicles you have done South. There's no real main road. There is one main road, but you know I was thinking there this morning, if I stop at the traffic lights in me car, on a normal crossing, I got to wait for four or more cars to go by, I think "come on". Bloody hell. If I was in [city] it would be 50-60 cars.

People have said more too, that people are um a bit more gracious about people who are pedestrians and cyclists. They're not so much in a rush to get there at a certain time. So it's a good spot from that point of view?

It is.

So that's the end, but before we finish is there anything else that you'd like to tell me about where you live, your physical activity, or the environment you live in that haven't covered.

Pretty well got it.

Yeah, I think so.

Um, see there was a time only three or four years ago that one of the guys here used to go shooting down the [river] [57:08] places like that. But as a young bloke I had guns and rifles, I used to do a lot of shooting. But as I get older, I am just more happy to watch than I am to shoot. They say, "come on" and I say "no I haven't got a gun license" and they say "nah, don't worry about that, we'll fix you up with a rifle, can you shoot?" I said "oh I can shoot". I got down there like you know, next thing I got a bloody gun on me a 22 and all the rest of it and I said to them, I said "don't think I'm a wimp. I don't mind youse having a shot and going for your life" I said, "I just don't want to shoot. I just want to enjoy the environment." So I just walked behind them and watched. It didn't worry me that they were shooting roo, they do it, you know they don't kill just for the sake of killing. They give them to their dogs and make a few roo patties out of them. But we were walking miles and miles and miles through the bush. And then the land owner we used to go and do it on, he changed his mind

and locked it all up and it doesn't happen any more. And I think what a shame, and I loved it down there. I just like the comradeship and the camping.

Where was all this?

Oh at a placed called [town], which is you're heading to the [region], and just before you get to [village] turn to the left. And it's up in the bush, up in the beautiful country. They shoot deer and all the rest of it, but you know it's all licensed and that. But you get to that age now, like I mean I will still shoot a bloody feral possum one day, I will, but you just don't want to kill anymore. Some of the guys might actually still do because they've never stopped, but you have a stop or you just don't do it for a few years and you think "poor bugger, poor bugger". But I will, if I've got feral problems I'll still shoot, yeah got to. But my place, I can hardly run anything on it, any sheep because they've got to compete with the wallabies. It's alive with them at night time. But that was something that we used to do nearly fortnightly, was head down there all the time. And oh absolutely buggered by the time I get home, I was walking and walking and walking.

Yeah, because you kept on walking.

And you ruin the back too, 20-30 kilos of ..

Oh it's weight bearing exercise. But look thank you very very much.

Oh no that's fine.

AUDIO RECORDING ENDS

Interviewer: Good. So I thought we might start by talking a bit about where you live, OK. And can you tell me what it's like where you do, your likes and your dislikes?

Respondent: OK. We live at the back of [town] on 17 acres.

Interviewer: Yep. So you're just outside of the…

Respondent: So we're just outside…

Interviewer: … the immediate sort of, what do you call it, a little hamlet, village?

Respondent: Yes. It was called [town] a long time ago, but it's now been incorporated in to I suppose the greater [town] township. So the property is set behind a

number of other properties, so you enter through an easement and then it's the land that opens up behind the row of houses along the street. So I guess being set back like that we have our privacy, but we also have very good neighbours that we know and we can access them in whatever way we want or need and vice versa. We like the privacy. We like the peacefulness I suppose of being there, and we like having space and garden and I suppose room to breathe in that sense. It does mean though that if we want to go down in to town to go to the park or the beach or the shops, we do need to actually get in a car. We could go down on a bike but it's a really, really steep hill, and so potentially coming back up again or even going down if you lose control and it's not good for… the kids are quite young still. So we do tend to get in to a car to go in to town which I prefer not to, I prefer to be able to walk.

Interviewer: And by town you're talking about…

Respondent: [town].

Interviewer: [town]. So in terms of distance, how far are you from [town] itself?

Respondent: We're one kilometre inland off the highway. So I don't know if you know but [x] Road… so [x] Road comes up becomes [x[Road and heads off towards [town], and so we are on… just off that road in a place called [x] Lane. So it's about a kilometre inland of the highway, so it'd be another kilometre coming down the hill and then down into [town]. It probably takes 30 to 40 minutes to walk in to town which we do sometimes, but I wouldn't want to do the walk back up the big hill with all the groceries.

Interviewer: Yes. That's the thing, that's a steep incline. Now you were saying that you've got a house on land, how much acreage have you got?

Respondent: There's 17 acres.

Interviewer: Mmm, nice size.

Respondent: And a part of that is pasture and part of it is bushland and a substantial part of it is garden and orchard and so on, so, mmm.

Interviewer: And how long have you been living there?

Respondent: It'll be ten years in August.

Interviewer: And your reasons for living there?

Respondent: My husband and I both were raised in big cities. I come from [city] and he's from [city], and we, I suppose, were looking for an alternative to being in very busy built up areas and we sold our house and bought a campervan and went travelling around [country]…

Interviewer: So this was from…

Respondent: From [city]. We were living in [city] after we married, he immigrated. And we spent a couple of years travelling periodically, so on and off.

Interviewer: Yes.

Respondent: And I have to say that north west [state] was the first place that we visited, and we came back a few more times during that two years and then bought a house and moved here. So we like, I suppose for [husband] it is reminiscent of some of the landscape and climate of the [country] but better, not as cold. We both felt the lifestyle was better than being in the city, and there was work available and the housing was more affordable so we could buy something that had land with it rather than just a little house like we'd had in [city].

Interviewer: And you have access to work? [city] seems to be quite a centre for people…

Respondent: We both work at the [workplace] in [city], at least we did then. We now work at both [workplaces] since it's become sort of amalgamated to the [x] Service. So we also travel to the [workplace] in [city], and luckily 'cause we live in [town], it's actually not too far in either direction, but in those days the [workplace] was separate, so we started off work there. Why do we like it? I guess…

Interviewer: Why did you pick [town] in particular?

Respondent: Why did we pick [town]? [town] a very pretty little town. It's very appealing visually and as we… so we picked it because we liked the look of it initially, but as we have lived here and met more and more members of the community and become a part of the community, we really like the people as well which is good. It's obviously not too far from work, but at that time we didn't really want to live in the town where we worked because having come I suppose from a city we were used to having anonymity if you like, and a separation between work and personal life, and we kind of didn't like I suppose the idea of running in to [clients] and things all the time. Now having said that, that obviously happens and we've got used to that and it's actually all fine, but that was a very foreign concept to us when we first came. So we wanted a little bit of space but not too much because of the on call, you know, driving backwards and forwards. So [town] was a good distance and just a very pretty little town, yeah.

Interviewer: OK. Thank you for that. So in research with women living in rural areas, people often refer to their local neighbourhood, and I'm interested in understand what the term neighbourhood means to women who live in rural areas, and if it's relevant at all. So what does the word neighbourhood meant to you?

Respondent: OK. Neighbourhood to me is your immediate neighbours. It's where you live, it's your street, so it's the other people who live on [x] Lane. And then there's the community that you live in, and my community is [town].

Interviewer: Yes, and I was going to ask you about community too. So neighbourhoods' that, and may be it…

Respondent: It's that very small little group that you see when you're putting your rubbish bin or your recycling or collecting your post or walking your dog. They're those people that you bump in to very, very proximate to where you live.

Interviewer: That's really clear. And if you needed to put a distance around that, for you in [x] Lane, what would sort of be the distance?

Respondent: [x] Lane.

Interviewer: Which is how far?

Respondent: Which is, how many houses?

Interviewer: Or distance. Half a kilometre, a kilometre.

Respondent: Not even. As in it goes on much longer, but there's a little clutch of houses and then it's paddocks, and then there's a few right around the corner down the end, so…

Interviewer: So there's this cluster.

Respondent: There's probably about 11 or 12.

Interviewer: A couple of minutes walk of each other?

Respondent: Yes, yes.

Interviewer: Yep. OK. And what about the word community, what does it mean to you?

Respondent: OK. The community I suppose are the people that I actually spend more of my time with than my neighbourhood, that live locally. They use the same shops, they use the same parks, walk on the beach. People whose children go to the school that my children go to. So the parents and the children and the siblings of those families that live in my area that access the same, I suppose, shops and the library and the bank and the post office and all that. That is the community, so the people who live in that township that I see regularly and have some interaction with.

Interviewer: And if you needed to put a distance on that, what… that's [town] as well as…

Respondent: [town], yes. It does...

Interviewer: Yeah, [town]. So it's a couple of kilometres, [town]?

Respondent: Yes, yes, it is, it is. Now having said that, there are people that I consider part of the community who don't live within [town], who might live at [x] Creek or [x] Bay or somewhere, but they bring their children in and they use the shops and so forth and I see them regularly. But I wouldn't consider where they actually physically live as part of the area that the community is in.

Interviewer: Yep, yep. Do you have a school in [town]?

Respondent: Do I have a school? Yes.

Interviewer: There is a school?

Respondent: There's a primary school, a secondary school and there's a small private Christian school, an Adventist school up on the hill.

Interviewer: OK. Now I want to talk a bit about physical activity, and I was saying, that's a very broad term. So we're interested in what does the term physical activity mean for you?

Respondent: OK. I see it as two things. I see one as physical activity that you use in doing things, that sounds terrible, productive things. And then there is exercise for the sake of exercise. So going to the gym or going running or swimming or whatever it is, where it is purely and simply about actually exercising. And but most of what we do every day is actually the other sort of physical activity.

Interviewer: That's a clear way of thinking about it, yes.

Respondent: So and one might keep you generally kind of nimble if you like, but necessarily give you cardiovascular fitness, whereas the other is, well, is about cardiovascular fitness or for some people it may be about shape like body building, you know, weights and things like, whatever people chose to exercise in.

Interviewer: For whatever reason. That's fine. And do you consider yourself to be physically active?

Respondent: No. I'm definitely not fit. I don't do any regular real exercise, and my level of physical activity changes to according to the season, according to whether it's school holidays or not, and what we are doing. So it's very responsive if you like to what's going on in our lives. So when we're on holidays and we're camping and we're walking and bushwalking da, da, da and out with the kids, we get a lot more physically activity. When it's spring time and there's a lot of work to be done in the orchard and the veggie garden and just down in the

bush and things, then again maybe it's chopping wood or whatever it is, then I might have, you know what I mean, I'm more physically active. And of course in wintertime we don't get out as much, the days are shorter. Even taking the kids to the beach or the park or whatever is more limited. So all our activity is more limited, although because the children do like particular classes, they get their regular exercise, whether it's swimming or circuits or whatever it is, but I don't. So become less fit as you might imagine during the winter months and probably a bit fitter, not what I call fit during the summer months or more physically active in the summer months.

Interviewer: So those changes. And where does physically activity fit in to your life, is of no interest, priority, high priority?

Respondent: Unfortunately, when I was younger it was a high priority and I used to exercise very regularly and go to the gym every day, all of this sort of thing. And as my life changed and priorities changed, it's come further down my priority less, particularly since having children who have gone flying right up to the top of the priority list. And I am mindful that it is something that is lacking in my life, and periodically do something about it and then it sort of fades, you know what I mean, but the last time…

Interviewer: Yeah, 'cause there's other things to do in life and other priorities.

Respondent: There are other things to do. And it sort of increases, you know what I mean, like, you know, and if I do cut out something else to make time for, for instance, exercise, all I find is something else creeps in to that gap instead. So it's clearly, even though I know it's important, it's clearly not as high up my priority list as it needs to be to be able to keep that time protected for it. I will use that time for something else related to family almost invariably, sometimes work, but it's nearly always something to do with the family.

Interviewer: Yes. Well, that makes a lot of sense in terms of... and I think that's one of the things people are really sort of starting to say about being time poor of…

Respondent: We're all incredibly time poor, it's just life is very full and very busy, and if you're a normal person, you do tend to put your children and your family ahead of your own to an extent, and the exercise thing ends up a bit further down than it used to.

Interviewer: Well, that's why when you were saying… and I think it's really valuable what you've actually been saying about it, changes with seasons and your activities. 'Cause it's very useful to find out that sort of fluctuation that… and the sorts of struggles people have with it. OK, it's really nice when some people have got it sorted out, but we're really interested in finding out the sort of struggle that people have around. That's much more…

Respondent: And the other issue I have is…

Interviewer: … meaningful.

Respondent: … that it has to be interesting or fun. It's just like a lot of this sort of running on running machines, which you can do in winter, I just find that incredibly tedious and boring. It doesn't matter if they put up TV screens in the gym, or sometimes if it's music that I really like and you get in to the rhythm, that's OK. But, you know, I know you're exercising your heart and lungs but I just will spend the whole time thinking of all these other things I could be doing, and I would actually rather be with my family and doing things with them than isolating myself. So I need to do physical, well exercise anyway, with company so that I'm feeling that actually that time is productive, and, you know.

Interviewer: I think that's a really good example you've given, I really understand that. Yes.

Respondent: So we'll get out all the bikes and we'll all try and go do a bike ride in summer, but winter, by 5 o'clock it's getting dark and it just doesn't happen after school, you know, it's cold.

Interviewer: No. It's busy and it's been a long term for them too.

Respondent: Hasn't it. Oh, I know. It's already… it's winter tomorrow and they're still at school for term 1, it's just, you know.

Interviewer: I come from [state] so I'm used to, you know, we've had…

Respondent: The four terms.

Interviewer: …four terms, yeah. And especially this time… I appreciate that it's great for kids in [state] 'cause they've got that longer summer break, and that's when you've got the light and the sun and that's great and it reminds me of being a kid, that's the sort of long summer holiday you get. But it's this time in winter that makes it very hard.

Respondent: And they could really do with a break in the middle of winter, because I mean at the moment they're all full of coughs and colds, but it will just go right through term 2, and they'll end up actually losing a reasonable amount of school time anyway. If they just had that break, but anyway, we'll see what the government decides on that.

Interviewer: Yeah, it'll be interesting. So in our research, we've talked about, you've been talking about physical activity, when we think about physical activity we're actually thinking about any activity that lasts for at least ten minutes.

Respondent: Uh huh..

Interviewer: That's the health benefit effect as you know. And it raises your heart rate, your body temperature very good at this time of the year I might add, and a bit of huffing and puffing. So what I want to do is just think about the type of physical activity for ten minutes at a time over the last couple of weeks, yep, and I'm just going to look at different parts of your life, not segment your life but just to make sure that we pick up on all the different sorts of activities that you might do, OK?

Respondent: Uh huh.

Interviewer: And also check whether it's changing with the seasons too, 'cause I think that's a really valid point. So in the past two weeks, have you been active at all at work for at least ten minutes at a time?

Respondent: No.

Interviewer: OK.

Respondent: No, at work, no. Never active at work.

Interviewer: Are you working part time or full time?

Respondent: Part time, which involves sitting down a lot.

Interviewer: Yes, and that's what we're getting to hear. I mean my job's the same; you just sort of sit, yep.

Respondent: Or if you're not sitting you're just walking, but that's just walking and that doesn't get me huffing and puffing and my heart rate up at all though.

Interviewer: OK, and so if you weren't active at work, why not? That's sort of more about the nature of your work isn't it?

Respondent: It's an office job, yeah.

Interviewer: OK. And if you'd wanted to, is there anything that would have helped you be more active at work?

Respondent: There are… I know once a week there's a lunchtime walk that they do.

Interviewer: Is there? OK.

Respondent: It is on a Tuesday which is the day I generally don't work, but the point is I guess I could think about changing which day I worked to access that, or no reason why I can't, I suppose, go and do that on another day. But, again, there might not be anybody to do it with, they might all do it on a Tuesday and so forth. So I probably could…

Interviewer: And I think it's always hard…

Respondent: … be more proactive about that.

Interviewer: Yeah, though it's always difficult being part time because you are often time pressured in your day.

Respondent: Well, I tend not to actually, you know, you don't stop for lunch breaks do you, you just work straight through because you want to get however much done, but not stay back beyond your hours because there's a reason why you work part time which is you've got to pick up kids or whatever it is, so…

Interviewer: Yes, I have things to do.

Respondent: You do, you work straight through your lunch break don't you and often don't actually ever eat lunch which is, you know, eating pattern's another whole

thing. But, yeah, so, thinking, taking an hour out or half an hour and going, I think I'd rather work through, finish and, you know, pick up some groceries on the way home or something. You're always juggling all your little time slots for what your priorities are, and as we said before, the activity side of it tends to slip further down that list.

Interviewer: OK. Now in the past two weeks, have you walked or cycled for at least ten minutes at a time to get to and from places?

Respondent: Nope.

Interviewer: OK. And if not, could you tell me why that might be?

Respondent: Again, I think just… my husband's been away which always makes things a bit busier.

Interviewer: It does doesn't it. There's more juggling involved.

Respondent: So you tend try to, yes, and I didn't take any time off work while he was away this time, so trying to do more in the same amount of time. So, no, it hasn't happened. Probably just felt there wasn't enough time. The weather was fine, it wasn't that. I'm just thinking very hard about the last two weeks. Even when I've had time I've stood around watching soccer for an hour, you know.

Interviewer: Or if it helps, pick another couple of weeks if that… That's OK. That's fine.

Respondent: Yeah, no. Sometimes I will down in to town or go on a bike ride, but really, really uncommon to be perfectly honest, yeah.

Interviewer: No. As I say, it's really good to talk to everyone and the sorts of juggles and balances they have.

Respondent: It's even been a while since I've actually taken the dog for a proper walk because she's quite happy actually just racing around the paddock whilst I'm doing other things, and so she gets her exercise whilst I'm doing whatever I need to do with the chickens and different other things, so, but, no.

Interviewer: And what that change at all depending on the time of the year?

Respondent: Yes, the longer evenings, we tend to often go for a walk after the evening meal with the kids and the dog, or go down to the beach a bit more. Bike rides more as well, and it also tends to be like, again, with the kids long summer holiday. So that's when I'm more likely to take some time off work or I might modify my hours so that maybe I do half days and then have afternoons off the kids and we'll do things, that sort of thing rather than do some full days. So there is more flexibility I suppose to access family time and we might do some of those sorts of…or go on bush walks which we do. It's even been a while since we…I'm trying to think when we last went up to [mountain]. But when we go up to [x] Mountain, which we did… whenever that last weekend was when had three days a row, we did like nice big walks every day, but it wasn't in these last few weeks. So it's patchy I guess.

Interviewer: OK. That's fine. When you do do that walking, like you mentioned the activity, I've got walking down to [town], now do you walk back up or…

Respondent: Sometimes. It depends…

Interviewer: Or you arrange to get picked up and go back up the hill somehow?

Respondent: It depends on whether I've got a whole lot of stuff I need to carry or so forth.

Interviewer: It's always the trouble.

Respondent: But if it's actually just walking because let's get some exercise or whatever, then I'll just walk back up as well, or we might drive down in to town and then [daughter] and I will walk back up and the boys will go up in the car and whatever.

Interviewer: So when you do that, could you describe the intensity of it? Is it steady, moderate or vigorous would you say?

Respondent: Steady.

Interviewer: Steady, but vigorous going back up.

Respondent: Oh, no, look that would probably still be steady too because, as I mentioned earlier, I tend not to do things by myself so I have my daughter with me who's eight.

Interviewer: Yeah, so that it makes a very big difference.

Respondent: So we will go with her pace and that is actually one issue with physical activity with the children. As you know with very little ones, you know, it's one step forward two steps back, and you're walking at their pace and stopping and looking at things. So there is no sustained period where your heart rate is up if you like. Although as they're getting bigger, that is improving, so, but she's the eldest one and she's eight so probably there is a little bit of…

Interviewer: You've got a little while to go, yes.

Respondent: Yes.

Interviewer: Often women say that, you know; when we're with the kids, well, mmm.

Respondent: You are out about a lot or in the parks but you're not actually exercising.

Interviewer: No, no. They often say, well, if I've got the opportunity, you know, by myself, yes. OK. So there was that exercise and occasionally you go for bushwalks and things like that.

Respondent: Yes, yes.

Interviewer: So would that be moderate?

Respondent: That would be our preferred form of exercise.

Interviewer: Would that be sort or steady or moderate or…

Respondent: That'd be moderate. Again because of the children, but they're pretty good. The children are obviously fitter than we are because of their regular sports and things that they do. So even the five year old, we've done some… depending again on the season because some of the walks that would normally be, you know what I mean, you wouldn't take them on in winter…

Interviewer: No.

Respondent: …that might be moderate ones in summer are OK for the littlelies, in winter the mud is too deep and, mmm.

Interviewer: They're just too hard, yep.

Respondent: Yeah, and they go at a fairly good pace. So actually on the bushwalks, we're actually pretty good and up at moderate, not hard, not exhausting because of them. We don't want to put them off, we want them to enjoy it and want to keep doing it.

Interviewer: No. So how long a walk, those two activities walking, how long would you do that at a time?

Respondent: Which, sorry?

Interviewer: You've talked about going down into [town]…

Respondent: OK.

Interviewer: … and also the bushwalks, how long would they be for?

Respondent: All right. The bushwalks would usually be a minimum of an hour, and then up to sort of three hours probably with kids at the moment.

Interviewer: They're doing very well.

Respondent: They are, they are. Look, and it's all very weather dependent and all the rest of it. But they're actually really good but you've got to pick interesting ones, you know.

Interviewer: Yep, yep.

Respondent: A good thing about [state] is that the scenery is very changeable, so it is actually quite interesting, and they like the boardwalks and the steps and the different things so it's quite easy. Down into [town], it's about 30 to 40 minutes in each direction and you stop and do your bit of shopping or we like to do the little walk along the front where it's got the little bits that you read about the history, and the kids like to look at the little pictures and read those, so the bit in between is not keeping your heart rate up, and to be honest, the one that really does is the walk back up the hill afterwards, and there is a bench half way up where you can sit and have a rest…

Interviewer: Ah, that's good.

Respondent: … which my daughter is always keen to do.

Interviewer: So it becomes a bit of a marker of…

Respondent: Yeah, so you do it in two lots.

Interviewer: And so how often might you do that, those two activities?

Respondent: Probably the bushwalking more often than walking and things locally, 'cause usually actually you're on your way to get something and dah, dah, dah. So the bushwalking, probably about every, depends what [husband]… how much he's away, maybe about every six weeks, something like that, but not as often as we'd like.

Interviewer: And with the walking down in to [town], how often might that be? Every few months?

Respondent: Yeah, yeah.

Interviewer: Yep, yep, and that's weather dependent too.

Respondent: Yes, absolutely.

Interviewer: OK. And generally your exercise is you're with the kids and your partner and/or, yep.

Respondent: Yes.

Interviewer: OK. And if you wanted to, is there anything that would have helped you walk more often to and from places?

Respondent: Probably if I didn't work.

Interviewer: Yeah, you'd have more time.

Respondent: It's more time, yes. And probably if there wasn't a big hill. And I know that shouldn't put you off 'cause that's actually really good having the big hill, but you'd probably do it more often if there wasn't a hill because you'd carry all your stuff back, you know what I mean, or you could take a bike and load it up, and that it really is. There is another way, [x] Road, which I've not done yet because there's no footpath and I'm not a great road rider with my bike, and so the edges… the road is right to the edge and then there's these culverts. So I haven't done that yet but it's another… it's an alternative route, still a hill but it's more windy so that the gradient is…

Interviewer: OK. It's a bit gradual, more gradual.

Respondent: And I'm thinking, I've been thinking more and more about it, I should actually go for rides down in to town and come back up that way.

Interviewer: And have you got a footpath on your road when you go in to town?

Respondent: Once you hit the highway, then there's one the rest of the way down. But inland of the highway, there's not, but it's a fairly wide road, so you can just sort of go along the edge of it.

Interviewer: OK. Now in the past two weeks, have you been active around the house or yard? So at home either inside or outside?

Respondent: Yes, I can actually say yes to this.

Interviewer: And can you tell me what you did?

Respondent: All right. Things I do are mow the lawns with a push mower, I don't do any of the ride on stuff. Pruning, been pulling out all the tomato plants, cutting all the raspberry canes. Oh, we dug out some plants on the weekend; I was stiff and sore after that. So it's been mainly gardening, so there'd be digging, clipping, some climbing, and picking lots of fruit and getting down underneath to pick up rotten fruit to take out.

Interviewer: It's that time of cleaning up isn't it, the garden?

Respondent: It is, it is. So we've done… what have we done pruning wise? What did we prune? We did the cherries I think. I think that's that time of year we did cherry trees. And just, yes, and pulling out things that needed to come out. I'm trying to think what else we've done.

Interviewer: So you've got a bit of work with the little orchard that you've got?

Respondent: It's orchard and veggie garden. Cleaned out and I've got two chook sheds, so cleaning out the stuff out of the bottom and putting new straw in.

Interviewer: Yeah, that takes, yep.

Respondent: I don't actually chop wood very much, but I suppose barrow loads of wood backwards and forwards between the wood shed and the wood box and filling the wood shed before too much of the rain comes. Yes, I've pruned all my hydrangeas but that's not very… there's a lot of them but it's not very, you know, they're right at that sort of level.

Interviewer: And I guess inside's the inevitable housework.

Respondent: Actually somebody comes and does the housework, so I don't do that either.

Interviewer: Lucky you.

Respondent: I know, very lucky me.

Interviewer: But there's usually other things… you still don't get away with it completely.

Respondent: Oh, look, there's still all the making beds and flipping cats and dogs and muddy paw prints, and I don't know if you've had the mouse plague where you at the moment, but…

Interviewer: No.

Respondent: …anyway there is a… every day, yesterday two mice and a rat, I have to say one of our cats brought in. So I don't know if crawling around under dining tables and picking up bits of entrails and cleaning up counts as physical activity or not, but I seem to be doing a lot of that at the moment.

Interviewer: I think it might.

Respondent: So it's all bits and bobs, you know, tidying up after the dog and just, it's bits of stuff.

Interviewer: So thinking about those things, which is mainly sort of the gardening and the orchard work and some of the inside things, what sort of intensity would you put to those activities as being?

Respondent: It depends on what they, like it…

Interviewer: Steady, moderate, vigorous?

Respondent: Yes, they would be steady, they'd be steady 'cause lots of carting backwards and forwards and my husband's cutting the canes and I'm carrying them all

and putting them in the trailer and jumping up and down, and so all that sort of steady stuff for that. Not on that weekend that we've just done, but fairly recently, and it's again weather dependent, when you have those bad storms and winds and all the branches come down out of trees and there's lots of tidying up and dragging big branches up the hills to where we tend to chop them up and sort them out or whatever. So that sort of work can be more intensive. Again, if there's a slope involved then everything is more intensive.

Interviewer: Yes, absolutely.

Respondent: But steady generally I would say.

Interviewer: And how long would you have done those activities for?

Respondent: This weekend just gone by, I would have spent three hours each day in the garden both afternoons doing things.

Interviewer: And so how often, that would be two days in the last fortnight?

Respondent: No. No, it's more than that. It'd probably be at least, oh, probably two days a week, or two half days a week, there would be that sort of activity, and these are sometimes the things that I do, like when the kids are at school if I'm not working.

Interviewer: So that would be four times, yeah, or more by the sound of it.

Respondent: And again it's very seasonal, so sometimes there's a lot to do.

Interviewer: I was going to ask you, yes, does it change with the season and at the present moment it does, yeah.

Respondent: I mean for the while there I was having to mow the lawns every week, and now of course it's cold, I'll probably have to mow them one more time and then that will be that until spring comes. So it is variable, but then there will be other things to do as well.

Interviewer: And so you sometimes do them when the kids are at school during the week and also at weekends.

Respondent: Uh huh, if they're like…

Interviewer: Is there a particular reason why you do it at those times?

Respondent: Depends what the activity is. If it's the something the kids don't like being involved with, I'll do it when they're at school, because what I hate is being outside and doing things and the kids sit inside. And if they're, particularly the little boy, it's all electronic screens and things, you know, or computer or whatever it is, and he'll just sit for hours for as long as I'm outside doing things and I really, really don't like that.

Interviewer: So you prefer to pick something that…

Respondent: So I pick things that they like to be involved with to do on the weekends when they're home. So on the weekend they were helping pick the figs and with the raspberry canes to a degree but they're a bit prickly. He pulled up a whole row of carrots for me and things like that. So they get involved and he went and picked up all the wind fall apples and so on. So there are things that... and then he'll sit around and eat apple and chat whilst I'm getting on with other things. So I pick and choose the jobs…

Interviewer: Yes, indeed.

Respondent: … to try and get them outside as well.

Interviewer: Fair enough.

Respondent: I'm probably much better about making sure they get exercise and fresh air than I am about myself.

Interviewer: So who do you usually do your activities with then, these activities? You were saying sometimes the children and sometimes your partner.

Respondent: Yes.

Interviewer: And sometimes by yourself.

Respondent: And sometimes by myself although we do, yes, either by myself or with the family.

Interviewer: And if you'd wanted to, is there anything that would have helped you be more active around the home inside or outside?

Respondent: Again, just time.

Interviewer: OK. Now I just…

Respondent: There's no lack of jobs.

Interviewer: … want to check if there's anything else we've missed. Have you been physically active for at least ten minutes during your leisure or spare time? Now…

Respondent: When.

Interviewer: Yes, the last two weeks.

Respondent: No.

Interviewer: No, it doesn't sound like you might have very much in the way of time for leisure and spare time. But you've also started telling me what you're doing in terms of walking and things. So is there anything else?

Respondent: Although, oh no, I really enjoy like the gardening and all that sort of thing, so whether that counts or not but it probably doesn't really get my heart rate up. It's good for general kind of, you know, but not cardio vascular fitness. I don't think so, not in the last two weeks. I'm trying to think. We've had a lot of birthdays in the last two weeks and it's all been…

Interviewer: That's fine. We've covered gardening and walking and various different things. That's fine. And again if you'd wanted to, is there anything that would have helped you be more active in your leisure and spare time? Like more time.

Respondent: More time. More time. It's not lack of… I don't think it's lack of availability of things to do or places, you know what I mean, like there's, you know.

Interviewer: It's time for doing it.

Respondent: It's time to do it.

Interviewer: OK. I'll talk a bit now about the last bit of it which is the physical environment, the environment that you live, work and play in.

Respondent: Uh huh.

Interviewer: And when I talk about physical environment, I mean all the physical things around you, the roads, trees, houses, shops, traffic. And some of those things

are things that people living in urban areas have actually said might be important to being physically active. So I wanted to ask you whether they're relevant for you as well. So can you tell me a bit about how easy or difficult it is to be physically active in your area?

Respondent: I think it's very easy. I think the physical surrounds outside are very beautiful, we've got forests, we've got beaches, it's a pretty town. There's a bike path, well, you're not supposed to have bikes and skateboards on it, but there is a path that you can use for walking or running or whatever, and there's a beach. And I do see lots of other mothers from the school going for walks, going for runs, walking their dogs and so on. So it is there, easily accessible and there would actually be company to do it with if I wasn't on my way to work or wherever I'm off to. So I think I actually live in a place where it's quite easy to…

Interviewer: It's easy and then you were also describing that often you would need to get in to the car to get down to [town], that would have been the only...

Respondent: Yes. So that, so if for instance I wasn't then heading off to work, if I'd taken to the kids to school so I'm already in town with my car, I could then leave the car and go and do, if I wanted, along the foreshore or something and then drive back up home. It's too far to walk to school with the kids, 'cause you'd have to walk all the way down in to town, around and then back up another big hill. There's sort of like… and back over the highway again. There's no sort of direct ways, it's like a big U to get there. So if were in town, if we lived closer then we'd walk to and from school and that would involve a big hill everyday which would be really good. But it's actually just a bit too far.

Interviewer: Too far. OK. All right. And I just wanted to talk about a few features of the physical environment, and the first is availability and accessibility. So if I was to ask you about places to be active in your neighbourhood, your community, what sorts of things would you tell me about?

Respondent: OK. Just outside of town is a private pool, indoor pool, where, I don't know if they've doing it anymore 'cause it's been 12 months since I went, but they were doing aqua aerobics classes in the pool two evenings a week, which I used to go to until 12 months ago. And you could also book for private sessions, so you can go and you can go swimming and so forth, and I think they do yoga and some of those other sorts of things there as well. There's also a hall in town that I haven't been to where they do do yoga classes a couple of times a week. There is, I mentioned the walkway where you… walking or running or whatever, and there's a footpath all the way through [town] right to the far end to this coastal road that joins between [town] and [town] to where the houses run out, there's a footpath that whole way, so there is actually quite a long way you can walk on a footpath or the beach, and walking on the beach is a really good thing. There's a dog beach down this end, so a lot of people go down there with their dogs.

Interviewer: So when you're talking about this end, is that [town] end?

Respondent: [town] end, yes. There are people who horse ride, I don't, but that's… there's a pony club and there are horses and there are places where people go riding, it's available if you wanted to. I probably know more about what's available for children. There is surf live saving which involves both adults and children based… it's actually based at [x] Bay, I think that's where the actual… that first little beach as you go to [x] Bay, but some people are involved with that. I'm trying to think what else there is. I know football happens, down on the football oval but…

Interviewer: Is that far away or just…

Respondent: I know about Auskick but I don't know about the adults, but then I'm not a footballer as you might imagine.

Interviewer: That's fine. No, no, that's fine.

Respondent: So there must be, and I know they do dancing every week at [x] Creek which you can go to. So I think there's things… and there's all the stuff in [town] or [city]…

Interviewer: There's availability and there's access.

Respondent: There is.

Interviewer: Yep. And they're relatively easy to access in terms of their cost or opening hours?

Respondent: Yes, yes. My issue is I don't like going out in the evenings without… like once I'm home, I'd much rather stay home with my family then go out by myself to do Scottish dancing or whatever it is that you want to go and do, which I actually enjoy, but given the choice, I'd rather spend time with my family. So that's actually my own barrier to accessing things rather than a barrier itself, yes.

Interviewer: And does that change with the seasons? 'Cause I know in winter, once you're in you want to stay in.

Respondent: Yes. Even in the summer time I'd rather go out and walk with the family than go off somewhere by myself, yeah.

Interviewer: No, that's a really good point, no, thank you for that. And does having places to be active influence whether you're active or not?

Respondent: I think it does. When I was doing the aqua aerobics which was in the evenings, because it was very nearby and I'd get back before bedtime, I was happy to do it. If I had to travel further like to [city] to do the same thing and

miss the kids bedtime or just, you know, it added that extra travel time either way, I would say, no, that's too much time out of my evening. So I think having things available nearby does increase the likelihood that someone like me would access them and use them.

Interviewer: And if you wanted to be more active, are there things or places that would help if they were available or more accessible in your local community?

Respondent: The answer to that's probably, yes, but I don't know if I'd want it in my local community. I mean what I used to do a lot was gym was, you know, like aerobics, and I don't know and it, you know, it's a big gym, lots of classes so lots of flexibility with the times. You're not going to have that in a small community and I don't think I want it in my small community, but if it was right there I'd probably use it, but I don't think I want it there, you know what I mean?

Interviewer: Yep.

Respondent: And even to travel to [city], because it's compared to [city], the class times, they do have some that are after work. The morning ones are not good times unless you're not working obviously, so there's not really, well put it this way, the before work and the after work classes impacts so much if you've got young children, you just can't actually do them because otherwise you're looking at before school care, after school care, you know, if you've got a husband that works or whatever. So you just don't do them.

Interviewer: No, that's a really good point. Yes, and there's also that, what you were saying before, is that balance of tasks as well, of wanting to get home and be with the children.

Respondent: And for them to run ones in the middle of the day for people who work part time or shift work or whatever, you have to have a big enough population to

justify having classes and teachers and all the rest, for all those different time slots, which it's just not a big enough place to justify that, so…

Interviewer: Yep. No, that's a really good point. Now destinations, if I were to ask you about places that you could walk or cycle to from home, what sorts of things would you tell me about, and you already have?

Respondent: So [town] basically for walking or cycling. Cycling you could continue round to [x] Bay, [x] Creek. People do cycle to [town] along this road. I find that a bit scary, 'cause I know in a car and you come across a bike you don't…

Interviewer: It's narrow.

Respondent: Yeah, and overtaking them you can't see what's coming. So I probably wouldn't just because safety. So, yeah, so it'd be my local, but within that, you know, there's things you can do.

Interviewer: And does having destinations influence whether you're active or not? Having somewhere you can cycle to or walk to?

Respondent: Possibly. If you had someone to go that was nice to then, like a… yes, I know what you mean, like a natural sort of end point, and then you sit and have a little look or a cup of tea or something and then bring yourself back, it probably would if there was actually somewhere to go to, rather than just… sometimes you have these bike tracks don't you and they go on forever, and I know they're building one all the way through. But at what point do you stop and turn around? I don't know.

Interviewer: Yes, yes. OK. And if you wanted to be more active, would it help to have more destinations within easy, OK, walking?

Respondent: Well, in terms of now…

Interviewer: Within easy walking and riding distance.

Respondent: Oh, Ok.

Interviewer: Now that's the, mmm.

Respondent: Probably, yes, probably. Actually sometimes because we drive to destinations, we want to be active like [x] Mountain or whatever. Sometimes I think if there were more places like that a bit closer, we would probably go more often. But you tend to drive those sorts of places and do then all the activity when you're there.

Interviewer: How long would it take you?

Respondent: [x] Mountain is an hour and a half.

Interviewer: Yep, it's a sizeable time.

Respondent: Which is not terrible. But I was coming back from [town] last week and I, you know, you go past down near the [attraction] and I thought, oh, I should bring the kids up here. And then I thought, actually I don't know if they'd really want to sit in the car for that long, because, and I know it's probably no further than going to [x] Mountain, but [x] Mountain has a lot more to offer once you get there. So I thought, gee, it's a long way just to sit in the car, for all the reasons we don't like to use cars too much, to get there and then to go up to [attraction] and do your walk and come back down again, you spend more time in the car travelling to and fro then you actually would doing the activity even though it's a lovely spot. So if some of those sorts of spots were closer, we probably would access them more, you know what I mean? But there are lots of lovely spots nearby too, but, you know, if you want to branch out you've just got to get in the car basically.

Interviewer: Yes, yes. Now a bit about neighbourhood design.

Respondent: Uh huh.

Interviewer: If I was to ask you about the built or the manmade features, the non-natural bit of your area, what sorts of things would you tell me about?

Respondent: OK. There's no footpath, so we either walk on the grass or we walk on the road.

Interviewer: Do you have adequate enough lighting? Like if…

Respondent: We only do it in the daytime, we don't go walking at the night time, and that might be because we are… so we wouldn't have enough lighting up our driveway to get up to [x] Lane. Once you do, there are lights on the road, the actual lighting on the street itself is fine with the built up areas and then when it runs out and you hit the paddocks, which is where we tend to walk, then there's obviously no lighting. So we wouldn't walk at night.

Interviewer: So you tend to walk down the other end of the lane?

Respondent: Yes. It just goes… and there's bushland…

Interviewer: Yes, it's quieter.

Respondent: … you can go in to the dock and run around and just…

Interviewer: Just a little lane and what I gather that these sort of…

Respondent: Where we are, there's like another sort of two houses on each side and then it stops and then there's a farm over there and…

Interviewer: Is it bitumen or a dirt road?

Respondent: Bitumen.

Interviewer: Yep. And then it's a quiet road so you can walk on the road or just on the edges, it's OK?

Respondent: Yeah. The edges, like a lot of those sorts of road, the edges are gravel and they tend to be a bit sloped, so you lose your footing and they're not good for bikes.

Interviewer: Yeah, you sort of all off, yes.

Respondent: So you tend to be on the bitumen. And also there's electric fences so you don't want to get too close to the edge, not to mention the road kill that tends to accumulate by the sides. So you tend to be on the roads, so you are mindful of watching for cars, because if you do go up and down and it's steep and windy, but it's very, very pretty. We don't go too far down if we've got the children on bikes because they don't like coming back up the hills.

Interviewer: So you can walk that way and you can walk in to [town]…

Respondent: Uh huh.

Interviewer: …so that's sort of walkable, but it's fairly steep, and then once into [town], 'cause the other issue is streets being able to connect so you can actually go for walks that they go to somewhere rather than…

Respondent: Yes, yes.

Interviewer: … some places are just, you know, one main road and you can't get off it.

Respondent: Yeah, no actually it's not bad. [town] not bad actually in terms of connections. There are few sort of where you can't get through, but you basically need to come down and cross the railway line. Once you cross the railway line, then the connections are quite good. And there's connections across the railway line in a number of places as well, and there's sort of, like if you go around [x] Park and then it comes back around, and there's a few little places where you can do quite nice circuits in [town], it's quite pleasant really to walk around.

Interviewer: Good. So do the built features of your area influence whether you're psychically active or not?

Respondent: In my actual neighbourhood or down in [town]?

Interviewer: Both.

Respondent: Down in [town], yes?

Interviewer: Yes.

Respondent: The path is very nice, and they actually did a lot of work on that a couple of years ago and built it up and it's got some little seats and it's got those little history things which we really enjoy reading. And they did the skate park and it now winds around past the skate park and continues around dah, dah, dah. So it has joined up more parts of [town]. And the parks are very nice with the windmill and ducks and the sort of the bit of the creek that they've made in to sort of a lakey bit, that's got a path all the way around it so we always do the circuit around that and do different, you know what I mean? There are physical features that attract us to do certain things in the park. So, yes, I think the answer will be yes.

Interviewer: And if you wanted to be more active, are there any built features in your neighbourhood or broader area community that would help?

Respondent: That are there already or…

Interviewer: No, that aren't there or you'd like more of, what would help be more… help you be more active in your area?

Respondent: I don't know, it's probably there, it's probably just. I don't access it probably because of time.

Interviewer: Do you have a footpath from the main road down to Penguin from where you are?

Respondent: From the highway there is, there's a highway. From the highway there's a footpath all the way down. The inland part doesn't have a footpath, but that doesn't stop me from walking there with the dog and so on. It was trickier with the pram I have to say, because you're going over… you know what I mean, you're either on the road which I was really reluctant to be when they're very tiny, very protective.

Interviewer: Absolutely.

Respondent: So you were going over people's like lawns and gardens out the front. So it would be good to have footpaths up there.

Interviewer: And this is up to…

Respondent: So between the highway and [x] Lane, and then [x] Lane, so [x] Road continues, the houses continue as you head off towards…

Interviewer: Yes, I'm aware of where you're talking, yep.

Respondent: Yes. So up that section.

Interviewer: OK. Now the natural features of your environment, the aesthetics. If I were to ask you about the aesthetic qualities or the attractiveness of your area, what sorts of things would you tell me about? You've actually been telling me quite a bit already.

Respondent: Yes, yes. Look I really like it. There's lots of lovely trees, there's lots of gardens. You've got the ocean which is lovely, and buildings in [town] have actually been really well maintained. There's some old historic buildings and the churches, there's pretty churches, and they've redeveloped that foreshore area which is really nice now. And going along that main road, there's shops… they've got quite interesting things to look at in the windows and so on I really like, and [x] Park is very pretty, so, it is.

Interviewer: So it's [x] Park, yep?

Respondent: The one with the windmill?

Interviewer: Yep. And do you think your local environment is aesthetically pleasing and attractive near your home?

Respondent: Yes, yes.

Interviewer: Yep. And do the aesthetics of your neighbourhood or area, your broader community, influence whether you're physically active or not?

Respondent: Not in terms of my neighbourhood, that's just my neighbourhood.

Interviewer: But in terms of your community?

Respondent: Yes, I think in [town] the fact that it is quite pretty, it might not influence how often I access it, but it will…I will stay longer. I will stay longer because it's an attractive environment…

Interviewer: Yes, and you'll certainly enjoy it.

Respondent: … and we'll be down there any maybe have a coffee and I'll say, "Oh, look, let's go for a walk along the beach," because you're there and it's a nice thing to do, so, yes.

Interviewer: And if you wanted to be more active, are there features of your area that would help?

Respondent: Yes. Yes.

Interviewer: What's that?

Respondent: I think, again, so the physical, the aesthetics, the physical environment, the beauty, and having those paths down there. I could easily walk, maybe even run, bike ride, you know, so it's not that it's not available and would be enjoyable, I think again it's a time thing and I just might have to actually just be more disciplined about how I spend my time and just try and free up more time and keep it protected rather than letter other things wiggle their way in to those time slots and take over.

Interviewer: 'Cause there's always things to do aren't there?

Respondent: There are always things to do, and things do crop you, you know what I mean, and then other things have to get postponed, you've got to find time slots for them and it's just because it's not as high up the list as it perhaps should be, it's one of the ones that get sacrificed each time.

Interviewer: So the last area I wanted to just ask you about is safety in your neighbourhood. If I asked you about that, what sorts of things would you tell me about?

Respondent: Look in terms of people, very, very safe. In terms of… I think they've actually done a lot to improve things but I know that there were parts of the footpath that were broken and uneven, and certainly a friend of ours who is elderly but used to walk regularly had a nasty fall 'cause she tripped and so on. So maybe some physical things, barriers, particularly for maybe not so much myself but maybe older people or little ones tripping. But a lot of that has been addressed. I think the lighting is adequate in the central part of town. So we'll go down in to town to out for a meal and it's fine. You see the Police around a lot, so they're a very sort of physical presence there. Not in a threatening sort of a way, but you just know that they're around. But it's not a town that feels unsafe or anything like that, so that's not a barrier I don't think.

Interviewer: OK. So does personal… well, I was just going to ask you…

Respondent: Personal safety?

Interviewer: … does personal safety influence whether you're physically active or not, because it's a safe town so no.

Respondent: No. And there's a lot of people with dogs but you don't get those vicious dogs, you know, they're not barking, they're not growling and that sort of thing as you go past people's gardens. They're more very friendly, so…

Interviewer: And does road safety influence whether you're physically active or not?

Respondent: No.

Interviewer: Now you have mentioned about the bicycles, but there's places you wouldn't…

Respondent: Yes, and there's lots of people who do ride their bikes, it's just that I'm not a very confident bike rider, so I don't like roads. But the roads themselves, no. There's traffic lights at the main intersection which is well controlled, and obviously lights for the railway crossings at both places, just lights, no barriers, which I know have been issues in some other places, but to be honest, if the lights are working and the noise is working, then you should stop. So I think… and it's not… it's really just that main road that probably has a fairly high volume of traffic. But because it's very busy in there, because you've got all the shops and lots of people parking and crossing the road, people do actually travel very slowly through that area, so there's no difficulty crossing the roads and things like that.

Interviewer: So, just to check, if you wanted to be more active, are there any personal or road safety issues that if they were addressed might help you be more active?

Respondent: No, but in town I would probably ride the bike on the footpath which I'm not quite sure you're actually really allowed to do through that busy built up area with lots of, you know, where the cars for the shops and things are. Or I could take…

Interviewer: So if you had a little cycle path or…

Respondent: … I could take the alternative route. Yes. Now I think it is a shared footpath in one section, I'd have to check to the signs, but then in other sections it says you're not allowed to have your skateboard or your bike or whatever. So I'd have to check which sections you're allowed to ride on and which ones you're not.

Interviewer: Well, it is quite an issue for bike riders having sort of a safe area to ride. It's an ongoing issue, yep.

Respondent: Uh huh.

Interviewer: OK. Just to finish, is there anything else that you'd like to tell me today that we haven't covered about where you live or the environment or your physical activity, anything else we've missed?

Respondent: Probably, I mean it's actually very comprehensive. Probably not. I mean the bottom line is I'm just not very physically active and I should be, I used to be. I was very, very active, very fit before I got married. I could blame my husband couldn't I?

Interviewer: These are all times in our lives aren't they?

Respondent: Uh huh.

Interviewer: Yes. And what's, well, more interesting is…

Respondent: There's no doubt you have a lot more time when you're young and single for things…

Interviewer: Exactly, yes.

Respondent: … for yourself...

Interviewer: Yes, exactly.

Respondent: … before you have to share yourself.

Interviewer: When it's easy.

Respondent: Even sharing yourself with one other person, suddenly, you know, your diary's accommodating two, and by the time you add children in…

Interviewer: What's much more interesting is when it gets hard; you have to juggle things, yeah.

Respondent: And interestingly as I've, these last years, particularly as I've got older, I know that physically I am starting to age, you know what I mean? The joints you feel it, and I can feel I'm starting to get arthritis in some of my little joints, my mother's got very bad, and they play up and I think, mmm, this is not good. If I'm not careful I won't actually be able to do some of these things I want to do, but I don't know whether actually being physically active prevents that, or just helps you to live with it, I'm not sure. But as you get older, not only do you become time poor during that middle part of your life, but also your body is starting its deterioration.

Interviewer: Mmm, it's hard isn't it? You can't take it for granted in the same way that…

Respondent: No, and it takes long.

Interviewer: … young people often do.

Respondent: It takes longer to gain fitness, and it just seems faster to lose it as well, you know what I mean? Like we're not as plastic I suppose as we were when we were young. But the biggest thing is the time and that you are sharing yourself amongst all these other things, that when you were young, you had lots time. Didn't realise. So, no, that's probably about a pea in a nutshell really.

Interviewer: Thank you very much.

Respondent: That's OK.

Date: 11 October 2011

Duration: 37 minutes, 14 seconds

Interviewer: [interviewer]

Transcriber: [transcriber]

Now I thought I would start by talking about the place where you live. And, so if you could tell me a bit about where you live and what it is like to live where you live. Your likes and your dislikes.

Ah, well I live in [town] right next to the river.

Oh. Beautiful spot.

It is. Yes. Um, so there's plenty of park space, there's plenty of room for activity. The issue I think in [state] for us is weather. And – can you hear me?

Yep. I can hear you clearly. How are you? Can you hear me?

Yeah, not too bad. It's just that every so often I think you've gone. But perhaps you haven't. Yeah, so facilities are good and the area is good and it's a flat sort of an area. And there's lots of infrastructure that's set aside to do all sorts of sports. And there are strong sporting clubs in our area. I coach a number of um sports. And what I've noticed from a lot of people is that they will come from October or so, and then they have up to six months of the year where they don't do an activity because it's too cold, too wet, too dark.

Yeah.

And so six months of the year they are trying to get fit. And then the rest of the time they're unfit.

Oh that's a really interesting point. Yeah. And what we want to do, as we go through the questions, some of them will be around um you know seasonal difference. But it's good that you, because I'll be more focussing on what you are doing, so it's useful that you've given me the you know, any broader comments about the community. And indeed, as we go through, if there are particular comments you want to make which are broader, please feel free to do so.

Alright. Thank you.

OK. So ah, just a bit about where you live first. Um do you live on a house block, the usual standard block size, in [town]?

Ah yeah it's right on the river but it's about almost a hectare.

Oh wow. That's a nice size.

Yeah, so we bought a big block because we had four kids and we thought they'd need lots of room to run around. But they don't use it so it's just a lot of landscaping now.

But it's nice to be right on the river.

It is, yes.

And what's the length of time you've been living there?

Ah 17 years.

And your reasons for living there?

Um, in my job we get moved, we get moved around the state. But you can sort of work circuits. So you can base yourself in [city] and work all around [city]and never have to move. And in [city] you can do similar things and on the [area] you can do similar things. On the [area], [town] is quite central so I've managed to live there for all this time bringing my kids up and being able to transfer without moving my house.

Mm. That's excellent.

So that's the main reason.

That's the best of both worlds. It's always a bit hard when you've got work that you inevitably have to move on to different work place sites.

Yeah.

And so where you are in [town], how far are you from the actual town centre?

Oh it would be 2km.

OK. Now in research with men living in urban areas, and I was saying that most of the research has been done in urban areas, people often refer to their local neighbourhood. And I was interested in understanding what the term neighbourhood means for men living in rural areas. And indeed if it's relevant. So what does the term neighbourhood mean to you?

Mm. I'm not convinced it is relevant to me. I think my neighbourhood would come in terms of not the people that live near me but the people I interact with. I mentioned I coach, I'm in sporting clubs. And I would say that that is my neighbourhood.   So I live at home but then I go to these various locations and clubs.

Yep. So would the word community be more relevant to you?

Yeah. It would. Yeah.

OK. And so what would the, the word community would sort of bring in the clubs that you go to. What else would it mean to you?

Oh, it would mean sort of um, - can you still hear me?

Yes. I can hear you fine.

Oh, I think it's my phone.

Yeah.

I might put you on the loudspeaker.

OK.

So the community-

Oh you're a bit softer. [name] can I just stop you, you are actually a bit softer, so it might be a bit hard to pick up the recording on speaker.

Yeah. So in my job it's quite ah, there's some nasty things and there is some not good people. And so a bit of an outlet is to deal with good people that are sort of like minded. So I'm involved in swimming and athletics. And so dealing with a community that is more aimed at helping their own young people achieve things. And develop into good sort of citizens. Not that you would ever say that. But that is sort of what you are aiming it.

Yeah. You want to be positive about it, about community.

So I enjoy being with people that are like that, that actually want to do something positive. So you train with them, you compete with them, socialise with them, help them, they help you. So it is a genuine community. And I've grown up – well I've been grown up for a long time – but my kids have grown up with the same sort of people, doing the same things for a very long time now. And they've gone off to uni and keep those networks together. And sort of, I've grown with their parents as well.

Yes. It's a really good point. And with community, if you needed to put a distance around it, um how big a distance would it be? Just in kilometres. That you were talking about?

Well it's actually a very extended community because once you are in it for long enough – I'm involved at a state level and a national level, so the community gets quite large. But within a – and smaller, so…

So there's different notions of community.

Yes and –

So it could be the whole state. I could be national. And it could be local.

Yeah.

So when it's local, how big an area might we be talking about when it's local?

Well it just depends on where people live in [town]. So it could only be in a 10 kilometre radius.

Great. Now I want to talk about physical activity. And as I said it's a broad term, it's very much an individual thing. What does the term physical activity mean for you?

Well I just brought it down to movement.

Yep. And do you consider yourself physically active?

Yeah. I –

And why? Why would that be?

Ah, well I do have a sedentary job. So that's 8 hours for a day, and I try and move during that. But I get up at 5.30 am – this is now, I used to go swimming every morning – so I do a gym thing for an hour, six days a week. Then come to work and I do a pilates thing, group, three times a week. I do sprint training, well I coach but I also do the training, six times a week, swimming three times a week.

So you're very physically active.

Yes, well I've just learned that it's not a question of intensity, it's just doing things a lot. Because physically as you get older you need to do something, not just every day, but particularly with a job like mine where I just sit at a desk.

Yes. So you have to do it regularly enough. That's a really a good point. And it feeds into what you were saying about you know sometimes people sort of think it's a seasonal thing, and it's not, no.

It's not.

Where does physical activity fit in your life? Is it for instance no interest, a priority, or a high priority?

Ah it's one of the highest there is. I mean you need your body to live, and because I'm 48 now, I've seen lots of people die young. I've seen – my wife is an intensive care nurse and so she talks about that sort of thing a lot. I put on weight easily. And I can just see, you've just got to move to keep fit. And you feel better that way. You not only live longer, but your quality of life. I mean it is all pretty basic stuff, but your quality of life is just so much better.

No, but it's important.

You can be a very old 50 year old or you can be a young 80 year old.

Yeah, and I know which one I'd rather be. Yes. Absolutely. But no it is important because um, not everybody talks like that. They don't think about things like that. But um, I agree with you it is important in terms of being active, and whatever that may mean, you know, for different people it will mean different things. But as you said, it is important to do it regularly and then you do get a well-being effect from it too. Like a quality of life, yeah. No they are really important points. So, looking at it a bit more, and we will look at physical activity a bit more for you, um in our research when we think about physical activity, we think about any activity that lasts for at least ten minutes, that's the evidence of health benefit that you were talking about. It causes your body to work harder than normal and your heart rate to go up and etc. etc. So thinking about this type of physical activity during the past two weeks, um what I'll do is I'll go through different parts of your life, not to segment your life at all, but just to make sure we cover everything. So in the past two weeks have you been active at all at work for at least ten minutes at a time?

Active at work?

At work.

Um, yes. Doing my core group. Ah, well I just sort of run a core session, so it's about 40 minutes of a program [11:46]. So we do all sorts of leg exercises, push ups, core strength exercises, stretching, so we do that at work twice a week.

Yep, yep. OK. So you do it twice a week? At work?

Ah at work, yes.

Yep, that's what I mean. And the intensity of that exercise? Is it steady, moderate, or vigorous?

It's um, oh it depends on your fitness level, it's probably I'd say vigorous, yeah.

And um, OK, so when you do that is usually in the day time?

Yep.

And is this usually what you do? Or is it one off?

Ah no, we've been doing it for a year or so?

And does it change depending on the season or time of year?

No, it's indoors.

And if you wanted to, is there anything that would have helped you be more active at work? I mean you have taken actually, actually decided to do something about that already, but if there's anything else that you wanted to comment on.

Oh it's awkward isn't it? It depends on what job you are doing. My job is very sedentary, and even though I try to move as much as I can just doing my normal work, I can't get ten minutes of effort. All that I can do is walk every so often, get up. And that doesn't get much use. Better than nothing, but..

No. And that's a really important comment and people who we've been talking to in the study have actually been saying that, that that is a really common one. That's the nature of work these days. OK. In the past two weeks have you walked or cycled for at least ten minutes at a time to get to or from places?

Ah yes. Many times. Yep.

Yeah. OK. So can you tell me about what you did?

Um, well I mentioned that I put on weight easily. So, whilst I have running training, that's at various locations around [town], but I usually either walk or jog to the running. My running training really isn't a weight loss thing, it's sprinting, so I walk there to get – it's always 10-15 minutes to get there and back.

OK. So thinking about those activities of walking or jogging, um what would be the intensity of that? Steady, moderate or vigorous?

Oh that's only steady.

Yep. And the duration, sort of on average?

Oh about 15 minutes.

Yes. And how often would you have done that in the last couple of weeks?

Oh well it would happen every day, it's there and back. So it's 14 isn't it?

Yep. And um, when would you have done those activities? 15:01

Um, yeah I do it every day.

Was that before or after work?

I'm sorry, yeah that's after work.

Yep. And weekends?

Yep. Weekends.

Um, and where – OK, you've described where, you've done that, which is actually going to other activities. And were you alone or with others when you did those activities?

When I'm getting there?

Yeah.

Everyone else drives and I walk.

Oh, good on you.

They're all young.

Exactly. They don't think about it too much. Is this what you usually do or is it one off?

No, I do that all the time.

And does it change depending on the season or the time of year?

Ah, no.

Yep. And um, I need to ask this, but it sounds like you are really active anyway. If you had wanted to, is there anything that would have helped you use, you know, walking or jogging more often to and from places.

Ah, no. I guess sometimes there's time constraints. That's the only thing that stops me is if I'm late home from work and I might have to drive, I've got to meet a group of people, that sort of thing. I try not to let that happen.

Yep. Fair enough. In the past two weeks have you been active around the house or yard? So at home, inside or outside for at least ten minutes?

Ah, yeah I've got a gym thing that I do for an hour each morning.

And what would be the intensity of that? Steady, moderate or vigorous?

Um, vigorous probably.

And you do that every morning? Is that, how many days a week would that be?

Six.

And how long would you do it for? An hour did you say?

Um, almost an hour, 55 minutes.

Lovely. And would you do that by yourself or with others?

Ah, by myself.

And is this what you usually do or is it one off?

No, all the time?

And does it change depending on the season or time of year?

No.

OK. And if you wanted to is there anything that would help you be more active around home inside or outside?

Ah no, other than the time.

No, it sounds like you are pretty busy as it is. OK. So have you been physically active for at least ten minutes during your leisure or spare time? Now this is picking up all the other activities that we haven't covered yet.

Yes, I have.

Can you tell me about what you did?

Um, yeah after work six times a week we go sprinting. So twice a week it's sprinting up a hill. And the other times it's various locations; at the beach one day, showgrounds just running on grass the other days.   And after that I have swimming which is done at the pool in [town].

So thinking about those activities, could you describe to me the intensity of them? Whether they're steady, moderate or vigorous?

Oh they're vigorous.

Yeah. And how long would you do those activities at a time?

Ah, the sprinting is 90 minutes each time. And the swimming is about an hour.

And the frequency. How often would you do them?

The sprinting six times a week and the swimming is three.

Yep. And um, that's usually after work?

Yeah, after work.

And on Saturdays, yep. And um, do you do that locally?

Yeah, all that training is local yeah, in [town].

And swimming?

Ah, at the [town] pool, yes.

Yep. OK. And would you do that by yourself or with others?

Ah, that's all with others.

OK. And is what you usually do or is it one off?

Ah it's all the time?

And does it change depending on the season or time of year.

No, the running doesn't. The swimming changes locations. In the summer we go to [city]. Because the pool in [town] closes.

Oh over the summer?

Yeah. It's owned by the [organisation] you see, and that goes by school terms.

Oh OK. Right. Because there's been a couple of people I've talked with who said it would be really nice if um, there was more you know, more a public pool that was open all year round in [town]. From that point of view, yeah, OK. And um, I'm meant to ask the question, but it sounds like you are pretty active as it is. If you wanted to is there anything that would have helped you be more active in your leisure or spare time?

No, again it's just the time.

OK. The last part of the study is the physical environment. And we've talked about where you live and physical activities. And now I want to talk about the physical environments where you live and work and play. And when I talk about physical environment, I mean all the physical things that surround you like the roads, the trees, houses, parks, traffic. And some of these things are things people living in urban areas have said might be important for their physical activity. So I wanted to ask whether they were relevant for you. Now, first of all can you tell me a bit about how easy or difficult it is to be physically active in your area?

Yes. It's very easy. There's heaps of paths and parks and grassed areas. Great long beach. As I said the only issue when it's dark. But you can still do things in the dark. People just choose not to. It's often cold and that's hard too.

Yeah. Sometimes it's a bit hard to see what you're doing too.

Yes.

Though generally people are starting to say [town] is a fairly safe community, so it's not necessarily about safety. It's more about being able to see where you're going.

Yeah. Well I've never had any concerns with safety issues. Yeah, the only danger is you might hurt your foot sometimes if you tripped on something, but I've never had any trouble all the years I've been doing that. You can find excuses, but you can do it if you want to.

Yeah. OK. I'll just talk about a few different areas now. Availability and accessibility of places to be active. Now if I were to ask you about places to be active in your area, what sorts of things would you tell me about? And you've actually told me about a lot of the sporting clubs. And this is a broader question. It may not be the things that you use, but it's you know, more broadly what's available in your community.

Yeah, in my view there's no excuse for not being active, other than just laziness. And there's the river that we kayak on and you can swim in it, a basketball court, there's tennis courts, squash courts, there's an athletic track at [town], there's the swimming pool, a softball club, there's just oh, no limits.

Yeah, you've got really good availability up there haven't you?

Yeah and it's all close and it's all good, and the clubs are well established. The only issues are just within people, they think I'll do that later. Or it's sometimes not always easy to tap in. Because it's all run by volunteers of course. It's often not easy to find how you join something. And I spent a couple of years trying to get into athletics but just couldn't work out how to do it. There's a track there, but there is just never anyone there. And there's a phone number there that no one would ever answer.

Oh that's a really good point.

It is actually a big point. And the surf club I've seen people come that want to join and they sort of hang around. And unless someone latches on to them and holds on, they sort of drift off again. Getting started is a huge problem for people. It's just a human condition. Nothing else really for it. And sometimes clubs just need to work out how to do that better.

Yeah, cause it's as you say, make the point that you know, like in terms of people staying at it, it is sometimes a lot easier when they can actually find information out more easily.

Yeah, so you walk into a place and everybody knows everybody else and they are all chatting and knowing what to do. And you are just standing there. And a lot of people will just get up. And then there is the expense of it. So do you pay for membership straight up, or do you wait two or three times? Those sorts of issues are there. But mainly I think it's just a compound psychological thing of before exercise, everybody gets a bit nervous. And decide not to do it, or they'll do it tomorrow instead. So you've got to defeat that. And ease of becoming involved, not worrying about the fact that you are not as good as the best person yet. And then in [state], I really do think weather is just the hugest thing. Whether you live in a city or a town, people just hibernate in winter. I've had people who don't turn up to my running sessions because it's raining and they think, well no one will come. But you can do it. And if in [state] you don't exercise in the winter, then you are just not going to be fit and healthy.

No, it's no use just coming out when the sun is out.

Yeah, but people do. Yes, so I think that's pretty much what I've worked out on all this. Oh work is the other thing. All the shift work that people do affects team sports. I mean gyms are opening up everywhere, so people get memberships of gyms. But again if you just go by yourself, don't know what you are doing, people pay for gym memberships and don't do anything. And it's not an actual sport itself. So a lot of the improvements or support or

motivations that you get from playing sport you don't get in gyms. It also gives you a false belief of your fitness. So I think gyms are actually not really a good thing. Because if you don't have to commit to a team or a group, you can put it off. You just do it tomorrow. But if you have got to go and train because your team needs to train or you got beat that week, then you do. But anyway, sort of shift work and Saturday morning trade and that sort of thing is what's caused an awful lot of the problems, I think.

Thank you for those comments. They're really useful. People have actually sort of said with the study areas, they may not be doing gym work but they say, you know, an activity they do actually helps doing with others.

Yes. Well that's been my experience. You can do so much on your own for so long. But eventually you need motivation to do it. And it's very difficult to do it on your own. And you adapt and lift levels when you are doing it with other people. So that's I mean, I guess that's how organised sport started in the first place. The community that are doing it. And now it's become so much more individualised, with gyms and just going for a walk by yourself. Yeah, it's just easier to put it off. That's what happens. Or you do it for a year or so and then you get sick of it.

Yeah. The other side of availability is access. Talking about, thinking about those, the places you've been talking about for being active, are they accessible. Are they easy to access in terms of their cost and opening hours. Now we've talked about one point about that the information may not be quite as easily accessible. But, um, with cost and opening hours, other things?

I guess that that is a possible barrier, but it's not – I mean as I said most things are run by volunteers, most of the sporting things and it fits around their work. So things aren't available always the minute you want them to be. But again that's just life and you can work ways around it. And I don't really see that you can do much about that. In [town] the pool closing, well that's a bit of an issue, but if you really want to swim it's probably a 20 minute drive to [city]. And we've done that for years in summer. It's not really much, but some people would just never consider that, driving to [city] to go, from [town].

Mm. So does having places to be active influence whether you are active or not?

I don't really think so. I think that if you want to you can.

Yeah. Yeah. And if you wanted to be more active are there things or places that would help if they were available or more accessible?

Yes. Ah, yeah you've got to have the facilities. I mean [town] has built that walking track from [town] to [town] and now they're talking about continuing that to [town]. *And that's proved very popular already I believe.*

Oh it has. And you see lots of people out walking or riding on it. And anything new like that does seem to get people going for a while. But again I don't know that it keeps them going.

Yeah, well that is a very good point. Yes, yes. OK.

I really do think organised activities are what will sustain people's activity in the long term. Because on our own, I mean things like the [city] 10 are good because people will train for those. So those community – and triathalons that some people do, or duathalons – all those sorts of new sorts of things are good for getting people involved and training for them.

Yep. And destinations, if I were to ask you about places you could walk or cycle from your home, what sorts of things would you tell me about? Now, you've already told me that you actually walk or jog to sporting clubs anyway. And so, we've sort of covered that. But it's also covering things like shops or schools, workplaces. Now I presume your workplace, you, like you were saying that you move around, so you may not be able to walk there. But it's more in the car. Are there any other destinations?

Yeah, I got to drive to work, because it's the time thing. I used to ride a bike to work. But it's too far. But um, yes I don't think there is anywhere in [town] that you need a car for. You could walk anywhere. There's people who do sort of, there's people who walk everywhere. And there's enough shops everywhere else, the schools are close, it's relatively flat. We could all abandon our cars and just walk everywhere if we were of a mind to.

That's an interesting thought isn't it? And does having destinations influence whether you are active or not?

Ah, no, not me it doesn't no.

And if you wanted to be more active, - I've got to ask this question, cos you already are really active – would it help if there were more destinations within easy walking or riding distance?

Ah, no, I don't –

No, because you are already saying that they are within, they're accessible. Now, another area is neighbourhood function and design. If I were to ask you about the built or man-made features of your area, what sorts of things would you tell me about?

Um, there are many parks, and there's footpaths and there's good street lighting. So parks with play equipment for young kids are quite good. But there's also parks that are big

enough and wide enough and sparsely populated enough for people playing any game they like on them. And the beach is nearby – oh, that's not man-made of course.

And do the built features of your area influence whether you are physically active or not?

No, no. It just depends on where I go.

And if you wanted to be more active are there any built features in your area that would help?

Ah, no, not really.

It's fairly well organised, [town], isn't it?

I think it is. Yeah. Must just be a good size and have enough people interested enough to do enough for a long –

Now, the other side of it is the aesthetics, the natural environment. If I were to ask you about the aesthetic qualities or the attractiveness of your area, what sorts of things would you tell me about?

Well it's sea side, and with the river and it's surrounded by the hinterland has got mountains. Ah, so aesthetically it's quite good to be outside in it. And the hinterland does give a lot of opportunity if you like for that sort of thing – which I don't but – but there's plenty of opportunity if you want to.

For other activities, yep. So do the aesthetics of your area influence whether you are physically active or not?

Ah, no it just makes it more pleasant while you are doing it. But no, it wouldn't make any difference, I don't think.

Um, and if you wanted to be more active, are there any features in your area that might help if they were more aesthetically pleasing or attractive?

Ah, no. Sorry.

Fine. No, the last area is on safety. If I was to ask you about safety in the area, what sorts of things would you tell me about?

Um, well it's well policed. There are footpaths in good repair. The roads are in good repair. The parks that are running are good and safe and kept in good repair. There's street lighting. It's entirely safe.

And does personal safety influence whether you are physically active or not?

No, not at all. No.

And does road safety influence whether you are physically active or not?

Um, no, not really. As I said I coach other people. And sometimes I do worry about the roads with people crossing them. And if it's wet with some drivers losing control and things. But I guess that is going to happen anywhere.

It's true. There are some things that are going to happen. It doesn't matter where you are. And if you wanted to be more active are there any personal or road safety issues that if addressed might help you to be more active?

No.

Great. Thank you. That's the end of it. Before we finish though, is there anything else that you'd like to tell me about today about where you live or physical activity or the environment that we haven't covered today?

Um, no I can't really think of anything. It's all about attitude. And I agree, the survey might be to say well we need to put this infrastructure in, all those sorts of things, but I guess extra to that someone needs to be at least as much worked out on making people understand that their attitude of doing it and how much they need to do, and should do – but I guess that's rather – you are just looking at making sure or seeing what is available. But I would argue that you can find places to exercise and do things wherever you are, and buildings are not really necessary. There needs to be a lot of work done on attitudes and we didn't even talk about diet.

Mm. That's another one in itself too. Mm. And hopefully, no thank you very much for that.

AUDIO RECORDING ENDS
