# Supplementary material for: A Qualitative Study of Environmental Factors Important for Physical Activity in Rural Adults
Source: PLoS One. 2015 Nov 10;10(11):e0140659. doi: 10.1371/journal.pone.0140659 (PMC4640845; doi:10.1371/journal.pone.0140659)
Supplement: S2 File — (PDF) [file pone.0140659.s002.pdf]

Interviewer: OK so I thought we might start by talking a bit about the place where you live, so can you tell me a bit about what it's like to live where you live and maybe your likes and dislikes of it?

Respondent: OK, well we work from home so we spend a lot of time at home and our home is situated halfway between [town] and [town] on the coast.

Interviewer: Oh, yeah.

Respondent: So it's beautiful, it's on the [x] Island so from a…

Interviewer: I know the spot.

Respondent: Do you?

Interviewer: Yes I had a few moments yesterday so I thought OK we're working with [town] and [town], I'll have a look [laughs].

Respondent: You would have gone past.

Interviewer: It's a beautiful spot.

Respondent: Yeah.

Interviewer: And someone had mentioned the islands off there.

Respondent: Yeah, it's absolutely amazing.

Interviewer: So that's where you are?

Respondent: That's where we are, so whilst we've got nature surrounding us we've got very limited facilities, well you know I mean it's not to say we have a footpath we can go an exercise or a pathway we can…

Interviewer: Are you close to the main road there because it's quite tight that coast there.

Respondent: Right between the main road, us and the sea so just on that little point, there's a little point there.

Interviewer: So your seaside or…?

Respondent: Seaside.

Interviewer: Oh, lucky.

Respondent: I know, very lucky, very, very lucky yes.

Interviewer: [Laughs] Wow.

Respondent: Yes.

Interviewer: Because there's not many spots there.

Respondent: No when I bought it it was just apparently ten minutes between me and the other person getting it, securing it with the real estate agent so I was very, very happy with it. So whilst we're lucky where we live, you know nature is all around us; having access on your doorstep to infrastructure for exercise probably is just a little bit of an impediment. It means that if we want to go for a walk we can't really walk on the beach around us because it's too rocky.

Interviewer: Yes.

Respondent: We have to get in the car…

Interviewer: Yes.

Respondent: … and drive, even if it's only two minutes down the road where you can walk on [x] Beach or drive into [town], go to the gym or park close to [town] and walk on the pathways.

Interviewer: OK.

Respondent: We can ride our bike but it's too dangerous on that windy…

Interviewer: It's very narrow isn't it?

Respondent: It's very narrow around there for riding on the road, but then again the other advantage is nature around us, we can get in the car and go for lots of bush walks and things.

Interviewer: So are you on a regular house and block there?

Respondent: Yeah, yeah.

Interviewer: Yep, OK. And what's the length of time you've lived there?

Respondent: I've lived there for seven years now and [partner] has been there with me for about four.

Interviewer: And what's your reasons for living there?

Respondent: Well I had a big lifestyle change about seven years ago. I'd been spending a lot of time living ten minutes away farming and always wanted to have a quiet, sort of a quiet lifestyle near the sea. That was always my vision so chose to live there rather than in town.

Interviewer: And is there any reason why you particularly picked that area?

Respondent: Just because it's absolutely stunning [laughs].

Interviewer: [Laughs] Fair enough.

Respondent: It's a nature reserve.

Interviewer: Yeah.

Respondent: So the seagulls breed on the islands. The bird life is amazing, sea eagles fly by and all the peregrine falcons and it's just amazing, it's beautiful, it's the best place in [state] I reckon [laughs].

Interviewer: Lucky [laughs].

Respondent: Very lucky.

Interviewer: And so how far are you from the nearest town? How far are you from [town]?

Respondent: Six k's each way.

Interviewer: Oh OK from between [town] and [town], OK.

Respondent: Yeah.

Interviewer: OK, now in our research with women often we've been looking at, most of the research has been done in urban areas of course, and people often refer to the their local neighbourhood and I'm interested in understanding what the term neighbourhood might mean to women who live in rural areas. So does the word neighbourhood, what does it mean to you, does it mean anything to you?

Respondent: Oh it does, I mean where we are we joke and say it's called loner village even though it's not [laughs].

Interviewer: [Laughs].

Respondent: So our neighbours who are close by, there's just a little cluster that's there, we all know each other well and rely on each for simple things like if we're away to put the rubbish out or pick up a newspaper or help if you know you've left the tap on and you've gone away for a few days, in the garden, and things like that so, and we know them all personally so it's probably the most connected neighbourhood I've lived in. And in comparing to living in a farming area only ten minutes away, whilst you know your neighbours it's not the same sort of thing because you're separated by distance, so you don't necessarily have that closeness of casual interruption or casual meeting that you might see someone across the fence while you're our doing something.

Interviewer: You're closer yeah.

Respondent: Yeah.

Interviewer: So neighbours are quite close in that way?

Respondent: Yeah.

Interviewer: Sort of maybe not suburban close but…?

Respondent: Probably in between suburban and, what do they call that in-between style, rural residential, you know rural residential blocks are usually about a couple of acres or something, probably in between those two scales.

Interviewer: Yep, and if you were to put a distance on that how big a distance would that be?

Respondent: Between houses?

Interviewer: No in the neighbourhood itself.

Respondent: Oh the little group in the neighbourhood? How big would it be?

Interviewer: Yeah, would it be a kilometre or…?

Respondent: Oh no.

Interviewer: Less than that?

Respondent: It's probably, I'd say within, it's whole diameter would be probably about 800, 700 to 800 metres.

Interviewer: OK.

Respondent: So it's not massive.

Interviewer: No, OK. And what would the, about the word community, is that relevant to you?

Respondent: Oh yes [laughs].

Interviewer: [Laughs].

Respondent: Yes it is. I mean I've worked in community development for 20 years or more so I mean obviously I have a work view about what that is.

Interviewer: And where you live, do you have a sense of what this community means to you?

Respondent: Probably where I live is more just a sense of that local neighbourhood and those individual people.

Interviewer: Yep, yep.

Respondent: Rather than a holistic sense that we all belong to something bigger or…

Interviewer: Yeah, so there's a difference between neighbourhood and community?

Respondent: I think there is yeah, definitely.

Interviewer: So how would, what term would more aptly describe for you where you live?

Respondent: Neighbourhood.

Interviewer: Neighbourhood?

Respondent: Yeah definitely.

Interviewer: OK. Now I want to talk a bit about physical activity and as I was saying it's very broad and everybody has their own idea about what that means to them, and we're interested in finding out what that means. So what does the term physical activity mean to you?

Respondent: Well physical activity for me has changed over the years and more recently getting older and understanding how your body changes when you hit about 40 as a woman. You need, well I found for myself you need to actually make more effort, so physical, probably in the past I would have said walking and gardening would have counted for physical activity. So now I'm actually structuring my physical activity and making it more intense and more about specific things to do with maintaining a certain heart rate, working on building certain muscles in the body and coupling it with diet, cutting things out, putting things in the diet. So it's more, for me physical activity is a more structured thing that I make more effort and more time to actually do now.

Interviewer: And focussing on sort of good health for you?

Respondent: Yeah, absolutely. Some weight outcomes, some endurance outcomes to do with bushwalking and being able to carry a pack(?) better and carry more weight, those sorts of things. And also I've got an [x] year old too, it's now more, and he's a [gender], and being able to keep up with him, rough and tumble and building cubbies and doing all those physical things with him, so that's just an added benefit along the way.

Interviewer: You've found that extra strength of the focus that you have are your exercises also [laughs].

Respondent: Yeah [laughs].

Interviewer: Play with him. [

Respondent: Absolutely.

Interviewer: OK. And where does physical activity fit into your life?

Respondent: Well it fits in on a daily basis. So every day I will have to make a choice as to how I'm going to schedule it in.

Interviewer: OK.

Respondent: So it's along with deciding how many hours, because working from home of course…

Interviewer: Uh-huh.

Respondent: … you've got to put boundaries around that.

Interviewer: Uh-huh.

Respondent: Either really, really slack or really, really dominating [laughs]. So it's along the lines of [partner] I would say OK what are we going to do today, we'll work for a couple of hours and we might go to the gym for an hour, or we might leave off the gym today because we've only just started the gym last week because our muscles are sore so we'll go for a walk on the beach today. Or it might be pouring with rain or foul and we've gone well we're going to have to settle for the exercise bike [laughs] today.

Interviewer: Yes [laugh]. So you have to really sort of have a plan of what you're wanting to do?

Respondent: Yeah.

Interviewer: So would you call it a high priority or a priority or something you don't have any interest in?

Respondent: At the moment it's a high priority for us because we've noticed that we've both put on weight. And I don't know whether it's a physiological thing as a woman as you get older, all of a sudden you think oh dear [laughs], how did I get here? I weigh ten kilograms more than I did you know 20 years ago, so at the moment it's a high priority for us.

Interviewer: Yeah, OK. Now we're going to talk a bit more about physical activity and the easiest way to do that is in different parts of your life but you may not see, you know, your life in parts, but it's just easier to make sure that we cover everything.

So in our research when we think about physical activity we think about any activity that lasts for at least ten minutes, so that's the evidence of the health benefit. And it causes your body to work harder, maybe your heart rate comes up, huffing and puffing a bit more, feeling warmer. So thinking about this type of physical activity over the last two weeks, have you been active in your work for at least ten minutes of the time, bearing in mind you work at home as well as I presume out and about like your down to [city] for work as well?

Respondent: It's sort of a little bit hard for us because sometimes we consider going out and taking photos as part of our work and when we do that we're always in natural settings.

Interviewer: OK.

Respondent: So that means a pack on or even the camera bag on, so yes we would.

Interviewer: Yes.

Respondent: But if we were to be very purist about it and we work at our computer, no.

Interviewer: No, so how often would you, perhaps in the last two weeks, have gone out and you know, done the activity of taking photographs?

Respondent: Yeah well we did on Saturday so we went to [x] Falls, and that was a good walk on Saturday. Today walking into town and back we would have done, well it's 40 minutes each way to our accommodation today so we'll do that and did that yesterday, we did it the day before. Sunday would have been the only day when we didn't work although packing the car [laughs].

Interviewer: Yeah, yeah, travelling is that counted as [laughs]?

Respondent: Bit of huffing and puffing there, maybe not for ten minutes [laughs].

Interviewer: Yeah [laughs].

Respondent: And then last week work-wise probably not. We had our structured exercise separate from our work last week.

Interviewer: So would you describe the intensity of the exercise as, if you had to describe it what would it be, vigorous or moderate or steady?

Respondent: Separate from our structured exercise are you talking about?

Interviewer: Yeah the…

Respondent: So with work?

Interviewer: Yep the activities you've already talked about, like taking the photographs and walking backwards and forwards to meetings here and stuff like that.

Respondent: Yeah I'd say probably vigorous probably, moderate to vigorous.

Interviewer: OK.

Respondent: Yeah, because obviously walking, walking down here and things yes we're getting hot and...

Interviewer: Yeah you do, [laughs].

Respondent: [Laughs].

Interviewer: I always walk to work and you always end up hot, let alone going back home

Respondent: [13:29].

Interviewer: Yeah it keeps on, everyone stops as you start going up the hill [laughs].

Respondent: [Laughs].

Interviewer: Good one. So how long would you have done that exercise for?

Respondent: As in how…?

Interviewer: The time taken to do it, so it's obviously over ten minutes.

Respondent: Yeah.

Interviewer: So when you walked in how, you said that was 40?

Respondent: Yeah, 40 one way so two lots of forty so that's what, 80…

Interviewer: Yeah.

Respondent: … so 80 today, yesterday we did 80 plus we actually rode out to [attraction] and back on our bikes.

Interviewer: Really?

Respondent: Yeah, and it was [laughs] great. [attraction](?) was just fabulous.

Interviewer: Yeah.

Respondent: So that took another 80 so two lots of 80 yesterday.

Interviewer: Uh-huh.

Respondent: Day before would have been 80. Probably ten minutes on Sunday, actually I mowed the lawn on Sunday.

Interviewer: Uh-huh.

Respondent: So that's an hour and a half…

Interviewer: We'll get to that one.

Respondent: … on the lawnmower.

Interviewer: Because we'll get to the house, around the house later.

Respondent: And Saturday we had the walk which was…

Interviewer: Yep.

Respondent: … that was three hours.

Interviewer: Oh right, OK yep. And how often would you do that? Those activities?

Respondent: Look when the weather's good we tend to be, that's probably slightly more active than we usually be, probably twice the normal amount.

Interviewer: Uh-huh. But because you've come down here…

Respondent: Yeah. Weather is the main thing up there that stops us…

Interviewer: Yeah.

Respondent: … from being able to get out and about.

Interviewer: I was going to ask you, so how often would you normally do that then, once or twice a week, for work?

Respondent: For work?

Interviewer: Yeah.

Respondent: Probably, we'd like, once would be minimum.

Interviewer: OK once a week.

Respondent: You see, so what it is, because [son] is, now that he's older he's shared, one week with me one week with his Dad. So what we tend to do, sorry this sounds complicated but this…

Interviewer: No, no.

Respondent: … this is the pattern of how it goes. If he's with us, for the week he's with us, because obviously he goes to school and things like that, we tend to be around home more so our exercise would be more walking on the beach with him after school or me doing things with him, head down to the beach after school. On the week that we don't have him, if the weather's good, especially over summer months we take the camper and we go walking and taking photos.

Interviewer: Yep.

Respondent: And things like that. But say that off week the weather's bad and we think blow it we'll have to do a lot of work this week so we can have the time off.

Interviewer: So it does depend on the weather and the season and…?

Respondent: Yeah.

Interviewer: So less so in winter?

Respondent: Less so in winter and more so in summer.

Interviewer: OK, and if you wanted to is there anything that could have helped you be more active at work?

Respondent: Not having to do work [laughs].

Interviewer: [Laughs] Not have to work at the computer.

Respondent: Not have to work from here I think [laughs] because somehow I [16:43] something where I think oh get an iPad and sit like that on the, I don't know, yeah I don't think we've got many barriers there to be honest.

Interviewer: No, so…

Respondent: We're pretty lucky.

Interviewer: Yeah, and you've actually sort of really thought about it too.

Respondent: Uh-huh.

Interviewer: OK, so another part of life, in the past two weeks have you walked or cycled for at least ten minutes at a time to get to or from places?

Respondent: Yes.

Interviewer: We've already mentioned that [laughs].

Respondent: It was only yesterday yeah, which is good.

Interviewer: Uh-huh.

Respondent: So that was 40 minutes from when we get the bike out to [attraction] and then 40 minutes, and that was pretty hard going on those bikes.

Interviewer: Uh-huh.

Respondent: Because the gearing's all different.

Interviewer: Did you take the, can you get out far the cycle way?

Respondent: Yeah you can, it's awesome.

Interviewer: Oh great.

Respondent: It's absolutely amazing, what a terrific asset that is.

Interviewer: I meant to get out there but…

Respondent: We were just raving about that.

Interviewer: Uh-huh.

Respondent: So lucky to have that there. Just think all those houses and I mean I saw a whole new part of [city], because I lived down here when I went to Uni way back, and I saw a whole new part of [city] I've never seen and we were just saying all those houses along that cycle pathway, what a great thing for anyone who had to walk in town.

Interviewer: Yeah.

Respondent: You know, hop on that and wiz in.

Interviewer: It's great. So you were able to access that from where you were staying, you could…?

Respondent: Yep well we walked; we always walk from where we're staying into town.

Interviewer: Yeah.

Respondent: So we came down to [x] Wharf.

Interviewer: Oh yeah.

Respondent: And they hired them at that…

Interviewer: Oh OK.

Respondent: They've got this great thing where you can just pay $15 for four hours for the bikes and if you decide when you ride out there that you don't, can't handle riding back you can catch the ferry back at no extra cost.

Interviewer: They have done it well.

Respondent: Yeah, and we thought $15 for the bike, the quality of the bike and the helmet for each bike for about four hours is really good value.

Interviewer: Uh-huh, so they really have sort of thought about, yeah, making those offers for people.

Respondent: Uh-huh.

Interviewer: OK so that's cycled and walked to and from places, sounds like you have on the beach and things like that?

Respondent: Yes.

Interviewer: OK, so the intensity of that exercise, how would you describe it?

Respondent: Oh well we try to push it.

Interviewer: Yeah so…

Respondent: There's no point in being sedentary.

Interviewer: Yeah, vigorous so…?

Respondent: Yeah I mean if [son] comes with us I usually joke with him and he and I will try and sprint to the next stormwater drain or something like that until I've pulled something or [laughs] decided to, not fit enough to be doing that sort of thing.

Interviewer: [Laughs] Fair enough. And the duration, how long would you have done that, the cycling and the walking for?

Respondent: As a pattern you mean?

Interviewer: Uh-huh, in the last two weeks.

Respondent: Oh in the last two weeks.

Interviewer: To get to or from places.

Respondent: Oh well once with the cycling in the last two weeks and the walking, well we walked every day since Monday.

Interviewer: Uh-huh.

Respondent: Walked on Saturday. We would have had one other day walking the week before on one of the off days we didn't go to the gym so one, two, three, I don't know at least seven.

Interviewer: Uh-huh, that's fine, and did you do it at any particular time of the day?

Respondent: Yes we have changed, well if [son] with us it will tend to be after school.

Interviewer: Yep.

Respondent: If we haven't done it in the morning. So it just depends. We're finding with our work at home, in the morning we'll do, get into the, see what emails have come in or anything to do with work in the morning. If we choose not to go early in the afternoon before he comes home then we will do it when he comes home. If he's not with us we'd probably do just the first little bit in the morning and try to still make it before lunch.

Interviewer: OK. And…

Respondent: If that makes sense.

Interviewer: It does make sense. And for your walking and cycling why did you do it there? What attracted you to the place?

Respondent: The pathway.

Interviewer: Yeah.

Respondent: That was you know, we wouldn't have attempted it otherwise if we had to negotiate on the road.

Interviewer: No absolutely.

Respondent: Too spooky with the cars here in town.

Interviewer: Yep. OK, does this change depending on the season or the time of the year?

Respondent: Well it was funny because we got on the bikes yesterday and we thought oh hell that's cold [laughs].

Interviewer: It was a little crisp yesterday [laughs].

Respondent: Freezing, and initially I thought oh what have we done here but by the time we'd warmed up and got out there we thought no its OK we'll ride back home so…

Interviewer: I was in [town] and it was I think three point five but I got told at home in -[suburb] it was two point five [laughs].

Respondent: Yeah.

Interviewer: There was solid(?) [laughs].

Respondent: Definitely colder there yeah. It's probably rain which would be the main thing to stop us doing that.

Interviewer: Uh-huh, yeah, and you were saying that you wouldn't cycle at home because of the road.

Respondent: Yeah look we do sometimes but rarely.

Interviewer: Yep.

Respondent: We're more likely to take our mountain bikes…

Interviewer: Somewhere else.

Respondent: … out on the bush track somewhere yeah.

Interviewer: Yep, fair enough. And if you'd wanted to is there anything that would have helped you to use these forms of transport more often?

Respondent: Oh look if we had a cycle way like you've got here going past our doorstep we'd be on it all the time, [son] would be on it all the time, it would be absolutely marvellous.

Interviewer: Uh-huh I've noticed lots of kids here that cycle yeah.

Respondent: So yeah they really do need that kind of public infrastructure up there.

Interviewer: Uh-huh. So so far when I've talked to people like that they've talked bout the, there must be a shared walkway, cycle track to [x] Beach…

Respondent: Yeah.

Interviewer: … but that's from [town] so that's the other way.

Respondent: It is yeah.

Interviewer: But there's nothing…

Respondent: No.

Interviewer: … your way?

Respondent: No, in fact I've just finished the consultancy for the whole pathway.

Interviewer: Oh, I'm fascinated to find out what's happening [laughs].

Respondent: Yeah.

Interviewer: It'd be interesting.

Respondent: Yeah that's been really, so it's a personal interest of mine too so that'll be a great project, so the cost benefit to the community is massive up there for that to happen.

Interviewer: Absolutely, particularly that I was over in [town] because of the community centre that's there and I noticed how accessible you know like to the coast that area is as well as people have told me obviously the other side is. I was thinking oh it's very accessible.

Respondent: Oh it is, it's not hard. It's not hard, I mean it only needs 27 million and that would get it completely from [town] all the way to Port [x] which is 70 kilometres. I mean it's a, you know it's an ambitious project but I suggested that what they ought to do is be concentrating on a three kilometre radius and a five kilometre radius of the populated areas because that gives enough infrastructure for at least 30 minutes of exercise, whether its walking one way or cycling both ways quickly. Everyone could achieve that.

Interviewer: Uh-huh, that's interesting, because one of the things that's coming out in the study of course too which you would obviously have been talking to people about is that you know you've got the more township areas, the bigger centres that are starting to, you know people are talking about the accessibility of tracks in [town]. But then you've got people who are sort of, as you are, slightly out, the next round out that just want some linking, wants to…

Respondent: Yeah.

Interviewer: Yeah. OK, so, in the past two weeks have you been active around the house or yard, either inside or outside?

Respondent: Yes.

Interviewer: For at least ten minutes?

Respondent: Yes.

Interviewer: And can you tell me what you did?

Respondent: Mowing that wretched lawn [laughs].

Interviewer: [Laughs] And the intensity of that?

Respondent: Well it's pretty intense because as you know where we are our front garden drops down.

Interviewer: Yeah.

Respondent: And we've got lots of vegie beds, raised vegie beds so I've got to get in around those so that's like back forwards but I tend to choose to

mow in a pattern which forces me to push the lawnmower up the hill so I can utilise…

Interviewer: Oh your strength building, that's well thought out.

Respondent: It's easier to go sideways but much harder to go up and down [laughs].

Interviewer: Yes [laughs].

Respondent: Sometimes we do, we've got two lawnmowers, we do it together at once but I've done it myself the last couple of times yeah.

Interviewer: OK, so that sounds pretty vigorous?

Respondent: Yeah it's about an hour.

Interviewer: An hour, and how often would you do that?

Respondent: Well with the season we've had up there it's been, I've pulled it out about as far as I can, about once every one and a half weeks.

Interviewer: Yeah.

Respondent: And I can't really, it gets too long if I let it go for two, and I can't be bothered doing it any more.

Interviewer: A number of women were saying they have to do it, oh ever so long.

Respondent: Yeah.

Interviewer: OK.

Respondent: And I garden. We grow our own vegies so that's pretty minimal, I mean we only have to pull a few weeds here and there and we try to get [son] to do as much of that as we can to encourage him to be active.

Interviewer: And inside?

Respondent: Yeah well it's pretty low maintenance because we only really have to run the vacuum over, we've got floorboards and we've got central heat pump heating so we don't have an actual fire and things like that. And it's a pretty smallish sort of house so it's not too bad.

Interviewer: So it's fairly moderate exercise?

Respondent: Yeah it's not too hard.

Interviewer: Usual regular bit of housework.

Respondent: Yeah, and I suppose for someone of my fitness, not that I'm bragging, it's not really doing much.

Interviewer: Uh-huh.

Respondent: Do you know what I mean, if you weren't fit it'd probably get your heart going a bit but it's not that demanding. We've only got rugs pretty much sort of thing, just the floorboard bits here and there.

Interviewer: OK, and does it change depending on the season and time of year?

Respondent: Housework?

Interviewer: And outdoor activities.

Respondent: Yes it does, housework, obviously the lawns don't need mowing as much in winter.

Interviewer: Uh-huh.

Respondent: The inside stuff would be just the same.

Interviewer: Uh-huh.

Respondent: Oh we do actually do, we run an accommodation cottage so we kind of change that too.

Interviewer: Oh right.

Respondent: So we clean that, that's my job to vacuum that one and make all the beds there, and usually we try to do that as a, we have a bit of a competition to see how quickly we can do that so that's pretty quick, doing beds and…

Interviewer: Sounds a good idea to get through it [laughs].

Respondent: Yeah [laughs].

Interviewer: I've always wondered how that went [laughs].

Respondent: So that house, that increases in the summer but goes quiet in the winter.

Interviewer: Oh OK.

Respondent: As the occupancy comes down a little bit.

Interviewer: So is that sort of self-contained?

Respondent: Yeah it's a full house so it's mostly…

Interviewer: Yeah so you only have to…

Respondent: Tell them the card to get the key and they help themselves and then when they've finished come back and clean up.

Interviewer: Yeah, so that's a regular extra?

Respondent: Yeah and of course I hang, do all the washing and hang all the linen out and everything like that.

Interviewer: Uh-huh.

Respondent: Not that that's really, that's not get your rate up but its stretching and those things I guess.

Interviewer: Uh-huh, it's extra than you know just your home. And if you wanted to is there anything that would have helped you be more active around the house or the yard?

Respondent: The only thing that I would, if I had more time I'd probably spend, apart from all the other wonderful things I enjoy doing I'd probably do more gardening.

Interviewer: Uh-huh.

Respondent: But I wouldn't want to choose to spend more time around the house because there's too many other more exciting things to do in life than have a house and yard looking immaculate.

Interviewer: Fair comment, OK. Now have you been physically active for at least ten minutes during your leisure or spare time, that other part of your time, in the last couple of weeks?

Respondent: Our leisure…

Interviewer: Because you were talking about gym and…

Respondent: Yeah, do you see that as leisure or is that…?

Interviewer: Yeah.

Respondent: Yeah, certainly have, we just started last week at the gym.

Interviewer: Yeah.

Respondent: So we're concentrating on split routine of lower body muscles one day and then upper body muscles the next.

Interviewer: OK.

Respondent: So that's things like own body weight plus dead weight whatever. Concentrating on leg strength, the upper parts of the leg so that, and [partner] got, she broke her Achilles tendon so she's got to try and work on her calf.

Interviewer: That's always tricky building that up again.

Respondent: Yeah and she's just upgraded her camera gear to a professional kit so carrying the weight for our day walks has become more challenging for her and probably me too because I've been taking that extra weight for all our other bits and pieces so we concentrate on that and upper body just to have upper body strength.

Interviewer: So that sounds fairly vigorous?

Respondent: Yeah.

Interviewer: And what's the duration when you go there?

Respondent: An hour at least.

Interviewer: An hour.

Respondent: Yeah it takes us an hour to do the routine.

Interviewer: OK, and that's during the day or at night?

Respondent: Yep during the day, in the morning.

Interviewer: Yep.

Respondent: When no-one else is there.

Interviewer: Yep, and you also mentioned your walks, bush walks?

Respondent: Yes, we like to get out and about when we can.

Interviewer: How often would you, but you combine your purposes anyway?

Respondent: Yeah, everything's sort of a bit blurred for us.

Interviewer: Of course it does.

Respondent: Which is good.

Interviewer: Yeah you're not the only ones, it sort of makes a lot of sense, you don't just…

Respondent: It does doesn't it.

Interviewer: That's what we're starting to find is that people are starting to look at the notion of activity in a much broader sense in your life.

Respondent: Uh-huh.

Interviewer: And that it often fits in to different purposes, it's not just I'll go there and that's what I do [laughs] and then I go and do something else. No its part of their day.

Respondent: I'd say probably as an average we'd probably do one walk once a fortnight.

Interviewer: OK.

Respondent: It'd be interesting to see what [partner]; whether she thinks it works out that way.

Interviewer: Uh-huh.

Respondent: That probably…

Interviewer: And that's fairly long…

Respondent: … once a fortnight.

Interviewer: … walk by the sounds of it?

Respondent: Yes, day walks.

Interviewer: Day walks.

Respondent: Yeah, so they would be walks of around duration probably about three to four hours or two to four hours. We'd like to do some longer ones but we're just waiting for [partner] to sort of increase her fitness on her leg.

Interviewer: So apart from the walks you do on the beach, oh you were saying you can't actually walk where you…

Respondent: No we've got to drive down the road two minutes.

Interviewer: Two minutes.

Respondent: On that [x] Beach.

Interviewer: Yep.

Respondent: I don't know if you know that one.

Interviewer: Yep I do, I know where you mean.

Respondent: And so we just park at the end closest to use, western end, and we walk right up around to the caravan park, the mouth of the river…

Interviewer: Yeah, yeah.

Respondent: … and back.

Interviewer: Yeah I know where you mean, OK. And with the gym I presume that's getting, you've got to get in the car too?

Respondent: Yeah that's just in [town].

Interviewer: Yep.

Respondent: In the [shop] car park.

Interviewer: Yeah, OK. And the bush walks are obviously a drive?

Respondent: Yeah.

Interviewer: Yeah OK.

Respondent: At least an hour I reckon.

Interviewer: Yeah, and that's in the general vicinity of your locality?

Respondent: Yeah, we've spent a lot of time…

Interviewer: Because you've got the [x] Ranges?

Respondent: Yeah actually we do, we use the, yeah that's another one, we do use, I forgot to count that actually, last week we, Mount [x] which takes about, we can do it now I think in 50 minutes.

Interviewer: Yeah.

Respondent: So we tramp up there 50 minutes, have a quick drink and then we come back down. And sometimes we'll do that instead of the walk on the beach.

Interviewer: Yep.

Respondent: More intense sort of hill climb.

Interviewer: Yeah good, and does this change depending on the season or the time of the year?

Respondent: Yeah it does, so we'll find ourselves much more active in the summer, outside active, than in the winter. You know there's nothing nicer than putting on shorts and a t-shirt and strolling…

Interviewer: Longer hours…

Respondent: Yeah.

Interviewer: … light too.

Respondent: Having said that though photography's always really good in the winter.

Interviewer: Yes.

Respondent: So we probably tend to do, well it's sort of different, because your daylight hours in the winter for photography are shorter and usually it's wet and slippery because we spend a lot of time down at [forest] doing natural values report or project for the last two years and we

did a book as well. So we spend a lot of time down there in the winter taking photos as well but it's a bit different in that it's slower because it's slippery. So it's more about core strength and not falling over [laughs].

Interviewer: [Laughs] Yes there is a difference.

Respondent: It's a little bit different that exercise.

Interviewer: It is yeah. And if you'd wanted to is there anything that would have helped you be more active in your leisure or spare time?

Respondent: Probably just more time.

Interviewer: Uh-huh. I'm going to talk about the physical environments now. We've talked about where you live and physical activity. So the last part's sort of just talking about the environments where you live and play and work and when we talk about physical environment we mean all the physical things that surround you like your roads, trees, parks, houses, shops, coast as you have, lights, whatever. And some of these things are things that people who live in urban areas have said actually may have an influence on their physical activity. So we're interested to find out whether they're relevant for you. Now can you tell me a bit about how easy or difficult it is to be physically active in your own neighbourhood?

Respondent: Yeah it is difficult because the terrain just doesn't really cater for it because its rocky and you can't really, there's not like open stretches of sand much that you can really get out and about apart from just kicking the football with [son]. And the road's dangerous so it's not good for cycling.

Interviewer: Yeah, the road walking along I imagine would be, you'd prefer to walk along the coast anyway?

Respondent: Yeah I mean from time to time we've walked on the train line around but then that's…

Interviewer: Oh yeah how does that work, I noticed that?

Respondent: Well it's really difficult because the ballast is big.

Interviewer: Oh.

Respondent: The ballast is alright when its not so coarse and then you've got the sleeper things in between, so you've got your head down all the time trying to tread on the…

Interviewer: Yeah, I used to live in the hills where a disused railway line and I used to try and walk along that, it's really difficult.

Respondent: Yeah it is.

Interviewer: And there's never really much room on the other side either.

Respondent: No.

Interviewer: So you're either sort of focussed on trying to hop over it [laughs]…

Respondent: Exactly [laughs].

Interviewer: … or you know your sliding down the back [laughs]. It's not much fun yeah. And that looked a really narrow gauge too.

Respondent: Oh yeah, yeah it is, and you've got those cuttings and things everywhere so you certainly wouldn't want to get stuck in there.

Interviewer: Pity isn't it because they've got the land there.

Respondent: Oh it's absolutely gorgeous.

Interviewer: Obviously they don't use it, its disused now the railway is it?

Respondent: Well from [city] to [city] it's not used but from [city] through to [town] it is used.

Interviewer: Is it?

Respondent: Oh definitely yes.

Interviewer: How often?

Respondent: Four times in a 24 hours period.

Interviewer: Really?

Respondent: Yeah, yeah definitely.

Interviewer: OK.

Respondent: They've got containers, lots of containers.

Interviewer: Yeah, so you have containers sort of rumbling past?

Respondent: Yeah.

Interviewer: OK so that, what you've been saying about what makes it difficult, now we're going to look at sort of different aspects and the first one is availability and accessibility to places to be active. So if I were to ask you about places to be active in your neighbourhood what sorts of things would you tell me about?

Respondent: Sorry give me that question again?

Interviewer: It's about places that are available and accessible in your neighbourhood, so if I was to ask you about that what places would you tell me about?

Respondent: Oh in my specific neighbourhood?

Interviewer: Uh-huh.

Respondent: Well nothing much really. Very limited, very, very limited. Well extremely limited, there's no footpath, the road's narrow, the cars go very fast, so the road's actually quite dangerous. Really you've only got the natural environment of the beach and things but only probably for fishing and a gentle walk around rocks and things like that.

Interviewer: So any recreational facilities that we've been talking about are more in [town]?

Respondent: Yeah.

Interviewer: So walking tracks, cycle paths, parks?

Respondent: Yeah, yep.

Interviewer: And the beach walk and gyms are all…?

Respondent: Yeah.

Interviewer: Yep OK, and they're all there but they're…?

Respondent: You've got to get in the car and drive to them yeah.

Interviewer: OK. Right, so not available and they're not accessible obviously [laughs].

Respondent: Yeah.

Interviewer: No. And does not having places to be active influence whether your active or not?

Respondent: Well I think what it does is forces you to have a mindset where actually you have to be consciously choosing to exercise.

Interviewer: Yep.

Respondent: If you were relying on some sort of, more of a passive view about it then you'd probably tend not to bother. So yeah structuring it means that you do get out and do it.

Interviewer: Uh-huh, OK. And if you wanted to be more active are there things or places that would help if they were available or more accessible?

Respondent: Look if that pathway went right across the front yard to the garden that would be so good, because we could hop on that, for example [partner] and I could hop on that and cycle into the gym or if we wanted a beach walk we could just cycle so far or we could even walk

so far around to the beach. [son] and I could go on it or [son] could even go on it as he gets older.

Interviewer: There's been talk about longer term plans of that has there?

Respondent: Yeah there is, so that whole…

Interviewer: How definite? Where's is up to?

Respondent: Each individual council I think is trying to source money to they can fund it.

Interviewer: OK so it's definitely…?

Respondent: Yeah and the [x] Authority's trying to help the councils to do that. But I mean it's a fundamental barrier for where we are.

Interviewer: Uh-huh.

Respondent: Without that then we can't really, and it would be really good for people in [town] too because, especially people in the west, they could come out and go to [town].

Interviewer: Yeah, it's not far at all. It would be good.

Respondent: It's not far yeah.

Interviewer: Beautiful coast too.

Respondent: It is a beautiful coast.

Interviewer: Destinations, now if I were to ask you about places that you could walk or cycle from your home what sorts of things would you tell me about? You wouldn't.

Respondent: No.

Interviewer: [Laughs].

Respondent: [Laughs] Zero [laughs].

Interviewer: No. Does not having destinations influence whether you're active or not? Like if you had, I don't know a recreation facility, oh friends as well, friends and shops and whatever?

Respondent: If we had that kind of infrastructure would we be more active? Probably would.

Interviewer: Uh-huh, OK.

Respondent: How much more I don't know because we're pretty committed as we are in terms of making sure we do exercise a fair bit. It would probably mean also mean also that we'd have to have more time to exercise.

Interviewer: Uh-huh.

Respondent: It's probably more to do with [son] I reckon, probably making sure, he would have more of an opportunity to exercise.

Interviewer: Uh-huh, yep.

Respondent: Combination of supervised and unsupervised which is always really important for kids as they're growing up to be able to you know have opportunity for unsupervised exercise. And friends, social for him too like as he gets older he'd be able to ride to his mates or his mates would be able to ride to him and things like that.

Interviewer: Yeah. And so these are sorts of things, like the next question which your answering now is what would help, those things would help in terms of more destinations?

Respondent: Uh-huh.

Interviewer: OK. Now neighbourhood design, and by that we're looking at the built or non-natural or man-made features of your neighbourhood. If I were to ask you about those features which are not natural, what sorts of things would you tell me about?

Respondent: Good or bad?

Interviewer: Yep.

Respondent: Well the road, see the road is popular with some road cyclists, you know people who are confident, proper road, like ones that go in groups and things like that.

Interviewer: Uh-huh.

Respondent: If that road wasn't so, if they changed the speed limit and if motorists would improve their attitude towards, because there's some pretty bad attitudes out there, I've been abused several times on the road cycling.

Interviewer: So if you put the speed limit down?

Respondent: Speed limit down, motorists changed their views then that would be one thing would make it more possible to…

Interviewer: Uh-huh, are there any options with that road? Because I seem to remember it's very narrow and there's not much room either…

Respondent: Physical space no.

Interviewer: … side.

Respondent: Yeah and the tightest point, you've got the cliff, the road.

Interviewer: So even if they were to do a shoulder there's nothing much there anyway?

Respondent: Space no, you'd have to slow the speed limit down and there'd be very good reason for them to because there's not really, it's only really for people, its only residential now because the [x] Highway goes…

Interviewer: Yeah I wondered that, it doesn't, yeah.

Respondent: It doesn't serve any other major arterial purpose or anything.

Interviewer: No, OK. And do the built features of your neighbourhood influence whether you're physically active or not?

Respondent: It certainly cuts out options.

Interviewer: Yep.

Respondent: So cycling, it really cuts out that cycling for a main option.

Interviewer: Uh-huh, cuts out walking as well.

Respondent: Cuts out walking yeah.

Interviewer: To get to the beach.

Respondent: Yeah cuts out walking as well. So really what it does is just limit any exercise around the house.

Interviewer: Uh-huh, and if you wanted to be more active are there any built features in your neighbourhood that would help?

Respondent: Like changing or new you mean?

Interviewer: Yeah like, yes, what change would help?

Respondent: Well the pathway thing we talked about.

Interviewer: Yep.

Respondent: Changes to the road.

Interviewer: Because that alleviates, if you had the pathway it alleviates having a footpath or…

Respondent: Oh yeah absolutely. Definitely my number one choice would be the pathway.

Interviewer: OK that's good. Now the other side of it is the aesthetics, the natural qualities. Now if I was to ask you about the aesthetic qualities or the attractiveness of your neighbourhood area what sorts of things would you tell me about [laughs]?

Respondent: Oh well it's wonderful, its world class.

Interviewer: Yeah.

Respondent: We're living on a nature reserve so…

Interviewer: You've got a lot of coastal vegetation there.

Respondent: Yeah.

Interviewer: And behind you've got…

Respondent: Little farms and farmland behind.

Interviewer: That goes up into the ranges.

Respondent: Just up there yeah, so natural assets absolutely fantastic.

Interviewer: And any other, the other side of it is things like rubbish and litter and graffiti?

Respondent: Pretty minimal.

Interviewer: OK. Do you think your local environment is aesthetically pleasing and attractive?

Respondent: Very.

Interviewer: And do the aesthetics of your neighbourhood influence whether your physically active or not?

Respondent: Yeah, well I suppose yes they do in terms of I guess because where we're situated we're lucky enough to be able to have a good view of what the weather's doing and things like that. So for us to be able to decide oh we'll go, it looks good now we'll go for a walk on the beach. We can see the environment where we are. I can look from my telescope right across to the beach we want to walk to and see what the tide's doing.

Interviewer: [Laughs].

Respondent: [Laughs].

Interviewer: Remarkable isn't it.

Respondent: Yeah and see how many people are there with big fat dogs [laughs]. So yeah.

Interviewer: Yeah, and if you wanted to be more active are there features in your neighbourhood that might help if they were more aesthetically pleasing?

Respondent: No I don't think there's anything aesthetically, it's actually fundamental infrastructure that would have to change.

Interviewer: Yep that's fine. Now one last area is just about safety, and I were to ask you about safety in your neighbourhood area what sorts of things would you tell me about? We've talked a bit about road safety.

Respondent: Yeah, look I think the scariest thing where we live is just that road. There's cranky young guys in cars that hoon around there like absolute damn maniacs sometimes.

Interviewer: Uh-huh.

Respondent: Which is a bit of a worry. But you know we can't really do much about that. I think if they changed the speed limit.

Interviewer: Makes it less attractive for them to hoon along.

Respondent: And some motorcyclists see it as a bit of a racetrack too.

Interviewer: Uh-huh.

Respondent: But you know, I guess you get that pretty much everywhere.

Interviewer: And are there any aspects of, we've already said about road safety influencing the activity you have, it limits, you get in the car and go to where you need to go, but does personal safety influence whether you're physically active or not?

Respondent: No.

Interviewer: No, you feel pretty safe out there?

Respondent: Yes, look I'm pretty worldly. There's no point being scared about things that are unlikely to happen. Everyone's different in that regard, I think you know if you've travelled, you know done travelling overseas on your own and a few things like that you sort of get through a bit of a fear thing.

Interviewer: You get a bit of a barrier that goes with that.

Respondent: Yeah I think so.

Interviewer: And it sounds like it's a pretty safe community too.

Respondent: It's pretty good.

Interviewer: Yeah and you know your neighbourhood. So if you wanted to be more active are there any personal or road safety issues that, if they were addressed, might help you to be more active?

Respondent: Definitely, just that one to do with the traffic, attitude of people, how people use that road, the speed limit and then overall attitude of motorists towards cyclists.

Interviewer: I think that's really important, to have been a cyclist, I've just taken it up again and like half my concern is you know is the attitude of hoons behind that wheel.

Respondent: Yeah absolutely. And you know there's a lot of, I don't understand why some people are bitter and abusive but I mean some of the…

Interviewer: And that they just can't to wait pass too.

Respondent: People that have abused me are like well mate you look like the sort of bloke who needs to get out and do some exercise, so what is this actually all about?

Interviewer: Yes.

Respondent: It's not about me actually being on the roads, you actually being angry that you're so lazy probably.

Interviewer: Yeah.

Respondent: I know that sounds a bit judgemental but I mean it's not very nice.

Interviewer: Well there's certainly an attitude people need to learn that if they don't know how to pass people on a cycle they can just wait [laughs].

Respondent: [Laughs] Yes.

Interviewer: It's like with horses you know, I always really slow down for horses…

Respondent: Absolutely.

Interviewer: … and the horse rider always thanks me because I know what it's like.

Respondent: You have to, you have to because those poor creatures…

Interviewer: It's not fair.

Respondent: It's so dangerous.

Interviewer: Absolutely.

Respondent: I mean if they get spooked.

Interviewer: Absolutely. OK, so just in conclusion before we finish up is there anything else that you'd like to tell me about today about either where you live or your physical activity or the physical environment that we haven't covered today?

Respondent: Probably the only thing, I know [partner] will problem hit big on this being someone who's very keen on gyms, is that whole corporate business sector and the availability to good quality gyms close by with good operating hours.

Interviewer: Oh, yes.

Respondent: It's been a real problem where we are.

Interviewer: And the cost as well?

Respondent: [partner] probably knows, because she's had a lot of history of being in gyms whereas this is the first time I've really done the gym work.

Interviewer: Uh-huh.

Respondent: I think the price is reasonable, but it's pretty damn casual.

Interviewer: Oh OK.

Respondent: Didn't have to sign anything.

Interviewer: Oh OK.

Respondent: Just paid cash [laughs], you know it's pretty how are you going. But I mean there's some gear there but it's not like, her experience that she's had elsewhere.

Interviewer: Uh-huh.

Respondent: You know, I mean you've had state of the art stuff and all that sort of thing. It certainly, and probably for some, I mean I'm lucky in that [partner] is our personal trainer and she's had a history of structured sport and exercise but if I hadn't had her then I would be relying on personal trainers so you'd want to make you had access to good personal trainers too.

Interviewer: Yes, yeah, because how does the gym work? Do you just go in and use the equipment unless you've got someone like a personal trainer that shows you and works out the best thing for you.

Respondent: [Laughs] Yeah, we just went in there.

Interviewer: To avoid injury and all that sort of stuff [laughs]?

Respondent: Oh no very casual yeah.

Interviewer: Really [laughs]?

Respondent: Yeah they said have you done gym stuff before and I said oh well [partner] has, she knows what she's doing.

Interviewer: Oh well that's alright, fine [laughs].

Respondent: Oh well off you go [laughs]. I mean that's nice in a way [laughs]. But anyway, [partner] probably more a person to comment on whether you know its standard or the cost, availability and all those things.

Interviewer: Because a lot of people don't know.

Respondent: It's very important, I mean we can only rely so much on what we do around the house or our personal interests and as we're women and

as we're getting older, I mean as you full well know we've got to be able to maintain good overall strength haven't we?

Interviewer: Yeah, health and wellbeing.

Respondent: Health and wellbeing.

Interviewer: Very important.

Respondent: It's very important and…

Interviewer: Yep, you've got to use it.

Respondent: Use it or you lose it [laughs].

Interviewer: Yes [laughs].

Respondent: Yeah and it's like things, they sneak up on us don't they? I mean it's OK…

Interviewer: Exactly, things you take for granted and then you go oops [laughs].

Respondent: It's alright when you're in your 30s you know.

Interviewer: Yep.

Respondent: When you're doing things and probably even like I don't know for mothers too like, they're raising children and they're carrying kids around and there's all those things.

Interviewer: Yes.

Respondent: But as soon as you get, I found for myself talking personally, as soon as you get 40 your no longer, you can no longer rely on youth.

Interviewer: Uh-huh.

Respondent: You now have to make an effort to set yourself up for the next 40 years.

Interviewer: Yes, yeah.

Respondent: So you know, you need expertise around you to be able to achieve that.

Interviewer: Yeah and its interesting because it touches on what somebody else was saying about, it's like, and you have to find that pathway yourself. She was commenting on it would be really helpful if there was sort of more information or help or support around.

Respondent: Uh-huh.

Interviewer: But nobody seems to touch on that, it's more you've got to find your own way by learning often the hard way [laughs].

Respondent: Yeah that's exactly right. I mean thank goodness I've got [partner], I wouldn't have any idea on how to do it, what a split routine is or what muscles we have to work on.

Interviewer: Uh-huh.

Respondent: Or no you need to stop now because you're shaking because you're going to be dreadfully sore [laughs].

Interviewer: [Laughs] That's right, good point.

Respondent: Yeah, don't put too much weight on there because if you twinge you'll break something, you won't be able to come back tomorrow.

Interviewer: [Laughs].

Respondent: So yeah, it's all good. But you're right, it's important.

Interviewer: Yeah.

Respondent: Very important.

Interviewer: And we were talking about that in terms of it's really important to, rightly so you want to avoid those medical conditions we're talking about just about how the general health that you have, well the focus should be more on health and wellbeing and how you can do that better.

Respondent: Well yeah absolutely and probably the big thing that got us going to the gym was, as I said about [partner] with her Achilles and this camera gear, we went down to [x] Falls which is in [forest] probably about three or four weeks ago and she just really, really struggled.

Interviewer: Yeah.

Respondent: And she's just said look you know I've got to budge this weight and I've got to do something about the Achilles injury otherwise I'm not going to be able to get out and do this. And for me that was the switch of oh well you're absolutely right, if we want to be able to have overall quality of life doing the things that are important to us then

we need to make a structured effort, we can't just rely on banking on what we happen to do on a casual basis.

Interviewer: That's a fair comment.

Respondent: Uh-huh.

Interviewer: Thank you very, very much for that.

Respondent: Thank you.

Interviewer: They're stuff…

Respondent: They're not as scary because then I have my big headphones and my microphone, you've got to work really hard to get people to try and forget that it's there.

Interviewer: This is one you sort of don't really notice.

Respondent: Uh-huh.

Interviewer: OK so first of all I thought we might just start by talking about the place where you live and so I wanted to ask you what's it like to live where you do? Your likes or dislikes.

Respondent: I like it because it's quiet. We're at the end of a road.

Interviewer: Oh OK...

Respondent: So we're at the end of [x] Road and our farm just borders the bush, so it's the end of the residential area. There's quite a lot of houses on the road, and then after us it's a gravel road and it goes up in to [x] Reserve...

Interviewer: Oh OK.

Respondent: And through to bush walks and (laughs)…

Interviewer: So you overlook the coast and the forest…?

Respondent: So we've got sea views and we've got mountain views, we can see the whole [x] Range from our place, so I really love the scenery and I love the quiet. I mean I grew up in the country, so it's what I like...

Interviewer: You love it…

Respondent: Yeah, I like the space, not having neighbours looking (laughs).

Interviewer: And how far out of [town] are you? You say you're on the verge of the residential…

Respondent: It's about eight or nine kilometres south.

Interviewer: OK.

Respondent: Not as the crow flies, but along the road. Yep, so…

Interviewer: Yep.

Respondent: … it's sort of a funny spot because it's not on the way to anything really, and while it's a tourist area it's not very well

promoted so we (laughter) don't really get much traffic. We get a little bit of traffic on weekends, bushwalkers and things and we get a few four wheel drivers going through the rough road because you can end up at [town] yeah.

Interviewer: Yeah.

Respondent: And we have lots of animals, so I like… I need the space for the animals.

Interviewer: So you've got a bit of space there, is it a house on how much land?

Respondent: 93 acres.

Interviewer: Whoa how lovely (laughter)!

Respondent: Yeah so it's, we're trying to… it's a working farm sort of. My partner's on the farm full time now.

Interviewer: Great. And how long have you lived there?

Respondent: Two years in a week or so.

Interviewer: Oh celebration time.

Respondent: (Laughter) yeah. Not that long I suppose yeah.

Interviewer: Alright, and your reasons for living there?

Respondent: Because both of us had grown up on farms and we wanted to have our own bit of land and we wanted to live a fairly self-sufficient lifestyle, so enough land to grow our own fruit and vegetables and all our meat as well, and to be able to make enough money from the land hopefully one day that we don't have to go to office jobs as often and just as well to have that style of lifestyle for our children if we have children to grow up in. Wouldn't want them to grow up in town.

Interviewer: OK. Now in research with women living in it's as you probably know from talking to [staff member], most of the research has been done in urban areas. So people often refer to their local neighbourhood, and I'm interested in understanding whether that term has relevance to you in a rural setting. So what does the word neighbourhood mean to you?

Respondent: It doesn't mean much in the place that I live.

Interviewer: Uh-huh. Uh-huh.

Respondent: It sort of, I think because we're quite close to town really, you know it's ten minutes into [town], we're not, we don't even have a proper name for the country area that we live in, so it's just [x] Road and we still say that we live at [town] even though that can mean the town too. And while we're, we've got four neighbours and we're close to those neighbours but we're not really close to any of the other people on the road. And they do have, they have a party once a year for the road.

Interviewer: Right (laughs).

Respondent: But we, I wouldn't say that we have a community or a neighbourhood, whereas if you lived half an hour out of town, because you've got that bit of extra space there's more of a need for a community. And most of us have town jobs as well, most of the people who live in our road, so they're driving to [town] or [city].

Interviewer: So the word neighbourhood doesn't really strike relevance to you, I was actually going to ask you does the word community?

Respondent: Maybe a little bit more (laughs).

Interviewer: Yeah, OK. What might it mean?

Respondent: Because I suppose where if you how you know say at this road party when you meet other people on your road you do feel connected to them, and I probably feel a bit more connected to [town], even though I don't do my shopping and I don't actually get to spend that much time in [town], but I like to feel that you know, I know the beach and I know the shops and you know, you know the shop keeper and you feel part of that wider community.

Interviewer: Uh-huh.

Respondent: And I think a neighbourhood is a lot closer and probably supportive than a community.

Interviewer: Uh-huh, so if…

Respondent: Can we turn the fan off now?

Interviewer: Absolutely.

Respondent: (Laughs) I've warmed up.

Interviewer: You've warmed up, good.

Respondent: Thanks.

Interviewer: That's great. So in terms of community which you are saying it has probably a bit more relevance to you, does it have a distance to it?

Respondent: Yeah. I think that's what I mean when I sort of said when I think of neighbourhood as being a bit more supportive I mean thinking about things like Neighbourhood Watch or neighbour's closeness, keeping an eye out for each other. Community it's more you're part, part of a bigger picture but you don't know everybody in it.

Interviewer: Uh-huh.

Respondent: But you sort of feel connected to a place maybe.

Interviewer: Oh OK. So if you needed to put a sort of distance around it like would it be community, would it be kilometre wise, you're immediate area and [town], or how, what sort of size would you call it?

Respondent: It wouldn't go as far as [town] and it wouldn't go as far as [city], but it also wouldn't go as far as [town] or [town] or [town], definitely just that… because I mean the north west coast is all in valleys and hills and each they all run down to the coast and I think each, each one is its own…

Interviewer: Own community.

Respondent: … community probably and once you go over to the next hill that's a different community. So I'd say at [town].

Interviewer: [town] and?

Respondent: And around the [x] Range. Along the Range.

Interviewer: Range. So what sort of distance?

Respondent: How many kilometres?

Interviewer: At a guess?

Respondent: You know… maybe 15 kilometres. What's from one side of the circle to the other? Di…?

Interviewer: Diameter.

Respondent: Diameter yeah, not radius. Yep.

Interviewer: Yeah, yeah.

Respondent: So maybe about 15 kilometres.

Interviewer: Yeah, yeah that gives a bit, yeah no that's good. Now I wanted to talk a bit about physical activity, and that's a really broad term as you know. So we're really interested in in what the term physical activity actually means to you. What does it mean to you?

Respondent: Pushing your body and making it work hard. There's sort of extremes aren't there? But because there's people who sit in chairs (laughs) and don't do much at all (laughs) and then there's physical activity of gardening and doing general jobs and then there's the exercise so I believe more at the exercise deliberate sort of end of things.

Interviewer: Uh-huh, that's what you'd see physical activity as being for you?

Respondent: Uh-huh.

Interviewer: OK. And do you consider yourself physically active?

Respondent: Yes but no. So I suppose I should go back to the last bit. Maybe I should say that physical activity is some of that gardening and that sort of thing as well, because I'm I'm not, I don't exercise but I I always, I don't sit down.

Interviewer: Uh-huh. You're active.

Respondent: I'm always doing something.

Interviewer: Uh-huh, so it's about the activity in your life rather than a particular under the heading of exercise.

Respondent: Uh-huh.

Interviewer: OK. And so you do consider yourself to be physically active in that sense?

Respondent: Yep.

Interviewer: That broader sense?

Respondent: In the broader sense yep.

Interviewer: Yep, yep that's fine.

Respondent: But not in a fitness sort of way.

Interviewer: Yep. Whatever that...

Respondent: (Laughs).

Interviewer: And I think that's the intriguing thing about this sort of study is it starts to talk about a much broader notion of actually what people think about what it means for them in their life and…

Respondent: A lot of blurred edges too.

Interviewer: Yeah, yeah and it's interesting there just seems to be a bit of a theme going that some people are clearer about it than others, there's a you know range in there, but it's something you do in your whole life, it's not sort of an add on of you know something I do three times a week by going to this place.

Respondent: Uh-huh.

Interviewer: Gym or whatever. And where does physical activity fit in your life? Is it high priority? Something that you've sort of got moderate interest or no interest at all?

Respondent: So when we talk about this are we, are we…

Interviewer: This is for you, how it fits for you.

Respondent: … but it means exercise more?

Interviewer: Yeah.

Respondent: Oh (laughs).

Interviewer: But under your definition of what you see as physical activity.

Respondent: Yeah OK.

Interviewer: How does it rate for you?

Respondent: Well it's important say during my day at work that I like to go for a, like if I walk into town I like to walk briskly (laughs), make

myself puff up the hill on the way back to work. I probably, I wouldn't always go for a walk, but I like to get the blood pumping when I've been sitting down at my desk for a long time. And then when I come home I like to at least walk around some of the farm you know, go and check on things, and then I like to do my gardening and that wears me out you know I do a lot of hand digging and pulling weeds and that feels really good. And I like because we're on the edge of the bush I like to sort of walk around the bushy parts and… but the the more you know deliberate going and putting on your (laughs) sneakers sort of physical activity isn't as important. So it's important to me you know I don't I mean I sit down at work but I never sit down at home except to eat my tea (laughs).

Interviewer: Uh-huh.

Respondent: So it's important when I'm at home that you're doing jobs and you're moving around constantly. But it's not a high priority to go and you know to do deliberate exercise.

Interviewer: No. So within your definition of physical activity it's important. It's within your life, yep. OK that's great...

Respondent: Uh-huh.

Interviewer: Now I want to actually have a look at at bit more about physical activity and different parts of your life. And in our research we often think about physical activity as any activity that lasts for at least ten minutes. Health benefit affects, so it could be any exercise that you feel a bit like you get a bit of heart rate up, feeling warm, a bit of huffing and puffing. So within that definition, I'd like you to think about your physical activity over the last two weeks OK? Now starting at work because you start at… yep, in the last past two weeks have you been active at all at work for at least ten minutes of that time? You started to talk about how you like to…

Respondent: Go for walks.

Interviewer: … go for a walk yeah, so.

Respondent: Yeah, I'm just trying to remember the last two weeks because so much goes on (laughs).

Interviewer: Yeah, or just pick a sort of yeah because you are really busy and you do different things, if it's easier pick a sort of sample two weeks that seems to fit more for you.

Respondent: Uh-huh.

Interviewer: Because you know like sometimes people are away, I was just talking to someone yesterday who'd been away for ten days, so it sort of wasn't…

Respondent: Yeah skews it a bit, yeah.

Interviewer: … quite different.

Respondent: We had two days of [event] last week and that certainly involved a fair bit of physical activity walking around the [event] site, so that's not…

Interviewer: (Laughs).

Respondent: … not typical but I suppose I would maybe one day of each work, of each week I'd go out doing a story where I'd need to do a bit of walking around a farm. Go exploring somewhere in a property or in the bush looking at something, but that's probably only once, once a week.

Interviewer: Uh-huh. And what would be the sort of intensity? Would it be steady, or moderate or vigorous exercise when you're doing that?

Respondent: So steady's faster than moderate is it?

Interviewer: Ah steady's more slower than moderate.

Respondent: Oh slow. So steady, moderate.

Interviewer: Uh-huh, and vigorous. Whatever words you want to use.

Respondent: I'd say moderate.

Interviewer: OK, yep.

Respondent: Because often you have to stop puffing before you can do your interview (laughs).

Interviewer: I imagine walking up hills, down valleys (laughs) they're everywhere. And the duration, how long would you be walking for at that point?

Respondent: I'd only say ten or 15 minutes.

Interviewer: Uh-huh and you've already told me how frequently you do that, every sort of couple of weeks or so.

Respondent: Uh-huh.

Interviewer: OK, and that would be during your work day?

Respondent: Uh-huh.

Interviewer: OK and is it something you usually do? Does it change with depending on the season or time of year?

Respondent: Yes I do less in winter (laughs). Generally you know my job being a rural reporter you're a bit led by the seasons, so there's a lot more going on in the warmer months when we're growing lots of things, so I go out and about a lot more during that time and in winter I tend to do more office work and going to conferences and meetings so not so many farmy, outdoory things.

Interviewer: So seasonally it might actually work out that in summer you do more than once a week.

Respondent: Uh-huh maybe.

Interviewer: And in winter you would probably do less in winter…

Respondent: Nil (laughs).

Interviewer: Nil (laughs) fair enough.

Respondent: Occasionally.

Interviewer: Yeah because also things, things tend to sort of, yeah as you said it runs by the season and the harvest and the plantings anyway in terms of often working with rural women I have in a health capacity, if you want to do sort of health care education work, you sort of wait to… sometimes you wait to the winter when it's we don't certainly don't do it in harvest season and seeding season. Yeah so you're sort of…

Respondent: Calving season (laughs).

Interviewer: Yeah, sort of times that you don't do things because it's too busy.

Respondent: Uh-huh.

Interviewer: OK, now another part is in the last two weeks have you walked or cycled for at least ten minutes at a time to get to or from places?

Respondent: No. That's because we live too far out (laughs) even though it's only…

Interviewer: Yeah, yeah you tend to drive.

Respondent: Yeah.

Interviewer: Yep.

Respondent: Yeah I drive everywhere that we're going.

Interviewer: Uh-huh.

Respondent: Probably if we didn't live up a mountain range I might (laughs).

Interviewer: It's quite steep is it?

Respondent: Yeah, yeah, so going down would be oh no, it's actually all up and down.

Interviewer: Uh-huh. And if you had wanted to, is there anything that might have helped you use, well walk or cycle?

Respondent: Ah…

Interviewer: From place to place.

Respondent: Well our road from our place to [town] is very narrow, it's pretty dangerous. So there's, there's nowhere to walk safely or ride…

Interviewer: Uh-huh.

Respondent: … between our place and [town]. I suppose if the road was wider (laughs) if there were some, there are no guards either on the side of the road, there's some quite sheer drops off the side of the road into the valley.

Interviewer: Yeah you've got a cliff one way and…

Respondent: Yeah you've got nowhere to go if…

Interviewer: … and the coast…

Respondent: … if a car comes. And I suppose if the road just wasn't so up and down (laughs) it would be a huge use of energy, but yeah there's no accommodation for pedestrians at all. You can barely fit two cars.

Interviewer: OK.

Respondent: On the road.

Interviewer: Likewise with work, is there anything that might have helped be more active at work?

Respondent: Probably not because I like to get my work day over as quickly as I can so I don't take an hour lunch break or even a half hour lunch break.

Interviewer: Yeah, you prefer to sort of work through them then?

Respondent: To squeeze it all in.

Interviewer: And then go home.

Respondent: And go as soon as I can.

Interviewer: So you've got some time at the end of the day.

Respondent: Uh-huh.

Interviewer: Fair enough.

Respondent: And I mean at the [business] in [city] they have a walking group (laughs).

Interviewer: Oh OK.

Respondent: They try, they bring the listeners in, I don't know where they're going at the moment because it's cooled off a bit. Certainly during the warmer months and when it was light as well the breakfast presenter would come off air and they'd all gather and so they'd get listeners would go on the walk.

Interviewer: Oh lovely.

Respondent: I mean if there was, we've only got five people in our office, but if there was some sort of group activity, or if there are other businesses in the area that went for a walk you know at 7.30 in the morning you know it's flexible enough at work that I can take half an hour out to go for a walk. I think the [business] also

had a walking group for a while, but it didn't suit the day. We get really cold in our office and I joke about doing exercises (laughs).

Interviewer: (Laughs).

Respondent: With our you know, it would be nice to have all of five of us doing star jumps…

Interviewer: Yeah.

Respondent: … and running on the spot together.

Interviewer: It'd be a lot warmer.

Respondent: But you feel a bit silly when you're doing it by yourself.

Interviewer: Right, so it does help if it's a group activity?

Respondent: Yeah.

Interviewer: You're just having somebody else around too to do it.

Respondent: Uh-huh.

Interviewer: OK, fine. Now another part of your life is in the last two weeks have you been active around the house or yard that means inside or outside your actual home for at least ten minutes of the time?

Respondent: Over the last two weeks?

Interviewer: Uh-huh.

Respondent: Yes. I've been madly digging my winter garden.

Interviewer: Oh, (laughs) how's it going?

Respondent: Slowly. Lots of weeds. So weeding and digging. Are we including farm as well?

Interviewer: Yes, all of that.

Respondent: So moving, moving pigs, and moving cattle and moving sheep and I'm just trying to think of any other work. Yeah we did a big farm yard tidy up at the weekend, so lots of lifting pallets and moving stuff.

Interviewer: Uh-huh.

Respondent: Tidying up.

Interviewer: Uh-huh, so more outside than inside?

Respondent: Oh just did a big houseclean at the weekend too for Mother's Day (laughing).

Interviewer: Oh wow.

Respondent: (Laughs) and when I houseclean I try and get it out of the road quickly, so I'm pretty vigorous with my floor washing and dusting and things. But no running on the spot inside (laughs).

Interviewer: (Laughs).

Respondent: Just vigorous cleaning.

Interviewer: OK so bearing those two activities in mind, the intensity of it, what would you describe it as? Moderate or vigorous?

Respondent: I suppose the inside would be moderate, although I do get pretty hot and have to take off layers, but outside I work pretty hard so that I puff.

Interviewer: Yep, Uh-huh.

Respondent: And have to have spells and have a drink (laughs) and then go back to it.

Interviewer: Good. And how long would you have done that for?

Respondent: So are we talking about each…?

Interviewer: Yeah each.

Respondent: Each activity?

Interviewer: Yeah Uh-huh.

Respondent: Across the whole period?

Interviewer: Uh-huh. Does it help to answer the question of how often you might have done these activities first and then how long?

Respondent: I'm just trying to remember what I did the weekend before (laughs).

Interviewer: Because there's about three activities you've been talking about.

Respondent: Yeah.

Interviewer: You've been talking about the garden, so…

Respondent: Because I've done a few hours in the garden, maybe I've done three hours, three hours of hard work in the garden.

Interviewer: Over the last couple of weeks, Uh-huh.

Respondent: I reckon because it's a big job clearing for the garlic and the barasicas spreading mulch.

Interviewer: And the clean-up.

Respondent: And the clean-up, yeah I probably did… oh outside?

Interviewer: Uh-huh.

Respondent: I'd say two hours of that. Housecleaning I did about three hours (laughs).

Interviewer: Uh-huh.

Respondent: Mum was coming for afternoon tea and it's always better just to have a clean house.

Interviewer: (Laughs). OK so OK so when did you do it? That was mainly over the weekend?

Respondent: This was over just this weekend. I can't remember before that.

Interviewer: That's OK.

Respondent: (Laughs) I've got no idea.

Interviewer: I have that sort of memory… what week are we in? What did I do? And OK so and who did you do it with? Was it by yourself or with others?

Respondent: Oh well my partner [partner] helped a bit of the gardening for a while, he lasted about half an hour (laughs), but he probably got more done than I do in a long time, and yeah I did the yard work by myself. Oh he might have done a little bit, just a couple of heavy things, so mostly by myself. And certainly housecleaning by myself.

Interviewer: Uh-huh. And is that what you usually do or was it one off?

Respondent: That's what I'd usually do.

Interviewer: OK. And does it change depending on the season or the time of year?

Respondent: When it's really hot in summer I don't do much active work during the day. I might do little bits of gardening in the evening if it's cooler because I've got very fair skin I don't, I don't like to go outside too much at that time. And in winter because I got

really cold I try and be fairly active so I'll go and shovel my compost heap or go and do some digging just to warm up and get the blood going even though it's so cold outside (laughs). It helps warm you up.

Interviewer: It's amazing when you start doing things isn't it?

Respondent: Uh-huh, yeah. So so it doesn't, I mean unless it's, it gets pretty windy at our place and it's not very nice when it's blowing a gale to go outside, but you know probably not it wouldn't change that much across the seasons.

Interviewer: Uh-huh. And if you had wanted to, is there anything that would have helped you be more active around the house or the yard?

Respondent: Just more general stamina (laughs) because I work pretty hard during the week at my job and I don't get very much sleep because I start work so early and I just I just don't sleep very well, so often at the weekend I don't have the energy that I want to do as much around the farm and in the garden. I get worn out, so I suppose just better general physical health, physical stamina would allow me to do more.

Interviewer: Uh-huh. And the other, the last part of your life…

Respondent: And not having to do so much paperwork (laughs) too that's another thing…

Interviewer: Yeah, that would help too. To be able to get up and do things (laughs). Have you been physically active for at least ten minutes during your leisure or spare time? Whatever you may see that as being.

Respondent: So over what period?

Interviewer: Over the last couple of weeks.

Respondent: Well yes, that's what I, that's my leisure time isn't it, the time we've been talking about.

Interviewer: Yep.

Respondent: The gardening and farm work and things, yes.

Interviewer: And mainly by asking these questions it's to make sure that we sort of cover the whole of your life.

Respondent: Uh-huh, I guess its perceptions.

Interviewer: And some of them get covered in other parts and… OK and we've yep... and is there any other thing that you want to say about what might help you be more active?

Respondent: Just generally?

Interviewer: Yeah.

Respondent: I suppose because, because we have a farm and I have to work off farm you know, there's there's just not enough time (laughs). So you go to work, I leave at 5:30, you get home mid to late afternoon, you know yesterday I got home at five o'clock and …

Interviewer: It's a long day isn't it?

Respondent: … then it's starting to get dark and you do jobs that you have to do and then it is dark and then you cook tea and then I work on the computer for another couple of hours for work.

Interviewer: Oh my goodness.

Respondent: And and then you know, but it's about priorities I suppose. I would like it, I know you know I'm an intelligent person I know that I need to be doing more exercise you know to get my heart rate up for what is it? Half an hour three times a week (laughs) and I'm certainly not doing that and I know that I'm not that physically fit you know I'm always active and I've always got a lot of adrenalin and I don't stop, but I'm not, I'm not very strong so when we go for a bushwalk I get really worn out because I hardly, don't do that. And I, I suppose and so when you when you're in the situation with a farm and a job and we haven't got children, that'd be an extra thing but there's a lot of expectations so you go to work you know I want to still cook dinner most of the time. You do the cleaning, you do the paper work you do the bills as well.

Interviewer: Because you were saying you're going back home usually to there were things that you did when you got back, now I don't know whether you used the word chores or what, but…

Respondent: Yeah jobs, jobs to do.

Interviewer: Farm jobs?

Respondent: Yeah, just some feeding animals and checking water and...

Interviewer: Yeah exactly well that's…

Respondent: … counting animals and tidying up.

Interviewer: No that's important, that's things that you do every night?

Respondent: Yeah, yep.

Interviewer: Yep, yeah, that's all activities. Uh-huh.

Respondent: Uh-huh, but I mean I suppose that if yeah as I say it's about priorities and I'm sure that you can squeeze in half an hour if you really want to if it was, if I saw it as more important I suppose what would I do I'd I wouldn't do such an involved dinner that takes me an hour to cook, or I'd put something in a crock pot and then I'd say this is the time because I also think that women you know in this farm situation, they, they you know it's horrible in the 1950s but they still feel it's their duty to, I mean I'm a young person, I still feel this is my duty to produce a nice meal and I produce a meal you know made from all our home grown vegetables and our meat and you know, you want to do that and you want to have the house tidy and you want to you know, do everything right and I think women just put themselves at the bottom of the list. And if they had children it would be even worse because then they'd be doing homework and all that sort of thing, and what is it? More than half of farming families in [state] now have a somebody who works off farm and most of that's the women, so it just piles up further and further the amount of jobs that they do, because and they want to be involved with the farm too, they don't want to just you know… leave it so…

Interviewer: So it becomes a real juggle and balance of…

Respondent: Yeah they have to be quite selfish. I mean occasionally I will go for a run or a walk and I'll have to be strict, you know no I can't come and help you with this now…

Interviewer: But there's always something else that you could be doing, I know what you mean.

Respondent: Exactly, you know.

Interviewer: You tend to… but the time for you tends to be at the end of all the rest of the list.

Respondent: Uh-huh, but I suppose if, I mean I know about the need for exercise. I suppose I'm lucky that I'm slim and I don't have to actively work on it, I've just got that sort of metabolism, but I suppose if it was put you know, in terms of health promotion, if it was put more in the front of my mind, OK maybe I'm fit now but you know my dad was the same shape as me and he had a triple bypass and lots of strokes and ended up dying of heart disease, so if you if it gets put into people's brains more about the longevity that they'll have or the impact of exercising now on their later life and you know reducing cancer and heart disease and all those bigger things that often you don't realise you know, until you're getting a bit past it. Because you know while you're while you're busy and feeling alright you don't probably see the importance of it in the long term, and it's easy to put it, put it off. I'll exercise when… you know when I've got an hour's leisure time every day (laughs) or when I don't have to do this and this and this…

Interviewer: (laughs). Or you know, when they you sometimes you go, 'oh do I just want to stop (laughs)?' It's a hard one, (laughs) yeah.

Respondent: Yeah, and I suppose maybe if there was, I mean we talked about neighbourhood and community and there's a few people who walk by themselves, but you know if I had somebody of a similar sort of age or with a similar sort of…

Interviewer: Interest.

Respondent: … style of lifestyle. I mean we've got people who are retired and around us and people who are at home all day, some of them, so they can walk when they feel like it and they'll go for walks during the day, but you know if there were I maybe I would walk with other people perhaps. And as well because we're at the end of the road and we do get these occasional four wheel drives and things coming through it's a little bit scary so I wouldn't walk towards town because we talked about the narrow road and no room for pedestrians, but I can walk up the bush road but it, you know it's a fairly narrow gravel road and you do get people sort of coming through unexpectedly going very fast and there's, of course there's no lighting, it's very dark in the bush.

Interviewer: Yep.

Respondent: And maybe safety in numbers (laughs).

Interviewer: Yeah, yeah, yeah absolutely.

Respondent: It would help there.

Interviewer: Yeah, no that's fine. Now I actually wanted to go and talk about physical environments.

Respondent: Uh-huh.

Interviewer: Because we've talked a bit about where you live and your physical activity, and so now it's about your physical environment about where you live, work and play, so and when we talk about physical environment I mean all the physical things that surround you, the things that you've just started talking about, the roads, trees, houses, shops, traffic etc., and some of these things are things that people in urban areas have said might be important for their physical activity, so we wanted to sort of check with, ask you whether they're actually relevant for you and you managed to actually start talking about that...

Respondent: Uh-huh.

Interviewer: Can you tell me a bit about how easy or difficult it is to be physically active in your neighbourhood? In sorry… now how do we refer it to it? In your area?

Respondent: Uh-huh.

Interviewer: In your area. Yep, yep.

Respondent: Uh so we've talked about the on the road that's bitumen…

Interviewer: Yeah.

Respondent: … that that's narrow.

Interviewer: Yes, and you've talked about the dirt road that's narrow…

Respondent: Fairly unsafe, yeah and then so then the dirt road's safe as well narrow and you don't feel very safe. I mean you can dash into the bush I suppose rather than the other way you've got to dash over the side of a…

Interviewer: So it's not exactly very easy is it (laughs)?

Respondent: No.

Interviewer: No.

Respondent: What else was I just thinking? I can't remember. What other physical things? I suppose…

Interviewer: Well more about how easy or difficult it is.

Respondent: Uh-huh …

Interviewer: I mean for instance when it's dark you probably don't have any lighting out there and all that sort of stuff…

Respondent: That's the thing you know I mean especially in winter when it gets dark so early. Because I lived in town for maybe four years, something like that when I came back from uni. How many years? Oh I can't remember how many years it was. And I, I would do a lot more… I did a lot more exercise, so it's interesting to compare that, at one stage I'd go to the gym like three times a week because the gym was only five minute's drive away and it was really convenient, or I'd go for a walk every day and there were nice footpaths to walk on, you didn't have to walk on the

road, you could get in [town] you could get where I lived you could get a nice mixture of flat and hills and (laughs)…

Interviewer: Yep, yep, yeah.

Respondent: … go a different way and look at different things and because there were street lights you wouldn't have to be worried if you were in the evening if… you felt a lot safer.

Interviewer: Yeah.

Respondent: And lots of comings and, you know there's always traffic, didn't feel…

Interviewer: So talking about some of these things in a bit more detail, availability and accessibility of places to be active. If I were to ask you about places to be active in your area, what sorts of things would you tell me about?

Respondent: Well we're lucky because we're against the [x] range that we've got this amazing natural place that you can exercise, so I can drive a kilometre up the road or I can walk a kilometre up the road and I can do a bush walk to Mount [x] which is only an hour to the top and less coming back. And then there's a lot, you know day walks and all that sort of thing, so there's all these bushwalks in the area. And then I suppose only seven kilometres down the road there's a sports centre, but I haven't had anything…

Interviewer: Mmm.

Respondent: (Laughs) yeah I haven't investigated it at all. There's I think you can play squash and basketball and there's a bowls club and a tennis club and a golf club all in one, so I suppose seven kilometres that's not actually very far away, but I haven't, I've never investigated that.

Interviewer: It may not be of interest to you.

Respondent: Uh-huh.

Interviewer: It's interesting to see what facilities there are in the area.

Respondent: Uh-huh.

Interviewer: And so there's walking, walking tracks, cycle paths, anything?

Respondent: Yeah there's mountain bike tracks through the [range] as well.

Interviewer: OK oh OK.

Respondent: But I suppose you know from our place to a place where you'd feel safe to start on a bike you'd have to drive, drive a few kilometres, you wouldn't ride from our place on to the tracks.

Interviewer: Again because of that safety feature of the road.

Respondent: Yeah the narrow road and the hoons in their four wheel drives and there's trail bikes as well. That's something about in the [range] that the trail bikes share the paths and…

Interviewer: Oh.

Respondent: … they go fairly fast (laughs) so that would, I mean if I was going to cycle I'd be nervous about that, especially at weekends. And I mean there's horse riding tracks too and that'd be the same thing.

Interviewer: Uh-huh. And so are these places convenient and easy to access?

Respondent: Well I'd say the…

Interviewer: To some degree but they…

Respondent: Yeah.

Interviewer: … mainly involve driving don't they?

Respondent: Yeah.

Interviewer: And some involve cost and have opening hours and things like that.

Respondent: Uh-huh. I mean the bush is certainly, I mean it's pretty convenient really, you don't have to drive very far up the road. I suppose it's not convenient at the end of a day when it's dark (laughs) to go bushwalking, and you shouldn't really go by yourself either, so and you know [partner] busy with his things so it's difficult to arrange for us both to have time to go for a walk.

Interviewer: Uh-huh.

Respondent: And I suppose with the sports centre if I wanted to be part of that, having strict times would be very difficult to fit in with probably with what I've got to do (laughs).

Interviewer: The sort of lifestyle that you have yeah, yeah.

Respondent: As well because every day's a bit different and you have different demands, so it's difficult to do something at a regular time.

Interviewer: Uh-huh, and that's another thing that's coming up. People sort of start to say you know with the sort of life I live, you know it's very hard to do sort of some things on a regular basis.

Respondent: Uh-huh.

Interviewer: Uh-huh so that's interesting too. Does having or not having places to be active influence whether you're active or not?

Respondent: I suppose that we've said that we've got places that I can be active but I'm still not in those areas haven't we? (Laughs).

Interviewer: So may not have a great influence on that?

Respondent: Uh-huh.

Interviewer: It's more about, you know, how much time you have really.

Respondent: Fitting it in with the rest of it.

Interviewer: Yeah, yeah and everything that you've got to do.

Respondent: Yeah.

Interviewer: Yeah, yeah.

Respondent: I mean I can't think of anything else, maybe if there was a swimming pool closer I might go swimming.

Interviewer: Might be tempting? Uh-huh.

Respondent: Yeah. Probably because I like that sort of thing you know it's something you can do by yourself and well I don't really like team sports, I've never liked team sports so I wouldn't you know I wouldn't want to go and join a team at the sports centre.

Interviewer: Uh-huh.

Respondent: And I suppose going to a swimming pool's a fairly safe sort of thing too and you could do it at odd hours as well. Early in the morning, late at night.

Interviewer: Uh-huh. And if you wanted to be more active, are there things or places that would help? I think this is the sort of thing we've been talking about.

Respondent: Uh-huh this is the answer. Yeah.

Interviewer: OK. Now destinations, if I were to ask you about places that you could walk or cycle to from your home, what sorts of things would you tell me about?

Respondent: So I could walk to the bush walks (laughs).

Interviewer: Uh-huh and that's really…

Respondent: That's pretty much it.

Interviewer: … that's the main one isn't it?

Respondent: Yeah.

Interviewer: Yeah there's no sort of services or schools or etc…

Respondent: No.

Interviewer: … friend's houses?

Respondent: No.

Interviewer: OK. And does having or not having destinations influence whether you're active or not?

Respondent: Yeah because when I lived in town I would ride my bike to the supermarket or I would walk to the supermarket…

Interviewer: Oh yeah.

Respondent: Or to band practise in town or down to the corner shop or you know to somebody along the road that I wanted to visit (laughs).

Interviewer: Uh-huh.

Respondent: Then I don't do that now, I always have to drive.

Interviewer: Uh-huh. And if you wanted to be more active would it help to have more destinations within easy walking or cycling distance?

Respondent: I suppose so; I mean the topography's always going to be a challenge (laughs).

Interviewer: (Laughs) for walking and cycling?

Respondent: In our area.

Interviewer: Yeah, yeah.

Respondent: Yeah.

Interviewer: Yeah, yeah as well as the as you said the road safety as well.

Respondent: Uh-huh.

Interviewer: OK well let's move on to neighbourhood design and what we call the built or man-made, the non-natural features of your environment. If I were to ask about those in your area what sorts of things would you tell me about? This is the footpath lighting type thing, walkability of the roads. You've already told, and you've already sort of talked a bit about that, is there any other things you'd like to talk about?

Respondent: No we don't really have anything (laughs).

Interviewer: That's basically what it comes to.

Respondent: Other than a narrow road. We've got a picnic site a couple of kilometres down the road (laughs).

Interviewer: Wow (laughs).

Respondent: But then again you wouldn't, you wouldn't walk to that because it's too dangerous.

Interviewer: Uh-huh.

Respondent: I mean you could go through the bush to it, (laughter) but it would take you a few hours to go that way.

Interviewer: (Laughs) yeah. And so if you wanted to be so… so these features do they actually influence whether you're active or not? Or the lack of them really in this case.

Respondent: Uh-huh they do. I mean if, if there were footpaths I'm sure I would, or room for pedestrians then I'm sure I would exercise more. You know if there was lighting, all that sort of thing, but also if you had those things it wouldn't be a country area…

Interviewer: It wouldn't be where you were at, no.

Respondent: At the end of the road that's nice and quiet.

Interviewer: No, no, yes.

Respondent: So it wouldn't work (laughs).

Interviewer: No.

Respondent: Because imagine if you had lighting, then you wouldn't be able to see the stars probably (laughs)…

Interviewer: That's right and it sounds like it's more the nature of as you said, a few people that sort of come along and drive quite fast and various different other things that's a bit unpredictable too.

Respondent: Uh-huh.

Interviewer: Uh-huh. And talking about safety in your area, now you've talked a bit about that in terms of traffic, is there any, and you've talked about a bit of hooning as well, is there any other things around safety either personal or road safety?

Respondent: I suppose just being in a bush area if I was to walk sort of through the bush I get nervous about, I mean we talked about hoons, but you know people in cars (laughs) you know some people go and park up in the bush or drive up in the bush and you're not really sure…

Interviewer: And you go 'hmm'…

Respondent: … what they're doing, so you wouldn't really want to be alone in that sort of situation. I'm not sure what the mobile, I think you can get reasonable mobile coverage on Mount [x] at least, so that would be OK. Nowhere… in summer I don't walk around our farm as often because I get nervous about the snakes (laughs) and I'll go weeks without having walked around the perimeter of the farm because I know that I'll trip over a snake and that makes me nervous, especially if you're at the bottom of the farm and nobody's going to hear you…

Interviewer: No.

Respondent: … from there. I think that would be the…

Interviewer: So does personal safety affect your whether you're physically active or not? As you're saying in summer that it does.

Respondent: Yeah it certainly would yep, and the same with going through the bush too. I'd worry about snakes.

Interviewer: Uh-huh and does road safety influence whether you're physically active or not? Yes you've said it does.

Respondent: Yep.

Interviewer: Yep, yep OK. And if you wanted to be more active are there personal or road safety issues that if they were addressed might help you be more active?

Respondent: I suppose if there was a lower speed limit on the gravel road because it's just an open speed limit I think. That's probably the main thing.

Interviewer: Uh-huh, slow people down a bit.

Respondent: Uh-huh.

Interviewer: Uh-huh. And is it, like you were saying, is it mainly because of this connection to the bush walks the area that people come up and are speeding, or?

Respondent: I don't think it's the bushwalkers that are speeding (laughs) they're the slower ones.

Interviewer: No, it's these other people who just happen to be… (laughs).

Respondent: Yeah you can take a really rough four wheel drive track through to [town].

Interviewer: Ahhh.

Respondent: And so I think, I understand from locals that some people will do that if they're trying to avoid police if they might have been drinking too much.

Interviewer: Ahhh, OK, so it's a bit of a short cut.

Respondent: Well it's a long cut actually.

Interviewer: Long cut.

Respondent: That's a dreadful, dreadful road.

Interviewer: So that's why, one of the reasons why.

Respondent: Yeah, and just people liking, you get some really big four wheel drives covered in mud and they just like it for the adventure I suppose.

Interviewer: Oh yeah a bit of a nice gravel road for them.

Respondent: Because it's a big rough road yeah.

Interviewer: Uh-huh. Right. OK and the last area is the aesthetics, the natural qualities.

Respondent: Uh-huh.

Interviewer: So if I were to ask you about the aesthetic qualities or the attractiveness of your area, what sorts of things would you tell me about?

Respondent: Oh it's incredibly beautiful (laughs). We're very lucky and that's what encourages you outside, you know like when I go down the back of the farm you get this amazing 360 degree view of the ocean and then to the [x] Range and then Mount [x] and Mount [x] and Mount [x] and just turn around and see the lot.

Interviewer: Wow.

Respondent: And then your farm as well in that picture and you know and then when you hone into and you go closer into the bush then you can look at the ferns and beautiful amazing man ferns and the small plants and…

Interviewer: Oh so you've got some bush as part of the farm as well?

Respondent: Yeah we've got about 15 acres of bush too.

Interviewer: Lovely.

Respondent: We hear we've got a waterfall on the property but we haven't actually found it yet (laughs).

Interviewer: Right.

Respondent: We've been there for two years and we still haven't found it. You know and you can go looking for fungi and there's plenty of things to look at.

Interviewer: And as you said you know you probably tend to be physically active in your own area it's a big enough area to explore as well.

Respondent: Uh-huh, but I mean if you go into the bushwalks, there's heaps to look at.

Interviewer: So do you think your local environment is aesthetically pleasing?

Respondent: Yes.

Interviewer: Yes. And do the aesthetics of your area influence whether you're physically active or not?

Respondent: They do because because it's so beautiful I want to get outside and enjoy it.

Interviewer: Uh-huh. Absolutely. And if you wanted to be more active are there features of your neighbour, or the area that might help if they were more aesthetically pleasing or attractive? And that's talking about you know the other side of aesthetics which is whether you have rubbish and litter or graffiti or something like that?

Respondent: No, we don't have any problems with that sort of thing so it wouldn't need to change.

Interviewer: OK, that's fine. So before we finish up is there anything else you'd like to tell me about where you're living or your physical activity or the physical environment that we haven't covered today?

Respondent: I think we've covered a fair bit haven't we?

Interviewer: Uh-huh.

Respondent: We've talked about fitting it into to life and work and farm work and family and then the physical stuff.

Interviewer: Uh-huh, Uh-huh. And I think you've been talking about like some of the real issue of living rurally in a, what attracts you to the places is living rurally but also that also means that it may not be quite as easy to just get out and go for a walk and certainly not to cycle. So it's an interesting combination, although you actually have a property which you really enjoy and are active on, other than that you'd have to get in a car and

	actually go somewhere and that's part of the nature of where the beauty of the place that you live in.
Respondent:	Uh-huh. And it's funny isn't when when we've, people in rural areas have got these beautiful places to walk and exercise and enjoy, but then the statistics aren't backing up that we're doing that.
Interviewer:	Uh-huh, either that or we don't know quite as much about it, like I don't know, I mean what's interesting and what's coming out of this study is… we might as well finish.

Date: 8 November 2011

Duration: 34 minutes, 43 seconds

Interviewer: [interviewer]

Transcriber: [trasncriber]

So today, as I mentioned, I'm really interested in your thoughts on a number of issues related to what it's like to live where you live and how that might be related to your physical activity. So as I mentioned there's no right or wrong, I'm just interested in hearing about your experiences and opinions. And as I said if there is anything you are unsure about just say so. And if you don't want to answer any of the questions for any reason, you don't have to.

OK.

So I thought we might get started by talking a little bit about the place where you live. For example what it's like to live where you are and what your property is like and that kind of thing.

Yep. Um, I live at [suburb] which is just on the eastern side of [town] near [town].

OK, yep.

The [x] Council. The area where I am is kind of um, it's undeveloped. It hasn't got any footpaths or gutters, no stormwater in the area. It was kind of someone's big farming property and it's all been subdivided. And that's pretty much it at [suburb], former farms. Houses are all on large blocks, 5 acre blocks or slightly smaller. Not

the traditional house in urban area. So bounded by the river, the ocean, and a highway.

Yeah. OK. So it's in that little pocket there.

So quite busy the highway. So you've got to struggle to get out of there if you want to go for a run or a bike ride.

Yeah. OK. Well I plan to hear about those sort of issues from you in a little bit. So the property itself that you live on, is that just the sort of standard block or are you on a bigger block?

Ah, we are on about half an acre. It was a larger property kind of split in half. There's a bit of vacant area out the back which we just use for children to run in or I feed the horse in there occasionally. Tie it up and get it ready.

And how long have you lived in [area]?

Ah we've lived there for two years.

Two years?

Yeah.

And were you from around that area before?

Yeah. I grew up in [town]. So I've lived in [town] before. Six years prior to that I lived in [city], but sort of come back up here two years ago.

And what made you decide you to live there? Is there a..

Um, really it was the location was quite [3:03] um it's nice and central and the [region]. You could work at either [city] or [city] um without too much of a commute either way. And it's close to family as well, so that's why we returned.

Yep. And how far is it for you to drive to either way to [city] or [city]?

Ah, at the moment to get to [city] it's only 12 minutes. And [city] would be a little longer, probably 25 minutes.

Yep. So pretty convenient. And is that where you would do you know your shopping and your chores and things?

Ah it's usually probably at [town], for day shopping or yeah, [city]. Sometimes we go to [city]. But mostly [city].

Sure. OK. When we've doing some work with people that live in larger cities I guess, they often refer to their local neighbourhood. And I'm just interested in knowing whether the term neighbourhood means something to you and if so, what?

Um, not really. There's not much of a sense of ah, community in [town]. Everyone sort of keeps to themselves a bit I think. My neighbourhood is pretty quiet really. There's a Neighbourhood Watch, but I think that's about to fold. Um, in [suburb] because there are the bigger blocks, no-one is really sort of over each other's fences, so to speak.

So could you put a distance on something like neighbourhood or is it a bit hard to quantify?

A distance?

Yeah.

Yeah, probably a bit hard to quantify. I guess it's, you know, keeps to themselves. The neighbourhood itself is in an area, it would just be that area that I sort of described that's bounded by the sea, the highway and the river. Um, everyone, people there I guess refer to that as lower [town]. Because it's on one side of the highway as opposed to the other, which is still [town]. The two sides don't get together very often, I don't suggest.

Yeah, OK. And you mentioned that sense of community. Is neighbourhood different to community or is it the same thing essentially?

Ah, to me it's the same thing, I think. Um, yeah I would say it's pretty much the same thing, like for a lot of people, yeah.

And so in other places where you've lived have you felt more of a, sort of that you are in a neighbourhood or a you know, a greater sense of community? Or is it not much different.

Oh yeah, well when we lived in [city] we lived at [suburb], and we had a street that was an interesting street in the way it was shaped. It was dead end at each end and you sort of entered it in the middle. So it was two cul de sacs, so everyone sort of knew everyone in there, and kept an eye out for each other. You don't get that sense at [town], really.

Bit more isolated.

Yes.

Alright. I'm going to have a talk to you a bit about um physical activity and what that sort of means to you and what you do. I'll sort of probably break down your day a little bit and think about different parts of your life and that kind of thing. Just so we

can cover off on everything. So can you tell me what does the term "physical activity" mean to you?

Um, it means my health and well being and um being free to move. I do a fair bit of running. I like to keep fit for work, so a do bushwalking, running, bike riding and all that. And I just like to be an outdoor sort of person. So, um important to me to be fit and healthy.

Yes. Yep. So you consider yourself pretty active?

Yeah.

Yep. And in terms of the fit, where does physical activity sort of fit into your life. Is it sort of a priority or um, something that you just do or…

Um it's not a priority. It's something I do when I can I suppose. You can't always get home and go for a run. But if I can manage it I will. Um, with the kids and everything else that goes on it's a bit difficult at times.

Yep. How many kids do you have again?

Ah, we've got two children.

And they're at primary school? Oh, they're little..

Oh no they're two and four.

That's right. OK. So I guess sort of the definition that we kind of use in our research is um, for physical activity, is any activity that lasts for at least ten minutes. And it causes your body to work a bit harder than normal. So your heart rate goes up, you might huff and puff a little bit and you get a bit warm. So thinking about that sort of definition I guess, in the last week or two, have you been active at all at work?

Um, yes, have been, yes.

Yep. Do you want to tell me a bit about that?

Um, one of my roles is as part of [job]. So whenever that happens I'm very active… So, and we were waiting around so that was a fairly strenuous day. Um, and my primary role is in the [workplace]. So of late we've spent a fair bit of time in the bush, walking around looking for whatever. So the last few weeks actually have been fairly busy at work.

Fairly active at work. So things like your search and rescue, does that happen very often? Is that sort of regular or just every now and again?

Ah we probably get called out, well just recently it's been fairly busy, but usually you'd say we seem to get a call out every couple of weeks or so, which is a job where we have to go walking or doing something, climbing or – yeah, every couple of weeks or so there seems to be a job which involves getting out there and being fairly active.

Yes. And with the other work, with the [workplace] when you are out, is that just walking for long periods of time?

Ah yeah, generally walking.

OK. And does this, I mean this sounds like a bit of a funny question maybe, given the work that you do, but does it change at all depending on the season or the time of year?

Oh it does. Yeah because at the moment the crops are in and we're out there looking for them.

Of course.

Yeah, so from that regard it's seasonal. Um, but that can happen at any time of year, if we searched a rural property or um for items or whatever. So, yeah.

Yep. So it wouldn't matter if it was the middle of winter or anything, it's not going to put you off is it?

But definitely over the summer months we spend more, or we do get quite active out in the field.

And are there times when your job is desk based? Do you do a bit of office work as well?

I do. Yeah. I'm the [job] here so I have a fair component which is administrative. So I can probably spend, like easily spend a couple of days a week tied up in the office if I have to.

Yep. OK. And I'll ask this question, which it sounds like it might not be relevant to you, but you can tell me that yourself, if you'd wanted to is there anything that would have helped you to be more active at work?

Um, as in plain program or..

Um, I guess just as part of your everyday job? If you'd wanted to be more active, is there something that would have helped you in doing that?

Um, no. Because I could always transfer to another area I suppose. Um, but the one I'm in is fairly active. I could go somewhere where maybe you were doing a lot more

walking. In [city], on the [x] coast there aren't sort of … as opposed to [city] or [city]. At this point in my career, that probably would be a backward step.

Yep. So being more active, would mean changing essentially jobs and roles and things. Yep. So it's not ..

No, you sort of go on your career path. I'm trying to go towards more desk based administrative based role as I move forward. Tend to be less in the field and more in your office.

Yes, I think that happens in a lot of jobs. The more senior you get the more sitting you do. Alright. So OK, we've had a bit of a chat about work. And now I want to talk a little bit about what we think of as sort of active commuting. So have you walked or cycled for at least ten minutes at a time to get to or from places in the last week or two?

Um, I have. Well yesterday, was it yesterday – the day before yesterday - we went on a long bike ride, just going to [town].

OK. How long did that take you?

Ah that takes, oh I suppose we rode all up for an hour. Part leisure, part bike ride I guess. A purposeful journey but also pleasure, riding around.

So it was a bit of both.

Bit of both, yep. Um, but I don't ride to work, on the highway. And I haven't ridden at work or anything like that. So no.

Not really, OK.

No, not really.

Yep. And um would that change depending on the time of year or is that pretty typical?

No, it's just the highway is too difficult, too dangerous to ride on. I wouldn't – and if I did it would be in the summer months, it wouldn't be in the winter months.

OK. So that highway is essential. You've got to go on the highway if you want to go anywhere?

You do. Yeah.

There's no bike – there's no path up your way yet?

No, there is um, from [town] to [town] there is an excellent bike path. But then it stops.

*Yeah, it stops at [*town*] doesn't it?*

And then you've got the [village] River. So to get to my place to the bike track you've got to cross the bridge along the highway, which is, I have done that, but I wouldn't take my children. We load the bikes in the car and then we get out at the bike track and then go from there.

Yep. No that makes sense. And so I guess I'll ask that question again, if you'd wanted to is there anything that would have helped you to use more active forms of transport?

Um, no. I don't think there is anything that could have helped me. No, there's no public transport to take.

What about, is there some sort of solution you could see with the highway if you wanted to say walk or cycle to work?

No, I'd have to ride too. There's a different route but that would be through [village] and that would just take too long.

Yep. OK. No problem. Alright, now we're going to think a bit about things things that you do around the house and the yard. Um, so in the last week or so have you been active around the house or yard for at least ten minutes at a time?

Ah, yes I've mowed the lawns twice.

Oh wow.

It growing so fast.

It's like that at the moment isn't it?

It's out of control. That takes about an hour each time.

OK. Yep. And you do it, you've got the push mower.

Yes. I'm about to get a ride on though.

Are you?

So I can do it quicker.

You need it at this time of year. So that's about an hour's duration each time?

Yep.

And how sort of hard would you rate that? Is that sort of something that's moderate or really vigorous?

Ah no, just moderate.

Moderate. Yep.

Yeah.

And when do you sort of fit that in? Is that an after work job or a weekend job?

Um, it's a little bit of both. If it's a fine afternoon when I get home I'll do it after work. Or part do it. Otherwise it's just a weekend thing.

Yep. Yep. And do you do that, is that something you just do on your own or have you got some helpers?

No, I just do that by myself.

Just do that yourself. And that I'm guessing changes a bit depending on the time of year?

Yes. Yes. Especially at the moment.

Yep. And are there other, anything else around the house or the yard that you tend to do, in the last week or two?

Um, no just like I've got a motor bike that I do a bit of work on, and a car that I'm repairing for my brother in law. That's hobby type stuff I suppose you could say.

Yep, so a bit of tinkering.

Yep.

OK. And if you'd wanted to, was there anything that would have helped you be more active in the home or around the yard?

Um, only time I suppose. Yeah.

Yeah, if only we could bottle that, yeah we'd be doing well.

Yeah that's right.

OK. Now the last sort of area of physical activity that I'm going to ask about is what you do during your leisure time? So you touched on before, you went for a ride with the family. But is there anything that you have sort of done for at least ten minutes at a time, um physical activity in your spare time?

Yeah. Motor bike riding is something I do fairly regularly.

OK. So how often would you do that?

Um I try and do it once a week, every weekend. But realistically it's probably once a fortnight.

Yeah. OK. And how long do you get out for?

Um, we go usually for probably two or three hours.

OK. And I've never done, is that sort of, is that quite hard work?

Ah yes. It's very hard work, yes. Off road riding as opposed to road riding.

Yeah, OK. And so you do that sort of an weekends?

Yes, or if I have a day off during the week I'll go. Like the last few weekends.

And where do you go to do that? Like is that local?

Um, you can go, we go through the [x] range which is behind [town]. Or further afield [17:26] or [town] which is nearby [town] or towards the [x] Coast.

OK. So you've got to drive somewhere.

Yep.

And usually within an hour or so's drive, is that..

Ah, yeah it'd be – um, sorry down the west would be the furthest we'd go and that is probably a bit over an hour. But you could say in an hour, yeah.

Do you go on your own or with other people?

Ah usually with other people.

Yep. And does that change depending on what time of year it is?

Um, no not really. We quite enjoy it as much in the winter as we do in the summer. We may do it a little bit more in the summer because um when we go to [region] we camp. But probably we go a bit more in summer months, but we still don't mind it in the winter.

And you mentioned before you do a little bit of running? Is that ..

Yeah.

Yep. Do you want to tell me a bit about that?

I probably, I try and run at least twice a week. Usually around ten kilometres. I have a running machine at home now. But that's one thing. With running and little children, it was easier to um get a running machine sort of thing, you don't actually have to leave the house.

Yes. That's one thing I was going to ask, where you go or do you do it at home?

Yeah, so um my wife uses it more than I do. I still like to go for a run. But I sort of run within a 10 km radius of where we live. I'll cross the bridge and run along the running track, or on the beaches.

Yep. Yep. So there's somewhere for you to go that's pretty, that's nearby, you can go straight from home.

Yeah, it's excellent for running. More so than anything else probably, where we are.

Yep. And do you tend to go on your own?

Ah, yes.

Yeah, and was there anything else you sort of do in your spare time?

Um, I like to go bushwalking as well. For training I guess, keeping fit for that. But ah, I probably don't get to do that as often as I'd like. But when I can I'll go.

And what do you just sort of head out for a couple of hours?

Yes just an afternoon. I'll just go out for a couple of hours in the [x] Range again, behind [town]. Um, if I've got a few days off and can manage I'll go for a fairly extensive walk somewhere, for a few days.

Yeah. OK. And if you'd wanted to, is there anything that would have helped you be more active in your spare time?

Um, probably again it's just a time management thing I suppose. If I had the time to sit down and plan a proper time, I would. But then the work always messes that up. That happens. Yeah, but that's all really.

More time.

Yeah, more time.

Yep. Alrighty. Sounds like lots of people that we've spoken too.

Oh yeah, time poor.

Yep. Alright. So we talked a bit about where you live and your physical activities. And now I'm really interested to hear a bit about the physical environment. Where you sort of live and work. So when I talk about that I sort of mean all the things that sort of surround you, you know the roads, the trees, the houses, shops, traffic and all that kind of thing. And these are all things that people living in cities have told us are important for their physical activity. So I guess I'm interested to hear from people that are living outside of cities um whether that's the same for them. So do you want to tell me a bit about you know, your physical environment.

Yep. Well where we are at [suburb] is, what I really like about it is the beaches. Um, there's like huge wide open spaces, like when you get out at low tide at [town] and there really isn't anyone else out there. So quite often you can be the only person on it when you're running. Um, but that's where I live, that's the best part about being able to go for a run up here. Um the new bike track that's been put in is really good too. And I can take my children on it without too many worries. The biggest

thing that impacts getting on the bikes and going from home is being bounded by the highway I suppose, the dual carriage way. So that's a, I mean I'm happy to run along, a short run along the side of it to go for a jog, but bike riding is out of the question.

So is there a bit of a sort of a shoulder on there at all? Or not?

Yeah, not much. It would be great if they extended the bike, just kept the bikeway going from [town] to [city], which is only another 10 or so ks. So that would be good, it could follow the train line.

Do you know if there's any plans to do that?

Oh there's a fairly long term plan to take it all the way to [town] I think. I don't know whether that'll happen. That's a long way away. Um, so yeah it's kind of the seaside environment that I like of where we live. Um, and you have [suburb] itself is kind of sparsely populated I suppose. You don't have a sense of like being in real urban environment, where it's sort of crammed in. You feel like you've got a bit of privacy and space around you. It's good.

Yeah, sure. It sounds very nice.

Yeah. And workwise, well we've got a pretty dilapidated old police station here in [city], so um. And [city], I mean sometimes I'll go for a run from here after work, because they've got nice bikeways all around to [23:05] so it's not too bad here.

And would you do that very often? The sort of run after work or from work?

Oh it comes in sort of stops and starts. I get a bit excited when I start going running again and then it drops off a bit. So I've more so run from home than from here. But every now and then I'll bring my gear in and go for a run when I knock off.

Yep. OK. Um, so if I was to ask you about, um you've touched on this a little bit, but places to be active in your sort of neighbourhood or area. Like things like, you've talked a bit about walking tracks and cycle paths and the beach, but what about things like sports clubs or recreation facilities? Do you have access to that kind of thing?

Um, no not really. There's nothing in immediately where I live. And in sort of the whole [suburb], [town], there is only a lawn bowls club. Which tends to be more a social club for drinking. There's a football club but I don't play football, so.

Sure. Is that, that's in Leith?

Ah, the [town] football club. And then there's one at [village], but if you're not into that then that's the end of that. Then yeah, but other than that there's [city], to find a golf club or – I used to play golf, I don't any more.

And are there any gyms or sort of aquatic centres or anything like that around?

Oh there's a – well it's not that far from where I live I suppose, [city] Aquatic Centre. Um, but that's only open in the summer months, because it's an open pool, it's not closed in. There's talk that they are building a new one which would be around two or three years away. So that's somewhere I would go if it was more sort of reliable.

Yeah. OK.

I actually have friends with an indoor pool that we go and swim occasionally. So that's something that if the big pool complex kicked off, was finally built, then I would probably go there for a swim more regularly.

Would that be for you or would you go with your family?

Oh probably the family would all go I reckon. I would probably swim before work even. But we would definitely go out as a family group regularly. Yeah. Um, and there's some gyms. I'm not really a gym person I suppose. A couple of those new gyms in town. One of them is a 24 hour gym, so you could get into that if you wanted.

So they're there, but um not necessarily your cup of tea.

No. No. I don't worry about that, so.

Yeah. OK. And so um, do you think sort of having or not having these places influences whether you are active or not?

Um, probably to a degree it does. Because it sort of sets, if they are here, if I had started when I was younger, than I would be that way inclined perhaps. Probably more involved. But it's hard to sort of get – you know I guess as a junior and sort of get that attachment to it that keeps you going through life. And a bit[26:17] on the [x] Coast.

Yep. No that makes sense. And if you had wanted to be more active are there things or places that would help you if they were more readily available, or not really?

Um I could join the gym and get on the program there. They have programs, they encourage you through work to be active. So I think there is discount for police to be

a member there. Um, other than that, not really. There's not a lot going on where you could just go and join it and away you go.

Yep. Yep. Too easy. OK. Um, what about, you've sort of touched on this a bit already, I was going to ask you about sort of places that you can get to, um walking or cycling from your home. Um, but it sounds like there's not too many places you can get to from home on foot or bike, is that right?

Yeah. That's probably right. But yeah, for me it's more I guess it's cos we live at [suburb]. Other people might not have that problem, but we're a little bit limited for that, in that regard.

And so does sort of you know not really having those, I guess destinations nearby, does that influence whether you are active or not?

Um, I guess it does. Yeah. I think if the bike track went past our house I'm sure we'd use it all the time. But it's just that you've got to load the bikes in the car and pack it all up and go. And that probably, you don't go as often as you would.

Yeah. OK. And I just wanted to ask you as well a bit about the sort of the um, the built or manmade features of your area. So I guess here I'm thinking about you know footpaths and streets and lighting and that kind of thing. What's it like around your area?

Um, we don't have any footpaths, very little lighting.

OK. So neither.

No gutters, nothing like that at all. People come and say "oh it's like having modern houses but in a beach shack environment". So it's kind of got that feel about it.

Yeah, right. So that sort of adds to the sort of the feel of the place?

It does. Yeah. Yeah.

Yeah. OK. And do those sorts of things make any difference to whether you are active or not?

Um, well no. The traffic flow in [suburb] is pretty low, so um you can easily go for a walk of an evening if you want to go. Have tea and go for a walk. People walk there all the time, around in [suburb], walking their dogs.

Yep. And not having footpaths isn't an issue because ..

Yeah, it's just kind of doing a big loop around [suburb].

Yeah. OK. Alright. And what about the um, the sort of aesthetic qualities or attractiveness of your sort of area? You know what sort of things would you tell me about? Is it a nice sort of place visually?

Yes. Yeah. It's quite tidy. Most people take pride in their houses and the neighbourhood. Um, and of course with the beach and the coastal features it makes it quite, quite nice. Especially on a nice day. The [village] River comes out, that's always nice down there. I generally, it's quite, quite good in the area.

Quite pretty. And does that sort of influence whether you are active or not? How attractive the area is? Or not really.

Yeah, to me it does. I like to walk or run somewhere that is a nice place to run. I like to run on the beach. That's what I really like, getting out on the open beach and jogging along. So yeah.

Yep, that sounds lovely. Makes me wish I was out there and maybe not at work. Alright. One last area I wanted to touch on was safety. Um, what sorts of things would you tell me about if I was to ask you about safety in your area?

Um, crime rate is low.

Is it?

I think people feel safe when they go for a walk. Yep. Um, the again to go back to the highway again, that's not safe, but um to get across it- traffic is generally what you'd expect on a dual lane highway I suppose.

What's the speed limit through there?

Ah, 100. 110 out on the highway. 50 in [suburb]. Um, we don't get a lot of through traffic so it's generally, it's quiet. You can walk on the road no problem. The river mouth is a little bit dangerous, plenty of warning signs about not swimming in at the river mouth itself because of the currents. Kind of common sense thing. But the beaches are perfectly safe. You can wade and walk around in that water no dramas. I swim over at [town]. Um, yeah that's pretty it. With no hotels or anything like that there you don't sort of get any of those troubles.

Yeah. OK. So not really issues around violence and that kind of thing.

No. Probably good

Yeah. OK. Um, and I think again you've touched on this pretty well, but I'll ask it anyway just in case there is something else you wanted to add, if you wanted to be

more active in your area are there any personal or road safety issues that could be addressed?

Um, no. Probably [31:48] I guess. Yeah, just be get people off the highway more so by extending the bike track I think, that would be the main thing.

Yep. That sounds key, sounds very important up there. OK. So that's pretty much all my questions. I guess before I finish up is there anything else that you'd sort of like to tell me about that you think I might like to hear or that we haven't covered today.

Um, no I think we've done fairly well. I think in [town] itself, that's well set up. I guess they've amalgamated the councils between [town] and [town] and um [town]. But I guess that's going to have an impact on it as well. Because [town] does seem well set up – it's the sort of outer lying areas that are a bit behind. But slowly they're catching up. Yeah that's probably about it really.

Yeah. So that might have an impact on some of the things that are going on locally.

Yeah. I just think in an area like the [x] coast where the population is a bit more spread out than say [city], you've just got to travel more if you want to be involved in sporting clubs. And so that makes it a bit harder for communities, harder to get there after school to your basketball club or whatever, tennis or…but the transport, public transport is pretty poor.

Yeah, I mean what is it, just bus up there? Is that the main..

Well yeah, there's buses. But there's Metro operates in [city] and then there's a private company in [city]. The coast isn't linked up very well. You'd struggle to find a bus to get from [city] to [city] I'd say. It would be maybe once or twice a day and probably not at the best of hours.

And you mentioned that people sort of have to go somewhere to do those sort of more organised sports, is that, do you think that is something that's seen – does that just become the norm then or is that still seen as a burden?

Um, as in the need to travel?

Yeah, that idea that you've got to go somewhere else to be ..

Yeah, I know just from growing up, like if you wanted to be involved in tennis - I played tennis and basketball and things – if you wanted to be involved in a higher level in competition, you sort of had to travel anywhere from [city] to [town] or [town] to [town]. It does become a bit of a burden in the travel. I know you can be

easily involved in a sporting club in [city], like tennis, and you've just got to get to the [facility] and play all the tennis you like and then go home. Up here the actual facilities are fairly, apart from [city] and [city], the facilities are fairly poor. And you've got to travel a long way to get between them.

OK. Alright. Thanks so much for the chat.

That's OK.

I'll just turn this recorder off.

AUDIO RECORDING ENDS.

Date:	11 October 2011
Duration:	41 minutes, 18 seconds
Interviewer:	[interviewer]
Transcriber:	[transcriber]

OK so I thought I'd start with a bit about the place where you live. Could you tell me a bit about where you live and what it's like to live there and your likes and dislikes really.

OK. Yes. Live in [town]. We've been there about 12 years. I live approximately, about 200 metres from the river and about 200 metres from the beach. And the parks are all nice and flat. So it certainly lends itself to a healthy lifestyle. [town] has an abundance of sporting things available. All sorts of sports. It's always been a good place for children to play team sports because it's all so central. They can walk to just about any of the things, to the basketball, to the soccer, to the hockey, it's always within walking distance. So it's always been very good for that. Um –

That's really good.

Yeah. They've just done a new walking track from [town] through to [town] about 12 months ago. And that has certainly um been a welcome addition as well, the bike and running track and walking track. So it joins [town] and [town], which was a initiative of the Council I believe. Which is being utilised really well.

Yes, it's interesting. All of the people I've been talking to. Because we've been talking to men who live in rural areas in this study, and earlier in the year, the women. And everybody has been mentioning this bike track, the walk track, has been a really good addition to the existing walking tracks and parks and through [town] area.

It is. I think people, a) they feel safe, they're not you know crossing roads and the traffic. And it takes away a bit of the self consciousness of some people. Say if they are a little bit overweight. They are not worrying about what people think when they are walking up the footpath and stuff there. Because the other people that are on the track are walkers and that as well.

Yeah, everybody is doing the same thing.

Yeah, I think it takes away a bit of that. And they can walk with their kids on bikes and things like that. So that is very good.

So you live in a very beautiful spot. Is it a standard house block, pretty well.

Yes, it's a standard house block.

And how long, you mentioned how long you've been living there – just remind me again.

Ah it's 12 years.

12 years. Yep. And what were your reasons for living there.

Ah I transferred in my job from [city] back to the [region]. And I was brought up in [city] next to the, being near the water and then lived in [city] for five years with my work and realised that um what I missed was being near the water. So when we moved back to the [region], I didn't care where we lived as long as I could see the water and was in walking distance to the sea and the beach and stuff. So that was my only prerequisites when we came back to the [region]. And my wife worked at [workplace] and I was working at [city], so [town] just sat nicely in the middle.

Yes. And it's a very beautiful spot. I was up there a few weeks ago, and that particular part of [town] is really very beautiful and very well appointed. So you decided very well.

Yeah, I was very happy with the choice. A couple of hundred metres from the river. And I've got a surf ski, so I just carry that down to the river and go for a paddle. So it's very stress relieving from the work. It's nice.

Yeah. Absolutely. That's important. Now how far are you out of the centre of town.

Um, again only probably from the shopping centres, shopping centre and main street, half a kilometre. We are just, do you know where the football oval is? Or the yacht club?

Mm, I know where the yacht club is. Yep.

So just up from the yacht club. The street just up from there. So it's walking distance. You can walk into town and grab your groceries and get a coffee or whatever. No need to move the car at all. It's all flat, so it's good.

Sounds good. Now I'll just go onto talking a bit more about your local environment. So as we've said the research has been mainly done in urban areas and people often refer to their local neighbourhood and we are interested in understanding in what the term neighbourhood means to men who live in rural areas and indeed if it's relevant. So what does the word neighbourhood mean to you?

Um, well certainly when we moved it was very welcoming. Because the person who lived directly across the road was from the local surf club. And as a result of that my two kids and our family got involved in the surf club. And they've been in that ever since and I was there for a number of years. So the neighbour was welcoming of people, was more so than anything else, was the neighbourhood.

And so if you needed to put a distance around that, how big a distance would you call your neighbourhood?

Oh I would probably say um, a kilometre radius, probably. Just thinking of the size of the couple of blocks that we walk the dog around, stop and chat to people and yeah, so a couple of blocks probably. Yeah.

And what about the word community, what does that mean to you?

Um, people saying hello in the street, um and offering to help. And stopping and caring about people, when they drop something picking it up for them, not just turning the other way. Ah yeah, and community you know your Lions Clubs and things like that. So regular community events I suppose, put on by not for profit organisations. People not in it for themselves is probably the sense of community.

Mm. That's really clear, thank you. Um, and if you needed to put a distance around it um, how far would community be?

I think probably I would probably call [town] the community. Yeah, the whole of [town]. More so probably the east side rather than the west.

So that would be a few kilometres about what 5-10.

Yeah probably a 10 kilometre radius, that would include the town, yeah.

Great. Now we are going to talk a bit about physical activity, we'll come back to some of the things you have been talking about. And as I was saying it's very broad

and it really depends on the individual. So we are interested in well what does the term physical activity mean for you?

Um, regular exercise. Yeah, so any exercise I'd put down as physical activity, whether that be individual or team.

And do you consider yourself physically active?

Ah yes.

Yeah. And why so?

Um, probably I'm at the upper end. I'm a runner, I run marathons, and ah keep train every day sort of thing between running. And other sports. So I'm – 47 nearly 48, I'm at that end of the scale. You probably – I'm hoping you get people from the other end of the scale –

Oh yes, we certainly do.

Because it's good because –

Yeah, you need a range of people because you need to –

Otherwise you got a false feeling about –

Yeah and what works for some people is different for others so it's good to get the range. And um, where does physical activity fit in your life? Is it of no interest, or is it a priority or a high priority?

Um, certainly a high priority. Yep.

Yep. OK.

It ranges from the physical activities but it's the well being and the stress well being that um, that it fits into, the work life balance type thing. That it –

Yeah, that's really important isn't it?

Yes.

And people say that. It just really clears the stress and the day out of your system.

Oh yeah –

And that whole stuff of feeling just a lot better in yourself yeah. That well being feeling.

Yeah. Exactly. It just clears the head.

Yeah. Absolutely. So just going to go into the physical activity side of it a bit more. In our research when we think about physical activity we think of it as any activity that lasts for at least ten minutes, which is the evidence of health benefit effect, and

causes your body to work harder than normal. So, as you would well know, your heart rate might go up, you might be breathing a little heavier and you might warm up a bit. So thinking about this type of physical activity during the past two weeks, I'm going to go through different parts of your life, not to segment them, but more that we don't leave things out.

OK.

OK. So at work in the past two weeks have you been active for at least ten minutes at a time?

Yes.

And can you tell me about what you did?

Um, we actually do a core strength pilates class twice a week, um beginning of work, half a dozen of us. So we do that twice a week.

Wow. Is that part of work or something you've organised together.

Ah, something that myself and my 2IC organised. He's a fitness instructor and we encourage people to come along and get involved. So lead by example, that sort of thing.

That's great, that's really good now –

So we do –

So thinking about the intensity of that – and are there any other activities you do during work?

Ah probably during work apart from using the stairs probably no. I'm pretty well desk bound and –

Yeah, which is the case for a lot of people now isn't it? And also that balance of time and work that you are doing is always a bit difficult. Now thinking about that activity that you do do though, at work. Um, the intensity of the pilates, did you say pilates and –

Yeah a bit of pilates and core strength training, yep.

Yep. So the intensity of that activity. Would you call it steady, moderate or vigorous?

Um, yeah probably between moderate and vigorous. So between there.

Yep. And how long would you do it for at a time?

Ah, thirty minute sessions.

And how often?

Twice a week.

Yep. OK. And you were saying it's before work. Is that what you usually do? Or is it a one off?

Ah no, that's what we've been doing for nearly 12 months now. So, yeah.

Oh, wow. Does this change depending on the season or time of year?

Um, no not the season or time of year. But there may be some emergencies that come up for some reason that we'll miss one for whatever reason.

That's more about the nature of work.

Yeah, rather than the seasons or the um environment.

Now and if you had wanted to is there anything that would have helped you be more active at work?

Um, more time. Yeah, that's probably all it is. You know being able to allocate more time to do it. Yeah, it's probably the only thing that prevents us from doing more. We've got a small gymnasium that's enough to do a couple of things but we could probably do with more equipment. It's more about more time to be able to do stuff.

Is your work actually based in [city]?

Yeah.

OK. Now, in the past two weeks have you walked or cycled for at least ten minutes at a time to get to or from places?

Um, walked I have, yes.

And can you tell me about what you did?

Um mainly up to the shops to get a few groceries and bits and pieces. That's my walking. Or to meetings in town. Probably um trying to think, probably half a dozen times a week.

Yep, yep. So half a dozen times a week. Would you call the intensity of that exercise steady, moderate or vigorous?

Ah, yeah moderate. Or to the lower side of moderate. Just walking, yeah.

And how often might you do that?

Probably about six times a week. Yeah, with meetings and up to the shops.

Yep. And um, what time of the day would you have done that? Some of that would have been in work time so –

Some in work time and some after work or weekends.

Yep. And you said that might go up to the shops or walk to meetings, so that's obviously because you, yeah OK – and were you alone or with others when you did those things?

A combination, combination. Yes, so shopping sometimes, quite often my wife will walk with me. But meetings it depends.

And is this what you usually do or is it one off?

Ah no, certainly usually wherever I can, yes.

And does this change depending on the season or the time of the year?

Ah yes. I'd say it does a little bit. Yes because if it's the middle of winter and it's raining, yeah you might take the car or get dropped off. So yes, that one does.

Yes, it's not much fun walking in the rain.

No, not if you've got an arm full of paperwork and stuff.

No. And if you had wanted to is there anything that would have helped you walk more often to or from places?

Um, probably not.

Because you do that anyway, regularly.

A warmer temperature always encourages walking. So maybe the seasonal thing but apart from that probably – oh and probably the time to do it again. It's quicker to be dropped off in a car than to spend the 15 minutes to walk there. So for work purposes again it's just the time constraints.

So in the past two weeks have you been active around the house or yard. So at home either inside or outside for at least ten minutes?

Ah yes.

And could you tell me about what you did?

Pretty much all it would have been is mowing the lawn once, which takes about half an hour. And another stint of gardening for half an hour. So just the two in the last two weeks.

OK. So that would be sort of um, two activities and the intensity of those would have been …

Oh yeah just moderate.

Yep. And how often would you have done those two activities?

Ah, only once, once each in the last two weeks.

Yep. Yep. And when would you have done that?

Um, during the weekend, during the day during the weekend.

And um, who would you have done that with? Would you have done that by yourself or with others?

Ah with my wife. So with others.

And is this what you usually do? Or is it one off?

Yes. It's the usual.

And does it change depending on the season?

Ah yes it does. Obviously with um over winter it gets a lot less. Grass doesn't grow and you can't get out there. But now on it's very regular.

Spring onwards yeah. It's a bit full on in the garden. Everything grows. Now have you been physically active for at least ten minutes during your leisure or spare time? So this picks up any other activity that we haven't talked about, like your running.

Yes.

OK. And any other activity other than your running?

Yeah. I play basketball, so I've got a social basketball team.

And the surf ski on the river?

Oh the surf ski on the river, yes.

OK. So thinking about those three activities, ah would you call them steady, moderate or vigorous.

Ah vigorous.

And um how long would you have done those activities, just roughly, at a time?

Um, the basketball was one game a week of an hour. Um and probably at the moment only one paddle a week of an hour each. And run, I run pretty much every day between 30 minutes and an hour.

OK. And looking at when those activities were? The basketball sounds like it's one evening.

Yep, that's an evening.

And the um surf ski, the paddle would be what, the weekend?

Weekend.

Yep and the running is every day. Yep.

Is evening and weekend.

And where the basketball, is that local in [town]?

Ah I play at [city].

Oh, OK. OK. Right. And obviously the surf ski is with the river, next to where you live. And um the running that would be local would it?

Yes. Just from home, usually the tracks and roads.

Um, and the activities are there any activities that you would do by yourself? Like obviously the basketball you play in a team.

Yeah, the other two are by myself.

Yep. OK. And is this what you usually do or is it one off?

Ah usual.

Yep. And does it change depending on the season or the time of year?

Ah yes. Again, during the winter the paddling gets less and the running may go indoors occasionally. But apart from that it's the same.

And if you'd wanted to, is there anything that would have helped you be more active in your leisure or spare time? It sounds like you are pretty active already, but I'll ask the question.

Yeah, probably not. More time.

Absolutely. Now one last section is the physical environment. And we've talked a bit about where you live and your physical activity. So now we want to talk about the physical environment where you live, work and play. So when I talk about that I'll be talking about the physical things that surround you. So it might be the roads, the trees, the parks, shops, traffic. And some of these things are things that people living in urban areas have said may be important for their physical activity. So we're interested in asking whether they are relevant for you. So the first question is just a general question about can you tell me a bit about how easy or difficult it is to be physically active in your area?

Um, very easy. There's good parks and running tracks within 100 metres of where I live. Nice and flat. It's safe, it's away from roads. And there's grass or tracks. There's also the beach. And I run on the beach. So you can also, there's room to play a game of cricket or you can kick a football. And there's also really good facilities, as I said the basketball stadium is only just across the river. There's all sorts of – so I'd say yes, it's very easy to be.

Yes, and what makes it easy is that you have got a lot of choice but it is actually also very accessible.

Oh it is. Within probably five minute walk, at least within a kilometre of home, there's the basketball, there's the surf club, there's the football ground, there's the hockey ground and a couple of gymnasiums. So that access, and all flat and easy to walk to. And safe for kids to go to is a real asset.

So that's one of the areas that we are covering is availability and accessibility. So um, you know we've spoken about the availability being very good. The access, are they places, are they convenient and easy to access, like their cost and opening hours for instance?

Yeah, the access is good. Um, cost for me is not prohibitive. I can understand how some of these things can become prohibitive for struggling families, to pay registrations and entry fees and you know sandshoes and things like that. So whilst it's not for me I can understand how it can become prohibitive for families.

No that's a really good point. It can add up can't it? Yeah.

Yeah, two or three children and they always want the best and the latest gear. That sort of pressure that is on the parents to provide. I can understand it could be a bit, but for me it's not.

And this section is a bit broader anyway. It's yourself and also a bit about, you know, a comment about your community and what you know about it. So does having places to be active influence whether you are active or not?

Ah absolutely, yeah. The ease of access to the places is um, encourages it yes. And I found that from when I lived in [city]. I was living out of town and you had to, apart from you know you can run from your background but to go to anything else you had to get in the car and drive to a pool or to an area to go swimming or to a track for cycling or anything else like that. Take basketball, had to be in the car and drive; to where we are now, it's just so accessible, so there's certainly that benefit.

Mm. And that's really important. If you wanted to be more active are there things or places that would help if they were available or more accessible?

Oh no, I don't think so. I think I'm going OK.

Yeah, absolutely.

With what I have in my backyard, I think I'm going OK, yeah.

OK. Another area is destinations. If I were to ask you about places you could walk - or for instance cycle, but walk in your case because you haven't mentioned that you get on your bike – um to and from your home, what sorts of things would you tell me about?

Um, there's the park that used to be the old golf course and so it's um follows the river first and then along the beach. And that continues on all the way to [town], and so we have a very pleasant walking environment and a safe walking environment for that purpose. Plus a nice easy walk to town. So you can, you don't have to take, you can carry a couple of bags of groceries quite easily with walking. So that's a benefit as well. You can just use that ten minute walk to get your groceries. Again, the accessibility where it's not too far away, where you would get in the car to do..

Yeah, no that's good. So does having destinations influence whether you are active or not?

Ah yes, I think so. Um certainly with the walking, if I've got to go and pick up something for instance from the supermarket, um it's a destination that I've got to go to and there is simply the choice of whether I get in the car or walk. So otherwise yeah I wouldn't go. If it's a destination or a meeting or something like that then yeah, it's simply just a choice of how to get there and if I can walk do it that way.

Um, and if you wanted to be more active would it help if there were more destinations within easy walking distance? It sounds like with [town] everything is fairly close anyway.

Yeah, I think we are pretty lucky where I'm living. Everything that I need to get to is pretty well walking distance.

And it does make a difference doesn't it? It does give you choice.

It does make a difference. If it is that extra, if it is double that distance then you start to think oh, well really it is going to take my three quarters of an hour and I really haven't got that time. But if it is going to take twenty minutes then that's OK. You can slip up, ten minutes and ten minutes back, so yeah you can.

OK. Neighbourhood function and design. So if I were to ask you about the built or the man-made features of your area, what sorts of things might you tell me about? And you've already started talking to me about how [town] is very walkable. Um, but it is also about you know footpaths and lighting and whether the streets connect or not.

You know that you can actually have a reasonable walk. You know, some people in rural areas just live on a main road and it's a lot harder to just have a good walk there.

Ah, no I think it is very accessible and just easy to get round either by streets or um, you can go down to the river and the river just follows along around to the next park and then you know, all the way to [town]. So you can either walk the roads or the track. So I don't think there's any issues with accessibility to be able to walk. And the lighting, the footpaths are all pretty good.

Mm. I was up there a couple of weeks ago, and where I was staying they would say "oh just take this road and you'll get down to the beach" and I was amazed how accessible the beach is. And I had a lovely long walk and came back. But was very close to where I was staying. And the other nice thing was that with the parks and the beach you know, you could be, it's not like you are in the middle of a town either. [town] has really preserved it's dunes and vegetation well. So you can be out and you can think "oh I could be anywhere here, this long stretch of beach, that's all I see".

Yeah. You come over the dune and all of a sudden you're in the middle of the town.

Yeah, it's beautiful. So, um the other side of it, which is what we've been talking about is the aesthetics. So if I were to ask you about the aesthetic qualities or the attractiveness of your neighbourhood, what sorts of things would you tell me about? You've already said the parks, the trees, the river, the beaches. Anything else you wanted to add?

Oh I think the Council do a very good job of keeping the place neat and tidy. And the parks are its biggest, and the playgrounds, they've invested a heap in children's playgrounds around the parks. And I know lots of families come from [city] and [city] to [town] to spend time. It's one of its assets is the huge park area. So that is probably the biggie.

Yep. Yep. OK. And do you think the aesthetics of your area influence whether you are physically active or not?

Yeah. Definitely. Yes. When it's green and the trees, and whether you've got shade. Yeah, it just makes it that much more comfortable and pleasant. So it's a pleasant outlook rather than just a concrete um yeah jungle, for want of a better word. But

yeah, you are not just in concrete and cars. There's trees and grass and animals, you know, birds. It's pleasant to go for a walk or a job.

Yeah. And it links in with what you were saying about, it's pleasant and it's a stress free environment in that sense. So you know you were talking about well being and doing activity and also gives you that well being effect. And I'm sure the aesthetics of it, a pleasing environment sort of add to that too.

It does. The wind in the gum trees. And they have put a heap of, they moved a number of big palm trees that were down in the river and they were dying, and they have moved them along near the surf club along the park there. And they've taken off brilliantly. And it looks like the Royal Esplanade – it's really nice there, they've done a great job with it. So you might gather, I like living where I live.

Absolutely. Look I can actually – last couple of times I've been in [town], because we've been doing interviews for a little while um, I've actually always made sure that I have actually caught up with some of the places that people talk about. And particularly, particularly in the [town] area it is very beautiful. And you have just got so much choice of river, parks, beaches. And I even went and had a look at the cycle, walk track at one stage. And out to [town] as well because that is the other area that we are you know, asking people about. Yeah, so it's very beautiful. You go "oh this is, yep".

I can understand why, yeah.

Um, now and of course one last question about aesthetics is if you wanted to be more active are there features, any features of your area that might help if they were more aesthetically pleasing or attractive?

Um, probably a bit more lighting on some of the tracks, yeah for winter. Yeah just to light that up a bit. Yeah because toward the evenings in winter it's dark early. So that might be the only thing.

Yep. And the last area is safety. If I were to ask you about safety in your area, what sorts of things would you tell me about?

Um, I think probably going back to the sense of neighbourhood and community, makes it safe. It's a friendly neighbourhood, everyone says hello to everyone. And for the actual physical activity, yeah you can be away from the roads if you want to. There's kilometres of park and man-made track that you could walk, run, ride on

without um having to worry about cars and things. So there is that safety aspect as well.

Yeah, that's a really good point. So does road safety influence whether you're physically active or not?

Um, not me myself, no. I don't think, no.

No. And you've got other choices to make if you wanted do that.

I think the other safety thing is there is a lot of really well run clubs. There's a feeling of safety of your family and children. And yeah, well run, well established. The surf clubs and basketball clubs and the soccer clubs are really well established with experienced people. So there is that feeling of safety for your family and your kids as well.

Oh that's a really good point. Thank you for that. Because it is a broad question about personal safety and there's, and ah that's a good point about sort of those groups and clubs as well. Mm. Um, does personal safety influence whether you're physically active or not?

Um, no. No. I feel safe, so yeah.

Yeah and it's a safe community that you are living in. That's what we were saying. If you wanted to be more active are there any personal or road safety issues that if they were addressed might help you be more active?

No, I can't think of any. I mean we are spoiled for choice.

Yes, exactly. And as a lot of people say about [town], they um very much agree. But also the plan, it's not only a short term plan, it's a long term plan to continue that making um you know places for activity available for people. So it's going to get better and better by the look of it.

Yeah. It really is a nice place to live.

Yeah. OK. So that's it. So before we finish up is there anything else you'd like to tell me about where you live or the environment you live in or physical activity that we haven't covered today?

Um, only that, as I was saying, I think that a smaller community sometimes has benefits to it rather than the, I've found just in comparing the two from here to when I was in [city]. Because of if they've got sufficient um resources as in basketball stadium and a football stadium and soccer and of course being by the beach having a

surf club as well. If they've got sufficient resources of that type, I think there's a benefit in actually being in a community of this size. As to the um the size of [city] where again they may have a bit bigger swimming pool and stuff, but you've got to use your car or public transport to get there. And then you are not quite sure whether your kids are as safe as they are at the smaller community. Yeah, so there can be benefits I think. And [town] is probably a really good example of that. There would be other places of similar size that wouldn't have the same facilities that [town] has, where there probably wouldn't be the benefit to an urban, you know, a city. Yeah, so there is that comparison.

Yeah, I think that's a really, really important point that you've made. Because there are benefits of living in rural areas. And [town] is a very good example of that, and as much as anything we learn from, you know the point you have made means that we learn um with the study about what actually does work really well. Um and that's important to let people know about too. Especially at the present moment when there is such a focus on health and activity, and the importance of it. So mm. But yeah.

Yes, I think the bottom line is you can do physical activity, it doesn't matter where you are if you've got the state of mind, but obviously having an environment that assists that makes those decisions easier.

Mm. Absolutely. No that's a really good, no that's a really useful point too. Um, and sometimes when you ask people about rural areas and it goes with the course that you've picked the rural area for its beauty and aesthetics, so sometimes it's a bit hard to separate out OK, well does that help you be physically active or not. Um, but yes, yes it does. The environment does have -

Yeah, it does I think, yeah.

Mm. Absolutely. Look thank you so much for that.

AUDIO RECORDING ENDS

Interviewer: So today I'm here to really hear a range about your thoughts about a range of issues to what it's like living where you're living and how this might relate to your physical activity. And remember there's no right or wrong answers here, we're actually just really interested in your experience and your thoughts and your ideas. And if there's anything

you're unsure about, just please don't hesitate to ask. Stop the interview any time; just ask me, that's fine, not a problem. And also, if there's questions you'd prefer not to answer or don't feel you can, that's fine. Now just a general question around thinking about the place, the place you live, for example what is it like to live here? And so I just wanted to ask you first of all if you could tell me what it's like living around here, likes, dislikes?

Respondent: I love it.

Interviewer: Yeah?

Respondent: Yeah. I chose to live here and we live just out of town, we live up the hill behind the back of my husband's parent's farm. So my in-laws are down the bottom of the road, which is, and I get on really well with them so it's really nice to have that family close by.

Interviewer: Do you live on land?

Respondent: Yeah.

Interviewer: You're saying you're just outside the town itself?

Respondent: Yep, yep.

Interviewer: How much land do you live on?

Respondent: Together it's about 80 acres.

Interviewer: Right that's nice.

Respondent: Actually it might be a little bit more, might be about 90, between we own a couple of blocks and my parents-in-law own the remainder. And yeah we run, we've got eight cattle on there and a bull that's really just a hobby since it's, we make enough out of it to put back into the farm maintenance. So we don't make a profit from it, but we make enough to cover the fencing and the weed spraying and stuff like that. And we enjoy that, doing that work and working with the land. So that's, and having our own meat and stuff and working with the animals, so that's why we do it.

Interviewer: And that's what attracted you down here to this beautiful area to do?

Respondent: No.

Interviewer: So idea how it came together. So how long have you been living there now?

Respondent: About six years.

Interviewer: Six years in [town]?

Respondent: Yep.

Interviewer: Yep OK and so where you're living, you were saying you were just outside the town, just how far is it in terms of kilometres or driving time?

Respondent: It takes about four minutes to drive and I think it's six kilometres either way.

Interviewer: OK right, that just sort of helps sight, yeah.

Respondent: Yeah.

Interviewer: Now in the research, as you probably heard on the radio, a lot of this research is being done in urban areas, that's why I wanted to do it in rural areas. And people often refer to their local neighbourhood. And I was interested in understanding what term neighbourhood means to you who live rurally. Does it have a meaning for you?

Respondent: I'd probably tend to use the word community more than neighbourhood.

Interviewer: And what would, yep. So if I also ask you about the word community, what would that mean to you?

Respondent: It would talk about the context, the people and their place around where I live. So we live on the [town] side and we're actually technically closer to [town] the of shops and stuff so we link a little bit with that in terms of the shop and the swimming pool that we use. But then I work in [town] and we have childcare in [town] and we go to the supermarket in [town] the post office, we get our mail here and stuff like that. So and we've got a couple of friends that live around the [town] township. So yeah, that would be what I'd think about as my community or my neighbourhood, sort of between [town] and just in [town].

Interviewer: So that sort of becomes a whole community for you in a sense?

Respondent: Yeah that's where we sort of do what we call our local stuff.

Interviewer: Yep, yep. So it's really about where people are and also where facilities are that you go to.

Respondent: That we use in our everyday life.

Interviewer: That's a really good term that you use in your everyday life. And can it be quantified in terms of does it have a measure of distance for you? Is it a couple of kilometres?

Respondent: Well for us I guess it's probably sort of about six kilometres, so it sort of comes over to here, and maybe the other side of town, so six or seven kilometres. But it's sort of, you know for us it's sort of what's within five minutes. And I think we're pretty lucky that we live within sort of five minutes of town and I guess we probably, we've chosen to live reasonably close to town rather than we could live further out, but it's good to have that convenience. We don't like sort of travelling, commuting that much, so yeah.

Interviewer: So that works out really well. And that would be sort of driving distance that we're talking about too.

Respondent: Yeah, sometimes we walk to places or I used to ride a bike a bit, and sometimes I ride to work but I don't anymore. Yeah but mostly driving but a little bit of walking these days, yeah.

Interviewer: Well we'll go on to a bit more about that in a moment, so I'll ask you a bit more about it.

Respondent: Like we've got within walking distance from home we've got our parents-in-law and the swimming pool and we can walk around to the [town] shop. So we do some socialising and some recreation within walking distance. And sometimes, quite often we're lazy and we drive it but we can and do walk it sometimes too.

Interviewer: Yeah and that's often because you're juggling other things as well.

Respondent: Yeah, yeah or going onto somewhere else.

Interviewer: Yeah exactly so you drive, yeah. I want to ask you a bit about your physical activity, and again there's no right or wrong about this, it's really about what it means to you, to you personally. So what, if I

asked you about your physical activity, what would it actually mean to you?

Respondent: My physical activity mean to me. I think it's something that's important for my health, and that's important and that's actually, like it's something that should be doing so it's sort of an obligation to myself. But it's also something I enjoy doing, and that's so this sort of mental enjoyment out of it as well. Yeah I do get mental enjoyment out of physical activities.

Interviewer: Do you consider yourself as physically active

Respondent: Yes probably not as much as I'd like to be or could be.

Interviewer: Yeah whatever that means of course.

Respondent: Yeah, that's right. Yes I'm more active than inactive, but I should, I'd like to be doing more.

Interviewer: Yep. And why would you consider yourself physically active?

Respondent: Because of what I do, because I do do stuff. And because, and I'm sort of driven to do that because I'd rather be physically active than be physically inactive. I actually prefer to be.

Interviewer: OK now I'm going to ask a few more details, a bit more detailed questions about physical activity. But first of all, where does physical activity fit into your life?

Respondent: Probably around the fringes.

Interviewer: Yeah it's sort of part of your life but also there's other things that you get to do?

Respondent: Yeah it's not work and it's not the primary home focus. I can't afford, I can't do it as a core thing at home, but it's an add on.

Interviewer: OK. So in the research we're doing, when we think about physical activity we're often sort of talking about any activity that lasts more than ten minutes, something that gets your heart rate up, huff puff, whatever, at least warm up a bit. So thinking about this type of physical activity here, just over the last two weeks, now at work have you been active at work for at least ten minutes at a time?

Respondent: No. No I can't think of anything. No.

Interviewer: OK and is that a usual thing or would it be does your work change seasonally?

Respondent: It changes a little bit but that's a pretty usual thing for me at the moment. I do get some exercise opportunities at work but I'm mostly office based now and so it's just, it's sort of those fringe add on things. It's just now and then I'll get let out on good behaviour and get to go and do something physical or there will be a bushfire or some burning or something and I'll get to do something active with that. But not, yeah, not most of the time.

Interviewer: OK. Yeah and so it's been about a change of work and the type of work that you do which is sort of more based in the office, yeah?

Respondent: Yeah.

Interviewer: Yeah OK. Now if you wanted to, is there anything that could have helped you be more active at work, either in terms of time or someone to be active with or somewhere to be active?

Respondent: [text removed because of identifying information]

Interviewer: So what could have helped that in terms of a change so that you could become more active?

Respondent: More female role models within the workplace. And a less gender stereotyped culture. That's still a problem down here in [region], and probably in Tasmania, in [workplace] too. It's not in mainland [workplace].

Interviewer: Now I'm going to ask you in the last two weeks to think about whether you've walked or cycled at least ten minutes at a time to get to and from places?

Respondent: Yeah.

Interviewer: Yeah?

Respondent: Yep.

Interviewer: OK and could you tell me a bit about it?

Respondent: I think there was some stage last week where I walked down a hill with my daughter to our in-law's house. I think it was on the weekend actually, just on the weekend just gone. Yeah, so that's sort of about

ten minutes or a bit more and just pushing her in the stroller. And then I started walking back up the hill and then my hubby came down because he finished what he was doing.

Interviewer: So is that a usual thing, it would be a couple of times a week that you would?

Respondent: Yeah, yeah. And I went [x] sort of about a week and a half ago as well and so that involved walking to the [x] and then c[x].

Interviewer: And how often would you go [x]? Not as often as you'd like?

Respondent: No, probably about once every three months. Once every two or three months.

Interviewer: Yep OK. So in terms of the intensity of the exercise that you did at the time, you've told me a bit about how long you've done it and how often, what would be the intensity? Would it be sort of vigorous or moderate, slight?

Respondent: Walking down the hill and caving were probably more moderate. I did some wood cutting on Friday as well and that was a bit more intense and that was a couple of hours of cutting wood. But not walking or cycling.

Interviewer: No that's fine.

Respondent: But that was definitely physical activity or exercise.

Interviewer: Yep, yep absolutely. And so does this change at all, like you were saying that you were with your daughter, does it change at all in terms of the time of the year or the season?

Respondent: Absolutely yeah. Winter here it gets really hard to get out and do stuff outside, just because the days are really short and it's, it can get really cold and it can be wet. It's probably wetter during spring but there's probably sort of four or five months there where you just really notice that it's hard to get outside. You get up and you go to work and the sun comes up as you're going to work, not while you're at home. And then you knock off work and the suns gone down. And sometimes I'll get out, if it's a fine or warmer evening, sometimes I'll just get out in the evening after I put my daughter to bed and I'll just

go for a little five or ten minute walk down the road just to be outside and to be doing something.

Interviewer: And you can do that.

Respondent: Yeah, yeah. But it's walking in the dark but that's alright, we had lights on yeah. And we knew the road.

Interviewer: And the next question is actually looking at being active around the house and the yard, and you've already told me about the wood chopping and that it was vigorous. Is there anything else that you might have done for at least ten minutes at the time, that's inside and outside, like housework and things like that?

Respondent: Cleaning is never really that vigorous exercise. I do a little bit of gardening now and then which isn't usually hugely vigorous but it can be. Through winter we feed the cattle and that can involve a bit of good exercise because it depends where they are but sometimes you drive there but sometimes you walk across a paddock to the next hayshed and then you grab a new bale of hay out and carry out, hopefully not too far, or you might put it in the wheelbarrow and carry it a bit further and then throwing that out to the cattle. So yeah, so that counts as exercise. And yeah so you actually sort of do, we probably do do more of that in winter.

Interviewer: Yeah so it sort of varies? There are other things; there are other sorts of exercise you do in winter versus summer.

Respondent: Yeah I don't, I still don't think it's as much as you get in summer because I still, I know I get that feeling towards the end of winter and as the days start getting longer, and you get sort of a cabin fever feeling and you're just… I know I'll get those evenings where I'm sort of like I just need to go and do something because I'm not physically tired. I'm mentally tired from work but I'm not physically tired because I haven't done anything and I just needed to actually get rid of a bit of energy so I can go to bed and go to sleep.

Interviewer: OK now when you do do exercise, is it around the house and outside, is it, actually you've said feeding cattle can be together with your

partner… I'm just trying to see whether some of the exercise is with by yourself or with others.

Respondent: It varies.

Interviewer: Varies?

Respondent: It varies yeah. And it's yeah, some of it's by myself and some of it's with my partner or my daughter.

Interviewer: And if you have wanted to be more active, is there anything that could have helped you be more active around the house or the yard?

Respondent: No not really. Better weather but that's what we've got so, yeah.

Interviewer: And with walking and cycling, is there anything that could have helped you be more active?

Respondent: It's juggling priorities to be able to sort of do that stuff versus the big thing is caring for my daughter. And like I can't really justify putting my daughter in childcare to go and do my own exercise.

Interviewer: Yeah.

Respondent: Yeah that's not a black and white statement because I do sometimes, I'll leave her with my parents-in-law and then I'll go and feed the cattle for example, but I guess it becomes a choice that I've made to do that.

Interviewer: You sort of work out a balance for your life?

Respondent: Yeah and sometimes I can, like I can bring her with me, we can go for a walk with the pram and so sometimes I can do both but I don't like to impose on her purely for my benefit. So like if she's tired and worn out towards the end of the day and she doesn't mind sitting in the pram, then it's fine to do that. But if I hate the idea of taking the pram for a walk and having her distracting me and saying "Mummy I want to get out", she wants to get out and run around or go and do something, that's not, yeah. And she's not big enough yet for her to be out and running around and doing something, that's not really… It is exercise actually sort of running around after her a little bit.

Interviewer: How old is she now?

Respondent: Carrying her around or whatever. She's two and a half.

Interviewer: Yeah, that's a great age.

Respondent: She's still small enough to carry and piggy back on my shoulders and stuff like that, so we do get exercise that much, she's just next door there somewhere. I saw her before.

Interviewer: Is she, because childcare is next door.

Respondent: Yeah.

Interviewer: It's a lovely day for playing outside.

Respondent: Yeah.

Interviewer: And OK and the last bit of this is having a look at being physically active in your leisure or your spare time. And in the last couple of two weeks did you do that, were you physically active for at least ten minutes?

Respondent: Yeah.

Interviewer: Thinking back…

Respondent: Well the [x] that I did over Easter was recreation. But I probably only get to do it once every two or three months. Yeah but then it's a good go, it's sort of a good half day or day activity in the bush.

Interviewer: And that's a group activity isn't it?

Respondent: Yes, yes and I'm part of I guess a sporting club, we've got a club for that. Yeah we've…

Interviewer: Is that very far? No it's not that far away, the [x] are they?

Respondent: [text removed because of identifying information] Yeah, yeah every now and then very, very occasionally, so probably once every couple of months I'll go for a swim at the local pool, like just me swimming, and swim laps. And like firewood cutting, I guess, I don't know if that's… But we kind of do it as recreation but we get a bit of income from it as well. And then some of it's our wood so it's just an extra duty, but I feel good about, I enjoy doing that too.

Interviewer: And I think what we do in our lives isn't sort of very easy to segment into the different spots, a wide variety of things that we don't miss out on, sort of parts of our lives so that's fine. And if there's anything, if

you wanted to, is there anything that could have helped you be more active in that sort of spectre? If you wanted to.

Respondent: No.

Interviewer: That's fine. I'm going to shift the topic a bit and actually look at the physical environment OK, and that's the other part of the questions. So we've talked a bit about where you've lived and your physical activity, and now I'd like to talk a bit more about the physical environment of where you live and work. And when we talk about physical environment, we often mean the physical things around you, the roads, the trees, the houses, the shops, the traffic, whatever, footpaths. Some of these things that people have, in urban areas, have actually said might be important for their physical activity, so we'd like to know whether that they're relevant for you as well. I guess OK just a general question to start with is can you tell me a little bit about how easy or difficult it is to be physically active in your actual community area? Is it easy or difficult?

Respondent: You pretty much need a car. I find it fairly easy.

Interviewer: So as long as you've got a car to get to places, that's fine.

Respondent: Yeah, yeah.

Interviewer: OK so the availability and accessibility of places to be active. If I were to ask you about places to be active in your community, what sorts of things would you tell me about? We've talked a bit about this.

Respondent: We're really lucky that we live really close to the pool. And I know that's not common infrastructure to have around, but we have an indoor heated pool just about like walking distance from home.

Interviewer: Is that [town] side?

Respondent: Yeah over on the [town] side. So we're really, really lucky with that, and we use it. I guess otherwise you might have a river or a, like around here you probably tend to have a river or a beach or a foreshore or something like that but we've got the pool so I use that. I know when my daughter was a big younger, it's a bit tricky; it's a bit hard pushing a pram around. There are spots in town that have

footpaths but then there's spots along the way where there's like a flight of ten steps to clunk your pram down or there's a section with no footpath across a bridge or something like that. So there are obstacles, reasonably frequent obstacles along the way to, if you want to sort of walk with a pram type vehicle, if you want to carry a child no worries but yeah, walking with a pram, it can be a bit difficult. And that's where like if you have a car and you can drive somewhere else where there is a footpath or a walking track or something, then yeah you can take advantage of that and do that. And that's the same sort of thing, you can drive down the beach or something like that or drive to go for a bushwalk or something like that.

Interviewer: And you were mentioning you had access to the river as well? That was for swimming wasn't it?

Respondent: Yeah swimming or splashing or walking or whatever.

Interviewer: You can actually walk along the river?

Respondent: You can walk, there's a little path along the river in town here so you can, like if it was a hot summers day you could have a little walk along that and sort of go down and splash your feet in the river or whatever, so that's nice. And we've got the [x] River sort of along there, so there's various places along the river if we went for a drive to look at the beaches or foreshore parts or something like that, we could go for a drive, stop there and then go for a splash or a paddle or a planer boat or a walk or something like that.

Interviewer: OK and are there any other recreational facilities, parks or anything like that?

Respondent: There's some really good playgrounds around. Yeah, the Council has done a really good job of putting playground equipment around. So yeah there are some really good playgrounds around for the kids. There are a few different walks sort of out in the State Forest and some of the parks nearby. Yeah I don't sort of do them so much, I guess I probably do bushwalking as part of my [x] so I tend to sort of do it that way. And I don't get as much of an opportunity as, yeah I

just sort of kind of prioritise a lot. I mean I used to do a lot more, before I had a kid I'd be out there at least every three weeks or something, but yeah. And that will change. That will change as she grows older.

Interviewer: So you're saying there are available places and accessible are they, you've actually talked about the pool, are they easy to access in terms of their cost and opening hours and things like that?

Respondent: The pool is a little bit pricey but it's worth it.

Interviewer: Is that just part of a pool, it's a pool or is it a gym complex with it?

Respondent: Yeah and a basketball court and a couple of squash courts there as well.

Interviewer: So it's a recreational facility?

Respondent: Yeah, yeah. And I think there's a gym there, yeah.

Interviewer: That's very impressive.

Respondent: And now and then they do aerobics classes and stuff like that. They used to do Zumba there but…

Interviewer: Yeah, Zumba seems to be a popular thing to do.

Respondent: Yeah, yeah.

Interviewer: So does having places to be active influence whether you're active or not?

Respondent: Yes. Yeah definitely.

Interviewer: Yep clearly.

Respondent: Definitely. Yeah if we didn't have that pool there, that would be much harder to, we probably wouldn't be motivated enough to drive 20 minutes to the next pool which I don't, which is outdoors so it would be a bit colder. And we wouldn't use it as much because it was colder as well. Yeah so definitely, yeah.

Interviewer: And if you wanted to be more active, are there things or places that would help if they were available or more accessible?

Respondent: It's helped me before be more active having like classes, like when the Zumba class was running at the local pool or sports centre, that got me motivated and I'll just go in and do that each week. Yeah, and I

sort of have to probably join, get back into one of the other classes so that's really helped, just having that sort of thing. It would probably help if they ran yoga classes at the local hall or something like that, it would be encouraging and perhaps giving you a bit more motivation, that sort of thing, yeah. I'm not motivated enough. I've got a friend who drives 20 minutes to [town] to do yoga classes but I'm, you know it's the evening and I'm not motivated enough to drive that far.

Interviewer: Which sort of leads onto the next question, you've already talked a bit about this, destinations. If I were to ask you about places you were to walk or cycle from your home, what sorts of things would you tell me about? And is there anything you want to add to that because you've actually told me a fair few of your destinations, in terms of shops and schools and things like that.

Respondent: Yeah well from home, like if I was self propelled, I could be going to a neighbours or the parents-in-law or down the paddock to the animals or to the firewood or something, or very occasionally around to the pool or the shop.

Interviewer: Yep. And does having more destinations influence whether you're active or not?

Respondent: Yes. Yeah it makes it easier to make the choice to do that. I can wake up Sunday morning when we go for a swimming lesson at the pool and sort of say to my husband "Right the sun is shining, we've got time, I'm not running late, I'm going to put my daughter in the pram and walk down the hill to the pool. Can you come and pick us up in 20 minutes or half an hour or something?" So yeah, whereas if it was only driving distance, I'd only drive. I wouldn't say I'm going to walk ten minutes down the road and then you drive past and pick me up and we'll drive the next five minutes, having being able to go all the way there. And we do do that a bit too, we sort of do one way trips, partly because we live up a hill and it's a challenging push of the pram back up the hill. So partly we sort of cheat a little bit with that and I'll chose to do downhill trips and go out somewhere and then coordinate

that my husband is knocking off and driving past on the way home so he can give me a lift home or something. So partly that but sometimes I do walk up the hill.

Interviewer: It's good, I mean especially if you're living rurally, sort of working out some ways to do it so you're not sort of left with having huge distances so that's a good idea. OK and if you wanted to be more active, would it help if there were more destinations in easy walking or riding distance?

Respondent: Yeah of course it would but then I would lose one of my destinations, which is the paddocks around me. So I chose to live without people or buildings or infrastructure around me…

Interviewer: Because you've got the land around you.

Respondent: Because I've got the land and the animals so that becomes a replacement destination.

Interviewer: That's a really good point.

Respondent: So we've still got destinations around us but they're just not, yeah. Yeah and so, yeah no in that sense no I don't think it would necessarily help because I wouldn't be walking up the top of the powerlines hill to feed the cattle, I'd be walking up the top of that hill to visit [woman] or [man] or [woman] or whoever or go to the shop or whatever.

Interviewer: No that's really a good point, yeah absolutely. Now I'm going to talk about community or it's more in the urban sense of neighbourhood function and design. So if I were to ask you about the built or manmade features of your community area, what would you like to tell me about?

Respondent: It used to be a predominately rural landscape with people living in the town and then outside the towns was farming production oriented, so either paddocks or orchards, and the farm houses or the houses of the workers that worked there. But it's changed and it's now, there are a lot more houses and dwellings out in what used to be the rural landscape, of people who I would sort of say don't work in that landscape, so they commute out of it to go to their work. Sometimes

they're retired and they do occupy themselves in their landscape with their retirement, with their veggie garden or building their boat or whatever they do, but they're much more drive in drive out people. And so that kind of, you're talking about destinations, so that kind of gives…

Interviewer: No more about the features in terms of footpaths or lighting or do the streets connect so you can sort of walk along them, their walk-ability.

Respondent: Well what it does do is it increases the traffic along those outer roads. So where you used to be able to push a pram along one of those roads out of town and push your pram like across the narrow bridge that doesn't have a separate footpath and you push the pram on the road or you'd push it kind of on the edge of the road…

Interviewer: Because you're on the edge of the road, yeah on the footpath.

Respondent: Yeah and you could do that and you could walk two or three prams abreast and you could walk with another young mum and have a chat as you went along, and you might have to pull over once or twice for a car coming and you'd hear it coming, it's more difficult to do that because there's more traffic. So you have to sort of keep separating and pulling over.

Interviewer: So that's something that you've noticed in the six years that you've lived here?

Respondent: I really noticed it, well I really noticed it when I had my daughter and was doing that. All of a sudden, like I'd used to, it didn't worry me walking along the edge of the road and pulling off the road but when you've got a pram, it's more difficult to manage. And I did really notice it then that yeah, it was.

Interviewer: It had become more busy, the traffic.

Respondent: Well no, not that it had become more busy, but I noticed the effect of the busyness. And I noticed that if I was just walking on my own or just walking with a friend, I could, it was easy enough to just sort of fall in behind the other person and have your car go past. But if you had a pram, and it was really easy just to step onto the gravel but if

you have a pram and you're walking two abreast, then you take up much more length and width so you actually have to pull off, you can't just sort of snuggle up to the person next to you and stay on the bitumen, you've got to pull your pram in behind them. And if you're trying to get off the bitumen because you're a bit more concerned about your child too, you don't want that car flicking a rock up or anything. So you do sort of want to get off the road further and minimise how far out you are. And it's more difficult because you can just walk onto the gravel with your feet, but with a pram you go onto the gravel and you know, the child will drop a toy. So that was much, I noticed that that doesn't really work. And if you're trying to walk two prams around town and up that flight of ten or 12 steps, God maybe you know… Walking up the steps is easy if you don't have a pram but if you've got one pram it becomes more challenging, if you've got two or three, it's just a headache. Let's not do this walk again, let's drive somewhere where there's a flat path and we can do it easy.

Interviewer: Yep and are there any paths like that around here? Because it sounds like around the township you were saying it's quite broken up in terms of access to footpaths.

Respondent: Yeah, yeah.

Interviewer: And you really notice it when you have a pram.

Respondent: We've been for a good walk with the prams in [town] which is about ten minutes' drive away up the river, and [town], and yeah, yeah.

Interviewer: But in [town] you notice a difference?

Respondent: Sorry?

Interviewer: But in [town], no.

Respondent: No.

Interviewer: No.

Respondent: Not for pram walking.

Interviewer: OK.

Respondent: You'd choose one of the roads going out of town that would be your best option. You could walk around the middle of town but those

interruptions, the sticks or all the bits without footpaths are a pain. So you're better off trying to find one of the roads that goes out of town that's quieter, but I've tried a few of them and I've noticed they tend to have more traffic on them now. You might find the odd road that's quieter. There's one road I can think of that's quieter, but most of the roads, yeah there's just houses thrown up along…

Interviewer: Does that make you think twice about it?

Respondent: Yeah it's just not as much fun; it's just harder work, yeah.

Interviewer: OK so this sort of starts to answer that question of if you wanted to be more active, are there manmade or built features in your own neighbourhood and community that would help? And obviously you've talked about the lack of and started looking at other places where it's actually easier to walk perhaps.

Respondent: But that's really just affected me in what for me is the recent chunk of my life, whether I have been carting a pram around and a young child. So before that it wasn't a problem. Walking as an individual or as an independent adult is fine.

Interviewer: Now aesthetics, if I was going to talk to you about aesthetic qualities or the attractiveness of the community that you live, the area, what sorts of things would you tell me about? Because I remember you were saying one of the reasons you live here.

Respondent: Well I actually just really enjoy walking out of town along those roads and looking at the landscape go by and now and then passing points of interest that you comment on, someone's cattle yards or someone's house or some junk heap or whatever, I just really enjoy passing through that landscape.

Interviewer: So you find it really pleasing, your environment around you? Beautiful trees and river.

Respondent: Yeah, trees and hills and sometimes water.

Interviewer: And just on the other side of it, are there any aesthetics of, I usually sort of cover rubbish and litter and graffiti, that's the other side of it, is

there any of those sorts of things in your environment that you notice much?

Respondent: No, it doesn't spring to mind. Fog on the cold winter mornings and evenings down in the valleys, the fog that you might have sunlight above it but not down in the valley so it's a little bit colder and mistier and so forth, that makes it less nice. But I'm just trying to think, unpleasant? No it's OK, nothing springs to mind.

Interviewer: And so the attractiveness of the landscape and the area, does that influence you whether you're physically active or not? Does it make it easier to be physically active?

Respondent: Yeah, yeah. It makes it more enjoyable.

Interviewer: And if you wanted to be more active, are there any features of your area that might help if they were more aesthetically pleasing? Overall?

Respondent: No.

Interviewer: No, because you live in a beautiful area.

Respondent: Yeah there's not, like there's not really too many problems with rubbish or whatever. You notice it sometimes walking along the road and having rubbish thrown from cars on the side of the road, and I've only done it a couple of times and sort of taken a plastic bag and actually picked a bit of it up. I used to do a bit of that with the Girl Guides.

Interviewer: Yeah I've done that too when I'm doing bush care, cleaning up the road.

Respondent: But I haven't really done a lot of that, yeah.

Interviewer: OK. Just one last question about safety in your area, what sorts of things would you tell me about? Now you've already told me about some increased traffic, does, if I was to ask you about safety what would you tell me about it?

Respondent: I'd think about whether I'd carry a mobile phone with me when I went out walking. And I think generally if I've got my daughter with me, then yeah, generally I would just in case something happened with

her. Just in case. Or just in case I got stuffed pushing her back up the hill and I wanted to ring my husband and see are you coming home soon because if you are you could pick me up on the way back up the hill. So yeah, so I'd sort of think about the mobile phone for that but I don't know whether I'd always take it on my own. It would depend how I was feeling.

Interviewer: Does road safety influence whether you're physically active or not?

Respondent: Well it has like when we were doing the pram pushing it influenced us so to say, well this isn't so much fun anymore because we keep feeling like we might get run over and it's too hard work trying to keep getting off the road. Yeah I don't know whether we could have found a better time to walk along, like whether there would have been a quiet time for traffic or stuff like that, I don't know. But yeah, yeah that's a bit of a problem.

Interviewer: And so if you wanted to be more active, are there any, well you've actually talked a bit about some personal safety issues because you've talked about a mobile phone, or road safety issues that would need to be addressed that might help you be a bit more active.

Respondent: Yep. There's a section of bush on our road that has trees that sometimes drop limbs and I think the key with that, we think about that in terms of safety and they key to that basically is don't walk down the road through the bush when it's windy. But you generally don't really want to go out walking when it's windy anyway. Sometimes we have to feed the cattle when the weather is bad and you think, like that would be a time when I would probably tend to carry my phone on me because if I have to walk across a paddock and it's wet and it's windy or something, if a branch fell on me or if I slipped in the wet mud and fell over and did my leg or something, then I'd carry the phone as a backup communication or, yeah. So that's a bit… But that's just managing your own safety in your environment.

Interviewer: That's fine. Well that's the end of the questions but before we finish is there anything else you'd like to tell me about where you live, your environment or your physical activity that we haven't covered today?

Respondent: No I don't think so.

Interviewer: Thank you very much.

Respondent: No that's alright.

Interviewer: So, I thought I might just start with a bit of general questions about the place you live and so can you tell me a bit about what it's like living around were you do and the likes and dislikes of it?

Respondent: I really like it. It's a little rural village. It's very hilly.

Interviewer: So this is [village]?

Respondent: Yeah.

Interviewer: Which is about 15… did you say about 15?

Respondent: Between 10 and 15 minutes from [town].

Interviewer: Yeah.

Respondent: Yeah, so it's basically farming land.

Interviewer: Yeah.

Respondent: You've got lots of cropping and lots of farm… and sort of steep hills down into the river. It's a nice quiet place. Beautiful scenery. Absolutely stunning and we've sort of got an acre block so we've got a bit of ground around us.

Interviewer: OK so you've got just a nice bit of land in that sense, yeah.

Respondent: Yeah.

Interviewer: And you're surrounded by farmland?

Respondent: Yeah.

Interviewer: Yeah, OK. SO the property is a sort of standard house with a bit of just the acre around it, is it?

Respondent: Essentially, yeah.

Interviewer: Yeah and what is the length of time you've lived there?

Respondent: Ten years.

Interviewer: OK and your reasons for living there?

Respondent: I grew up on a farm and so I like the open space. I find it difficult sort of living in close to people.

Interviewer: Right.

Respondent: So I like the quieter country feel to it. A bit more space.

Interviewer: And OK the distance to the nearest town. We've talked about… it's about 15 minutes' driving time.

Respondent: Yeah.

Interviewer: In kilometres, how far is that?

Respondent: Good question probably about 10 kilometres I reckon.

Interviewer: OK so that's pretty close by.

So in research with women living in urban areas and cities which is where a lot of this research has been done that's why we're wanting to… it hasn't really been done with rural people, people often refer to their local neighbourhood. And we're interesting in understanding whether the term neighbourhood actually has meaning in a rural setting for people who live there. So, what does the word neighbourhood mean to you?

Respondent: I guess it's got more of a city feel to it in some sense. It's something I would associate more with sort of being in residential built-up areas. With the defined geographical space, perhaps. I don't know whether I'd say the [village] neighbourhood. I don't closely identify with the concept. I understand you know the concept of neighbours and place. But perhaps not neighbourhood. I don't know what other term I'd offer instead.

Interviewer: OK.

Respondent: Whether community might be more…

Interviewer: I was just about to ask that to you… ask you, 'What about the word community?'

Respondent: I think community has got more sense of identifying people who are living in a space and share a similar living geography perhaps. And certainly…

Interviewer: Yes. So for you living in [village], what geography would that be?

Respondent: Since the kids have started school I've developed more of that sense of [village] community. I think that the school brings people together so I get some sense of identification.

Interviewer: And there's a school in [village]?

Respondent: Yeah.

Interviewer: OK yeah.

Respondent: So I wouldn't have perhaps felt a part of a community even though I lived there prior to the kids going to school. So for me I guess it's a collection of people that live in a shared place that are sort of …yeah it's probably the school community that I most closely identify with.

Interviewer: And is there a distance associated with that in [village]?

Respondent: In terms of school do you mean? Or in terms of how far I think the community spreads?

Interviewer: How far it might be, yeah? A few kilometres or... ten kilometres or…

Respondent: Probably even like 10 or 15 kilometres.

Interviewer: It's the sort of radius the school what do they call it? Catchment is in.

Respondent: OK, no that's good.

Interviewer: Just an aside, [staff member] the support worker is in the office there but she's just leaving to literally walk out the door and leave at about nine.

Respondent: That's fine.

Interviewer: Yeah, OK. Now, I'm going to move onto physical activity and I just want to ask you what physical activity means for you, because it's a really broad terms.

Respondent: I think it's changing, that concept, for me. Since I've had children. Pre children I would have seen physical activity as a planned set time that you sort of did and went you know for a walk or to the gym or… that you'd structure it like that. I guess since I've had kids physical activity has more become for me, movement within the day. But more sustained movement if that makes sense. So I guess I'm changing my perception of that to more incorporate that in terms of the activities of the day. Because to find extra time as a mum of three and working, to do set exercise or physical activity seems too hard. But if I incorporate it into what I'm doing…

Interviewer: Yeah, into life itself.

Respondent: Yeah so that concept's changing for me.

Interviewer: That's really interesting. And I think it's an interesting concept that we're… I agree with you, I think that we're really just starting to explore it. With the study we're starting to find a number of people are starting to talk about that which is really interesting. And do you consider yourself physically active?

Respondent: Becoming more so. Up 'til now, no. I had post-natal depression with my second child so my sort of world contracted a lot with that. So energy and motivation was a bit of an issue there for a while for me.

Interviewer: It would have taken quite a lot… it would have taken a lot out of you. It would have taken a lot of time, too.

Respondent: Yeah and not having a history of being physically active either… but

Interviewer: Having kids is enough.

Respondent: Yes but I have learned that you know in terms of managing stress, exercise is a good thing. Still struggle with the motivation at times but I've also just started Weight Watchers.

Interviewer: Right, OK.

Respondent: And that's been a very good thing in terms of just being aware of what I nourish myself with food but also my activity level. So that's been giving me some sense of check and balance so I'm becoming more physically active and more aware of the importance of it and doing it within the day.

Interviewer: OK.

Respondent: So that might be that you know I can go two different routes in the [workplace] but if I go one way I go up multiple flights of stairs.

Interviewer: OK.

Respondent: So physical activity for me would be more breaking things down into parts.

Interviewer: Yeah.

Respondent: So that's still trying to be more physically active in a day without you know going to the gym so much.

Interviewer: OK yah I understand that.

Respondent: So that…

Interviewer: And we'll get plenty of opportunity to talk a bit more about exactly you know the different parts of your life and what you're doing. Where does physical activity fit into your life? Is it a high priority or an interest or something you've got no interest in?

Respondent: I'm starting to turn that around as being an important part. And when I… an example of that is when I look at what I need to do for the day. One is around you know I've got a column that looks at what I need to do in the house, I what I need to do for the kids, what I need to organise for work. But another column is me and usually I put exercise on that.

Interviewer: Oh well.

Respondent: So, try to incorporate that within the day.

Interviewer: That's interesting. OK, that's a good idea, excellent. OK, that's a very good idea. So, OK just to look a bit more at it, in our research we think about physical activity as any activity that lasts for at least ten minutes. That's the evidence for health benefit. But it's anything that causes your body to work harder. Warming up, huffing and puffing, heart rate.

Respondent: Yeah.

Interviewer: OK, might go up. So, thinking about that type of physical activity over the last two weeks.

Respondent: Yeah.

Interviewer: And then I'll sort of ask you questions about different parts of your life.

Respondent: Yeah.

Interviewer: OK. So in the past two weeks have you been active at all at work for at least ten minutes at a time?

Respondent: Yes.

Interviewer: Can you tell me what you did?

Respondent: That would be taking that route with the stairs and going that bit further.

Interviewer: Yes. And would that, in terms of intensity would that be sort of steady or moderate or vigorous?

Respondent: It would be steady or moderate.

Interviewer: And the frequency? How long… how often would you do that?

Respondent: Well I work two days a week so each day I work I try to do that.

Interviewer: OK and how long would you do it for?

Respondent: Each time? It would probably… it would be close to the ten minutes.

Interviewer: Yeah.

Respondent: Maybe sometimes a couple of times a day.

Interviewer: And OK so that's during the day and you usually do that. Does the situation change at all depending on the season or the time of the year?

Respondent: No.

Interviewer: No, because it's something that you can do at work.

Respondent: Yeah.

Interviewer: And if you wanted to is there anything that would have helped you be more active at work?

Respondent: If they had a gym at work it would be very enticing to finish up and have half an hour there. The uni is very good they do offer… you can do free swimming laps. But it's sort of between 6.30 and 8.30.

Interviewer: OK.

Respondent: So family is a bit prohibitive of that.

Interviewer: Yeah it's a bit hard. Yes, because how long does it take you to get over to [city] isn't it?

Respondent: Yeah it would lake about 35 minutes.

Interviewer: Yeah so that on top if it, yeah.

Respondent: Yeah.

Interviewer: Yeah, OK. So you're looking like what would help is something that within you work day you could get there.

Respondent: Yeah.

Interviewer: OK… in the past two weeks have you walked or cycled for at least ten minutes at a time to get to or from a place?

Respondent: As in a deliberate sort of thinking about doing a walk? I went around [event] and that was to be with the kids and to do that but it was also an awareness that it's a very good physical activity. So I'd say yes to that.

Interviewer: Yeah, and the intensity? Sort of steady or moderate?

Respondent: Steady to moderate.

Interviewer: Yeah and how long?

Respondent: That was about four hours.

Interviewer: Yeah it was quite a stint, I imagine.

Respondent: Yeah.

Interviewer: It looked really interesting actually.

Respondent: Yeah.

Interviewer: So that was… and the frequency?

Respondent: That would have been well just the once.

Interviewer: Was that over a couple of days tough?

Respondent: No just the one day.

Interviewer: You just went the once yeah, over the four? Yeah OK and when did you do it? Was it before, after work or weekends?

Respondent: That was on a Sunday… Saturday, sorry.

Interviewer: And where? The [event] was at [village] wasn't it?

Respondent: At [village], yeah.

Interviewer: And were you alone or with others?

Respondent: With others.

Interviewer: The family? And is this what you usually do or is it a one off?

Respondent: More so it's becoming more that we would go and take the kids with the bikes. But probably frequency we might get there once a month maybe, once every three weeks.

Interviewer: So where would you take the… go bike riding?

Respondent: Well they have just built a bike track from [x] Beach to [town].

Interviewer: OK so you bring your bikes down?

Respondent: So we get in the car and bring them down.

Interviewer: Yeah.

Respondent: So that's been good.

Interviewer: Excellent OK. I'm starting to hear quite a bit about this bike track and walk track. Yeah.

Respondent: Because it's somewhere that you're not having to walk in and around cars an…

Interviewer: Yeah I know.

Respondent: So it's good. Certainly we've done more bike riding since that's been there and walking.

Interviewer: That's interesting. So how long has that been for you that you have noticed a change? I don't know how long that's been operational for so you'll just need to tell.

Respondent: Goodness, I don't know, you'd need to check that. Probably in the last 12 months.

Interviewer: OK and so when… how often would you go bike riding as a family?

Respondent: It's probably about every three weeks or four weeks or something.

Interviewer: Yeah and does this change depending on the season? The time of the year?

Respondent: Yeah, it would.

Interviewer: Yeah so when it gets colder and it's winter it's less likely to happen?

Respondent: Yeah.

Interviewer: OK in the past two weeks have you been active around the house or yard. Either inside the house or outside the house?

Respondent: Yes.

Interviewer: For at least ten minutes?

Respondent: Yeah.

Interviewer: And can you tell me about what you did?

Respondent: Gardening and making a new garden bed so lots of digging and mulch carrying and things like that. So I've done that probably two or three times in the month on the weekends for fairly long stints, like a few hours.

Interviewer: So you've got room for a pretty big garden where you are?

Respondent: yes for an acre we do.

Interviewer: Right.

Respondent: So there's been level. I've also tried just started putting music on when I'm doing housework because I hate housework. I thought I may as well get a bit of a lift so I try and dance while I'm folding washing or outing away the kids' things. I let everything build up to

usually a Saturday or a Sunday so the frequency is probably… you know when I think of it maybe a couple of times a week for maybe you know half an hour or so.

Interviewer: And would you call the intensity sort of steady or moderate or vigorous?

Respondent: It would probably be moderate.

Interviewer: In the house and out of the house?

Respondent: Mostly in the house… in the garden you mean?

Interviewer: Yeah.

Respondent: That's quite vigorous.

Interviewer: Yeah I thought it might be.

Respondent: And the inside dancing and stuff would be moderate.

Interviewer: OK is there a particular time of day that you did these activities?

Respondent: Housework tends to be morning. And the outside sort of that mid-morning to afternoon.

Interviewer: Do you do that in the week or weekends?

Respondent: The gardening is on the weekends, yeah.

Interviewer: All right and do you do that lone or with others?

Respondent: In combination, yeah.

Interviewer: And does it change depending on the weather and time of year?

Respondent: Not necessarily. Sometimes it's better to work in the garden in winter. If you can rug up… if it's raining obviously.

Interviewer: Inclement, yeah.

Respondent: Yeah but the inside stuff isn't dependant on weather.

Interviewer: No, OK and if you'd wanted to is there anything that could have helped you be more active around the house or yard?

Respondent: Well I've done some… I have done three sessions of aerobics on the TV which I would say is vigorous for about 20 minutes each time. So that's helped. I've just pulled out the old Aerobic Australia Style video tapes because it gives you probably a good sustained 20 minutes. So I've started doing those. And the kids are old enough now that they sort of sit and watch and think, 'How ridiculous'. But… so that's helped. I don't now, in terms of…

Interviewer: It sounds like you've been quite creative.

Respondent: Starting to be, yeah.

Interviewer: Yeah.

Respondent: And I have signed up to do a post-natal fitness thing with a small group of six women. And it's sort of half time in a pool and then half time in a home gym.

Interviewer: Oh right.

Respondent: And just some information around taking care of yourself and physical health. And I like the idea of that because that's on Wednesday afternoon… Wednesday night. It's sort of 7.30 so kids are in bed and it gives me something that I have to be accountable to.

Interviewer: Where would that… where is it?

Respondent: That's only about ten minutes… five minutes down the road in [x] Beach.

Interviewer: Right, OK.

Respondent: So part of where the kids go swimming the woman is offering this which I thought was really interesting. And I do… have gone to the gym in the past and liked that. But I think if you had a friend to sort of motivate you and things.

Interviewer: Yeah.

Respondent: But it's very hard in the winter I think.

Interviewer: Yeah.

Respondent: Getting out and getting so exhausted.

Interviewer: Yeah, it's dark and it's cold.

Respondent: So I think for me exercise really needs to be part of the day generally. Or as a stress relief type thing. Perhaps swimming is something I've

thought about looking at over the winter. And I'm more likely to do walks after work with daylight saving.

Interviewer: Do you have an area around where you are that you can walk?

Respondent: I can but you need to feel pretty up to the challenge because it's fairly steep. You've got a steep hill going down or an ever steeper one going up. So because my fitness level hasn't been great it's been a real arduous task. But yes I could do a little loop around through the village and back up and it would probably take me about 30 minutes which is a really nice little walk. But I haven't tended to do it to make that a priority or to get the energy to do it.

Interviewer: OK that's fine, thank you. Now, have you been physically active for at least ten minutes during your leisure or spare time whatever you may see that might be because I having a very active family I can't imagine you have that much leisure or spare time.

Respondent: In the last two weeks I'd have to answer it's probably three times in that leisure time that I've made that a priority.

Interviewer: So what did you do?

Respondent: That would be the aerobics on the TV.

Interviewer: Yes. OK and also probably the [event] as well?

Respondent: Yeah.

Interviewer: Yeah and so with the intensity of the aerobics, what would you call it?

Respondent: That would be vigorous for me at the moment.

Interviewer: And the duration?

Respondent: Twenty minutes.

Interviewer: And frequency was…

Respondent: About three to four times in the last fortnight.

Interviewer: And during… you did it during the day. Where? At home. Who with? Yourself.

Respondent: Yeah.

Interviewer: With the kids supervising.

Respondent: Yeah.

Interviewer: OK and does it change depending on the season or the time of the year?

Respondent: No.

Interviewer: Because it's something you can do inside. And if you wanted to is there anything that would have helped you be more active in your leisure spare time?

Respondent: I don't know, if somebody had come and said, 'Look, I'll have the kids for half an hour, go for a walk', maybe. Because it just seems too much of an arduous task to put them all and pack them up and get them I there and then you have to carry a bike and then they're hungry so I think for me maybe that might have been a way to go.

Because I love walking on the beach and I haven't done that. So it's interesting, it's a good check and balance for me. My question is, 'Well why haven't I done that?'

Interviewer: And how far would the beach be for you? Obviously it's a drive but…

Respondent: Only about five or ten minutes.

Interviewer: Sounds like you're well places at [village].

Respondent: Yeah.

Interviewer: OK so we're now going to talk a bit more about physical environments. We've talked about where you live and physical activity. So this is about talking about physical environments where you live and work and play. And so when we talk about physical environment I actually mean the physical things that surround you. The roads, the trees houses, shops, you mentioned a village. Traffic lights, all of those sorts of things. Now some of the things… some of these are things that people in urban areas have said sort of are important for physical activity. So we'd like to sort of see whether they're relevant to you. Now, can you tell me first of al a bit about how easy or difficult it is to be physically active in your own area, your community?

Respondent: You mean in terms of walking or in terms of activities offered or…?

Interviewer: All of the above. What you want to do, how easy is it to do it, in [village]?

Respondent: In [town]? I really like Tai Chi so if there was a Tai Chi group in [village]. I have done them… come in to [town] or [city] but I really enjoy Tai Chi and I really enjoy swimming.

Interviewer: So you generally have to travel to that?

Respondent: To that. So you have to sort of factor that into your time away which isn't necessarily such a big thing but I think psychologically when you're trying to fit everything in it becomes more troublesome

Interviewer: So would you call it easy or difficult to be physically active in your own locality?

Respondent: It's probably more difficult in some sense that you'd have to travel elsewhere to access the activities.

Interviewer: Yeah and I guess the other thing you talked about is the work I mean that's building up to, it's quite steep and challenging?

Interviewer: OK so what makes it not quite as easy?

Respondent: I think the availability of activity and the time you need to factor in to get to the place on top of the time that you'd be there. For me like with kids and timeframes. Sort of not fantastic sidewalk things, pathways but it's OK.

Interviewer: OK I was going to ask you about that one yeah.

Respondent: Yeah it's tricky with the pram. I haven't really liked taking the pram down because if I walk down the hill I've sort of got to walk down the steep hill and there's a road right down at the bottom.

Interviewer: OK so when… OK this is… now this steep road is this a side road?

Respondent: No, you come out or driveway and there's a steep one down.

Interviewer: So that's main road?

Respondent: Well.

Interviewer: No, it's not very…

Respondent: It's a bit tricky to talk about main roads in [village].

Interviewer: OK they're fairly quiet roads?

Respondent: Yeah.

Interviewer: They're quiet roads.

Respondent: Yeah.

Interviewer: So they've got a dirt shoulder?

Respondent: Grassy, yeah.

Interviewer: So not footpaths.

Respondent: Once you get down the little hill there's a path that they've just put in sort of recently.

Interviewer: OK.

Respondent: So that follows down and up and that' fine.

Interviewer: OK.

Respondent: But pushing the pram down that hill there's a road that runs along the front.

Interviewer: OK.

Respondent: So I've always been panicked if I lost the… let go of the pram or something it would go straight across so it's prohibited me doing that. So what I have been doing in the past is I drive… get in the car, load the kids up, drive down to the bridge and then sometimes walk [daughter] to school with the other kids in the pram, get back in and come up. Because that bit of hill was stopping me.

Interviewer: That's a fair comment.

Respondent: So yeah. But still you can sort of work around it. It was hard to get the kids in thee especially when they're little and get down but there was benefit.

Interviewer: So as they're getting older.

Respondent: Yeah it might be easier. So that little bit of traffic come up… it's a bit tricky yes and the other way is quite steep. And you sort of get you know tractors and harvesters and things like that and the sight distance isn't good along… beautiful view but your sight distance and there's no pathway… no sidewalks up the top road.

Interviewer: OK.

Respondent: But it's gorgeous.

Interviewer: OK so there's no sidewalks… the top road. Going down the road theres' some footpaths that you can access that goes through the village itself?

Respondent: Yeah.

Interviewer: And it goes to school and…

Respondent: Yeah.

Interviewer: OK, good. OK now we're going to talk about a number of aspects of it and availability and accessibility is the first one. And paces to be active. Now are you warm… not too hot?

Respondent: No, I'm OK.

Interviewer: OK if I were to ask you about places to be active in your community what sorts of things would you tell me about?

Respondent: Well, there's the tennis court. And there would be a tennis club. Not that I've investigated that. There's a football club. And that's as much as I'm aware.

Interviewer: Any parks?

Respondent: There's some play equipment that backs onto the… a bit like a reserve, I guess near the football oval.

Interviewer: And OK and access; are these places convenient? Are they easy to access in terms of opening or cost or whatever?

Respondent: I haven't investigated. I would assume reasonable, yeah.

Interviewer: OK and does having places to be active influence whether you are active or not?

Respondent: I think it would.

Interviewer: Because it sounds like most of the places you go are further out than [village]. There's something you can do in [village], locally, the walking but… and those places aren't too far away. They are however, as you said you do have to add in the drive time.

Respondent: But I think you know if they had badminton or if they had Tai Chi or if they had aerobics or something and it was… I think that would really make it easier for me to access and more likely to.

Interviewer: Yeah. And if you wanted to be more active are there things or places that would help? That would be more available and more accessible? That's exactly what you've just said. Is there anything else you want to add to that?

Respondent: Also you asked the question about neighbourhood or community I think those things would build a sense of community. Especially among women. So in an ideal world if you have a good sized hall or something and somebody that's taken the initiative or funded to provide you know a couple of different whatever. Then they would all create a meeting place, especially for women. So I think that would have the dual effect of increasing physical activity but also connections amongst people. So I think that would be an interesting concept in a rural community and whether that will be taken up.

Interviewer: Yeah. And some people would just be interested. Some people would be interested because it was part of their local community. Yeah,

good comment. Now, destinations. If I were to ask you about places you could walk or cycle from your home, what sort of things would you tell me about? Like shops, schools, services friends.

Respondent: I'm not quite sure I understand the question.

Interviewer: OK so if I was to ask you about places to walk to.

Respondent: Yes.

Interviewer: Or cycle to.

Respondent: OK, yeah.

Interviewer: From your home?

Respondent: Where would they be and what would they be?

Interviewer: Where would they be?

Respondent: They'd be the school. They'd be the corner shop. And possibly the park. Well it's not really a park but the play equipment that's there.

Interviewer: OK. And does having destinations influence whether you are active or not?

Respondent: Yes in the sense that like I've talked about before. Incorporating physical activity in what you have to do during the day rather than additional to.

Interviewer: Yes, good point.

Respondent: So it does, if I've got to go out anyway, to take [daughter] to school and I'm organised enough and it's not too cold then it's more likely that I would try and be organised to go earlier so I could walk. So yeah, I think that concept of integration into activity - normal everyday activity does increase the chance that you'd do it.

Interviewer: And if you wanted to be more active would it help to have mote destinations within easy walking or riding?

Respondent: You mean… I guess if people are living in town they've got lots of destinations. They could take the kids to the library. They could take the kids to…

Interviewer: When you're talking about town where are you…?

Respondent: Which I would say [town] where you've got the services and things.

Interviewer: Yeah but you're living in [village] so that's a bit different.

Respondent: Yeah so a lot of destinations I go to are further than that.

Interviewer: Yeah.

Respondent: So it's in to the bank or it's in to [town] or in to [city]. So yeah.

Interviewer: So there's nothing that would sort of help you locally with destinations?

Respondent: Only in terms of… say if the re was Tai Chi class on a Tuesday night in daylight saving time then it's a destination but it's an activity but I'd be more likely to walk down there, do the Tai Chi and walk back.

Interviewer: Yeah fair enough, fair comment.

Respondent: Now let’s look at neighbourhood design which is sort of… it’s the built or man made or non natural features of your community. And that’s when we start talking about footpaths and lighting and roads and walkability. So if I were to ask you about those, what sorts of things would you tell me about? About your local community?

Respondent: I think the council’s in a bit of a mess around [village] because there’s the rhetoric that they’re trying to keep it as a village so the mindset…we can’t get out mail delivered because there’s a shop down there that has historically taken the mail so the mail gets delivered to the shop. And the argument was, ‘We want to keep it as a village’. As having you know that central I guess meeting spot sort of the old fashioned type of thing, which is very frustrating because I have to get a mailbox in [town]. Because the shop is a bit of a mess out there itself and it’s very busy and chaotic.

Interviewer: So it’sore accessible for you to choose.

Respondent: Yeah so they have a concept out here of keeping it as a village because it’s sort of an old sort of area and more rural. But I think they’ve got a tension with doing that than having services that work. Do you know what I mean? In terms of footpath and in terms of actually planning and growing. I don’t see a lot of evidence of how they’re inputting to the area. I think they’re confused in their aims. So I think that’s tricky because if they did have a walking track… because you can only go to [village]. You can’t really keep going through to [x] Beach and [town]. Because the road has no footpath once you get really much past the shop. So there’s a big gap of windy road.

Interviewer: [Town] would be your closest next locality?

Respondent: So if I could get to [Town] from [village], then you can get on the [town] bike track to go to [town].

Interviewer: Right, so it would connect up.

Respondent: But the road… I walked it once and it was terrified. Because it's sort of a windy road. It's narrow and you get log trucks and you get all sorts of you know heavy farm equipment and there's no place for you to get off the road. So you're limited by what…

Interviewer: So there's no decent shoulder at all?

Respondent: No and no footpath.

Interviewer: No footpath.

Respondent: So I think planning; if they're looking at making more active communities then they need to look at how…

Interviewer: Link up to other things.

Respondent: Yeah.

Interviewer: And how far is it again from [village] to [town]?

Respondent: They would only have to probably put in about seven kilometres of footpath or something to link the two. And it's the same… upwards to [city] or [village] there's no footpath. But again for about ten kilometres you could then hook in to the bike track at the [x] River railway which would take you right the way through [city]. So you are

isolated by services such as footpath and you know tracks that are walkable.

Interviewer: It's one of the discussions that need to happen just now.

Respondent: Yeah.

Interviewer: I mean I think it's almost like this is the first stage to look at like obviously bigger communities and these tracks but then it's sort of looking at well how do you then start to link the next outer communities with that?

Respondent: Yeah.

Interviewer: At the present moment your only option is to put everything in the car and river there.

Respondent: And especially as women with young children and if they're looking at mental health issues for rural women in isolation…

Interviewer: It presumes you have a car.

Respondent: Yeah, that's right. So I think it would be really interesting to see what the council vision is and what their understanding is of the needs and whether it's even on the radar.

Interviewer: Interesting.

Respondent: That's a long answer way to answer that question but…

Interviewer: That's an interesting one. It's a very important issue. OK do built or non-natural features, man made features in your community influence whether you're physically active or not?

Respondent: The roads, yes,

Interviewer: Yes because you've just been giving an account of them and if you wanted to be more active we've talked about that and what would help. OK?

Respondent: Yeah.

Interviewer: Now for the natural part, the aesthetic qualities or the… if I was to ask you about the aesthetic qualities or the attractiveness of your local area what sorts of things would you tell me about?

Respondent: It's gorgeous. Beautiful. Big old trees, really nice buildings, river. You know you can see the patchwork of all the farming land. Glimpses out to the sea and mountains out the back so it's absolutely stunning and soulful. That's the biggest thing for me to actually want to get out and going is to look at all that.

Interviewer: Yeah, it just draws you out.

Respondent: Yeah.

Interviewer: OK obviously 'Do you think thee environment is aesthetically pleasing?' 'Yes' and nearer home, do the aesthetics of your neighbourhood influence whether you are physically active or not?

Respondent: Yes.

Interviewer: And if you wanted to be more active are there features of your neighbourhood area that you might… might help you be…?

Respondent: The bike track or a walkable path.

Interviewer: Yeah so you could actually enjoy it a bit more.

Respondent: And a building or facility. A very basic sort of… someone that would coordinate a couple of activities. I'd be more inclined to do so.

Interviewer: Yeah and you're talking about that and also some connectivity as well between things that are already there as well. That's good, OK just one more area. Safety; if I wanted to ask you about safety in your community, what sorts of things would you tell me about? I think you've touched on some of this in terms of road safety.

Respondent: Yeah, there's road safety issues. Tractors and all sorts. Depending on… I probably wouldn't walk down into town of a night. There's a pub right on the corner. So you get everyone spilling out onto there and farmers' with lots of alcohol in their system is not particularly pleasing to walk past. So yeah so that's something that I would be aware of. I wouldn't walk of a night generally. And the steepness… my ability to take the kids in prams or bikes are really limited in [village].

Interviewer: Yeah and I guess it would be a question of not only you know your personal safety and road traffic safety but your visibility and lighting and all sorts of things.

Respondent: Yeah.

Interviewer: Because I presume it would be happening in the village but to get there.

Respondent: Yeah.

Interviewer: Does personal safety influence whether you are physically active or not? You've said, 'Yes, at night'. And does road safety influence whether you are physically active or not? You've said, "yes, particularly at night and generally'. If you wanted to be more active are there any personal or road safety issues that if they're addressed might help?

Respondent: You could walk in a group. I've often thought about… because my background is in social work and in palliative care I've often thought about starting up a walking group. Or a collection of people that could come along id they've had grief or trauma in their lives. It's some way that they could just physically connect and walk.

Interviewer: Just be.

Respondent: Yeah. So… yeah I think so.

Interviewer: So that's one thing. OK, thank you. And just a last question, before we finish up is there anything else that you'd like to tell me about; where you live, physical activity or your physical environment that we haven't covered today?

Respondent: I think family plays a large role.

Interviewer: Yeah.

Respondent: And I don't know if that's captured in your study or not in terms of people being active or wanting to be active but unable to be. I've found that really quite challenging. There's so much to do in the day and if you're working and you're trying to a balance those things.

Interviewer: Yeah it's enough of a balance trying to deal with all of that. With children all going in different directions for different stages of their life and development.

Respondent: So I don't know, my experience has been that when you start to have a family your whole personhood changes. That you probably lived a certain way pre children and had a certain view of yourself and lifestyle and that gets a sudden shake up when you have children. And I've always wondered if you're looking at women and you're looking at health it's quite a tipping point. There's something fairly charged at that transition time of becoming a single person to being a mum. Do you know what I mean? Or a married person into a mum.

Interviewer: Yeah when you go from a single into a multiple part of something…

Respondent: So for me if you're trying to integrate health and physical health and activity like embedded into somebody's lifestyle then the best way to do that could be at that juncture and look at it from the point of view of health but then also mental health. And there's so much that's done in maternity and it's about the birth and it's about how to put a nappy on and how to breastfeed but all the other aspects of who you are as a woman and how you're going to live your life and stay well for you family aren't addressed. And I think that's often… if people… I'm not sure how people have been, if they've been really physically active and gone to the gym and do all that before kids I'm not sure how they transition that. So for me and I also know that you've got a cohort of women that don't have children so I'm not sure how that's captured.

But for me there needs to be something within our health programmes and information around how are we going to do that? Because what I had to learn, especially with the post-natal depression was breaking it down. So I couldn't do 45 minutes in the gym but I might be able to put the music on and dance for five minutes. So I guess it's changing that paradigm. That's the only thing I would think to add in to it.

Interviewer: Yeah that's really good thinking yeah. Because you're right. It's sort of like people are just left to sink or swim, basically.

Respondent: Yeah and you're looking at health and you're looking at wellness.

Interviewer: It's really an area that's a really important one.

Respondent: And how you embed it into every day rather than the thing you do at the end of the day if you've got time. Which was always my concept probably beforehand.

Interviewer: And not only for yourself bit it's obviously it's a good learning for your children anyway. I mean this business that you know we regiment our lives in to…

Respondent: So I think that… having just come out of that experience I think looking back at that…

Interviewer: There's a lot of learning to be… that sounds like a really positive learning to take back into health too.

Respondent: Absolutely.

Interviewer: But I agree with you. My background in health is much more from a preventative and wellbeing one yeah. I've been a social worker too. I've been a community worker in rural areas but… and that's the area that always interested me because it was much more on women's health and well being so it was getting communities together to have a look at what they needed.

Respondent: Yeah and I think you even need to go back earlier. Because e it's not part of the school curriculum. You learn how to do this but you don't actually learn how to take care of yourself so that when you do hit rough times in your life that you've got those strategies that have already started to grow. I don't think we do that for young people generally.

Interviewer: I agree with you. I don't think there's those sorts of inbuilt supports at all.

Respondent: So I think there's a real challenge for the government looking at how do you… because we know what's happen if people aren't physically active and take care of their health in terms of what that means for the health system and their length of life and quality of life and caring issues but I don't think we've got it embedded early enough.

Interviewer: No because as we're talking about it here's that aspect of it but earlier it's about enjoying the fullness of your life and if it can… our health and wellbeing is really essentially a part of that that keeps you in good stead.

Respondent: Yeah but I'm sort of learning that at [age].

Interviewer: Yeah.

Respondent: It would have been good to… and maybe there's certain times you're more aware of it but I think it's the integration in your daily life and the importance of it.

Interviewer: I think it's really hard and I think this whole business about health and focussing on that. I know we have to have good emergency department staff but there's been a trend that it's meant that they've got to cut funding in the sort of…

Respondent: The community stuff. Yeah and I think it has to go back that other way.

Interviewer: It's going to catch up again, yeah.

Respondent: So it will be really interesting…

Interviewer: But it's no use going around pointing the finger and… you're diabetes or you're obese or…

Respondent: No.

Interviewer: Whatever that is anyway.

Respondent: So it will be really interesting to see what comes out of it and whether council have any investment into it too.

Interviewer: Well you see council are really… have come forth as being a very big payer.

Respondent: Fantastic. And it might be in the town planning that they may need to have a diverse stakeholder group.

Interviewer: What it's coming… what it really means is going to be interesting.

Date: 3 November 2011

Duration: 49 minutes, 55 seconds

Interviewer: [interviewer]

Transcriber: [transcriber]

So I have a range of questions that I'm going to ask you about, and some of them may or may not be applicable to you. We'll see how we go. There may be things that you mention earlier that I ask again about later. But we are just trying to you know, cover off on everything. So we'll see how we go. So as I mentioned I'm really keen to hear your thoughts on a range of issues around physical activity and where you live. Remember there is no right or wrong answers. I'm just really interested in your experiences and opinions, thoughts and feelings. If you are unsure about anything don't hesitate to ask. And if you don't want to answer any questions you do not have to, and you can stop the interview at any time if you need to. So I thought, my first question today is about, talking a little bit about the place where you live. So I just wondered if you can tell me what it's like to live around where you live in your area?

Well, firstly I live on the main street of [town] town. So in the sense I live in the CBD of a very small town. And I live and work on the same premises. And yeah, I don't think of myself as living in a rural area. Although for demographers I suppose I would be.

I think for demographers all of [state] is considered rural, so there you go it's all relative isn't it?

Yeah. Ah, there is one good thing about [state], particularly the [region], ah is that you have a, instead of having like [city] is basically one big populated area, ah it's much more diverse here, spread out. So you are not very far away from what I think of as rural.

OK. Sure. Yeah, sounds good. And so in terms of the property that you live on, is it sort of a standard block or have you got any sort of acreage?

No, no acreage. Ah it's yeah quite a small block.

And can I ask how long you've lived there?

Ah, since 1989, so what are we looking at? Twenty two years.

And what brought you to [town]? How did you end up living there?

Well first of all before that I spent four years living south of [city] in the [region] area.

Oh beautiful.

And one of the reasons why I moved to [town] was that the climate is much more pleasant in [town]. We have olive groves to the south of us. I have a friend in town here who can grow pineapples without a glasshouse.

Wow.

Yeah, one of the good things about living, I suppose I should say, if you lived in [city] or west of [city] it's much wetter and windier. On this side, you could say between where I am and [town] it's more a Mediterranean climate. We are cooled by [x] in summer and I think it's because of the mountains we have behind us that seem to make our winters a little bit more pleasant than they are in [city].

Yeah. OK. So it's a bit of a tropical north, north coast.

Just a slight exaggeration.

It sounds very nice anyway. So in relation to the [town] town centre, how far away are you, like ks or driving time?

From the centre of my town?

Yeah.

Ah, well I live basically in the centre.

In the centre. So you're on the main road aren't you?

Yeah, the main street yeah.

Yeah OK. So it's pretty convenient for you to get to the town centre.

Yeah, across the road from me is the [business]. Not that I'm in need of it, but I can imagine for an elderly person that it would be something that you would think of.

Sure.

And I'm within walking distance of shops.

Great. Sounds very cosmopolitan.

Well actually, since I've lived here, I'm originally from [city], and I grew up, my childhood was in the [city] area, and I've noticed the café culture has improved in this part of the state over the years.

Yeah, it's getting better, yep.

I think there is a lot of new blood coming here. And I think their coffee standards are rather high.

That can only be a good thing, can't it? In some ways.

In some ways. Well I don't drink coffee but –

No, neither do I actually.

But for those who do it seems to be an important part of their lives.

Yeah, I can't quite understand it myself either, but yes, I agree with you there. OK. Alright, so we've got a bit of a sense of where you live. When we talk to people who live in sort of more urban areas or cities, they often talk about their local neighbourhood. And I'm really interested in understanding what that word neighbourhood means to you. And for example whether it can be quantified, is it the people, what does neighbourhood mean to you?

Ah, I tend not to think in terms of neighbourhood. I would use the word community.

Yep, OK. That was my next question actually.

Where I live is right on the corner of the street which is a little bit different in that it is zoned commercial and rural – sorry commercial and residential.

Residential, OK.

Yes, residential. … And I meet a lot of tourists as well, a lot of people who are interested in moving to this area. I'm involved, I've been a volunteer for over a decade with the local fire brigade. I do other volunteer work as well. But that's what I mean by community. I don't, my last memory of neighbourhood would have been as a child growing up in a town just outside of [city] where the neighbours all knew each other. I don't have that sort of a life now.

OK. So neighbourhood then was a bit about the people and who lived there. Whereas to now where you live it is more of a community.

Mm. Yeah. I suppose it's because I don't have children. So it's not as if you know, a lot of people get together and they have something in common. So they have children going to the same school, or a lot of mums get together, that kind of thing.

Yep. Sure. OK. That makes sense. OK. So we are going to talk a little bit about physical activity now. So I just wondered what the term physical activity means to you?

Right. Well some of my work and employment would be physical as well. But I take it for most people including me, something physical would be, ah I do Hatha yoga each day. Um, I walk. And yeah, I garden. So I'm out with the fire brigade.

Yep. So do you consider yourself to be physically active? Is that –

As much as my employment situation will allow me. I work 7 days a week. So my, what I would think of, and I suppose most people would think of as physical activity – exercise, I have to do before I start work or after work.

Yeah, and so that sort of limits I guess what you can and can't do?

Ah –

Or not really?

Well it just depends on how early I'm willing to get up. And daylight saving.

Yep. So in terms of physical activity and being active, where does that sort of fit into your life? Is it something that sort of is a priority for you or is it something that you kind of do as part of your everyday, or just something that you don't really think about all that much?

I have been doing yoga and meditation since I was about 5 years old. Pretty well part of my life. I run meditation groups, that kind of thing.

Yep. OK. That's great. Alright. So what I'm going to do in the next little bit is sort of break down your life a little bit into sort of different areas and talk a bit about physical activity that you might do or not, for different purposes. So in our research we generally think of physical activity being something that causes your body to work a bit harder than normal. And your heart rate might go up a little bit. You might be breathing a little heavier. Your muscles warm up a little bit. And it usually lasts for at least ten minutes. I guess that is sort of our research definition of physical activity. So thinking about this type of thing in the last two weeks, um have you been active at all at work for at least ten minutes at a time? Do you do any physical activity at work?

Ah ten minutes at a time, ah I don't think I would call it that. Athough, no, I wouldn't be able to quantify it. Because if I have to, if a customer comes into my show room I have to run from one side of the building to the other. And that is throughout the day. And also because I'm a [occupation]… I have to carry boxes and things like that. So just off hand I can't quantify you know how long that would be at a stretch.

Sure. So it's a bit more sporadic would you say?

Very much so, yes.

And so those types of activities that you were talking about, you know the running from one side of the building to the other and stacking boxes, in terms of sort of how intense they are to you, do you feel that they are sort of moderate or hard vigorous activities?

Ah it's not stressful in that way. I mean the heart is pumping but I have to be able to speak to somebody when the door opens. I can't be too flustered. No, it's not an effort. I'm not one of those people that if they climb a flight of stairs they're out of breath. The only time I would be out of breath, and this is nothing to do with work, is if I find out that I am fairly unfit in that if I go out with the fire brigade and have to run up a hill about five or six times, then I'm very much out of breath.

I think a lot of us would be.

And it has nothing to do with age. Because I've actually found myself, like I'm 45 and there are some guys who are just out of secondary school and they're out of breath. So they're in worse shape than I am.

Yep. And it sounds like hard work. OK. So just one more question or one or two more questions about your sort of activities that you do at work, is it sort of the same most days or does it change a little bit and does it change with the season as well?

It changes with the season because at this time of the year when the weather becomes a bit more pleasant there are more tourists. So really what changes for me I suppose you could say going from just the local customers I end up having some more exotic people from interstate and overseas. So the faces change, the work intensity also changes. So from now until about February March is fairly flat out.

Yeah, OK. And so are you sort of doing more physical activity at work over this time of year.

Yeah, well that running from one side of the building to the other is a bit more frequent.

Bit more frequent, sure.

I'm having to be on my feet a lot longer, so yeah.

OK. I'm going to ask you a question which might be a bit irrelevant to you because it sounds like you are already quite active at work, but I'll put it out there anyway: so if

you had wanted to, um is there anything that would have helped you to be more active at work?

There's nothing I could have done as far as work goes. It's what I do outside of work that has a bearing on my overall fitness.

Yes, sure OK. And we'll talk about that in a minute. I didn't think that one might be all that relevant for you. OK, so moving onto another sort of type of physical activity, in the past two weeks have you walked or ridden a bike for at least ten minutes to get to or from places? So to get say from your home to the supermarket or to get to somewhere, to a certain destination.

Yes. I've walked.

You've walked, yes. So do you want to tell me a bit about where you go and why you did that?

Well, ah because of necessity. You know, having to buy groceries, fresh fruit and vegetables and the like. It just means that I have to physically walk from my home business to the shop and I carry the shopping back with me.

OK. Yep.

So there's the walking and I suppose the weight lifting side of things.

Yeah, added exercise. Are there other places that you might walk or ride to?

Well it's a bit too far to actually walk outside of the town, like behind me are mountains called the [x] Range. Now, if I rode a bike I suppose I could go into the [x] Range, but it's just a question of, as I said I work 7 days a week, so I fit in the physical exercise around either side of working hours.

Yep. Sure. So getting back to the walking for the supermarket and that kind of thing, do you tend to, is that sort of a regular thing or just every now and again a bit ad hoc.

Well I would be walking every day, and definitely for at least ten minutes because it would take me, let's see, around about twenty, if I was to walk from where I am to the last shop where I would go, it would take me about 20 minutes.

To get there?

No, no, no.

Round trip.

Round trip, yeah.

OK. And sort of what time of day would you normally do that? Is that a before or after work thing or during the day?

Well it would sometimes be, well it can only be, it can't be before work because I open at 9, and there are very few businesses that open before 9 unless it's the supermarket. Ah yes, so it would be after 9 and possibly – I close at about 4.30 and so sometimes after 4.30.

OK. And where did you do that? That sort of walking is there like a footpath or a cycle path or something that you go on? Is it just on the main roads?

Yes it's just along the main street, so you could say a footpath.

Yep. No problem. And do you usually do that on your own or with someone else?

On my own, because it's usually considered a chore.

Yes. And does that one change depending on the time of the year, whether you walk or not?

Ah the time of the year isn't the issue, it's just the weather on the day. But just because it's raining doesn't mean that I won't actually walk. It's just that if it is ah, well I'm like everyone else if it is a journey where I have to purchase more than I can carry, well I have to take the car.

Yes, OK. So that's a more important factor than say whether it's raining or a bit dark outside.

Yeah.

OK. And if, again I'm going to ask this question – it sounds like you are doing this a fair bit anyway, but if you had wanted to, is there anything that would have helped you to use more active forms of transport, like walking and cycling to get to and from places?

Ah, no I couldn't have done any more.

OK. No problem. Alright. Now we're going to think a little bit about activity around your house and your yard, inside and outside, for ten minutes at a time. So thinking about that type of activity, did you – this is sort of outside, I know it is a bit hard for you because you have got your work at your home, residence as well, but thinking about outside of that sort of 9 till 4.30 work time, um have you been active around the house or yard, sort of doing chores and things?

Oh yeah.

Yep. OK do you want to tell me a bit about it? What you do and why, and where and when?

OK. Well if you're talking about, what sort of period?

Like in the last two weeks?

Oh well it's been extra busy because I'm landscaping the garden on the property. So I've converted what on one side of the building – see most of the land that my house is built on is rather odd. There's no, there's not what you would call a backyard or a front yard or anything like that. Basically the house is plonked in the middle and you've got two gardens on either side. And on one side I've created from what used to be a native garden jungle which got out of hand, I've started again and I've created something that is a mini sort of a zen garden. I've replanted seedlings. I've had to get rid of a lot of ivy which is one of the most strenuous things I think anyone can do in a garden.

Yeah, it's hard work pulling that out isn't it?

Yeah I mean getting down to those roots and making sure that there aren't any left. And now that side of the house is sort of on the mend. I started on the other side of the house and there is even more ivy. And I've been having to basically start building footpaths. So I've been shovelling gravel and then I'll be adding paving stones later.

Wow. So you've been doing a fair bit of activity around the yard in the last week or two.

Yes. So as soon as I clock off after 4.30 or whenever, then as long as I've got daylight I'll be out in the garden doing that.

Yes, sure, OK.

So yes, some people arrive early. So it would be sort of every, well if you were talking about let's say in the last week, it would have been about three days out of seven.

Yeah OK. And you were doing that after work aren't you? You are not doing that in the morning.

Yes.

Yep. And are you generally doing that on your own as well or have you got someone to help you out?

Ah, sort of in shifts. I have my Dad around on occasion to help.

Oh good, OK. And so apart from the garden are there other things that you might do say inside the home that meet that definition of physical activity? You know, ten minutes at a time?

Oh well, yeah I suppose you can count things like vacuuming. Ah what else is there, ah climbing up and down ladders and putting batteries in and out of smoke alarms and things like that?

Yep, yep. And those sorts of things, the vacuuming, is that a sort of a weekly, fortnightly, monthly kind of thing?

Yeah, a weekly thing.

Yeah a weekly thing. Alrighty. And sort of how long would that usually take you?

To get from one side of the building to the other, it would fit within that ten minutes.

Yeah, OK, good one. Alright. And again I'm going to ask this question about um, it sounds like you are doing lots and lots around the house already, but if you had wanted to was there anything that would have helped you to be more active around the home or the yard?

Um, basically stop work. Close the business. That's the only way I could have done it. Because as I said it's a case of the customer is always right. So, they call and I have to run.

Yeah, no problem. Alright, onto our last sort of I guess domain of physical activity. This is the sort of physical activity that you do during your leisure or discretionary time, your spare time. Although it sounds like you don't have much of that, but perhaps this is where your meditation or yoga – well I'm not sure, maybe you can tell me about what you see as physical activity that you might do in your leisure time.

Well, do you classify something that might be considered as sedentary, meditation as something that would give health benefits? Because I know it's debatable amongst western scientists. Because you know mental wellbeing is an aspect of overall physical health.

Yeah, absolutely. Look I do, I agree with you there that it is certainly good for health. But I guess in the sort of –

It doesn't get the heart pumping -

It doesn't get the heart pumping and I guess in terms of cardio respiratory fitness it's probably not doing a lot there, but certainly in terms of you know mental fitness, if you think about it that way, I think it's –

Because I combine that with Hatha yoga, that – are you familiar with Hatha yoga?

No. I've done, I know Iyengar yoga, um but –

Yeah, well basically Hatha yoga is you could say that what most people think of as yoga in the west, so you could say it's the physical aspect of yoga with the spiritualism stripped off that. OK, so in a sense that gets the heart pumping because there are weight bearing exercises and postures which get the heart pumping.

Yeah for sure. So how often, did you say you'd do your yoga every day?

Yeah, every morning. Every morning and well I certainly in terms of physical activity, rather than just meditation, yeah the Hatha yoga is a morning activity.

OK. And sort of how long would you spend doing the yoga in the mornings?

Ah, most days it would be at least half an hour.

Yeah, right, wow. Um, and what was I going to – and you do that at home?

Yeah, that's right.

Yeah, OK. And again is that one just an activity on your own?

Yes.

And it sounds like that is sort of your regular routine, it's not a one off is it?

It's a daily activity. Because for one thing if I didn't do it I would have trouble holding the physical postures I do for sitting meditation. So in a sense it helps with flexibility.

Helps with that. And does that change at all according to the season?

Ah, that's a good question. Because as I'm getting older I've been developing a little bit of arthritis. So now I understand what it's like for older people when they start talking about their ailments. So when there is a change in the weather or something there is a little bit of physical discomfort.

Yeah, OK. So does it sort of stop you from doing it or do you just alter your routine?

Well I have to alter the routine. So I think it's that I'm learning at the age of 45 what if I should live into my 70s, 80s or beyond that I will just have to adjust to my situation.

OK. And do you want to tell me a little bit more about your volunteer fire fighting? You were mentioning running up and down the hills and things. Is that sort of a regular thing that you do, the physical side of that?

Ah well the brigade trains every second and fourth [day] of the month, so I don't think that would be regarded as frequent. And as far as being called out to incidents, we would be, compared to [city] or the like we would be regarded as a fairly quiet brigade. So yes, it's fairly sporadic.

Sure. And is there anything else that you do that you wanted to tell me about that we haven't talked about in terms of your sort of leisure or discretionary time?

Ah not if it is in terms of the cardiovascular exercise.

Yeah, that's probably what I'm – you know I'd love to hear about everything else, but um yeah that is what we are sort of focussing on at the moment. Yep.

It's a shame really, because I wish the research was a little bit expanded into the area of mental wellbeing as well. Because I've taught meditation to terminally ill patients and psychiatric patients in private practice, and it yes, it does marvellous things. So just focussing on just the physical exercise is missing out on, it's sort of missing out on half of the deal really.

Half of the story isn't it? Yeah, oh that, yeah that's a shame. Um, but yeah I'd love to talk about it sometime with you but I don't want to keep you all night so um -

That's alright.

We'll stick with our little program that we've got here. But yes I certainly acknowledge your point. Alright. So we've talked a lot about your physical activity and sort of your home and life and that kind of thing. So what I'd like to talk about now is a bit more about the physical surrounds, so your physical environment. So what I mean by that is all the things that are around you. Whether it be man-made things like roads and houses and shops and traffic or whether it's trees and nature and open spaces. Some of the things that I'm going to ask you about are what people who live in cities tell us are important for their physical activity, but again I'm really interested in how that crosses over or not to people living in more rural or regional areas. So I guess my first question is whether you can tell me a little bit about how easy or difficult it is to be physically active in your neighbourhood or in your area?

OK. One of the issues, even in a small town where I live, is that I would like to extend the areas that I walk in. But I'm prevented by the situation with roads and the lack of footpaths.

OK. Do you want to tell me a bit about that?

Well there are footpaths along the main street of my town, just as there are in many rural towns, but when you start going further afield like into like in residential areas for instance, you don't always have continuous footpaths. And if you wanted to go even further, because I mentioned to you that there were mountains behind us, um I'm prevented from doing that because really I would have to walk on roads which is not particularly safe. Ah so with what we do have, compared to some of the – like [city] for instance – I suppose I'm fortunate. But I would like to walk further, but I'm prevented. One side, on the west of the town I'm prevented by the [x] Highway. And in a sense I've got the mountains to the south of me, I've got [sea] to the north of me, and to the east I've got the coast road, so that's another road that I run into.

Yeah sure. And there's nowhere for you to kind of cross over or go along –

So really, if it was on my wish list, I wish I had more variety in the root that I take when I do my walks for fun. So there is a bit of lobbying going on for eventually the whole [region] having a walking / cycle track uninterrupted from at least [city] to maybe [city] or something. But until that's built people like me are fairly restricted in what we can do.

Yeah, OK. That's really good to know. Alright. So we've talked a bit about footpaths and roads and the road network, but thinking about I guess availability and accessibility of places to be active, what sort of things would you tell me about? Are there places you can go to be active in your neighbourhood or in your area?

Ah that I can go to, yes. Do you mean walking distance or using other transport like cars?

Either or. Either or.

OK, well if I'm willing to drive I can do just about anything. Not all of it is available in my town. I might have to go to [city] for instance, which is about you know a quarter of an hour's drive away or [city] which is 25 minutes away. So if there is one drawback to living in a rural area, it's the limited variety or diversity of things, activities that you can do.

OK. I guess thinking about [town] or [city]/[city], what sort of things are available there in terms of you know the recreation facilities or parks or what springs to mind?

Ah well for one thing, although I don't take advantage of it myself, I'm not a member of a gym for instance. I get that exercise here at home. Those who, there's no gym, ah no gym premises in my town. I would have to go to [town] or [city]. That means driving. So for the elderly that creates a challenge. And yeah, bit of an awkward one.

Yep. No, that's fine. So I guess you've kind of touched on this but I guess we were asking whether having or not having access to places to be active influences whether you are active or not?

Um, at this stage of life no. I mean for instance there would have been a time in my life when I played team sports, like soccer for instance. And if my town doesn't have a soccer club then of course I would have to go further afield if I wanted to do that activity.

Sure. But it doesn't affect me at this time of life.

At the moment. Sure. And if you wanted to be more active are there things or places that would help you if there were more of them available or accessible? Is there anything that you would recommend apart from the things we've already talked about? You know if you could have your dream thing that was nice and close and convenient, is there anything else? Or not really.

There's nothing that I could think of for myself.

No, that's OK. That's fine. Yep.

I mean the only thing that I could possibly think of is that more people were interested in doing what I do. You know most people these days outside of working hours, their idea of physical exercise is being a member of a gym. And they, you know, or the treadmill or whatever, and that is their physical exercise. Ah, for me I do it basically at home, so it's not an issue for me.

Yep that's fine. OK. I think we might have talked about this a fair bit already, but I was going to ask you about sort of the, all the different places you could walk or cycle to from your house. We've already talked about the supermarket, is there anything else sort of locally that is within walking distance? Sort of other services, or shops or friends' houses or whatever?

Ah walking distance?

Yeah.

It always depends on time. [town] is flat, where I am, but within, easily within less than ten minutes you could walk up hills. So in terms of visiting friends it's a case of having to hop in the car, otherwise time becomes an issue.

Yeah. Sure. OK. And in thinking about if you wanted to sort of do more activity would it help you if there were more destinations, more places to go within easy riding or walking distance? Or you don't think that would sort of make a difference?

Ah, I think it would make a difference. There is a lot really is to do with, ah less to do with facilities than access, like footpaths, cycle / walkways.

OK. Yep. Well that's exactly what I was about to ask a little bit more about in this sort of next question. So we might move onto that one. So if I was to ask you about some of the built or the man-made features of your neighbourhood or your area, um what sort of thing would you sort of tell me about?

Ah built or man-made. It's basically in low rise commercial residential. Yeah, I don't know quite how to describe it.

Yeah, that's OK.

You don't really have to go far before it becomes residential. All residential. But where I am, I'm right on the edge of a commercial area.

Yeah. OK. And you've mentioned footpaths on the main road, what about sort of lighting and things like that?

Lighting. Yeah, that is something that is fairly poor. Once you go off the main street, street lighting tends to be a low priority.

Yeah OK. Yep.

And I could imagine particularly for women that that would have a bearing on – I know you are doing research on men's health, but as far as personal safety goes, that's an issue.

Yep. We've also been talking to some women actually, earlier this year, so yeah certainly interested in women's health as well. OK and so I guess thinking about those things, like footpaths and roads and lighting. Do they influence whether you are physically active yourself or not?

Ah well when, there are certain times of the year if the lighting is poor, if I can't see where I'm going, I have to walk much later, which means I have less time in which to

walk. So if there was lighting then I would be able to extend the walk. Otherwise I could just do what a lot of morning joggers do, which is to get hold of a headlamp or something.

Yeah, I always worry about those things. They don't show you everything. Um, alright and so moving on to aesthetics. If I was to ask you about the aesthetic nature of your area or the attractiveness of your area, what sort of things would you say?

Ah, absolutely beautiful.

OK.

Across the road from me is a lovely beach, white sand. I have hills behind me and not completely built on. I have parklands around me. Apart from, as I said, the commercial side, of where I'm living. So I've got a couple of – I've got a [business] right next door to me, and a [business] I suppose you would call it, which has just recently closed. And yeah, there are times where not just the look of the place, but there are noise and fumes occasionally. So it's just where I'm located is a mixed area, so there are certain times of the day or certain days of the week where you know it impinges on my quality of life.

Yeah, OK. And does that sort of influence whether you're physically active or not, or is that a separate issue?

That's a separate issue.

Yeah, so it doesn't impact on whether you get out and go walking or whatever.

No, because it's right next door. So even if there was horrible fumes or something that I had to deal with, I could just walk through it all. It's over in a second.

And apart from that immediate area, your broader area sounds like it's very um very attractive and beautiful.

It's just like a traditional country town really, and that's why there is a lot of people interested in moving here. You know, the sea change / tree change thing.

Yes definitely. I can understand it too. OK. So if you wanted to be more active are there sort of features of your area that might help if it was more sort of aesthetically pleasing? Or not really?

Ah, let's see. There can always be a little bit more parkland. But you can't you know demolish people's homes to do that. Um yeah, and we actually had an issue a number of years ago in having some interstate developers coming here. And they

tried to build, um they tried to turn this town into, they were calling it the [x] Bay of the south. So at one stage we were going to have apartment blocks and shopping arcades.

Right. I think I read about that in the papers.

Yeah, well when we had the global financial crisis that put an end to any plans that people had. So now it's just gone back to being a, it's basically a dormitory town where I live. So there is very little employment, large scale employment in the town. So yeah, you would really call [town] a dormitory town.

Yeah. OK. Onto our last kind of area for the day. I was going to ask you a bit about safety in your neighbourhood. And you touched a little bit before on personal safety. So I guess, what kind of things come to mind when you think about safety in your area?

Safety. OK. Well because I have a business I have to be aware of security. Now, even in a small town like mine, there is a little bit of a crime issue. So even in [town] we have an area where there is a housing commission area, a particular street, up the back. And the kids that live there are often single parent families, you know the usual story. Ah it would be much better if we had mixed housing, but that's the way Housing [state] deals with things, that's where a lot of the problem seems to stem from. And so if those people are mobile, as they have every right to be, then they tend to bring any problems that they have there to other parts of the town. And there tends to be, if there is any crime or any personal safety issues, it tends to be more opportunistic. So if you are foolish enough to leave your car doors open or you leave a nice pair of sunglasses on the driver seat, you are asking for problems. But that's the same everywhere.

Sure. OK. And I think that's a bit about I guess personal safety. Were there any other like road safety issues for example?

Well road safety isn't an issue right where I am. As I told you if I was foolish enough to walk – the main street of this town is what is the old [x] Highway. So it's still a reasonably busy road. And if I go to the west of the town the new [x] Highway borders it. So that impinges on you know sort of the distances I would like to cover. I mean I would like to get back into cycling. I used to do road cycling. But, the infrastructure doesn't really cope well for the lone cyclist.

Are there any sort of, is there anywhere you can cycle sort of on the side of those roads? Is there a cycle path or a cycle main or anything?

Ah there, it's very difficult. There are in places, but you actually have to get onto the highway to get to those. And if you are talking – that's the highway, but if you are talking about the old coast road, well there's nothing like that. There are no footpaths once you get outside the town.

OK. So you would sort of be taking your life in your own hands going on the old highway.

Mm, in a sense you are. I mean um I suppose I'm sort of wimping out in that way. But the issue that I have is that even on a bike, even though I can actually go a great distance, to get the health benefits I can't go far enough. You know the difference between cycling and walking is, with walking you don't have to cover the same difference. It's more a case of you know getting the heart pumping.

Yep. That's right. That makes sense.

But with cycling of course you've actually got to go further and the terrain has got to be a bit more challenging. Um, there was one thing I wanted to add which I should have done earlier.

Yep, sure.

The only other safety issue that we would have in this little town is um there is a problem with alcohol abuse on certain nights of the week. There is one particular pub which is within about three minutes walk away from where I am. So I can guarantee that every Friday, Saturday night no matter what the weather is like, you are going to have, particularly young people, drunk and potentially violent heading to and from the pub. And they are going past my place. So within about the last month, I woke up one Sunday to find the footpath covered in blood. And you know, there was apparently somebody cut themselves. These are problems that you get everywhere, but it would come as a surprise to someone in a capital city to find out that a small town like this had this sort of issue as well.

It's sometimes a bit more noticeable in a small town as well because it's maybe not so common and then when it happens it's quite in your face, it's there.

Yeah, well it's like a little bit like a number of years ago there was a violent murder committed. So, in a place where violent crime is very rare, to have a couple of lives taken brutally, that you know the town was in shock for a long time after that.

Yes. It's quite disturbing I can imagine. OK, so in thinking about personal and road safety if you'd wanted to be more active is there any sort of issues around safety that could be addressed that would help you to be more active? Or are those things a bit more peripheral? Apart from what we've already talked about?

The only thing that they can improve is by building footpaths and cycleways.

Yep. So it's the footpaths and cycleways and the road safety things. Yep. OK. Well I think we've just about got to the end of our um interview schedule, but before we finish up is there anything else you'd like to tell me about sort of where you live or your environment or your physical activity that we haven't really covered off on today?

Not particularly. Ah, it's just a lovely place to live.

Oh good that's nice. It's nice to like where you live. I think that's a wonderful thing.

Oh but I am like everyone else. You know, the grass is greener elsewhere. If you've lived in one place for quite a long time you do get itchy feet.

Yeah, I can imagine.

Yeah, I mean I spent four years near [city] and I enjoyed that to a certain extent. But I was glad to leave at the time.

Oh actually thinking about that time that you spent down the Channel I think you mentioned –

I lived at [suburb].

Yeah, OK. So do you think your physical activity sort of patterns and behaviours have changed since then or not really?

Ah well that's interesting because when I, I moved to [suburb] so that I could do a three year solitary retreat. So as far as physical activity goes, walking was out, I was still doing Hatha yoga, but as far as outdoor activity goes I was limited by walls basically. So apart from chopping firewood and things like that, yeah my activities have changed drastically.

Yeah, so you didn't leave the property basically. Is that what you mean?

Yeah, it was a solitary retreat yeah.

Wow. OK. Um, so I imagine your physical activity would be very different by the sounds of it. OK. Alright. I might switch the recorder off.

AUDIO RECORDING ENDS

Date:	10 October 2011
Duration:	48 minutes, 6 seconds
Interviewer:	[interviewer]
Transcriber:	[transcriber]

OK. So I thought we'd start with talking a bit about the place where you live. Um, and what it's like to live around where you do. So could you tell me about that and maybe your likes and dislikes? Now, where are you actually living, is it in [town] itself?

Yes, I actually live in [town] town itself, really close, fairly close to the beach. Probably within 150 metres of the beach.

Sounds lovely. Um, so do you live on a house and standard block or are you on a few acres there?

It's a house and standard block. It's actually a rented house, rented from the [organisation].

Oh of course, of course.

Fairly decent sized garden, but its close obviously to the beach.

Um, that would be a main plus. Now what's your reasons for living there.

Um, I suppose work really. The house is supplied with the job, but having said that I actually was living down in [city] and decided to come up to this area. So I actually drove up here prior to moving to see what the area had to offer. So the decision, although job wise, was actually partly mine anyway.

Yep. Fair enough. And so um – just getting a sense of your nearest township – you are actually in [town] itself in the centre of town I presume?

Yes.

Yep. OK. And so that's your main centre, the nearest next one would be [town] I presume?

Yes.

OK. Now in research with men living in urban areas, and I was saying that most of the research has been done in urban areas, people often refer to their local neighbourhood. And we are interested in understanding what the term neighbourhood means to men who live in rural areas and indeed if it is relevant. So what does the word neighbourhood mean to you?
Neighbourhood really, I mean [town] is a town that has a fairly defined area. I tend to think that it fits into an ampitheatre sort of area and that would be what I would define as the neighbourhood, ah the town. That is, there are other areas also classed as [town], which tend to be more sort of rural as you go further out from the town.
Yeah, cos [town] itself, you know there is that immediate centre of [town] but the actual sort of [town] goes a few kilometres back up the hill doesn't it?
Yes.
So, if you needed to put a distance –
Ten or five minutes of the CBD I suppose would be the neighbourhood.
Yeah, so if you needed to put a distance of kilometres on that, how big a distance would it be? In terms of neighbourhood?
Probably a kilometre and a half.
Yep. And what would the word community mean to you?
Um, pretty much the same thing I suppose. Community would be people that um, are encompassed in that sort of general area. Ah, but having said that people will travel from outlying areas into [town] um for community events. So they could be classed as [town] community as well.
Yeah. So thinking about that, if we were thinking about a distance around that, what would be the distance of the community then?
Ah, probably about five, between five and ten kilometres even.
Yeah, so a bit further out. OK. Thank you for that. It's interesting talking with people because it seems to come across as quite consistently neighbourhood is a closer thing than community. Community usually encompasses something a bit broader. OK. Now I wanted to talk a bit about physical activity and also what the term physical activity means for you? Understanding it is a broad term. So what does it mean to you?

(Laughter) Getting off me backside and actually doing something. Something like, well something in the open air, walking, cycling, that sort of thing. Or domestic chores which involve some sort of physical input like lawn mowing and gardening, that sort of thing.

Mm. Which can be quite strenuous. Especially on this time of the year. Now, do you consider yourself to be physically active?

Yes. I do. Yes.

And why so?

Why? Um, good question. I actually make a point of actually trying to do something every day that would be classed as physical activity, be it walking the dog or going for a bike ride. Um, not to the point where I'm a sports nut. So I don't go out playing football, cricket and all those other things. But from a health point of view I try and do something that will you know, keep me fit.

Oh that's really good. Yeah, that's really clear, thank you. Um, where does physical activity fit in your life? Is it for instance of no interest, or a priority or a high priority?

Um, I've never really put a, or never really prioritised it or put it on a scale. I'm aware that if the time and the circumstances are available then I should go out and do something, rather than just sitting around. So for instance like today it's a nice sunny day, better go out for a bike ride or walk the dog.

That's really clear too. That's um, it's important. Like a lot of people actually say they don't really think about it, something that it's part of their day to day. I mean they are aware that they want to do it but yeah, in terms of priorities. So in our research when we think about physical activity, we think about any activity that lasts for at least ten minutes. That's the evidence of health benefit effect. And causes your body to work harder than normal. So your heart rate might go up. You might breathe a little heavier. Or um, you might be, you know you feel yourself warming up in temperature. So I'd just like you to think about that type of physical activity over the last couple of weeks. And I'll go over some different parts of your life. Not to divide up your life in segments, but mainly that we don't miss things. So in the past two weeks have you been active at all at work for at least ten minutes at a time?

Actually at work?

Yes.

Yes.

Yep. And can you tell me about what you did?

Um, at work I'm either, it's either in the car, in the office or walking. So I would actually be walking around the town, at work.

Yes, so that would be the nature of your work.

Yes.

Yep. And so thinking about that activity, what would be the intensity of say that walking? Um, would it be steady or moderate or vigorous?

Ah it certainly wouldn't be vigorous, unless I was intent on getting somewhere quickly. Steady I would say, just plodding around, yeah not fast paced walking, fairly steady, yes.

OK. And um over the last couple of weeks how often might you have done that in the course of work?

Oh three or four times.

Yep. And for how long at a time?

Oh, how long it takes me to get round the town. Probably, I don't know, 20 minutes, could be 25 minutes.

Yep. And when? During, what time of the day would you have done that?

Ah, all sorts of, depending on the shift.

Yep. So that could be um, day shift or –

Yes.

Which shifts do you have? Afternoon shift?

Afternoon shifts and then sort of late afternoon shift.

Yeah, that take you through to the evening.

That's it, yeah.

And is that what you usually do or was it one off?

No, that's what I generally try to from a work perspective, try and get out there and walk, walk and talk, that sort of thing.

Yeah. And does it change depending on the season or time of year?

Yes. Yes, certainly in the winter it's not so inviting to go for a walk around the town.

No, especially if you want to catch up with people. And often a lot of other people aren't around when it's wet and raining too. Um, OK so if you'd wanted to is there anything that would have helped you be more active at work?

Ah, just thinking about things like you were talking about gyms, that sort of. Yeah I believe that some of the larger, well I suppose stations, ah would have rooms set aside as gyms. But I understand that they are now providing sort of, not membership, but access or encouraging people to become members of gyms. I mean it's in your own interest to um maintain –

Yeah, keep fit for work, yeah OK. So the workplace might provide you with information or um,

Yes.

Mm. Would it be the offer of a reduced membership or ..

I'm not sure actually. I was trying to look at it last night. But I think various different organisations, various different gyms have different packages for like corporate memberships, that sort of thing.

You'd need to be part of a bigger centre would you, to really, like being in [town] is a smaller area from that point of view.

I have to say that I'm not really, as I say, a sports nut. I know that I would actually, not be motivated enough to want to sort of really go off to a gym every, you know, times every week. I'd much rather do it myself.

Thank you for being honest about that. And what's interesting is, you know like physical activity doesn't have to be about gyms. And quite a few people I've talked with, that isn't their thing either. And so this is what is interesting about this study, is it is actually starting to give much more of a reality to what people do do about physical activity. Like you know it's something that they're interested in but it doesn't mean they go to a gym to do it. So thank you for being, sharing that one, it's really good to hear. Very useful for the study. So, in the past two weeks have you walked or cycled for at least ten minutes at a time to get to or from places?

Yes.

OK. Can you tell me about what you did?

Um I generally, if I'm on some sort of night shift, or afternoon shift, and I'm able to, ah when the weather is OK, I will generally take the dog for a walk prior to that shift.

Or if it is the other way round, now with daylight saving I will do the day shift and then I'll come home and take the dog for a walk. So that probably involves 30 minutes or at least 30 minutes of exercise down the beach running around with the dog etc. If I'm on a day off, um quite often I'll do both. I'll take the dog for a walk and then go out for a bike ride. So a bike ride could be from here to [town] or [town] or [city] or somewhere like that. So fairly intense physical activity for you know, a couple of hours. But that's mainly on days off. Because I don't want to wear myself out for the day.

Yeah. Absolutely. And that is what this question is getting to. So thinking about the intensity of those two main activities you are doing, the cycling and the dog walking, um would you call it steady, moderate or vigorous?

Um, the dog walking is steady to moderate. Begins steady, we get down the beach and then he runs around so it's fairly moderate. Um the cycling is fairly vigorous.

And OK, thinking about the last couple of weeks, how often would you have done those activities?

Um, probably been out on the bike three or four times, and walk the dog pretty much every day. So, missing a couple of days when I would have been sleeping from night shift. Probably over the last 14 days, probably ten.

That's one thing about dogs, having got them myself, they certainly encourage you to go out and enjoy a walk don't they?

Yes. Absolutely.

And how long would you have done those activities for? The cycling and the dog walking? At a time?

Dog walking would be anywhere between half an hour to an hour. That's down to the beach, along to the end of [town] and back, that's the long walk. Ah, or I do a shorter walk down the other way which is probably half an hour. And then the cycling um, over to [city] and back. Or over to [city] and back, probably a couple of hours. Two or three hours each time.

Right. And um, when would you have done those activities?

Ah, the dog walking generally during the day, but with a couple of exceptions sort of late afternoon. Um, cycling generally sort of mid morning around lunch time.

Yeah and that sort of fits in with your shifts and yep. Um now I just need to check about where you do this cycling and walking. Now you've told me about the walking with the dog down the beach, and you've told me about the bike riding in to [city] and [town] and [town]. Are there any other destinations that you ride to?

Not yet. I'm gradually branching out.

Yeah, that's a long ride.

Well I'm getting old. Getting a second wind you see. I did a lot of cycling when I was young. Not an elite sort of racing types, but I do have all the gear. So um, so often I've just gone out to [suburb] and –

OK. You've got a beautiful area to cycle. That whole coastline is just absolutely gorgeous.

Not too bad. The bit between [town] and [town] along the [x] road is a bit scary sometimes.

Yeah, a few people have mentioned that. And we will obviously go onto that a bit more, but it's a bit narrow. There's not much room for bikes on there. OK. So having a think about where you did do these activities, why did you do them there? What attracted you to those particular places?

Um, well apart from the obvious, I suppose because they're nearby. But the ride between [town] and [town] to [town] is a nice ride, lots of um –

Do you go on the cycle track from [town] to [town]?

Yes, yes I do. Yeah and that's great. That's a nice ride. As I say the bit between [town] and [town] can be a bit um scary around the bends. Basically I suppose because it's there. You either go east or west.

Yes, one person suggested maybe um, I don't know, a few people have said that it would be nice to improve it a bit so it was a bit more cycle friendly. Because it is very narrow and there's not much room either side of that section from [town] to [town].

Yes. If not that, actually sort of educate some of the drivers about cyclists.

Yes, one person suggested maybe dropping the speed limit a bit might help too. Just maybe 10ks, so people aren't trying to go 80, or I doubt that you'd get 80 along there anymore. But certainly people seem to be enjoyable that bit of bike track from [town] to [town].

Absolutely. Yes. And I always see other cyclists, people walking, prams and kids on little bikes. It's a really nice, user friendly track.

And it certainly seems like it's watch this space for the next bit of bike tracks and walk tracks along there. Sounds like it's ah, yeah. So now when you did those activities were you alone or with others?

No, on my own.

OK. And Is that what you usually do or was it one off?

Yes, no that's the usual.

Yep. And does it change depending on the season or time of year?

Ah, yes. I'm not, ah when I was younger I used to cycle a lot in all sorts of weathers, but the joy factor sort of goes down if you're cycling in the rain. Although I suppose it's about positive mental attitude. I prefer to be in the sun.

That's a fair comment I think. And if you wanted to is there anything that would have helped you use, you know, walking or cycling more often, to and from places?

Um oh well obviously the better cycle path. But no, not really, it's generally dictated to by shifts and days off, that sort of thing. I mentally say to myself, I've got four days off, I must go out for a couple of rides. It's very easy to sit round the house and do not too much. So you have to make a point of actually doing stuff. I mean I talk about cycling. And I'm walking obviously. There's other, like lawn mowing and stuff like that which is physical, but that's only probably every two or three weeks.

I'm just about to ask you about that one. In the past two weeks have you been active around the house or yard, inside or outside the home for at least ten minutes at a time?

Yes.

And can you tell me about what you did?

Yep. Mowing the lawn, doing the whipper snipper and generally tidying up the yard.

And thinking about the intensity of that, would that be steady, moderate or vigorous activity?

Um, fairly vigorous actually. It's a fairly decent sized lawn, so yes, fairly vigorous.

And the duration? How long would you have worked on outside for at a time?

Oh probably a couple of hours.

Yep. And how often in the last couple of weeks?

Um, once, yeah, once. It takes a couple of hours to do all the edges, mow the lawn, get rid of the clippings and generally have a tidy up. So that's a couple of hours every couple of weeks.

Yeah, it's a lot of work. And especially when it's spring and all this grass just appears from nowhere. Um, so and when would you have done those activities?

Um, during the day probably, the same thing, mid morning till around lunch time.

Yes, yes. So that's more dictated by the time of the day, rather than during the week or on weekends.

Yep.

And that's because of your work. Yep. Um, and would you have done those activities by yourself or with others?

Yes, no by myself.

And is this what you would usually do, or is it one off?

Yes, yes that's what I'd normally do.

And does it change depending on the season or time of year?

Yeah, the grass grows quicker in the spring and the summer, or certainly the spring, so um yeah I mean there was times in the um, during winter when I thought right I must mow the lawn this weekend, or I got a couple of days off and of course it's raining and it doesn't happen. So yes, it is dictated to by the seasons.

Now, have you been physically active for at least ten minutes during your leisure or spare time? So that's anything else we might have missed.

No.

Great. OK. Um, now the last section is about the physical environment. So we talked about where live and your physical activity, now I want to talk a bit about the physical environments where you live, work and play. And when we talk about physical environment, we mean all the physical things that surround you, the roads, the trees, houses, shops, traffic. And some of these things are things that people living in urban areas have said might be important for their physical activity. So we're interested in finding out for people who are living rurally if they're relevant to you. So just first up, can you tell me a bit about how easy or difficult it is to be physically active in your area?

Um, fairly easy really. Because [town] is, as I said earlier, a bit of an amphitheatre, but the beach area is fairly flat. There's three sort of beaches and if you head east along the road, apart from a few little hills between [town] and [town], it's fairly flat. And the same the other way going towards [city]. So generally I would say it's fairly easy if you are a runner or a cyclist, like swimming, walking on the beach, that sort of thing, it's fairly easy to be active. If you had the intention, there's a sports centre, a footy ground and various other amenities here. But if you were just relying on just yourself, yeah there's plenty to do. As I say, being a cyclist, my main thing would be the safety of cyclists on the road and improve bike tracks.

Mm. And we'll certainly go into a bit more about safety as well. Um, so there's a few areas that I will be asking you about. One of them will be safety. Now the first one is availability and accessibility, which is what you've been talking to me about just now. Um, so I'll just ask you if there's anything else you want to add, um so the question is about if I were to ask you about places to be active in your area what sort of things would you tell me about. You've already just told me that. And we've also been talking about the cycle paths and tracks and beach and as well as the recreation facilities. So is there any other things that you want to add in there?

Oh it just comes to mind although we've got the sea here, and I've been in for a swim a couple of times last year, but the water is still cold. We lack a decent aquatic centre up this end of the state. [city] and [city] have got them and I know that there's swimming pools that are available to the public and various places that are generally linked with schools, that sort of thing. But a stand-alone aquatic centre would be lovely and I think would promote sort of swimming during the winter, that sort of thing.

Thank you for that. Actually a couple of other people, one other person started, talked about that who lives in [town], and was saying that it would be good to have like an aquatic centre that was accessible for everyone. And I think that he was saying the pool there you actually have to be member of a club or something to get access and it's not all year round.

If you decided in the winter that you just wanted to go for a swim, you could pay a couple of dollars and go for a swim.

Yep. So thank you for that, that's great. Now, access. Are places convenient? Are they easy to access? For instance, with their cost and opening hours?

Not that I use them, but I understand that there's a couple of gyms that have opened up in [city] and [city] that are open 24 hours a day, 7 days a week, every day of the year. Um, but for walking and cycling, um all day access, no problems at all. Plenty of availability, yeah.

OK. And um, so does having places to be active influence whether you are active or not?

Ah, for me personally, not really. Because I don't rely on facilities or anything like that for my activity.

No. Though this is a broader question, you know like you've been talking about cycle paths and walking tracks, so it is a broader question. It doesn't have to relate to those facilities that people have to pay to get into. Um, it's just a more general question about um, if you know, like having that place to be active. For instance, you know the cycle path that you talked about, um does that actually help um influence being active?

Oh absolutely. Yes. Previously I'd just been cycling through Ulverstone and back, and probably would be sort of well fairly puffed out, not so much now because I'm getting fitter, but then thinking right well I'll try the cycle path. I did it a couple of times and thinking this is just fantastic, why don't they have this all along the coast? And it certainly has had an impact on –

Yeah. And everyone I've talked to. And we not only have um, this part of the study is talking with men, but earlier in the year we talked with women as well, and everybody has been saying, yes, please extend the cycleway. The walkways and the cycle paths that we've got are great, but everyone is very keenly watching the next step of the plan coming to fruition. So it sounds very exciting up there, from that point of view. Um, now if you wanted to be more active are there things or places that would help if they were more available and more accessible? And you have just told me about extending the pathways. Are there any other things that might help?

Um, in terms of facilities?

Yeah. Oh that's right, the aquatic centre as well.

When I was younger and lived in [country], ah it was a regular thing to go to a local swimming pool a couple of times a week. And I would happily do that here if the facility was here. Although as I said, we've got three lovely beaches here, it's just, you've got to be a hard core swimmer to..

Yeah. And so a lot of the year you can't actually go in the sea and swim. So yep. That's good. Now destinations is another one. If I were to ask you about places that you could walk or cycle to from your home as opposed to getting in a car and driving, what sorts of things would you tell me about?

Um, just trying to understand the question. Destinations, as in if I wanted to go to [city].

Yeah, things like ah, yes, in terms of um [city], could be shops, schools, ah workplaces, recreation facilities, could be friends. Some people talk about you know, going for coffees, and things like that. So anything that is a destination.

Yeah, ah, no problem at all cycling to [city] or [town]. Or even the goal is obviously to get to [city], because I know that there are coffee shops and other shops and things to go to. So that's not a huge issue, like a bad issue, they are all accessible.

Yeah. So does having destinations actually influence whether you are active or not? Does it help?

Yes. It's almost like it's, I suppose it's like a psychological thing that almost like saying to somebody, well you are running a marathon, you are running so many kilometres. You are cycling and at the end of that right, you can sit down and have a coffee. You've got a finish point. That sort of thing. It's clearly defined.

Yep. And if you wanted to be more active would it help if there were more destinations within easy walking or riding distance?

Yeah, yeah I think so.

OK. Another area is neighbourhood function or design. So if I were to ask you about the built or man-made features of your area, what sorts of things would you tell me about? They are the sorts of things like footpaths and lighting and whether the streets connect. You know so they are walkable and cycleable.

Certainly um the road infrastructure, that sort of thing is good. Apart from, as I say, the bit along the coast.

Yeah, that narrow bit.

But that is dictated by the geography anyway. There isn't much you could do in the way of widening, expanding the roadways that sort of thing. But generally I think the layout is good. Um, don't really have sort of too many issues with that.

Mm. Do you, do the built features in your area influence whether you are physically active or not?

Um, yeah I suppose they do actually. I mean certainly the ability to walk along, certainly along decent footpaths, down to the beach using decent steps and access points, that sort of thing. Cycling, having certainly between here and [city] the road is wide enough that the edge like the hard shoulder is wide enough to cycle safely along. Um, yeah I've no issues in relation to –

Yeah, no someone was saying that to get to [city] you actually have to go back on the main road at one point. The last point. So is that safe enough, that you've got enough room?

No, there's a couple of bits around between, I'm just trying to think, between [suburb] and probably [suburb] they are a little bit scary because of like rivers or bridges going over the geographical features, the bridge themselves actually narrow the roads, so you are then forced back into the traffic. That's the only concern there. And in fact, I noticed the same thing cycling to [38:06] a couple of weeks, everything else was OK, the cycle path was good and I got to the bridge over the [x] River at [town], then all of a sudden there is basically no um bit on the side of the road. You're on the road. And I nearly got cleaned up by a bus going across the bridge. And I thought that's not right. There should be a rider bit there.

Mm. Thank you for that. That's a really useful comment. Um, now if you wanted to be – OK the other side of it is the natural environment. And the aesthetics. If I were to ask you about the aesthetic qualities or attractiveness of your area, what sorts of things would you tell me about?

I just think the area is beautiful. The whole [region] is lovely. Um, then if you go inland as little as half a kilometre or a kilometre the countryside changes, green, fertile land. Very picturesque.

It's a beautiful area. It has all sorts of diversity in that way.

I often comment when we see campervans and people coming over on holidays that people have paid thousands of dollars to be on holiday here, when we actually live here.

That's a very good point, yeah.

Tasmania generally, not just up this area.

And the interesting one with this study, although it's not a focussed point, but a lot of people I've talked to have actually chosen to come and live in [town] and [town] areas. They may have come from elsewhere actually, and they've actually chosen to come because of you know, obviously the beauty of the area and what it has to offer. Yeah. And um, do you think your local environment is aesthetically pleasing, near your home?

Yes, absolutely.

And do the aesthetics of your area influence whether you are physically active or not?

I think so. Certainly when I take the dog for a walk, ah walking along the footpaths with lovely trees and the sea views and all that sort of thing. It all sort of fits together in that sort of package. Yes.

Yeah, it makes it a very enjoyable experience.

Yes.

And if you wanted to be more active, are there features of your area that might help if they were more aesthetically pleasing or attractive? And that's the natural part of it.

Ah, I don't know whether you could make it more.

I was going to say, it's pretty beautiful as it is. OK, just one last area which is the safety area. If I were to ask you about safety in your area, what sorts of things would you tell me about? And that's about traffic and etc. So one thing was that narrow road between [town] and [town], in terms of safety for cycling, what other things?

That's probably I think generally safety is quite good. Street light could be better. I certainly notice that me being from [country], I mean I've been over here for twenty years but I notice that the lights and the street lighting in [country] is better than it is generally in [country]. Um, having said that certainly in the town centres, um in [town] and [town], the lighting is great. Um, very very good. Um, safety wise, as I say, the only real issue between [town] and [town] is that coast road. Having said

that most of the traffic travels on the [x] Highway. The traffic that does travel between [town] and [town] certainly a heightened awareness of that there may be cyclists or slow moving vehicles by way of signs or other ..

Yes, that's a really good point. Yeah, for warning people too. As you say, because it's actually not the major route, it could be some fairly simple things could be thought of to make it ah more you know understandable that it's a coastal route that is enjoyed by cyclists and indeed everyone. I've seen people trying to walk along there too. So does road safety influence whether you are physically active or not?

I suppose it does actually. I certainly notice a difference um cycling during the day and I have cycled over to [city] later on in the afternoon and I can say that that is a bit scarier than doing it during the day because of the traffic density. So going back to the cycle path that is another reason why that is such a good facility.

And does personal safety influence whether you are physically active or not?

I suppose um just trying to think how to answer that. Obviously it's my job to ensure that everybody else is safe.

Yeah, I know, I know. I mean generally people have commented that [town] and [town] is a really safe area. It's just if people are living on the land and then walking on their properties, they just point out that they have got to watch out for snakes at certain times of the year. So that sort of tends to be how people have answered that one.

I don't think generally there is any safety issues here. You can't be complacent but we are also very lucky to be in this part of the world, where it isn't too much of an issue. Obviously common sense plays a fair part in it, when you go outside and walking. You put some light clothes on, that sort of thing. But from a, compared to like walking around a major city, ah late at night, I don't think there would be any issues walking around [town] or [town].

No, that's generally what people have commented. It might be just that you know that further out of the area where the lighting isn't quite as good might be one of the considerations, but it's not about um, people feel overall safe in the community.

Now, um that's actually the end. But just um before we finish up, is there anything else that you'd like to tell me about where you live, your environment or your physical activity that we haven't covered today?

I don't think so. I'm very well aware that the, and I'm not sure how it works with some of the other councils, I'm aware that certainly the previous mayor, sadly he has passed away, but um certainly the input from the council um has been really good as far as physical activity. I know they got this program going up here called [name] In which supports cycling, walking and activities, that sort of thing.

Oh OK. And what's it called again? The program?

[name].

[name]. Oh, yeah, yeah.

It's part of the [x] Council's physical activity sort of awareness thing, and there's a link on their website with a few things that they organise. They are certainly very proactive as far as well obviously the cycle track shows that and the new sports centre. That's really, that's a part of um, I suppose the bonus of living up this way that the local council actually take an interest in.

Yes. In fact they've been very supportive of this study. The mayor herself um actually organised it so we could, I actually came up to [town] for one trip a couple of weeks ago and she actually organised that I could um, you know people who wanted to participate in the study, I could see them there at Council, which was really good because it's a nice central location for people. So they're really interested in this study and what comes out of it too, so it's good. It's very good. Mm. OK.

AUDIO RECORDING ENDS

Interviewer: So, I thought we might start a bit by talking about the place where you live.

Respondent: Yeah.

Interviewer: OK, now you were just saying you lived at the top of [x] Road in [town].

Respondent: Yeah.

Interviewer: And can you tell me a bit about where you live – is it a house with a block on, or...

Respondent: Yeah, we've got 30 acre farm, quite steep, because we're on the top of the hill.

Interviewer: Yeah, and your likes and dislikes of living around the place?

Respondent: Oh, I love it. Yeah, but as I said as the arthritis and everything has progressed, it's a bit hard to get around. I mainly tend to get a little bit fitter when it's calving time – you tend to walk around the paddocks a little bit checking on the cows then.

Interviewer: So that's spring is it, when it gets a bit warmer?

Respondent: Yeah. Yeah. But other than that I don't walk around the property much anymore. I like doing my garden though, and because... yeah.

Interviewer: Hmm, so get to talk a bit more about that as we go along. Now, the length of time you've been living where you have?

Respondent: 13 years. Yes.

Interviewer: Uh-huh. So you're quite settled?

Respondent: Yes.

Interviewer: And your reason for living here?

Respondent: Well we lived right in [town], before in the town, but the opportunity came to get out further, because...

Interviewer: Yeah, how many acres have you got?

Respondent: We've got 30 acres up there.

Interviewer: Yeah, that's a nice size.

Respondent: But we were born and raised in the country. I'll never live anywhere else. I couldn't cope with town life.

Interviewer: Yeah?

Respondent: Yeah.

Interviewer: So what got you moving out of [town] onto land? Just to get further out?

Respondent: Because I came from a farm originally and just wanted space.

Interviewer: Yeah.

Respondent: Yeah, I don't like feeling closed in and having too many houses too close.

Interviewer: Fair enough. And OK, so how far are you out of [town] now?

Respondent: It's just under five kilometres.

Interviewer: Right. OK. Now, I was saying that most of the research has been done in urban areas and in this research with women people often refer to their local neighbourhood, and I'm interested in understanding what the term neighbourhood means for you living in a rural area, and indeed if it's relevant. So what does the word neighbourhood mean to you?

Respondent: Well, the outlying area. I mean our closest neighbour is probably a kilometre away. I mean so it's nice to know that you've got neighbours when you need them, but they're not in your face too much. And the neighbourhood – I mean so we come down and do our shopping, it's nice to catch up on friends that are around.

Interviewer: Yeah, so you would see the neighbourhood as your immediate sort of neighbours who are a kilometre or more away, and then into [town], about five k?

Respondent: Yeah. Yeah. And I mean to say we still do a carpool with friends with our kids, running backwards and forwards to Scouts, and take turns, and that kind of thing.

Interviewer: Hmm, hmm, yeah. Because [woman] said you... you know, she was saying that your connections were with the parents, and friends, and children, and so she was saying she's still got those connections, which is what you're saying too. It's great.

Respondent: Yes. Yes.

Interviewer: And what about the word community – what does that mean to you?

Respondent: Pretty much the same. Yeah.

Interviewer: OK. Yep. So it's the same sort of distance, it's the sort of people that you've got like minded interests with?

Respondent: Yeah. Yeah.

Interviewer: Yep, into [town]. Yep. OK. Now I just want to talk a bit more about physical activity now. And as I was saying it's a very broad thing,

physical activity, and it's really up to the indiv... what it means to the individual, and it even changes in different stages of our life.

Respondent: It certainly does.

Interviewer: Like what you were saying before [laughs], yeah.

Respondent: It certainly does, yeah.

Interviewer: So what does the term physical activity mean for you?

Respondent: A bit frustrating at the moment, especially when I was such a physical active person, and never a lazy person, but since I've had my knees done, and because within a couple of years I'll have to have my hips done, and that's slowed me down heaps.

Interviewer: So that's within the last – when did you have the operation you were saying?

Respondent: Since November I've had my knees... I had my knees done, but they've been really bad for quite a few years. But all of a sudden I've kind of felt lazy [laughs], because I haven't been able to do as much. You either don't do enough, or next thing you've overdone it, and then you can't move for a few days.

Interviewer: So you're tight... start to err on trying not to get into that zone.

Respondent: Yeah. And I'm the type of per... once I start doing something, I've got to push myself to get it finished, and then you suffer for it.

Interviewer: Uh-huh.

Respondent: But I use to do a lot more than I did, like walk more, and do more exercise, play a bit of sport – but I can't do that now. I've gone to the Sports Centre, but I find a lot of the machines are a bit hard. But I was doing swimming, but the cold drives a lot of people out – the water is just so cold down there. It's supposed to be heated, but you still freeze, it's still cold. And a lot of people say nuh, they don't do it this time of the year, they only do it in summer when it's hot, so yeah.

Interviewer: When it's warm. So does that affect you in terms of... is swimming good for you?

Respondent: I felt a lot better when I was doing it, but it got to the stage where it was just too cold.

Interviewer: Cold, yeah.

Respondent: Yeah, and you spend the rest of the day trying to warm up. So I've got an exercise bike at home; I'm about to change that because my sister-in-law's got one of those low ones, because I had trouble getting on my old high one now because of the hips. But she's actually ordering me one on the internet this week – one of them low ones – so hopefully I can do that while I watch telly. I'll just... I made a little sling to fit on the top of my... to read my books while I'm doing it, or whatever.

Interviewer: Yeah, so you're trying to work your way through what you can do at what stage of...

Respondent: Yeah.

Interviewer: And I presume with the operations you've had around... it's going to take a wee while, isn't it?

Respondent: To get going again, because there still is a little bit of stiffness in one and it still swells by the end of the day. That'll take up to 12 months or whatever. But the wintertime always is harder. At least in the summer months you're out mowing the lawn, or you're in the garden, or whatever. Yeah, but wintertime is always hard to find something to do.

Interviewer: Yes. Yes, yes.

Respondent: And something different to do – you want to be able to go out for a walk or whatever. Somewhere safe to go for a walk, where you're not going to get chased with dogs – that's what I find in the town area, walking around the town, you're always frightened as dogs come out and barking at you, and yeah, so therefore I just... sometimes I just go round and round and round the oval, which is a little bit boring after a while, seeing the same thing.

Interviewer: Hmm, we'll talk a bit more about safety and also the physical environment, and what's accessible and stuff like that. So you've described what activity you can do at the present moment at the point of your recovery, where you're up to, and saying it was just... it is different than it was before. Do you consider yourself physically active at this point?

Respondent: Not at the moment.

Interviewer: Because of that?

Respondent: Yeah. Yeah.

Interviewer: Yep, yep. And...

Respondent: I'd like to be more active, but yeah.

Interviewer: OK. And so that's what I was going to ask you – where does physical activity fit into your life? Is it a priority, or not of interest?

Respondent: Well since I've had bad joints, and I haven't been able to move about, so I've put weight on, and I don't feel fit for it, so I'd like to lose quite a few kilos to be able... it's a vicious circle.

Interviewer: Yeah. Yeah, yeah.

Respondent: Yeah, so I'd like to be able to lose a bit of weight so I can be more physically active.

Interviewer: Yeah. So it is a priority for you?

Respondent: Yeah.

Interviewer: Yeah. OK. So we're just going to look a bit more at physical activity, and in our research when we think about physical activity we think about it as any activity that lasts for at least ten minutes, because that's where the evidence of health benefit is, and causes your body to work harder than normal. OK? And so your heart rate might go up, you might breathe a little heavier, and you might get warmer.

So thinking about that type of physical activity over the last two weeks, now you were saying that you were doing some work here – we'll look at different parts of your life, not to divide up your life, but just make sure we cover everything. So with work in the last two weeks, can... have you been active for at least ten minutes at work?

Respondent: I'm only on call.

Interviewer: Yeah, so would you have done that in the last couple of weeks?

Respondent: So, not really. There was...

Interviewer: Well OK. Well pick a time when you did go in.

Respondent: When I went into work I... yeah, you're flat out at work. Yeah.

Interviewer: Yeah. Yep, yep.

Respondent: Constantly.

Interviewer: And can you tell me about what you did?

Respondent: You're preparing the Residents meals, and then you're cleaning up as you go, and you're just...

Interviewer: Uh-huh, so you're on your feet the whole time?

Respondent: You're on your feet the whole time.

Interviewer: OK. Right. Now, having a look at that, I just want to have a look at what the intensity of that exercise would be – would it be steady, or moderate, or vigorous do you think?

Respondent: Oh, you'd say it's a constant flow. You've got to keep moving, so...

Interviewer: So it'd be almost like steady to moderate?

Respondent: Yeah. Yeah.

Interviewer: Yep. And the duration – how long would you have done that for?

Respondent: First day, eight hours. You stop for a half hour lunch, and then the tea shift is three hours, but you're there about three and a half by the time you finish cleaning and that.

Interviewer: Yeah. So that's... would you say it was eight hours all up?

Respondent: All up you'd say one day it was say seven and a half – you stop for a half hour for your meal – and then, but the tea shift there's no breaks.

Interviewer: Oh, OK.

Respondent: You're just going constantly for that three, three and a half hours.

Interviewer: OK. OK. And how often would you on average do that?

Respondent: Oh, you'd probably get call... I'm only on call relieving other people, so you'd probably only get – one month you'd probably only get half a dozen days. And then like in September one of the ladies is going on long service, she does a tea shift, so probably fill in for her for the month. And that'll be probably your... it'd work out your 15 hours a week.

Interviewer: So you'd work what – five days filling her...

Respondent: Yeah. Or there might be another lady, one of the other casuals take a turn as well. So it's hard to tell.

Interviewer: Yep. No, that's fine. And when during... is that during the day that you would usually work?

Respondent: Yeah.

Interviewer: Yep. And is this what you would usually do, or is it one off? So is something you'd usually do at work, the activity is fairly constant?

Respondent: Yeah. Yeah.

Interviewer: Yep, yep. And does it change depending on the season or time of the year?

Respondent: No.

Interviewer: No? It only changes if people are on leave, or you need to work extra hours?

Respondent: Yeah.

Interviewer: OK. And if you'd wanted to, is there anything that would have helped you be more active at work?

Respondent: No.

Interviewer: No, because you [laughs]... that's why you're continually active.

Respondent: It's a set routine, and that's it. Yeah.

Interviewer: Yeah. OK. Now, in the past two weeks have you walked or cycled – it's probably walked, isn't it?

Respondent: Uh-huh.

Interviewer: For at least ten minutes at a time to get to or from places?

Respondent: Not walked in this weather, but...

Interviewer: I know. It's been really cold, hasn't it?

Respondent: Yeah. No, the only thing I've done is set myself 15, 20 minutes on the exercise bike.

Interviewer: Uh-huh. OK. Yep, yep. And so really sort of at this time of the year where it's very frosty and cold.

Respondent: Uh-huh. Set outside... step outside the door and you're back in again. [Laughs].

Interviewer: Uh-huh. And so does it change depending on the season or time of year?

Respondent: Yeah.

Interviewer: Yeah. Yeah.

Respondent: Yeah, when the weather's much nicer I'd get out and walk more often, and you just seem to enjoy life more too.

Interviewer: Yeah. Yeah, so how often would you get out and walk?

Respondent: I try and do probably little... oh, at least the school mornings, drop my son off on the school bus, I do a... probably a quick lap around the block, do the [x] walk around here.

Interviewer: Yeah, yeah. Yeah.

Respondent: And then probably in the afternoon before I pick him again...

Interviewer: Yeah, do another.

Respondent: ...do a couple of laps around the oval, something a bit different. But this time of the year I don't.

Interviewer: Uh-huh. So that would take you what sort of time?

Respondent: Well I got up... before it got too cold I got up to four laps around the oval, which took me about 20 minutes, half hour.

Interviewer: Uh-huh. And the [x] walk?

Respondent: Oh, that's only about ten, 15 more minutes. Yeah.

Interviewer: Yeah. And when it's sort of warmer, how often might you do that in a week?

Respondent: I try and do it one a morning and evening as I drop him off – you probably miss a few days, it just depends what you've got to do. Or if I've mowed the lawn...

Interviewer: So you might do it a couple of times a week, you might not.

Respondent: Yeah. Yeah.

Interviewer: And what would be the intensity of that exercise?

Respondent: Oh, at this stage it's just a gentle walk.

Interviewer: It's pretty steady?

Respondent: Yeah.

Interviewer: Yeah, steady, gentle walk.

Respondent: Yeah.

Interviewer: Yeah, yeah. Getting those knees gradually back. [Laughs].

Respondent: Yes.

Interviewer: Yeah, it's a long time. It must be really frustrating.

Respondent: Yeah.

Interviewer: Yeah. And if you'd wanted to, is there anything that would have helped you walk to and from places? The weather. [Laughs].

Respondent: Yeah, that's about it.

Interviewer: Yeah. And obviously feeling a bit more yourself.

Respondent: Yeah.

Interviewer: Getting your strength back. OK, in the past two weeks have you been active around the house or yard – so at home, either inside or outside?

Respondent: Yeah, kind of started spring cleaning.

Interviewer: Uh-oh. [Laughs].

Respondent: I was up scrubbing ceilings yesterday, and walls.

Interviewer: Wow. Wow.

Respondent: Just mow the lawn, and while the weather's like this I've been out kind of catching up on the garden a bit, and just started a little bit of spring cleaning.

Interviewer: So thinking about those activities, like its spring cleaning and the usual housework, of course, and the gardening, all three you've mentioned, what sort of intensity would they be?

Respondent: Scrubbing the walls and ceiling, yesterday I was mainly getting up and down on the ladder.

Interviewer: Yeah. Would it be steady or moderate?

Respondent: Yeah, steady. Steady, yeah. I take my time.

Interviewer: OK. Yep. So all of tho...

Respondent: Still cautious but... yeah.

Interviewer: Yep, so all of them would be steady. Well you have to be steady being in recovery.

Respondent: Yeah. I have to be. Yeah.

Interviewer: And how long might you have done those activities for? Just sort of broadly at a guess.

Respondent: Yeah, I'll say between ten and two-thirty, so about four hours spring cleaning yesterday. And then the other days it takes me quite a while to do the lawn – it takes me an hour to do the side and the back, and then I'll come in and have a cuppa, and then I'll go back out and do the front.

Interviewer: So it's a few hours?

Respondent: It's a couple of hours mowing.

Interviewer: Yep, yep. And the housework?

Respondent: Oh, it takes me – the basics, like washing, and vacuuming, and washing the floors – normally it takes me two and a half hours of a morning.

Interviewer: Yeah, so that would be... and how often would you do these activities in the last couple of weeks?

Respondent: Vacuum most days. I've got three messy boys – that's including the husband – so I vacuum every day.

Interviewer: Yep. So housework's every day, so...

Respondent: Yeah, and wash every day.

Interviewer: ... yep, so it's about one and a half hours every day.

Respondent: Yeah.

Interviewer: And then your spring cleaning, how often might you do that?

Respondent: I do that a couple of times a year to go through the whole lot.

Interviewer: Yeah. Yeah. So in the last couple of weeks you've just started that.

Respondent: Yeah, I just started yesterday actually.

Interviewer: And the lawn – how often might you do that? It depends on the weather too, doesn't it?

Respondent: Yeah. Not as much this time of the year, but in the springtime mow the lawn a couple of times a week. But we've got fruit trees; I'll get up there and prune them as well. They need pruning.

Interviewer: Oh, OK. Yeah, I was going to say, it's that sort of time.

Respondent: Yeah, I've got 16 fruit trees, so I've got to start that. Grow our own veggies and everything, so I'm in the garden...

Interviewer: Because things are already starting to reshoot.

Respondent: Yeah.

Interviewer: It's incredible.

Respondent: Yeah. Yeah, and I've just planted a few things in the hothouse, so... yeah.

Interviewer: Uh-huh. You've got all that to do too. Yep. OK. So when would you do these activities? Would they be during the day, during the week?

Respondent: Yeah, during the day. Yeah, week days.

Interviewer: Yep, OK. And who would you do them with?

Respondent: I do them myself.

Interviewer: Yeah. And you've just sort of explained that this is... some of the activities are what you usually do, some of them they're dependent on the weather.

Respondent: Yeah, and the seasons and that. Yeah.

Interviewer: OK. Yeah. And if you wanted to, is there anything that would have helped you be more active around the house, inside or outside?

Respondent: Not really. No.

Interviewer: No. OK. And have you been physically active for at least ten minutes during your leisure or spare time? So that just picks up anything else that we might not have picked up already.

Respondent: Not... oh...

Interviewer: Well you were talking about your exercise bike before.

Respondent: Yeah, if I feel that I've been sitting around – because I've been re... since I've had my knees done I've read more books than I have in my whole life. I'm thinking no, I'm getting a bit lazy, so I made a little sling to fit on the handlebars of my exercise bike, so I put my book up there, and I'll get on the exercise bike while I'm reading.

Interviewer: So let's have a look at that one. Would you describe that exercise, like thinking about the last two weeks, as steady, moderate, or vigorous – the exercise bike?

Respondent: Oh, I start off steady, and then I'll probably do a minute as quick as I can, til I get... I can feel my heart rate coming up, then I'll ease down for a minute, and then I'll have another bit of a burst. Yeah, so I'd do that for about five, probably six...

Interviewer: So an average start, fairly moderate?

Respondent: Yeah.

Interviewer: And you'd do that for how long?

Respondent: I try and get 20 minutes in.

Interviewer: Yep. And how often would do you that?

Respondent: I don't often get the 20 minutes in. I'd try and do that at least once a day.

Interviewer: OK.

Respondent: Doesn't always happen, but... yeah.

Interviewer: No. OK. Yeah, so that's been important, working out something for you that you can actually do right now after knee replacement.

Respondent: Yeah. About... the exercise bike is about the only thing I...

Interviewer: Yeah, and also the weather too.

Respondent: Yeah.

Interviewer: Yep, yep. OK. And this was during the day at home, and who would you be doing the exercise with – you would be doing it by yourself?

Respondent: Yep.

Interviewer: And this is what you usually do?

Respondent: Yeah.

Interviewer: Yep. And does it change depending on the season or time of the year?

Respondent: Yeah. In the summer and the spring I just spend more time outside in the garden. And daylight saving, I love that, you get outside more often.

Interviewer: OK and you'd have more walks?

Respondent: Yeah.

Interviewer: As you were describing. OK. And if you wanted to, is there anything that would have helped you be more active in your leisure and spare time? Like, we were talking about the swimming pool, having it a bit heated.

Respondent: I'd love to spend more time around there, because I had a routine of water aerobics, and because when I was... had to do from the physio.

Interviewer: Yeah, oh, they're wonderful. Yes.

Respondent: It was brilliant, and I felt healthy for it, and I was starting to feel real good, but the water just got too cold.

Interviewer: Yeah, you have to have warm water for those sorts of things.

Respondent: Yeah. And just couldn't move, and it just took so long to warm up afterwards, and you spend the rest of the day aching or trying to warm up.

Interviewer: Yeah, it's not suitable for it.

Respondent: No, it's just not.

Interviewer: No.

Respondent: And yet I loved doing it, but... yeah.

Interviewer: OK. Yep. No, that's a really important point. OK, let's look at physical environments now – the environment that you work, play, and live in. And when I talk about physical environment I mean all the physical things that surround you – so it can be the trees, the paddocks, the roads, the houses, lights, and footpaths.

And some of these things are things that people living in urban areas have said might be important for their physical activities, so we're interested in seeing whether that's... what it means in rural areas basically. Can you tell me a bit about how easy or difficult it is to be physically active in your area?

Respondent: Other than my garden and the exercise bike, it is a bit difficult for me now because we live on the top of a hill.

Interviewer: Yeah, it's got a slope, huh?

Respondent: Yeah. On [x] Road, a lot of trucks and things, so I normally get in the car, drive down here to town to find a flatter area, either around the oval, or around the back streets and things. Yeah, normally have to drive somewhere to go for a walk.

Interviewer: So what makes it difficult is the steepness?

Respondent: Steepness.

Interviewer: And you mentioned traffic as well?

Respondent: Yeah, up the [x] Road the traffic on blind corners and log trucks, and just cars.

Interviewer: Yeah. And I presume it's just a bitumen road – there's no sort of footpaths or anything?

Respondent: Yep, there's no footpaths.

Interviewer: It's just gravel on the other side?

Respondent: Yeah.

Interviewer: And if it's narrow in places with bends and corners, it's not safe.

Respondent: Yes, it's just not safe. It definitely isn't safe.

Interviewer: OK. No, that's really interesting. Now, we're going to talk about availability and accessibility of places to be active, and I'll talk about a few features, but this will be... we'll start here. If I were to ask you about places to be active in your area, what sorts of things would you tell me about?

Respondent: Well there's the Sports Centre, but as I said a lot of the machinery at the moment I can't use, and the pool was the only thing I enjoyed, but it just got too cold.

Interviewer: Yeah. And so you'd need to drive there because it's sort of six k over at [town], isn't it?

Respondent: Yes, you've got to drive.

Interviewer: Yep, so you drive in, and so it's about probably ten, 11k here?

Respondent: Oh, five... yeah.

Interviewer: Yeah. Yep, yep. OK. So it's a bit difficult having equipment there that doesn't...

Respondent: And I found too, because I was doing in the sum...

Interviewer: Did they have an Instructor there to use the equipment? Or do you go in by yourself to use it?

Respondent: You go in by yourself.

Interviewer: Yep, yep.

Respondent: But I was doing a water aerobics class of a night there for a while in the summer months.

Interviewer: Yeah, when it was...

Respondent: But what I found, the parking is shocking there – there's not enough parking room – and sometimes you have to park around the side of a night, not enough lights. I have to take the torch out of my car, to use a torch to walk to the front where the lights are. There is a big light at the side, but it's just the lighting wasn't good enough. I didn't feel secure walking when my legs were bad.

Interviewer: No. No, you feel very vulnerable. Yeah.

Respondent: Yeah. So therefore that was another down point on going there.

Interviewer: Yep. So that's a real issue around accessibility and what's available.

Respondent: Yeah, the parking – the parking's a big issue there of a night time.

Interviewer: Hmm, OK. And you... now what other things are available in your local area? You talked a bit before about the oval that you walked on, and also the [x] walk – any other walking tracks and areas like that?

Respondent: No. No.

Interviewer: No? No, that's it, is it? And that's only a little... a very little walk.

Respondent: It's only a very small one, yeah.

Interviewer: Hmm, hmm. And any other parks?

Respondent: No.

Interviewer: No? And sports clubs and things?

Respondent: No, there's not actually.

Interviewer: OK.

Respondent: Only the football and I'm not really into football.

Interviewer: No, people have to travel if they're going to find something.

Respondent: Yes.

Interviewer: Yeah, it's a comment that people have made.

Respondent: Some people play basketball, netball, at the Sports Centre, but that's beyond me.

Interviewer: Hmm, hmm. Yep. I've heard people say they go up to [city] to play.

Respondent: Yeah.

Interviewer: Tennis – tennis, and no tennis courts, is what I've been told. [Laughs].

Respondent: Yeah, there used to be, but the bulrushes kept growing up through where it was.

Interviewer: Not anymore. And so having or not having places to be active, does that influence whether you're active or not?

Respondent: Yes, it does.

Interviewer: OK. And if you... because it's just a lot easier having places that you can... that work for you, yeah.

Respondent: Yeah. Yeah, sometimes I drive to [town], and my mum, she's 82, we actually found a little walk there behind where the [x] boats are.

Interviewer: Oh, yeah?

Respondent: There's [x] Walk – the Council's put like a little walking track right through there, but that's only a short one too.

Interviewer: Yeah, yeah. So I mean that... and that's what people have said, there just isn't long enough tracks.

Respondent: They're not. Unless you want to...

Interviewer: And they don't connect with anything.

Respondent: Yeah, unless you want to walk...

Interviewer: And you can't walk by the river. [Laughs].

Respondent: No. If the river floods, no. You can walk through there if you want to walk right back down through the town and back. I suppose you could. But I only did that one with, as I said, [mother], my 82 year old mum, which she's fitter than me at the moment.

Interviewer: Yeah. But [town] itself doesn't really have any tracks or walkways.

Respondent: [town] itself, no.

Interviewer: Yeah. And so if you wanted to be more active are there things or places that would help if they were available or more accessible?

Respondent: Yeah, definitely.

Interviewer: Yeah. Which is the things we've been talking about.

Respondent: Yes.

Interviewer: The gym equipment, the warm pool to be able to do aqua aerobics, and some flat areas that are sort of a bit longer in terms of being able to walk.

Respondent: Yeah. Yeah.

Interviewer: Yep. OK. And destinations – if I were to ask you about places you could walk to from your home, what sorts of things would you tell me about and indeed would you walk from your home? You've just said to me that you wouldn't it, it's too dangerous.

Respondent: No, it's too dangerous. Yeah.

Interviewer: Yep, OK. OK. So you'd really have to get into the car to come down to [town] for shops, and schools, and things like that.

Respondent: Yeah.

Interviewer: And your workplace. Hmm, OK. And friends, and...

Respondent: Yeah.

Interviewer: Yep, yep. So does not... well you do have destinations, but you can't actually walk from them, so does not having destinations that you can walk to/from, you know into, influence whether you're active or not?

Respondent: Yes, it does. Yeah. And I'd like to get back into a little bit more, because before I got the arthritis bad, when the boys were a bit younger, we used to do a couple of bushwalks to the waterfalls out the [x], or the [x] Falls. They were a bit of a... they're beyond me now. Like, the [x] Falls, you're scrambling up and down hills and that, but I use to love doing that. I'd like to be able to get back into that, and be able to spend more time with the kids doing it. So it kind of restricts me taking them places too, because of...

Interviewer: Yeah, it feeds on, doesn't it? Hmm.

Respondent: Yeah. Yeah.

Interviewer: OK. And if you wanted to be more active would it help if there were more destinations within easy walking distance?

Respondent: Yeah. Yeah.

Interviewer: Safe walking distance too. [Laughs].

Respondent: Yeah.

Interviewer: Now I'm going to... there's a couple more areas, and this one's looking at the neighbourhood design, which is about your built or manmade features of your area. So if I was going to ask you about that, what sorts of things might you tell me about? Now, we've just said there's lack of footpaths, and I presume there's lack of lighting to go with that.

Respondent: Yeah.

Interviewer: And you've also said it's too steep up there, but down in [town] it's fairly flat.

Respondent: Yeah.

Interviewer: Now how walkable is [town], given that in terms of we've talked about its limited how things connect, because connect... when things connect its important too, because you have somewhere to walk. [Laughs].

Respondent: Yeah. I kind of... there's... I do... like to do the [x] walk, which goes actually across down along the creek here [demonstrating], behind the [building], go up over [x] Street and back down into town. You get up over [x] Street – I haven't done that for a while because there's two big [dogs] run out, they weren't in their yards – that frightened the hell out of you.

Interviewer: Yes, you were saying about dogs. Yeah.

Respondent: I use to go right out the end of [x] Street, out on the main road – which is a main road, no footpaths – and around, but there's a couple of big dogs out that road, so that puts you off a little bit.

Interviewer: Absolutely.

Respondent: And because I haven't been down in [town] for a while you don't know where the dogs and that are, and sometimes you're taken by surprise. So that puts you off a bit.

Interviewer: Yeah, so your safety with dogs is sort of a definite factor, isn't it?

Respondent: Yeah. That's what... like the [x] walk, and I'll go... sometimes I go... get... the little park goes down along the river behind the [building] so I'll turn around and come back. It cuts your walk shorter because I'm trying to avoid where I know the dogs are.

Interviewer: The dogs. Hmm, hmm.

Respondent: But I enjoyed the one that... the new one, having to drive all the way to [town], I enjoyed that one because it's different. You get sick of doing the same ones all the time.

Interviewer: Yeah. So where were you saying you drove to?

Respondent: That was behind [x]. You just do down onto [x] Road, and it's straight opposite the little shed where the jet boats are.

Interviewer: So in[town]?

Respondent: Yeah, in [town].

Interviewer: [town]?

Respondent: Yeah. So it's a 20, 25 minute drive.

Interviewer: Oh, OK.

Respondent: Yeah, to get there.

Interviewer: OK.

Respondent: But my mum's in [town], so... yeah.

Interviewer: OK. So that sort of works quite...

Respondent: Yeah, sometimes when the weather's nicer, I'll say well right, let's do a couple around... laps around the [town] oval, or we did that one, which is a bit different. Yeah, because you get tired of going to the same places, too.

Interviewer: Oh, absolutely.

Respondent: It gets boring.

Interviewer: Yeah, exactly. Yeah, so a very important point there. You need some options, yeah.

Respondent: Yeah, you do.

Interviewer: And if you wanted to be more active are there any built features of your neighbour... well, you're area that would help if they were there?

Respondent: If they were there. It would be nice to have a few more walking tracks.

Interviewer: Yes. Yes.

Respondent: Yeah.

Interviewer: Yep, yep. Absolutely. Now the other side of it's your natural environment, so if I was to ask you about the aesthetic qualities, or the attractiveness of your area, what sorts of things would you tell me about?

Respondent: I mean there's a lot of little rivers and creeks down here. If you had access and paths along the river and through little bush areas, it would be lovely.

Interviewer: Yeah. So what stops the access at the present moment?

Respondent: There's none. [Laughs].

Interviewer: There isn't? There is no pathways?

Respondent: There's no pathways or access to those prettier areas.

Interviewer: Are they fenced off, or are they private property?

Respondent: Some are, but...

Interviewer: Some are?

Respondent: Yes, some are.

Interviewer: It's just you can't get down to them?

Respondent: You can't get down to them to see, yeah.

Interviewer: Yeah. Are they in bush or scrub?

Respondent: Little bits and some are not.

Interviewer: Yeah, it's just hard to follow through to them?

Respondent: Yeah.

Interviewer: Yeah. Yep. And that's certainly been a comment that's come out – it'd be nice to have a bit more.

Respondent: Hmm.

Interviewer: Hmm. And OK, and the other side of it, like you obviously live in a beautiful 30 acres, you've got trees around you, beautiful scenery.

Respondent: Yeah.

Interviewer: Now, OK, so we've looked at the nice positive side of your environment. The other side is things like rubbish, or graffiti – is there any... that noticeable in [town]?

Respondent: No.

Interviewer: No. OK. So do you think your local environment is aesthetically pleasing?

Respondent: Yes. I wouldn't be anywhere else.

Interviewer: Absolutely. [Laughs]. OK. And do the aesthetics of your area influence whether you're physically active or not?

Respondent: In the summer months, yeah. And as I said it's my physical condition at the moment is the only thing that's holding me back.

Interviewer: Yeah. Yeah.

Respondent: Yeah.

Interviewer: And if you wanted to be more active are there features of your area that might help if they were more aesthetically pleasing or attractive?

Respondent: No. I'm happy with the environment that I live in. Yeah.

Interviewer: Yep. And the last area is safety, and we've touched a bit on that in terms of road safety and also personal safety with dogs, is there any other things that you'd like to talk about, about safety in your area?

Respondent: Oh, not really.

Interviewer: That's the two main things?

Respondent: Yeah, the dogs and traffic is about the only thing.

Interviewer: Yeah. So personal safety around dogs – does that influence whether you're physically active or not?

Respondent: Yeah.

Interviewer: Yeah. Much? Very much?

Respondent: It has done, yeah.

Interviewer: And does road safety influence whether you're physically active or not – and you've actually said that. [Laughs].

Respondent: Yeah. Yes.

Interviewer: Yep. And if you wanted to be more active are there personal or road safety issues that if addressed might help you to be more active?

Respondent: Yeah. Mainly on... it's only on the main roads, because it's just finding areas to...

Interviewer: Where you... yeah.

Respondent: ...where you can go.

Interviewer: So in [town] itself it's a bit safer?

Respondent: It's limited, very limited.

Interviewer: But limited?

Respondent: Yeah.

Interviewer: Whereas as soon as you... like everything's on a main road, and just dirt.

Respondent: Yeah.

Interviewer: Yep. OK. So having more options around that would be helpful?

Respondent: Yeah.

Interviewer: Hmm, as well as people keeping their dogs in their yards would be helpful.

Respondent: Yeah. Yes.

Interviewer: I mean it's not pleasant even if they're in... fenced in, if they come barking at you a bit.

Respondent: Yeah. Yeah.

Interviewer: OK. So just one last question before we finish up – is there anything else that you'd like to tell me today about where you live, your physical activity, or the physical environment, that we haven't covered?

Respondent: I don't... I think we've just about covered most of it.

Interviewer: Uh-huh.

Respondent: Yeah. Yeah.

Interviewer: Uh-huh. Thank you very much.

Interviewer: OK. So I thought we might start a bit by talking about where you live, OK? So can you tell me what it's like living around where you do – this is in [town]?

Respondent: Yes.

Interviewer: Yep. And your likes and dislikes?

Respondent: I like living at [town]. I'm originally from [city],[state], and I've been here 20 or so years. I like living at [town]. I live with my family, my husband; he was an [occupation] before I came, so he had the farm that we live on now before I came.

Interviewer: Ah, OK.

Respondent: So we live on an apple orchard, we've got two farms.

Interviewer: Oh, beautiful.

Respondent: So we have the hills, and we have lots of open spaces around us. So we're not hemmed in – we don't actually live in the little town of [town].

Interviewer: Yep, so you're on a property.

Respondent: Yes.

Interviewer: Is it just outside on the outskirts of [town]?

Respondent: Yes, we're on the top of [x] Road, right at the end.

Interviewer: Ah, yep. Yep. Yep. So how far would that be out of [town]?

Respondent: Well we're [x] Road, so we're [x] kilometres from the [x] Road, which is down in the town.

Interviewer: OK. Yes, yes, the main intersection down there.

Respondent: Yeah. Yep.

Interviewer: Yep, OK. And you've obviously got a property – what sort of size property?

Respondent: Oh look, I can't tell you how big, but...

Interviewer: Just broadly guess work?

Respondent: Ooh...

Interviewer: Within the sort of... is it 20 acres, or 200 acres [laughs], you know that sort of thing.

Respondent: Oh, probably just over a hundred.

Interviewer: Yep. Yep.

Respondent: I think that's right, just over a hundred putting the two together.

Interviewer: No, that's absol... yep, yep.

Respondent: Yeah. And its nice being down there, because you've got the nice fresh air, spacious, you haven't got your neighbours too close to you.

Interviewer: Hmm, hmm. Hmm.

Respondent: Yeah, not like the city.

Interviewer: How long have you lived there?

Respondent: Probably 20 years.

Interviewer: OK.

Respondent: Yeah, when I... I went... I lived there when I first came from [interstate city].

Interviewer: OK. Yep. And your reasons for living there?

Respondent: Because of our orchard.

Interviewer: Yes. So that was the family orchard?

Respondent: Yes, it was my husband's family. Yes. Yeah, so that's why we lived there.

Interviewer: Yep. OK. Now, as I was saying a lot of this research has been done in urban areas, and people often refer to their local neighbourhood, and I'm interested in understanding what the term neighbourhood may mean for women who live in rural areas, and indeed if it's relevant. So what does the word neighbourhood mean to you?

Respondent: Well a neighbourhood would refer to our neighbours that are actually around us, around the property, not necessarily who's in [town]. So that's... so we've got two farms – we've got our home farm on [x] Road, and then the second farm is down on [x] Road, so we would sort of say that the people that live around the [x] Road property are our neighbours as well, and we have to keep in contact with them because of different issues.

Interviewer: Yeah. And that would be what, how many kilometres apart would the home farm be from the other farm?

Respondent: Oh, probably one and a half kilometres.

Interviewer: Yep, yep.

Respondent: Yep. Not very far at all.

Interviewer: OK. So just if you needed to put a distance on it, so you've got the one and a half kilometres between the two blocks, and then...

Respondent: So yes, we would say that our home farm and [x] Road, we would say all those people around in that area, one would be... that's our neighbourhood.

Interviewer: So it'd be a couple of kilometres? About three kilometres?

Respondent: Yeah. Yeah.

Interviewer: Yep. OK.

Respondent: We'd encompass all of those.

Interviewer: Yeah, yeah. In – what do you call it – in radius or diam...

Respondent: Radius. A radius.

Interviewer: Yeah, radius. Yeah, yeah, yeah [laughs].

Respondent: [Laughs]. And also when I first came over here there was a gentleman that lives just down the road, and he did neighbourhood watch. He was the leader of it, and it was just for our little area.

Interviewer: Hmm, hmm, that's very interesting.

Respondent: So not... it didn't... I don't know how far down the road it went, but we did neighbourhood watch just in our little area at the end of [x] Road.

Interviewer: Yep, yep. Oh right, that's very interesting.

Respondent: So that might be helpful.

Interviewer: OK. And the other word that often people talk about is community. Now, what's community mean to you?

Respondent: Community means a lot actually for us. For me, I use to... when I first came here my husband and his family belonged to the [x] Church, the [x] Church, so we got to... I got to know everybody in the district, and it was mainly the older people, the Elders. And then when we had our children they went to [school] at [town], and so of course I was very involved with the P&F, so and other people's children, and other families. So to me that's community.

Interviewer: Hmm, hmm, that's community as well.

Respondent: Definitely. Yeah.

Interviewer: So if you needed to put a distance around that, what would... is that more the whole of [town]?

Respondent: Yes.

Interviewer: Yeah.

Respondent: Well there's... the school is just on the outskirt, but there are people that come from all over the [town] to go to [school].

Interviewer: OK. So would you consider it as a more... more than [town]then as community?

Respondent: Yeah, because we have people coming up from [town], [town].

Interviewer: OK. So again if we needed to put a distance on that, sort of how far would it be to [town] from town]? Don't know?

Respondent: 40, 40k's.

Interviewer: 40? Around 40? Yep. No, that's alright.

Respondent: 40k's. But it's sort of...

Interviewer: And [town] about oh, 20 odd isn't it?

Respondent: Hmm, yes. Yeah, something like that.

Interviewer: Uh-huh.

Respondent: That's what I would call community. I call that my community because of my involvement with the school, with those families. Our children did go to [school] kindergarten, but only for kinder. And I still sort of think that the families that go to that school are still community – we're all a community, we all work together. You've got different groups that live in [town] that do different things. We're still a community.

Interviewer: Yeah. So community is defined more by the interest that pulls you together?

Respondent: Yes.

Interviewer: You know, like those... yep, yeah, and the geographic comes from that, depending on what it is.

Respondent: Yes. Yes, that's pulls us together because it's children, where you live, and the work that goes on around, the forestry, and of course the agriculture, which is us.

Interviewer: Yes. Yes, a very important part of it.

Respondent: And even though our girls still... they come to town now, we still feel we're part of that community down there.

Interviewer: Broad community, because they've still got... well, you've all got your friends down there.

Respondent: Yes. Yeah.

Interviewer: Yep, yeah, because it's come from those connections.

Respondent: Yes, that's right. Uh-huh.

Interviewer: Yeah. And I guess some of your daughter's friends probably ended up coming up to town as well.

Respondent: They did, yes. Not very many, but...

Interviewer: But a few, hmm.

Respondent: ... a few, yeah.

Interviewer: OK. Lovely. Now, I'm going to talk a bit about physical activity now, and as I said it's very broad for each individual [laughs].

Respondent: [Laughs]. Yeah.

Interviewer: So what does the term physical activity mean for you?

Respondent: Physical activity means to me taking time out from your housework, or whatever you're doing around your house that's a chore or a job, and going and doing something special for you, like going and playing a sport, going for a walk – doing something for your body.

Interviewer: To enjoy for you. Yeah. Yep.

Respondent: Yes. Yeah. And that it's going to have a health benefit.

Interviewer: Yes. Yes. That's true.

Respondent: Or a mental health benefit [laughs].

Interviewer: Absolutely. [Laughs]. No, that makes a lot of sense. And do you consider yourself to be physically active?

Respondent: I'm not physically active in the way that I use to play hockey, but I don't anymore – I haven't done so since we built our house and had our children.

Interviewer: Hmm, hmm. This often changes things doesn't it, different times of your life. Hmm.

Respondent: Yes. Yes, definitely. I don't play any sport, but I think I'm physically active in that I run my children around everywhere.

Interviewer: [Laughs]. Yes.

Respondent: And when I'm at home you're not sitting still, you're always doing housework.

Interviewer: That's right.

Respondent: And I use to work three days a week here on [x], and now I'm five days.

Interviewer: Yes. So you have less time for yourself.

Respondent: So I have less time to do all that.

Interviewer: Hmm. It's really the weekends for everything.

Respondent: So I find my weekends are taken up at the moment – this week is the last weekend of netball, because I bring my youngest up.

Interviewer: Hmm, for the... for your girls.

Respondent: Yes.

Interviewer: Oh, she plays up here?

Respondent: Yes, at [x] Road.

Interviewer: Ah. Oh, of course, because she goes... it's through her school.

Respondent: She's playing for [suburb], so yes, we come up to [x] Road on Saturdays, and that takes up our day. So I might get home at four, something like that. But if the girls want to come to town we'll walk around the city, we'll go shopping, that's the time to take them, and I find that can be quite physical.

Interviewer: Absolutely. No, [city] a very walkable city that way.

Respondent: It is. You have to walk to go from here, and everything's not in one place.

Interviewer: No. No. And it's some nice up and downs too. [Laughs].

Respondent: Yeah. Last year, while I was here, we did the [x] – and that's where we all had to wear little pedometers, and for about three months we had to put in how many steps we'd taken every day. So I did that, and I found that was not playing a sport, but that was physical activity for me because I didn't walk a lot of steps every day, and it was quite amazing – we had to walk 10,000 steps a day, or try to, that was the aim.

So on the weekends, if I... you know normally the weekends I'd sit down, have a rest, do the housework, but I wasn't getting many steps, so I would go for a walk down the road – down to [x] Road, down to our other farm, and as I would go I would pick up the rubbish on the side of the road, and then walk all the way back. And then I'd get my 10,000 steps, and I would be exhausted. [Laughs].

Interviewer: [Laughs]. Yes. As well as doing your community bit as well.

Respondent: So that was another thing... yeah. So that's about all I do. I don't do any at the moment – I haven't been walking. Yeah, it's mainly just all housework and running around, and work, doing work here.

Interviewer: Yeah, you don't have the time.

Respondent: I find in records it's quite physical because we're getting files in and out, and we're...

Interviewer: Oh, OK.

Respondent: Where it’s different to Reception – you sort of... you’re moving around a lot.

Interviewer: Yes. Yes, you’re not sort of sitting in one place.

Respondent: Yeah, so you have to watch when you bend down, make sure you don’t pull anything. We get a lot of paper cuts, and we use a lot of hand cream. [Laughs].

Interviewer: Yeah, I know, they’re disastrous, aren’t they. [Laughs]. Yeah. OK. So I’ll go on to talk a bit more about different parts of your life, but just to finish this little bit off, where does physical activity fit into your life? Is it of no interest; is it a priority, high priority?

Respondent: For me it’s not really a priority at the moment. I just look after the girls physical activity needs, and that’s either sport, or if they have to go somewhere. I have one daughter that doesn’t play sport though – I’d like to get her into doing something, and she does go for walks around the orchard. But for me, I think I get enough physical activity just doing my job, and the housework, and shopping, and running around.

Interviewer: Hmm, hmm, absolutely. Yeah, it’s in your daily life, and that’s...

Respondent: Yeah. I have got a friend here that still plays hockey, and she’s often said would I like to come and play, but I just don’t think I could fit it in – I just haven’t got the time.

Interviewer: No. Well, that’s one thing that’s come out is that women are really talking about the activity they do in their day, not separating out a really specific activity that you wait for the end of the week to do.

Respondent: Yes.

Interviewer: It's what you can do...

Respondent: And yet there are ladies here that will go to the gym first thing in the morning, and I have a friend that comes up from [town], she goes to the gym and then she comes here to work.

Interviewer: Wow.

Respondent: And there are others that go during their lunch break. But for me I like to have my lunch break – either have a rest...

Interviewer: As a break.

Respondent: ... have a break, or go and... there's usually an errand that I have to run, so I go and walk around the city.

Interviewer: Yes. Yes, I've always wondered how people fit anything in – there's always something that you've got to do.

Respondent: I don't know how they... I don't know either. [Laughs].

Interviewer: [Laughs]. Especially with an active family. Yeah.

Respondent: So no, I don't do any gym work or anything like that [laughs].

Interviewer: That's fine. I'll let you into a secret – most people don't. [Laughs]. Not in this study. So OK, in our research when we think about physical activity, we're thinking of any activity that lasts for at least ten minutes – so as we were saying that's the evidence of health benefit

effect – and causes your body to work a bit harder. You might feel your heart rate go up, you might feel a bit warmer, and walking around [city] a good [laughs] test of that one.

Respondent: Yes. Yeah.

Interviewer: And, or you might breathe a little heavier, huffing and puffing. So thinking about that sort of physical activity, and in the last... just in the last two weeks, just as an indicator, I just want to talk about different parts of your life – not that we're trying to separate out bits, but we just don't want to miss things. OK? So in the past two weeks have you been active at all at work for at least ten minutes at a time?

Respondent: That's made me puff? Not really.

Interviewer: Oh, OK.

Respondent: About the only time I think I can...

Interviewer: Or get... keep warm.

Respondent: ... is walking to and from the car.

Interviewer: Oh, OK.

Respondent: Because... oh, I beg your pardon, there... [daughter] is up at [school], and I had to run twice up there – once to visit her. There was an art display up there, so I walked up there during my lunch break, and the hill up there past [church] is very steep, and I got halfway up there and you just feel like gravity is sucking you into the ground, but I had to stop because I was [expressive noise], and the heart rate was going, yes, and I was warm.

When I got to the top of the hill I had to stop, have a drink, take my jacket off, and get my breath back. So I did that, yes, in the last couple of weeks.

Interviewer: OK. So just thinking about those couple of activities, like the walking to and from the car, and the running up...

Respondent: Yeah, [laughs] up to [school].

Interviewer: ... up to [school].

Respondent: Yeah, [x] Street.

Interviewer: And also you mentioned like your job isn't sort of sitting down – it may be more steady, rather than...

Respondent: It's half sitting down, and then the other half is putting files away, and you're up and down.

Interviewer: Yeah, yeah.

Respondent: You're not... Reception can be the same – you're sort of up and down, up and down, because you've got people coming to the counter.

Interviewer: Yeah. But you're actually lifting things as well.

Respondent: Yeah, moving things around.

Interviewer: So think about that – it may not be vigorous exercise, but it's pretty steady.

Respondent: No, it's not vigorous, but yeah, it's steady.

Interviewer: Yeah. OK. So thinking about those three things, could you tell me what the intensity of them is? Like, we've just talked about records being probably steady – the other options for the other two are steady, moderate, or vigorous. So walking to and from the car?

Respondent: Moderate.

Interviewer: Uh-huh. And the other one sounds a bit more... [laughs]...

Respondent: Walking to [school] – oh, that was pretty strenuous.

Interviewer: Yeah. [Laughs]. It sounds like it. OK, so pretty vigorous.

Respondent: It was. Yes.

Interviewer: And the duration – how long would those activities have taken?

Respondent: Well records is all day. Walking to the car is probably seven minutes – between seven and ten minutes. And walking to [school], oh about 20 minutes I'd give that – 20 minutes.

Interviewer: Yep. And how often would you have done that? Records are obviously five days a week.

Respondent: Every day, uh-huh.

Interviewer: OK. Walking to and from the car?

Respondent: Car park – every day. And [school] is...

Interviewer: A couple of times you mentioned?

Respondent: Yeah, it's... I can't see that I'll be walking up there...

Interviewer: [Laughs]. You hope [laughs].

Respondent: [Laughs]. Not for a while [laughs], unless something goes... something happens.

Interviewer: OK. Alright. Now when obviously we've talked about records being all day, the going to and from the car is every day – the five days.

Respondent: And on the way home I'll shop.

Interviewer: Oh!

Respondent: I'll stop at [supermarket].

Interviewer: To squeeze something that's...

Respondent: Walk around the supermarket, but that's quite... that's not... I don't... it's not leisurely, but I don't dawdle.

Interviewer: Do you have to stay nine to five here, or do you have shorter days?

Respondent: No, it's twenty past eight, til five.

Interviewer: Oh, long days.

Respondent: Uh-huh.

Interviewer: Hmm, hmm, OK. So, shopping as well. Yep, OK. And is this what you usually do, these three activities, or were they one off? Obviously work is usual, walking to and from the car is usual.

Respondent: It's usual. Walking to [school] is not usual [laughs].

Interviewer: Yeah. [Laughs]. But can happen from time to time.

Respondent: Yeah. Look, I haven't been for walks around the orchard because it's winter. I haven't really done anything at home because you're getting... you're leaving in the dark, you're getting home in the dark.

Interviewer: That's OK.

Respondent: So...

Interviewer: We can get onto that area.

Respondent: OK.

Interviewer: We'll just look at work right now.

Respondent: Yeah.

Interviewer: And so with those three activities at work, does it change depending on the season or time of year? No, not really?

Respondent: Not really.

Interviewer: No.

Respondent: Uh-uh.

Interviewer: And if you'd wanted to, is there anything that would have helped you be more active at work? Like more time, or somewhere to be active?

Respondent: Not really.

Interviewer: Hmm, although you were talking about the [x] – what was it – [x]..?

Respondent: Oh yes, the [x]... the [x]... was it the [x], or the [x]?

Interviewer: Yeah. Yeah, often workplaces have something like that, that they commit to.

Respondent: Yes. I don't know... I don't think they're doing it at the moment, so...

Interviewer: This year, hmm, hmm.

Respondent: ... that would be... well that was a way for me to be active – that was last year – and to be involved in something, and to try and make an effort, and just...

Interviewer: Yeah, so it helps if there are some opportunities at work?

Respondent: Yeah.

Interviewer: Uh-huh. Uh-huh.

Respondent: I think... yeah, it did.

Interviewer: Hmm. OK. So in the past two weeks have you walked or cycled for at least ten minutes at a time to get to or from places? Now, we've already talked about getting to and from work, and also the [school] –

getting to [school]. OK, now this is to and from, because we'll look at leisure activities as well as a different category. So this is more destinations – so any other times that were particular walking to and from?

Respondent: Not really.

Interviewer: Or we've covered that by every day?

Respondent: I think so. Walking from the car to get to...

Interviewer: Absolutely. Fine.

Respondent: ... to go the netball court, and then walk back to the car – it's not very much [laughs].

Interviewer: Yes. [Laughs]. No, that's OK. And if you'd wanted to, is there anything that would have helped you use more active forms of transport – like walking? Like, this would be in [town], if you were wanting to walk down to the shops, or something like that, or cycle down? I don't know if cycling... is cycling your thing, or it's more walking?

Respondent: No.

Interviewer: Walking?

Respondent: Walking.

Interviewer: Yep. Yep.

Respondent: But no, I don't... I've never ever walked from home to [town], to the centre of [town], but I did get a phone call from the Heart Foundation, and they have signed me up to walk from the bottom of [x] Road to the top of [x] Road, and do the collection – the doorknocking.

Interviewer: Oh, great. OK.

Respondent: That's in September, I think.

Interviewer: Oh, wow. Yes, because they've got a very active [x] Walking Group in [town], obviously during the day, during the week, but yeah.

Respondent: Yes. Yes, it's Tuesdays, isn't it?

Interviewer: Hmm. It's a very big group I got told. Hmm.

Respondent: Hmm, yes. You're right.

Interviewer: They said it's either biggest in Australia, or they have the oldest members in Australia.

Respondent: It might be the oldest members...

Interviewer: There's a few in their 90s.

Respondent: Yes, my husband's great Aunt, she's one of the walkers, but I don't know if she's still walking because she just... her hip's going.

Interviewer: Oh, poor love. Yeah.

Respondent: Yeah. And she's as healthy as.

Interviewer: That's pretty remarkable.

Respondent: Yep.

Interviewer: That's good. That's good. OK. So...

Respondent: So no, I haven't walked down to [town].

Interviewer: That's fine. That's fine. So those are the sorts of things that one's on about. And OK, you haven't – you know – can I ask you a bit about why you might not of?

Respondent: Usually because I have to go to [town] to buy groceries, and I can't walk all the way back [laughs] with groceries.

Interviewer: No, you can't.

Respondent: Or put fuel in the car.

Interviewer: No, you can't. I find though – like I live in [suburb], so it's not very far, but going to [x]Street, and you go ooh, I should really walk or take the bike, but usually I don't... you know, you go to the shops and you've got full bags of shopping, it doesn't work that way. [Laughs].

Respondent: Yes. No.

Interviewer: No.

Respondent: I'm too far out. Perhaps if we were closer, because I do see a lot of people down closer to [town], they'll walk. Yeah, but I'm just a bit too far out of it.

Interviewer: Yeah, absolutely.

Respondent: And I'm uphill.

Interviewer: Yes. Yeah, dragging shopping back uphill is not fun.

Respondent: No. [Laughs].

Interviewer: No, it's not fun at all. [Laughs]. I don't think it's very helpful to the body at all [laughs].

Respondent: It'd be alright if it was a nice day – I sometimes think me and the girls should go for a walk down to [town], then get [husband] to come and pick us up [laughs] and bring us back.

Interviewer: Well that's an idea. Yes.

Respondent: Just to go for a walk, because you see more when you're not in the car.

Interviewer: Yeah. That's true. Yeah, that's an idea. I know one woman who lives in [town], they live a bit out too, she's got a young child, so there's... yeah, so again she can maybe get down a bit, but going back up the hill's too much, so she organises her partner to go and pick her up.

Respondent: Yeah. Yeah. It sounds good.

Interviewer: Yes. [Laughs]. OK. Now, if... OK, so in the past two weeks have you been active around the house or the yard – now that's at home, inside or outside – for at least ten minutes?

Respondent: Not in the last two weeks, but very close – just recently actually, probably a week before, I did a little bit of gardening.

Interviewer: Oh, I'll count that then.

Respondent: So we pruned – what did I do – I think I raked leaves, and pruned the roses. I like to try and get out in the garden. I find that is my physical exercise.

Interviewer: Yeah. Are you on a flat block, or a slopey block?

Respondent: We're flat around our house, and then everything's downhill.

Interviewer: Hmm, downhill.

Respondent: Or uphill behind us.

Interviewer: So if you go... yep, yep.

Respondent: Yeah, so around the house is flat, but I do like gardening, and winter's not the best time to garden.

Interviewer: No, it isn't. It isn't.

Respondent: There's not a lot you can do. So yeah, I would be more active in the better weather.

Interviewer: Hmm, hmm, and that's what women often say too, that... yeah, yeah. Now, OK, obviously there's the housework as well, housework and the gardening, thinking of those two activities, what would have been the intensity of them – steady, moderate, or vigorous?

Respondent: Steady.

Interviewer: OK.

Respondent: Yeah, steady.

Interviewer: Yep. And duration – how long would you have gardened for?

Respondent: Half a day.

Interviewer: Yep. And housework?

Respondent: I'll just start at one end and then keep going, and then I'll just keep going and clean up as I go, so if I get tired I can stop.

Interviewer: Yep, you can stop. That's a good idea.

Respondent: Or if something comes up that I have to do something else, I can just stop, put the tools away, and then I haven't left a little trail, [laughs] that I have to go back later. Yeah.

Interviewer: And the... OK, and with the housework, how long would you have done that one for?

Respondent: Oh, include washing the car in that.

Interviewer: Oh, yeah, absolutely.

Respondent: On Sunday I washed the car – did the housework, washed the car, and I also cleaned the windows on the house. So you go around with the hose and the truck wash, and the broom...

Interviewer: Hmm, hmm, that's quite an effort.

Respondent: ... hmm, and the hose. So, sorry, what was your question? [Laughs].

Interviewer: Just how long you might have done the housework for, including all those...

Respondent: All day. [Laughs].

Interviewer: ... all of them. Goodness. [Laughs].

Respondent: Yeah, spaced out over a day.

Interviewer: OK. Yeah.

Respondent: The day, yeah.

Interviewer: So the frequency of that – those two activities, the housework – how... you would do that every week?

Respondent: All day. Yes.

Interviewer: Yeah. So it's often a Sunday activity?

Respondent: Washing the car would be every couple of weeks. Windows perhaps every month, every couple of months – usually after we've had bad weather.

Interviewer: Yeah, I know.

Respondent: Because it blows in under the verandah.

Interviewer: It's very discouraging isn't it, you just get them all clean and we get this windy wet weather again.

Respondent: Yes. And we have a fantastic view of our valley, and the orchard, and our neighbours, so it's nice... and we've got heaps of windows, so I like to keep them clean, so they look nice when you look out.

Interviewer: Beautiful. Yes. Yes. And the frequency of the gardening, how often would you be doing that?

Respondent: Not so much in the winter, but in the spring and the summer, and when I'm at home, I like to get out there and tweak – I call it tweaking – go pruning, or raking, or just tidying up.

Interviewer: Fortnightly? Weekly? On the weekends?

Respondent: Yeah.

Interviewer: Yep.

Respondent: Weekends, when I'm home.

Interviewer: Yep.

Respondent: Yep, weekends.

Interviewer: So, and obviously it's why you might have done that is it's after... you're not working at that time.

Respondent: Yes, that's right. Yeah, and when I wasn't working...

Interviewer: Does that change in summer at all, when you have a bit more light?

Respondent: Yes, it does, because yes, I do find that I'm outdoors – that's the other thing about Tasmania, it's very dark in winter, and it's... I love the daylight saving because you can get home, you can do things in the light afterwards.

Interviewer: Yeah, that's the other side of it – we have that lovely light.

Respondent: Definitely.

Interviewer: That's why as soon as it gets to be – what is it, the 21st, second, of June, OK we've just got into winter, but it's when the solstice – and so you go oh, something to look forward to, we get more light from here on. It takes a little while, but we get it back.

Respondent: Yeah. And the other thing is when we usually start picking the fruit, we have cherries, and I'll start picking those. We have an acre of cherries, and I'll pick those myself, with a little bit of help from the girls – that's about mid February, for about three weeks. That's when I'm not working. And when I was working I would pick the days I wasn't.

And then we start picking the apples, which I don't pick apples, but I might help John tag bins, run around – I do a lot of the running around, when I wasn't working, when I wasn't here. So that was quite strenuous.

Interviewer: Yep. And I imagine the cherries are fairly specialised in terms of they need to be taken care of in picking them.

Respondent: Yes. Yeah.

Interviewer: More so than the apples. Yeah.

Respondent: Oh, the apples are just as bad I can tell you.

Interviewer: Really?

Respondent: Yeah.

Interviewer: OK.

Respondent: You can't bruise... you can't... oh.

Interviewer: Oh, yeah. And thinking about those activities, the gardening and the housework, who would you have done them with – yourself, or with others?

Respondent: Myself.

Interviewer: Uh-huh. And OK, and if you'd wanted to, is there anything that would have helped you be more active around the house or yard, inside or outside? Things like more time, or... yep.

Respondent: More light. [Laughs].

Interviewer: More light [laughs], OK.

Respondent: Yeah, more time.

Interviewer: Absolutely. OK.

Respondent: Yeah, definitely.

Interviewer: Now, have you been physically active for at least ten minutes during your leisure or spare time? Now, I know you were talking about walk...

Respondent: In the last two weeks?

Interviewer: Uh-huh, walking down between the properties, and around the orchards.

Respondent: Oh, not really. No, not in... no.

Interviewer: OK.

Respondent: The weather's been too bad. We've had very cold snowy rain.

Interviewer: Yeah, it has. We've had very cold, haven't we? You've had a fair amount of snowy type weather down... hmm.

Respondent: Yep. And my week... yeah, I just haven't had time.

Interviewer: Uh-huh. No, that's fine. And it would be on the weekend with the light that we're in, in winter anyway.

Respondent: Yep.

Interviewer: And OK, does that change depending on the season and time of year?

Respondent: Uh-huh. Yes.

Interviewer: Hmm, OK, so what happens in...?

Respondent: If it's still light, and it's a nice evening, come home, when I was... see, I've only been working for the last 18 months, but previously before that well, if the weather was nice I'd be outdoors.

Interviewer: Yeah. Yep. So particularly in summer, yeah.

Respondent: Yeah, spend more time outdoors. It's just too nice to be inside.

Interviewer: Yep, absolutely. OK.

Respondent: Gardening, or running around doing things on the orchard.

Interviewer: Yep, yep. And that's when you'd more likely take your walks as well.

Respondent: Uh-huh. Because what happens is the husband, my husband, when it's lighter he spends more time out in the orchard. He comes... it takes him longer to come in. Yeah, we've got cattle – we move cattle.

Interviewer: Ah, you've got cattle as well. That was a... orchards would be hard... the amount of work that you've got to do in winter after the fruit production, and pruning and stuff, when you've got shorter hours, is hard. OK, and if you'd wanted to, is there anything that would have helped you be more active in your leisure or spare time?

Respondent: If I got my daughters to help, give them chores, I reckon I would have more leisure time.

Interviewer: Yeah, you would actually have a bit of time.

Respondent: Yeah.

Interviewer: It sounds like life's a bit busy right now.

Respondent: It is. I don't put too much pressure on the girls because I know they've had a big week travelling up and down on the bus.

Interviewer: Yes. Yeah, they must get tired.

Respondent: They also have a limited... less time to do their homework than their peers, because they're not travelling.

Interviewer: Than most others, yep.

Respondent: So they use that time on the weekends. They also use that time to relax, at night they'll be watching TV and I'll be working away.

Interviewer: That's important too, isn't it? Hmm.

Respondent: But then on the other hand, look if I'm busy doing something like washing the car, or busy doing some housework, they... if I say to them, "Would you like to make a pizza for tea, or can you make up a salad, or can you..." they will do that.

Interviewer: Oh, good.

Respondent: They're more inclined to do that than... I'm slowly getting them around to putting things in the washing machine, and hanging things out. So it does slowly, but I haven't put a huge amount of pressure on them.

Interviewer: Yeah. No, that's a big activity in itself, getting teenagers to...

Respondent: It's sort of a balancing...

Interviewer: Yeah, it’s a balance. Yeah.

Respondent: ...yes, it’s a balancing act getting them to pick up their clothes, or just... like I like to have the beds made every day, and if the girls don’t do it I’ll do it. But now that I’m working five days I don’t do it, because I think they should do it.

Interviewer: No, they would have to. Yeah.

Respondent: And on the weekends I think right, they should do it, so I don’t – unless I really think oh, I’ve got the time, I’ll go and do it. So it is a balancing thing.

Interviewer: Yeah, OK.

Respondent: I try not to be too mean.

Interviewer: Now, the last area is physical environment, so this is about the areas that you live, work, and play in.

Respondent: Righto.

Interviewer: OK. So when we talk about physical environment, I mean all the physical things that surround you – roads, trees, houses, shops, traffic – and some of these things are things that people in urban areas have said are important, and that they might actually... for physical activity. So we’re interested in finding out whether they’re relevant for you who live in rural areas. So first of all, can you tell me a bit about how easy or difficult it is to be physically active in your area?

Respondent: In [town]?

Interviewer: Hmm, in [town].

Respondent: I think it would... if you wanted to go for a walk it would be easy. If you wanted to play a sport you can't, because you've got to come to [city], or you have to go to [town] and join a group.

Interviewer: Yep. Yep, yep. Yeah, I've heard... people have actually said, you know like... and one woman was saying she likes to play tennis, so she has to come up to [city] really.

Respondent: Yes. I think Council were thinking about building a tennis court down there, but nothing happened. I think they've built [community centre] instead.

Interviewer: Oh, OK.

Respondent: Yeah, they've gone in for that kind of thing, community buildings.

Interviewer: Yeah, yeah, yeah. Yep, yep.

Respondent: Yeah, so if you wanted to go to [town] Sports Centre, that's usually an evening activity, or they have activities during the day which I'm not available to because I'm working, and I don't get home in time, so that rules me out. My weekends – I can't go round there because of netball at the moment, and Sunday's they're closed. In the city, if I wanted to go...

Interviewer: So you don't really have access other than...

Respondent: There's no sports clubs down there except golf – if you want to go and play golf.

Interviewer: No. You probably... by the time you finish in the evening and get home there wouldn't be any time to, no.

Respondent: No. No. And that would be the same like at [town] – they have the [community group], but it's mainly for, well mainly the kids.

Interviewer: What's [community group]?

Respondent: The... at [town], it's near the... behind the football oval, the swimming pool.

Interviewer: Just explain to me what [community group]?

Respondent: [community group] is [x].

Interviewer: Ah, OK. Yep, OK.

Respondent: Yes, I think Rotary and the Council... oh, or the [community group] built it. And that's... its very similar activities there what happens down at [town].

Interviewer: Oh, OK.

Respondent: At the sports centre.

Interviewer: So you're saying in terms of easy or difficult, it's easy if you want to go walking.

Respondent: Yes.

Interviewer: You can sort of...

Respondent: Or cycling, or horse riding – I don't have a horse, but I don't have a bike.

Interviewer: Would you walk from your house if you wanted to?

Respondent: I would go for a walk around the orchard. I might go down, say for a walk down to [x] Road, depending on the light. I wouldn't do it in the dark.

Interviewer: No.

Respondent: I would do it in the finer weather.

Interviewer: And like, it would be OK to walk along... I presume it's walking along the road?

Respondent: Yes, you're walking on the roads.

Interviewer: It's a quiet enough road?

Respondent: Yes. Yeah.

Interviewer: OK.

Respondent: It's usually... it's pretty quiet, yeah.

Interviewer: Yep, OK.

Respondent: In the city, if I wanted to get some exercise in the city I would go for a walk. I would go for a walk if I wanted to, like you say, get the old heart beating – I'd walk up to [school] and back [laughs], or walk up those hills.

Interviewer: Guaranteed. Yes.

Respondent: And so that's how I... I wouldn't go to a club, or a physical place.

Interviewer: Yep. No, that's really clear.

Respondent: And playtime, our family has a shack down at [town], so if we're down there I try and go for a walk every day. Everybody walks along the road, and that would be...

Interviewer: So that's OK.

Respondent: So that's the playtime.

Interviewer: Yep, OK. Yep, yep. Yep. So in general walking is OK, it's fairly easy. It's more difficult if you want to do an organised activity, sort of?

Respondent: Yes.

Interviewer: Yep. OK.

Respondent: Yeah, definitely.

Interviewer: Now, OK just a couple of areas we'll look at – first of all is availability and accessibility of places to be active – so it's a bit like what we've been talking about. If I were to ask you about places to be active in your area, what sorts of things would you tell me about? Now, you've already told me about...

Respondent: If you wanted to be active down at [town], well you can go walking. The other place, if you wanted to be indoors, would be the [town]

Sports Centre – you would go swimming, you could play netball, they have aerobics there. They also have this guy who does – oh, what do they call those – boot camps.

Interviewer: Oh, yeah, yeah. Yes.

Respondent: Boot camp; and I've seen women walking through [town] with a great big log over their shoulders.

Interviewer: [Laughs]. Really? Oh, no.

Respondent: Yeah. That's not for me, thanks.

Interviewer: [Laughs].

Respondent: So it's there. I mean there is something there.

Interviewer: Yeah. And [town]'s about five/six k from [town]?

Respondent: Yeah. Yeah.

Interviewer: Uh-huh. Uh-huh.

Respondent: It's not far, but that's during the day, and like I said in the evenings when – well, me working fulltime, I just can't get there.

Interviewer: Yeah, it doesn't... yep. OK. Alright. So accessibility – now, we've talked about the opening hours and things like that, and in particular at the sport centre over the weekends, and is it easy from a cost point of view?

Respondent: I think so. I think they try to make it so that it's available to everybody.

Interviewer: Yeah, the broader. Someone explained to me, who lived in [town] actually, and she was saying, because a lot of people mentioned the sports centre, they may use it or they may not, it depends, but the explanation she gave me was they tend to have – and I don't know if I've got it in the right order – for the older members of the community are more morning activities, kids afternoon activities, and adults in the evening.

Respondent: Yes, that's right, adults and youth.

Interviewer: If you can get there. [Laughs].

Respondent: Yes, that's exact... you're right, yes. They have swimming and water aerobics for the elderly, mainly in the mornings. Yes, you're right.

Interviewer: Hmm. So does... thinking about that, does having or not having places to be active that you can access influence whether you're active or not?

Respondent: Not really. I think if I wanted to be active I would find a way. I've always enjoyed my sport – use to.

Interviewer: Hmm, it's more a balance of life right now.

Respondent: Yes.

Interviewer: Yeah, in what you can do.

Respondent: Definitely. Hmm.

Interviewer: OK. If you wanted to be more active are there things or places that would help if they were available or more accessible?

Respondent: In [x]?

Interviewer: In [x].

Respondent: If we had a tennis court we might be able to hire that, because my daughter – middle daughter – is learning how to play tennis. I mean I use to play tennis, so we could hire it on the weekends, or have a go at that. No, nothing comes to mind. That's [daughter] sneezing.

Interviewer: [Laughs]. I thought you might know who it was. [Laughs].

Respondent: She's got a cold.

Interviewer: Oh dear.

Respondent: She sneezes every day, and she's sneezing a lot today. Oh no, that's about the only thing I can think of.

Interviewer: That's fine.

Respondent: Netball courts – if there were some courts or something, but if the kids want to do that there's... they've got the netball court indoor at [town] Sports Centre.

Interviewer: Oh, OK.

Respondent: Yeah, because they've got the pool, and then the netball court, which they use for all sorts of things.

Interviewer: It's a big centre that one. OK, destinations – if I were to ask you about places that you could walk to from your home, what sorts of things would you tell me about – i.e. shops, services, and recreation facilities, friends?

Respondent: So you want me to tell you what I would...

Interviewer: Yes, what you could walk... well, you've already told me that it's a bit far to walk to your local shop, especially as you're carrying things.

Respondent: Yes. But it would... if I didn't have a car I would have to do it.

Interviewer: Yeah. So you generally sort of get in a car – you would need to.

Respondent: Uh-huh. My friends don't live near me.

Interviewer: No.

Respondent: [friend] lives on [x] Road, so I'd get in the car and go and see her, or she comes in her car. [Laughs].

Interviewer: Yep, yep, yep.

Respondent: So we see each other. I would go for a walk. There are no shops up there, but there are just other farms, houses, about. Might walk down to the other farm, but that's not... it's very rare.

Interviewer: OK. So does not having destinations influence whether you're active or not? Does it help to have something within an easily accessible walking distance?

Respondent: I think so.

Interviewer: Like the tennis court that you would like to have in [town]? [Laughs].

Respondent: Well, like you hear on the radio, they try and get the children and the parents walking to school. I mean we've never... I've never ever walked to school with my girls because [school] was too far, and it's on the highway.

Interviewer: It's too far. Yes, two very good reasons.

Respondent: [school], they get dropped off on the bus, or I drop them off.

Interviewer: And [school]l's?

Respondent: Down at [suburb].

Interviewer: Yes. Hmm.

Respondent: Same with [school] for [daughter].

Interviewer: Yep.

Respondent: But I think if there were shops closer, yes we would walk. If the schools... well, if we lived in [city] and we could walk to school, yes we would. Yeah, it's just where we are. We're just... we're in the open.

Interviewer: Yep. And that's the nature of the rural area.

Respondent: Yes, that's right.

Interviewer: And women often say well, that's why I chose to live in the rural area was that I was away from – you know, not necessarily right next to shops and facilities, but... hmm.

Respondent: Uh-huh, uh-huh. Yep.

Interviewer: OK. And if you wanted to be more active, would it help to have more destinations within easy walking distance? And we've already said that it would if...

Respondent: I don't think it would... I don't think... because you're in a rural, no you've got to get in your car, and you've got to...

Interviewer: Yeah. OK. Yep, yep.

Respondent: Yeah, it's not really going to make...

Interviewer: No. No.

Respondent: I don't think so. [Laughs]. If that makes sense.

Interviewer: So looking at just a couple more areas – neighbourhood design – so if I were to ask you about the built or manmade features, the non-natural features of your area, things like footpaths and lighting, what sorts of things would you tell me about?

Respondent: Well there's footpaths and lighting down at [town], around the town.

Interviewer: Yes. Yes.

Respondent: Some places aren't lit, but yes there are footpaths and lighting, and there's parks – some nice parks down there. So if you wanted to be

off the road you could, but that's down in the town, in the centre of [town].

Interviewer: Uh-huh, uh-huh. And for you it would...

Respondent: [x] Road has no footpath; and [x] Road has no footpath. A lot... quite a few kids and people do walk up and down those two roads, but no footpath.

Interviewer: Yeah. Yeah. And you were saying it wasn't so heavily trafficked.

Respondent: Limited lighting – there's no lighting up where we are. I think the lighting stops at about [x] Road. So there's not a lot of lighting up on the [x].

Interviewer: Uh-huh. And so for walkability you were saying it's a bit far to walk into town, and it's also not flat on the way back.

Respondent: You'd be walking downhill into town, because we're quite high up, til you get to [x] Road – you're walking down [x] Road, then it's flat, so it'd be the same coming back. Walking around [town]... within [town] it's flat, but then around [town] you can walk up hills. There is a little section where you can walk up a hill if you want to.

Interviewer: Yep. So within [town] and the surrounds, the other thing is are the streets connected? Like, some places it's got a main road through, and there really aren't any streets off that connect with each other. And is [town] well enough laid out that you can actually sort of walk on it...

Respondent: Yes, we've got the town centre, and you can walk around this section [demonstrating], you can link back around via the[building], and then

back around [x] Road, and there’s all these little – well, there’s [x] Street, and these little streets, and then [x] Road comes along, and then [x]. But if you were going to go for a walk you’d want to walk around [town].

Interviewer: Yeah, you could do that, and it’s got various parks and little walks by the river that you could connect up with too.

Respondent: Yes. Yeah, yeah.

Interviewer: Yep.

Respondent: You could walk through.

Interviewer: OK, do the built features of your area influence whether you’re physically active or not?

Respondent: The built areas?

Interviewer: Do the built features – like not having footpaths, or you’ve already said the road’s quiet enough to walk on, not having lighting – does it affect you being physically active?

Respondent: Not really.

Interviewer: No.

Respondent: No.

Interviewer: And if you wanted to be more active, are there any built features in your area that would help?

Respondent: Well if I wanted to be... if I really wanted to make an effort, and I could walk around [town], because I know the footpaths are there, and the lighting. I would still wear a reflective jacket. Because I know [friend] walks around – she comes down from her place, and she'll walk around the park. There's a [x] walk, and she does a lap.

Interviewer: Uh-huh. Hmm, hmm, yeah someone else has mentioned the platypus walk. OK. Now the other side of it is the aesthetic qualities – the natural part of your environment – and if I were to ask you about the aesthetic qualities, or the attractiveness of your area, what sorts of things would you tell me about?

Respondent: I like where we live because I love birds, I love the peace and quiet, I love the birdsong, so walking down the [x] in the quiet is nice. I don't have to have features, and manmade features and things. I like hearing the wind in the trees, just blue sky – that's fine by me, and birdsong, and just open...

Interviewer: Yeah, you've got a beautiful view.

Respondent: Yeah, we live in a lovely area. So that's fine. That's good.

Interviewer: And any problems with... oh yes, you did mention that you... I was going to say any problems with rubbish, or litter, or graffiti – I just remembered you do sort of walk and collect...

Respondent: Rubbish actually, yeah. We get people driving up and down the road, and they just toss things out the window. It's mainly cans, alcohol and Coke. We don't get the KFC [laughs], because they normally dump that at [x].

Interviewer: Oh, another place, do they? Oh, OK.

Respondent: [x] – that's normally the dumping ground. I think it's just people coming up the road, like for a little midnight swansong, and then they just... or they're coming out of the... my husband says it's the forest, if they're ever doing any forestry work up behind us, it's the early morning, late night, late afternoon – he thinks they're tossing it out the window. I don't know. But I'll... like I've said, I'll walk down and I'll pick it up.

Interviewer: Yeah, it's a shame. I use to look after a bush block that was on a main road, connecting road between different parts of the hills, and you spent at least half your time picking up rubbish versus looking after the native little things. [Laughs].

Respondent: It's a little pet hate of mine, and I think if I can make a difference, just pick that up and it makes the environment and where we live a better place, I'll do it. My husband doesn't like it. He says, "You shouldn't have to take other people's rubbish to the tip."

Interviewer: No. It'd be nice of people woke up to it.

Respondent: But I... it annoys me when I drive past it, so I think I've got to drive past it, I've got to live here.

Interviewer: Yeah, you've got to live with it.

Respondent: I'll go and pick it up, and hopefully they won't do it again. [Laughs].

Interviewer: Yep. Yes. So do you think your local environment is aesthetically pleasing and attractive near your home?

Respondent: Yeah. Yeah, definitely.

Interviewer: And do the aesthetics of your area influence whether you're physically active or not?

Respondent: Yes and no.

Interviewer: Uh-huh. It's often a strange question to ask because this... obviously these questions got asked about people living in an urban situation, who may not have a pleasing environment. Whereas people who live rurally, that's the reason why you live there. [Laughs].

Respondent: Right. I must say, I must add, when I use to work at [suburb], when I first came to Tasmania, I would go walking during my lunch breaks because it was just so nice – it was quite a peaceful area down near the [x] Club. I can't tell you the road – [x] Street I use to work.

Interviewer: Yes, it's very lovely.

Respondent: But I use to like going for walks around the streets to look at all the old houses, the gardens, and it was quite... even though there were cars parked everywhere, there wasn't traffic, and it was quite pleasant. So I would find if you were walking around those areas, they can be quite a nice area to walk around as well. They would be aesthetically pleasing. So I got enjoyment out of that.

Interviewer: Uh-huh. So yes, it does. And if you wanted to be more active are there features of your area that might help if they were more aesthetically pleasing?

Respondent: Don't know. [Laughs].

Interviewer: It already is. [Laughs]. OK.

Respondent: Yeah.

Interviewer: The last area is just about safety. If I were to ask you about safety in your area, what sorts of things would you tell me about?

Respondent: Safety issues?

Interviewer: Uh-huh.

Respondent: Oh, lighting I suppose, and being able to get off the road. Like I said there's no footpath on [x]. And I do know in the winter, and there are young students walking from their houses to the bus – there's a bus that leaves at seven, and half past seven, and in the winter they're walking along the road, you can't see them.

So even when we do things on the farm, if we have to move cattle and we're on the road, be it in the evening or during the day, we wear reflective clothing. We have reflective jackets. I think safe... you've got to be safe, and no I wouldn't go walking in the dark unless I had a torch, or there was lighting.

Interviewer: And that's a very general comment actually. Everyone's saying that – no, I don't go walking about.

Respondent: It's just plain commonsense – don't leave yourself open to trouble.

Interviewer: Yep. So does road safety influence whether you're physically active or not?

Respondent: Slightly. Yep. Yeah, definitely.

Interviewer: Yeah, and you take precautions about it.

Respondent: Yep.

Interviewer: And does personal safety influence whether you're physically active or not?

Respondent: Yes, sometimes I think oh, you don't know who's about, but where we are its generally quiet. But I wouldn't go walking in the night, at night – I would go during the day, and I would make sure that my husband knew where I was going. The girls – I don't let the girls go anywhere on their own.

Interviewer: No.

Respondent: Even if they want to ride their bikes, I make sure there's two of them, and take their mobile phones.

Interviewer: It's just safety conscious, isn't it? Yeah.

Respondent: Yeah. Yep.

Interviewer: Yep. Because even in summer, people have said particularly living rurally on properties, you know snakes and things like that.

Respondent: We get snakes where we are, and a lot of people have dogs, and we don't have a dog – so if I know that there are dogs, and as I said to the girl at the [x], I said, "I'm not going in anyone's house where there's a dog."

Interviewer: No, fair enough. They should be under control and well behaved, but they're not always.

Respondent: Yeah. Yeah. And occasionally you get dogs...

Interviewer: And you don't know with dogs, and I've got a dog [laughs], so no, you don't know.

Respondent: Uh-huh. If we know that there's a dog down the road that can run out and jump at the fence, we know they're there, they can't get out, that's fine, but we'd cross over the other side of the road and walk past. But yeah, that is a bit of an issue. I'd be a bit worried.

Interviewer: Yep, fair comment. And if you wanted to be more active are there personal or road safety issues that if they were addressed might help you be more active?

Respondent: I don't know. If there was a footpath on the [x], but I don't think they'd put a footpath and lights right to our place, right up... there's aren't... there's not... there aren't enough houses up there to do that, to warrant the cost. So we just... we just adjust.

Interviewer: It's more if in [town] itself it's sort of accessible.

Respondent: Yeah.

Interviewer: Yeah. You just adjust to that.

Respondent: Yep.

Interviewer: Uh-huh. OK. That's fair enough. Just before we finish up, is there anything else you'd like to tell me today that we haven't covered about where you live, your physical activity, or your physical environment?

Respondent: Oh, I don't know. I think it... you know, how much physical activity you get in – like for me, and working fulltime, that certainly cuts down the time that you've got to do things, because when I wasn't working – I didn't work for 16 years, but I was still involved with voluntary work around the school, relief teacher aid, canteen. But then other times I would be helping in the orchard, running around especially during the picking time, so that was a physically active time.

But our circumstances have changed, and I'm working, so that's... yeah, so it's just the way it is at the moment. So I'm just... as I said to you before, we'll just capture what I'm doing at the moment. [Laughs].

Interviewer: Yes. Yes. And that's a recognition that, as you're saying, our life changes depending on what part of our life it is, and what's going on. But always working fulltime, and also travelling a distance, makes it... doesn't give you that much time, and with the family as well, especially with three girls at their stage in school.

Respondent: Yes. Well, of course things change when you have children, but when I was first married – well, single and then first married, like I said I played hockey, and I use to travel – we use to travel up from [town] to [city] on Saturdays, and we didn't mind that. But then when we started to build the house, we had that – lost our time to do that, so and my husband use to sail, so he stopped sailing.

Interviewer: Uh-huh. And now what you do is you take your daughter out to play netball.

Respondent: Just your circumstance... yeah, to play netball. There use to be a club down at [town], [town], but that all folded I think just before I came to Tasmania.

Interviewer: Oh, that's a shame.

Respondent: There's a lot of nice ovals down there that it would be good to run around on. And if there was some sort of club, or some sort of activity down there, I would really make an effort. [Laughs].

Interviewer: And that's another thing that's coming out of the study – people are actually saying that too – if there was something actually happening in their own community they'd make that effort, and part of it is just being part of the community, as well as doing something physically active.

Respondent: Yeah. There's a lot of netball, basketball, like the aerobics, the swimming, I think, but no-one's really put their hand up to do hockey. I don't know if there's a women's soccer, because there's some nice ovals down at [town]. Maybe they get too wet in the winter, I don't know.

Interviewer: Yeah, so there are some possibilities for facilities already there.

Respondent: Possibilities, yes. And look, if there was summer hockey – in the summer...

Interviewer: Hmm, when you've got more light and time.

Respondent: When you've got more light, and there's no football, or no soccer, you could perhaps use that facility, the green grass to run around on and have a run.

Interviewer: Yeah, so that would be a good use of facilities that way.

Respondent: Yeah. Yep, if no-one else was using it.

Interviewer: Uh-huh. Thank you very much.

Respondent: Oh, you're welcome.
