# Supplementary material for: A Qualitative Study of Environmental Factors Important for Physical Activity in Rural Adults
Source: PLoS One. 2015 Nov 10;10(11):e0140659. doi: 10.1371/journal.pone.0140659 (PMC4640845; doi:10.1371/journal.pone.0140659)
Supplement: S3 File — (PDF) [file pone.0140659.s003.pdf]

Interviewer: OK so I thought we might start with a bit about talking about the place where you live.

Respondent: Sure.

Interviewer: OK? So now that's [town], so can you tell me a bit what it's like to live around in [town] and your likes and dislikes?

Respondent: I actually really like [town]. I think it's a really friendly town. It's very central to all of the things that I'm involved in and I need to access too.

Interviewer: Yep.

Respondent: So I travel from as far as [city] through to [city], back to [town], so it's quite centrally in that triangulated area.

Interviewer: Are you in [town] central itself…?

Respondent: Yes. Yes. I'm only five minutes walk from in the city, from town…

Interviewer: Oh right. Yeah.

Respondent: Yeah, which is very convenient. I do a lot of walking in the Summer time, but Winter time things go a bit slack 'cause it's too cold [laughs].

Interviewer: Fair enough [laughs].

Respondent: Yeah. And wet.

Interviewer: Yes, yes.

Respondent: So I love [town], I think it's a great little town. It's very handy in as much as you don't pay for parking, which is a thing when you're on a very limited income.

Interviewer: That's a bonus.

Respondent: Yeah, so I really love [town]. I actually had my choice.

Interviewer: Yep.

Respondent: I'm in a housing unit.

Interviewer: Oh right. I was going ask you…

Respondent: Yeah.

Interviewer: … your reason for picking [town].

Respondent: And I had my choice, when you sign up for a housing you actually have a choice and [town] was my first choice, I didn't want to come to [town].

Interviewer: Yep.

Respondent: And I got my pick.

Interviewer: Wow.

Respondent: So I was really, really happy with that. Yeah.

Interviewer: And type of property? You mentioned it was a unit. Was it with a block around it…?

Respondent: Yeah. I'm in a one bedroom, in a block of 30.

Interviewer: Oh, OK.

Respondent: So it's very small. I actually moved from [town] down to here in 2004 and I came [laughs] out of a three bedroom house into a little one bedroom unit and I had to cram all my stuff into this little tiny unit. And it's not ideal, space-wise, but all the other factors far outweigh that minor inconvenience, so yeah.

Interviewer: Fair enough. And what's the length of time you've lived here?

Respondent: In [town] would be now seven years, going on to seven years.

Interviewer: So you're quite settled.

Respondent: Yeah, yep.

Interviewer: And so the distance you have from your town centre?

Respondent: Yeah, five minutes walk.

Interviewer: OK. Yeah. So a lot of the research that has been done is really in urban areas and people often refer to their local neighbourhood and I was interested in understanding what the term 'neighbourhood' means to women who live in rural areas, whether it's relevant.

Respondent: Yep.

Interviewer: So what does the word 'neighbourhood' mean to you?

Respondent: I guess general locality. A close knit group of community-minded people is also another definition I'd give it.

Interviewer: Does that have a distance like if you…

Respondent: Well I think so. I think it does, I mean…

Interviewer: What sort of distance would it be?

Respondent: I don't know. I guess it'd be about a kilometre radius.

Interviewer: A kilometre radius…?

Respondent: Yeah, roughly.

Interviewer: OK.

Respondent: Roughly. Although I quite frequently have this talk with my grandfather about what neighbourhood is and how he misses his close-knit neighbourhood and I don't think we have the same type of close-knit neighbourhoods we do now…

Interviewer: No, that he would have had.

Respondent: …that he would have had, yeah.

Interviewer: Yeah.

Respondent: And I have to admit I'm shocking when it comes to staying in contact with my neighbours.

Interviewer: Yeah.

Respondent: Like I talk to them when I see them, but I don't go and have coffee with them. I mean we're on good terms, but we're not certainly in each other's pockets. And I tend to like keeping my distance 'cause I'm quite busy too.

Interviewer: Well exactly. It often reflects the busyness of people's lives.

Respondent: That's right. Yep.

Interviewer: And what about word 'community', what does that mean to you?

Respondent: I think that's probably a little bit of a more close-knit word. A community I guess is a group of like-minded people who want the same things, who live close by, who work together.

Interviewer: Yep. Have a…

Respondent: Have a common link.

Interviewer: Yep, OK. Yep, yes. OK. So in that sense it's different from neighbourhood. It's more the linking between people and there's a commonality.

Respondent: Yeah, yep. I think neighbourhood's more to do with location, whereas community's more to do I think with relationships.

Interviewer: Very nicely put. Yes, yeah, yep. And can that… is there a distance around community for you?

Respondent: No, I don't think so.

Interviewer: Yep.

Respondent: Not in the new modern age that we live in. We're hooked up to the Internet and I can Skype my friends who are overseas, and they're a part of my community.

Interviewer: Yep.

Respondent: My mum's in [state], she's a part of my community and we are on the Internet and Skyping and talking quite frequently, so I don't think community really is in this modern age linked to…

Interviewer: Distance. More of a…

Respondent: Communication.

Interviewer: Communication.

Respondent: Yep.

Interviewer: The means of communication.

Respondent: Yep.

Interviewer: Fair enough. I want to talk a bit about physical activity and what it means to you and that's a very broad term. Each individual has their own meaning to it.

Respondent: Yep.

Interviewer: So what does the term 'physical activity' mean for you?

Respondent: I guess first thing that comes to my mind is thirty minutes a day. It's a challenge for me, this one, because it's something I struggle with. So physical activity I guess for me would be just being active.

Interviewer: Just being active in your life?

Respondent: Just being active in your life, yeah. Doesn't necessarily mean going out for a jog, or going out for a walk. I'm involved in local theatre, so I do activities with that. So yeah.

Interviewer: Yeah. The women we've been talking with have all said that it's about activity in your day to day life, it's not about specific… I like once a week to go to the gym… yep.

Respondent: Yeah. I mean gardening, I enjoy gardening as well, so that's an activity…

Interviewer: Absolutely.

Respondent: … that gets you physically working.

Interviewer: Absolutely.

Respondent: Yeah.

Interviewer: So thinking about that, do you consider yourself to be physically active?

Respondent: No. No, I don't.

Interviewer: Why's that?

Respondent: Because I have actually tried to set myself some goals to become more physically active and I'm not achieving them. Like for example at the moment one of my goals is try and get up half an hour earlier in the morning and take the dog for a walk, whether it's just up and down the street and that's a ten minute walk, or a bit more, but I just haven't been able… and I've been trying to do that for the last month.

Interviewer: And that's every morning?

Respondent: That's every morning. And I've not even done it once.

Interviewer: Well it's getting darker and darker.

Respondent: Colder and colder. Yes and it's… 'cause I'm not actually… it's the middle of study week at the moment…

Interviewer: Yeah.

Respondent: I don't have to go and be somewhere at a specific time at nine o'clock in the morning for a lecture or anything like that. So tempting just to roll over and hit the snooze button and go back to sleep.

Interviewer: Absolutely. And where does physical activity fit in your life? Is it of no interest, of interest, or high priority?

Respondent: It's a priority, but I guess through actions it's not a priority. I'm not giving it a priority. I'd like it to be, but in reality it's just not happening. So yeah.

Interviewer: Thank you for being honest about it. It's really important to talk about if you're feeling like you're not as physically active as you want to be, it's really useful to talk to you about it.

Respondent: Yeah.

Interviewer: Now… and as I said there's no right or wrong.

Respondent: Yep.

Interviewer: Now in our research we think about physical activity as any activity that lasts for at least ten minutes. That's the healthy benefit factor.

Respondent: Yep.

Interviewer: You get a rise of heart rate or you get warmer, especially once the cold…

Respondent: Yeah.

Interviewer: And you know you might breathe a little heavier.

Respondent: Yep.

Interviewer: So what I'm going to do is just ask you to think about this type of physical activity during the past two weeks and just go over parts of your life, mainly so we don't want to miss things, but life isn't quite neatly in parts like that, so that's fine. Now are you working at all at present?

Respondent: No. No.

Interviewer: OK. That's fine. In the past two weeks have you walked or cycled for at least ten minutes at a time to get to or from places?

Respondent: I've walked yes, but not cycled.

Interviewer: Do you cycle at all?

Respondent: No.

Interviewer: No, fine. So we'll focus on walking. Can you tell me what you did?

Respondent: I was in [city] and I decided to park my car, 'cause I donate plasma once a fortnight, I decided to park my car at the Red Cross. I was there an hour or so early, and I walked into town and did some shopping and had some lunch before walking back to the Red Cross centre to donate plasma. And that probably took me 15 minutes each way.

Interviewer: Yep. OK. And what intensity would that exercise be, steady, moderate or vigorous?

Respondent: Moderate.

Interviewer: OK. And how often would you have done that? In the last two weeks...

Respondent: In the last two weeks? Once.

Interviewer: And when did you do that? Was it during the day?

Respondent: Yes.

Interviewer: 'Cause that's when you had the appointment. OK. Right. And why you did it there is because you had an appointment and some time, so…

Respondent: Yep.

Interviewer: And is that what you usually do, or is it a one-off?

Respondent: No, I don't normally do it that way. It just happened to work out that I had some extra time to kill and I didn't want to go through the process of having to park my car, pay for parking, and then do what I had to do, get back in time to not get a parking fine and then drive back over to the Red Cross, and I thought well no, I'll actually pull my finger out, so to speak, and park the car there and walk into town. And that way try and meet this little goal I have of doing some walking.

Interviewer: And so you did.

Respondent: So I did, yeah.

Interviewer: So does this change depend on the season or the time of year?

Respondent: Yes, absolutely.

Interviewer: Yep. Easier around Summer?

Respondent: Absolutely. Yep.

Interviewer: So as it gets colder…

Respondent: Yeah, I tend to hibernate.

Interviewer: Which is a natural thing of… you can feel the season pulling you in [laughs]. OK. Time to replenish.

Respondent: Yes [laughs].

Interviewer: In the past two weeks have you been active around the house, or yard? So inside or outside at home.

Respondent: Oh, just doing the housework.

Interviewer: Yep. And what intensity would you call that activity? Steady, moderate or vigorous?

Respondent: Steady.

Interviewer: Yep. And for how long?

Respondent: A good couple of hours just to the floors and a bit of dusting, vacuuming. Yeah.

Interviewer: Yep, it always takes time, doesn't it [laughter]?

Respondent: Even in a tiny little one bedroom unit.

Interviewer: [Laughs]. And how often did you do it?

Respondent: Over the fortnight, I probably only do my housework once a fortnight.

Interviewer: That sounds very wise.

Respondent: It's pretty simple when [laughter] you live on your own. It does stay pretty clean.

Interviewer: When would you have done it?

Respondent: Crikey.

Interviewer: Time of the day or…

Respondent: Middle of the day.

Interviewer: Middle of the day? During the week?

Respondent: Yes.

Interviewer: Yep.

Respondent: No, hang on. I did it on Saturday. No, I did it on a Saturday.

Interviewer: Oh right. So weekend.

Respondent: Yeah.

Interviewer: And who with?

Respondent: Just myself.

Interviewer: And is that what you usually do, or is it one-off?

Respondent: Yes.

Interviewer: And does it change depending on the season or the time of the year?

Respondent: Not necessarily, no, I'd have to say. Being inside I don't think it's weather dependent.

Interviewer: [Laughs]. And if you'd wanted to, is there anything that would have helped you be more active around the house inside or outside?

Respondent: If I wanted to. If I was motivated. Yes, I guess…

Interviewer: Actually we were talking about gardening earlier.

Respondent: Yeah, but I haven't had the chance to actually get out and get into it.

Interviewer: Yep, yep.

Respondent: I mean there's been other factors with the gardening thing at home. When I first moved into the units I was actually looking after quite a large garden bed that backed up to my side fence. But the contractors came through, new contractors came through and they took over responsibility for it and I was told I wasn't allowed to look after it anymore, so really I'm only responsible now for what's in my little back courtyard.

Interviewer: OK.

Respondent: So there's not really much to do. I've got a tiny little patch of grass for the dog that I mow, and that only takes half an hour. A couple of pot plants which I water, a little herb bed which I water. That's about it.

Interviewer: OK. And likewise with walking, if you wanted to be more active, is there anything that would have helped you do more walking for instance?

Respondent: Anything that would have helped me to do more walking? Probably somebody who would keep me accountable.

Interviewer: So maybe someone to walk with…

Respondent: A walking partner maybe. Yeah. And the same again with being inside, I have an aerobics video, a DVD, and it's sitting there and it's available to me, I'm just lacking the motivation to actually do it. And I think that's the big thing. I know that's the big thing that means I lack motivation.

Interviewer: And have you been physically active for at least ten minutes during your leisure of spare time?

Respondent: Not recently, no.

Interviewer: OK. And you've talked about the walking which… yeah. OK. Now… if not, why not?

Respondent: Well again, lack of motivation. And secondly I've just been so flat out…

Interviewer: Yeah, with study.

Respondent: … with studying at the moment.

Interviewer: Yep, yeah. And that takes priority.

Respondent: Absolutely.

Interviewer: And it tends to take over.

Respondent: It has done this year.

Interviewer: And often you… I know when I studied you didn't… because it became such a part of your life you didn't realise how much it was…

Respondent: … getting in the way of other stuff.

Interviewer: … taking over. Yes.

Respondent: Yeah. I actually went out with a girlfriend on Saturday night. We went and had dinner and watched a movie and it was the first time I'd actually… the semester it was the first time I'd actually gone out and just done something like that. And I thought wow, this is terrible. Life's… the study is really just taking over.

Interviewer: How long have you been studying for?

Respondent: Well this is my first year in uni, but I studied [course] last year at [university], so… and the year before I tried it by distance and it didn't work.

Interviewer: You have been busy.

Respondent: So yeah, I'm into my second year of full-time study.

Interviewer: That's a big load. OK, so I want to talk a bit about physical environments now. We've talked a bit about where you live and physical activity. And so physical activity's about where you live and play and when I talk about physical environment I mean all the physical things around you. Anything from…

Respondent: Open space…

Interviewer: … open space, parks, trees, to shops and recreation facilities, houses, roads, all those things. And some of these things are things that people in urban areas have said may be important to their physical activity. So we're interested in finding out if it's relevant for you as well. So can you tell me a bit about how easy or difficult it is to be active in your neighbourhood community?

Respondent: I guess it's pretty easy. There's plenty of footpaths.

Interviewer: Yep.

Respondent: I'm not that far from the walking track down at the beach. If I wanted to get there I could quite easily. Generally in my neighbourhood area, it's usually pretty well footpathed. So access to flat space, flat area is good.

Interviewer: Yes. That's really important rather than really steep hills. So is there a walking track to the beach or a walking track along the beach?

Respondent: There's a walking track along the beach.

Interviewer: Oh, OK.

Respondent: I can't remember exactly whereabouts it starts and ends. They have the RSPCA Million Paws walk there recently.

Interviewer: Yep, yep. OK.

Respondent: Yeah. It's actually quite a nice walking track too.

Interviewer: OK. So it's not the road, it's…

Respondent: No. It's a dedicated walking track.

Interviewer: Lovely. OK.

Respondent: And they've also built one through to [x] Beach recently as well.

Interviewer: Right.

Respondent: Yeah, which is an extension of that and I know that the one that they're thinking of doing right the way along the [x] coast is going to incorporate those that are currently there.

Interviewer: That would be fantastic.

Respondent: So yeah, it'll be really good.

Interviewer: Yeah. So you can just enjoy the coast rather than the roads and the traffic.

Respondent: Yeah. Yep, yep.

Interviewer: That's good. So if I were to ask you about places to be active in your area in terms of availability and accessibility first of all, what sorts of things would you tell me about? We've just been talking about a few. Any other things that are available and accessible in your local community?

Respondent: There are a couple of gyms in the local area. And I have thought about popping in to see what they're like and what they charge, but again time's been a factor. There are a couple of Heart Foundation walking groups…

Interviewer: Oh right.

Respondent: … that operate in [town]. But the timing for those groups doesn’t suit me. I’ve looked at those. What other things? They’re the only things that really come to mind at the moment.

Interviewer: Open spaces, parks?

Respondent: There’s no real lack for that in [town]. There’s quite a large… like I said where that walking track is down by the beach. That’s quite a large open space, very accessible.

Interviewer: And I guess you’d have a range of sporting clubs as well in [town]?

Respondent: Yeah, yep. Yep.

Interviewer: So, OK, so there is a range of things that are available. Are they actually accessible as well? Are they easy to access or do they have cost to them or hours of opening?

Respondent: That’s a good question. I imagine with the gyms there’s a cost. I don’t know what the clubs are or what is involved with those…

Interviewer: You’re more likely to be walking anyway, which is something that’s successful to you...

Respondent: That’s right.

Interviewer: Fine.

Respondent: It's something that's very achievable for me. And once the motivation factor's fixed [laughs], something I can probably stick to in the right circumstances.

Interviewer: Does having places to be active influence whether you're active or not? Like having, you were saying about being able to walk around and have footpaths and walking tracks…

Respondent: Well I definitely less inclined to actually be active if those things weren't there. Definitely.

Interviewer: And if you wanted to be more active are there things or places that would help if they were more available or more accessible?

Respondent: Yes, I think so. What those things are, that's… good question. I really don't know.

Interviewer: 'Cause I know we get used to how our community is.

Respondent: But kind of being a bit visionary about what could be there… I guess I've never really thought about it. Yeah, I haven't really thought about it.

Interviewer: That's fine 'cause it sounds like [town], there's quite a bit that's available anyway. OK. Now I wanted to talk to you about destinations. If I asked you about places you could walk to and from your home, what sorts of things would you tell me about?

Respondent: Walk to the shop, do some groceries. Walk around to visit a friend, I guess. I do that. My church has a community centre up the road, I walk there, sometimes. Yeah, actual destinations. There's not many things in my local area that I would actually walk to at the moment.

Interviewer: Those are certainly things you've mentioned though.

Respondent: So I've got a choice of actually supermarkets. There's a little railway IGA which is not that far away from home. And then if I walk further into town you go to either Coles or Woolworths, which I've done in the past.

Interviewer: So does having destinations influence whether you're active or not?

Respondent: I really don't think so because if I was to make it my focus just to be physically active I don't think it would matter whether I was going somewhere specifically or not. Like in the past when I have been physically active just to take the dog for a walk around the block. The destination is to get back home after having walked a certain distance. So I don't think it is actual… it's really important.

Interviewer: Though is it one part of it? 'Cause you were saying…

Respondent: But it is one part, yeah.

Interviewer: … you get to walk to the shop, and you've got a choice of making it a longer walk or you get to walk to a friend's, so…

Respondent: Yeah.

Interviewer: So in that sense is it helpful?

Respondent: No, I don't think so.

Interviewer: OK, fine.

Respondent: Not for me.

Interviewer: It's just handy.

Respondent: Yeah.

Interviewer: Yeah, OK. And if you wanted to be more active would it help if there were more destinations within easy walking distance?

Respondent: Yeah, I think so.

Interviewer: You think so?

Respondent: Yeah, I think so. Yeah, if there were more I guess facilities or places to go. Like if I decided… if there was say a cinema here and I decided I wanted to go and see a movie and it was in town, well then I would more than likely walk there rather than drive the car there.

Interviewer: OK.

Respondent: As an example.

Interviewer: Yep. Yeah, that's a good example.

Respondent: Yeah.

Interviewer: OK. Now neighbourhood design, if I were to ask you about the built or man-made features, the non-natural features of your neighbourhood, what sorts of things would you tell me about?

Respondent: Street lighting is an issue.

Interviewer: Oh right.

Respondent: Just about every street that I've walked down in the evening, the lighting is almost always on the other side of the street to the footpath.

Interviewer: Oh, OK. And does that influence your visibility?

Respondent: Visibility, safety more than anything else, I think that's quite a turn off. Yeah, and I've put in… I'm always very physically conscious of safety I guess. So I'd never, ever go anywhere without my phone or… that's if I'm on my own. When I've got the dog it's totally different because she's a bit of a bruiser, but yes, if I was to walk on my own, looking around…

Interviewer: Yeah. That's a really important observation. And what about, in saying you've got footpaths, but what about are the streets connected so you can actually walk where you need to go rather than… some places here there's just one main road and that's it.

Respondent: Yeah, I think they're pretty well connected.

Interviewer: Yep. OK. And they're fairly walkable?

Respondent: Yeah, pretty much. Yep.

Interviewer: Do the built features of your area influence whether you're physically active or not?

Respondent: Well I think yes, I'd have to say yes. Particularly with the lighting issue.

Interviewer: A night?

Respondent: Yeah. And some of the footpaths I have to say are pretty shonky here in [town], particularly like [x] Street's just up the road from where I am, and it's got a lot of old trees lining the nature strip, and they've raised the footpaths. Like I know a couple of years ago the council did try and address the issue, but they're still quite a number of footpaths around that have buckled or are in need of repair. And the other thing, and this is not relating to me, but the other thing I have noticed here in [town] is a lot of older people who rely on their motorised scooters to get around are using the road because the curbs aren't built for their motorised scooters.

Interviewer: So they can't get up…

Respondent: So they can't get up and down on the footpaths, from the road to the footpath and vice versa. And that's an issue, a safety issue for them. Yeah. But obviously for me walking that's not. So yep.

Interviewer: So that would be the same issue for prams and things?

Respondent: Yeah, mums with prams, yep.

Interviewer: OK. And if you'd wanted to be more active are there any built features in your area that would help? It sounds like the lighting on the right side of the footpath…

Respondent: … would be good. I really don't know. Never really thought of that.

Interviewer: You've already been talking about the curbs and some of the footpaths need some work, so I guess if there's any other comments you want to make.

Respondent: No. Nothing that really comes to mind.

Interviewer: OK. Aesthetics, is the other side of it. If I was going to ask you about the aesthetic qualities, the actual natural features of your environment, or the attractiveness of your area, what sorts of things would you tell me about?

Respondent: Yeah, it's not bad. It's not bad. There's not many eye-sores I guess from a visual perspective.

Interviewer: Yeah, so no rubbish, litter, graffiti?

Respondent: I was just going to get to that [laughter].

Interviewer: Oh right.

Respondent: Just up the street from where I am there are more housing units and there's a residence that's next door to these units that constantly the fence gets sprayed with graffiti. Out the front of my block of units there's a garden area that's next to the footpath and there's a lot of litter that accumulates in there I notice, so I'm out there every now and again with a bag picking up stuff, but it just keeps turning up. But generally speaking it's not too bad. I grew up in an area in [state] that was terrible, it was just dreadful. There was litter and vandalism everywhere and graffiti everywhere. That was pretty bad, but this is… [town] pretty good.

Interviewer: OK. Do you think the local environment is aesthetically pleasing?

Respondent: Generally, yes.

Interviewer: Around where you live?

Respondent: Generally, yes. Like the vandalism, the graffiti is a bit off-putting. I noticed it when it started to occur a couple of years ago, it actually started when some residents moved in, so… but prior to that it wasn't really a problem.

Interviewer: OK. So do you have trees and parks?

Respondent: Yeah, yep. Yep. Yep. Yep.

Interviewer: As well as the beach obviously...

Respondent: As well as the beach, which is nice.

Interviewer: How far are you from the beach?

Respondent: Probably a ten, 15 minute walk. Very nice.

Interviewer: Very nice.

Respondent: It is very nice, yeah. And there's a patrolled beach down there as well, so in Summer time you can go down there and be quite safe, it is safe. Yeah.

Interviewer: OK. And do the aesthetics of your area influence whether you're active or not?

Respondent: Yeah, I think they do. Like when the rubbish and the graffiti is very prominent I'm less inclined I think to go out. I don't want to see that. If I can turn a blind eye and not look at it and just appreciate the trees

and beautiful weather and that I think I'm more inclined to go out and enjoy it.

Interviewer: OK. OK. And if you wanted to be more active are there features that might help if they were more aesthetically pleasing?

Respondent: Yes. Yes, I think so.

Interviewer: And what would that be?

Respondent: Well, just a clean area…

Interviewer: The rubbish, graffiti…

Respondent: … graffiti. The occasional stray shopping trolley [laughter]. Yeah, yeah.

Interviewer: OK. There's only one more area to talk about and that's safety. If I were to ask you about safety in your area, what sorts of things would you tell me about?

Respondent: Oh, sometimes I haven't felt safe, but I think most of that goes back to the lighting issues. Yeah, I think that's pretty much the only downside to safety.

Interviewer: And you've actually said… well I'll ask you the question, does personal safety influence whether you're physically active or not?

Respondent: Yes.

Interviewer: In particular at night.

Respondent: Yep.

Interviewer: And does road safety influence whether you're physically active or not?

Respondent: In what terms?

Interviewer: So is road safety an issue, the traffic, roads?

Respondent: No, not if I'm being observant.

Interviewer: The main thing is you do have somewhere to walk off the road that's OK.

Respondent: Yes. Yep.

Interviewer: OK. And if you wanted to be more active are there any personal or road safety issues that, if addressed, might help you be more active?

Respondent: No, don't think so.

Interviewer: Other than the lighting.

Respondent: Other than the lighting, yes.

Interviewer: OK. Well before we finish up, is there anything else that you'd like to tell me about, where you live, your physical activity or the environment that you live in that we haven't covered today?

Respondent: No, I think that's it.

Interviewer: Great.

Respondent: I think that’s it.

Interviewer: Great.

Respondent: Yeah.

Interviewer: Thank you very much.

Respondent: No worries, my pleasure.

Interviewer: OK, so I thought we'd start by talking a bit about where you actually live?

Respondent: Right.

Interviewer: OK. So can you tell me what it's like to live where you do and your likes and dislikes?

Respondent: I love living where I do, at this stage in my life.

Interviewer: Now you're in [town]?

Respondent: Just out of [town].

Interviewer: Just out, OK.

Respondent: So we cannot walk anywhere to any facility, well you could walk to the school when the kids were at school, that was it, we're sort of up the [x] Range, up towards the [x] Range, too far out to really walk into town, so that's fine when you've got a car outside to hop into and pop to the shops, but the long term plan for retirement is to move down into [town] because of those things that you can't get to the shop, the doctors, the chemist that sort of thing, but for this stage of our life, and it was a superb place to bring the kids up, just excellent, I wouldn't have liked it myself as a teenager but my boys love it.

Interviewer: So tell me, you've mentioned you're a little bit out of [town], what sort of distance?

Respondent: Well it's only two kilometres, which sounds a bit pathetic, but it's on a…

Interviewer: … no, no, I've been talking to people in [town] I understand.

Respondent: So you can get into [town] fine.

Interviewer: But it's getting back up, so you're saying that you're closer to the school.

Respondent: We're just a bit further up than the school, about another kilometre up.

Interviewer: I was talking to someone who was saying where the school was in relation to, she was actually on the other side so she was saying well it's too far to walk to school because you come down and then up.

Respondent: Yes, those hills for the kids, they can't walk to school, my kids could always walk to school but they couldn't walk anywhere else, I always knew where they were.

Interviewer: So what's, where you're living is it a house with house block or a bit of land?

Respondent: What would they call them, bush block, two acres.

Interviewer: OK very nice, very compact but easy to deal with and you've got a bit of land as well.

Respondent: We manage OK at the moment.

Interviewer: Keeps you busy?

Respondent: Yes, there's always something to do.

Interviewer: And how long have you lived there?

Respondent: Eighteen years last month.

Interviewer: And your reasons for living there, why did you pick there?

Respondent: We arrived, my husband's [state], we met in [city] and when we had our first child we didn't want to stay in [city] so we were moving away from the city so it was either going back up [state] or moving to [country], came over to [country] for a visit, stayed with the in-laws in [city] and hated it, we came over to [state] for a visit and oh this is much better, and the next thing I knew I was living here and I don't remember that bit in between, I know I was pregnant, but for some reason we decided we'd come to [state]. So his mum had a rental house in [town] so we stayed there to start with while we looked around, now I thought we'd be in [city] because I thought it was just too much of a quantum leap from [city], but then [husband] was offered a job while we were in [town] so we stayed there and looked around for a house in the area, and I wanted, we wanted a place with a bit of land and just something that you couldn't get at all in [country], so that's why we chose where we were, didn't want to be a house on a street, just a bit different, it's very different.

Interviewer: Very different from [city].

Respondent: Yes.

Interviewer: Now in research with women most of it's been done in urban areas, people often refer to their local neighbourhood, and I'm interested in understanding what the term neighbourhood means to women who live in rural areas and whether it's relevant or not, so what does the word neighbourhood mean to you?

Respondent: I suppose I'd have to take neighbourhood to mean [town] really, but I just can't talk about my little area of neighbours because we haven't got that sort of idea, so I'd say [town] is my neighbourhood.

Interviewer: So you haven't really got that many people around you?

Respondent: No, it's not like when you live on the street when you talk about your bit of the street with the people who live next door to you and the people who live opposite, like in a city.

Interviewer: That's a city concept, OK so if I asked you to put a distance around neighbourhood for you, how big would it be, so it's a couple of kilometres into [town] and then?

Respondent: I would say [town] and the surroundings of a couple of kilometres all the way around is our local.

Interviewer: So it's getting to [town] and [town] itself, so what would be the distance of that overall if you had to think of how long a distance that would be?

Respondent: Five kilometres maybe.

Interviewer: OK, now what about the word community, if I asked you about that what does that mean to you?

Respondent: [town].

Interviewer: So it's a similar distance to neighbourhood?

Respondent: Yes in [town] yes, in other places I lived I might have thought of that as slightly different, but [town] I would.

Interviewer: Does it have a slightly different meaning though in terms of neighbourhood or is it the same?

Respondent: Again in [town] it's the same.

Interviewer: From what I gather from what women have said it's a rather lovely community.

Respondent: Yes, I think especially so when you've been involved in the school, it gets you to meet so many people around that you wouldn't know otherwise.

Interviewer: OK, now about physical activity, there's a saying, it's very broad and everybody has a different understanding of it, and different times of your life is different too, so what does the term physical activity mean for you?

Respondent: Well I suppose it's any activity that you do at all, not necessarily just doing it for sport purposes, health purposes, anything, it's anything, just your general movement around.

Interviewer: What you do in your life, your day to day?

Respondent: Yes, I know I'm very aware because I wear a pedometer all the time, and just how many steps you can get, just doing your housework, which is just as much activity as it is if you'd gone out for a hike across the moors.

Interviewer: Absolutely.

Respondent: Just shopping, how many steps you get in shopping, wonderful activity.

Interviewer: Amazing, so given that do you consider yourself to be physically active?

Respondent: No, I'm reluctantly active, I'm active because I know I need to be for my health purposes, not because I particularly enjoy or wish to be.

Interviewer: OK, so you do consider yourself active?

Respondent: Yes, I suppose so.

Interviewer: And why do you consider yourself active?

Respondent: Because I try to be.

Interviewer: And where does physical activity fit into your life, is it of no priority, high priority?

Respondent: It's not a high priority, I can find better things to do, so if somebody phoned up and said do you want to come for a coffee just as I was about to do a gym session I'd be off for a coffee not the gym.

Interviewer: I think I'd be the same.

Respondent: It's something that I force myself to do.

Interviewer: Now in research when we think about physical activity, we think about any activity that lasts more than ten minutes, that's the health benefit definition, and it causes your body to either work harder than normal,

so your heart rate might be up, you might be feeling a bit warmer, you might breathe a little heavier, a bit of huffing and puffing, so thinking about this type of physical activity over the last two weeks, I'll just ask you about different parts of your life, not to segment your life, but just so that we pick up on all the parts of your life really, so are you working at the present moment?

Respondent: No.

Interviewer: So in the past two weeks have you walked or cycled for at least ten minutes of the time to get to or from places?

Respondent: Specifically to get to and from places, no, I drive wherever I have to go to as I work and walk to get, except last week I was in [city] which is slightly different, but normally when I'm at home I have to drive wherever I go to.

Interviewer: OK, so if you wanted to, is there anything that would help you to be able to walk to and from places or cycle to and from places when you need to?

Respondent: Specifically to go and do something rather than for exercise sake?

Interviewer: That's right, to get to and from places.

Respondent: To get to and from places, flatten the [x] Range, no at the moment I just couldn't walk anyway.

Interviewer: Yes, and hence what you were saying about maybe the next stage you're thinking of moving down to [town] so that would be quite different.

Respondent: I'd be able to walk to the shop, I wouldn't bother driving along to the shop or the post office or such things.

Interviewer: It's very common when women are talking about is you have to get in your car to go to places before you can get the walk or cycle or whatever.

Respondent: Yes, when I'm down at my son's in [city] I do a lot more walking around because it's a lot easier to do so.

Interviewer: So in the past two weeks have you been active around the house or yard, either at home inside or outside?

Respondent: Yes, oh I'm an inside person I'm not much of an outside person.

Interviewer: Can you tell me what you've done?

Respondent: Just general chores.

Interviewer: Absolutely, they don't go away do they. So if you had to describe the intensity of the chores, housework and various different other things, how would you describe them, steady or moderate, vigorous?

Respondent: Just steady.

Interviewer: And the duration, how long would you have done them for?

Respondent: It varies day to day when I actually have a house working day it takes me roughly about six hours to do the whole house, and then probably two hours other days with different bits and bobs.

Interviewer: And how often would you have done that in the last two weeks?

Respondent: Again the last two weeks I've spent a week in [city], an ordinary two weeks when I'm not away for a week, I'd have one day a week as a real cleaning the house session, and then every other day I'd be spending a couple of hours.

Interviewer: And when would you have done that, during the weekdays?

Respondent: Mostly during the week so I can do more interesting things at the weekend.

Interviewer: And during the day or?

Respondent: Yes during the day.

Interviewer: Who with, alone or with others?

Respondent: Just alone.

Interviewer: Is this what you usually do?

Respondent: Yeah.

Interviewer: Does it change during the season or the time of the year?

Respondent: It changes when the boys are at home from uni, which they are at the moment, they came home on Friday.

Interviewer: So they come home at the end of each semester do they?

Respondent: Study period at the moment and exams start at the end of this week, so it's a bit stressful.

Interviewer: So they're down at?

Respondent: No it's not even that easy, one's at [city] and one's at [city].

Interviewer: OK.

Respondent: Don't make life easy for yourself, so I do a lot of driving around because neither of the boys drive, they just decided they didn't want to, I make life too easy for them, I should stop driving them around and they might think about learning.

Interviewer: That's interesting, particularly as you were saying where you live, you actually have to get in the car to, OK. Now if you'd wanted to, is there anything that would have helped you be more active around the home? It sounds like you're pretty active.

Respondent: I can't think about the home, I can think of things outside, you know doing for leisure activities.

Interviewer: OK we'll get onto that, that's actually the next bit, have you been physically active for at least ten minutes during your leisure or spare time?

Respondent: Yes.

Interviewer: Can you tell me about what you did?

Respondent: We have a gym in the house, so I try to use the gym most days, I don't succeed, every weekend day because I've got a friend who comes up and forces me into it, so that's a definite, and then I try.

Interviewer: That helps, having somebody else to do it with.

Respondent: And then during the week I try to say at least two or three times, but I’m very good at finding excuses.

Interviewer: There’s always something else to do (laughter).

Respondent: Yes, and at the moment really the gym is about all I’m doing, I haven’t been outside for a walk for quite some time.

Interviewer: Is that something you would have done?

Respondent: Yeah but I had a bit of a problem with my knees recently, that’s why I’m doing this other study with the [research group], the arthritis in my knees, and I can walk flat fine, but up the hills.

Interviewer: But you’re not flat.

Respondent: No, so I mean I can drive somewhere, stop and go for a walk there, but really you have to stop and think about it.

Interviewer: OK, so the activity that you have been doing how would you describe the intensity, would it be steady, moderate, vigorous?

Respondent: Well I’ve had to reduce my aerobic activity because everything aerobic wise is hurting my knee, so the exercise bike and the cross trainer is just out of the question now, the rower, so the only aerobic activity I can do is my treadmill on the flat, I can’t use the incline on it, so I’ll be on that for about twenty minutes and then the knees start hurting, then I do the rest of the time doing muscle work, which has changed, I used to do more aerobic and just a little bit of core work.

Interviewer: So both of those activities would be fairly moderate in intensity?

Respondent: Yes.

Interviewer: And how long would you do your exercise workout for?

Respondent: Forty-five to sixty minutes.

Interviewer: And you mentioned how often, once definitely on the weekend.

Respondent: Definitely both days on the weekend then probably three times during the week, so four to five times during the week.

Interviewer: So what time of the day would you tend to do that?

Respondent: Just after breakfast.

Interviewer: And why do you do it at that time?

Respondent: That's what time [friend] comes over to do it, I also, I like to get it over and done with, because it's done then.

Interviewer: So you can start the day.

Respondent: The only thing is if you do your exercise first you think I've done my exercise today I can just sit here and watch this program and you have to still realise you've got to stay active the rest of the day not just veg out, so that's why I wear a pedometer as well, just to keep an eye on.

Interviewer: OK, now you're doing that at home, and why do you do it there?

Respondent: It's just more convenient, as my husband said we spent all that money getting the gym in there we've got to use it, it's mostly convenience, because I know, occasionally I've joined gyms, and you spend more time driving there and getting changed and things and driving back again, and it's taken three hours out of your day for forty-five minutes exercise, especially when the children were little and you had to organise childcare and things, it was very inconvenient.

Interviewer: That's a very good point. Is this what you usually do?

Respondent: Recently yeah.

Interviewer: Does it change during the season?

Respondent: In the summer I'd probably get out for walks more.

Interviewer: And if you'd wanted to, is there anything that would have helped you be more active in your leisure or spare time, you've actually mentioned one thing on the weekend that actually helps, to have somebody.

Respondent: Yeah she had a year off last year, she was coming up more often during the week and I wasn't getting away with anything then, I suppose having a walking group or something like that would definitely.

Interviewer: The other thing you mentioned was where to walk, if you had something a bit flatter.

Respondent: A walking track or something like that would be lovely, I'd love to cycle, but I've never been able to where we are.

Interviewer: It's too hilly, and you're on a main road aren't you?

Respondent: Yeah.

Interviewer: OK, so I want actually go on to talk a bit more about that, your physical environment, so when we talk about your physical environment it's where you work, live and play, that sort of environment, and it could, it means all the physical things around you too roads, trees, houses, shops, traffic, parks, open spaces, beach, those things, and some of these things are things that people in urban areas have said might be important for their physical activity, so that's why we want to find out what, is it relevant for women who live in rural areas as well, so first of all can you tell me a bit about how easy or difficult it is to be physically active in your area?

Respondent: I think it's probably a little bit harder because you can't just access it that easy, you have to go out and find it.

Interviewer: Get in the car and find it.

Respondent: In the car and go to it which sometimes seems a bit daft to drive somewhere to go for a walk, it's negated.

Interviewer: And part of the incline that you live on, but you also live on a main road, so to do with, you'd have to walk on the road to do anything.

Respondent: It's not pleasant.

Interviewer: With traffic?

Respondent: Say another one and a half kilometres to the [x] Range walking tracks and that's nicer, but that's even steeper by the time you get there.

Interviewer: So that's one and a half kilometres further?

Respondent: Further up yeah.

Interviewer: So I'm going to talk a bit about different features of the environment the first one is availability and accessibility, so if I was to ask you about places to be active in your area, what sorts of things would you tell me about?

Respondent: Well I suppose there are near to where I am, there is the big basketball stadium, so there are the facilities there if you were that sort of person into those sort of sports, that's available, and we have a wonderful [x] Range walking track which is fantastic for great bush walks, when your knees are capable of coping with it, there were some lovely walks around there, apart from that everything is in the car and driving off to look for it.

Interviewer: So you do have places to be active in your area, and accessibility, that's more the issue, but you really do have to get in a car to access them, but other things about, you know cost and opening hours, they're not activities that you would do anyway.

Respondent: Not things that are just sort of drop in, you'd have to be in a team sport to use that sort of thing, the only one that I know of in [town] anyway, the basketball stadium, I don't know of any facilities that you could just pay to go in, like say a swimming pool, that sort of thing, I don't know if we've got anything like that.

Interviewer: So it's more an organised sporting.

Respondent: Yeah if you were in a basketball team or squash or something like that.

Interviewer: So just having, or not having places to be active influence whether you're active or not?

Respondent: It makes it a bit more difficult, you have to think more about it probably and try to take more opportunities to maybe change your activities.

Interviewer: And if you wanted to be more active, are there things or places that would help you if they were more available or accessible?

Respondent: I think the thing would be you'd have to make it a priority.

Interviewer: Or if there are things that you may want to do that aren't available locally at the present moment?

Respondent: Not at this moment, in the past I would have loved if we'd had a swimming pool, a year round indoor swimming pool available, especially when we had the kids with us as well, I was quite surprised when I moved to [country] about how few swimming pools there were, I thought they would be all over the place, here they're only available in the summer, there's a few privately owned ones that are available for renting out but you have to know about them, they're not really public accessible and back home, and also when I first came here I noticed there wasn't as many leisure centres, drop in places where you do aerobics and gym and all those sort of things.

Interviewer: So destinations, if I were to ask you about places that you could walk to from your home, what sort of things would you tell me about? Now you've already said.

Respondent: I can walk to the school, the basketball stadium, you could walk to the golf course from our place, but I'm not a golf person, and there's the [x] Range walking tracks, and that's about it.

Interviewer: How far off did you say the [x] Range was?

Respondent: About another kilometre further on until you're in the car park for the base of Mt [x].

Interviewer: Does having destinations actually influence whether you're active or not?

Respondent: I don't think so, I think the activity is because I want to be fit and want to be healthy not because it's there.

Interviewer: It might help but it's not the be all and end all of it. So if you wanted to be more active, would it help if there were more destinations within easy walking distance?

Respondent: Yeah probably.

Interviewer: Now neighbourhood design, if I was to ask you about the built or manmade features, the non natural features of your area, what sorts of things would you tell me about?

Respondent: Do you mean leisure facilities?

Interviewer: Footpaths and lighting and rides and walk ability, those sorts of things?

Respondent: Well they have just improved the footpath along the beach so occasionally I'll walk from one end to the other of the beach because the footpath is there, lighting I wouldn't dream of going walking at night time, you hear about people that get up at six o'clock and go out before the sun's up, and I think how keen are you, but no that's not for me, so I wouldn't go out at night. I can't think of anything really.

Interviewer: That's fine, do the built features of your area influence whether you're physically active or not?

Respondent: Well I thought it would, I thought I'd be walking down the beach much more often, but it didn't really, I don't actually go down and do the walk that much more often before we have the nice footpath on the beach, I thought it would but it didn't.

Interviewer: So if you wanted to be more active, are there any built features in your area that would help?

Respondent: I can't think of any.

Interviewer: Apart from what we've already said, that you have to really get in the car, you don't have a footpath outside?

Respondent: No.

Interviewer: I want to talk about the other side of it which is the natural features.

Respondent: We've got plenty of them.

Interviewer: If I was to ask you about the aesthetic qualities or the attractiveness of your area, what sorts of things would you tell me about?

Respondent: You just have to look out the window it's just absolutely beautiful.

Interviewer: So you've got the coast and the hills and trees?

Respondent: Yeah.

Interviewer: Do you think your local environment is aesthetically pleasing and attractive near your home?

Respondent: Yes.

Interviewer: Do the aesthetics of your area influence whether you're physically active or not?

Respondent: I would say so yes, it's nice to go out when it's nice around.

Interviewer: That's part of the reason why you're living here, and if you wanted to be more active are there features of your area that might help if they were more aesthetically pleasing and attractive.

Respondent: I can't think that it could be particularly more attractive than it is.

Interviewer: One last area is about safety, if I were to ask you about safety in your area, what sorts of things would you tell me about, you've mentioned a few already, traffic being one.

Respondent: Bull ants, jack jumpers, snakes, that's something that concerns me when I'm out walking is that I always have my phone with me because if you were to even just say go over on your ankle or something and

you're up the [x] Range, it might be three days before somebody comes across you, so that is something that I'm very aware of, occasionally I've wandered out of mobile phone reception and I feel very insecure which isn't something that does happen when you're exercising around an urban area, and you do have to just be careful about the wild life and such like as well.

Interviewer: Does personal safety influence whether you're physically active or not?

Respondent: It makes me, I'm aware of it but it doesn't stop me doing it.

Interviewer: The other side of personal safety is [town] a fairly safe community in that sense?

Respondent: Yeah.

Interviewer: Does road safety influence whether you're physically active or not?

Respondent: No, because, I'll still walk on the road and just be careful about it, but it would be nice to have a path to walk on rather than the road, and in one way I walk with my son sometimes and he doesn't like to walk on the road at all, so he always wants to be on the track off the road and that means that it's less flat surface for me to walk on which makes it a bit difficult, so it's getting hard for us to go for a walk together now.

Interviewer: If you wanted to be more active, are there personal or road safety issues that if they are addressed might help you be more active?

Respondent: I think that's a little bit unfeasible to have paths up there, the remotest part of the, so I don't think really.

Interviewer: That’s fine, now just one final question before we finish up, is there anything else that you’d like to tell me today about where you live, your activity or your environment that we haven’t covered today?

Respondent: Not that I can think of no.

Interviewer: That’s great, thank you very much.

Interviewer: So I thought we might start by talking a bit about the place where you live.

Respondent: Yep.

Interviewer: OK? And so what’s it like? Your likes and dislikes about living where you do?

Respondent: I like, I actually quite like being remote, like I didn’t think that because I’ve been from the city, I’m from [city], I just thought oh this is going to be a nightmare moving up there, but my husband is from the farm in [town] so that’s why I’ve moved up there. And we moved back last August, and then I got this job here…

Interviewer: Oh OK.

Respondent: … and I was thinking with the travel is quite hard, and just the convenience is really annoying if you, like last night we were cooking dinner and we didn’t have any basil, well you can’t pop down to the shop, that’s over then, so it’s really annoying.

Interviewer: Yep.

Respondent: And you miss out on things, like if someone wants to do something in town, one of my friends, well I can’t.

Interviewer: You can’t just turn around and go.

Respondent: It’s more of an effort, yeah. You still can do things, but it’s just that little bit further to go, but I love the quiet.

Interviewer: Yes.

Respondent: I absolutely love that side of it.

Interviewer: So you were saying that you live just outside of [town]?

Respondent: Yeah.

Interviewer: How far?

Respondent: It's about a ten minute drive.

Interviewer: OK.

Respondent: On the way to [town].

Interviewer: So it's, what would it be? A few kilometres? About?

Respondent: Yeah, it would probably be, I don't know, it'd probably be, what'd it be ten kilometres? Or more.

Interviewer: Ten or 20?

Respondent: Yeah.

Interviewer: 20? Yep.

Respondent: Probably. Hmm.

Interviewer: Yep, OK. And do you live on the farm?

Respondent: Yep.

Interviewer: Yep, OK so you've got, you're living in a house on a farm. OK.

Respondent: Yep.

Interviewer: And how big's the property?

Respondent: Um 2,500 acres.

Interviewer: Wow. Sheep or cattle country?

Respondent: Sheep.

Interviewer: Right, OK.

Respondent: Sheep and crops.

Interviewer: Yep. And your reasons for living there?

Respondent: [x] is managing the family farm.

Interviewer: Ah huh.

Respondent: His parents have moved up into the [region].

Interviewer: Oh OK.

Respondent: So they don't want to be farming full time anymore, so…

Interviewer: Yep.

Respondent: … yeah, that's why we're there. So he's taking over eventually.

Interviewer: Oh OK.

Respondent: Yeah.

Interviewer: OK. And um, so your nearest township is actually [town] itself.

Respondent: Yeah.

Interviewer: OK. Now I want to talk a bit about in urban areas where most of this research is being done, um people often refer to their local neighbourhood.

Respondent: Yep.

Interviewer: And I'm interested to know, well particularly as you've lived in [city] …

Respondent: Yep.

Interviewer: … whether it's a relevant term for living in a rural area? OK? So so what does the word neighbourhood mean to you?

Respondent: Hmm… to be honest living up there I haven't, I actually don't know many people at all. I know maybe one couple that live in [town] …

Interviewer: Yep.

Respondent: … and no one else, so I really am quite disjointed I suppose from the community, like I'm still very you know, I guess settling in and…

Interviewer: It's very settling in and also with your work you'd be leaving very early…

Respondent: … yeah exactly.

Interviewer: … and getting back quite late.

Respondent: I'm here full time, so I don't really, like one of the other ladies that lives up there, she was saying oh I didn't know anyone until I had a baby because then I was there all the time…

Interviewer: Oh OK.

Respondent: … so that was the difference for her, but I'm, I'm really there on the weekends and I travel sort of a couple of nights a week as well, but…

Interviewer: Oh right.

Respondent: … I guess the word neighbourhood to me has never really been… maybe when I was younger and I lived in [city] and I had like a lot of friends that lived in that neighbourhood…

Interviewer: Yep, yep.

Respondent: … but other than that, that's…

Interviewer: Yeah, the immediate few streets.

Respondent: Yeah, when you saw someone you know, you went for a walk or whatever with them, but living out there…

Interviewer: Yep, yep. So that term's not particularly relevant for you.

Respondent: Not for me.

Interviewer: What about the term community? Has that got any? What would that mean to you?

Respondent: Um, yeah that probably has more…

Interviewer: Is it more relevant?

Respondent: … I'd say yeah for some reason that word to me means a bit more because I think of not just [town] the word community to me is the whole area, so it might be [town] is someone in the community as well.

Interviewer: Yep.

Respondent: Um, and neighbourhood I suppose makes me think of you know, neighbourly friends that kind of thing which we don't have, so the community to me is more a broader term I suppose.

Interviewer: Uh huh.

Respondent: And people help out, like there was a death in [town] …

Interviewer: Yes, I heard about the tragedy. Yes.

Respondent: … last week, and um he was a friend of my husband's and the really amazing thing was living up there that they all went round and helped out to tidy the farm up in time for the funeral.

Interviewer: Oh, right.

Respondent: And it was a real amazing community spirit, whereas we were talking about that and saying I wonder if that would happen, it probably wouldn't happen in the city. You wouldn't just get random people going around, whereas this was people that didn't know people, but everyone's affected when it's in a community.

Interviewer: Yep, yeah.

Respondent: Yeah, that was really nice.

Interviewer: So if if I talked about community having a distance factor around it, how big would you see the community? The local community there?

Respondent: Um, probably as far as I'd say like [town] and [town].

Interviewer: Yep.

Respondent: You know, to [town].

Interviewer: Yes.

Respondent: Maybe put in probably [town] in that kind of vicinity as far as the [region] up towards [town], I'd say that's all that kind of you know, [region].

Interviewer: So that could be about, it's about 20 odd k to [town], so it would be about what, 50 or something kilometre radius that you're talking about?

Respondent: Maybe?

Interviewer: Maybe.

Respondent: I'm not good with distances (laughs).

Interviewer: Yeah, distances, I'm learning them myself.

Respondent: Maths isn't my forte (laughs).

Interviewer: (Laughs) no, no that's fine. OK and it's that sense of community of being in the [region] that sort of, name it for you?

Respondent: Yeah, yep.

Interviewer: Yep, OK. Now I want to talk a bit about physical activity, and as I said it's a very broad term and very individual. So I'm really interested in what does physical activity mean for you?

Respondent: Um well I'd say to me it's anything from walking or yoga or you know, actually doing cardio kind of getting your heart rate up, that kind of thing.

Interviewer: Yep, yep.

Respondent: Yeah.

Interviewer: Yep, that's really clear, yep. And do you consider yourself as physically active?

Respondent: No, not at the moment. I haven't been not since summer.

Interviewer: And can I ask why?

Respondent: Yeah, there's really no excuse I don't think for not being physically active, but my excuse at the moment, but since it's gotten dark you know I leave in the dark…

Interviewer: Yeah, and you'd come back in the dark. Hmm.

Respondent: … and I get home in the dark and I mean I could do, that's the thing I mean I could do videos in front of the telly, but I don't. You know, it's just a long day so I suppose the drive kind of I get a bit tired and I'm just…

Interviewer: Well you're working ah what? Full time.

Respondent: Yeah.

Interviewer: So that's at least a seven, seven and a half hour day isn't it?

Respondent: Yep.

Interviewer: And then it takes a while to commute backwards and forwards.

Respondent: It's about an hour and twenty.

Interviewer: Yeah, yeah.

Respondent: You know in peak hour it's…

Interviewer: Yeah in peak hour so it always takes a bit longer.

Respondent: Yeah.

Interviewer: Yeah, yep.

Respondent: So unfortunately I haven't been physically active at all.

Interviewer: You haven't had much time to be.

Respondent: Bit naughty.

Interviewer: Oh no I don't think there's any right or wrong here, it's just…

Respondent: No, but…

Interviewer: … about what's interesting is that in your life and your life at the present moment is the timing of…

Respondent: Hmm, I just keep thinking I'll get there eventually and…

Interviewer: Yeah, it takes a while to settle in. You've had lots of change for yourself haven't you?

Respondent: Yeah.

Interviewer: And winter.

Respondent: Yeah, winter's never good, and…

Interviewer: No.

Respondent: … we're in the process of me working here is to work from home a couple of days a week…

Interviewer: Oh really?

Respondent: … so once I get to start that which hopefully will be in about another month, I'm hoping that that means that I will get a chance, because I have a half an hour lunch break normally…

Interviewer: Yes.

Respondent: … so I'm thinking at least I could do that in the day up there.

Interviewer: That's a really good opportunity, a supportive company that you're working for.

Respondent: Hmm, yep. Fantastic.

Interviewer: That's great, yeah, yeah.

Respondent: I'm very lucky, so once it happens it's going to be I'm hoping that will make a difference to me.

Interviewer: Give you much more um, a bit more flexibility.

Respondent: Yep, absolutely.

Interviewer: Yep. And ah where does physical activity fit in your life? Is it of no interest, or you know, it's an interest or a priority?

Respondent: Look I'm not, I can't say I'm really motivated to do physical activity.

Interviewer: Yep.

Respondent: I wish that I was because you know I have friends and my sister are really great and they can just go off and go for a run, whereas I kind of like doing activity with other people, so if I was going to go for a walk I'd much rather…

Interviewer: You'd go with a friend.

Respondent: … go with a friend, or I want to, I want to enjoy it, and I don't really enjoy yeah, a lot of activity some times.

Interviewer: It's more enjoyable to be able to do it…

Respondent: I'm more of a team, yeah.

Interviewer: Yeah.

Respondent: Like I used to play hockey when I lived in town and that was fun…

Interviewer: Oh OK.

Respondent: … because it was a team sport, whereas I… you know…

Interviewer: Yeah, yeah.

Respondent: … and because my husband's on the farm at the end of the day he doesn't want to do, we've got a tennis court and he, that's the last thing he wants to do.

Interviewer: Yeah.

Respondent: He wants to sit down, whereas I've been sitting down all day so I'm ready to maybe do something. So it's hard.

Interviewer: Yep, and on weekends there's farm work seven days a weekend as well.

Respondent: Yeah, sometimes on the weekends I usually manage to get him to play tennis with me or I will just go for a walk on my own, but I don't really enjoy it as much as if I had a friend around the corner kind of thing to go with me.

Interviewer: Yeah, yeah. That's fair enough. And OK. Just having a look at when we were talking about the doing exercise for getting your heart rate up, in our research when we think about physical activity we think of it as any activity that you'd do for at least ten minutes that might raise the heart rate, you feel warmer, um it might be huffing and puffing a bit. So thinking about this type of physical activity during the last two weeks…

Respondent: Uh huh.

Interviewer: … OK we're just going to go through sort of different parts of your life mainly to sort of capture you know, what happens.

Respondent: Yep.

Interviewer: Um, in the past two weeks have you been active at all at work for at least ten minutes at a time?

Respondent: Yes.

Interviewer: OK can you tell me what you did?

Respondent: I've walked to the top, you can walk up to the top of a tower here.

Interviewer: Oh OK.

Respondent: And so I do that maybe twice a week.

Interviewer: OK.

Respondent: Just with some other work people, and that's really good, so that's only ten minutes, ten to fifteen minutes.

Interviewer: That's good that there's a few of you do it too together.

Respondent: It's a bit more exciting yeah.

Interviewer: And um, OK, tell me what would be the intensity of that exercise? Would it be steady, moderate or vigorous?

Respondent: Um I'd say it'd be moderate.

Interviewer: Uh huh.

Respondent: I mean you do get a little bit puffed but you, so maybe your heart rate's up a bit, but you're not running, so…

Interviewer: Hmm.

Respondent: … but it's more than your normal walk.

Interviewer: Yep, yep. And is, and do you do that during the day obviously when you're here.

Respondent: Yep.

Interviewer: Is that what you usually do? Or is it a one off?

Respondent: No I was doing it before the wedding, before I got married in February I was trying to be a little bit better about doing exercise, so I'm going to try and make that part of the routine, and more that other people are motivated it makes me a bit better when they come and say do you want to go for a walk? So…

Interviewer: A couple of times a week.

Respondent: … it's a bit easier. Yep.

Interviewer: Yeah, yeah, yep. Um and does it change depending on the season or time of the year?

Respondent: Um, workwise it would probably during our peak seasons when I'm really busy later in the year.

Interviewer: Yes. Is that around summer? Spring summer?

Respondent: It'd be a little bit harder. Yeah, yep because I do [occupation], so yeah October probably isn't going to happen.

Interviewer: Yeah, yep.

Respondent: But you can find time if you really want to really, so…

Interviewer: OK and if you wanted to, is there anything that would have helped you be more active at work?

Respondent: At work? Um maybe if we got to use the gym and the pool for free, that would be good.

Interviewer: Oh right, of course you've got…

Respondent: We get a discount but it's still quite expensive.

Interviewer: Yes, you've got those facilities, yes. OK.

Respondent: Yes that could be good, but so maybe I would use that if it was free, but other than that probably not.

Interviewer: OK.

Respondent: Yep.

Interviewer: That's good. And in the past two weeks have you walked or cycled for at least ten minutes at a time to get to or from places?

Respondent: Um, last two weeks what have I been doing? Just in a work basis are you saying, not at home?

Interviewer: No, no that can be anywhere in your life.

Respondent: Oh OK. Have I walked? Yes I did go for a walk last weekend.

Interviewer: OK.

Respondent: Yep.

Interviewer: OK can you tell me um what you did?

Respondent: I went for a walk with some family on the farm just for about half an hour, not long.

Interviewer: OK that was on the farm itself?

Respondent: Yep.

Interviewer: Yep.

Respondent: And I went for a walk, I do sometimes go for a walk with a friend in town after work.

Interviewer: OK in [city]?

Respondent: Yep.

Interviewer: Yep.

Respondent: And then drive home.

Interviewer: And where do you go for a walk?

Respondent: Um sometimes on the bike track…

Interviewer: Oh yeah.

Respondent: … or she lives in [suburb] so out that way.

Interviewer: That's handy.

Respondent: Yeah.

Interviewer: OK now thinking about those two activities, what would you describe the intensity of them as being? Steady, moderate or vigorous?

Respondent: Um the one on the farm was just steady. It wasn't overly fast, it was more leisurely.

Interviewer: Uh huh.

Respondent: And with my friend in town it's a bit more for exercise, so probably it's moderate.

Interviewer: A bit brisker, yep. Yep. It's great that cycle track.

Respondent: Yeah, it's nice isn't it? Nice and flat.

Interviewer: Yeah. And the duration of the activity?

Respondent: Um on the farm it was only half an hour I would say, and in town probably 45 minutes.

Interviewer: Uh huh. And how often? Now you said on the farm was once and the weekend, and what about the walk in town?

Respondent: Usually I'd say once a fortnight.

Interviewer: OK.

Respondent: Yep.

Interviewer: OK and when did you do it? The weekend would have been during the day?

Respondent: Yes.

Interviewer: And the walk with your friend in the Hobart's after work?

Respondent: After work yep.

Interviewer: And why did you do it at that time?

Respondent: Um the one on the farm was just because we had family staying over.

Interviewer: Uh huh.

Respondent: So we wanted to go for a walk in the morning, and why did I do it at that time? The one in town was just because that's the only time I can really go.

Interviewer: After work.

Respondent: After work yeah.

Interviewer: Yep, yep. Um, now you've told me where you've done it, um why did you do it there? What attracted you to that place?

Respondent: Um well the walk on the farm was just because it's there obviously, um and in town we went around near town because my friend lives there, so it's easier. And then it's sort of on the way home, I don't have to go out of my way anywhere really.

Interviewer: Yep.

Respondent: So it's nice.

Interviewer: And um, OK and you were doing the walk with other people?

Respondent: Yeah.

Interviewer: And is this what you usually do, or is it a one off?

Respondent: Um, the walk, no I would say that it's - I do do it, but it's not really, it's not a routine, so I would usually try and go for a walk on the weekends at the farm.

Interviewer: Yep.

Respondent: Or in town, wherever I am really, I'll try and do something, it's probably not going to be really hard core, you know kind of exercise, I haven't done anything cardio vascular for a long time but the walking in town with my friend after work we try and do that reasonably regularly, as I said once a fortnight.

Interviewer: Yep, yep.

Respondent: You know, we try for once a week but it's not always possible, so…

Interviewer: Yes, so yeah you've got to balance it out don't you.

Respondent: Yeah, it's not a priority, even though you know I have this thing and I think it should be you know, I should really make time for exercise. I'm a bit naughty.

Interviewer: It's hard when it's dark so early now isn't it?

Respondent: I just don't feel like it.

Interviewer: No, I know it draws you in doesn't it…?

Respondent: (Laughs) I know it's terrible.

Interviewer: … to go inside when you get home.

Respondent: Yep.

Interviewer: And does this change depending on the time of year?

Respondent: Um yes, yeah. Over summer because it's light until late it's so much easier, so during summer when I was driving home it would still mean that we could have a game of tennis, or I could go for a walk and the same in town as well. Like it wouldn't matter so much about the timing, whereas now I'm kind of because it's dark I just really want to get home so I'm not really feeling like doing it as easily. Yeah.

Interviewer: Hmm… OK and if you'd wanted to, is there anything that would have helped you to to walk to and from places?

Respondent: Um…

Interviewer: More.

Respondent: To and from places?

Interviewer: Yep.

Respondent: There's nowhere… I mean up at [town] you don't really walk, I mean you can't walk anywhere, I suppose like for a purpose kind of there's no end destination if you know, like you can't walk to the shop or anything like that.

Interviewer: Hmm, it's a bit too far.

Respondent: Yeah.

Interviewer: Yes, you'd have to go in your car and go in. Hmm.

Respondent: Um… no I mean we get free parking here, so if we didn't get that then I'd park somewhere and have to walk, so that would be good.

Interviewer: Yep, yep.

Respondent: But I wouldn't want to give up my free parking.

Interviewer: No (laughs).

Respondent: Much more convenient (laughs).

Interviewer: Absolutely.

Respondent: So…

Interviewer: So is there anything that would have helped you in terms of walking more?

Respondent: I mean at the farm obviously there's no lights so you can't, I mean that's why I walk in town after work because at least we can just walk under the lights and it's fine.

Interviewer: Yep.

Respondent: So up there, definitely having somewhere that you could walk that was lit, that would be good.

Interviewer: Yeah, yeah.

Respondent: Or if there was you know, like a yoga session in [town] or...

Interviewer: Oh yeah, yes.

Respondent: … [town] if there was those kind of things maybe I'd look at doing something like that maybe.

Interviewer: Yep, OK.

Respondent: Um, it's just a bit hard really with winter I suppose. Daylight savings is so much easier.

Interviewer: Yeah, yeah.

Respondent: And I mean you could, you could probably try and go for a walk because I was thinking I could go for walks in the morning before work when it's starting to get light, but you can't quite see that well and it's too uneven, it's just once you start walking in paddocks and things and there's rocks and, you don't know where you're really going. Thistles. That's not really the best. Hmm.

Interviewer: OK. Now in the past two weeks have you been active around the the house or the yard? So at home inside or outside? For at least ten minutes at a time?

Respondent: I'd say…

Interviewer: Can you tell me what you did?

Respondent: … I'd say cleaning.

Interviewer: Yes, yes.

Respondent: Cleaning the bathroom and doing the vacuuming.

Interviewer: Yep, yep.

Respondent: Slight bit of exercise, um and what have I done? I did get in the garden a couple of weeks ago and that was quite good. I was in there, that was a good sort of two hours which was a nice change. I'm just learning about gardening so I'm not really in there that much, and I do play, I've played tennis a couple of times as well with family when they've come up.

Interviewer: Yep. OK so thinking about those activities, again what would you consider the intensity of them as being?

Respondent: Um, probably the gardening, oh no that wasn't intense, I'd say just moderate.

Interviewer: Moderate? Uh huh.

Respondent: Yeah, yep.

Interviewer: And the cleaning?

Respondent: Probably moderate maybe, or maybe less, yeah less probably.

Interviewer: Steady to moderate?

Respondent: Depending on what activity, if it's scrubbing the shower sometimes you get quite into that…

Interviewer: Yes (laughs).

Respondent: … but if it's just hanging out the washing that's not really that strenuous.

Interviewer: And you can certainly get warm doing housework.

Respondent: Yeah, absolutely, you get right into it; it's a good bit of exercise.

Interviewer: And the tennis, what would you call that as being?

Respondent: That's probably a step up, that's, yeah.

Interviewer: So moderate to vigorous?

Respondent: Yeah.

Interviewer: Yeah, yep. Certainly. Um now let's see. Um, how long would you have done those activities for?

Respondent: The tennis was probably only; we got thrashed by my dad and my step mum, so that was only half an hour probably again.

Interviewer: Uh huh.

Respondent: In the housework depending, I suppose altogether you're doing, you know there's all lots of little jobs as you know, so I don't know, probably two hours maybe.

Interviewer: Yep, yep.

Respondent: On and off though, it's not really you know you might stop and have a cup of tea and then go and clean the bathroom.

Interviewer: And the gardening was a couple of hours wasn't it?

Respondent: Oh that was two hours.

Interviewer: Yep. And the frequency? How often would you have done it in the last couple of weeks?

Respondent: House cleaning I generally would do once a week.

Interviewer: Yep.

Respondent: And gardening I haven't as I said I'm just sort of getting into it, so that's only really been oh probably only a handful of times since I've been living there since August, so not regularly.

Interviewer: Yep.

Respondent: And tennis is whenever I've got someone that wants to play I'll try and play, but that might only be sort of at the moment probably only once a month, whereas in summer it was more, yeah.

Interviewer: OK so when did you do those activities and why?

Respondent: Tennis was on the weekend. Housework was on the weekend and gardening on the weekend.

Interviewer: Yep, they're all…

Respondent: On the public holiday.

Interviewer: So they're all, that's because that's the time you have available?

Respondent: Yeah, absolutely.

Interviewer: Yeah OK. Yep. OK. And who with? Thinking about those activities?

Respondent: Yep, the house keeping's on my own, and the gardening and I mean gardening sometimes is just on my own or with my husband, but the time that we had the two hours in the garden was with my dad and step mum and the tennis was with my dad and step mum.

Interviewer: OK.

Respondent: Yep.

Interviewer: And is this what you usually do, or was it one off?

Respondent: I'd like to think that it wasn't a one off, I'd like to think that it was a bit more regular, but that's really just going to depend on when they come to stay, so they're in Hobart so they'll probably come up to stay once every couple of months, but generally whoever comes to stay I'll try and get them to…

Interviewer: To do things.

Respondent: … go for a walk or yeah.

Interviewer: Yeah.

Respondent: Yeah I did go for another walk the week before yeah. So I'm generally going for a walk on the weekends.

Interviewer: And usually on the weekends, would that be on the property that you have the walk?

Respondent: Yeah.

Interviewer: Yep, OK. And does it change depending on the season or the time of year?

Respondent: Yep, so just depending on daylight hours really, yep.

Interviewer: Yep.

Respondent: And warmth as well you know when it's cold I don't really feel like doing a lot.

Interviewer: Yeah.

Respondent: But when it's summer you know, tennis is such a summer sort of sport, you kind of feel like it a bit more.

Interviewer: Hmm. And if you'd wanted to, is there anything that would have helped you be more active around the house and yard?

Respondent: Um again with light you know if there was light to see, um not really in the house because you can do that really any time because you can see, so sometimes after work I might do that.

Interviewer: Uh huh.

Respondent: Um if we had lights on our tennis court that would be good (laughs) so again it's really just lighting I'd say, yeah.

Interviewer: OK. Um and have you been physically active for at least ten minutes during your leisure or spare time? Sort of the last part of, now you probably have covered that a bit, but is there anything else that you would like to add about your spare time?

Respondent: Um… I don't think I've really done anything outside of that.

Interviewer: Yeah outside the walking and things like that?

Respondent: No, probably not.

Interviewer: These questions are just aimed so they make sure they capture each part of life, but you know things come up and… it's fine.

Respondent: I had a kick of the footy with my husband last weekend that was fun.

Interviewer: Wow! (Laughs).

Respondent: (Laughs) that was good fun, for about ten minutes and then I was really puffed.

Interviewer: Oh great. Yeah. And is that something you'd normally do or?

Respondent: No.

Interviewer: No. Something you mighten do again.

Respondent: It was a bit random yeah.

Interviewer: Yeah. That's fine. OK now the last part of it is just talking about the physical environment, and so we've talked a bit about where you live and your physical activity. Your physical environment is where you live, work and play and when we talk about physical environment it's about all those things that surround you, like anything from roads to trees to houses or traffic or whatever parks. And some of the things these things are things that people who live in the city have said could be of beneficial influence in their, in being physically active. So we're interested in finding out well what happens for people who live rurally.

Respondent: Hmm OK.

Interviewer: So can you tell me a bit about how easy or difficult it is to be physically active in your area? In your community.

Respondent: Yep so in [town]?

Interviewer: In [town].

Respondent: Um it is look it's not; it would probably be quite easy during summer.

Interviewer: Yep.

Respondent: When you've got so many more hours there, um…

Interviewer: And it's light.

Respondent: … yeah it's light and it's warmer so you feel like it a bit more. If I wasn't working full time then again it's around daylight hours, so having the time to do it. Um what else about it?

Interviewer: So would you see it easy or difficult? It sounds like it's difficult in winter…

Respondent: It is, yeah. I would say it is difficult, because you just don't have access to as many things. You haven't got street lights, you haven't got walk ways or nice little paths that you're just kind of out in the bush, it's not…

Interviewer: So you would either walk on the property which obviously means it needs to be light.

Respondent: Yep.

Interviewer: And but you're far enough out of the town that you'd have to get in the car to…

Respondent: Yep to get into [town].

Interviewer: … actually go to [town] or anywhere else that you might pick.

Respondent: Yeah, absolutely.

Interviewer: Yeah, yep.

Respondent: So I usually go…

Interviewer: Are you actually on the main road itself?

Respondent: Um yeah on the main road and then you, our drive way's 2 kilometres until you get to our house.

Interviewer: Oh OK so you've got a two kilometre drive, yep.

Respondent: It's definitely a lot different, when I was living in town it was so much easier, winter or not because I'd still have you know, the gym, I didn't really go to the gym but I used to go to the gym so that was a few years ago now, and just going for walks with friends I didn't have to be worried so much about the timing of that because I could just be

down at my friend's house in five minutes and go for a walk or you know, go to any kind of spin class or you know those kind of things that we just don't have the options of.

Interviewer: It's quite different.

Respondent: So, it is I'd say it is a lot more difficult.

Interviewer: It's a lot more difficult.

Respondent: Yep.

Interviewer: No that's really good, interesting to hear. Now I'm going to go through a few features of the physical environment. The first one's availability and accessibility of places to be active and we've touched on this a bit I think already.

Respondent: Yep.

Interviewer: If I were to ask you about places to be active in your area, what sorts of things would you tell me about?

Respondent: What is available in my area?

Interviewer: Yeah. Like are there any recreation facilities, or parks or walking tracks?

Respondent: Yeah. There's no walking tracks from what I know of. There is a swimming pool, a public pool, I've never been there, I don't know what it's like.

Interviewer: Uh huh.

Respondent: Or if it's open…

Interviewer: If it's open all year round or not.

Respondent: … at different times or what it's like.

Interviewer: Yes I actually asked about that…

Respondent: Did you? Yeah.

Interviewer: … the other day. It's only open in summer. It's just actually closed.

Respondent: Oh right.

Interviewer: For winter.

Respondent: OK lovely, and it wouldn't be heated I'm sure.

Interviewer: No (laughs).

Respondent: There are street lights on the main road in [town], but it's only a small town so you'd just be like walking up and down the road.

Interviewer: Yep.

Respondent: And I'm not really sure how comfortable I'd feel really doing that, it's still quite dark and…

Interviewer: Yes.

Respondent: I don't know, there's a lot of empty houses, it's just a little bit weird I think.

Interviewer: Yeah. Yeah.

Respondent: So I'd just rather be somewhere I felt a bit more secure I suppose.

Interviewer: Have you got access to any parks or any other walking areas?

Respondent: There's only, there is a little park there but it's basically I think it's just a playground really opposite the church there you may have seen on the corner. It's tiny.

Interviewer: Yes I remember it's tiny.

Respondent: You're not really going to walk around there. There's a football ground.

Interviewer: So you've got a better option on your property for walking.

Respondent: Yeah.

Interviewer: From that point of view.

Respondent: Absolutely, yep. There may be places that I don't know of, but not that I've heard of, yeah.

Interviewer: Yeah, OK. Um so there's a bit of a lack of places to be active and um they're not really very, well they're not there, so they're not accessible are they?

Respondent: No (laughs).

Interviewer: And does having, or not having, places to be active influence whether you're active or not?

Respondent: Yes. I'd say so.

Interviewer: Because you were saying in town it's quite different.

Respondent: Yeah, a difference.

Interviewer: And you make the most of once a fortnight catching your friend and going for a walk.

Respondent: Yeah, absolutely we like to have a giggle.

Interviewer: And if you wanted to be more active, are there things or places that would help if they were available or more accessible?

Respondent: Yeah I think so, if there was a gym or as I said before like a yoga, I enjoy that kind of thing, you know like different classes.

Interviewer: Is there a hall perhaps that is in [town] that…

Respondent: There is a hall yeah.

Interviewer: There is you know there is actually a facility to have something like that?

Respondent: Yeah.

Interviewer: Yep.

Respondent: If someone wanted to run a class or something that would be interesting.

Interviewer: Hmm.

Respondent: I mean I'd, I wouldn't say that I'd definitely go, but I'd go and see what it was like, I'd have one session and see and you know…

Interviewer: Yeah, well that'd be good.

Respondent: (Inaudible 34:02).

Interviewer: Now I've heard this again about [town] that people would really like some sort of activity like yoga or Zumba.

Respondent: Oh yes, oh that's good.

Interviewer: So there's a few people interested.

Respondent: I know I didn't think of that, I was thinking oh I should do a yoga course and have a little class or something.

Interviewer: Yeah, that would be lovely.

Respondent: I think that would be really nice. But…

Interviewer: Well particularly in your own locality and it's sort of peaceful and all you have to do then is just go home afterwards.

Respondent: It'd be really nice, yeah.

Interviewer: It would. A nice…

Respondent: Something I'd like to think about long term maybe.

Interviewer: A nice environment, hmm. And if, OK um OK. We've talked about… now the next one is destinations. If I were to ask you about places that you could walk or cycle, walk from your home, what sorts of things would you tell me about?

Respondent: If I could walk somewhere?

Interviewer: If you could walk, is there anywhere that you could actually walk to? Like the shops or services or friends?

Respondent: That's what I would be walking towards, yeah when I lived in town sometimes I'd walk to the shop or I'd walk down to [suburb] somewhere.

Interviewer: Yes, yep.

Respondent: That would be really nice, but where I live it's way too far to walk anywhere.

Interviewer: Yeah you have to get into a car. Yep.

Respondent: Yeah. You could walk to one neighbour's house, but in the house it wouldn't be the best going across the cattle grid, get a broken ankle.

Interviewer: No, no, no. OK. And how far is that neighbour?

Respondent: Ah two kilometres.

Interviewer: OK so that'd be a fair walk too.

Respondent: Just under.

Interviewer: Yep, yep, especially when you have to come home again.

Respondent: Yep, absolutely.

Interviewer: Um so that, yeah at night the only option then in terms of light would be in summer or in the weekend.

Respondent: Yep.

Interviewer: And that would be your closest neighbour would it?

Respondent: Yep.

Interviewer: Yep. Does having, or in this case not having destinations influence whether you're physically active or not?

Respondent: Yes, I'd say yes because if I was near a shop or you know I needed to get something it'd be nice just to be able to say oh I'll just walk to the

shop I'll just walk in and get us the paper or a coffee like I really miss that about living in town.

Interviewer: Yep, yeah.

Respondent: Such a big part of the weekend for me was oh I'll walk down to [grocer] and get us the paper and fresh loaf of bread, you know some times you'll drive into [town] and you can't get, you know the bread's sold out so too bad.

Interviewer: [grocer]'s great, I'm enjoying [grocer] (laughs).

Respondent: It's so good (laughs) yep I do miss that, you're lucky.

Interviewer: Yes, I can understand that.

Respondent: Yeah.

Interviewer: Um and if you wanted to be more active would it help if there were more destinations within easy walking distance?

Respondent: Yep.

Interviewer: Yes it would.

Respondent: Yep.

Interviewer: Yep. OK now I'm going to look at neighbourhood design which is the built aspects of your area which is manmade or non-natural features. And if I asked you about those, the non, the man made features, what sorts of features would you tell me about them?

Respondent: Um, manmade features? Well [town]'s quite old so there's quite a few old buildings in town which are quite pretty.

Interviewer: Yes, yes.

Respondent: Um, I don't know what else to say about it really.

Interviewer: You've touched on…

Respondent: The wide streets.

Interviewer: … yeah you've touched on the fact that you don't you don't have footpaths where you are.

Respondent: No.

Interviewer: They're mainly what in the town?

Respondent: No.

Interviewer: You don't think they're in the town either?

Respondent: No.

Interviewer: Yeah. There's sort of gravel on the side of the road.

Respondent: Just the gravel, yep.

Interviewer: And lack of lighting?

Respondent: Yep.

Interviewer: So the walkability, even if you wanted to it's a fair distance out, but could you actually walk on the main road at all?

Respondent: In [town] or in town?

Interviewer: Yeah.

Respondent: You could…

Interviewer: Or from your property.

Respondent: Oh you couldn't walk, even if I walked out to the end of my driveway and walked on the road it'd be really dangerous.

Interviewer: Yes.

Respondent: Because there's lots of corners and people wouldn't be able to see you.

Interviewer: Can't see you.

Respondent: Yeah, it'd be really bad. But in [town] itself you could go for a walk probably around there, but I don't think it's even still that light like I think there's a, I don't know, I've never really noticed it I suppose, but I'm not sure that the tele, like the actual lighting is that close together.

Interviewer: Yep.

Respondent: And there's just a lot more lights in the city in general anyway so there where you've got houses that are moved back quite a way from the street, you've only got a few lights, I just don't think even then it would still be that good probably.

Interviewer: OK. And do you think the built features in your area influence whether you are physically active or not?

Respondent: The built features…

Interviewer: Or the lack of them.

Respondent: Yeah well if there were lights there then it'd be differen.t

Interviewer: Yes.

Respondent: Or a safer footpath, if you had a family if I had a little kid that wanted to go for a bike ride or something I'm not sure if I'd, I mean in the day you could maybe.

Interviewer: But even if you wanted to have yeah, even if you wanted to go for a walk from your property…

Respondent: Yep.

Interviewer: … you were saying its, the road isn't safe anyway, so…

Respondent: No.

Interviewer: … even on the, so not enough visibility and the road sounds like it's a bit narrow and twisty.

Respondent: Yep absolutely. Yep.

Interviewer: So that's…

Respondent: And there's a lot of like trucks and things that go through [town] so I don't reckon it's safe. You know probably that might be alright, but I wouldn't do it if I had a baby or a stroller or something and…

Interviewer: Well it's a very common one; this is what women are saying it's just not safe to walk on the road.

Respondent: No.

Interviewer: Or on, often you don't have a choice, even if getting on the side of the road, there's not enough room or it's a slope or it's gravelly or it's not very safe walking anyway.

Respondent: It's not enjoyable there.

Interviewer: Nope, nope. No. Yeah when I lived rurally and walking along the road is not exactly my idea of fun. NO.

Respondent: Nup that's it.

Interviewer: No you don't normally go to… yep.

Respondent: Exactly. The other thing I didn't mention there is a golf course in [town] that's probably for me a bit weird.

Interviewer: OK.

Respondent: But I don't play golf, so…

Interviewer: You have to play golf then don't you?

Respondent: (Laughs).

Interviewer: So if you wanted to be more active, are there any built features in your area that would help?

Respondent: Um as we touched on before the community hall is something that could be utilized probably.

Interviewer: Yep.

Respondent: Um, and there's also the pool we talked about and I'm sure there must be a gym, a gym at the school so I don't know what they've got in there.

Interviewer: Yeah because…

Respondent: Those things already in place.

Interviewer: … often the schools become a bit a bit more open to the community.

Respondent: Yeah.

Interviewer: These days too. Yep. OK. Now the other side of it is the natural part of the environment. If I were to ask you about the aesthetic qualities or the attractiveness of your area, what sorts of things would you tell me about?

Respondent: It is nice having that tiny playground there, it's good because there's a few trees in there.

Interviewer: Oh yeah.

Respondent: But it's really small. The natural features?

Interviewer: Yes, like you often walk on the property.

Respondent: Around the farm, yeah that's beautiful. We've got a lot of bushland and you can walk down to the river as well that's nice.

Interviewer: Oh how beautiful.

Respondent: But it's covered in gorse bushes so you can't really walk along the river, so it's not really that beautiful at all, but it's still nice being near water. And in [town] there's the [x] River that goes through which is nice and then it goes pretty much into farmland so you can't really walk anywhere else really outside of that.

Interviewer: Yeah, so it's limited. There's not the what we would call the walks don't connect so you can't sort of…

Respondent: No, you can't keep going. You could walk down maybe near the bridge there to the river, but I don't think there's any actual grassy bit maybe.

Interviewer: Yeah, it's another comment that's coming out very clearly is that it's just not thought out, so it's limited you know you find yourself not being able to like in one area people can't get down to the river you know, even when they put a park there you can't actually get there because there's no access, and…

Respondent: Yeah, I don't, yeah.

Interviewer: … so just sort of little things like that.

Respondent: Little things like that. Because it's a nice spot, but…

Interviewer: That aren't huge but it makes a difference.

Respondent: Yeah, I think so I don't, yeah… it's probably true, you probably can't get even get down to it, I'm not quite sure.

Interviewer: And so do you see your local environment as aesthetically pleasing and attractive?

Respondent: Yes.

Interviewer: Near your home.

Respondent: Yeah, yep. I think it's really nice.

Interviewer: Yeah. You've got it sounds like fairly undulating country.

Respondent: Yeah it's beautiful.

Interviewer: And bushland as well as those open spaces of farmland.

Respondent: And at the moment in autumn with all the trees turning…

Interviewer: Oh yes.

Respondent: … it's awesome.

Interviewer: It's been a beautiful autumn.

Respondent: Yeah.

Interviewer: And if you wanted to be more active, are there features of your area that might help if they were more aesthetically pleasing or attractive?

Respondent: Um… yeah if it was you know accessible like if there was a pathway along the river that would be lovely to be able to walk along there.

Interviewer: Yes.

Respondent: That's what I would say with the natural kind of environment, incorporating that, but otherwise we'd need to be in you know, an actual man made structure I suppose.

Interviewer: Yeah, yeah.

Respondent: But along the river that's really in the actual town that's the only aesthetically nice place you could really go unless we had street lights and maybe proper sidewalks.

Interviewer: Yep, yep. And you're saying that that's limited at present moment in terms of just how far you can go?

Respondent: Yeah.

Interviewer: Yeah.

Respondent: I don't think the government would spend that much money to put in a sidewalk in a community of 300. Probably, a big investment but, I don't know there might be ways around it you know like a pathway along the river.

Interviewer: Yeah, yeah.

Respondent: I don't know. Because there's a lot of people that just live at least on the land so we can just go for a walk out there, but I feel sorry for people in the town because they don't have that chance.

Interviewer: Yep, and often people come up with fairly simple solutions too that don't cost huge amounts of money, so it's really helpful asking these sorts of things.

Respondent: Yeah. It'd be interesting to see what people suggest if you know.

Interviewer: Yeah, um so just one other quick area to ask you about, and that's about safety. What sorts of things would you tell me about if I asked you about safety in your area?

Respondent: Um I would say that in terms of exercising it's not probably what I would call safe.

Interviewer: Yep.

Respondent: Um there's a lot trucks, you know people moving sheep and things around, it's you know, I don't know… because there's no lighting so, and there's no footpaths, it's not really…

Interviewer: So the roads aren’t very safe?

Respondent: I wouldn’t say it’s overly safe, and people come flying through there as well. It’s a 60 zone but I must admit even when I’ve been going along and you know last minute you’ll see a little boy on a bike or something and think god, this is a bit dangerous, you know, because it’s sort of in the middle of - it feels like [town]’s in the middle of nowhere sometimes because you’re driving along and sudden you’re in the town.

Interviewer: Then you’re in it and then you’re out of it.

Respondent: And out on the other side, yeah. It’s a weird little set up there really.

Interviewer: Really.

Respondent: So yeah.

Interviewer: So um does road safety influence whether you’re physically active or not?

Respondent: Yes. I would say so because I wouldn’t feel, as I was talking about before, just with walking in the town I wouldn’t feel overly safe. I don’t know anyone in the community to sort of make sure that you know it’s all OK because there’s not many people around, I don’t know like I’d feel safe in that sense of other people being violent or whatever.

Interviewer: So that’s pers, and that would be personal safety for you too.

Respondent: Yeah, personal safety as well as getting hit by a car who hasn’t seen me.

Interviewer: Road safety, yeah absolutely.

Respondent: Yeah so both aspects.

Interviewer: Yeah, yep. Particularly when it’s the, the visibility isn’t good.

Respondent: Yeah, definitely. It’s very quiet and I’d probably spook myself out even if I went for a walk in town I think because it’s you know, eerily silent I suppose as well.

Interviewer: Yeah, yeah. And if you wanted to be more active, are there any personal or road safety issues that if addressed might help you be more active?

Respondent: Yep, just again with the lighting that would be one good one. Um footpaths again would be good…

Interviewer: And as you said…

Respondent: … more people living there.

Interviewer: … yeah exactly, and as you said the kids it's really important it's like you were saying about little kids riding bikes.

Respondent: Think about… yeah it's so dangerous because you know I'd like to have a family but I wouldn't take a stroller around the streets of [town] probably. In the day time it'd probably be fine, but…

Interviewer: But you'd have to walk on the gravel.

Respondent: … if it was slightly dark. Yeah, and it wouldn't be that enjoyable really, I'd rather face the paddocks and you know…

Interviewer: Yeah.

Respondent: Or our driveway is probably just as safe.

Interviewer: Yeah, well there's that thing too that you know you live rurally not to not to walk the streets.

Respondent: No.

Interviewer: It's about the beauty of the place and you want to, yep.

Respondent: I'd rather come into town and take my stroller around the streets here probably.

Interviewer: Yeah, yeah, yep.

Respondent: Hmm.

Interviewer: OK so just before we finish up, is there anything else that you'd like to tell me about where you live or your physical activity or the environment that you live in that we haven't covered today?

Respondent: Um I don't think so, no, about covered it, yeah.

Interviewer: That's great.

Interviewer: So I thought I might start by asking a bit about the place where you live, and if you'd like to tell me a bit about what it's like to live where you do and your likes and your dislikes.

Respondent: OK. Well we've just moved to, last September, we bought a house on the coast which is across the road from the beach at [town], so it's really nice. We've got a big garden and we came from the country which said at [town] and we still own that property, 110 acres or something. But moved down the coast for a change of lifestyle thing and to, you know, down the track we'll sell our property 'cause we're both getting older. And, no, it's nice, it's across the road from the beach so even though they don't have footpaths, there's lots of nice walks along the beach and things like that. Yeah.

Interviewer: Was there a particular reason you picked [town] itself?

Respondent: It's still a little bit rural without being a long way from everything, so you still have the access of either [town] or [city] or, you know, close to facilities and things like that, without being on top of people, yeah.

Interviewer: And you're living in a house and is it a house block or it's like larger?

Respondent: It's a double block, yeah, it's a double block, with a big...

Interviewer: So it's about an acre, half with that?

Respondent: I think it's about three quarters of an acre.

Interviewer: Yep. And what would be the distance to your closest, sort of town's not quite the right word...

Respondent: No.

Interviewer: ... but how far are you from [town] and [city]?

Respondent: I think we're five kilometres from [town], and I think we're probably eight or nine from, yeah, the outskirts of [city] sort of thing, yeah.

Interviewer: So when you think about your next closest village or township...

Respondent: Yeah.

Interviewer: ... which one would you...

Respondent: [town]. [town] would be.

Interviewer: You would think of [town]. Yep. OK. So, as I was saying, most of this research has been done in urban areas and people often refer to their local neighbourhood, and I'm interested in understanding what the term neighbourhood means to women who are living in rural areas, if it's relevant at all. So what does the word neighbourhood mean to you?

Respondent: I think like a community, your community, your neighbours either side sort of thing. When we lived in the country we didn't have neighbours, but people that shared our boundaries sort of thing, even though a long way away, I'd say they would be our neighbourhood.

Interviewer: Be your immediate neighbours, yes.

Respondent: Yeah. And even in the country where everyone is spread out, there's still a sense of belonging in a community, you know...

Interviewer: Well, that was the other question I was going to ask you is does the word community have meaning for you and what meaning would it have?

Respondent: Probably similar to neighbourhood and community and, yeah, things that go on in that area of where you live is all part of being the neighbourhood and community, yeah.

Interviewer: So now when you live in [town], thinking about neighbourhood first up and then I'll ask about community...

Respondent: Uh huh.

Interviewer: ...if you needed to put a distance around it, what sort of distance would you put?

Respondent: In [town] I'd put a couple of kilometres, say two kilometres sort of thing is a neighbourhood in that area, you know, something like there's a community hall, there's one little shop. But, yeah, 'cause, you know, I do lots of walking and you see the same people and they're part of that, you know, you see each day, even though you don't know who they are but you just wave at them, yeah.

Interviewer: Yeah. And people are obviously friendly enough. Then, OK, if you had to put a distance around community, would that be the same?

Respondent: Probably I think community's a bit more extended, yeah.

Interviewer: Yes. OK, so what sort of...

Respondent: So you'd probably go into [town], that would be part of that community, and the activities that go on in that community, just...

Interviewer: So you were saying, how far was [town] again?

Respondent: Probably five kilometres, yeah.

Interviewer: Five, yep. OK. So that's more a five kilometre, OK.

Respondent: Yeah.

Interviewer: Now I wanted to talk a bit about physical activity and what that actually means for you. So what does that term mean for you?

Respondent: Doing some sort of exercise, whether it might be vacuum cleaning the house or gardening, you know, going for a walk. It doesn't have to be arranged, you know, exercise activity that you do at the same time or, you know, that you get...

Interviewer: So it's a bit like activity in your life, your (indistinct 5.30) in life?

Respondent: Yeah, that's what physical activity is for me.

Interviewer: That's coming out loud and clear too, that women see it as much broader...

Respondent: Uh huh.

Interviewer: ... and in their day to day, so, yeah.

Respondent: And do you consider yourself physically active?

Interviewer: I'd say so, yeah. Yeah, I think so, yeah.

Respondent: And why?

Interviewer: Oh I go for a walk just probably every morning, before work, go for a walking every, you know, half an hour to 40 minute walk every

afternoon after work. Do something physical like either a bushwalk or, I don't know, what did I do this weekend, probably nothing? Something on the weekend was a bit more strenuous that you may have arranged with your friends or something, but, yeah. I don't go to a gym or anything, but, yeah. I consider myself active.

Respondent: Absolutely, every day. And where does physical activity fit in to your life, is it no interest in particular, priority, high priority?

Interviewer: Oh, yeah, I think it's a priority, priority to me to keep fit and to be active so that, you know, you don't... all those aches and things that come with old age, you know, it's important to keep fit and healthy. I think it's everyone's responsibility for themselves really, yeah.

Respondent: OK. So I just want to talk a bit more about physical activity. In our research, we think of physical activity as any activity that lasts for at least ten minutes...

Interviewer: Yeah.

Respondent: ...that's to feel the health benefit effects, and that you can be, you know, that you feel your heart rate going up a bit, feeling warmer and could be a bit of huffing and puffing with the breathing. So thinking about this type of physical activity over the last two weeks, in the past two weeks, have you been active at all at work for at least ten minutes at a time?

Interviewer: At work? No I don't think so at work.

Respondent: Or getting to work, or...

Interviewer: No. Not that your heart rate would go up.

Respondent: ‘Cause you’ve got a job that’s...

Interviewer: Yeah, I’m pretty deskbound job.

Respondent: ... deskbound, yeah.

Interviewer: Yeah. OK.

Respondent: Probably the biggest physical activity for us is to walk up the stairs; three flights of stairs which, yeah, not enough people use the stairs. There’s a lift in the building, so, but yeah.

Interviewer: And that seems to be a really common one that all women are sort of saying about, you don’t get to do exercise at work, well, I certainly I don’t, no.

Respondent: No.

Interviewer: Sitting down. And if you wanted to, is there anything that would have helped you be more active at work?

Respondent: Yeah, I think it should be a priority, especially I work in the [workplace], it doesn’t seem to be a priority. We’re not encouraged or... even though we’ve got a park outside, we don’t make a point of using it. And it’s probably up to us as our team to be more active, but we don’t because we’re all busy doing what we’re paid to do, you know, so it’s...

Interviewer: And it doesn’t get less so, it gets more so busy.

Respondent: It’s not a, yeah, it’s not a priority where it probably should be.

Interviewer: So can you think of anything that might help be more active?

Respondent: Arranged walking times and things like this. Arranged, I don't know.

Interviewer: It sounds like even a bit of encouragement at work might too from...

Respondent: Yeah. Arrange, you know, an exercise class or something out in the park on the nice days or, just to get outside in the sunshine on the nice days. But, yeah, we're all sort of heads down doing what you're supposed to be doing.

Interviewer: In the past two weeks have you walked or cycled for at least ten minutes at a time to get to or from places?

Respondent: Yeah, both, yep.

Interviewer: Can you tell me what you've done?

Respondent: Yeah. Usually on the weekend I cycle in and out to [town]. Talk the dogs for a walk twice a day. Do yoga twice a week after work.

Interviewer: Now thinking about those activities...

Respondent: Uh huh.

Interviewer: ... can you tell me what the intensity of them, like whether a steady, moderate or vigorous?

Respondent: I'd say moderate or steady would be the words to use. Yeah, I don't flat out ride a bike or anything, not a racing bike; it's just getting from A to B.

Interviewer: And the walking as well?

Respondent: Yeah. Walking, sometimes I do a bit harder pace walk, but, yeah, most the time it's just, yeah, exercise or...

Interviewer: And thinking of those activities that you've done, the bike riding and the walking, how long would you have done them for?

Respondent: Walking's half an hour to 40 minutes, and the bike ride usually takes me, I don't know, half an hour again, half an hour and half an hour back, an hour or so.

Interviewer: And how often would you have done them

Respondent: Walking each day twice a day, bike ride just once a week.

Interviewer: OK. And, OK, you've told me about when you do the cycling was at the weekend, and that's during the day. The walking is during the week, at what time of the day...

Respondent: Yeah, morning.

Interviewer: In the morning.

Respondent: 6:30 to quarter past seven, and then it's usually 5 o'clock to a quarter to six or something.

Interviewer: And why did you do those activities at that time?

Respondent: Just to fit it in, because it's before work and after work really, and I take more time on the weekends so I can do it a later time or, you know.

Interviewer: And where did you do your cycling and walking? You've told my cycling's [town] and walking?

Respondent: Yeah. Walking's along the beach at [town] midway point.

Interviewer: So local...

Respondent: Yeah.

Interviewer: ... for you?

Respondent: Yep.

Interviewer: And how far away is your beach did you say?

Respondent: It's just across the road, but you have to walk probably five minutes up the road to...

Interviewer: To be able to access.

Respondent: ... to get the, yeah, sandy beach, yeah.

Interviewer: OK. And why did you do it, those two activities?

Respondent: Yeah, it's accessible; it's easy to get to. It's a nice spot, yeah.

Interviewer: Nice spot. And is that what you usually do or is it a one off?

Respondent: No, I usually do that, yeah.

Interviewer: And does it change depending on the season or the time of the year?

Respondent: Oh, it changes in time, yeah, and probably longer in summer time. Summer time I'd go for a swim at the same time, but, yeah, a bit chilly now, and it's getting dark, the light is... the sun, you'd in before the sun comes up, so, yeah.

Interviewer: And if you'd wanted to, is there anything that would have helped you cycle or walk more to and from places?

Respondent: Yeah, I'd actually cycle to work if I could, but it's just so dangerous along the highway cycling, I find...

Interviewer: Would it have to be the main highway, or have you go a back road...

Respondent: Yeah. No, the main highway. There's no... there's a bit of a path but not all the way to [city].

Interviewer: And that's what about, did you say nine, ten kilometres?

Respondent: Yeah, probably eight kilometres, yeah.

Interviewer: Eight, OK. Yep.

Respondent: If there was a cycle path, yeah, I definitely would cycle to work. But it's just... at the moment, if I went earlier, it'd be dark, and then there's too many trucks and traffic around that time to really... to be confident to, yeah, on that highway.

Interviewer: Yeah. It's a very fast, busy highway.

Respondent: Yeah. Yeah, it's just narrow. There's sort of no lane way you get off really. Yeah.

Interviewer: OK. In the past two weeks, have you been active around the house or yard for that time inside or outside for ten minutes?

Respondent: Sure. I do lots of gardening; I like gardening, so, yeah. Yeah, gardening and active around the house. We're pulling out ceilings and doing renovations so...

Interviewer: Big job.

Respondent: So we do plastering at night time.

Interviewer: The joys of renovating. You look back on it and go, did we really do all that?

Respondent: Yeah.

Interviewer: But at the time you just keep on keeping on.

Respondent: Yeah. Anyway.

Interviewer: And thinking about those activities, how would you describe their intensity, steady, moderate, vigorous?

Respondent: Yeah, gardening's pretty steady, yeah. I do lots of digging and stuff like that, yeah.

Interviewer: And the renovating?

Respondent: Oh, the renovating, yeah, that's probably moderate too.

Interviewer: And how long would you have done those two activities for over the last couple of weeks?

Respondent: Gardening I probably did on the weekend, I probably did a couple of hours, two, three hours each day, Saturday and Sunday, and then the renovating we do probably, I don't know, an hour an night, something like that. It just depends.

Interviewer: And how often would you be doing those activities in the last fortnight?

Respondent: In the last fortnight, the gardening probably two days in the last fortnight of two or three hours. And the renovating, probably an hour over five days, or, you know, an hour in five days.

Interviewer: And who would have done those activities with?

Respondent: My husband, yeah.

Interviewer: And is that what you usually would do, or is it one off?

Respondent: No, we'd usually do that, yeah.

Interviewer: And does it change depending on the season or time of year?

Respondent: Oh, yeah. The weekend we were cutting lots of firewood, so, and stacking firewood, so we wouldn't be doing that in the heat of summer. Yeah, gardening certainly we do most weekends.

Interviewer: And if you'd wanted to, is there anything that would have helped you be more active around the home inside or outside?

Respondent: No, the weather was fine this weekend, so it was good. No, I don't think there's anything that would change.

Interviewer: Yep. It sounds like you're quite active as it is. And have you been physically active for at least ten minutes during your leisure or spare time? Now you might have been telling me about this with the cycling and walking anyway, but, is there anything else you wanted to...

Respondent: Yeah, cycling, yeah, no.

Interviewer: 'Cause these questions are just to capture, or parts of your life, it's so we don't leave things out, but they may not all be relevant.

Respondent: No. Occasionally, not in the last fortnight, but occasionally a group of us would go bushwalking for...

Interviewer: That's right. You mentioned that.

Respondent: ... a day or half a day or something, so, that might happen.

Interviewer: And when you go bushwalking, what sort of intensity of the activity would it be?

Respondent: It's constant and you probably do eight kilometres or so in the two to three hours, but we certainly don't climb mountains or anything.

Interviewer: So it's fairly moderate?

Respondent: It's pretty steady, moderate, yeah.

Interviewer: OK. And the duration of that? You've just...

Respondent: Yeah, half a day to a day some days.

Interviewer: And how often might you do it?

Respondent: Probably once a month.

Interviewer: And usually at weekends?

Respondent: Yeah.

Interviewer: Yep. And where would you do it, where would you go bushwalking?

Respondent: Usually in the local area. Like the last bushwalk we went is up the [x] River to [bushland] and along there, so, yeah. Local places, [x] Ranges and things like that.

Interviewer: So for those activities, like I'm just interested in how you're getting there, you would need to get in a car and drive there?

Respondent: Yeah, we would drive there, yep.

Interviewer: And would you be doing that activity alone or with friends or...

Respondent: No, with friends, yeah.

Interviewer: OK. Any reason why you would chose to do it with others?

Respondent: With others?

Interviewer: Uh huh.

Respondent: Oh just for company and leisure, yeah, enjoy the same things, yeah.

Interviewer: And does this change depending on the season or the time of the year?

Respondent: Yeah. We usually do it in the cooler months. Like other times we'd be on the beach together or kayaking or something in the summer time, yeah. So it's usually a winter time activity for us.

Interviewer: Yeah, where it's cool enough to do it.

Respondent: Yeah, yep.

Interviewer: And if you'd wanted to, is there anything that would have helped you be more active in your leisure or spare time?

Respondent: Just the weather, yeah.

Interviewer: Now the last section's about your physical environment, the environment around you that you live work and play in.

Respondent: Uh huh.

Interviewer: And when we talk about physical environment I'm meaning all the physical things that surround you, the roads, trees, houses, traffic, you know, open spaces, shops, all of those things. And some of these things are things that people living in urban areas have said are relevant to their physical activity, so we're interested in finding out whether they are relevant for you as well. Can you tell me a bit about

how easy or difficult it is to be physically active in your neighbourhood, or community?

Respondent: Yeah, down in [town] it's much easier than up at [town], 'cause we don't have any footpaths or anywhere to walk really up there. It's just a country road and it's quite dangerous to either walk or walk a dog or cycle or anything like that 'cause it's all too narrow. Even though there's no footpaths down at [town], and other community people have sort of said there's not footpaths here or street lighting that I find it much easier down there because you've got the beach right close by.

Interviewer: So you've got the alternative...

Respondent: Yeah.

Interviewer: ... of walking on the beach?

Respondent: Yeah.

Interviewer: Yeah. But...

Respondent: It's not, you know, it's not accommodated for people to be physically active I don't think, the area, 'cause there's no designated footpath or no designated bike tracks or, you know.

Interviewer: And that sort of vicinity, that location of [town], how long would it extend for that we're talking about of no footpaths?

Respondent: All the way to [town], five kilometres, and it's quite built up now, so, you know, there's...

Interviewer: Oh, OK, so it sort of seems like it extends most...

Respondent: Yeah.

Interviewer: ... of the way. Yep.

Respondent: Most if it's way, and...

Interviewer: And so you've got a main road with a shoulder basically...

Respondent: Yeah, yep.

Interviewer: ... and that usually drops off a bit so it's...

Respondent: That's it. And the train track, the train track's quite close by, so that's another sort of obstacle sort of thing.

Interviewer: Especially as there's enough... there's not like there's trains on it all the time...

Respondent: No.

Interviewer: ... but there's enough that you have to be aware.

Respondent: That you have to be aware of them, yeah.

Interviewer: And walking along train tracks isn't the easiest also.

Respondent: No.

Interviewer: That isn't easy either.

Respondent: Yeah.

Interviewer: OK. So I'm just going to go through a couple of features of...

Respondent: Yeah.

Interviewer: ... the physical environment, and first ups availability and accessibility. So if I was to ask you about places to be active in your area, what sorts of things would you tell me about, and you've told me a few things already about the beach and...

Respondent: Yeah, the parks are, you know, there's nice parks around, like in [city] there's [reserve] and certain nice areas you can go for picnics and things like that.

Interviewer: And that would be getting in your car and driving?

Respondent: Yeah.

Interviewer: Yep. Versus the beach where you can actually walk from your house, yep.

Respondent: Yeah. And I mean close by being the beach you can kayak and things like that, so that's good. And even the rivers too, you know, you can kayak. River and things like that, it's easy access to them. Fishing is a good... my husband does lots of fishing so he, you know.

Interviewer: So both of... you were saying for both of those activities it's fairly accessible, like often there's issues around private public access and for activities so, but there isn't for...

Respondent: No.

Interviewer: ... around [town] so...

Respondent: Not at all, no.

Interviewer: ... is some of it private land?

Respondent: No, no it's all...

Interviewer: No. OK. So it's all public that's by the river?

Respondent: Oh, up on the river, yeah, or where you can put a kayak in and things like that, I mean there's lots of houses going on to the river, but you can put a kayak in and kayak up the river, that's all the river belongs to whatever, everybody.

Interviewer: Yeah, yeah. But something that has come out across, actually across all the three areas, is that often people can't actually physically get access to the river, that all the areas that I've talked to women in...

Respondent: Oh, OK.

Interviewer: ... it was just... so I thought I'd just ask you, and that's what they were saying, was if there was actually some public access, some if it isn't accessible at all. You sort of are distanced at this...

Respondent: Oh, OK.

Interviewer: ...and it's often one of the, like for the [x] highlands and [town] that's their beautiful natural asset, one of those is the river, yeah. OK, so in [town] you've got easy access. That's good.

Respondent: Uh huh.

Interviewer: Now any sort of recreation things that are close by?

Respondent: Oh there's a hall not from our place but we haven't really investigated that. I know they do dancing and things like that, and I think that's once a month, but that's close by. Yeah, there's tennis courts too. I don't think they're very well used but they're close by.

Interviewer: And then you've got all sorts of things in [city].

Respondent: Uh huh.

Interviewer: There are a whole range of options as a community.

Respondent: Oh yeah. Yeah.

Interviewer: And [town], it sounds like it's got some things as well.

Respondent: Yeah.

Interviewer: So I was just interested in, it may not be what you... you don't actually use them yourself, but it's just...

Respondent: Yeah. But they're there, sure.

Interviewer: ... getting a broader idea of knowing what's in... accessible to you.

Respondent: Yeah. I mean there's golf courses and there's a sports centre in [town], yeah.

Interviewer: So, yes, you do have places to be active in your area, and are they convenient, are they easy to access would you say for you?

Respondent: Yeah, I think, so, yeah.

Interviewer: So does having places to be active influence whether you're active or not?

Respondent: Yeah. Yeah, sure. It gives you a good range of opportunities to, yeah.

Interviewer: And if you wanted to be more active, are there things or places that would help if they were available or more accessible?

Respondent: Probably more accessible as in cost wise I think. Things that, you know, some sports things are quite expensive. I used to do Pilates for a while and I haven't really found anywhere here, but, and I like that... the adult ed courses are quite expensive, I find they are anyway. So if costs were down they'd be, you know, I'd certainly...

Interviewer: More accessible.

Respondent: Yeah.

Interviewer: Absolutely. Actually it seems to be adult education things, a lot of things have taken a bit of a hike up I've noticed. You have to sort of think twice, whereas it was... sort of seemed to be a bit more within reach.

Respondent: Yeah, no I think, yeah, and just for the normal things that are not accessible to people, I don't think so, they're too costly.

Interviewer: Certainly another thing that's coming out is that women will tend... the women I've talked to will tend... their main sort of form of activity is walking, and certainly that's accessible and they've worked it out how it's accessible in each community there's, yeah, as you appreciate, you have to go to work your way out. So, yeah, I haven't talked to too many people where they've actually sort of got paid activities, yeah, so.

Respondent: Yeah.

Interviewer: Destinations is another one. If I were to ask you about places you could walk or cycle to from your home, what sorts of things would you tell me about? Things like shops, services, recreation facilities, friends, houses, those sorts of things.

Respondent: Yeah, those sorts of things. Shops, friends, yeah. Just to, yeah, I don't know. I try and cycle to yoga if I can. In the summer time, I don't do it this time of night 'cause by five o'clock it's dark.

Interviewer: And yoga is, just remind me?

Respondent: In [town].

Interviewer: In [town], that's right.

Respondent: Yeah. But, yeah, it's to meet friends or...

Interviewer: So does having destinations influence whether you're active or not?

Respondent: No, I don't think so, no.

Interviewer: You would be anyway.

Respondent: Yeah, yep.

Interviewer: It just provides you with some variety of how you're active.

Respondent: Yeah, that's right.

Interviewer: And if you wanted to be more active, would it help if there were more destinations within easy walking or riding distance?

Respondent: Oh, yeah, I'm sure it would be. Yeah, yeah. We went to the dog thing last weekend or the weekend before, you know, the RSPCA...

Interviewer: The Millions Paws.

Respondent: ...the Millions Paws but we would have walked there but we had to drive because it was in [town], so if there had have been one in [town] or [city], we might have walked there and then walked back, but, yeah.

Interviewer: And I wanted to ask you about neighbourhood design which is the sort of built on manmade or non-natural features of your area.

Respondent: Uh huh.

Interviewer: So if I ask you about those built features, what sorts of things would you tell me about?

Respondent: In your neighbourhood?

Interviewer: Uh huh.

Respondent: Oh, I don't know, like the community hall that's there, I think it's been there quite a while. Parks, they're not... I'm sorry, yeah, things that are built? Squash courts or something like that. There's some badminton things they play in that hall too.

Interviewer: And the lack of footpaths.

Respondent: That's right, the lack of footpaths, yeah.

Interviewer: What about lighting?

Respondent: Street lighting, yeah.

Interviewer: Yeah, lack of?

Respondent: Yeah.

Interviewer: So, yeah, I'm sort of interested in and what helps or hinders walkability, those sorts of things and cycling and, yep, yep.

Respondent: Yeah. I mean need good footpaths and access to footpaths and stuff, and, yeah, lighting, like especially now, you're walking in the dark sort of thing. Not that you want huge streetlights, but now with solar stuff surely they could have some proper lighting, you know, a bit better lighting than there is with councils.

Interviewer: And what about another aspect of walkability is being able to streets to connect, so rather than one...

Respondent: Oh, yeah, having the...

Interviewer: ... long strip as you were saying where you lived before...

Respondent: Yeah.

Interviewer: ... you've actually got things that connect.

Respondent: Yeah, that's a good idea, yeah.

Interviewer: Do you have that in [town]?

Respondent: No. No, I don't. But, yeah, it would be good. It makes it more interesting and, yeah.

Interviewer: So do you think... do the built features of your area influence whether you're physically active or not?

Respondent: They don't at the moment, but they could make me more physically active.

Interviewer: Exactly, yes. And if you wanted to be more active, are there built features in your area that would help?

Respondent: Yes, good footpaths and lighting, yeah. And nice, yeah, like you say, where you could go around then come back a different way or just something like that, yeah, to make it more interesting, yeah.

Interviewer: Yeah, I think that's really important. Yeah, I used to live in a rural area where you could sort of walk a bit. But friends lived in this one thoroughfare with this deep valley, and it was like, as soon as they moved there, I went, how are you going to go? 'Cause, OK, they could sort of climb up the hills and over people's properties, that was OK, but when they had a little baby it was a bit tricky to go walking along the main road.

Respondent: Yeah. Well, we've... at our property at [town], we actually made sort of walking tracks and different things so that we could go in to different spots and come back a different way and things like that, you know, like...

Interviewer: Wow. Did they actually take quite a bit of work to do?

Respondent: Oh, I mean they're not boards or anything, but we cleared bush and stuff...

Interviewer: Oh, OK, so you could actually walk through.

Respondent: ... so that we could, you know, yeah, that sort of thing.

Interviewer: Yeah, that's a good idea.

Respondent: We made tracks, yeah.

Interviewer: And that's the other one that women are saying is often in more country areas like [region] is a good one, an example is if you do have land you tend to walk on your property.

Respondent: Yeah, sure.

Interviewer: Road safety and...

Respondent: Yeah.

Interviewer: ... things like that.

Respondent: Yeah.

Interviewer: OK. So now the other part of it is the natural part of your environment, or the aesthetic qualities. So if I was to ask you about the aesthetic qualities or the attractiveness of your area, what sorts of things would you tell me about?

Respondent: Oh the bush and the environment, like the sea and that now where we are.

Interviewer: So you've got a fair amount of beautiful country around you...

Respondent: Yeah, we are very lucky.

Interviewer: ... sort of coast and bush is it?

Respondent: Yeah, yeah.

Interviewer: And a river as well?

Respondent: The river's further along, but, yeah. Yeah, it's very nice; it's a nice spot, yeah, but, yeah.

Interviewer: And do you think your local environment is aesthetically pleasing and attractive near your home?

Respondent: Yeah, it could be better, but, yeah.

Interviewer: And in what way could it be better?

Respondent: Oh, just, you know, where the railway track is, it's sort of been cleared and it's a bit ugly, you know, they could have some bushes and stuff like that, a bit of a garden there, but, yeah.

Interviewer: And do the aesthetics of your area influence whether you're physically active or not?

Respondent: Oh, yeah, of course it does, if you like to go out and have a look at gardens and things like that, yeah, for sure, yep.

Interviewer: Yeah. Something about being able to enjoy the beauty of the area isn't it?

Respondent: Yeah.

Interviewer: Now if you wanted to be more active, are there features of your area that might help if they were more aesthetically pleasing and attractive? You've mentioned the railway line.

Respondent: Yeah. The railway line and, I don't know, encouraging people with their gardens and things like that. I don't know, like the nature strips and stuff like that, less concrete or less asphalt.

Interviewer: That's a good point. Now the last area's just about safety, and if I were to ask you about safety in your area, what sorts of things would you tell me about?

Respondent: The road's pretty busy, yeah. It's not really a... we're in a quite a built up area and I think it should be about 50 kilometres, but it's 70 kilometres in front of our house. So I think that traffic wise it could be... because it's not a... even though it is a thoroughfare, it's not the main road, whereas the highway's at the back.

Interviewer: Yeah, someone... a couple of women have also made that point who live further along the... that that coast road, that it's for the narrowness and the fact there is a major highway...

Respondent: Yeah.

Interviewer: ... and it's fairly built up now, that, yeah, the...

Respondent: The speed zone.

Interviewer: ... the speed needs to come down.

Respondent: Mmm. I noticed even on this side of [town], it's 60 before you come in, it's 60, yeah, but it's 70 on the other... on the [town] side, so it probably should all be 50 really.

Interviewer: Yeah, there needs to be a lot more consistency with that.

Respondent: Yeah, I know, you know what it's like when you're driving, it goes from 80 to 60 and then it goes 100, like from our place to work, I go 110, 60, 100, 80, you're like, you know, what's sign, what you're in, but, yeah.

Interviewer: Let alone the other ones...

Respondent: And that's on the main highway.

Interviewer: There's also this little trap that I've worked out is that often they say the end of 60 but they don't actually say what it's meant...

Respondent: What the end of whatyeah.

Interviewer: ... which is quite frustrating yeah. It seems like there could be a bit more consistency in thinking about those sorts of things.

Respondent: For safety I think, yeah.

Interviewer: Safety, yeah, and as you said, it starts to encourage people to be active if, yeah.

Respondent: Yeah.

Interviewer: And I think we'd all be... and I think this study's made me much more aware too in rural areas that that may be the only place that people can walk along, and that I'm sure if we all knew that, that you're actually trying to decrease the speed to encourage people to be active in their own areas, people would understand that a lot more.

Respondent: Yeah.

Interviewer: It's like with school kids, everybody understands that zone...

Respondent: Yeah, fortunately, yeah.

Interviewer: ... there's a reason for it, so, yeah. So it doesn't sound, as you said, it doesn't seem to be too hard but it would need a bit of education.

Respondent: Yeah.

Interviewer: Yeah. So does personal safety influence whether you're physically active or not?

Respondent: Oh, yeah, for sure, yeah.

Interviewer: OK. So tell me about that.

Respondent: Yeah, I mean I'd ride a bike more if there was bike tracks. I'd probably take the dogs to... I always walk my dogs but I only take them to certain things because if it's too close to traffic or things like that, it's too dangerous.

Interviewer: Mmm. So particularly road safety does influence you.

Respondent: Yeah.

Interviewer: But personal safety in terms of, well, that's the other side of if you're not feeling safe with traffic, but things like are there any areas that you wouldn't walk at night and at night and stuff like that?

Respondent: Yeah, yeah, for sure.

Interviewer: And part of that is sometimes is 'cause of the lighting...

Respondent: The lighting and things like, yeah.

Interviewer: ...and you can't see. But is it a fairly safe community that you live in?

Respondent: Oh, yeah, I think it is, yeah, very safe.

Interviewer: So it's more those things in the environment that would help.

Respondent: Yeah.

Interviewer: OK. And if you wanted to be more active, are there any personal or road safety issues that if addressed might help you to be more active?

Respondent: Yeah, put some bike paths in.

Interviewer: Everyone's been saying that.

Respondent: Yeah, it'd be really good if there was bike tracks. Yeah, people would be much keener to cycle to work and things like that.

Interviewer: Yeah. Well, I know when I moved to a rural area I gave up riding 'cause it's...

Respondent: Yeah.

Interviewer: ...the main road and traffic don't...

Respondent: No.

Interviewer: And then like I was just talking to a woman this morning and she was saying part of it then is when you get back on a bike which we both are, is getting that confidence and you don't feel quite as ofay...

Respondent: Yeah.

Interviewer: ... about dealing with all this road traffic...

Respondent: Yeah.

Interviewer: ... especially when we're going fast and narrow roads.

Respondent: Yeah.

Interviewer: And nowhere to go.

Respondent: Yeah, the older you are it takes longer to heal, that's what I think.

Interviewer: Exactly.

Respondent: If I've got a broken hip or something or...

Interviewer: Exactly, you don't bounce as well you used to.

Respondent: No.

Interviewer: No, absolutely not.

Respondent: That's what I think.

Interviewer: OK. Well, thank you for that.

Respondent: You might keep yourself fit and healthy and eat the right things and do the things, but if you get hit by a something, bones just don't bounce back to what they might have done when you were 18.

Interviewer: No, no. And it takes you then so long...

Respondent: It'd be awful to have that chronic...

Interviewer: ... to get back in to being able to...

Respondent: ... chronic pain and chronic something or other to live with.

Interviewer: Yes, so it's best to avoid it.

Respondent: Yeah, or don't put yourself in to it, yeah.

Interviewer: No, don't put yourself in those situations. Yeah, absolutely. OK. Just before we finish up, is there anything else that you'd like to tell me about where you live and your activity and the environment?

Respondent: No, I think maybe you should include animals in your thing, 'cause I really walk because I've got dogs...

Interviewer: Yeah, me too.

Respondent: ... and they are the things that keep you active I think.

Interviewer: Yeah. Yeah, me too. I've got a [dog breed], so every morning...

Respondent: Yeah.

Interviewer: ... and when I'm not working during the week, I'm part time, it's two walks a day. She's great for it.

Respondent: That's right. They make you, yeah.

Interviewer: Yep. And you get to meet all sorts, you know, lots of people and...

Respondent: Yeah.

Interviewer: ... and it's always good fun and they always enjoy themselves don't they?

Respondent: Yeah.

Interviewer: Yeah, I think, yeah, dog walking's a very good one. Yep, so what are your two dogs?

Respondent: Well, my two dogs are dogs that my children that have left behind that, but one's a [dog breed] thing, and the other ones a, well, it's from the pound. It's got part [dog breed] and part [dog breed] in it.

Interviewer: So one that's landed on...

Respondent: It's very bouncy.

Interviewer: Yes, yes.

Respondent: You know, pick me, pick me sort of thing.

Interviewer: So it landed on all four paws when it got to come to live with you.

Respondent: Yeah. No, it's a nice dog, yeah.

Interviewer: OK. Thank you very, very much.

Interviewer: So I thought we might start by talking about where you're living. Can you tell me a bit about what it's like to actually live in [town], your likes and your dislikes?

Respondent: Well we chose [town] because it's a small town. We've lived in small mining towns all our married life on the [x] Coast and then in [state] and we just like the small town feel that you know people. [town] was also close enough to [city] and [city] the larger centres for the kids to be doing other activities and we knew there was a lot of activities in and around [town] and [town] as well for the kids. And living near the sea is really important to my husband as well. [town] was just the ideal… it was a good distance from my parents not too

close, not too far, they're in [city]. So it just seemed like a good place.

Interviewer: So how far are you from [city]… [town]…?

Respondent: It is about 20 minutes from [city].

Interviewer: And [town] about...?

Respondent: [town] ten minutes and [city] about 25 or 30 minutes depending on the traffic.

Interviewer: What type of property do you live in is it house and house block in [town]?

Respondent: Yeah we have got some land we have got 23 acres and a house. We didn't really want that but the land came with the old house and we built on that land and took the old house down…

Interviewer: So you are in the centre of [town] itself?

Respondent: No we're near where the flower gardens go along the train tracks. So we're well within the [town] boundary, close to the dog beach.

Interviewer: OK so you're more on the outskirts rather than right in the middle of [town] itself, is that right?

Respondent: Yeah as I said we're still well and truly within the [town] boundary where the flower gardens go along by the train track. So we've got lots of neighbours [laughter].

Interviewer: What's the length of time you have been living there?

Respondent: We've been on that land and that house for about 11 years now, and we lived for nearly a year in a rental place before we bought.

Interviewer: And that was in [town] too?

Respondent: Yes.

Interviewer: And your reasons for living there?

Respondent: As I've said small town, close to the sea, close to… within walking distance of town, close to the larger centres.

Interviewer: So in research with women living in urban areas people often refer to the term neighbourhood. And I'm interested in understanding what the term neighbourhood means to women living in rural areas and if it's relevant.

Respondent: Oh I think it's really important. We know most of the people in our immediate vicinity, all our neighbours. As I said we've got a lot of neighbours because our land bounds... we have about… boundary fences with about 16 neighbours and we know most of those. We have… there is a few people, good friends of ours that live not very far away on the same street, we regularly get together and have dinners and BBQs and things like that. It's lovely… the neighbourhood feel you are walking along and you can say hello and you know just about everyone you pass and it is really nice. You know that people will look out for you. Our dog wanders... well she was wandering and we'd have people bringing her back and saying, "Oh we saw [dog] up at such and such and got her off the road" [laughs]. The concept that you know people and they will look out for you and your family.

Interviewer: That's a bit of a friendly community that one...

Respondent: Yes.

Interviewer: If I asked you to put a distance around it, what sort of distance would you put around your idea of neighbourhood? Would it be a couple of kilometres?

Respondent: Yeah it probably would be.

Interviewer: Sort of radius?

Respondent: Yes probably a couple of kilometres would be the maximum I should imagine.

Interviewer: What about the word community what does that mean to you?

Respondent: I suppose it's the same thing as well, just that sense that everybody… people know each other maybe not intimately but they know each other by sight, and everyone cares about the area they live in, cares about the people, look out for each other.

Interviewer: And that's about the same sort of distance?

Respondent: No community would be bigger I think.

Interviewer: OK if that needed to have a distance around that how big would you make that?

Respondent: Well I would make it the whole of [town].

Interviewer: So that would be roughly double that size?

Respondent: Yes. That would include the schools and the sporting organisations etcetera as well.

Interviewer: Now I wanted to talk a bit about physical activity and what that meant to you, as I said it's a very broad concept and everybody has ideas about it. So what does it mean to you physical activity?

Respondent: Getting to the gym regularly, going for walks, keeping active, not just sitting down at the computer or watching the TV.

Interviewer: Do you consider yourself physically active?

Respondent: Prior to this shoulder injury yes. Not as physically active I would like, as I used to be. When we were building the house we were doing a lot of physical activity.

Interviewer: In terms of building stuff?

Respondent: Building and digging and painting and all of those things. I go to the gym a couple of times a week; three if I can. I walk the dog, try and do some gardening. Not as much as I should though.

Interviewer: And where does physical activity fit into your life? Do you consider it of interest or is it a priority a high priority or no interest at all?

Respondent: Well it's a priority for me but it does have to be squeezed in but I've decided I have to go to the gym given my age, so I will either call in after work or make an effort and go on the days that I don't work.

Interviewer: And where is it the gym for you?

Respondent: The gym is in [city]. So I have to drive back to [city].

Interviewer: So is that a separate trip for you?

Respondent: Yes it would be. As I said occasionally I try and call in at least one day after work on the way home from work so you are doing that quite late.

Interviewer: So you are working in...

Respondent: [city]. And then I would make separate trips. It is only ten minutes' drive because it's on the outskirts of [city] thank goodness. If I had to actually drive all the way into [city] I probably wouldn't be as inclined to do it. With this shoulder injury I'm feeling very bad that I'm sitting around doing nothing.

Interviewer: It really does debilitate you.

Respondent: Even going for a walk with the dog it hurts it every time the foot hits the ground it reverberates up to the shoulder. So I'm thinking oh this is great. So physical activity is important to me especially as I'm getting older I'm realising use it or lose it.

Interviewer: So it's very much of your daily life in that way.

Respondent: Uh-huh.

Interviewer: Now I wanted to have a look a bit more about physical activity. In our research when we think about physical activity we think of any activity that last for at least ten minutes i.e. that is the health benefit effect and it causes your body to work harder, get warmer,

heart rate up and a bit of puffing and huffing maybe. So I wanted to look at the last couple of weeks in your life and just look at different parts of your life mainly because we don't want to leave out bits.

Respondent: Go back... like the shoulder was really bad on Friday so I will back past that.

Interviewer: Yes given the circumstances. In the past two weeks have you been active at all at work for at least ten minutes of a time.

Respondent: At work no.

Interviewer: If that's the case why is that?

Respondent: Because it's a sedentary job.

Interviewer: It's a sitting at a computer is it?

Respondent: Yes sitting at a computer and working with clients so not a lot.

Interviewer: Do you do that part-time or full time?

Respondent: Part-time, not a lot of work. I do walk up… I have started walking up the stairs, like we're on the fifth floor and I have started making sure I walk up the stairs at least once a day when I'm at work – five flights.

Interviewer: And if you'd wanted to, is there anything that would have helped you be more active at work, like you were just saying you try to walk up the stairs?

Respondent: Yes I just don't know how you could combine it with the work we do. No I can't see how, unless there was a set time where we all did some aerobics for [laughter] ten minutes.

Interviewer: So as a class or something. So that there would be a few people doing something or rather in your break or whatever breaks you have.

Alright, in the past two weeks have you walked or cycled for at least ten minutes at a time to get to and from places?

Respondent: Yes. Walked...

Interviewer: So walked or cycled?

Respondent: Walked.

Interviewer: Can you tell me about what you did?

Respondent: Walked the dog down to the beach and back.

Interviewer: And the intensity of that would that be steady or moderate or vigorous?

Respondent: Steady probably. So what do you want to do about gym sessions?

Interviewer: That you can either mention that here or leisure or spare time I will also ask you about. As I said I'll ask you different parts of your life and as long as we cover the activities I don't really mind where they get talked about [laughs]. The best way we try and make sure we don't leave things out.

Respondent: I would have walked the dog also down to the shop to get some groceries that would have been in the last two weeks

Interviewer: And that would have been pretty steady as well?

Respondent: Yes.

Interviewer: And how long would you've walked the dog for?

Respondent: By the time you go there it's about half an hour there and back.

Interviewer: So both of those activities would be about half an hour?

Respondent: Yep.

Interviewer: And how often would you have done that?

Respondent: Two weeks that I'm thinking of probably about four or five times.

Interviewer: What time of the day would you have done that?

Respondent: Gosh it depends. If it's a weekend it could've been… it probably is afternoon. If it's a day off it would be in the morning.

Interviewer: OK you've talked to me about walking the dog at the beach and also going to the shops. Why did you do it there what attracted you to do it…?

Respondent: Oh we go to the beach because the dog just loves the beach and it's a dog beach…

Interviewer: Oh you have got a dog beach?

Respondent: Yes good place for her to meet other dogs.

Interviewer: I'm mapping all the dog beaches I've got a [dog] who loves swimming [laughs].

Respondent: Well this one's a lovely dog beach and she loves the beach. And going to the shops why did I do that because [laughter] I needed something at the shops.

Interviewer: Were you alone or with others?

Respondent: I might have walked with my husband... oh no I walked with my daughter… one of those times would have been with my daughter.

Interviewer: So a couple of those times…

Respondent: Two, both daughters separately yes.

Interviewer: It would have been with other members of the family...

Respondent: Yeah and there would have been a couple of times when it was just the dog and me, so it's probably more than four, over two weeks it's probably about five.

Interviewer: And is that what you usually do or was it one off?

Respondent: No that's the usual thing. If my husband's around and he's not busy we'll go for a walk together and take the dog together.

Interviewer: And does it change depending on the season or the time of year?

Respondent: Uh-huh probably yes because we don't tend to walk if it's really, really windy or rainy.

Interviewer: That [laughter] tends to be winter.

Respondent: Well actually spring.

Interviewer: Spring too.

Respondent: Spring here is windy and horrible so I dislike going out walking when it's really windy, and I'll get the kids to do it. Can someone please walk the dog?

Interviewer: [Laughs]. If you'd wanted to is there anything that would have helped you be more active with walking…?

Respondent: Yeah not taking the dog she slows me up, she's getting a bit old and she wants to also sniff at things. So you don't actually get a really good fast walk with her but then you can't leave her behind.

Interviewer: She likes her walk. OK. In the past two weeks have you been active around the house or yard so at home inside and outside?

Respondent: Well vacuuming doing general household duties yes. Yes I was doing a bit of gardening it would have been about two weeks ago doing some pruning, cutting old kangaroo paws off and pulling up weeds, so yes.

Interviewer: And could you tell me about the intensity of that, would that be steady, moderate or vigorous?

Respondent: Probably moderate for the gardening because I was doing a lot of bending and pulling.

Interviewer: And how long did you say you would have done that for?

Respondent: Probably about an hour.

Interviewer: And how often would you have done it in that time?

Respondent: In that two weeks only the one day.

Interviewer: And when did you do it, during the week, weekend?

Respondent: Weekend.

Interviewer: During the day?

Respondent: Yes. And the vacuuming and all that kind of stuff would have been a few times over a two week period, probably four times maybe.

Interviewer: And would you call that steady, moderate or vigorous?

Respondent: Probably steady.

Interviewer: What time of day would you have done that?

Respondent: Gosh don't know.

Interviewer: Whenever you've got the time and the energy.

Respondent: It wouldn't have been at night not in the evening.

Interviewer: I must admit I haven't got into housework at night either. I like to sit down at that time and you're usually too busy cooking than doing other things.

Respondent: Yeah exactly.

Interviewer: And who would you have done that with is it alone or with others?

Respondent: The gardening was alone my husband was doing some gardening elsewhere and then the vacuuming would have just been me.

Interviewer: And is that what you usually do?

Respondent: Yes.

Interviewer: Does it change depending on the season or the time of year?

Respondent: I suppose gardening depends on the season and time of year.

Interviewer: There is more to do sometimes than the other.

Respondent: Spring time there would be more of us out there we would be possibly dragging the kids to do a little bit.

Interviewer: If you wanted to is there anything that would have helped you be more active at home either inside or outside?

Respondent: Oh the shoulder problem because I was having to do it with my left hand, the shoulder wasn't that bad then but if I hadn't had my injury yes it would have made it easier as well.

Interviewer: Have you been at least physically active for at least ten minutes during your leisure or spare time?

Respondent: Yes.

Interviewer: And can you tell me what you did?

Respondent: That's going to the gym. Those two weeks that I'm thinking about I went to the gym three times each of those weeks and that's a circuit. So there is a three to five minutes and I've been trying to do at least four to five minute warm up on an exercise bike. And then machines interspersed alternating with 45 seconds of some kind of activity to keep your heart rate up. So it takes probably about 35 minutes to do the whole circuit and then a warm down afterwards, a stretch afterwards. So I would have done in those two weeks I would have gone to the gym three times each week, so six times.

Interviewer: So the duration would have been about an hour by the time you have sort of warmed up and...?

Respondent: Probably about 40 minutes.

Interviewer: And the intensity?

Respondent: Yeah a couple of those sessions were very intense ones because the gym lady was making us all keep going, but I suppose it would average out to be moderate with a couple that were...

Interviewer: With some that would be vigorous.

Respondent: Yes. Oh actually probably more… the majority of them would have been vigorous.

Interviewer: OK. When would you have done those six sessions?

Respondent: Well two of them were definitely after work coming home from work so 5.00 o'clock in the evening or 5.30 by the time I get there and the other… four were on days off so they were in the morning; there was a Sunday morning I went. All of them would have been in the morning I would have gone over in the morning.

Interviewer: Where is in [city]?

Respondent: Yes on the eastern side of [city].

Interviewer: Why there?

Respondent: That's where it is.

Interviewer: That's where it is. Was there anything that attracted you to that particular gym?

Respondent: The fact that it was a women's only one, so you're not surrounded by men huffing and puffing and trying to increase their muscles and checking you [laughs] out. There's a wide range of people that go so there's a lot of women that are much older than me, all shapes and sizes. It's a nice blend of short bursts of machine work interspersed with the aerobic work so it's not boring, and you've got the enthusiastic instructors there to keep you going if you're needing a bit of motivation or some ideas. You don't have to think too much of things to do yourself.

Interviewer: And that's really useful in what actually works for you in knowing that, that's good to know.

Respondent: You feel given they said you can do a whole workout in 29 minutes, I mean that doesn't include the warm up and the stretch down but it does… it makes it feel like it is achievable. Yes I can do that I can fit it in. You're probably there a bit longer, well as I said I am but you still think well that's achievable that can fit in.

Interviewer: That's a very good point. And who with?

Respondent: Most of the time it's on my own.

Interviewer: And is that what you usually do?

Respondent: Oh I used to go with a friend but she's no longer going to that gym because she works in a different town now, so for all of this year that's what it's been, but you get to know people there.

Interviewer: In that sense yeah. And also is it what you usually do or is it one off?

Respondent: No that's a usual.

Interviewer: And does it depend on the time of year or the season?

Respondent: No.

Interviewer: And if you wanted to is there anything that would have helped you be more active in your leisure or spare time, like more time or someone to be active with?

Respondent: Just more time I suppose. If I didn't have to race home for taking kids to sporting things in the evenings I'd probably call in on another

day. But as far as the rest of my leisure time just having the time to do it [laughs].

Interviewer: Yeah it's always a balance isn't it?

Respondent: Uh-huh.

Interviewer: So we've talked a bit about where you live and your physical activity, now I just want to talk about the other part of it which is the physical environment that you live, work and play in. When I talk about physical environment I mean physical things that surround you. So that can be absolutely anything from roads and trees and houses and parks. And some of these things are things that people living in urban areas have said might be important to helping with physical activity. So I wanted to ask if they're relevant for you.

So first up I just wanted to ask you can you tell me a bit about how easy or difficult it is to be physically active in your own area?

Respondent: It's fairly easy. Once we get down our steep drive we've got flat areas to walk in. It would actually be good if we could go in the other direction because there's no walking track going east once the footpath ends that's it. So you do tend to go the same way all the time so it would be nice to go on some different walks. And the fact I suppose [town] is hilly so you do tend to try and stick to the foreshore so you don't [laughs] have to go up and down the hills. Can't think of anything else.

A cycle path would be wonderful between [town] and [town], not that I've ridden a bike for ages but it would be nice.

Interviewer: Yes you might contemplate it though if it was there.

Respondent: Yes.

Interviewer: Now availability and accessibility of places to be active. If I were to ask you about in your own area, in your neighbourhood what sorts of things would you tell me about?

Respondent: Oh it would be good to have the gym here and then I would go...

Interviewer: You don’t have a gym in [town]?

Respondent: No.

Interviewer: Obviously you’ve talked about the beach, what about recreation facilities and open spaces and those sorts of things are they available in [town]?

Respondent: [town] is pretty lucky we have got the beaches and the park and then there is the recreation oval if you are wanting to do things like that. Yeah [town] is pretty lucky for a little town.

Interviewer: And accessibility are the places convenient?

Respondent: Well as I said I have to go to the [city] for the gym or [town] there is another one there but it’s not quite the same that makes... yeah I would probably do more if it was in [town].

Interviewer: Does having or not having places to be active influence whether you are active or not?

Respondent: Oh I’m sure it influences it. Even the fact that you are always walking the same path sometimes I think oh I can’t be bothered but the dog needs to be walked.

Interviewer: It would be nice to have another option.

Respondent: Yes.

Interviewer: And if you wanted to be more active are there things or places that would help if they were available or more accessible? And you have already told me about cycle paths and another sort of walk option.

Respondent: Or a walking track that goes east.

Interviewer: And ideally a gym would be nice.

Respondent: I shouldn’t complain only ten minutes drive really. If you are in the city you would have to drive more than that.

Interviewer: Get caught in traffic that far. So destinations, if I were to ask you about places that you could walk to from your home, what things would you tell me about and you’ve talked a bit about that already.

Respondent: Beaches, town.

Interviewer: So you’ve talked about shops and the beach and neighbours and friends around. Does having a destination influence whether you’re active or not?

Respondent: No probably not.

Interviewer: And if you want to be more active would it help if there were more destinations within easy walking distance?

Respondent: No most of the time the walk itself is... it's not a means to an end it's the end itself.

Interviewer: OK. I just want to talk about neighbourhood design which is the built features or man-made or the ones that aren't natural. What sorts of things would you tell me about if I asked about the built features of your neighbourhood, for instance footpaths and lighting those sorts of things?

Respondent: Well the lighting actually ends right near our driveway so there is nothing more if you go east anyway, so even though there is a bit of a footpath going past all the houses going further east there is no lighting there, no street lighting. And the street lighting that ends at our place isn't wonderful either so that could certainly be improved. And the footpaths as I said could be a walking track further on. Another thing that we find frustrating is so often the footpaths are narrow and they have that stupid little bit of grass and you think it's not even big enough for three people to walk, you don't need that little verge. Anything else?

Interviewer: So walk ability of your neighbourhood in terms of whether things like streets connect together and you can sort of walk.

Respondent: Yes. [town] knowing [town] is quite hilly once you get a little bit further away from the beach it does tend to be very hilly but you can certainly get anywhere you want to if you're prepared to walk up those hills.

Interviewer: Well do they have areas where you can actually walk or is it walking alongside the road?

Respondent: No most of the places around [town] there are footpaths…

Interviewer: They do have them. Do these built features in your area influence whether you're active or not?

Respondent: I suppose they do because I wouldn't be inclined to walk. If we ever go east I stop walking once the footpath ends because I don't want to be walking on the road, so I suppose yeah they would.

Interviewer: And that way it does. If you wanted to be more active are there any built features in your area that would help?

Respondent: Well I would like to get into cycling as a means of getting to somewhere and I think there could be more cycling tracks all around the [x] coast. Even between... this is probably nothing I would use but between [city] and [town] there is a bit of a cycle path but it ends and then anyone else who is cycling has to go on the road. There are really wide areas between the white line and the actual edge of the bitumen, they just need to sweep the little stones off and paint a bike thing and there would be enough...

Interviewer: You could have a cycle lane.

Respondent: Yes almost all the way between [town] and [city] without actually having to build one. They just need to sweep all the stones away and designate that area, and there might be a few little areas they might have to seal but basically as I drive the whole time I keep thinking the cyclists are on the road but they don't have to be because there is so much other space there, but that's where the

little stones are and things. I keep thinking one day I'll write to the council and say why don't you get a road sweeper out there and sweep it all off and paint a bike sign on it and make that the designated cycle way.

Interviewer: A fairly easy option.

Respondent: Well look at what [city] done in the centre of the city, they've made all those cycle ways and it wasn't hard they didn't have to build anything extra.

Interviewer: It was all there.

Respondent: Yes.

Interviewer: Good point.

Respondent: Not that I'd probably use it at my age but there are a lot of people that would maybe my kids would.

Interviewer: Now the aesthetics or the natural qualities of your environment. If I were to ask you about the aesthetic qualities or the attractiveness of your area what sorts of things would you tell me about?

Respondent: The sea - just overlooking the sea so close to the sea.

Interviewer: You actually overlook the sea?

Respondent: Yes we're only 100 metres from the water.

Interviewer: So you can just gaze [laughs].

Respondent: Yes. And the fact that we've also got that little bit of land we've got greenery around us.

Interviewer: You've got trees and some bush, very beautiful. Do you think your local environment is aesthetically pleasing and attractive near your home?

Respondent: Yes.

Interviewer: Do the aesthetics of your area influence whether you're physically active or not?

Respondent: Oh I suppose so because we want to walk down to the beach because it's so close.

Interviewer: It draws you down there.

Respondent: Yep.

Interviewer: And if you wanted to be more active are there features of your area that might help if they were more aesthetically pleasing and attractive?

Respondent: No probably not…

Interviewer: It's all pretty beautiful as it is.

Respondent: Uh-huh.

Interviewer: And just one last area is on safety. If I were to ask you about safety in your area what sorts of things would you tell me about?

Respondent: Well as I said the street lighting especially in winter if you're wanting to go for a walk after work.

Interviewer: You can't see and be seen?

Respondent: Uh-huh. And if you wanted to bike ride for instance as I said between [town] and [town]...

Interviewer: That's the road safety.

Respondent: I won't let my kids do that. I know other people do but you're on a very narrow winding road with cars zooming past you.

Interviewer: Do you think road safety influences whether you're physically active or not?

Respondent: Well it certainly influences whether I choose to go bike riding. I do have a bike but it hasn't been used for ages and probably more because... apart from going down to the shop and back there is not many others areas I would feel safe to go.

Interviewer: Yeah I think that's a really important one, it's has come up, women have generally said that road safety is one of those important things.

Respondent: And as you are getting older it would lovely to go for a little bike ride down to the shop with your basket in front – it would be lovely.

Interviewer: Absolutely. Does personal safety influence whether you are physically active or not?

Respondent: No probably not although I wouldn't go walking at dusk, at night.

Interviewer: And that would be because you just can't see?

Respondent: Yes I don't feel unsafe in [town].

Interviewer: Because it's a fairly safe area isn't it?

Respondent: Uh-huh.

Interviewer: And if you wanted to be more active are there personal or road safety issues that if they were addressed might help you be more active?

Respondent: Yeah just what I've mentioned about the cycle ways.

Interviewer: Yes absolutely. OK just to finish. If there's anything else you'd like to tell us about where you live, your physical activity or your environment that you live in that we haven't covered today.

Respondent: No I think you've covered most of it.

Interviewer: Thank you very much.

Respondent: Can't think of anything else.

Interviewer: Thank you.

Interviewer: So first of all I wanted to look at a bit about the place where you live, OK? And so I wanted to actually have – ask you if you can tell me about what it's like to live around here? The likes, dislikes.

Respondent: I've lived in [town] full time now for 30 years.

Interviewer: Right.

Respondent: I was – I'm a farm girl so we come off a…

Interviewer: Oh OK.

Respondent: …farm but I left quite early at 15, went to the mainland for a couple of years and then come back here and brought my children up here. I love the [x] coast. I think we have the best of absolutely everything…

Interviewer: It's very beautiful.

Respondent: … here and it depends on, you know, for the lifestyle, for the safety factors, for the - just the general environment, I love it. But then I don't like to travel so I'm happy to travel to go to things and to go away and I do a lot of travelling around the state, I do a lot of travelling interstate and have travelled overseas a bit. I'm always happy to come home so that mine will be a biased point of view. I understand that it can be frustrating when you've got mainland visitors over here and [state] shuts down early, the [x] coast shuts down early. But I think we make up for it.

Interviewer: And are you living in [town] itself?

Respondent: Yes, in the centre of town.

Interviewer: Is that on a house and house block?

Respondent: Yes.

Interviewer: So the length of time you've been living in [town] itself?

Respondent: [x] years.

Interviewer: OK right, so ever since you came off the farm.

Respondent: Since I come back from living in the mainland for a couple of years, yep.

Interviewer: And your reasons for living here?

Respondent: Family, it's home. I was bought up here so all of my family were here, yeah, so that's where we came back to, my late husband and I. So it was more just, "This is home" – have lived, you know, had spasmodic times before that…

[Interrupted by phone]

Interviewer: So in the research with women a lot of it's been done in urban areas and cities and people often refer to their local neighbourhood.

Respondent: Uh-huh.

Interviewer: And we're interested in getting an understanding of what the term neighbourhood means to women who live in rural areas. So what does the term neighbourhood mean for you?

Respondent: Just [town] the town. That would probably – yeah, I think the whole of [town] is my neighbourhood not just necessarily the – as in I live just over the bridge so it's neither west, it's centre, it's quite central.

Interviewer: Yes. So is there a distance though? Because [town] – a few kilometers?

Respondent: Yeah, look not out there - the neighborhood's probably – no, nowhere outside the immediate populace – populated area.

Interviewer: A few kilometers in each direction? A couple of kilometers?

Respondent: But that's probably because coming off the farm the neighborhood was a few kilometers as well. Your closest neighbor on the next farm at times was a decent hike. So I don't know whether that's – the neighborhood is just not necessarily the immediate streets around me.

Interviewer: It's a bit bigger than that for you.

Respondent: Mmm.

Interviewer: What about the word 'community'? What does that word mean to you?

Respondent: Again probably broader. Community to me is the whole, sometimes the – would be a bit broader than just [town] to me but that's because I work outside of [town] and I work in health so sometimes it's the whole – the community as in, yeah – so broader to me.

Interviewer: So that's sort of linked to your work networks and…

Respondent: Yeah. I've never really actually even considered that as a question so…

Interviewer: Yeah, it's interesting to consider both, whether they're the same...

Respondent: Yeah, it's whether we're talking about…

Interviewer: …or different.

Respondent: … the living environment community or the wider – I often need to talk about the health community so that's quite broad or sporting community and that encompasses the coast but if we're talking just about…

Interviewer: … living community – that was the other category.

Respondent: … living community – so that would be – that's my town.

Interviewer: Yep, OK.

Respondent: So if I was to say that then yes, probably the neighborhood is probably a little bit smaller but the community's the whole of the town if I had to differentiate between the two, now that I've been asked the question. So I was probably thinking neighborhood as in community.

Interviewer: OK, so community would be the whole of [town] and the few kilometers in each direction.

Respondent: Yeah and the neighborhood is probably closer in a little bit.

Interviewer: So what sort of distance would that be?

Respondent: I suppose if I was to say I lived close to the town, so that's in my neighbourhood, it's not far to go to the supermarket or to go – and I've got a local shop but it shuts early so I probably haven't got one

close in my neighbourhood. I've got to go in a car. So probably neighbourhood is where I wouldn't have to go in a car to somewhere.

Interviewer: Yep, and that would be what, walking and…

Respondent: Yeah, walking.

Interviewer: How long?

Respondent: A couple of Ks, a couple of Ks, yeah.

Interviewer: That's good, that's clear, OK. It's interesting when you start to – they are things that you start to think about when you start – and you go, "Oh yeah, OK". So I'm now going to ask you a few questions around physical activity and just want to talk to you about firstly what physical activity – what the term actually means to you because it can mean lots of different things to different people. So what does it mean to you?

Respondent: Not necessarily sport, just being - actually being able to get around so if I…

Interviewer: Day to day getting around?

Respondent: Just any physical activity and that's my health background cause just actually the fact that somebody can even just go for a stroll or a walk or get around unaided, to me that's a level of physical activity because I've met people whose inability to get from the lounge room to the kitchen impedes their life.

Interviewer: Very good point.

Respondent: That would be my take, that's just my reasoning on it and then there's all sorts of levels of that and I'm probably in the spot where I now have a grade 3 sprain in my ankle which has actually impeded me from doing a few things so – but compared to some people it's not an impedance at all. I just can't do anything high impact but it doesn't stop, it doesn't impact or influence my life in any way.

Interviewer: Do you consider yourself to be physically active?

Respondent: Yes.

Interviewer: And why?

Respondent: I go to the gym twice a week. I don't get to probably do as much as I like because my work means that I need to sit in a car and travel a lot. But I also find that as much as I can, especially when I'm tending to – when I'm working in [city], I will think about it and if I can walk to an appointment, I will very much try to do that. Probably more than I would up here because parking's an issue so that probably has an influence, it certainly does because it's more of a hassle sometimes I think that I need to park just as far away from a place as it takes me to walk, especially when they're in the CBD area. I live in a region that doesn't have a good public transport system so I've never really had to have that as a – never really considered that.

Interviewer: Where does physical activity fit in your life? Is it high priority or just something that is something you do or?

Respondent: It's just something I do. It's not as high as it should be but I'm certainly making more of an effort now.

Interviewer: Things change from time to time in people's lives.

Respondent: I've always been a shift worker.

Interviewer: Oh, OK.

Respondent: So it's been very difficult for routine things which is why I haven't participated in team sports for 20 years because it was too difficult when you were a shift worker and you work weekends and that you can't always go to training, you can miss a number of weeks in a row if that's your weekend on so team sport is difficult for that and just regularly being able to go to things. I've worked four on four off or I've worked other shifts with [job], it can be sometimes difficult to be consistent and it doesn't take you long when you're not consistent to get out of the routine of something.

Interviewer: Absolutely, yeah.

Respondent: That's, probably more than anything, would have had more of an impact on things.

Interviewer: That's important. Now I'm going to ask you a series of questions around physical activity and in our research when we think about physical activity we think of it as any activity that lasts for at least 10 minutes or getting the body to work harder than normal or your heart rate going up or a bit of huffing and puffing and warming up. So thinking about that definition during the last two weeks, OK? Now in the past two weeks have you been active at all at work for at least 10 minutes at a time?

Respondent: Yes.

Interviewer: Can you tell me about what you did?

Respondent: Walk and cardiac compressions.

Interviewer: Right that's fairly vigorous isn't it? So that was the question – how often would you have been doing that?

Respondent: Prior to - if I take prior – because from the last – from January I've gone to an office more environment, but prior to that it was spasmodic so you were maybe fairly active but it always wasn't physically active but you were on the go and you had spasmodic things. So no, it wasn't hard work, but I just – in the last two weeks I've actually done half a shift on [workplace] and I had to do a cardiac rest so I've done some cardiac compressions, but that's not an every-day event but more just being, getting up, walking, moving, up and down stairs…

Interviewer: That's more regular.

Respondent: …carrying stuff, that's more regular, yep, certainly.

Interviewer: And would you describe that as - at the intensity of that as vigorous or moderate…

Respondent: No.

Interviewer: … or just steady.

Respondent: That's just steady, yeah. But you do have to have a level of it because to carry kits that weigh between eight and ten kilos and you've got a couple of them and you've got to carry them for a distance or up stairs or around you need to have…

Interviewer: You're certainly getting effort.

Respondent: Yep, but that's just at work.

Interviewer: Yeah, OK, and that's something that can happen as part of your work at any time.

Respondent: Yep.

Interviewer: And if you wanted to, is there anything that would have helped you to have been more active at work?

Respondent: Just your own impetus to go and do something. I work with a lot of people that can spend the whole day sitting in the armchair if there's nothing going on.

Interviewer: Right.

Respondent: So if there's no – if we're not out working you can just sit.

Interviewer: So it's either full on or it can be…

Respondent: At work, yeah. And it's not like we can actually – you could sort of go and go, "Well, this hour of the day I'm going to put my shoes on and go for a walk somewhere", because you can't.

Interviewer: So there's nothing that could really help that.

Respondent: No, not in my – if I use – cause I have two work environments – so if I use [workplace] as a work, I share a station with [workplace] so they go to the gym and use the gym equipment and that's fine for them.

If they get a call they just come and put their stuff on but we can't actually change into gym gear and go really to the gym when you're on shift because if your pager goes off then you need to be in the vehicle, out the door and you don't necessary want to go after you've been running on a treadmill for half an hour and you're head to toe in sweat. Having said that, I have done that when I've been – cause I've done on call for about ten years so if I didn't actually go and do anything in the four days that I was on call or working in branch stations – which does impede a lot of our officer. A lot of them don't like to do that role because you can't do your normal life for four days because you can't be far from your vehicle and you can't…

Interviewer: So it really restricts what actually…

Respondent: It can do, yes.

Interviewer: It sounds like you worked your way through that one. Now in the past two weeks have you walked or cycled for at least ten minutes of the time?

Respondent: Yes.

Interviewer: Walking or cycling?

Respondent: Both.

Interviewer: Oh, OK, to you get to or from places. Can you tell me about what you did?

Respondent: To get to places I've done a lot of walking because even if I take the last week – because [city] was spasmodic because of that but before

that – cause I know the two days I was in [city] I had appointments about three times a day each day and I walked to all of them in town, I didn't take the car at all and they would have been 15, 20 minutes away. And at home I have a bike so I ride a bike and I use the bike track here at [town].

Interviewer: OK. So you're fairly well served having a bike track?

Respondent: Yep.

Interviewer: And the intensity of the exercise?

Respondent: Moderate, yeah, it's moderate.

Interviewer: And how often?

Respondent: Oh a couple of times a week.

Interviewer: Yep, OK. When did you do it? You've said, "during the day", but the…

Respondent: The bike track's been of a – both of a morning in the last two weeks, but that can be anytime over that and walking's been all day and a couple of evenings, cause if I've spent a lot of time inside I often try to just go and just walk – even just walk around the block at home just to clear my head a little bit.

Interviewer: What a good idea.

Respondent: Just get a bit of fresh air, cause I'm not actually used to working inside all day every day.

Interviewer: Why did you do it there? What attracted you to that place? You were talking about walking and bicycling, obviously the walking during the day when you're at walk, that's clear, but what attracted you to the other walking out and cycling?

Respondent: Because they're activities that I can do. I don't play sport as in such – I'm not actually very coordinated and I enjoy them. I just enjoy them because it's thinking of time, you can be as hard or as not hard as you like so it can just be just a walk or you can actually quite stride it out a little bit and as I said, I'm actually impeded to actually do any high-impact stuff so both – neither of those…

Interviewer: So they work very well for you.

Respondent: They work very well for my – for what I can do. Cause I used to do a bit of jogging, I wasn't a serious jogger, but I would try and get up a bit of a – for half an hour, but I can't actually pound on my ankle so cycling and walking are the way to go.

Interviewer: Good ones, OK. Would you usually do that or was it a one-off?

Respondent: No, no. No. That's usual.

Interviewer: And does it change at all with the season?

Respondent: Ah yes, yep. I would definitely do more bike riding in the warmer weather and margin – and a bit less walking obviously in the winter weather. It's no fun to walk when it's blowing a gale or it's pouring with rain. So definitely less active in the winter and the daylight hours…

Interviewer: Yes, makes a big effort.

Respondent: Especially if I’m walking by myself.

Interviewer: Sure, OK. Now in the past two weeks have you been active around the house or the yard – inside or outside?

Respondent: Yes.

Interviewer: Can you tell me a bit about what you did?

Respondent: General housework: floors, vacuuming, mopping floors, dusting, gardening – I’ve mowed the lawns, done the edges, tidied up a little bit.

Interviewer: And the intensity of those?

Respondent: Moderate, yeah.

Interviewer: And how long?

Respondent: The lawn mowing took me – the actual lawn mowing itself only takes about 15 minutes. The edges take about 10 minutes and then just the tidying up, sweeping around after that and tidying up. So an hour’s worth of…

Interviewer: And how often would you do that?

Respondent: I don’t know. The outside – I don’t do a lot of outside cause I have a very low-maintenance yard and that’s why cause it’s not something that I enjoy, it’s a chore and when it needs to be done, it needs to be done. So it’s every six weeks or whatever it is.

Interviewer: OK and inside?

Respondent: Every week, normal house…

Interviewer: Can't get away from that, can we?

Respondent: I live by myself now so it's probably less than it's ever been because I've only got one person to take care of.

Interviewer: Fair enough.

Respondent: And I work five days a week now.

Interviewer: And is there a particular time of the day that you did these activities?

Respondent: Well, the lawn mowing was of a morning. The housework can be anytime. I can't remember actually when I would have done them, but I don't have a – put the washing on of a morning and probably cleaned the bathroom of a morning, but the floors can get done – actually probably the last time I done the floors would have been late afternoon cause I vacuumed and washed before I went out for dinner, or went out for tea as a family.

Interviewer: Does this change this activity depending on the season or the time of the year?

Respondent: Probably the time, not actually what you do, but probably when you do it. Summer time: would definitely tend to do things much earlier in the morning so that your days are – you can go and do stuff. Winter: it's often cold so it might be – and specially now, this is the first time I've ever worked a full Monday to Friday sort of job, so

before that I would have – and that may have depended on what shift you were on.

Interviewer: Fair enough. If you'd wanted to, is there anything that could have helped you be more active around the house - inside or outside?

Respondent: Only my own enthusiasm.

Interviewer: Have you been physically active for at least ten minutes during your leisure or spare time in the last two weeks?

Respondent: Yep.

Interviewer: Can you tell me about what you did?

Respondent: That was…

Interviewer: You've already mentioned that.

Respondent: … the cycling and riding and I go – as I said – go to a gym so I have a personal trainer twice a week.

Interviewer: And that intensity?

Respondent: That's higher, yeah.

Interviewer: So that would be fairly vigorous?

Respondent: Yes.

Interviewer: And how long?

Respondent: 30 minutes.

Interviewer: 30 minutes for the two times that you go?

Respondent: Yep.

Interviewer: And that's when during the day?

Respondent: Usually morning. 90 per cent of the time is morning. I've got one Friday afternoon but that's more because I'm going to be away.

Interviewer: And why do you do it in the morning?

Respondent: I just like to get it over and done with cause sometimes by the end of the day it's too easy to go, "No, I can't be bothered". Or something comes up or you just – I like to get it over and done with in the morning and it does actually make you be – it makes you, sets you up for the day.

Interviewer: Fair enough. Where would you do that? You've talked obviously about the walking and the cycling, but the gym's fairly close by is it?

Respondent: Yes it is.

Interviewer: And you (inaudible – over speaking [25.23])

Respondent: It's within 500 metres of me.

Interviewer: Oh wow.

Respondent: So I walk to it and back.

Interviewer: Good. Do you usually do that with others?

Respondent: No, it's a one-on-one.

Interviewer: Does this change with the season and the time of the year?

Respondent: It's only new so I can't really comment on that, but when I was going to a different gym a while back, yeah, absolutely.

Interviewer: So how long have you been doing – going to this one?

Respondent: Only a couple of months now. I don't know, it will be interesting to see whether it changes in the winter when it's very cold and it's early of a morning. I probably'd be less inclined to work.

Interviewer: How early?

Respondent: Some are 6.30, some are 7, so 7, 7.30, so early morning. So I'd be probably less inclined to walk there than I am now because when it's daylight it's much easier to walk.

Interviewer: So bearing in mind all of these activities we've talked about, the walking, the cycling and the gym, if you wanted to is there anything that could have helped you be more active in your spare time, your leisure time?

Respondent: No, not for me personally, only my own enthusiasm to go and do stuff. Cause I don't have any impedance so it's not like I can't get to anything and I can't go to it. So for me, no.

Interviewer: Now, that's the end of those questions and now we're going to talk a bit more about the physical environment, OK? So talk about where

you live and work and play. And when we talk about physical environment I'm meaning all the physical things that surround you like roads and trees and houses and shops and traffic and parks and all those things. These are some of the things that people in urban areas have said are important so we'd like to ask whether they're relevant to you too. Now, can you tell me a bit about how easy or difficult it is to be physically active in your neighbourhood.

Respondent: I think in my neighbourhood, because it's flat and we have a very good pathway, it's easy if you want to cause it's a walkway that's safe as far as no – limited traffic and it's…

Interviewer: When you say "a walkway", is it a track or a footpath?

Respondent: A walking footpath. So we have a footpath – I can walk all the way out to [x] Beach on the walking track so I think personally and the use of that track has certainly – we would never go on it without there being other walkers or cyclists on it and just driving along the highway. It gets a lot of use and I think the tracks that are being done more around certainly enhance people's ability to get out there because it's – if you've got children it's safe, if you're – there's traffic, it's easy to get to, it's flat and it's well maintained.

Interviewer: Sounds great.

Respondent: It has spots of it that you're on like a beach road sort of thing…

Interviewer: Most of it's a track.

Respondent: Most of it's a track and well maintained and flat.

Interviewer: You've just described what makes it – why – what makes it easy. Now I'm going to ask you a few questions about specific bits about the physical environment. The first is about availability and accessibility of the places to be active. So if I was to ask you about the places to be active in your neighbourhood, what sorts of things would you tell me about?

Respondent: Well there's a good walking track, good cycling track, there's a bit sports stadium. I think [town] itself is a very sporting town. We have a lot of team sports and we have a number of gyms. Whether they meet everybody, I don't know, but we do have a number of them.

Interviewer: And then you've also, were you talking about the walking tracks, bicycle tracks along the coast and…

Respondent: Yep.

Interviewer: And access. Are the places convenient and are they easy to access in terms of…

Respondent: I think so. Well, they are for me because I only have – I walk out the door and go about 200 metres and I'm on the walking track.

Interviewer: And for the other facilities with the cost and opening hours that's…

Respondent: Yeah, I'm unable to comment on the cost and opening hours of some of them. I know they'll all be a certain amount but – and then if you can travel, if you can get to [city] they have a myriad of different gyms and there's a 24-hour one somewhere now. I think there must be one in [city]..

Interviewer: Really? My goodness.

Respondent: But I've never – I've not really used outside of my…

Interviewer: Your own community.

Respondent: … my own community, yeah.

Interviewer: Does having places to be active influence whether you're active or not?

Respondent: I'm sure it does, yeah. I'm sure it does because I have friends that come from the country that would come down – that come down and park their car and walk down here and go for a walk because of that – because it's safer because they can walk – and they only live five Ks out of town but you've either got to walk along a road and then if it's dark – or you can come down here and take a friend and your dog – and they're dog friendly. We have dog-friendly beaches, we have a couple of dog-friendly beaches here, so that makes it easier to go and if they're on a lead or the tracks are – and cause you're not walking past houses and yards and things like that – so if you're taking animals for a walk it's a lot easier cause I take my daughter's [dog] so when I take him, if you're not walking past people's houses and where there's other animals all the time and cars, then it's a lot easier to take your dog for a walk. You're more inclined to if it's animal friendly.

Interviewer: That's a good point. And if you wanted to be more active are there things or places that would help if they were available or more accessible?

Respondent: I'm sure there would be, yeah. There's gyms that have classes so there'd be the exercise classes and I'm sure you could join a cycling club.

Interviewer: And destinations. If I was to ask you about places that you could walk or cycle to from your home, what sorts of things would you tell me about? Now you've already told me quite about…

Respondent: Yes, it's easy to cycle to the towns either side so out to, which is six – out to [x] Beach and then – because if I ride from my house out to [x] Beach and back it's a 15 K ride all the way – all around…

Interviewer: Really? OK.

Respondent: … to get out there and back.

Interviewer: And you can do that relatively safely with the roads?

Respondent: Yes, well it's not on the road.

Interviewer: Oh OK, cause you can use that track. That track's a cycle and a…

Respondent: Yeah.

Interviewer: Oh excellent, it takes cycles and walking. That's great.

Respondent: My understanding is that we – cause they've got the [city] one which goes a very long way now and they're eventually trying to get one that meets up so they're trying to meet…

Interviewer: That will be exciting.

Respondent: Yes, and then they've got one near [city]. They're trying to get them all to meet. Eventually the plan is that you would be able to cycle the coast on a track.

Interviewer: Wow, that will be very exciting.

Respondent: I think it'd be excellent…

Interviewer: Yeah, absolutely.

Respondent: … because you could do it in a leisure environment, a group of people.

Interviewer: Things to look forward to. So does having destinations influence whether you're active or not?

Respondent: Quite possibly because we've just gone for a ride out for something to do because we rode out and had an ice-cream at the Strawberry Farm and rode back again. Or we've got on the bike – I can actually tell you I have some friends that come from [suburb] out the back of [city] and they bring their bikes and they park at [x] Beach. I ride out and meet them, we ride back in and come in for breakfast and then they ride back out again.

Interviewer: Oh right, that's good.

Respondent: So it's a whole, yeah – so I don't know that's just what we do so it's – I have a motorbike and go motorbike riding and often we'll ride to somewhere to do something and then ride back so it influences the ride.

Interviewer: And if you wanted to be more active would it help if there were more destinations?

Respondent: Yes.

Interviewer: With easy walking and riding.

Respondent: Yep.

Interviewer: Now neighbourhood design. If I was going to ask you about built, man-made or non-natural features of your neighbourhood, what sorts of things might you tell me about.

Respondent: Such as?

Interviewer: Footpaths, lighting, whether your streets connect together, those sorts of things, their walkability.

Respondent: Yeah I think that has – that's why I think [town] works well because it's flat and it caters for that and the tracks that the [town] treks you can go all the way from the rowing club all the way along the river and down and around and out and it's a few Ks worth and now out to [x] Beach and back so you can do a really good – as I said, I ride and it's roughly about 15 Ks. It's about seven Ks out there and seven Ks back so that's a good…

Interviewer: So do – I think you've already answered the question – do the built or non-natural features in your neighbourhood influence whether you're physical active or not?

Respondent: Absolutely because it's easy to go if you've got a well-lit street to go walking when it's dark, yep.

Interviewer: OK.

Respondent: Which helps in the weather if it's…

Interviewer: Gives you more choices too about when you want to go.

Respondent: Yep.

Interviewer: And if you wanted to be more active are there any man-made or non-natural features in your neighbourhood that would help?

Respondent: If we didn't have those tracks?

Interviewer: Uh huh.

Respondent: It wouldn't be as easy so I think they're the bits…

Interviewer: They're the things.

Respondent: Yeah, they're the things I think that make it worth while.

Interviewer: Great. Now the aesthetics or the natural qualities in your environment, the other side of it, the attractiveness of your neighbourhood area, if I was to ask you about that what sorts of things would you tell me about.

Respondent: Parks, the parks. We've got lots of parks so it's nice to walk.

Interviewer: Lots of nice green spaces?

Respondent: Yep, so even the walking track goes through a couple of parks so that's always nice and it's open and it's airy and it's well utilized so it's safe.

Interviewer: You've been telling me about the coast as well. You're rather blessed, aren't you?

Respondent: Uh huh.

Interviewer: And the other side of it is – apart from the scenery the other side is the rubbish, litter, graffiti. Is that an issue here?

Respondent: No, well not in the areas that I go to, no.

Interviewer: And so do you think your local environment is pleasing and attractive?

Respondent: Yes.

Interviewer: And near your home as well obviously, yes.

Respondent: And having spent a bit of time working in some [x] Coast areas and [council] I do appreciate how lucky we are because I've done – just tried to do the same things in those areas and you don't have the same activities. So if I work in – I worked in [town] in [city] and you can still go for a walk but it's not a track. You're either on a footpath walking past normal streets or on the road when there's no footpath and it's – it's just not as enjoyable because you can't just put your headphones on a walk 'cause you've got to consider…

Interviewer: Watch for traffic and what's going on.

Respondent: … traffic, yep.

Interviewer: So do you think the aesthetics of your neighbourhood influence whether you're active?

Respondent: Yes I do.

Interviewer: And if you wanted to be more active, are there features of your neighbourhood that might help if they were more aesthetically pleasing?

Respondent: Yes and I think that I do know that sometimes it's having just stops that you can have and have a bit of a rest so where there's a tap so you can fill up your water bottle or there's a seat so you can have a bit of a rest if you want to stop and have a bit of a look, yep.

Interviewer: It all helps, doesn't it?

Respondent: Yep and it certainly makes a difference when there's lighting and it's a safe area so you don't actually have to walk or go to spots that you think, "Oh yeah, I won't"…

Interviewer: That was the other question I was going to ask you, the last of the series of question is about safety. And if I was to ask you about safety in your neighbourhood, what sorts of things would you tell me about?

Respondent: Lighting, definitely lighting in the winter is a big issue I think because…

Interviewer: You need to have more lighting.

Respondent: … our mornings are long and our evenings are long so I think lighting is a really big issue, makes a big difference if you've got a well-lit area to walk in – safety, yeah and footpaths.

Interviewer: And footpaths, decent footpaths.

Respondent: Decent footpaths and lighting, yep.

Interviewer: Do you have decent footpaths?

Respondent: I think we have reasonable yeah.

Interviewer: But there's always room for improvement.

Respondent: Yeah, well I've just been for a couple of walks in [city] of an evening and I really considered – cause I didn't know the city at all – so I wasn't quite sure where I went but I done a bit of a back track a couple of times because I'd get sort of a block along one street and then I'd realize that was the only populace area of it and because I didn't know I would walk back that way to go a different block as opposed to keep walking along cause it looked darker and I didn't actually know the region.

Interviewer: Fair enough.

Respondent: Not knowing the environment at all I didn't know whether you were going to end up down a street and in the next block and two, three blocks down you think, "Mmm, OK, I've come this far, I don't actually know where the next well-lit or populated area is", so it was easier to go back, yeah.

Interviewer: Fair comment. So it is important. So does personal safety influence whether you're physically active or not?

Respondent: Yes.

Interviewer: So the feeling of physical safety here is important for you?

Respondent: Yeah and that's why I think it's so good to do that in [town] cause the areas are…

Interviewer: Not only from traffic but personal safety as well.

Respondent: Safety, yep.

Interviewer: And does road – talking about traffic – does road safety influence whether you're physically active or not?

Respondent: I'm not sure because I've always had – been able to use these so whether it would have had I not been – and the times that you're away and you have to use other ones – and that's probably when you realize how lucky you are so it quite possibly would if I didn't have them. That's only thinking about it, it doesn't now, but if I was think about it, yep.

Interviewer: And if you wanted to be more active are there personal or road safety issues that could be addressed that would help?

Respondent: Not for me personally cause I just use the other areas but I'm sure if you didn't have them to use there would be issues and I think there's things that we can do like – where I like seeing walkers in vests coming from a safety point of view because I've been to a few pedestrians hit so I like to see walkers in vests. I think that's a very –

it should be something that should very much be promoted to just make yourself more visible, absolutely and on bikes, wearing vests, having good lighting. I don't think there can be enough education around that.

Interviewer: OK, that's the end of those questions. So just the last one to finish up: is there anything that you'd like to tell us today about where you live or your environment or your physical activity that we haven't covered today that you think's important?

Respondent: I think I've just about covered it all.

Interviewer: OK.

Respondent: Sounds like it's all brilliant and can't be improved on. I'm sure it can but…

Interviewer: What's interesting is what works for you too. That's really very valuable. So thank you very, very much for that.

Respondent: No, thank you.

ate: 16 November 2011
Duration: 28 minutes, 8 seconds
Interviewer: [interviewer]
Transcriber: [transcriber]

So today what I was hoping to talk to you about was hearing your thoughts and experiences on a range of issues related to where you live, as well as your physical activity behaviour. So remember there's no right or wrong answers. I just want to hear what you think and what your opinions – and as I said, stop at any time if you want to, or don't answer any questions if you don't want to. So we start off in these

interviews just by asking a bit about where you live. So if you want to tell me a bit about, you know where it is and what' it's like, to live where you live.

OK. So I was brought up at [town] on a farm, an apple orchard, beef cattle. So very much a um, it's not a broad acre farm like I guess some of the people you've been interviewing. So it's more condensed farming. And I was down there until 12 years of age and at the boarding school in [city]. And now I spend a lot of time down there of course, but work in the city.

Yeah. OK. So you live down at [town]? Or you do a bit of…

Oh, I've got the back and forth. I've got a place in [city].

And down there too.

Yes.

So roughly in your week kind of, would you spend most week nights here?

75% in [city] and 25% at the farm.

Yeah. Sure. OK. And that farm, the apple orchard, that's where you are now? Or you've just got a separate..

Oh I've got 15 acres of land, just adjacent. So it's all on the one part of the property.

OK. And how far are you sort of from the main bit of [town]?

Oh it would be about 8km out of [town].

OK. So about a 5 minute drive or something.

Yep.

Yeah, so not too far. Alright. And how long have you had that property?

Oh it's been in the family seven generations. So 150 years and ah, but it was very small until my father, he expanded it. So it's now, it's a reasonable sized apple farming operation. So it would be the third or fourth largest in the State, I would imagine. So yeah, that's it.

OK. Cool. Um, now in some of the people that we've talked to that live in urban areas - and you would probably relate to this as well – they often talk about their neighbourhood. And I guess something that I'm interested in asking guys that have an experience of living in a rural area is does the word "neighbourhood" sort of mean anything to you when you're down that way?

Yeah. Because it's not broad acre, um there is a neighbourhood. So for example, where um [x] Rd, [x] Bay, where our farm is, so it's on the water and there would be

20 houses in the precinct, in that little stretch, in a kilometre stretch. And so there is a neighbourhood there where everyone knows each other. And there is a good relationship. And probably compare that to living in [city], um then the community is a lot tighter than living in [city] where it's more – it's a small city of course, but it's still hustle and bustle. And you might say passing hellos, but there is generally not, not a great – It's a good community feel I should say.

Yeah, OK. Well that was actually my other question, was is it sort of different to "community"? Is there a difference between the two words?   Neighbourhood and community?

Than neighbourhood and community? I think community is probably a stronger relationship for - you know important things for my children in a community sense is that they have that sense of tradition but also a sense of belonging. So you have an affinity with where you've been brought up. And I think ah, my best friend, who lived in one of our family properties – we are still great mates now – um, we first met each other when we were toddlers. So, and he still has an affinity with that place, yeah. So I would say it's more community than neighbourhood. Neighbourhood sounds like a suburb sort of thing.

Yeah. OK. So can either of those be sort of quantified? You know is it a distance thing? Or is it more just about the people?

Oh, I think a community is more about the people. Um, and your ability to communicate with them. And get in touch if you need be. Where a neighbourhood to me doesn't seem like a – it just seems like a geographical area, that you are there.

So it might be the people that live in this-

In this vicinity, yeah.

Yeah, OK. Sure. OK. Alright. So we are going to switch and start to have a chat about your physical activity. Um, and what I'm going to do is go through a few sort of areas of your life, I guess, just so we sort of cover off everything, and just see how we go. So what does "physical activity" mean to you?

Well, I'm actually very active. So physical activity to me would be – I play regular sports and played sport at quite a high level before I got too old. So, now it's more a daily hour walk with the dog, so and um some weight training, and also with the children. So the kids are quite active, so we do a lot of running at the ovals or kicking

a football or cricket. And I still coach a cricket team, so I'm still actively involved with that.

OK. You sound like you are doing quite a bit.

Yes.

Um, so in terms of whether physical activity is a priority in your life, or does it fit, you know is it something you try and squeeze in, or is it something that you consciously…

It's very much a priority in the life – but probably when we had the third child it became a backdrop to when I could fit it in. But I function best when I've got physical activity. And I know my wife does. And she has found it very difficult since we've had children to do that. But for her to function, mental health, I think there's a direct relation to physical health. No doubt, yep.

OK. Alright. Well we'll talk about each of those things in a bit more detail, but um I guess you know in the research that we do, we think about physical activity as you know, the sort of textbook definition is activity that lasts for at least ten minutes and it's usually of at least moderate intensity. So you might breathe a little bit harder, your heart rate goes up a little bit. So you know, anything from walking onwards. Um, so thinking about that sort of definition, um in the last two weeks have you been active at all at work, um for ten minutes at a time?

At work? No! (Laughter)

I'm with you on that one. Yep, no problem. And so why is that? Is that ..

My office job is based around this desk. And if I do walk to a meeting, it's in [city]. So you wouldn't get, your heart rate doesn't go up at all. So I try to get my exercise out of work hours.

So work – it's not something you do at work. And that doesn't change, that's pretty much the standard throughout the year – that's day to day. You're in an office job and that's that.

Unless we go and play golf or something like that.

I guess that counts as work.

Yeah, it doesn't happen as often as it should. But as opposed to a farming, if I can just say the apple industry, where it's not really machinery driven. It's not tractor driven where you are sitting on your backside all day, or driving a truck. It's a, you

are on your feet, you're walking, you're using your, all your levers to get the tree into a position that it's going to grow fruit, so that's *very physical.*

Sure. And do you do that? Are you involved in that aspect of farming?

Ah, my brother owns the farm now and runs it. But I do assist in the season when they need.

Sure. Is that more something you do sort of in your spare time? Like on the weekends and things?

Yeah. That's right.

OK. Alright. So thinking about work activity, if you wanted to is there anything that could be done to help you be more active at work? If you wanted to.

Um, we actually have [researcher] who is a PhD in physical health that she is doing in psychology, but she did her study on mental health in the workplace and the direct relation to physical activity. So we did a six month trial with her last year. All of the staff went on pedometers and brought in fresh fruit to eat during the day, we limited our coffee intake to one to two per day. Um, that was interesting. So they're the type of things that if it was consistent in a business environment, I think it's healthy. And people were going for walks in their lunch breaks. And making it a bit of competition with the pedometers. But yeah, they're the type of things that yeah you could, you probably should, put into an office culture.

Yeah, OK so that is something that could potentially help with, yep, if you wanted to. I'll have to ask you a bit more about her project when we finish the interview, that sounds really interesting. Alright, so thinking about activity for travel, for transport, have done any sort of that, you know at least ten minutes at a time to get to or from places? Either walking or cycling?

Yeah. Well I live in [suburb]. It takes me two and a half minutes to drive here in the morning and I haven't walked once. There you go. So that's..

OK. No, no, that's fine. That's what you do.

I guess the thing with my occupation here, a lot of it is um, my activity is driven by people to come and see me. So we're a sort of professional office. We're [industry] here. But we're not like the old insurance salesman or product flogger who would run around and do the door knocking. We don't do that. We try to treat ourselves

like an accountant or a legal firm where clients will come to us and we'll sit down and look at a broad range of things. So you do tend to get tied to the office.

To the office. Yeah, so you don't tend to walk or cycle to or from places. What about from home? Is there sort of anywhere that you – do you get in your car?

Yeah, oh definitely. At home we're, once again, a two minute walk from the [suburb] shopping precinct, but we, we do a lot of walking activities. So very rarely will be drive from home to walk to supermarket or whatever.

So, say that again? So you would normally walk to the supermarket?

Yes.

Yeah? OK. But that's sort of pretty close, is that what you're saying?

That's right.

So you do do a bit of active transport. It's just that it's shorter duration just by the very nature of where you live.

Yes.

Yep. OK. Alright. Um, and does that change at all any of those habits, depending on the time of the year or where things are at in your life? Or is it pretty much a…

It's pretty steady, pretty consistent.

Yep. No worries. And if you wanted to, so going back to that hypothetical again, is there anything that you know would have helped you if you had wanted to be more active? In terms of getting to or from places?

If you made a change, you could walk to work I think. And walk back from work.

If you wanted to.

If I wanted to. Um, I guess from my point of view, I sort of make up for that by saying "right, well I have a fairly set physical regime". So it sort of works.

Yep. So you're happy with how things are at the moment. Yep. Alright. So now thinking about, we'll talk about your outside and your sport and all the rest in a minute, but thinking about the house and yard? Um, anything you do either inside/outside that would sort of be, you know fall into that definition of you know ten minutes.

OK. Gardening. Yeah, do a lot of gardening. We've got quite a big yard here. And obviously at the farm, we've got a broad acreage. So um you know the children get a

lot of activity um and I guess we get our activity with them, by chasing them. So, but the gardening certainly provides that short initial burst of activity.

Yeah. And is that sort of something you tend to do on the weekend or after work?

Yeah. After work – depends on what needs to be done. But it has to be done weekly.

Yeah, OK. And is that the same as at [town]? Or is that ..

Yeah, ah [town] is a little bit, ah, doesn't have to be. Because the garden as such isn't as great down there. It's more pasture.

Yeah. Sure. How often did you say you go down there?

Pretty much every weekend. Yeah.

So you do do it every week.

When we can get there, yep.

Alright. And if you wanted to is there anything that would have helped you be more active around the home? Or are you happy with how things are at the moment?

[name] would probably say that there's lots of things I could do. Look, I think they're the main things. We're doing a big extension at the moment, so we probably can't do a hell of a lot there.

Alright. So now talking about the things you do in your leisure or your spare time? Um, you mentioned a few sporting commitments and walking.

Yes.

So do you want to tell me a bit more about, you know what it is that you do and when you do it and that kind of thing?

OK. So probably five days a week there would be an hour, personally, an hour walk with the dog. So that would be from our home up Mt [x] steps to the top and back. So it's quite a rigorous high heart rate walk.

Yeah, yeah I reckon. Is that just on your own or do you take the family with you?

Nah, it's just by myself. Um, so that's generally early in the morning, or later in the evening. Um, but I would do two runs a week. So those runs would probably be 6 - 8 kilometres. Um, and there'll be three, just weight sessions. Just at home, I've got a little home gym. Yeah. So that's personally. Then I'll be cricket training once or twice a week, um which would be for two hours at a time.

So you're the – are you the coach? Or are you…

I'm just assisting. I don't do it as a full time commitment. But ah, and I'm playing a little bit to help out when they're short. But I don't - look I stopped playing properly three years ago. So it's a bit more relaxing. Ah and then the other activity, my eldest boy, he is quite active now, so we are probably at the oval for an hour twice a week kicking balls or doing sprints and stuff like that.

Is that soccer ball or football?

Football. And cricket. Now he's getting into cricket. And in the yard we've got basketball set up and all that kind of stuff.

So a bit of active play as well with the kids.

Yep.

And thinking about your running, is that on your own? Or do you do that with someone?

Oh, by myself.

You go out by yourself? And the weights as well? Is that yep, just a solo sort of activity?

Yeah.

Yep.

It's just where you try and fit it in. I guess all my previous sport activity has always been as part of a team. And that's terrific. And it's much more enjoyable. But with my lifestyle at the moment, you can't really organise a set period, so. But I would probably commit ten hours a week comfortably to activity.

Yeah, that's great. Um, alright and so I'll ask that question again: is there, if you wanted to, is there anything that would have helped you to be more active in your leisure time?

Not having to work. (Laughter) I'm a very active, I'm naturally very active, so I mean in a confined space like this, does my head in. So I've got to certainly get out and about.

Yeah. And how, I mean how does that sort of change in terms of the weekend and things as well? If you go down to [town], does that sort of mess with your you know, doing your runs and walks and things?

No, it's better. It's easier. Because the children are more relaxed. There's no doubt the children are more relaxed in the farming environment. Because there's no fences

behind them and there's no cars to run them over. Dams are the only things that you've got to be wary of. And so it's a lot more relaxing for everyone. They can tootle off and you can keep your eye on them but without having to follow them.

Yeah, OK. Good. OK. Well we've talked a fair bit about your physical activity, but now I guess I want to have a bit more of a think about the environment. So where you live and work and play I guess, as well. Um, so when I'm talking about the environment I mean you know everything: the man-made stuff, the roads and street lights and that kind of thing, as well as the natural trees and mountains and those kinds of things. Um, so in terms of I guess in terms – it's a bit tricky for you because you've got two homes I guess, in a way. But maybe um probably maybe you can talk a little bit about both. But I'm particularly interested in what is going on when you are down in [town]. Um, in terms of availability and accessibility of places to be active, are there places – I guess what do you think of when I ask you about availability and accessibility of places to be active in [town]?

[town] is quite good from a small community, as such. Ah, it's got the big football oval. But unfortunately there's no football team anymore. Um, there was a successful cricket club there but that's no longer operating. So from a town point of view, a lot of those club/ community services are now dying off. Which is sad.

Yeah, OK. Do you know why that is?

I think the change in demographics. Um, it all of a sudden went to an older population. But now it's changing and there's some younger people coming through. But with the, I think, the accessibility of [city] now, or the major towns, a lot children are going there. Because they may perceive to get better coaching or support. Um, which is true on most aspects. But in terms of what's available, I think you know the whole, the township has tennis courts, it's got um a gymnasium with a pool, and basketball, squash. Um, it's got a tennis club, it's got a golf course. And they are all in very much close proximity. Which is handy. So growing up down there, all those activities were terrific. But I think the benefits of a childhood in the country, and adulthood in the country is that you've got this open space. So at the end of the day you've got this ability to get out and about. Probably one thing I would say now in those areas, there's a lot of potentially, my – not my friends but acquaintances that I went to school with down there or know of that – because it's such a laid back

lifestyle, people tend to become lazy. And a lot of that then revolves around going to the pub for drinking. Which I know there is a bigger drinking culture in the country than there is with my mates in the city.

OK. So that's maybe taken over from some of the sporting activities.

Yeah, sporting, yeah. You're right.

And so thinking about those places that you are talking about. You know the golf courses and all those things, they are reasonably accessible?

Yes.

So in terms of, they are easy to get to, um cost-wise, opening hours, those sorts of things?

Yes. There's no excuse for anyone really not to be able to do it.

Yeah, alright. Um, so there's this question around, you know, if you wanted to be more active when you are down there, are there things or places that would help you if there were more available or accessible down that way?

I think sporting clubs. I think that would be the thing that needs to be improved in these areas.

Yep. Would that be for you, or just in the community?

Not for me now. But the general community. If my children were going to – they wont be going back on the farm because it's my brother's, my brother's family's now. But if they were, definitely that would be a requirement. Because I think people need that competitive spirit in their life. And they need that community and team based experience in their life, I think. So that's the issue, and with all sports generally becoming more professional now, they centralise the services and the specialities. So that is at the expense of regional areas.

Yeah. So where do people go from [town] if they do want to you know, do this? Is that going up to [town] or..

Yeah and this is the issue. Talk about cricket as an example. If you're good, it's like if you are good you go to the big premier league clubs. And you know, [country] cricket is obviously the strongest environment in the world, and it's very competitive. But the best go to these sporting clubs, and the weak will perish effectively. And they miss out on the positive things of being part of a team. You

know, that involvement and friendship. All those types of things. And working together. They miss out. And I reckon that's a real issue in community areas.

Yeah, sort of in rural areas?

Yeah, rural areas, sorry.

Yeah. Alright. Um, so thinking about destinations from your place in [town]. Are there places that you could walk or cycle to if you wanted to? So services, shops, post box, friends places?

Yeah, the closest services are yeah, 8 kilometres away. So um, you're probably looking at a 40 minute run or a 20 minute bike ride.

Yeah. Um, and do you tend to do that?

Yeah, look I tend to, when I go off on my runs. I haven't cycled it. But when I run, like if I do the 8 kilometre run, my little boy will ride his bike alongside.

OK. And is that with sort of a destination in mind?

You run a loop or run back. Or you'll run to the shops and I'm too lazy to get back, so XX will come and pick us up.

Yeah. OK. And if you wanted, would having more destinations or different destinations influence whether you are active or not when you're down there?

Ah, not me personally.

Other people maybe?

I think so. Yeah. I mean there used to be in that little community, there used to be a post office and corner stores. And if you compare the generation of my father where they used to walk to football training or walk to school, and back every day – which would have taken an hour to walk to the school and back – where to now, it doesn't happen.

Yeah. Alright. OK. So now thinking about some of the man made features in the [town] area. So again this is sort of you know what urban, people living in cities are sort of telling us. And I guess what I'm trying to get at is you know is it the same in a rural area? Or is it a bit different? And trying to understand where the differences are or the similarities. So if I was to ask you about the built or the man made features of your sort of environment down in [town], what sorts of things would you tell me about? Are there footpaths and lights and things like that? What are the roads like?

Infrastructure is generally good. Like a lot of the back roads are still gravel, but ah in terms of the city, the town is um, yeah all the services are there. I would say it's very well serviced, and um maybe over serviced for all the different bits and pieces you can get. Um, there's enough lighting, even on the country roads there's lighting. So, I think it's fairly good.

OK. Footpaths, are there many around?

Ah, there's a few walking tracks in the main centre, yeah. And the footpaths, yeah they're fine in the shopping district.

And what about out where your property is? There's not..

No. No there's no footpaths.

Yep. And would you walk, would it be sort of safe to walk on the road there.

Oh, look, no, well you'd want to be, it's not safe to walk on the road at different stages of the day, like early in the morning, late in the evening. Because cars tend to whip through there. But ah, yeah walking tracks would be a lot more appropriate. And even cycling, bike tracks would be a lot more um, appropriate.

Yeah, OK. So they're not there at the moment, but they would ..

Generally the cyclists or runners running on the main road competing with log trucks.

And that's probably not great, not only for yourself, but for the kids as well I suspect. You don't want to send them out onto that. Um, so does that, do you think those features tend to influence whether you're active or not? Or not really.

No, it doesn't.

Yeah, you'd do it.

Yeah, regardless.

Irrespective. Yeah. And um, so the next question is probably a bit redundant, but if you'd wanted to be more active are there any features that would help you in terms of the infrastructure ..

No.

Alright. Moving onto aesthetics. Um, do you want to tell me about the aesthetic qualities or the attractiveness of your sort of area down that way? Is it a nice place to be?

Oh well if you talk [town], oh it's a bit of a hole. You know, it's a frost pocket, it can be cold, it's in a little gully. Where if you talk about the farm, it's beautiful. It's a sloping bank up to the top of big hill, all over the [x] River. So it's a lovely views and there's sun aspect all day long and so it's beautiful, yeah, stunning. So we spend our time at the farm, not at [town].

Not at [town], the town itself. And in terms of thinking about [town], the town itself, is it um, I mean is it not a particularly, in your view, not a particularly attractive place? Is that sort of what you were..

Yeah, oh it's not an attractive town.

Um, and does that influence whether you're active there or not?

Oh, no, I don't think so. No.

And is that because you would be active anyway, or because you're active in other places?

If I was living in the city, the town centre, it would impact. But because I live out, no it doesn't impact.

Sure. Alright. And we touched on um road safety just a little bit before and because I was going to ask about road safety and traffic safety. So are there anything you want to tell me about road and traffic safety, and as well as personal safety?

Yeah, there's a huge amount of accidents down there.

Are there?

So, very poor road safety and signage. I mean there's not enough population to have traffic lights. But there are a lot of intersections which are very poorly signed, I would think.

OK. And so a lot of car accidents. Is that what you mean?

Yeah. There is. And the advent of tourism, there is a bit of a tourism boom down there, there's a lot more traffic on the roads. So, because the roads tend to be windy because of the natural topography of the area, it can be – I think it needs to be improved.

Yeah. OK. And so um, does that sort of influence where you go when you're active? That safety aspect? You know, are there certain places you avoid or ..

Yes, there would be. I mean there's two roads where you can go for a run. One you are better off not running on, just in case, yeah. Especially with the children. I would only ever take them on one route, not the other.

OK. Yeah. And why is that? Is that just that on? Is it speed limit or is it the, just the danger?

Oh the speed limit is the same, but it's just the winding. It's the blind corners. And lack of signage.

OK. So signage around slowing down, and watching out for pedestrians, that kind of stuff?

Yeah.

Alright. And what about um, personal safety? Are there any issues around sort of crime and …

Oh no, that's not an issue.

What about going out at night and that kind of thing?

The night life down there (laughter).

Not that sort of going out! I mean if you want to go for a walk or a run or something in the evening would you..

I'd be very safe.

You'd be pretty safe, pretty safe to do so.

Yeah.

Um, and um you've sort of already touched on this, but if you wanted to be more active are there certain road or personal safety issues that could be addressed that might help that?

Yeah, look I think that the road signage and more awareness and improving of the roads is very important, and need to be done.

Yeah. OK. Alrighty. OK, well that's all my questions on my list. So um –

Easy.

Thanks very much for talking to us today. And before we finish is there anything you want to tell me, anything else about where you live or what you do and whether it's different or any other insights for me?

I think we've covered it pretty well.

We've covered everything off, yeah. Good. Alright.

Done well.

Alright. Thanks.

AUDIO RECORDING ENDS

Interviewer: OK. I just want to start by talking a bit about the place that you live in and can you tell me what it's like to live where you do, your likes and dislikes about it?

Respondent: OK. What I actually like is we live on a farm, so we've got acreage. And I like having the space and the freedom and I like the environment for my children because I feel that they can be quite safe, like they don't have to... they can go out and play in the paddock or just play in the extended back yard and they're relatively safe. Whereas if I was living in town we'd have a very small back yard and I wouldn't... they certainly wouldn't have the freedom that they have living where we live.

Interviewer: So town being for you would be?

Respondent: In [town].

Interviewer: In [town]. So tell me exactly where is the farm?

Respondent: OK. My farm is situated probably ten minutes from [town]. It's 14 kilometres out...

Interviewer: OK.

Respondent: ... it is. So for the kids and myself it's always a car trip.

Interviewer: OK. So what area is it in?

Respondent: It's in [town] [inaudible 1.18].

Interviewer: That’s right. In [town].

Respondent: Uh-huh. In [x], [x] coast.

Interviewer: Uh-huh I’m with you.

Respondent: Uh-huh. So it’s a rural, rural area.

Interviewer: And how big an acreage are you on?

Respondent: We’ve got 179 acres.

Interviewer: Oh, that’s a nice size.

Respondent: Uh-huh. It’s fairly substantial. Uh-huh.

Interviewer: A bit of work to do to keep it on.

Respondent: A lot of work. A lot of work. Uh-huh.

Interviewer: Have you got animals as well as...

Respondent: We do have animals. We basically... we don’t crop, it’s basically livestock and it’s probably more of a fairly high risk thing that we do. We’ve got a horse stud and we have some sheep and normally we have cattle as well. But we basically breed and train endurance horses on the property.

Interviewer: What breed of horses?

Respondent: [x] horses.

Interviewer: Oh beautiful. Love [horse]. Uh-huh. They're gorgeous. And certainly great for endurance work.

Respondent: Uh-huh.

Interviewer: So... and the length of time that you've been there?

Respondent: We've actually been there ten years we have.

Interviewer: And your reasons for living there?

Respondent: Originally we actually lived even ten minutes, 15 minutes further back and the reasons we actually moved to the property that we're at now, one, it was a bigger property, and the fact that it was ten to 12 minutes type thing out of town, more convenient for the kids. Because when my daughter was little it was a full day, we'd come in to town and you'd just be absolutely exhausted by the time you get back home. And it was also, yeah, fairly isolated I guess where we were previously. Whereas where we are now it's not as isolated. Uh-huh.

Interviewer: And now you've said your distance to your nearest town is [town] is ten minutes, what's that in kilometres roughly?

Respondent: It's about 14 kilometres.

Interviewer: OK. That's a nice distance actually.

Respondent: Uh-huh, it's...

Interviewer: It's not too bad at all.

Respondent: It's quite good. Uh-huh.

Interviewer: OK. So in research with living... well with women in urban areas, they often refer to their local neighbourhood and I'm interested in understanding whether the word... what the word neighbourhood might mean for women who live in rural areas and indeed whether it's relevant. So if I asked you what the word neighbourhood meant to you, what would you say?

Respondent: Neighbourhood. Well we kind of do have a small community where we are because although we're living rurally we actually have some houses that are scattered around us. So it probably... it's probably not neighbourhood as you would think in the urban area but there still is a little neighbourhood. And even one of our neighbours who's probably a kilometre and a half away type thing is still sort of might contact or talk with that person on a monthly basis, which is a monthly basis not abnormal for me.

Interviewer: That's quite often for it.

Respondent: Uh-huh. Our neighbourhood, I think most of the people are fairly private people where we live, but if something needed doing in the community or around us together or for... recently my husband had an accident and so all the neighbours were calling in to say look if we need, if you need a hand with lifting or different things we'll come and...

Interviewer: That's great.

Respondent: ... help. So there's a little bit of neighbourhood for the kids that they go and play at the neighbours, there's not a lot of that sort of interaction because there's a... like there's different sort of

generations. There's people that are retired and then there's people that have chosen not to have children and yeah.

Interviewer: So if you... if I asked you to think about a distance around neighbourhood what would you say it would be?

Respondent: Well for us, we're actually... so we're rural in a pocket. We've actually got a neighbour that would be probably 50 to 100 metres away.

Interviewer: Uh-huh.

Respondent: So quite close.

Interviewer: That's close. Uh-huh.

Respondent: And then we've actually got some other neighbours that would probably be a hundred and fifty metres away. And then we've also got some other neighbours that are probably about a hundred and fifty. So we've actually...

Interviewer: You are yes.

Respondent: We're actually very close. We're closer than where we were previously and closer than what some people living sort of rurally in town. And then we've got some other neighbours who we've got a lane where we walk through to their property and they do the same for us, who are probably maybe not quite a kilometre.

Interviewer: OK. And what about the word community, what does that mean to you?

Respondent: Well the word community is like a connectedness and a commitment towards some common values and... but I've got to be honest, at the moment I don't feel I contributed to our community, just through being very busy and not... being time poor. Uh-huh.

Interviewer: But that happens in life and then times of your life.

Respondent: Uh-huh. But I do think where we are we're very close to [town] so at [town], but we're also bordering [town] and there's like a community hall and they used to do... there were some great people that would put Christmas functions together and a few different community sort of events, but yeah, as I said at the moment I probably don't contribute as much as I'd like to. Uh-huh.

Interviewer: But your sense of community is about sort of some common linkages between people?

Respondent: Uh-huh.

Interviewer: And that you mention there's also a physical focus in terms of a hall.

Respondent: Uh-huh.

Interviewer: If you put a distance around that, what would it be?

Respondent: It would probably be, probably ten K radius.

Interviewer: Uh-huh. So that's a bit, yeah. OK. So that's... that would make the difference between neighbourhood and community too.

Respondent: Uh-huh.

Interviewer: Uh-huh. OK. No, that's really clear. Thank you. Now I want to talk a bit about physical activity.

Respondent: Uh-huh.

Interviewer: And as I said it's a very poor term. So what does the term physical activity mean for you?

Respondent: Well for me it means physically being active on a daily basis. I...

Interviewer: So as part of your life.

Respondent: It's a part of my life in a big way. Probably to an extreme way at the moment. As we breed horses we actually do endurance horse riding, so in the last two months I've done 340 kilometres of competition so it's a daily basis. I get up at 5:30 in the morning, I put a headlight on and out I go training...

Interviewer: Wow.

Respondent: ... for probably an hour most mornings.

Interviewer: Do you do that on your property?

Respondent: We do that... yeah on my property. I'm fortunate we can go down and do a ten K loop, yeah, on our property. Uh-huh. Other activity, physical activity for me, I work in [city], so I drive to [city] and I have to park my car about 15 minutes from I work so it's probably a kilometre walk into town, so that. And then just walking around the property doing things. So there's a lot.

Interviewer: There's a lot indeed.

Respondent: Uh-huh.

Interviewer: So do you consider yourself to be physically active?

Respondent: Uh-huh.

Interviewer: Uh-huh. OK. And you've told me why. You're very busy in your life. And where does physical activity fit in your life, is it a priority, a high priority?

Respondent: Well yes it is. It's part and parcel of where we live too, because we've got to walk up and down the property and around the property and...

Interviewer: Is it in a fairly undulating country...

Respondent: In places yeah, it's flat and then it's also undulating. And because of our sporting interest and our business interest, yeah, so...

Interviewer: That's a pretty high priority.

Respondent: Uh-huh. Absolutely.

Interviewer: OK. Thank you. Now in our research, when we think about physical activity we think about any activity that lasts for at least ten minutes, that's the health benefit, and causes our bodies to... you can feel a bit warmer, a bit of huffing and puffing, our heart rate goes up. So just thinking about this type of physical activity over the last two weeks, and I'll just go through parts of your life, not that I want to divide up your life, but it's more about so we don't miss things that's all. So in the past two weeks have you been active at all at work for at least ten minutes at a time?

Respondent: So once I actually start? Like when I...

Interviewer: No, it could be walking to... as you said you park the car and walk.

Respondent: Uh-huh.

Interviewer: So that would definitely come into it.

Respondent: It would, yes.

Interviewer: As well as being at work.

Respondent: When I'm at work that's often if I've got appointments and things, taking the stairs sometimes and sometimes not. It's kind of running in-between appointments otherwise I'm basically at a desk.

Interviewer: Uh-huh. It's a very common one these days isn't it?

Respondent: Uh-huh. I spend a lot of time at a desk so the other kind of really balances that out for me.

Interviewer: And thinking about the two activities you've mentioned, the stairs and parking the car and walking to work, what would be the intensity of those two activities? Would they be steady, moderate, vigorous?

Respondent: OK. When I walk into work by myself, vigorous, because I want to get there as quickly as I can, if I could run without sweating so I didn't arrive to work...

Interviewer: I know. That's the other catch isn't it?

Respondent: Uh-huh. I would probably do a slow jog and that, but I need to... I've got to be presentable to be around people, so yeah, I would say vigorous. I can feel it in my shin bones like pressure on them. I guess my heart rate would elevate a little bit.

Interviewer: And duration, how long would those activities have been for?

Respondent: Ten minutes morning and night at work.

Interviewer: Uh-huh.

Respondent: Uh-huh. To fifteen, just, yeah, just depends a little bit, yeah.

Interviewer: And how often would you do that?

Respondent: I do that at least four mornings and four nights a week, yeah. Unless it's raining. And I park in... I pay for parking.

Interviewer: And when? So that would be before work and after work?

Respondent: After work, yeah. So about quarter past eight of a morning and about quarter past five, five o' clock of a night time.

Interviewer: And is this what you usually do or was it one off?

Respondent: No. This is my routine as of this year.

Interviewer: Uh-huh.

Respondent: Uh-huh. As of this year.

Interviewer: And does it change depending on the season and the time of year?

Respondent: Uh-huh. If it's wet, no, I can't end up looking wet and...

Interviewer: That's a fair comment. Fair enough. And if you'd wanted to, is there anything that would have helped you be more active at work?

Respondent: The fact that I'm... my office is on the fourth floor.

Interviewer: OK.

Respondent: I get to make a choice whether I take the lift or the stairs, otherwise there's no incentive to go to the gym as part of our work programme, or that I'm aware of anyway.

Interviewer: OK. And in the past two weeks, have you walked or cycled for at least ten minutes at a time to get to and from places?

Respondent: Yes.

Interviewer: OK. And which one?

Respondent: I've walked.

Interviewer: OK.

Respondent: I do like to cycle but I haven't been on a bike at all this year, I haven't had time.

Interviewer: Uh-huh. Fair enough. And can you tell me about what you did?

Respondent: I walked into work, yeah, and back. Of a morning when I go training with the horses I walk down for a kilometre and a half because it's all

downhill, so to take the pressure off the horses legs I tend to walk, yeah.

Interviewer: And I'm happy if you want to talk about riding here, or we can talk about it in your leisure and spare time, but I mean that's the other very vigorous activity and certainly if riding fits into that category.

Respondent: Uh-huh. The riding. I did an 80 kilometre, not this weekend but last weekend, so that was on the 21st of May. So that was... I was riding for seven hours, it was pretty constant 'cause you're using a lot of your core muscles.

Interviewer: I'm very impressed. I mean I would... I used to ride just a little bit but I you know, your whole body would sort of ache after...

Respondent: It does. The last ten K's like your body's aching and you just want to get off. You do. But I must be getting a little bit fitter or body is coming a little bit more used to it because like the next day or so I barely notice it, I've actually done it now. Whereas if I have a big gap I'm always quite sore, but...

Interviewer: Uh-huh, that's often the worst bit isn't it? It's how long you're sore for, so...

Respondent: Sore after, yeah. Now I'm sort of, as I said well I actually went in to work after I did the last ride and yeah, there was no worries.

Interviewer: OK. So thinking about those activities...

Respondent: Uh-huh.

Interviewer: ... the walking and the riding, could you describe what intensity they may have been?

Respondent: I would...

Interviewer: Whether it's steady, moderate or vigorous and riding...

Respondent: Uh-huh. I was actually thinking moderate. I wouldn't put it a high intensity, sometimes I do get off and run. And it doesn't... like it's more of... more muscular. It's not so much cardio vascular workout although you've got to be relatively fit to sort of do it because you're not walking.

Interviewer: Well it's the time you're doing it for too.

Respondent: Uh-huh. So I'd say a moderate. Uh-huh.

Interviewer: OK.

Respondent: I guess it borders into high intensity, we're going to do a hundred and sixty kilometre in a few weeks time.

Interviewer: Oh I would have thought so.

Respondent: Uh-huh.

Interviewer: Uh-huh.

Respondent: It's just if I went to do a race or a sprint I would really probably struggle and I'd puff, so it doesn't actually, yeah, my lung capacity and cardio vascular sort of...

Interviewer: True. That's often... it's also often about the type of activity we're used to doing. If you swap activities it always take a while to build up.

Respondent: To adjust, yeah. This... when I'm doing the riding and that, particularly at the moment, like it certainly does make me feel better. I'm far more energised and my workload at works huge and home and to fit it all in, like having that fitness I think...

Interviewer: It helps you.

Respondent: It helps me.

Interviewer: Uh-huh. Absolutely. OK. And the walking, what intensity would you call... would call that?

Respondent: Uh-huh, I'd say that's moderate. Like I don't generally... I don't kind of walk at a steady pace, I'm walking 'cause I want to get there, I want to be there now.

Interviewer: Exactly. OK. And the duration, how long would you have done those two activities for?

Respondent: On a daily basis?

Interviewer: In the last fortnight. So you would have done them every day?

Respondent: Uh-huh.

Interviewer: OK.

Respondent: I did have two days off which was probably the first in six weeks, but yeah.

Interviewer: And you were saying, how long would you spend in the morning riding then?

Respondent: I try and do anywhere between a six to ten K loop in the morning. So I'm riding for probably an hour. Sometimes it's only at a very steady pace because it's dark and then sometimes a little bit faster. I sort of, yeah, it's more of a conditioning; I'm conditioning some young horse at the moment.

Interviewer: So that would be... and you were... have... you were also saying about doing a longer endurance ride as well.

Respondent: Uh-huh, in that as well. So the day I did the longer endurance ride I was riding for seven hours. The next day normally I would have come home and then trained another horse, but I had two days off. The day prior to the event I actually trained another horse, so I went for an hour to an hour and a half. Uh-huh, and then I'm still doing my four days or four mornings of walking.

Interviewer: Walking as well when you get...

Respondent: For work, yeah.

Interviewer: Uh-huh. And so, OK. Now I'll just get the frequency of all these activities. So there's four mornings of walking.

Respondent: Uh-huh.

Interviewer: There's most days of exercising horses during the week.

Respondent: Uh-huh.

Interviewer: And the weekends?

Respondent: Uh-huh.

Interviewer: OK.

Respondent: Sometimes twice in the one day...

Interviewer: So that would be every day...

Respondent: ... and then in the night.

Interviewer: ... it would average out to would it?

Respondent: Uh-huh at the moment, yes.

Interviewer: And then you've had a couple of endurance, longer endurance rides, two of those.

Respondent: Just in the last fortnight I've just had the one, but since March I've actually completed three 80's and one 100 kilometre ride.

Interviewer: I'm impressed. You must be so fit. That's hard work.

Respondent: Uh-huh.

Interviewer: I know. I find horse riding, you feel it.

Respondent: Uh-huh I think I've...

Interviewer: And it's the terrain and...

Respondent: Uh-huh. I think I've... I must have just, yeah. My body's just got used to it again this year. I had last year off riding and [inaudible 20.49].

Interviewer: And [horse] are lovely, but they are a bit bouncy.

Respondent: Well see my [horse] are like, one [horse] 15 three.

Interviewer: Ah uh-huh, a decent size.

Respondent: So big, big horse.

Interviewer: Uh-huh, so big stride.

Respondent: Big stride.

Interviewer: Makes a difference.

Respondent: And then I've got another one that's 14 three but a good stride, they've all... they're not super bouncy, they're big, pretty big striding, the one's...

Interviewer: That makes...

Respondent: ... I've got, yeah.

Interviewer: A big difference.

Respondent: 'Cause bouncy and small, sore knees, sore yeah, sore shoulders.

Interviewer: Uh-huh. OK. Now, OK. So when, what time of the day would you have done those activities?

Respondent: The horse riding during the week, 5:30, six o'clock. Weekends, 10:30 in the morning.

Interviewer: Sounds...

Respondent: Eleven o' clock, daylight and...

Interviewer: Makes a big difference.

Respondent: Uh-huh, certainly does, late mornings.

Interviewer: I'm amazed you can ride endurance in the dark, it's just amazed me.

Respondent: Oh it's like horrible and, yeah. Good headlight.

Interviewer: I think you have to. And, OK. So... and you've told me where you've done it, and why did you do it there? What attracted you to doing it there?

Respondent: So the training...

Interviewer: The training.

Respondent: ... of the horses. Because it's just... I guess it's a means to an end of the longer term goal.

Interviewer: And you do it on the property?

Respondent: I do it on the property.

Interviewer: Because rather than other places because...

Respondent: Uh-huh. It’s convenient.

Interviewer: Uh-huh.

Respondent: If I had the chance I’d like to go to the gym and do some other types of activity but this is about all I can stretch to.

Interviewer: I’m amazed if you had the time. OK. So were you alone or with others when you did these activities?

Respondent: Training, mostly alone. Competition, with others.

Interviewer: Uh-huh. OK.

Respondent: Walking to work sometimes with others.

Interviewer: And is this what you usually do or was it one off?

Respondent: At the moment it’s, yeah, it’s just usually I’ve been doing this since March.

Interviewer: What you usually do?

Respondent: Uh-huh. Last year I didn’t, but this... previous to that yes.

Interviewer: And does it change with the season or the time of the year?

Respondent: Uh-huh. It’s definitely... I’m probably a lot more active in summer although I’m having a fairly active winter.

Interviewer: It sounds as though, yes.

Respondent: Uh-huh. But I'm looking forward to not getting up, not going out in the dark. I sort of don't like that part at all.

Interviewer: Well we've only got... I'm counting how many weeks to the winter solstice which means the shortest day, and then we start returning... getting our life back and it's not that much to look at.

Respondent: It's not far, no.

Interviewer: It's only a few weeks. Three weeks.

Respondent: Uh-huh, that's good.

Interviewer: Uh-huh. And if you'd wanted to, is there anything that would have helped you walk or ride to and from places, if you wanted to do more?

Respondent: Having more time.

Interviewer: Uh-huh. Basically.

Respondent: Having more time.

Interviewer: OK. Now in the last two weeks have you been active around the house or yard? So either inside or outside.

Respondent: Uh-huh.

Interviewer: For at least ten minutes.

Respondent: Uh-huh. Feeding up horses, checking lambs, mopping floors, yeah.

Interviewer: And so thinking about those activities, what intensity would you say they were? Steady, moderate or vigorous?

Respondent: Steady. Uh-huh. Steady.

Interviewer: OK.

Respondent: Sometimes vigorous, depends how I'm going for time.

Interviewer: And duration. How long might you have spent? It's always hard to put a time on that.

Respondent: Uh-huh. It is hard.

Interviewer: 'Cause you would feed up and do all the horse activities and feeding the livestock every day I would be thinking.

Respondent: Uh-huh, that is a day thing. I tend to do that just after I've ridden. And then like inside and stuff like I probably spend, I don't know, two, three hours doing housework the other morning, four hours maybe. And then the rest of the time it's just band aid, putting things away, just... I'm on the go most of the time until I go to bed.

Interviewer: Uh-huh, I bet.

Respondent: Uh-huh.

Interviewer: So how long do you spend sort of feeding up, doing that every day.

Respondent: Probably ten to 15 minutes. Uh-huh.

Interviewer: OK. And when did you do these activities?

Respondent: On a daily... every day. Morning.

Interviewer: And... early morning.

Respondent: Uh-huh. Sometimes night. I really don't like, don't want to come home to go back out in the dark.

Interviewer: Uh-huh. And why is the time factor again, before and after work I presume? Uh-huh.

Respondent: Uh-huh.

Interviewer: OK. And housework. When does that get... when were you doing that?

Respondent: Well the housework, that was Saturday morning. As I said I probably spent, yeah, three to four hours just mopping and vacuuming and dusting and getting the sheets out, and then yeah, just the daily grind stuff. Before work, after work.

Interviewer: And the activities, would... who would you do those with?

Respondent: The housework?

Interviewer: Uh-huh.

Respondent: With the kids, my husband, he helps out a little bit. Uh-huh.

Interviewer: And the feeding up?

Respondent: Uh-huh. Husband and my kids. Uh-huh.

Interviewer: And is this what you usually do or was it one off?

Respondent: No, it's what we usually do.

Interviewer: Uh-huh. And does it change depending on the season or the time of the year?

Respondent: Occasionally. If it's wet I'm reluctant, or I palm it off to my husband. Uh-huh.

Interviewer: But yeah, usually it doesn't change that much.

Respondent: Uh-huh it probably... depends on what else is happening. My husband had an accident so I was probably doing a fair bit more.

Interviewer: You were ending up have to do more?

Respondent: Uh-huh.

Interviewer: Uh-huh.

Respondent: So yeah, so that's probably a little bit unusual.

Interviewer: OK. And if you'd wanted to, is there anything that would have helped you be more active around the house or outside in the yard?

Respondent: Not going to work.

Interviewer: Uh-huh, having more time. Absolutely. And have you been physically active for at least ten minutes during your leisure or spare time? So

this is chance if we've missed out on any of your, if there are any other time in your day.

Respondent: It's not at the moment, I work six days a week, sometimes seven, and then, yeah.

Interviewer: And if you did you'd probably want to just be able to relax a bit probably.

Respondent: I'm looking forward to... we're actually meant to be going away for a few months, so that's...

Interviewer: Oh right. OK.

Respondent: ... that is my dream.

Interviewer: That's your dream. That's yeah, keeping you going at present.

Respondent: Uh-huh.

Interviewer: That sounds great. OK. That's fine. As I said it's just to make sure that we pick up all of your activity that's all.

Respondent: Uh-huh.

Interviewer: Now the last bit's about physical environments. We talked about where you live and a bit about your activity. And this is about your physical environments when you work and live and play. And when we talk about physical environment I mean all the physical things that surround you, like roads, trees, houses, traffic. So... and some of these things are things that people living in urban areas have said that might be important to their physical activity, so I wanted to ask you

whether they did... they're relevant to you. Now can you tell me a bit about how easy or difficult it is to be physically active in your neighbourhood?

Respondent: In my neighbourhood, if I wasn't doing the horse riding it would be extremely hard to be physically active because I can't [inaudible 29.13] road, it's pretty dangerous.

Interviewer: There's no...

Respondent: There's no side.

Interviewer: There's no side. There's obviously no footpath.

Respondent: Uh-huh.

Interviewer: And...

Respondent: And cycling, last year I was doing a bit of cycling and stuff. And you're always a little bit dubious. Like my kids like to cycle out there with me, whereas where we are we generally like to pack the bikes up and bring them down into [town] and go on the cycling track out to [x] Beach or somewhere because it's just... it's a little bit dangerous the environment.

Interviewer: 'Cause I was going to say where would you be able to cycle where you live?

Respondent: Uh-huh, it's really hard. I generally do like... I don't know, I think it's a three K loop, four K loop when I go, sort of down one way and then huff and puff on the way back up.

Interviewer: So is that on the main route?

Respondent: On the main road.

Interviewer: On the main road.

Respondent: Uh-huh. So that's always... like I couldn't do it of a night time, I couldn't do it at the moment.

Interviewer: No.

Respondent: Uh-huh. And the same like with the horse riding. If we didn't have the property that we have there's no way I could even do that. We'd have to pack the horses up into a float and...

Interviewer: Uh-huh, be it just from road safety point of view?

Respondent: Uh-huh.

Interviewer: Be just too dangerous to try and ride them on the side of the road, let alone for you to walk or to cycle.

Respondent: Uh-huh. Absolutely.

Interviewer: OK. So what make it difficult is the road traffic situation of the road.

Respondent: Uh-huh. Absolutely.

Interviewer: And so what happens is that you would general... either use your property for your activity or you drive to your activity?

Respondent: Uh-huh.

Interviewer: Uh-huh. OK. Put the bikes, load the bikes up.

Respondent: Uh-huh. And just where we live. Like even if you wanted to go to the gym and stuff you just wouldn't 'cause it'd be that 14 kilometres, it would be like well I've just gone home. If I wasn't doing what I do...

Interviewer: You wouldn't want to turn round and go back again.

Respondent: Uh-huh. Not at all. Even though you might... yeah I'd like to go and do that and be, yeah, you wouldn't.

Interviewer: OK. Now I just want to ask about a couple of things.

Respondent: Uh-huh.

Interviewer: The first ones availability and accessibility of places to be active. So if I were to ask you about places to be active in your area...

Respondent: Uh-huh.

Interviewer: ... what sorts of things would you tell me about?

Respondent: I would tell you about the cycling track from [town] to [x] Beach, I think that's been amazing. I would tell you about the... there's a pool, an indoor pool.

Interviewer: Is that in Ulverstone too?

Respondent: Uh-huh, that's at... it's probably the only... it's the only indoor pool on the coast I think, well a community pool.

Interviewer: Is that open all year round?

Respondent: It’s not. And it’s... it is hard.

Interviewer: It’s seasonal.

Respondent: I couldn’t actually just turn up on the door either, you’ve got to be sort of accessed through a swim club or through a surf club or, yeah.

Interviewer: OK.

Respondent: Uh-huh. I’m pretty sure I couldn’t just turn up on the door.

Interviewer: OK.

Respondent: I’m not sure. What other... we’ve got a basketball stadium.

Interviewer: [town]?

Respondent: [town] got a big basketball stadium so if I wanted to be active... well if I was into playing basketball there’d be the opportunity there.

Interviewer: Parks, walking tracks?

Respondent: Uh-huh, there’s some really good walking tracks.

Interviewer: And we’re talking about [town] again aren’t we?

Respondent: Uh-huh.

Interviewer: So the picture I get is around your area there isn’t, you’d have to get in your car and drive ten minutes to [town]?

Respondent: Uh-huh. Definitely.

Interviewer: OK.

Respondent: And that is a deterrent. Uh-huh.

Interviewer: And... so there isn't anything immediately in your area. And access, are they easy to access in terms of cost or opening hours and...

Respondent: Opening hours. I couldn't tell you to be honest because..

Interviewer: It's a bit of a question on the pool isn't there?

Respondent: Uh-huh. And even the same, like with some of the other, like the gyms and things that are in [town] because to be honest I get home and the thought of just heading back out these days is just like I'm not going anywhere.

Interviewer: No. Busy enough as it is. OK.

Respondent: Well yeah, the only way I would do that is like if I do it for the kids but not for personal.

Interviewer: Uh-huh. No. That's OK. No, that's fine. So does having places to be active influence whether you're active or not?

Respondent: If I wasn't doing the horses absolutely.

Interviewer: Uh-huh.

Respondent: Absolutely. Uh-huh. When I was studying at uni if I had access to a gym I would have been using it like, but being remote I couldn't even

consider, yeah. So if you've... you know like... and if I was living in town you might hook up with some other people and go for a walk or a run, so yes.

Interviewer: And if you wanted to be more active are there things or places that would help if they were available or more accessible?

Respondent: Uh-huh, I think you know if our community... we've got the hall [inaudible 34.30] and like if there were some sporting classes or just some aerobic classes. Uh-huh I'd probably look at attending something like that just to get some...

Interviewer: In your local community?

Respondent: Uh-huh.

Interviewer: How far would that be away?

Respondent: That'd be like three K's for me.

Interviewer: Uh-huh.

Respondent: So it's be much closer and the thought of going out on a wintry night, I'd actually contemplate, whereas to go back 14 K's down, no.

Interviewer: OK. Now destinations. If I were to ask you about places that you could walk or cycle from your home, what sorts of things would you tell me about?

Respondent: To walk from home?

Interviewer: Now we've already talked about that in terms of safety, road safety, it wouldn't be a goer.

Respondent: No.

Interviewer: But in terms of destinations, are there any sort of local shops or recreation facilities or friends houses or...

Respondent: Uh-huh. The community up at [town], the community hall and stuff. The kids and I go... they've got a tennis court, we have actually put the tennis racquets on the back of our backs or [inaudible 35.50] and cycled three K's up the road to the tennis courts. Basketball court up there.

Interviewer: Is that safe to ride up there?

Respondent: It probably is a little bit dangerous. Like it's a country road so in places it's a little bit straight and we're, yeah, we're...

Interviewer: So is this a different road than the other road that you were talking about?

Respondent: Uh-huh. If I was heading down in to [town], yeah.

Interviewer: Uh-huh. That's the more main road is it?

Respondent: Uh-huh.

Interviewer: With more traffic and less...

Respondent: Well more windy, just windy, and coming up because like it's steep whereas to head up to [town] it's not quite as steep. But it's still... it's

certainly a risk. Is something that we would do, you've got... we couldn't do it of a night time or you've got to pick when you do it. Uh-huh.

Interviewer: So that's sort of a bit of a quieter road and it's not as windy?

Respondent: It's not as windy. So the fact that you're straight you can get across. Whereas when you're on a windy road people cut corners so you've just got to be really conscious. So at least this one it's kind of straight, there's some good lengths of straight, yeah.

Interviewer: OK. And does having, or in this case not having destinations influence whether you're active or not?

Respondent: Uh-huh.

Interviewer: 'Cause you were saying if things were... if there were things closer then that would be good.

Respondent: Uh-huh. Absolutely.

Interviewer: OK. And if you wanted to be more active, would it help if there were more destinations within easy walking or riding distance?

Respondent: Uh-huh.

Interviewer: OK. Now a couple of more areas, one's neighbourhood design, that built features of the environment, or the manmade features or the non natural features. If I were to ask you about those, what would you tell me about in your area? What's the built features? Now we've talked about a few things, the lack of footpaths, there's main roads...

Respondent: I'm only thinking of the negative at the moment.

Interviewer: No, no. That's fine. Do. What are you thinking of?

Respondent: We don't have rubbish collection or...

Interviewer: Uh-huh.

Respondent: But we have natural features.

Interviewer: I'm going to ask you about those too.

Respondent: Manmade features, we've got a road.

Interviewer: You've got a road.

Respondent: We've got a road. We've got power.

Interviewer: And lighting?

Respondent: Lighting, no.

Interviewer: No. And no foot...

Respondent: No.

Interviewer: No footpaths.

Respondent: No.

Interviewer: And walkability, no. We've talked about it's a bit too dangerous. And the other one is often for walking you need streets to connect to each other, rather than just a long linear strip.

Respondent: That's it, yeah.

Interviewer: Which one have you got?

Respondent: Well if we could have a little connecting street, this is a lane. There is a road that sort of goes down either side of our property, and then we'd have to come back up through our property.

Interviewer: OK.

Respondent: Uh-huh. So we could sort of do that, but it's very steep. So that's a deterrent.

Interviewer: OK. And do the built features influence whether you're physically active or not? Or lack of them in this case?

Respondent: Uh-huh, look it would. Uh-huh it would do because...

Interviewer: You've already talked about that a bit too.

Respondent: If there was a footpath or something hidden like, yeah, you'd you know, might get out there on the skateboard, who knows.

Interviewer: And if you wanted to be more active, are there any built features in your area that would help?

Respondent: Uh-huh. Footpath.

Interviewer: Footpath did you say.

Respondent: Lighting.

Interviewer: Lighting. Uh-huh. OK. Now the other side of it is the natural environment, the aesthetics. If I were to ask you about the aesthetic qualities or the attractiveness of your area, what sorts of things would you tell me about?

Respondent: Oh look, when you go down our property you can actually see the ocean, so we're 14 kilometres out to actually see the ocean, so that's really nice. We actually back on to the main water supply of [town], so there's a river that runs down through, so it's there if in summer if we wanted to go fishing, we can actually do, that's where we train the horses. I could motor bike if I so desired, so it does have some really good features. And just the natural beauty...

Interviewer: It sounds very beautiful.

Respondent: ... to walk a dog or whatever, like open spaces, safe. Uh-huh.

Interviewer: OK. And do you think your local environment is aesthetically pleasing and attractive near your home?

Respondent: Uh-huh.

Interviewer: Absolutely. Uh-huh.

Respondent: Uh-huh.

Interviewer: And do the aesthetics of your area influence whether you're physically active or not?

Respondent: Uh-huh. 'Cause it's nice to get out if it's a sunny day and have a look, and just absorb it all. If you're highly stressed just to go for a walk and just let it... let it all go.

Interviewer: And if you wanted to be more active, are there features of your area that might help if they were more aesthetically pleasing or attractive?

Respondent: Natural features, probably not.

Interviewer: No, 'cause they're beautiful as they are. Uh-huh.

Respondent: Uh-huh.

Interviewer: Just one last area is safety. You've touched on these... this already. If you... I were to ask you about safety in your area, what sorts of things would you tell me about?

Respondent: Safety as far as people predators, there's a lack of, you don't have to worry too much about people predators. We have to worry about snakes which is, that's...

Interviewer: That's a different one.

Respondent: It's a very much concern, five snakes a day or so by the time you go down through to the ten K in summer, lots of water. Safety, I guess like you were talking about walking on farmland and stuff too, so it's at times you can be at more risk to injury just with our natural environment. Uh-huh, just the farming environment, I guess there are safety issues, chemicals. There's people, you go out walking and you have a helicopter come over and things like that. So you're faced with chemical sprays.

Interviewer: Oh spraying.

Respondent: Uh-huh. Actually I've actually had happen, they're meant to actually give you a notification of things like that, so it's...

Interviewer: So that's the other side of it, yeah. It's on the personal safety.

Respondent: Uh-huh.

Interviewer: And road safety. Does road safety influence whether you're physically active or not?

Respondent: Uh-huh.

Interviewer: And we've already talked about that.

Respondent: Uh-huh.

Interviewer: And if you wanted to be more active, are there personal or road safety issues that if they were addressed might help you be more active?

Respondent: Uh-huh. The footpath thing.

Interviewer: The footpath, uh-huh.

Respondent: Would certainly help, or just a...

Interviewer: A safe place.

Respondent: ... safe area to get off.

Interviewer: Uh-huh.

Respondent: Uh-huh. I mean some places are safe and then others you're just really exposed.

Interviewer: Oh well that's interesting, so in terms of just getting better continuity of making it, if there are some places that aren't so bad, it's actually sort of bringing the others up to standard so. Uh-huh. OK. And just to finish, is there anything else you'd like to tell me about where you live or your physical activity or your environment that we haven't touched on today?

Respondent: Oh I think that's probably... I think we've probably covered it pretty well.

Interviewer: OK.

Respondent: Uh-huh.

Interviewer: Thank you very much.

Interviewer: So today I was wanting to hear your thoughts about a range of issues related to where you live and how this might be related to your physical activity.

Respondent: Uh-huh.

Interviewer: So I guess the important thing to remember is we're really interested in what you think. We don't, we're not into a right or wrong approach.

Respondent: No.

Interviewer: There's no right or wrong answers here.

Respondent: No, no.

Interviewer: So we'd like to hear about your experiences and your thoughts and your feelings about things.

Respondent: Uh-huh.

Interviewer: And if there's anything you're unsure about just ask. Likewise if there's anything you don't want to answer…

Respondent: Yep, I can say that too.

Interviewer: That's fine too, absolutely.

Respondent: OK, alright.

Interviewer: So just to start generally a bit more about the place you live.

Respondent: Uh-huh.

Interviewer: So it's sort of thinking about where you live and if you could tell me about what it's like living around where you do? And you're in [town] aren't you?

Respondent: I'm in [town] I've just recently changed house, I was living up, oh you won't know [town] yet, but anyway I was living five minutes away from where I am now. So I've moved into a house which is in a place called [x] Road which is, the house is maybe two kilometres from the closest little shop, two to three kilometres from the main area of

[town] which is not far at all. And it's a very rural area, all the properties around my place, all my adjoining neighbours are all on two or three plus acres.

Interviewer: Do you live on similar sort of acreage?

Respondent: Yes my house has two and half acres with it and there is still some orchard being farmed just near where I am. Most people have got their little vegie gardens around me and back behind the orchard and when you get to the outskirts of where the few houses are is forestry plantation, either eucalypt or pine tree and then it goes out into all forestry land and/or world heritage area.

Interviewer: Wow, so how long have you lived in that area?

Respondent: I've lived in [town] for the last, about the last 15 years I think, yeah. I did live a year before that, before that I lived for a year in [city] and I have lived a year in [city] but I was actually born in [state] and raised in [region].

Interviewer: Oh OK, so what attracted you to [town]?

Respondent: When we were moving back, we moved to [town] from [town]. When we were due to come back to Tasmania; [city] was only, we went to [city] for a year, [city] was only going to be a temporary thing; when we were due to come back to [region] my Dad was living in [town] and he was ready to sell his house and he was willing to sell it to us at a very nice price so we bought that from him and we wanted to be in the country anyway and I needed to be somewhere where I could easily access paddocks for my horses and so we bought this house off Dad and I had paddocks, I rented paddocks nearby and then we bought acreage ourselves just up the road and sold the first [town]

house. Lived in a shed while we built our own house and lived there for many years and then my partner and I recently split up and he has stayed almost right on the same property just in the hay paddocks that we had and he's going to build a house on that and I moved five minutes' drive away to another house, to a bought house.

Interviewer: So you've lived in the area for quite some time.

Respondent: Quite a while yeah.

Interviewer: About ten years?

Respondent: Well probably about 15 years I'd say.

Interviewer: 15, OK. And your likes and dislikes about living there?

Respondent: Well I've lived in [city] and [city] so I can compare living in an urban environment and I was raised in the [region], just in a different area in [region] so I come from the country background and I know that I much prefer living in the country and having the space and not having the neighbours up close and I don't need or want access to shops 24 hours a day seven days a week or anything else that is 24 hours a day and at this stage in my life I don't need access to medical care. On the other hand [town]'s got its own doctor's surgery now anyway which is fairly well functioning so in the future that's there.

My little girl [daughter] who's four, she's not sort of sickly or anything so again we don't need access to anything. There's childcare there, I work full time, there is family day care available which is a day carer down there and her Dad and I you know we live five minutes apart so we're both nearby if we need to do anything. No I love living there.

Interviewer: Yeah, so did you, what’s your distance out of [town] itself?

Respondent: Oh it’s probably only about three kilometres I reckon.

Interviewer: Yeah OK.

Respondent: Yeah it’s easy walking distance anyway.

Interviewer: Fine, lovely. Now just going to, in research with women living in urban areas people often talk about their local neighbourhood and we’re interested in understanding what the term neighbourhood might mean for women who are living in rural areas.

Respondent: Oh right, yeah.

Interviewer: If it has meaning in fact. So if I was going to ask you that question I’d be saying well you know, think about it, so what does the word neighbourhood mean to you?

Respondent: Where I am now, because I’m completely in love with where I am now, I would say neighbourhood means that I can see peoples’ houses but they’re not close enough for me to see their faces if they’re out in the backyard. I can see their lights on of an evening, so I feel people are close enough if I have a problem but none of us are all on top of each other and I already, my Mum’s from [state] but my Dad is from [region] area and his brothers and what not and just about everybody who I’ve met who has property adjoining mine or up the road from me when I’ve been riding past on my horses, either knows or has worked with my Dad or one of his brothers in the past and a lot of them are older people and so I have very, I mean in my neighbourhood because people know my family through my Dad it’s very nice, you know I know there are people there who would help

me if I needed it but I don't need to have you know interactions with them.

I mean I rode past last weekend with some friends and my daughter and a lady digging potatoes was there and I knew who she was and so I stopped and introduced myself because I only moved in there a month ago and we had a bit of a conversation and she said next time you're coming past bring a bag and I'll give you some of these potatoes. And so a couple of days later I had a day off and I took some frozen raspberries from my freezer up with me and said here I'll swap you for your potatoes and she said oh no, no I've got a freezer full, you take the raspberries back and here's a bag of potatoes. And so for me it's already a little community…

Interviewer: Yes, yes.

Respondent: … and I seem to have been welcomed into it without people knocking on my door and pestering me.

Interviewer: Yes, yes. So if you needed to, like you were saying that in the neighbourhood you can actually see the lights of…

Respondent: Uh-huh.

Interviewer: If we're going to quantify it what…?

Respondent: What does quantify actually mean?

Interviewer: In terms of is there a distance in terms of how far…?

Respondent: You mean what is the physical distance to their houses?

Interviewer: Yeah.

Respondent: Well the closest neighbour is across the road and down a little bit and there's probably I suppose as the crow flies that's probably 150 metres.

Interviewer: Yeah.

Respondent: And the next closest one would be 250 metres.

Interviewer: Yeah. So that would be sort of that sort of range you'd see then?

Respondent: Yeah and then the next one again's probably you know, probably 400 metres yeah.

Interviewer: So up to half a kilometre away?

Respondent: Yeah, yeah, so as I say you can sort of easily see you know the houses and what not but you're not close enough to be looking over their fence or anything like that.

Interviewer: No. And the other word that we often use is the word community.

Respondent: Uh-huh.

Interviewer: What's that mean to you?

Respondent: Well [town], I think [town] is, [town] is an old town so you've got a lot of residents whose families have been there forever, and just like lots of other places around Tassie and Australia in general you've got a lot of people that have moved there in recent years from other states and even a few from overseas. So I think we're getting a fairly, we've

got a fairly interesting community and we've got some people who are very keen to do community things and organise community events and other people who just like to live there and do their own thing.

Interviewer: Yeah.

Respondent: And I think both those groups get on, there's no, doesn't seem to be any agro. I mean if people want to be ra, ra, ra and join a group well you can certainly do that in [town] but if you want to do your own thing then you can do that too you know.

Interviewer: And for you, what does it more mean for you personally?

Respondent: Just to be able to do my own thing and not necessarily join a group and you know [laughs].

Interviewer: Yep, yep [laughs].

Respondent: I don't need to know how to make jam or you know, I think I could manage that by myself yeah.

Interviewer: Fair enough [laughs]. And again has that got a distance factor with it?

Respondent: Well I guess most of the community activities are probably promoted by the [x] centre.

Interviewer: Yep. So that would be a broader…

Respondent: Which is down in town.

Interviewer: … in [town] itself.

Respondent: Yeah, which is, but still it's like a two minute drive or I could walk from my house down into [town] in probably 20, 25 minutes if I wanted to, so it's certainly no problem.

Interviewer: Yep, great. And so do you see that as a relevant term for living rurally, as a community?

Respondent: I don't really understand that question sorry?

Interviewer: Yeah it means something to you obviously?

Respondent: What means something to me?

Interviewer: The word community?

Respondent: Yes I guess so yes, well everybody has a community don't they, unless you live surrounded by nobody you do have a community, whether you take part in community activities or not you are still part of that community.

Interviewer: Uh-huh, fair enough. Now I want to move onto talking a bit about physical activities.

Respondent: Uh-huh.

Interviewer: Again there's no right or wrong when it comes to this one.

Respondent: No, no.

Interviewer: Because we're actually really interested in exploring with you, talking about what physical activity actually means to you. And that's an understanding that it means different things to different people.

Respondent: Yeah well I have my own definite ideas on that sort of thing anyway [laughs].

Interviewer: Well [laughs] what does it mean to you?

Respondent: Well it means, I think that it means looking after your body and other peoples' health and doing physical things, moving around and not you know over-indulging yourself in lying around or eating too much or looking for reasons not to do things but more just going out and doing things so as to be, because I'm sure it definitely makes most people happier and your own happiness contributes to other peoples' happiness and in the end you're going to be less of a burden on, you know society and what not if you are physically well and able, and really it's up to you to do it, not up to anyone else to tell you how to do it or hold your hand while you do it. I think it's up to people to take responsibility for their own health.

Interviewer: Sure, sure. OK, and do you consider yourself physically active?

Respondent: Yes.

Interviewer: And why?

Respondent: Well OK so, well I can tell you what I do I suppose.

Interviewer: Good.

Respondent: So I run, I run a couple of times a week, I run a couple of kilometres a couple of times a week and where I live it's great because it's such a quiet area I don't have to deal with traffic fumes, I don't have to deal with strange people around the place who I don't know if I'm out

running in the dark before work or after dark. I've got forestry a stone's throw away where I can get onto the tracks either by myself, with my friends or with my friends on horseback or on foot. So I've got the perfect environment for the exercises that I like to do.

I have a gym that I go to, we have a gym here in [town] I used to go two days a week in my lunch hour so that was really easy, just toddle down to the [community centre]. I don't do that anymore but I do go to the other gym which is the [town] Sports Centre which again is just a toddle along the road.

And so you know everyone down there is well served by these things and so I do a self-defence class once or twice a week and the horse riding. In the winter, normally I turn my horses out in the winter but this winter because I'm in a different spot with lots more riding close by I will keep working them so that means they'll get worked on the weekends. In the summer I ride every second night of the week as well as on weekends.

Interviewer: More light.

Respondent: Yes more light after work yes. It's all to do with light, you don't really want to go out horse riding in the dark. You can run in the dark but you don't want to stuff around with horses. Yeah so I do all those things and I think I'm very lucky where I am because I've got ideal surroundings for that.

Interviewer: Yep, great. OK, now where does it fit into your life?

Respondent: Well it's a big part of my life so if I wanted to take on a new activity or do some extra study beyond what I'm doing at the moment I would have to eat into, that would be the time I would have to eat into to do

something else so it is a big part. I mean I couldn't probably qualify what percentage of time that I would spend doing either riding or running or gyming or what not or walking so, but it would have to be a very large per cent of my waking hours.

Interviewer: OK, alright. Now I'm going to ask you a series of questions around physical activity just to get your ideas. Now in research when we think about physical activity one of, you know the sort of thinking is around any activity that can last for ten minutes or more…

Respondent: Right.

Interviewer: … which raises the heartbeat, you know gets a bit of huff and puff…

Respondent: Yeah.

Interviewer: … going OK. So given that definition, thinking about the last two weeks and the type of physical activity I just want to ask questions about parts of your life, the first one is about work.

Respondent: Uh-huh.

Interviewer: OK, so in the last couple of weeks have you been active at work for at least ten minutes at a time?

Respondent: In my job no but in my lunch hour which is part of my working day yes. We have a hill here called [x] Hill which is just south of the [x] Bridge…

Interviewer: Oh yeah.

Respondent: … and it goes up to a quarry and two or three girls and I each walk a couple of times a week in our lunch hour up [x] Hill up to the quarry and back and that's, we have 40 minutes for lunch and we are able to walk up to the quarry and back and have five minutes to get changed back out of our walking clothes.

Interviewer: That's great.

Respondent: Yeah, yeah it's great because it does get your heart rate up. It's not just a flat walk and yeah so we're lucky with that. And so I would do that in a normal week and which I would have done in the last couple of weeks two or three times a week.

Interviewer: And would you describe that as sort of moderate or vigorous exercise?

Respondent: I wouldn't describe it as vigorous for me because I'm fairly fit so no, but I mean another person would certainly, I mean a lot of people wouldn't go up there. So I would call it moderate.

Interviewer: OK, and the other one, you've said that you usually do that, does doing this depend on the season?

Respondent: No we take…

Interviewer: You manage to do that whenever there's…

Respondent: … umbrellas if it's raining.

Interviewer: Oh OK.

Respondent: Yeah we take umbrellas if it's raining and it doesn't even depend on having the two, the friends to do it with.

Interviewer: No.

Respondent: If any of us can't do it at least one usually still goes anyway.

Interviewer: Yeah, that sounds really accessible.

Respondent: It is yeah.

Interviewer: Great. OK, now if you wanted to at work is there anything that would have helped you be more active?

Respondent: No because, no I don't think our work here at the council, I don't think they would ever have the funds to spend on you [workplace] setting up a staff gym or something. I mean if there was a staff gym yes I'd probably use that but as to whether that would make me more active, no that would just take me away from the walk at lunch time so, and in my job as a [occupation] it's a sedentary job, you're sitting at a screen, you're inputting information all day and what not so I can't see how I could make the job, the actual siting in the computer hours, more physical no.

Interviewer: Yep, OK. Now in the last two weeks have you walked or cycled for at least ten minutes of the time…

Respondent: Yes.

Interviewer: … to get to and from places?

Respondent: Well to get to or from places no, I only do it for exercise so I don't use it as, I don't use walking, because I live in [town] so I'm not going to walk to work from there and I think it's too far to walk down to the shops and get a load of shopping and come back so, because it's downhill all the way and uphill all the way back and it's you know…

Interviewer: And let's face it shopping is heavy.

Respondent: Yeah it is. I used to. The other place I lived in I would walk down the road and get the shopping and walk back, that was only about a kilometre and a half away from the house. No, so I don't use it as a form of transport anywhere. I stop at the gym on my home in the car from [town] to [town] so I don't walk to the gym because, no I don't, my distances are too great to use it in that way.

Interviewer: Yep OK. So if you wanted to is there anything that would have helped you to use walking or cycling?

Respondent: Physical activity or whatever as a form of transport?

Interviewer: Yeah, yeah.

Respondent: No, I live in [town], my work is in [town], so I can't walk to work.

Interviewer: Yeah.

Respondent: The shops for shopping, for groceries, is just that bit too far for walking. The gym is too far for walking too, it's a good few kilometres away and I do that on my way home anyway. Yeah, no.

Interviewer: Fine. OK, now in the past two weeks have you been active around the house or yard at home, inside or outside?

Respondent: Yes because I’ve moved into this new house and so I still have boxes to unpack, I still have gardens to set up. I’ve made a composting corner in the garden. I’ve dug some strawberry beds in the other night to put the strawberry runners in.

Interviewer: There’s plenty to do.

Respondent: I’ve got horse poo collecting up from the paddocks constantly. I raked the lawn yesterday morning because I cut the grass and didn’t have time to rake it at the weekend so there’s heaps of physical stuff that I do…

Interviewer: Including…

Respondent: … inside and outside the house.

Interviewer: Yep, as well as the horses as well.

Respondent: Yes, yeah.

Interviewer: Which need sort of…

Respondent: Well they, when they’re not being ridden during the winter weeks they…

Interviewer: There’s plenty of care going on?

Respondent: Well not really, no you only have to rug and unrug.

Interviewer: OK.

Respondent: They are going on the grass in my two and a half acres and a few biscuits of hay so there's only a bit of hay to be taken out of the hay shed and thrown to them. So no they pretty much take care of themselves during the week apart from rugging and hay and of a weekend, yeah you don't do that much extra to get ready for a ride and come back from a ride.

Interviewer: OK, so the exercise you were talking about, how would you describe it's intensity? Moderate or vigorous?

Respondent: The exercise, like the physical exercise that I've done in the last few weeks?

Interviewer: Yes.

Respondent: Well the self-defence class that I do I would describe as intense.

Interviewer: Around the home?

Respondent: Oh around the home?

Interviewer: Yes because we'll get on to…

Respondent: Probably really pretty low impact really.

Interviewer: Yep.

Respondent: Yeah, I wouldn't even say moderate, I wouldn't say any of that really gets my heart rate up.

Interviewer: And for how long? You were saying you were out one evening strawberry planting.

Respondent: Yeah I did that on Sunday evening…

Interviewer: Mind you moving in is full on isn't it [laughs]?

Respondent: It is, yes it is, but there's nothing that gets your heart rate up, to me, and really doing the strawberry beds didn't get my heart rate up, so no none of that. I would say it's all been pretty much low.

Interviewer: OK, and you told me about when you did that so that's fine. And who with? Have you been…?

Respondent: Well I have my girlfriends that go horse riding with me and we do bush walking together, or are we still just keeping to the last two weeks?

Interviewer: Yeah just the last two weeks in around the yard?

Respondent: Oh no, no there's just myself and [daughter] my little girl who's four.

Interviewer: Yeah, and is there anything that might have helped you be more active inside and outside?

Respondent: No.

Interviewer: OK. Now the other part of it is what's often called your spare time or your leisure or whatever [laughs].

Respondent: Yes, yes, it's about that much, yes [laughs].

Interviewer: There all the parts of life [laughs]. So can you tell me what you might have done? That's when you were saying about bush walking and horse riding.

Respondent: Yeah so last weekend for example a couple of the girls and I drove to [town] with the intention to do, what's it called, [x]'s Peak. Do you know [town] at all?

Interviewer: No, well I know [town] but I don't know…

Respondent: OK, can you picture driving into [town] and you see a very pointy mountain in the background?

Interviewer: Yep.

Respondent: That's [x]'s Peak.

Interviewer: Wow.

Respondent: So I did that walk years ago with some friends and they drove the car years ago and I just went along for the ride and the walk and this time with my girlfriends here from work I was in charge of getting the driving instructions to get to the start of the walk and so I got the driving instructions from a guy who does a lot of walks in [town] off his blog site, and sadly we couldn't find the start of the track. So we drove around for an hour and then we decided well we're here anyway and there are a million small side roads off in forestry areas, spur this, spur that, so we just drove up one of the spurs, parked the car, locked it and got out and walked for two hours up and back, because the track just went up, up, up, up into the logging areas and had a walk anyway and so that's a leisure time thing.

And the other thing I did that day was stopped off and helped a friend with some of her gardening and stuff that she's doing in an old apple shed that she's renovating so that was sort of vaguely physical. And then the following day I went for a horse ride in the morning with another friend and I had a run early on the morning of the first day of the weekend.

So in my spare time I would normally be doing, in my leisure time I would normally be playing with the horses or having a walk, a run or reading a book or you know doing things with my daughter if it's the week that she's with me. Yeah that's what I would do.

Interviewer: Yeah, and so how would you describe the intensity of those activities?

Respondent: Some of them I would say are moderate. The self-defence class again is the one that is probably the most intense thing that I do, physical activity wise. But the horse riding is moderate activity and the bush walking and the running is moderate activity.

Interviewer: And the self-defence is what, once a week?

Respondent: Once sometimes twice a week.

Interviewer: Yep.

Respondent: For an hour.

Interviewer: And the horse riding's usually…?

Respondent: The horse riding at this time of the year is just on the weekends and that usually lasts for two or three hours either on the Saturday or the Sunday or both and that's moderate really.

Interviewer: And the run of course.

Respondent: And the run yes, that's only, that's half an hour here and half an hour there in the mornings.

Interviewer: Oh OK do you often do that in the mornings?

Respondent: Yeah before work.

Interviewer: That's a good time.

Respondent: Uh-huh.

Interviewer: It's a nice time of day.

Respondent: It is, it's lovely. It's very nice, and the dog comes too.

Interviewer: I was going to say…

Respondent: And the dog gets exercise.

Interviewer: … that's my dog walking time [laughs].

Respondent: Yes that's right yeah.

Interviewer: OK. The other one is where, we've touched on where, but what I've picked up is there's a mixture of fairly close around versus sort of in the broader locality for walks and things like that.

Respondent: Yes, so there are the places that we drive for bush walks because we want to go up mountains like [x]'s Peak or Mount [x], we've got really

a great place for walking living down here in the [region], we've got so many walks in so many areas that are very accessible. So there's that or there are, where I live there are close walks, close bush walks and there are forestry plantations, the same places where we bush walk close by are the places where we ride the horses. So same sort of areas.

Interviewer: So when you do your activities like walking, some of them, the further away you do need to get in the car?

Respondent: Yes.

Interviewer: Are there others that you can just walk from…?

Respondent: Where I live because it's a very rural area that backs onto forestry land, there are bush walks, they're lovely walks but the other ones are, no-one lives there, they're in the mountains, they're mountain bush walks because they're mountains so you driving to get to them.

Interviewer: Yep. And does it change depending on the season and time of the year?

Respondent: The regularity of the horse riding changes so all the daylight savings months I can ride several times a week as well as both days on weekends because the days are longer. It's just to do with the light, it's not to do with the warmth or the coldness because you can ride in rain and wind and cold it's OK. So it's all to do with the hours of light that you've got, for me, and then as far as your running goes or bush walking, the longer walks, the ones where we're going to be out for whole day like an eight hour walk we keep again for the daylight saving and the summer months, because really when your walking you want to be walking in a nice climate, you don't want to be

walking, horse riding’s OK but you don’t want to be walking for hours and hours in the rain and cold and it gets, when you do the big long walks up in the mountains the climate up there is quite different to the climate down in [town].

Interviewer: Yeah.

Respondent: And you can have snow coming over and nasty weather coming over quite quickly so we save those for the summer months.

Interviewer: Yes, that’s very wise.

Respondent: But the shorter walks, the one we were going to do Saturday and the one that we did end up doing Saturday, we can do those any time of the year.

Interviewer: Yep fair enough. And if you wanted to is there anything that would have helped you be more active?

Respondent: No.

Interviewer: In your leisure, spare time? It sounds like you’re very…

Respondent: No there’s nothing that helps me, no.

Interviewer: … active as it is?

Respondent: Yes.

Interviewer: And enjoy being so?

Respondent: Yeah and look I think that everybody can enjoy being active. I've never had any debilitating illness though and the only thing I've ever broken was one little finger on my hand so I can't speak from experience of having to come back from some awful debilitating disease and get back into it. Maybe I would struggle with that. Maybe I've had it very easy but I think that more people could be much more active than they are.

Interviewer: Uh-huh. I think those little, as you say…

Respondent: It's a lot easier to be active in the country. I think it's actually easier because of, well there's lots of reasons, but it's easier. And it doesn't have to involve costly gym membership and stuff. When you live in the country you can go out for a walk, you don't have to pay any money to go for a walk.

Interviewer: Uh-huh. Well we're going to explore some of those reasons [laughs].

Respondent: [Laughs] that's a nice little segway onto the next section yeah.

Interviewer: And so like we've talked about where you've lived and your physical activity. Now we want to talk about the physical environment that you live in.

Respondent: Uh-huh.

Interviewer: Yep, for work and play. And when we talk about physical environment we're talking about all the physical things that surround you, like the trees as well as the footpaths, the roads…

Respondent: Right, yeah.

Interviewer: … parks, whatever. Now again when we've talked to people in urban areas these are some of the things that they've actually sort of said, that maybe important to their physical activity, having access to things in your physical surroundings. So can you tell me a bit about how easy or difficult it is to be physically active in your area?

Respondent: Where I live it's easy for me to be physically active because the things that I enjoy and the things that I can afford to do are the things that I can do. I can get out and go for a run or a walk. I don't anything for that, anything in particular, I just need a nice quiet road and fresh air and I've got that. And the horse riding I'm set up with my house and my land to keep my own horses and I've got places to exercise them so that's easy for me.

At work we have that hill nearby that we walk up and there's no difficulty with that, we just get out of the building and walk and walk up the hill and back. So there's no, I have no, there's nothing impairing my access to what I like to do for physical exercise.

Interviewer: Good. OK, we're going to talk about a few things; availability and accessibility is the first one, of the places to be active. So if I was to ask you about the places to be active in your area what sorts of things would you tell me about?

Respondent: In my living area the roads are obviously public access anyone can go on the roads, so there's my running and some of my walking if I want to.

Interviewer: Yep.

Respondent: The bush land that I ride in and sometimes bush walk in is owned by forestry which is like semi-privatised I guess, I don't know what you

call it, but forestry I take it as being accessible to everybody and there are not fences up to keep people out, there are gates to keep vehicles out but horse riders can go through. So that's easy, there's no, nothing…

Interviewer: And you also talked about the sporting areas and the gym and the…

Respondent: Yeah the gym, and the sports centre at [town] is open to me on hours and times that I like it to be open. It suits me so that's no problem either.

Interviewer: Yep, and you're not far from shops, you've said that.

Respondent: No.

Interviewer: Yep OK. Does having places to be active actually influence you to be active or not?

Respondent: I reckon. Although when we lived in [city] and [city], yeah I reckon it does because I wasn't as active in those places. In [city] I had to keep my horses at an agistment centre which I had to drive to and so I couldn't ride at the drop of a hat. And yeah I didn't run and walk as much in those places so I would say it probably would influence me yeah.

Interviewer: OK. Now destinations, we've talked about this too; if I was to ask you about places that you could walk, run or cycle to from your home what sort of things would you tell me? Now you've talked about a few things.

Respondent: I would tell you I spend a lot of time on forestry land in plantations, tracks around plantations, for the exercise that I do and as far as

running you're not really, when your running you're not really going for a destination you're just running along a track or a road and so there's no actual destination in mind other than to run for a certain amount of time and then go back.

Interviewer: Uh-huh.

Respondent: Yeah so, and as far as, I don't know where that sort of features in with the access to the gym, I mean the gym you stop there for a purpose and then leave there so how does that work in?

Interviewer: That's a destination, it's a recreation facility.

Respondent: Yes it is.

Interviewer: That works, yeah. And do you think having destinations influence whether you're active or not?

Respondent: No not really, no, if all I could do was run and ride on the quiet country roads and not necessarily get onto the plantations I would still do those things.

Interviewer: OK. Now I'm going to ask you some questions about neighbourhood design, the built or man-made features about the area.

Respondent: Uh-huh.

Interviewer: Now what sorts of things might you tell me about if we focus on the non-natural features?

Respondent: In my residential neighbourhood, so it's a very old area, the houses are all, there are no what you would call new as in the last ten year

houses up there. My house is 1940s, I would say most of the houses around there are that or older. So it's a rural area, there's no, there are just houses, it's an agricultural area.

Interviewer: Any footpaths?

Respondent: No there are no footpaths.

Interviewer: No lighting which is why you don't…

Respondent: No lighting no.

Interviewer: … go horseback riding at night?

Respondent: No, so no there's not any of that, it's just a very….

Interviewer: Do the roads connect at all?

Respondent: Well yes, yeah roads connect.

Interviewer: Yeah.

Respondent: I mean my road…

Interviewer: There's no through roads that go…?

Respondent: Well OK so up past my house if I go out my driveway and turn to the right I will eventually come to the end of my road and it ends opening into forestry land, it goes on for thousands of acres. If I turn to the left it goes down to where it joins onto a slightly bigger road which is a bitumen road which then leads down into [town].

Interviewer: So you're on a dirt road yourself?

Respondent: I'm on a dirt road yeah.

Interviewer: So sides, each side of the road where you can walk?

Respondent: The dirt road no just goes pretty much down into the drain. It's probably a metre back on most sections of it to the closest fence because it's all paddocks up there mostly but the fact is that there's maybe two or three cars a day.

Interviewer: That's not much traffic.

Respondent: No so it's never an issue no.

Interviewer: And you can go for, like for your run and horseback riding you can go for a fair way on the road and then you've got access to the forestry and bush land anyway?

Respondent: Yes, yes you can.

Interviewer: It goes through there.

Respondent: Yes.

Interviewer: Sounds ideal [laughs].

Respondent: Are you horsey, have you had…?

Interviewer: I have had horses in my life yeah, I've lived in the hills and had horses.

Respondent: Yeah it's great.

Interviewer: Always the wonderful thing about being able to ride on a nice quiet track or take them into forest land.

Respondent: It is yeah. Hooray for forestry I say [laughs].

Interviewer: Yes [laughs] I used to live opposite one of the pine forests as well and that was the way.

Respondent: A palm forest?

Interviewer: No a pine forest.

Respondent: Oh right yes.

Interviewer: A planted forest yes, and they're great, they go forever. The only trouble is when you have, one of the horses didn't like kangaroos which sort of bouncey hoppies.

Respondent: Oh OK yes.

Interviewer: That's always fun [laughs].

Respondent: Yes that can startle yeah. Mine aren't really keen on alpacas but there are no alpaca owners down there and mine don't like pigs either but again there are no pig owners so it's good.

Interviewer: Yeah [laughs] that's good.

Respondent: [Laughs].

Interviewer: Happy horses.

Respondent: Yes exactly.

Interviewer: OK, so first question I need to ask you is do these built features…?

Respondent: Or in my case no built features.

Interviewer: No.

Respondent: Yes the lack of built features.

Interviewer: Yes, so do they influence whether your physically active or not? No.

Respondent: No because we don't have any.

Interviewer: And if you wanted to be more active are there any features that would help?

Respondent: No.

Interviewer: No, I'd say you're happy with them being as quiet as possible. [laughs]. And I think that's the trade-off you know in some rural areas there are still those quiet spots which are lovely…

Respondent: Yes, yes they are.

Interviewer: … and then there are other areas which…

Respondent: Which someone it going to put in a big subdivision yeah.

Interviewer: Yeah or getting closer to town and noticing some traffic build up and then…

Respondent: But still not much.

Interviewer: No.

Respondent: [town]'s a tiny town, I mean no-one could say it's an urban metropolis really.

Interviewer: No. OK so the other side of it is the aesthetics, you know the attractive, beautiful, natural things in the environment. If I was to ask you about those natural features, the attractiveness of your area, what sorts of things would you tell me about?

Respondent: Well I'd say for me it's a very attractive area to live in and I look out my windows and I just see hills, either forested or pastured, or I look out another window and I see an apple and cherry orchard which the leaves are turning yellow at the moment on the cherries so that's glorious of course.

Interviewer: Gorgeous yeah.

Respondent: And I have no buildings in my windows to look at that restrict my view, you know when you live in rural areas your eyes can actually look a long a long distance instead of just always focusing on short distances. You can gaze whereas you can't gaze when you're in the city.

Interviewer: Uh-huh.

Respondent: Yeah so I'd say it's beautiful, I'd say there'd be nothing that I'd need to, that I'd want changed there that would make any difference.

Interviewer: Uh-huh, lovely. And the other side of that is that you know if there's the not no nice bits which are the rubbish, litter, graffiti. Anything around your area?

Respondent: Nothing like that no.

Interviewer: And OK, do you think the aesthetics of your area influence you in being physically active?

Respondent: No not really, no, I couldn't say that…

Interviewer: They pull you out of the house and go…?

Respondent: No, because I'm an outside person anyway, I'd rather be outside than inside anyway, you know maybe at a subconscious level I'm even more satisfied with everything because it is a beautiful area but it's probably not something that…

Interviewer: It's been so much a part of your life hasn't it?

Respondent: … it's not something I spend a lot of time thinking about because I'm used to it anyway.

Interviewer: Yep, fair enough. And if you wanted to be more active are there features of your area that might help?

Respondent: Aesthetically-wise?

Interviewer: Aesthetically, more aesthetically pleasing? It's already beautiful as it is.

Respondent: No, it's already lovely so no [laughs].

Interviewer: And the last one to ask you about is safety.

Respondent: Uh-huh.

Interviewer: Safety in your neighbourhood area, and that could be your personal safety or road safety. Are there any sorts of things that you can tell me about about safety? You've already mentioned darkness.

Respondent: Well personally I feel very safe because the people are either known to me or I'm known to them so I feel very safe in that way. We don't have the street lights but I still feel very safe when I go out for a run either before or after work when the days are short and it's in the dark.

Interviewer: Yeah so that's different in terms of, you were saying about not riding horses in the dark but that's not a safety feature, its more you can't see?

Respondent: It's just the aggravation of it and really, yeah I think, I mean lots of people do ride in the dark, people who train for events and things who also work have to ride, have to train in the dark.

Interviewer: Yeah I used to have a riding instructor who did that but she used to have all these floodlights.

Respondent: Floodlights yeah, there's a riding school down the road on the way to [town] from me, I don't think they've got it floodlit, but yes when I lived in [city] I'd do my lessons after work under the floodlit arena. No so there's not many aspects that I would feel that would need to be improved there.

Interviewer: Great, OK. The last question before we finish up is there anything else that you'd like to say that hasn't been said?

Respondent: No probably not.

Interviewer: About either where you live or your environment or your physical activity.

Respondent: No, probably just well already my little theme is that I think that where people live in rural areas that it is an ideal place to be active you know.

Interviewer: Yes.

Respondent: There's so many things that you can do and simple things and you don't have any sort of inhibiting factors.

Interviewer: Yep.

Respondent: Yeah.

Interviewer: And I think what you've done really is talk a lot about what the good features are.

Respondent: Yeah.

Interviewer: Which is really, really helpful actually, it's really good.

Respondent: Well I imagine that you would get some people that would talk about the negative features of living in a rural area and how it could impact on them not getting enough exercise but I really think that that's, you know that's not accurate.

Interviewer: Uh-huh.

Respondent: Anyway [laughs].

Interviewer: So that's very helpful, I mean it's really helpful to know what does help.

Respondent: Yes.

Interviewer: As well as…

Respondent: Yes, I think it all comes down to the person and if the person wants to exercise the person will exercise wherever they are, I think, unless you were living in you know, I don't know, war torn Iraq or whatever and you couldn't go and do these things. But generally, apart from the odd [x] here, generally we have a very safe place and I think if people want to they will do it and other people will find excuses not to.

Interviewer: Uh-huh.

Respondent: Anyway [laughs].

Interviewer: And you were saying the nice thing about community is that people do know each other, not necessarily…

Respondent: Yes, yes, look there's always room for the people that want to do the ra, ra, hands on stuff and that's fine, I have no problem with that. And in [town] people can involve themselves in that if they want to.

Interviewer: Uh-huh.

Respondent: There's no lack of community activities down there and they're all very well publicised so I don't think that really anything more could be done down there to promote that sort of stuff. But anyway, we'll see.

Interviewer: Thank you.

Respondent: That's alright.

Interviewer: Thank you very much.

Respondent: Thank you for letting me be involved.

Interviewer: Thank you for your time.

Date: 3 October 2011
Duration: 58 minutes, 31 seconds
Interviewer: [interviewer]
Transcriber: [transcriber]

OK. Well I thought we might start by talking a bit about where you're living. And um, if you could tell me a bit about what it's like to live where you do, your likes and your dislikes. Now are you in [town] itself?

Yes. I'm central [town].

You're central [town].

Um, yes we've lived in this residence um for 22 years now. Um, came from further west, um [city]/[town]. Ah, but really when we were looking for a place nearly a quarter of a century ago we were just about to have our first child. So we were sort of looking to park spaces, parklands and um outdoor living and a place that we could play with the kids really, growing up.

So you did really look at that when you were..

Certainly we did. Certainly we did. And I just believe in the um, you know, [town] has just got it all really for people who enjoy parklands and beaches and rivers and mountains and that sort of thing. So we quite happily settled, bought a house and

settled here. And have just loved the area because of what of offers for outdoor living really.

Yeah. And that's really, when we are talking to people, that's really what's coming out in [town], just the range in choice that you have.

Yes, it's extraordinary. And the little town is still little. We really like that too.

Yes. And I was taking a walk along the beach last night and realising that you know, you feel like you're not in the middle of a town at all. It's really, the dunes are protected and back from the dunes, so you've got no sense there's houses there. It's like you could be walking on the beach …

And you've got it to yourself most of the time.

Yes, well I did.

Mind you, most [state] beaches are like that.

Yes, pretty much. So do you live um, just to check the property type, do you live on a house and block, or have you got a couple of acres?

No, we have a house on a large block, ah within 15 minutes or so walk to the centre of town. So that's the other thing about [town] that's really quite peculiar is um in the planning way back they allowed these really large blocks to be allocated to residences and we were, our house is an old one on a really large block. And we have the benefit too of a wonderful big back yard. Don't know how sustainable that is going forward. So, it suited us.

So you are about 15 minutes walk from the centre of your town. So that's what about 1 kilometre is it?

Yep. Yep.

OK. So in research we were saying that most of it has been done in urban areas, people often refer to the word "neighbourhood" and we're interested in understanding what that means for men who are living in rural areas and indeed if it's relevant. So what does the word "neighbourhood" mean to you?

Um, well neighbours that you know, you have some sort of ongoing relationship with I guess. And again we're really lucky where we settled. We had an older couple on both sides. Um, two out of three of them have now died. But um they played a really important part in raising our children, helping to raise them. I mean they established a relationship with our kids, like grandparents really. You know, across

the road we have long term friends which really we made when we moved in and have stayed close since. So there's that. But also I guess neighbourhood is proximity to services that you regularly use. And you establish relationships with the people who run those services. I know the little store at the bottom of the hill, the paper shop and the place where you get milk. For a long time the doctor was just at the bottom of the hill and that was great for the kids, when you know you needed to get to a doctor quickly. Um, so I suppose for me neighbourhood has inherent in it the people, so in relationships and um those lovely relationships that are long term and enduring really.

Mm. And if you needed to put a distance on that, um what sort of distance would you be looking at in your neighbourhood?

Oh probably a 500 metre radius.

OK. What about the word "community"? What does that mean to you?

OK. Well community takes it all in, pretty much. So community, well here it extends out for maybe a couple of ks radius. And it's everything from the Council chambers where you, you know, you drop in and pay your rates and pay for your dog's registration and you know the person on the front desk. And the hardware store, and you know the people in the hardware store and who you almost have a relationship with those guys, through to you know, the local sailing club that I spend a lot of time at, and everything in between. The little soccer club that my children, or at least my son was involved in. So it takes it all in. It's the fabric. It maybe makes up the total, the whole I guess.

And if you needed to put a distance around that it sounds like it's slightly bigger. It would be more, what the whole of [town]?

Yeah. Probably it would be. Because it's not that big. You can walk the main street and just be saying hello to people pretty much all the time really. I think [town] is..

So what distance would that be?

From our house?

Yeah, when we're talking about community what sort of radius?

Yeah I reckon it would be in the vicinity of 1.5 or about 2ks radius.

Yeah, everything is really quite well organised around here isn't it?

Yeah it is.

And not very far away. OK. Now I want to talk a bit about physical activity, um and just I was saying before it's a very broad term and it means different things to different people at different times of their life? So what does the term physical activity mean for you?

Um, it means doing, being involved in activity over and above that which would be done during work time, that's for sure for me. I started off in a very physical job, um daily physical activity. But ah, through the years I, particularly in the last five or six years, my job is becoming increasingly desk bound. And um, so I'm sort of much more aware that I need to be out and about and getting that extra stuff in that 20 years ago I probably didn't even need because my job was so physical. So physical activity is on the weekends jumping on the bike early and getting a ride in on Saturday morning, it's doing a swim every Sunday morning, and just a minimum of walking. I try and get a little bit of walking in during the week, but with work it's not that easy. Um, yeah so I've led a very physical life and I think that laid the foundations for I'm still very healthy still, apart from a few creaky bones. But heart and lung wise I'm very healthy. So I put that down to a lot of foundation work a little bit early in my life.

That's a really important point, yeah, that you do that throughout your life. Yeah. OK. And we'll go back and talk a bit more about the activities you do. Um, do you consider yourself to be physically active?

Well, for my age, moderately so. I'm not a fanatic. And I often look at particularly, retirees and how they have a spurt of physicality about their lives. It's, you know they hit the road or they hit the sea kayaks or the bikes or whatever. So I'm certainly not there yet because I just don't have the time but I think the fact that I have a range of physical activity that I'm involved in, a bit of swimming, a bit of riding. When the sailing season gets going I sail most Saturday afternoons. Race in a small dingy which is pretty physical. So I think yes, moderately so.

Moderate. Yep. That's fine. And where does physical activity fit into your life? Is it of no interest for instance, or a priority or a high priority?

No, it's high interest and high priority. Absolutely. It always has been since I was a little fella.

That's another foundation isn't it? Always being involved in it.

Yes. My parents were very healthy physical people, so…

OK. So in the research we think about physical activity and we think about any activity that lasts for at least ten minutes. That's the evidence of health benefit. And causes the body to work harder, you might feel your heart rate go up, you might feel warmer, you might feel huffing and puffing a bit. So, I want to talk a bit about that sort of type of physical activity, and over the last two weeks. And I'm going to go through different parts of your life, not to segment them, but just to make sure that we've covered all the activity that you do. So in the last two weeks have you been active at all at work for at least ten minutes at a time?

No.

No, OK. Because it's mainly a desk bound job now. OK. And if you'd wanted to is there anything that would have helped you be more active at work?

Oh, I have, you know you are entitled to a lunch time and if I slip the sandshoes on and go for a walk, um that's probably my only option at this stage. But I just find myself eating at the computer and just getting work done at this stage, basically. Ah, getting around my team a little every now and again. But really it comes down to the end of the day a lack of desire to squeeze in that activity in the middle of the day. Yeah, I guess while I'm at work I work and then ..

Yes, and then outside work is when you do..that's a fair comment. And I think that's a really useful comment to make because I think it's how work is organised for a lot of different reasons now, yep, that we are there and we try and do as much as we can when we're there.

There is um, you know there is a bit of corporate health and well being stuff going on. There's an attempt where I work to get the employees out and a little bit active. I don't tend to get involved in those though. I tend to be someone that needs – if I go, I need to go hard at it. You know, elevated heart rate, get in and get your exercise fix and an hour later you are done and dusted almost. So no, I'm not one much for group stuff. Yeah, not organised.

Yeah, that's a fair comment too. Because I think another really valuable thing is that a lot of people aren't. You know often you hear the answer, I think people tend, well often this sort of area gets to be a bit simplistic, with the just go and do this at this

time. I call it a bit like Health Policemen. And the corporate stuff is the same. We'll all be happy chappies and not everybody wants to do it.

It's very individual isn't it?

Yes, it is. It is. But I think one of the other –

Especially for men, I think.

Yeah.

Women tend to appreciate the group stuff maybe a little bit more. For example, you know you see groups of two or three women leaving the building at lunch time with their sandshoes and they go for a walk together. But I could never do that.

OK. Now in the past two weeks have you walked or cycled for at least ten minutes at a time to get to or from places? That might be a bit different to getting on your bike and just going for a ride.

Actually, yeah I have. I have. I try as often as possible, because we are so close to town to jump on that bike and just trundle into town here to meet people.

[town] is great because it's flat. It's just wonderful that way.

Or, drop down to the shop and get the milk and that sort of thing. So yes I have.

OK. Well thinking about that. Um, now you were saying that you've ridden down to the shop. Have you done any walking to get to places?

Yes. I walk, on the weekends I usually go to the shop. Take the dog for a short walk down and pick the papers up and drop back again.

So thinking about those two activities, um the intensity, how would you describe that? Would it be steady, moderate or vigorous?

It would be steady.

And for how long would you have done those activities at a time?

Oh, it would be um, a 20 minute round trip.

And how often might you do them?

I might do those 1-2 times a week.

And when did you do it?

Um Saturday morning, Friday morning, um this last week because we had a long weekend up here. So it's usually in and around the weekends.

The weekends. Yep. OK. And um, OK, so where you did it was actually going into [town]. And why did you do it there? What attracted you to doing it?

Well I'm just conscious of every opportunity really, I need to be doing, particularly some more weight bearing stuff. Because of the swimming and the riding um is not so much. And I trained as a [job] so I'm really well aware of the benefits of heart lung exercise but also weight bearing exercise too for bone density and the loss of bone density if you don't. So I'm always aware of the need I guess to be out there doing it and looking after your body, the physical side of your body.

Yep. And were you alone or with others?

No, always alone. I don't even walk with my wife, who likes walking. Because I just like to do it individually. In fact I don't really like walking, it's not fast enough for me. When I exercise, for example when I ride or I swim, I go hard. And get in and do it. A really intense workout, and then it's over.

Right. And um, is this what you usually do? Or is it one off? When you were talking about walking and riding to the shops.

Yes, that's what I usually do.

And does this change depending on the season or time of year?

No, not really. I quite like getting out in all seasons.

And if you had wanted to is there anything that would have helped you cycle or walk to and from places?

No.

Because you do that, you are actually aware of doing that. OK. In the past two weeks have been active around the house or yard? So inside or outside home.

Yes.

Ah, for at least ten minutes. Can you tell me what you did?

Ah yes. Um, because we have a big block with a large garden, there's a lot working around there.

Especially at this time of year.

Yes. Absolutely. And um I have a little rental property as well that we are working on at the moment just to bring it up to speed. So there was a lot of working around gardening and a bit of maintenance work and that sort of thing, but usually it's always ongoing. There is always physical stuff to do around the home in that area.

OK. So talking about those two activities that you've named, what would be the intensity of them? Would they be steady, moderate or vigorous?

Um, oh steady.

Yep. And for how long in the last couple of weeks?

Um, in the last two weeks I would have got in about um 6-8 hours I suppose.

And how often?

Yes, ah I would always do 3-4 hours of steady work a weekend. It usually ramps up a little bit of course when it's fair weather and you can get out and do it.

And who would you do that with?

Oh I work a little bit with my wife, in the garden and things. Yep.

And is this what you usually do?

Yes.

And does it change with the season or time of year?

Ah, it tends to. Actually it tends to be more of a winter, spring, autumn thing because over the summer I'm always involved in the yacht club. Pretty much from morning till night, all day. So Sunday is really the day I have at home and we just scamper around doing the things that we need to do, just to maintain the place really. And see a little bit of each other. So um, the 4-6 hours or whatever, is in the down season.

OK. And um if you'd wanted to is there anything that would have helped you be more active at home?

No, I think I've pretty much got it right.

Yeah, it sounds like a pretty good balance. With the time you have. Now have you been physically active for at least ten minutes during your leisure time, or spare time? So this is all the other activities that you were talking about. The bike riding. And you were talking about sailing, so that's more in summer, so OK, were there any other activities that we haven't talked about?

Ah, I get a bush walk in reasonably regularly. It's suffered a little bit in the last 12 to 18 months. Um, but I have a long history of bushwalking, of kayaking, of skiing in the winter. And the whole mix without focussing too much on any in particular. The two consistent ones in my life in the last few years have been the swimming and the riding.

OK. So let's focus on those two.

Oh and the sailing. And the sailing.

So those three. Um, when you look at the intensity of those, would they be steady, moderate or vigorous?

Vigorous.

And the duration?

Yes, the swim session lasts for an hour. Probably do 1.7 or 1.8 ks. The rides usually last about an hour and can be between 20 and 25 ks. They're not terribly long. And the sailing is intense. Because it is racing, competitive racing and probably last a couple of hours.

So you do that off, um over there.

Just off here, yeah.

And the swimming, is there a centre in [town]?

Yes. We have a little indoor pool that I swim pretty much in the off season. But I tend to like to get down just in the open water over summer and it warms up a little bit and swim at the beach and body surf and generally have a bit of a frolic around.

Yeah, enjoy the summer. And OK, so thinking of those three, um the duration, how long would you do them for?

Yes, so about an hour really. Thinking through it. Well the sailing is a couple of hours, but the bike riding usually lasts an hour, though I'm trying to build that up just now because I've got a long ride, fundraising ride, coming up in November. But um, that would be an hour and hour and a half. And the swim, an hour as I said.

And how often would you have done those activities?

Weekly. OK.

And when? They are usually in the weekend, the bike riding and the sailing, the Saturday. Swimming?

Swimming is usually a Sunday morning thing. I tend to get a few rides in in the better weather just riding home from work. Or riding to and from work, to [town].

Oh alright. So how long does that take you?

That takes about an hour and ten.

Wow. Right.

With a, yeah depends on the conditions. If you do it pushing into a strong headwind, but when the wind is not too big a factor I can ..

Do you go the backway?

I trundle along the coast and yeah, stay on that coastal drive. But then you get to [town] where you have to join the main highway.

And yeah that's not as much fun.

Yes, fortunately there's quite a reasonable area outside the white line that's not too bad. Nothing like you guys enjoy in Hobart.

It's very blessed with cycle ways. The only thing is we've got all these – but then again outside [town] you've got all the undulating country. That's half the fun. Um, OK, so where did we get to.

Now we've talked about where and who with. You've got those activities. And the sailing too?

Um, I try and sail a bit with my son when I can get him organised. But I quite like the, just the thrill of being solo, single solo. It's all up to you then. I like that challenge.

Yeah, it's very impressive. And is that what you usually do. Or is it one off, those activities.

No, it's usual. It's very routine. And regular.

And does it change with the time of year or season? Well it does with the sailing.

Yes. But the other two are pretty much constant. And if you'd wanted to is there anything that would have helped you be more active in your leisure or spare time? It sounds like you've got a pretty good balance.

Yeah, not really. It's going to be sort of up to me. You know I can, no excuses really. No.

It sounds like you've got really good balance. I mean the only thing you would want for is less work time and more leisure, but wouldn't we all?

Oh I'm working on that too. I am 55.

After all. Exactly. Now the last one is on physical environment. So that's the physical environment that we live and work and play in. And when we talk about physical environment we mean any of the physical things that surround you. So that could be the roads, the trees, houses, shops, traffic, parks, any of those things. And some of these things are things that people have said living in urban areas may be important to their physical activity. So we are interested in finding out whether that is relevant for people who are living in rural areas as well. So first up, can you tell me a bit about how easy or difficult it is to be physically active in your area.

Um, it's very easy.

Yeah. And why? You've got to ..

You've got the spaces.

Yeah. You've got all the natural spaces, the beach, the parks as well as the facilities.

And the proximity, really, I mean you can –

Yeah, they are very close aren't they?

Within the space of a couple of minutes from my house I can be on a tennis court, on a football ground, on a beach, paddling a river, walking a parkland, um you know just cycling on a little cycleway. So, um swimming in a pool. Within a couple of minutes. So yes it's really easy. So when we talk about availability and accessibility of places to be active you have just told me about what sorts of things um are actually quite close to you.

Are there any other things that ah, there may be things that you don't personally use, but what other places may be available, locally.

Um, well there are a good range of indoor recreation facilities. Gyms and stadium, here. And good quality facilities too. Um, you know for those that are into motor bikes and things they have access to a centre in the [x] Range which is only 25 minutes away. For the bushwalkers, again the [x] Range is only 25 minutes away. And you can be into some lovely walking. Up to [30: 33] for some walking. I mean [x] Mountain is only an hour and half away. So for the walkers there. But um, there is some really good mountain biking infrastructure within 20 minutes of our house. Um nothing to rival [x] Park in [city] mind you, but we think it's pretty good. Yes and of course in the back country they're opening up beaut mountain bike trails and things. So you know, the proximity is really very unique.

And access, are places convenient? Are they easy to access? Like for instance opening hours and cost as well as convenient?

I think so. Yeah. Um, clubs, you know there is a proliferation. There are lots of clubs and associations up here that make it easy for you and your family to be involved. And I don't know whether you are sort of aware of sport and the strength of particularly under age sport up here. And it's usually strong. Basketball and soccer and football in particular. Softball too. Yeah, surf lifesaving club is being dominant club over the last twenty years. So there is a really strong junior sport culture here.

That's really important. Especially with what we were saying before is that importance of that early fundamental basics that you have in terms of being interested in being active as well as doing it. Yeah.

Yeah. OK. So you know somewhere through the twenties and thirties, men's health in particular just goes totally off the rails. I'd say if you walk the streets here, there are different sights to walking the streets in [city] certainly because, oh it's just immediately obvious to anyone that has got a bit of an eye for it. People are larger and unhealthier up here.

So that's interesting. Particularly as you are saying you know that sport, OK, it's not the only activity that young people do, but sport is a good indicator of it. And it's strongly supported.

Yeah. And all the fitness and health that goes with that earlier on really for some reason is lost in the late twenties and early thirties. And you have high incidences of coronary heart diseases and all those other chronic health issues.

Yes, that's what we're seeing unfortunately. That's why it's interesting to do this research, is actually to hear from people themselves about not only what they do in their own lives, but also what they've noticed like you were saying in your own community. And you know whilst it's good to talk to people who um may see themselves as physically active, it's really important to talk to people with a range of activity in their lives. Because it's useful to know what's going on, and what the reasons for going on. But yeah, there's some of the stuff that we need to unravel more. But also that stuff of you know well as you say, what does happen, because you know there is some people who keep a level of activity through their lives. Maybe in their twenties and thirties they don't think about it so much, or they don't think about their health so much. You know, that sort of comes back later. That's interesting. Yeah. OK. Now I'm going to talk about destinations. If I were to ask you about places you could walk or cycle to from your home, what sorts of things would you tell me about? And you've actually told me about quite a few. But certainly shops and recreation facilities. And you've talked about friends.

Yes. Because I don't enjoy walking, but I am aware that I need to walk for the health benefit, my walking is just short distance. Rather than walk for an hour or an hour and a half and enjoying that. But certainly cycling I try and cycle to the destinations

further afield, just to, for everyday things, just to get things done. But yeah, everything is …

You have a consciousness that you actually try and think about what can I do today.

Absolutely. So does having destinations influence whether you are active or not?

Um, I think so. Probably, yes for community it certainly does. For example, they've just opened up a cycle way in the last 12 months between here and [town].

Very popular.

Very popular, in fact just amazingly popular. Because um, when I go for a ride, I'd never use that cycleway. I have ridden it back and forwards just out of curiosity, but I'd never ride that for exercise, because you can't go fast enough on it and you're dodging people. But it's just really heartening to see family groups and just people just trundling along there. I was a little bit wary of it, when I first heard it proposed thinking "oh who would do that?" But goodness it's been a success. And good on them. And um, you know, so I'd like, gee I can't wait for the day when they start extending it west from here and continue on so you've got that dedicated cycleway. Because it's obvious if they are there, people will use them. The roads, even though there is quite a bit of space outside the white line, there is certainly no place for families to be. It's just too dangerous. We've had a few riding deaths over the years up here, of cyclists being knocked over, yeah. So yeah, no I wouldn't. But it's such a good thing for families to do. And most families raising kids get them out on bikes and enjoy going for a ride with their little ones.

Yes, that's good to see. So any more opportunities around that would be very welcome.

Absolutely. Yeah, no it's a good investment. Great investment.

And certainly when I've talked to people before, as I was saying the first part of the study is coming up and talking to women who were living rurally. They all talked about the cycle track. And even if they didn't live in [town], and if they lived in the outlying areas, they would pack up the family of kids and the family would come down with their bikes. And the other one that came up was that extension of the coastal track up west. Came up regularly.

The other facility that we really need up here in terms of destinations to get people out would be a year round swimming facility. We haven't got one really on the [region] that is even half comparable to [city] and [city].

So the little facility that you use, it's quite small.

Ah, well you need to be sort of part of a club. And the club books it out for an hour. And you know I swim with the Aussie masters. And um but you know, it would be nice to have a public facility that on a Sunday afternoon when it's horrible outside say, "come on let's go to the pool" and you know, enjoy some swimming. It'll happen eventually but probably with the way the State is going, not in the next couple of years. Yeah that would be another fantastic destination that would make a big difference up here.

And you know like that is around that whole thing of if you wanted to be more active you know would it help to have more destinations?

Absolutely, yeah.

OK. I want to talk a bit about neighbourhood function and design. The built environment. So if I were to ask you about the built features of your area, what sorts of things would you tell me about? And that is talking about the stuff around roads and footpaths and um, and how walkable the streets are and things like that.

I think for the size of the population up here, the built facilities are, is quite generous really. Um, and well maintained. Um, you know if you talk about stadiums and developed park lands and play equipment and little cycle paths here and there. You know, I think it's wonderful really.

So there is a bit more of a choice of, you don't have to walk or cycle on the roads or the footpaths. There are sort of walkways and cycle paths.

It's getting to them I think. So you have to throw the bikes in the car or on a trailer. Transport them to and then have your little play there or your cycle around. Oh I guess that's OK.

And what about the sort of cycling around [town]? Because you obviously do that a lot. But you did make a comment about safety for younger kids. And what about [town] itself? Because I know people have mentioned outside that coast road is really quite narrow. So it's really for people who know their cycling.

Confident.

Yeah, confident. So there's that comment. But what about in [town] itself?

Well it's getting there. The new bridge I think has got quite a nice section there for pedestrians and cyclists. So I think you can make your way over. But it's um, here and there it's still problematic just dodging traffic and crossing roads. And that sort of thing. So I think the Council are by and large doing a pretty good job. They have a plan at least. And the plan is coming together. It's just a bit bits and pieceish at the moment. So at the moment it's a matter of having to dodge bits and pieces to get to a destination, but the destinations themselves are pretty much high quality, I'd say.

And do you see that the built features of your area influence whether you are physically active or not?

Oh definitely. Yeah, having those built features available to you. Allows you, and the variety I think. You just come back to that comment from earlier. It is just the variety of things that you can do up here. Um, built and natural. Means that by and large you know there is this outdoor culture. And outdoor doing things, particularly when you are young.

And if you wanted to be more active are there any built features in your area that would help. We've talked about the swimming..

I think the swimming pool. Yeah, for me the swimming pool would make a bit of a difference. Um, apart from that..

And you've talked about the track going west, the cycle path.

For families, yeah it would make a big difference. I really don't mind riding the road. It is a little bit narrow, but you know someone that has a bit of experience or got a bit of confidence about them, it's really good. And I think that the traffic that uses that road is aware that there are always cyclists on it anyway. So most motorists are respectful of and aware of..

And one suggestion someone made, and I think that is a sort of balance between local and accessible is whether they drop the speed limit to just a little bit more. Whether it needs to be 80 or, I think it's 80 isn't it? Whether it needs 70 or something. Because it's really tight narrow bends.

Yeah, you've driven it obviously.

Yes, yes.

It's a beautiful little drive.

It’s gorgeous.

Yeah, it’s beautiful.

Because I spoke with some people that who, apart from well that is the study area so I wanted to go out and have a look, but I spoke with some people who actually live along that area. Amazingly they have got a place that’s um coast side, rather than close to the coastal reserves. They had a lot to say about the bike track and living close to the coast.

Gee they’re in privileged positions aren’t they?

Yeah, yeah. They just lucked in you know, years ago. Well not that many years ago.

Got to put up with that railway track being right through their backyards virtually.

Yes. That’s the only trick isn’t it? And that’s one thing you can’t do is walk along the railway track. It doesn’t work very well if there’s trains on it.

They need to rip that railway track up and use it as a cycle track.

Well exactly. That was one of the suggestions.

Oh I bet it was. That would make a difference to the value of their property.

Exactly. OK. Now if you wanted to be more active are there um – sorry, where are we? Um, are there any, yeah we’ve just talked about the built features in your area that would help. Now talking about aesthetics which is the other side of it, if I was to ask you about the aesthetic qualities or the attractiveness of your area, what sorts of things would you tell me about?

Um, I think [town] is a fine example of just beautiful settings really.

You’ve got it all haven’t you?

Yeah.

Coast, hills, parks, trees.

It’s just a beautiful, beautiful place. And the river you know just all brings it together. So it’s outstanding. And I have travelled a lot, a lot, all over the world. And I just, it’s just a treat coming back. And I also I really enjoy selling this place, based on how beautiful it is. You know, the natural beauty of the place. Um, yeah.

It is. It’s quite a gem really. Not only for it’s natural beauty, but for it’s, it’s got a big enough size, but it’s not too big. So it’s very well situated.

I sort of sell [city] for along those lines as well. Like it’s a beautiful size city I think. It’s got all that you want in a city, but it’s not too big.

Nah. Well I live in West [city]. We actually came from [state], so we always came down to [state]. And always [city] and one other place and we said "why aren't we living here in the place we really love?" So we did, so we just were able to live in a beautiful environment around the corner from [reserve] and the rivulet, but just walk down into [city]. And it's just stunning, wherever you look there is a mountain or it's the river. And it's like "yeah". Some of the world's best kept secrets.

So you've got a view down towards the [x] casino?

Yeah. That's right. And we've got a fair few trees, um [x] around house. So it's nice. I don't mind having a view screened of the water, that's fine. OK, so do you think your local environment is aesthetically pleasing or attractive near your home?

I do.

Indeed. And do the aesthetics of your area influence whether you are physically active or not?

Ah, that's a bit of a combination isn't it? I mean it's nice to be out in a lovely setting, but at the end of the day I think you've got to have a health consciousness really, to be there mentally, and to value it.

Mm. Though it certainly helps having a beautiful environment. But you would still have, you would still be aware of needing to be active wherever you were? You'd find some things that would work for you. They may not be as beautiful or pleasurable.

No.

OK.

At the end of the day you've got to value the importance of physical activity and healthy lifestyle really. Otherwise you are not going to get out in any environment, no matter how beautiful it is.

No, that's true. That's true.

And especially that 15 or 20 minutes of elevated heart rate stuff, um you've got to value the importance of that and get out and do it.

And if you wanted to be more active are there features of your area that might help if they were more aesthetically pleasing? No. Not here.

No.

One last area, safety. If I were to ask you about safety in your area what sorts of things would you tell me about?

Um, safety. Well in terms of biking and um, well let's take biking. I think there are still a few safety issues around because there are not those connecting cycleways yet. But I've taken some interest in the master plan and they'll be working on that.

It's starting to look like it could come together to address those issues?

Yep. Yep. Um, for pedestrian traffic and general you know joggers and those out and about. I think it's as good as any. Um, you know the beaches and the outside swimming areas are fantastically safe. Compared to like a [beach] or somewhere like that. You know they can be quite treacherous. Our swimming areas, our beaches are wonderful. Um, yes so I think no issues there.

So does personal safety influence whether you are physically active or not?

No, not really.

No, it's a sort of safe community.

I can imagine, because I've always been outdoors and physically active in the outdoors, you know I've got an awareness and a confidence I guess. But I can imagine safety being a high on um, you know a high, it would concern maybe a lot of people. Just the getting about. I mean there's not a safety to person, like security safety issue around here. Again it's a very safe neighbourhood, ah from bashings and getting into trouble that way. Yeah, no overall I think it's very good.

And does road safety influence whether you are physically active or not?

No.

No. Again you're aware of ..

Yeah, I think it's a matter of traffic being respectful of pedestrians and cyclists, pretty much. Every now and again you have a bit of a run in with car or a truck that wants to just give you a hard time. But ah, they are very few and far between.

Yep. And if you wanted to be more active are there personal or road safety issues that might, if addressed, might help you to be more active. Now you've already talked about the Council are putting together a master plan. Is that for cycling or traffic, or ..

Um it's really connecting these recreational areas that extend East to [town]. And follow the um, through the cycle way through the parklands here. Ah, connect

across the bridge and then extend west on that side somehow. And it's a pedestrian cycleway combination, with parklands sort of dotted along.

Sounds excellent.

Yeah, yeah it is. It's really impressive. Got a way to go, but..

As with these plans, they always seem to be 5-10 year plans, or even 20. At least they've got a plan.

At least it looks like it's coordinated and thought through and yeah..rather than ad hoc and just whimsical.

So that's it. But before we finish today is there anything that we haven't covered about where you live, your physical activity or the physical environment that you'd like to raise?

Um, not really. I mean it just concerns me that there is such a chronic health issue. And it gets worse. I mean [city] is worse than here.

Is it?

Yeah it is. You can tell if you just walk the main street in each, it's really, it's obvious to anyone that has got a bit of an eye for it. And it's linked to the socioeconomics of the area, and how struggling really on the [region], with [city] in particular. A lot of the health issues.

Yeah, I must admit, my health background, I come from a broader public health background which looks at what you'd call social determinants of health, all the other things that affect your health. Your education and what income you have. Environment is one of them. Still there are all the other things that affect it. Yep. Access and equity. But yeah, it's interesting. And then it's interesting to have a look at a community like this that um, of course there would be, you know, a range of people who were not, you know, in terms of who is doing better than others. But there is still a lot of things happening in this community.

Yeah. Access and equity is an interesting one isn't it? I had a state-wide role for a couple of years just recently. And so my team was around the state and I would go down to [city] and they'd say "oh can you get us some more money down here? Our facilities are shocking" and I used to just love saying "well you know maybe you should trot up and visit, I'll show you some facilities that really need money spending on them". But invariably what they'd do is just dismiss it. You know, so "Oh well,

stiff titties", basically. You know, "you live up there in the sticks, that's what you have to live with". And we get that all the time up here. And I sort of think "well, OK you know most of the taxpayers are down there and it's you know where the money is and that sort of thing, but our state politicians have just got to keep really fighting for some sort of equity and you know, just an equitable distribution of money into physical structures and things that make it easy for people to get out and enjoy living. And you know, your indoor swimming pool up here I mean I think it's just um, so well over time that we have that.

It's certainly a big enough centre.

It would make such a big difference to families up here, particularly through the winter months. So yes, I quite enjoy that debate.

Well you have to keep having it don't you?

No you do. That's exactly right. You just need to keep ah,

Keep on keeping on.

It's the squeaky wheel that gets the oil.

Yeah, and it sort of sinks in eventually. But look thank you very very much.

Thank you.

AUDIO RECORDING ENDS
