# Supplementary material for: A Qualitative Study of Environmental Factors Important for Physical Activity in Rural Adults
Source: PLoS One. 2015 Nov 10;10(11):e0140659. doi: 10.1371/journal.pone.0140659 (PMC4640845; doi:10.1371/journal.pone.0140659)
Supplement: S4 File — (PDF) [file pone.0140659.s004.pdf]

Interviewer: So I thought me might start by talking a bit about the place where you live and for example, what it's like to live there, so if you can tell me about what it's like and maybe your likes and dislikes of where you're living currently.

Respondent: It's just beautiful [town] is.

Interviewer: Are you in the township itself?

Respondent: In the township, across the river there near the beach really and so it's a really flat town mostly, so walking, you walk on a flat and it's just… mind you, it's such a small town, I don't have to walk very far to get to the supermarket or to go for a walk, there's tracks everywhere. They're easily accessible and it's just a lovely little place to live.

Interviewer: So it's had some thinking about people being able to walk to places, not just along the beach?

Respondent: I don't think that's how it really started out. In the beginning I don't think there was a lot of thinking, I just think over where I live it's just so beautiful and there's a lot of parkland, that was a golf course once, so when the town first began it was a golf course and then the town built up gradually around it and so it just became a natural sort of area where there wasn't any buildings and then I guess the council acquired it and now it's just a park area and walking track. The most recent walking track out to [x] Beach, that's only really new. That's only in the last two years it's been put in. It's direct to the [fruit] farm.

Interviewer: How far is that?

Respondent: Quite a while. I haven't walked there. It's quite a trot out there, but lots of people do it. There's always someone walking.

Interviewer: That's good. So, just a bit about the property type where you live. There's a house and a house block?

Respondent: A house on house block?

Interviewer: Yep.

Respondent: Yep.

Interviewer: And just the length of time you've been there?

Respondent: Ten years.

Interviewer: So well settled in.

Respondent: Yeah.

Interviewer: And your reasons for living here?

Respondent: We moved here from [city] and my husband got a job in [city] and I got a job at [town], so [town] was in the middle and so it's the same distance to each town.

Interviewer: Fair enough.

Respondent: It's a bit like the [x] Coast really here, it's just so lovely.

Interviewer: So you're right in the, yeah, it's gorgeous. You're right in the township itself?

Respondent: Yes.

Interviewer: You're not far from the town centre itself?

Respondent: Right near the [location] and it's all very accessible.

Interviewer: OK, I'm going to ask you a bit about… I was saying before with a lot of this research being done in urban areas and cities and people often refer to their local neighbourhood and I was interested in understanding what the term might mean with women who are living in more rural areas. So, what's the word neighbourhood mean to you? Does it have a meaning here?

Respondent: Yes. When I think of the neighbourhood, I think about whether it's safe or not.

Interviewer: OK, safe as in what sort of sense?

Respondent: Safe that you can go out and you're personally safe and whether the children can walk up to the shop or whether they're allowed to play in the park without me or walk to basketball.

Interviewer: And is there an area that you deem to… you were saying safety was important, so is there an area that you deem safe where you live?

Respondent: Well there's an area where they're not allowed to go without me and that is the furthest beach along. They're not allowed to go up there.

Interviewer: So that sort of distance is how far away?

Respondent: One and a half kilometres.

Interviewer: OK, so when you come to looking at what your neighbourhood is, does it have a distance?

Respondent: Yes it does. It's my walking track distance.

Interviewer: Oh OK, what's that?

Respondent: It's not very far. It's only one and a half kilometres around up one direction and then one and a half back, so three kilometre walk and that also sort of includes the main street, the shops, Coles, the library, the video store, it's all in that space.

Interviewer: That makes a lot of sense. What about in terms of walking distance? Are you saying it's one a half kilometres that would be, how long would it take you to do that?

Respondent: There and back?

Interviewer: Yeah.

Respondent: It's three kilometres all together and by the time you go and come back, oh, 20 minutes.

Interviewer: OK and what about the word community. Does that have any meaning for you?

Respondent: It had more of a meaning to me when my children were younger, but now that they're older, I don't think about it so much anymore because I'm not talking them to the surf club and I'm not socialising there and now I work full time that they're older, no, I don't really think about it as community. It's just home really.

Interviewer: And what was it then?

Respondent: Back then when the kids were littler?

Interviewer: Yeah.

Respondent: Yeah, it was their sports and having to stay while they played and so you met a lot of people there and…

Interviewer: So it was the people and the connections with the school and the activities?

Respondent: Their fundraising activities and getting involved in that whereas now I don't have to stay because they're older and I can cook tea while they're there.

Interviewer: Would that have had a distance associated with it when they were little? You were saying to get to the sports…?

Respondent: No, we could have walked, it's not far.

Interviewer: So it's within a kilometre?

Respondent: Yep.

Interviewer: Sounds like everything's really quite close by for you. It's good. OK, I'm just going to talk a bit about physical activity now and what the term physical activity means for you I'd like to ask you?

Respondent: I don't do a lot of physical activity. My friend's thought that I could be in the control group for your study because I don't do anything much.

Interviewer: It's really to hear from everyone when it comes to physical activity and it's actually more important to hear from people who may not think themselves as active as others.

Respondent: I don't… especially when it's cold, I don't want to go out and it's windy and I don't want to go out and I really am pushed along by someone else rather than myself, I'd just stay home.

Interviewer: OK, so what would physical activity mean for you then?

Respondent: I walk the dog when I have to, which is that three kilometre thing and I walk up the street. Sometimes I'll make myself walk, but sometimes I'll go weeks without doing a walk.

Interviewer: Particularly what, in winter?

Respondent: Particularly if I'm studying and I've got an assignment due. Just the table and nothing else matters and when it's cold and freezing. Mind you, walking around [town] is really flat and I went for a walk with the students at work and we walked up a mountain, goodness, it was just like a stress test, having to walk up a hill rather than walking around here.

Interviewer: Walking flat's very different isn't it? I used to walk and live in a flat, sort of close to the coast area, originally from [state] and then I moved to the hills and the difference is noticeable.

Respondent: Gets the heart going.

Interviewer: Yes, OK, that's a bit what I want to talk to you about now and in our research we often think about physical activity as any activity that lasts for ten minutes or raises your heart rate a bit or you get a bit of a puff up or a bit warm or feel the heart rate going and I wanted to just ask you, well, it's a number of questions around physical activity in the last couple of weeks and there'll be just different parts of your life I wanted to explore. In the last couple of weeks, just have a think about it, have you been active at all at work for at least ten minutes of time with that definition in mind?

Respondent: Once.

Interviewer: OK, what were you doing then?

Respondent: We had a meeting in [town] and I was at the [x] Hospital and it's just once in a blue moon, it'll never happen again, we decided we'd walk to the meeting down town, but that rarely ever happens.

Interviewer: And that particular… so it's not usual and that's often the case, we're not very active at work, particularly the sort of jobs we have. Now with the activity that you told me about, would you call that moderate exercise? It's intensity whether it's steady or…

Respondent: No, it was just walking.

Interviewer: Just steady?

Respondent: Yep.

Interviewer: And duration?

Respondent: It probably took ten minutes.

Interviewer: OK and frequency, you said it doesn't happen very often?

Respondent: No, usually because we have to carry things where we're going and so you kill yourself if you carry baskets full of stuff.

Interviewer: Occupational Health and Safety. OK and does it change at all depending on the season or the time of the year?

Respondent: At work?

Interviewer: At work.

Respondent: No.

Interviewer: And if you wanted to, is anything that might have helped be more active at work? Someone to be active with?

Respondent: Well I've just participated in a new [activity group] walking thing, so that helped. I had to wear a pedometer, made me realise when I had good days and really bad days with no walking and the activity days. I get a heap more activity when I go to work at [town] than what I do when I work at [city].

Interviewer: And that's because…?

Respondent: That's because, I guess I only go there once a week so I'm racing around trying to see everybody in one day, whereas at [city] I see them for the other four days and I don't have to race around like a mad thing trying to catch everyone, whereas at [town] I have to condense it all down and race around and up and down stairs whereas at [city] we only have one flight of stairs. [town] is two stories and lots of stairs.

Interviewer: So the nature of the work and you being there and also, yeah, just the nature.

Respondent: And my office at [city] is glass and everyone can see me, so people will come to me, whereas at [town], no one usually knows where I am and I'm a lost soul because I don't have my own office, so I just cruise around.

Interviewer: Now, in the last two weeks, have you walked or cycled for at least ten minutes of time to go to and from places?

Respondent: Well, I've walked a couple of times ten minutes each way.

Interviewer: So was that moderate or steady intensity?

Respondent: Just steady old walking.

Interviewer: Yep and duration?

Respondent: Probably 20 minutes.

Interviewer: And when did you do it?

Respondent: After work.

Interviewer: Why is the obvious question? You had some time?

Respondent: Yep, 20 minutes to spare.

Interviewer: OK and where would it be?

Respondent: My little track, up the main street and down the beach and back home. Take the video back to the video store and keep going.

Interviewer: That's a good one, some reasons for doing it, yeah that works. Were you alone or with others?

Respondent: I usually walk with my husband and the dog. He goes for a ten kilometre run and he comes home and makes me go for a walk.

Interviewer: So you've put in a few reasons (laughs). Is this what you usually do or was it a one off?

Respondent: It just depends how busy I get. I'll go fairly frequently, but then if I get busy with study then I just don't go so then it will be maybe three or four weeks where I don't go at all and once that assignment's in, I'll go more frequently, three or four times a week, sometimes five.

Interviewer: And does it change depending on the season or time of year?

Respondent: Absolutely.

Interviewer: So, in winter it changes?

Respondent: It does. If I can get out of going I will. It's just too cold.

Interviewer: If you wanted to, is there anything that would have helped you use more active forms of transport, like walking or cycling from places?

Respondent: Well I guess if I worked in our town I would walk to work, but I don't so… and I won't park a long, long way away from where I work because I've got a whole lot of stuff I've got to take backwards and forwards from home and work. I've got a trolley, but…

Interviewer: But living and working locally in the same area would help. Now, in the past two weeks, have you been active around the house, inside or outside in the yard?

Respondent: No.

Interviewer: Is there any reason why that was so? Was that different or unusual for you?

Respondent: No, that's pretty usual.

Interviewer: OK and if you'd wanted to, is there anything that would have helped you be more active, if you'd wanted to?

Respondent: Not really, I don't think so. The garden doesn't need doing.

Interviewer: You're one of the self sufficient ones? OK, now have you been physically active for at least ten minutes during your leisure or spare time the last couple of weeks?

Respondent: Yes, had a fair bit of spare time the last couple of weeks. I just go for my little walks.

Interviewer: This is a good time in terms of between assignments and…?

Respondent: But I don't do anything special, I just go for a walk.

Interviewer: And take the dog and go to the shops. So that was fairly steady exercise and it's usually after work. If you'd wanted to, was there anything that would have helped you be more active in your spare time?

Respondent: Motivation. I don't have very good motivation really. I used to do heaps of sports when I was a kid, but I think I just got over it. I don't want to swim, I don't want to do anything really.

Interviewer: Prefer to relax when you have the opportunity and you've got a busy life too. It's not enough time is it to relax?

Respondent: No.

Interviewer: That's good. It's really interesting to hear and you've got your physical activity area that you can do and this what you want to do. Now, I'm going to swap topics a bit and talk about physical environment as the second part of it. A physical environment where you live and work and play and when we talk about physical environment, I'm talking about physical things that surround you like roads, trees, houses, shops, traffic lights etcetera and some of these things, people living in urban areas have said may be important for them, so we like to ask if it's relevant to you as well. Can you tell me first of all a bit about how easy or difficult it is to be physically active in your local neighbourhood area?

Respondent: It's easy to be physically active. The paths are good for if you've got prams or whatever. There's lots of tracks, there's lots of places to walk.

Interviewer: So there's walking tracks or cycling tracks?

Respondent: They're both.

Interviewer: OK and places to take the dog for a walk as well.

Respondent: And there's a dog beach and human beach.

Interviewer: Now, availability and accessibility of places to be active. If I were to ask you about places to be active in your neighbourhood area, what sort of things would you tell me about?

Respondent: I know there's places, I just don't go there. There's the gyms, there's quite a few gyms and they do tai chi in the park on the weekend.

Interviewer: You've got a park as well you mentioned. How far away is that?

Respondent: That's about ten seconds away.

Interviewer: Oh you live close to a park. How beautiful and the beach?

Respondent: Right on the mouth of the river.

Interviewer: Oh, that's pretty special.

Respondent: It is beautiful. Yeah, so they all get down there and do tai chi and barbeques and all sorts of things.

Interviewer: So it's a popular park?

Respondent: Yeah. It's a recreation ground, there's the basketball stadium, netball courts and there's quite a few football grounds and hockey grounds around.

Interviewer: And accessibility. Are these places convenient?

Respondent: They are, they're on prime real estate.

Interviewer: So they're easy to get to and is there a cost or opening hours for things?

Respondent: There's a cost to the basketball because that's inside, so kids have to pay three dollars to go in and turn the lights on and I guess there's a cost to go to the gyms, memberships and that, but the netball courts and football grounds, I think they're just open all the time, there's no cost to go there.

Interviewer: Does having places to be active influence whether you're active or not?

Respondent: Doesn't influence me. It might influence other people, but it doesn't make me want to get out there when it's cold and I know gyms are warm and have heaters in them, but I don't want to go there either. It's just motivation I suppose. I control my weight by what I eat, not by what I do.

Interviewer: But it sounds like you've also worked out what suits you too. After all, we're not all gym people. If you wanted to be more active, are there more things or places that would help you?

Respondent: I think if I lived in [state] I'd be more active.

Interviewer: Warmer?

Respondent: Yeah and I suppose when you're there and you're on holiday, you just have so much time, you're just laying around, you can go for walks and just being warmer I think is nicer.

Interviewer: The next is to look at destinations. If I was to ask you about places that you could walk or cycle from your home, what sorts of things would you tell me about?

Respondent: The strawberry farm on the new walking/cycling track.

Interviewer: And you've got a park obviously next door and the shops and the beach. Does having destinations influence whether you're active or not?

Respondent: No, it doesn't influence me.

Interviewer: If you wanted to be more active, would it help if there were more destinations within easy walking?

Respondent: No, I just lack motivation.

Interviewer: And that's why you walk as it is, yeah.

Respondent: I make myself walk. I don't wake up and think 'oh great, I'll go for a walk'. I just think 'oh God, I better go for a walk'.

Interviewer: Fair enough. Now, neighbourhood design. If I was to ask you about the built or non natural or man made features of your neighbourhood, what

sort of things would you tell me about? You mentioned footpaths and things like that. You also mentioned that things connect together, the streets connect?

Respondent: Yeah, there's a lot of work been done here recently with the new walking and riding track and now there's going to be a new development along the river, the wharf development and there's a new bridge that's just about to open and all that wharf development, that's going to be a new sort of community area, so they're changing roads and putting in more roundabouts and slowing the traffic down and improving the footpaths and accessibility a lot in the last two years.

Interviewer: OK. Do the built or non natural features of your neighbourhood influence whether you're active or not?

Respondent: I don't think so. Just that it's safe and I don't have to worry about being chased by dogs or people or anything.

Interviewer: So that's a big thing.

Respondent: If I had of been younger, like, when I was younger and had the kids in prams, it absolutely affected me. Where I used to live in [city] there were no paths and it was too rough and I couldn't go walking with the pram where we lived, but I don't have to worry about that now.

Interviewer: But that makes a difference doesn't it. It's very nice and not only don't you have to when your children are older, but also if they were of pram age, your footpaths aren't a problem.

Respondent: And there's quite a few disabled people in [town] who have those motorised cars, so they seem to get around pretty easily.

Interviewer: That's good. Now, if you wanted to be more active, are there non natural or built features in your neighbourhood that would help?

Respondent: Well, there's a whole lot of new gym equipment down in the park. Leg exercises and sit up things where you do sit ups. I think there's four things they put in like a gym for adults down there you could use that if you were motivated.

Interviewer: Now, I wanted to talk about the natural aesthetic qualities or the attractiveness of your neighbourhood, what sorts of things would you tell me about?

Respondent: I'd tell you about how flat it was and how good the walking track was and how you don't have to stay on the walking track, you can flit off between the trees and get onto the beach anywhere, so there's beach on one side, there's kids play areas on the other side…

Interviewer: And you've got coastal vegetation you were saying?

Respondent: Little sand dunes. They often have Learn to Surf down at the beach. It's a safe beach as far as the tides go.

Interviewer: So it's a popular one for being down there. And do you think your local environment is aesthetically pleasing?

Respondent: I do, especially down by the tracks, there are toilets they're clean and everything's always well looked after.

Interviewer: That's the other side of it isn't it about rubbish, litter and all that sort of stuff, graffiti, but it's well looked after.

Respondent: Lots of barbeque areas. Couldn't even count the amount of barbeques down there where people can go and there's always timber there all stacked up for people to use.

Interviewer: Really?

Respondent: They're not gas, they're timber. There's newspaper and timber at every barbeque waiting for people. It's like having a butler.

Interviewer: That's organised and if you wanted to be more active, are there features of your neighbourhood that might help in terms of being more aesthetically pleasing?

Respondent: If I wanted to be more active? Well, it's pretty nice. I don't think they could improve it very much.

Interviewer: It sounds pretty scenic. Good. Now the last area is around safety in your neighbourhood and if I were to ask you about that, what sorts of things would you tell me about?

Respondent: Well, there's not a lot of crime, so it's safe to go out, even at night time you can go walking. It's just a quiet little town without a lot of crime and there's no naughty dogs running around and if there are then we just call

the pound and they take it away. Lots of neighbourhood watch, so I suppose everyone is always… there's a lot of retired people here so they take it pretty seriously.

Interviewer: So that's personal safety. Does road safety influence whether you're active or not?

Respondent: I guess it does, whether you can get across the roads or not because you wouldn't be able to get off your block unless you could, but it is a small, quiet town and there are crossing islands on a main road that runs thought. There are crossing islands, so you can just cross to the middle and wait and then cross again when it's safe, so it's not like you have to race across the road and risk your neck, there's lots of places you can cross and I think we have two or three road traffic lights as well, so it's pretty good.

Interviewer: Sounds like it's pretty well organised, it supports you and your activity and if you wanted to be more active, are there any personal road safety issues that would need to be addressed that might help?

Respondent: I don't think so. It's pretty well organised.

Interviewer: OK, before we finish up today, is there anything else you'd like to talk about or tell me about where you live or your environment or physical activity that we haven't covered today?

Respondent: No, I think we pretty well covered it.

Interviewer: OK, that's all the questions today. Thank you very, very much.

Interviewer: So I thought I might start by talking a bit about the place where you live, and can you tell me what's it like to live around where you do, your likes and your dislikes?

Respondent: I was born and bred where I'm living now, so I suppose I have a bit of a clouded view in that it's my ideal place. And then but saying that I went away for 12 years overseas, so I experienced a different culture, I lived in a city, a large city, and then obviously that makes you then appreciate to where you are. But because I've come back to where I was born and bred, I think you've got that little bit of – not like homesickness – but sort of a... I don't know.

Interviewer: It's a special spot for it, isn't it?

Respondent: Yeah. And people say... and because we're splitting up the farm – I'm on a farm – because we're splitting up the farm you still feel as if you should retain some because of your parents.

Interviewer: Hmm, hmm. Hmm.

Respondent: Yeah. So I've got a little bit of that on that side, but then I have to sort of think well, do I really like living here as well? So getting back to your question, I do, yes. Yes, I think I do, and I like the quietness and just the space as well.

Interviewer: Uh-huh. Uh-huh. Now do you live in [town] – the immediate area?

Respondent: Yes.

Interviewer: Yes, you do. Yes.

Respondent: Yes.

Interviewer: What do you call it? Do you call it a village or a hamlet?

Respondent: Oh, I was going to say a hamlet as in that respect, because originally we use to have a shop, a Post Office, a petrol station, two Churches, and now we just have like a roadside delivery Post Office, that's all. That's the only amenity we had. Oh, and a school – we would have had a school, Church, Post Office. So all we've got left is just a roadside delivery Post Office. So it would have been, hmm.

Interviewer: So now you're living on acreage now, are you?

Respondent: Hmm, hmm. Yes.

Interviewer: And so how big is the area that you're on?

Respondent: We're splitting... we were on 40 acres, so we'll be downgrading to about 20 acres.

Interviewer: Uh-huh. Uh-huh. And so the length of time that you've lived there, you've actually lived there most of your life other than about 12 years, where you've actually...

Respondent: Yeah, that's right. Yeah, that's right.

Interviewer: Uh-huh. OK. And you've already said your reasons for living there were you grew up there, but was there something that pulled you back as well?

Respondent: Family pressure I think. I have to admit it was family pressure. Yes. And also the country we were living in was [country], and I didn't like the [religion] [Laughter].

Interviewer: [Laughter].

Respondent: And once our kids started school it was getting a little bit pressurised, so I just found it easier to come back to a society where you could speak your mind, and do your think... although now it's changed. I mean I'm talking 20 years ago, so now the Church doesn't rule the people like it did when we lived there.

Interviewer: No. No. No, there's been quite a change.

Respondent: Hmm.

Interviewer: Yeah, I've travelled around there, but that's another story.

Respondent: Yeah.

Interviewer: Yeah, yeah, yeah. I know what you mean. And so what's your distance to the nearest town?

Respondent: [town] .

Interviewer: That would be... you'd classify [town] ...

Respondent: Yeah, we would regard [town] . Yes. So that's 15k isn't it? Something like that?

Interviewer: Yeah. OK.

Respondent: Yeah, 15k I think. Yeah. Hmm.

Interviewer: Yeah. And how far are you from [town] ?

Respondent: About eight I think.

Interviewer: About eight?

Respondent: Yeah.

Interviewer: OK.

Respondent: We're sort of ten minutes to here, and 20 to [town] , that's roughly it, so a quite broad...

Interviewer: Yeah, yeah. So in terms of your closest centre, [town] would be a bit closer than [town].

Respondent: [town] would be closest, but the amenities like the shopping would be [town] .

Interviewer: Yeah. OK. So, thank you for that. So, I was saying that this has mainly being done in urban areas, this research, and people often refer to their

local neighbourhood – so I was interested in understanding what the term neighbourhood means for women who live in rural areas, if it's relevant at all. And so what does the word neighbourhood mean to you?

Respondent: Well because – this is a tricky one – we spend most of our weekends at a place called [town] .

Interviewer: Yes. Yes.

Respondent: And we have a holiday home there. And I regard that more as our neighbour, as our cultural centre, whereas where we live now that's just a place where we live and work. So, and I don't find that there's any connectedness. I mean I don't even know – like, I know our immediate neighbour where we live now, but I do not know any of the people who live in the area because they come and go, and there's no meeting place.

Interviewer: Hmm. And that's changed as you've said.

Respondent: Yeah. There's no... as I said, there's no actual pub, so you might go to [town] , or [town] , or to [town] , and that would be where you'd see your neighbour. You wouldn't see them at the Post Office or anywhere.

Interviewer: So does it have any particular relevance then?

Respondent: No. So when you say you live at [town] , people say, "Oh, I know someone there." And I say, "Well I don't know them."

Interviewer: Yeah. Yeah.

Respondent: You know, it's sort of... it's just a place that you live. It's sort of lost that identity.

Interviewer: Yeah, that's a shame.

Respondent: It's just a name on a map.

Interviewer: That's sad. Yeah.

Respondent: So in one way it is, but then I suppose then you get towns like [town] that will then exploit those people and they'll go there if they want to belong to community groups.

Interviewer: Yep, yep. And so what about the word community – what does that mean to you?

Respondent: There again I wouldn't say [town] would have a community. I regard communities as towns, like [town] – obviously [town], [town], [town], [town] – although [town] I wouldn't class like that, because I think [town] is more of a through area, but definitely [town] and [town].

Interviewer: Yes, bigger and a through area. And so those are smaller, as sort of communities that sort of...

Respondent: Hmm, yeah.

Interviewer: Yep. Yep.

Respondent: [town] and [town] would be communities. [town] is just a name on like the roadmap to me.

Interviewer: Hmm. OK. And so your closest community then would be [town] really you were saying?

Respondent: Hmm, that's right. Yes. Yes.

Interviewer: Eight k away.

Respondent: Yes. Yes.

Interviewer: OK. Now I wanted to talk a bit about physical activity, and as I was saying it's a very broad term, and it's really individual what it means to you. So, I was wanting to know what the term physical activity might mean to you?

Respondent: Well I'm quite a competitive person, so to me physical activity is pushing myself, and I like to have a goal. I have to see a result. So it's not actually what perhaps I look like, but I have to win, or I have to have a result at the end. I couldn't just walk. To me the walking group would not be physical activity – if I say that in a nice way.

Interviewer: Yes, yes.

Respondent: Yeah. Hmm.

Interviewer: Yes, this is very important – what it means to you.

Respondent: Yeah. Oh, yes.

Interviewer: And it's often... it's different to each person. Yeah. But no, it's a good contrast, because I understand what you're saying.

Respondent: Yes. So I know that sounds very selfish in a way, but to me I wouldn't... so if I see someone walking I wouldn't regard that as physical activity, because I would do that regardless. But physical activity is just pushing yourself that little bit harder, and being competitive as well.

Interviewer: Yep. Yep. OK. And do you consider yourself to be physically active?

Respondent: Yes.

Interviewer: OK. Can you tell me why? [Laughs].

Respondent: Well I play competitive tennis once a week in [city]; I ride a mountain bike probably once a week, and alternatively with a road bike; I run a ten k race once a year, and obviously then I'm running once a week as well.

Interviewer: Yes, to prepare and keep yourself fit for those sorts of events.

Respondent: Yeah. So I try to do like a ten k competitive race once a year. I've been doing that [x] one, which is good. I like that one.

Interviewer: Hmm! Oh yes, I thought that...

Respondent: I like that one. [Laughs].

Interviewer: Yeah, yeah.

Respondent: I use to do the [x], but I don't like that one anymore. So my goal would be if I can do a ten k race once a year, so obviously to do that I've just got to do some training. And as I say, just do a bit of bike riding.

Interviewer: Hmm. And where does it fit into your life? Do you consider it as a high priority, a priority, no interest at all?

Respondent: High priority in that our weekends are structured around it.

Interviewer: OK.

Respondent: Yes. So every weekend, because my husband does it as well, so every weekend we either do a road bike or a mountain bike, depending on the weather, and then we'll fit in a run. And then I've got my tennis once a week, which is a regular thing as well.

Interviewer: OK. That's good. We'll go on and talk a bit more about that. Now in our research, when we think about physical activity, we talk about any activity that lasts for at least ten minutes, and that's the evidence of health benefit effect, and that you feel maybe a bit of your heart working – it might go up, the body might be working harder, there might be a bit of huffing and puffing, or getting warm, very beneficial when it's winter. [Laughs]. It's very handy.

And I wanted to talk about this type of physical activity over the last couple of weeks, and I'm just going to go through different parts of your life, not that... it was just to make sure that we cover it all really. So, now

you're working at the Library – that's your main employment off the farm?

Respondent: Yes. Yes, yes. That's the only job I have, yes.

Interviewer: Yep, yep. OK. Do you work on the farm as well?

Respondent: I do the accounts.

Interviewer: OK.

Respondent: Yes, just the accounts – and fencing, and just generally helping like that. Yes.

Interviewer: Yeah. So I'll let you work out what you consider is your work activity then – employed and activity. I mean we'll cover the other activities on the farm, but we'll... you can work out where you want to put it.

Respondent: I'd put... well I volunteer at the Library, and then I get part-time work.

Interviewer: Oh, OK.

Respondent: So I volunteer on a Tuesday, which is today, so I volunteer there for two hours a week, and so then I structure my other activities around that. Is that what you mean?

Interviewer: OK. Yeah.

Respondent: Yeah. And then I get relief work as well at the Library.

Interviewer: Oh, OK. So shall we focus on the library fit for this particular...?

Respondent: Yes, alright then. OK. That's fine.

Interviewer: And then have a look at home?

Respondent: Right, OK then. Right.

Interviewer: But I'm also happy if you want to add in the accounts in terms... anyway.

Respondent: Hmm.

Interviewer: That's the thing, you know people do very broad things in their life, it doesn't quite...

Respondent: Well just to say I'm a very structured person, I'm a control freak. So I have a set pattern to my life, and as I say I've got relief at the Library for two hours a week, and then... sorry, I volunteer at the Library for two hours a week, and then I've got relief when needed. So then I am able to, when they ask me to do relief, I then can drop some other things and what have you.

Interviewer: Yes, you've got...

Respondent: But I've sort of specified that I need Wednesdays for tennis. So I'm very structured that Wednesday is my tennis, but I can relieve other times.

Interviewer: OK. So thinking about that Library work, what would you... OK, so in the past two weeks have you ever been active at all at work for at least ten minutes of the time?

Respondent: So the last two weeks, yes – have I been active in the Library you mean?

Interviewer: Yep, yep.

Respondent: Well you're always on your feet – is that what you mean?

Interviewer: Yes.

Respondent: I mean you're on your feet all the time, and you're going around stacking books.

Interviewer: That's the sort of thing. Yeah, yep.

Respondent: Yeah. So it's not as if you're sitting at a computer in other words. Yeah.

Interviewer: Yep, yep, yep. And would you call... OK, so could you tell me about what you did?

Respondent: Yeah. Well I do a story time for the littlies, so that's basically sitting down for sort of 20 minutes and interacting with the kids. We do a few craft activities, so that could mean getting up and down and that. And then it's basically just standing at the desk and taking books in, and taking books out, and stacking.

Interviewer: Great. So if you had to think about the intensity of that exercise, what would you call it? Would it be steady, or moderate, or vigorous?

Respondent: Steady.

Interviewer: Yep. And how long for?

Respondent: As I say I'm only doing the hour and a half on that actual task, and then sort of the 20 minutes with the reading.

Interviewer: Yep, OK. And how often?

Respondent: And that's once a week.

Interviewer: That's once a week.

Respondent: Yeah, once a week.

Interviewer: And when – no, you've already said that it's...

Respondent: Yeah, Tuesdays.

Interviewer: ...it's Tuesdays for a couple of hours. And is this what you usually do, or is it one off?

Respondent: This is what I will be doing, yes.

Interviewer: Yes. And does it change depending on the season or time of year?

Respondent: No.

Interviewer: Hmm, OK. And if you wanted to, is there anything that would help you be more active at work?

Respondent: No.

Interviewer: It sounds like you're pretty active anyway.

Respondent: Yeah. No, that's right.

Interviewer: OK. In the past two weeks have you walked or cycled for at least ten minutes at a time to get to or from places?

Respondent: [Laughs].

Interviewer: Yes. [Laughs].

Respondent: Yes, I'd say that. Yes.

Interviewer: And can you tell me what you did?

Respondent: Well I went for a 30 minute run the other night – because it's all weather dependent at the moment, so you'd look out and think oh, it's not raining, you know.

Interviewer: Hmm. Yeah, you go yes, good. Yep.

Respondent: I went for a mountain – 25k mountain bike ride around the bush; played tennis, three sets of tennis. Oh, and then I have a social tennis on Monday morning, so I played some tennis then. I do some gardening.

Interviewer: OK, so keeping to the walking and cycling – we'll cover the other bits later.

Respondent: Yeah, yeah, sorry. [Laughs]. Yeah. No, that's alright.

Interviewer: So thinking about those activities of walking and cycling that you talked about, what would you say the intensity of both of those activities were? Steady, moderate, or vigorous?

Respondent: Yeah, I'd have to say moderate.

Interviewer: OK.

Respondent: You know, I mean I have to enjoy it. I don't want to... yeah, moderate. Hmm, hmm.

Interviewer: And the duration of both of those activities?

Respondent: Yeah, well as I say the run was about 30 minutes, and the cycling would take about an hour and a half.

Interviewer: Uh-huh, uh-huh. And how often?

Respondent: The cycling once a week, and the running once a week. Yes.

Interviewer: Uh-huh. And when did you do it?

Respondent: Well the run we did... oh, well so the cycling we did on Saturday, and then I probably did the run, probably Sunday night say.

Interviewer: OK. And why did you do it at that time?

Respondent: Weather dependent – yes, the weather was good – and before tea. [Laughs].

Interviewer: Yes, yes, so before it gets dark.

Respondent: Yeah, and before it gets dark, of course. Yes, weather dependent, and before a meal, you know.

Interviewer: Yes. Yes. OK.

Respondent: That's right.

Interviewer: And where did you do it?

Respondent: Well both those activities were done around [town] , in the forest. Hmm.

Interviewer: Uh-huh. And why did you do it there? What attracted you to it?

Respondent: Basically because we know the tracks, so we know what condition they would have been, and when you're running at this time of year with trees, and if it gets dark, you don't want to go somewhere that you haven't been before.

Interviewer: No, that's right.

Respondent: And we enjoy the terrain because there's a bit of up, and a bit of down, and it's not too much. Hmm, hmm.

Interviewer: And is it usually what you do, or is it one off?

Respondent: Yes. Hmm.

Interviewer: Yep. And does it change depending on the season or time of year?

Respondent: It does. Hmm.

Interviewer: Hmm, OK. So in what way might it change?

Respondent: Well obviously the time of year we might go – as I say if the days are longer you would drive, and you would probably go to a different area, and you'd just give yourself that bit longer. And also the weather's better, so you don't get as cold when you're riding, because obviously when you ride, when you do stop you do get cold. So you have to think of that factor as well – when you stop running, and just the length of time from when you finish running to when you get a shower, you know things like that, because in the summer we run at [town] and then we have a swim and what have you, so it's a little bit different.

Interviewer: Yeah, so it works out really well.

Respondent: Hmm, hmm.

Interviewer: Yep. So that's why you would prefer to be able to cycle closer to where you are.

Respondent: Hmm, oh exactly. Yes, because of the length of days, and just being able to...

Interviewer: So can you actually cycle from your place?

Respondent: Yes. Yes.

Interviewer: OK.

Respondent: Yes.

Interviewer: And that being [town] ?

Respondent: Yeah, [town] .

Interviewer: But also in [town] ?

Respondent: Oh, and [town] as well, yes.

Interviewer: Excellent. OK.

Respondent: Yes. Hmm.

Interviewer: Yep. And if you'd wanted to, is there anything that would have helped you use cycling or walking to and from places?

Respondent: Like what?

Interviewer: If you wanted to do more, what might have helped?

Respondent: Oh, I see. Yes, are you getting towards bike tracks and things like that, are you?

Interviewer: That sort of thing, yeah.

Respondent: Yeah.

Interviewer: Or for walking, like the footpath. [Laughter].

Respondent: Yes. Well obviously because [town] hasn't got the infrastructure we're never going to get footpaths, so it's not something that you would get up and just go for a walk. So I prefer the mountain bike because you're off the road, and I don't really enjoy the cycling as I say, the road cycling around on [x] Highway as much.

Interviewer: Yeah, you wouldn't go on the road?

Respondent: No. No. Oh well we do, but we try to go on the less busy times.

Interviewer: Yes.

Respondent: So you sort of go early, or a time when it wasn't as busy.

Interviewer: Yes. So you prefer to be off road.

Respondent: Hmm.

Interviewer: Yep, fair enough. And those roads are quite enough, the off road?

Respondent: Hmm. Yes, yes. Hmm.

Interviewer: OK. Now in the past two weeks have you been active around the house or yard – so inside or outside your home – for at least ten minutes of the time?

Respondent: Yes. Yeah.

Interviewer: OK. Can you tell me about what you did?

Respondent: Well the basic housework – so that's just the basic things. And then we've got quite a large garden, so this time of year not as much, but it's still – like the other week I went around and just tidied up, and raked a few leaves, and pulled a few weeds that were there.

Interviewer: Hmm. There's always something to find. [Laughs].

Respondent: A few branches that were hanging off, and just generally... and some old plants that needed pulling out. Yeah, so just generally things. Yeah.

Interviewer: Yeah. And would you... what would you call the intensity of that?

Respondent: That would be moderate again.

Interviewer: Hmm. And duration? How long would you have been out there?

Respondent: I usually spend about an hour and a half.

Interviewer: OK.

Respondent: Yeah. Hmm. I lose track of time. I don't wear a watch when I'm outside. [Laughs].

Interviewer: [Laughs]. That's fair enough.

Respondent: It's bad. [Laughs].

Interviewer: And in the last couple of weeks how often would you have done that?

Respondent: Being winter only once.

Interviewer: Yes. Yes.

Respondent: Yes, being winter. Hmm.

Interviewer: And the housework?

Respondent: Oh, slack. Oh, only about once as well. Yes.

Interviewer: Yes.

Respondent: Well there's only two of us.

Interviewer: There's only two of you, yeah exactly.

Respondent: There's only two of us, so I'm not the great house worker.

Interviewer: Yes, and you'd call that sort of fairly steady or moderate exercise?

Respondent: That would just be steady. Yeah.

Interviewer: Yep, yep. And how long might you have done that for?

Respondent: Say an hour. Hmm, yeah.

Interviewer: And thinking of those two activities, when did you do them?

Respondent: Let me think?

Interviewer: So sort of time of day.

Respondent: Yeah, I'm just think... I'm more of a morning person, so it would have been in the morning, and usually is sort of straight after breakfast. Yeah, that's right.

Interviewer: And why did you do them that time?

Respondent: Because a bit of guilt and thinking I should do it. [Laughs].

Interviewer: I'd still be like get over that. [Laughs].

Respondent: Yeah, that's right. Yes. No, I think it was guilt more than anything.

Interviewer: [Laughs]. OK. Now who did you do it with?

Respondent: On my own. Hmm, hmm.

Interviewer: OK. And is this what you usually do, or is it a one off?

Respondent: No, I'm usually on my own. Yeah.

Interviewer: Yeah. And you usually do... like you've talked about those two activities, you usually do them?

Respondent: Hmm. Hmm.

Interviewer: Yep. And does it change depending on the season or time of year?

Respondent: No. No. No, that would be my job to do that.

Interviewer: OK. And if you'd wanted to, is there anything that would have helped you be more active around the home, either inside or outside?

Respondent: No.

Interviewer: OK. Now, have you been physically active for at least ten minutes during your leisure or spare time – so this is picking up any activities we haven't talked about already? The tennis was one.

Respondent: Yeah, yeah, tennis. I can't sit still – I've got a book, but I can't read it. So I need to be thinking of things. Yeah. Hmm. Hmm.

Interviewer: So can you tell me what you did in the last two weeks?

Respondent: Oh, well I made some sewing – I made some neck warmers, because I went over to the snow, so I made some neck warmers to take. So [laughs] I was sort of, you know... yeah, so I try to do a bit of sewing, but I'm not good at it. But I just have to be doing something.

Interviewer: Yeah. And tennis?

Respondent: Hmm?

Interviewer: How often?

Respondent: Well I've got social Monday mornings, and competitive on the Wednesday.

Interviewer: Yep, OK. And the intensity of those activities?

Respondent: Yeah, I'd still only say moderate. Yeah. Hmm, it's only word – moderate.

Interviewer: OK. And how long?

Respondent: The Monday morning is an hour and a half, and the Wednesdays would go over about three hours.

Interviewer: OK. And what time of the day?

Respondent: No, they're mornings again.

Interviewer: Yeah, they're the mornings.

Respondent: Yeah, mornings.

Interviewer: OK. And why do you do them at those times?

Respondent: Social so I can practice, and the competitive because as I say I like to stretch myself a little bit.

Interviewer: Hmm, hmm. And any reason why you do it in the morning versus any other time?

Respondent: That's when it's offered, and also the morning is more suitable because you get up and go I think.

Interviewer: And where?

Respondent: Well the social is at a place called [town] , so that's very convenient. And the competitive as I say is in [city].

Interviewer: Oh, OK. So it's only what, a couple a kilometres to [town]?

Respondent: Yeah, that's right. Yeah. So we're just fortunate. When we first came here we had the tennis courts at [town] , and I played there, but they've gone now.

Interviewer: Oh.

Respondent: So it was just a social that I played. And then I joined the [suburb] Tennis Club, and now I play in [city].

Interviewer: OK. And how would you have got there?

Respondent: By car of course. Yes, this is the only inhibitor – and I'm the only one coming south of [town] that plays for [suburb], which I find very strange.

Interviewer: Hmm.

Respondent: Because [woman] used to live – I think she was at [x] River – she now lives over at [town]. So I'm the only one that comes south of [town] really, that plays tennis at [suburb].

Interviewer: Oh, that's interesting.

Respondent: Hmm. So it's not very strong. It used to be very big in [town] – it's not now.

Interviewer: Oh, that's interesting. Hmm. And OK, so you would drive there, and who with?

Respondent: Well that would be myself, you see.

Interviewer: By yourself. Yeah.

Respondent: Yes. Hmm.

Interviewer: OK. And then you sort of meet up with people there. And is that what you usually do?

Respondent: Yes.

Interviewer: And does it change depending on the season or time of year?

Respondent: No. No.

Interviewer: That's good that it keeps on going throughout the year though.

Respondent: Yeah, and we do a three school term.

Interviewer: Oh, OK.

Respondent: So it's only summer when we don't – well as I say getting to your question, but it's a three school term. So we're basically playing, instead of 12 weeks, at the 12 week...

Interviewer: Yes, so that's why you've had a break as well.

Respondent: Yeah, so we've sort of just had the two weeks off, that's right.

Interviewer: Yep, yep. And if you'd wanted to, is there anything that would have helped you be more active in your leisure or spare time?

Respondent: Living closer to an area like [suburb], I would have probably gone... oh, now I do an aerobics class at [town] on a Tuesday night.

Interviewer: Oh, OK.

Respondent: Sorry, I forgot to tell you before. But if I lived closer to [suburb] I would probably do another one of those classes. But there would be no way I could justify driving half an hour up to do an aerobics class. But I can justify driving half an hour to play tennis to[suburb].

Interviewer: Yes. OK. OK. And just to pick up on the aerobics exercise, you were saying that's at [town] ?

Respondent: Yes, yes, and that's at [town].

Interviewer: And intensity of that?

Respondent: Moderate to...

Interviewer: Yeah,

Respondent: Yeah, a bit up. Yeah.

Interviewer: And how long for?

Respondent: And that's for an hour.

Interviewer: In one evening?

Respondent: Hmm, one evening. Yes.

Interviewer: OK. Now, I want to talk a bit about the physical environments now, because we've talked about where you live and your physical activity, and the physical environments are where you live, work, and play basically. And so when I talk about physical environment I mean all the physical things surrounding you, like the roads, the trees, houses, shops, traffic, whatever.

So some of these are things that people who are living in cities, in urban areas, actually say are important for their physical activity – so we're interested in finding out whether it's relevant for you in rural areas. Can you tell me about how easy or difficult it is to be physically active in your area?

Respondent: I would say it was difficult.

Interviewer: OK. And why?

Respondent: And why? Because we don't have footpaths, we've got a busy road. But saying that, if people think of walking – I'm thinking of walking – but if you wanted to take the dog for a walk, or if you wanted to walk on paddocks, well that's a different thing isn't it. But I'm sort of thinking of city people – do they think of exercise as being able to walk out their door and whatever.

Interviewer: OK, so if you were walking, are you saying that you would have access to walking on your property?

Respondent: Yes. But I wouldn't just go for a walk like a city person – just out the door and go for a walk.

Interviewer: Hmm. So tell me what you usually do when you go for a walk then?

Respondent: So I wouldn't go for a walk. I would get in the car and you'd have the bike in the car, or you'd have your running... and you'd drive somewhere, and then you'd go for a run.

Interviewer: Yeah, and then you'd go for your run or your bike ride.

Respondent: Yes. Yeah. So it's prohibitive in that you can't actually go from your doorstep.

Interviewer: Yep, yep. OK. So I just want to talk about some aspects of that and availability and accessibility of places to be active is the first one – so if I were to ask you about places to be active in your area, what sorts of things would you tell me about?

Respondent: Well, we're so fortunate to have the [town] Sports Centre.

Interviewer: Yes. Yes.

Respondent: So I mean you've still got to have a car from where I live, or you could ride your bike, but so I mean the worst scenario you could actually ride your bike.

Interviewer: How many kilometres is it from where you live?

Respondent: Six, I'm thinking. It's a bit over five, I think. Yeah, about six. Hmm. So I mean if we didn't have that, as I say...

Interviewer: Hmm, and that's got a whole range of activities.

Respondent: It has. It has, hmm.

Interviewer: OK, so that's the main one. And anything else in terms of parks, or walking tracks, or cycle-ways?

Respondent: No, not really. As I say [town] we're lucky because we've got the walking/cycling track and the running track around the bay there, so we use that a lot of the time when we go for runs.

Interviewer: Yes. OK. And access to places to be convenient, like you were saying about the Sports Centre, is it accessible in terms of opening hours and cost, and things like that?

Respondent: Yes. Yes.

Interviewer: OK. And does having places to be active influence whether you're active or not?

Respondent: It does. Definitely. Hmm.

Interviewer: Hmm. And if you wanted to be more active are there things or places that would help if they were more available, or more accessible?

Respondent: Well, I think because we tend to do activities on our own, because we bike ride and run – which are in a way anti-social, they're not group

activities I suppose – we're lucky. But if you wanted to join more team sports I think you'd be restricted.

Interviewer: Yes.

Respondent: And that's why I think around [town] area, because there's no local tennis courts, and there's no local – just thinking – oh, there's a bowling green obviously. But if the facilities are there then people would be more inclined to do it, you know.

Interviewer: Yes. Yes, yes. No, it's important. Yes, so it's having a look at facilities. Now destinations – if I were to ask you about places that you could walk or cycle from your home, what sorts of things would you tell me about? And you already have, that you can't actually directly walk or cycle from your home.

Respondent: Yes. Yeah.

Interviewer: Yep. So anything that you do then requires getting in the car and putting the bike in or whatever?

Respondent: That's right.

Interviewer: So does having, or not having, destinations influence whether you're active or not?

Respondent: It does. Oh yes. Yes.

Interviewer: It would make it a lot easier.

Respondent: And I think people – that's the easy cop out. You know, they say, "We can't do this because we haven't got that." So I think that's...

Interviewer: Yeah, and it makes it harder to do something, isn't it?

Respondent: Yes. So you can't say to people... I mean you can say to people today go for a walk, and they say that's easiest form – all you need is a pair of runners, and you go for a walk. But then someone will say we've got a busy highway, the road's uneven, we haven't got a footpath – I mean they'll always put a barrier in front of you. So unless you provide someone with an asphalt path, and lovely weather – you know I mean it's too cold, it's raining. So I mean you'll never... you know, so... hmm.

Interviewer: And if you wanted to be more active, would it help if there were more destinations within easy walking or riding distance?

Respondent: Yes. Oh yes. Yes, definitely. Hmm. But it's just the cost factor, and the...

Interviewer: Yeah, exactly. In terms of whether it's likely to happen or not. Yeah. Well what may be some... I don't know, sometimes there's... it's sometimes talking to people that they come up with some ideas that aren't like changing the world, because we all know that's not going to happen, but some simple things that might just make it a little bit as a start to...

Respondent: And if people see people doing it that helps as well. So you just need someone to actually start off the activity, and then as the activity starts then the infrastructure comes in as well. But you know, so you don't

necessarily have to have the infrastructure to start it. If someone starts riding a bike, and then another person, and then the infrastructure might come.

Interviewer: Hmm. So for you, you've actually really sorted out what is important for you in terms of activity, and how you're going to do that.

Respondent: Yeah, well we decided rather than sitting around and saying where can we ride our bike, where can do that – but saying that we're lucky to have a car that we can put the bikes in and take them, because it's just getting that A to B scenario.

Interviewer: Yeah, absolutely. OK. And I wanted to talk a bit about neighbourhood function or its design. So if I asked you about the built features of your environment, the ones that are not natural, the manmade features, what sorts of things would you tell me about?

Respondent: Well as I say [town], I can't... because we haven't got any structure, so I suppose [town] and [town] you'd probably be relating there. We're not really community minded people in a way, we tend to keep... like when the kids are at school you tend to get involved in the school, but we're not really community people.

Interviewer: Hmm. And you were saying about [town] , just in terms of footpaths, there isn't lighting?

Respondent: No. No. No, that's right. No, no. No, I think no, that's right.

Interviewer: No. And street connectivity, like if you wanted to go for a walk for instance, can you actually sort of... is there anywhere off the main road that you can walk? Is there sort of areas that... streets that connect?

Respondent: Yes. The Landcare people have actually got a bit of an off road circuit. Yes, so you can actually walk that. Hmm. Yeah, and quite a few people do that.

Interviewer: Is that a very large area?

Respondent: I've never actually walked the whole length. I'd probably say about a good k, and that goes around the water or dairy(?), so it connects sort of one area to the other.

Interviewer: Hmm. OK. So do the built features of your area influence whether you're physically active or not?

Respondent: It would.

Interviewer: It would?

Respondent: But we're people that...

Interviewer: But you've overcome it. [Laughter].

Respondent: ...that it hasn't. But there are lots of people that would just sit there and say there's nothing to do, which I can see – I can see that.

Interviewer: Yes. Yes. And if you wanted to be more active, are there any built features in your area that would help?

Respondent: No. No.

Interviewer: Hmm, for you?

Respondent: For us, yes. Hmm.

Interviewer: Yeah, that's right. Now the other side of it is the natural part of the environment – if I were to ask you about the aesthetic qualities or the attractiveness of your area, what sorts of things would you tell me about?

Respondent: Well I suppose it's the water.

Interviewer: Hmm, and the beautiful river.

Respondent: Oh yeah, I mean that's sort of... you always sort of think oh, why do I live here – but then I've always lived near water, and even when we lived in Ireland we overlooked a lake as well. So I suppose it has to be the water. Hmm. Yeah.

Interviewer: Uh-huh. Hmm. And do you think your local environment is aesthetically pleasing and attractive? When you get home?

Respondent: Yeah, I think it is. Yes, yes. Hmm.

Interviewer: Yep. And if you wanted... do the aesthetics of your area influence whether you're physically active or not?

Respondent: Oh, it does, because it makes you want to get outside and do something.

Interviewer: Yeah. Yep. And you've got a – it sounds like a mixture of bush and water that you do your activities by too.

Respondent: Yes. Hmm.

Interviewer: And so we've talked about the water and the access – the bush access, is that through... you were saying that you mountain bike?

Respondent: Yes, hmm.

Interviewer: And they're off road tracks – are they roads, or they're part of a State Forest area?

Respondent: Forest? Yes, yes. So like we can cycle from where we live, but it means just going on a road for about two k, and then you're into the actual forest. So it's the forest plantations. It's just where they have the tracks around the outside of the forests, so we cycle through those, and run through those.

Interviewer: And if you wanted to be more active are there any features of your area that might help if they were more aesthetically pleasing or attractive?

Respondent: Not really. Yeah, I'm just thinking – I mean yes, like at [town] where they've actually done the foreshore and they've got the walk around, and the bike/running track around the bay, and that was the Landcare and Government funding – I mean that would be nice to have around the

water. And when we go to [town] it's nice to run around the water and that. So I mean that would be the only thing I would say, and they're still having problems in [city] trying to do that aren't they. [Laughs].

Interviewer: [Laughs].

Respondent: But I mean that's the only thing I would say, you know. And they reckon that if the Government does provide walking tracks around nicer areas that people would be more inclined to walk, which is fine. But I can't justify the money – I couldn't justify the money for that.

Interviewer: OK. Now the last area is safety – if I were to ask you about safety in your area, what sorts of things would you tell me about?

Respondent: As in doing exercise?

Interviewer: Hmm.

Respondent: Yeah. Oh, well just the road obviously, not being able to cycle on the road. But I mean that's life, isn't it? I mean cars rule the road, don't they, so I mean that's life.

Interviewer: So does road safety influence whether you're physically active or not?

Respondent: Oh, it would. Yes. Oh, yes. No, definitely.

Interviewer: Yep. So you actually obviously choose to not go on the road as much as you can, and go off road.

Respondent: Hmm. Yeah. Hmm, that's right.

Interviewer: And any... does personal safety influence whether you're physically active or not?

Respondent: Oh yes. Yeah. Yeah. I mean you're riding on your bike, and you're always thinking what's that guy behind – is he going to stop or whatever. Yeah. Hmm.

Interviewer: Yep. Yeah. So I mean that's one of the reasons most people don't do activities in the dark, particularly when there's no lighting.

Respondent: Oh, yes. Oh, I mean you know...

Interviewer: You just don't do it. [Laughs].

Respondent: Yeah, yeah. That's right.

Interviewer: And if you wanted to be more active, are there any personal or road safety issues that if addressed might help you to be more active?

Respondent: No, I don't think so.

Interviewer: Hmm.

Respondent: Only because we're lucky to have off road areas. But if you did live in an area that you had to be on the road, well obviously that would be (indistinct word – 39:06).

Interviewer: Yes. That would be, yeah. Yep.

Respondent: But we're just lucky that we can actually go to areas that we don't have to – or you go those times like, you don't go on a long weekend, on a Friday, or a Sunday. You know you just sort of pick your times when you go.

Interviewer: Yep. And last question there before we finish up, is there anything else that you'd like to tell me about today that we haven't covered, about where you live, or your activity?

Respondent: No, not really. I don't sort of...

Interviewer: Your physical environment?

Respondent: I mean I know this is a rural sort of talk, and city, but I don't feel as if we're that isolated, but only because I have access to a car. I think if you didn't have a car I think it would be different. And even though people sort of... I know at tennis they sort of think oh, you've come all this way, but to me it doesn't sort of... you know, like something that I want to do. You know, that I'm not being forced to do.

Yeah, I don't... people put so many boundaries on things, don't they? I don't know if... you know they... I don't know. Yeah. Hmm. No, I can't think of anything else. I think that's everything.

Interviewer: No, that's excellent. Thank you very much.

Respondent: As long as... yeah.

Interviewer: That's great.

Date: 18 October 2011

Duration: 26 minutes, 29 seconds

Interviewer: [interviewer name]

Transcriber: [transcriber name]

OK the first thing I'd like to talk to you a bit is a bit about the place that you live in and what it's like? So could you tell me about what it's like living where you do? Your likes and your dislikes?

Ah, I don't really have a great deal of dislikes. The only major dislike is cold winters. I like it because it's not in [city] but it is only 45 minutes away from [city].

Yeah. Do you live around the [town] area? Is that right?

Yep. And you know it's just a nice part of the world to live in. Um, we've got a lake close by which you can go swimming in and ah got a little golf club. And yeah, it's just a nice community.

It's lovely up in the [x] Highlands. So can you tell me a bit about, just about the property you live on?

Yeah, it's an 1100 acre farm. Um, 150 hectares of it is irrigated. Um, we milk about 500 cows in the spring. And we milk about, oh 200 cows through the winter. Um, yeah, so we've got a bit of dry land and a fair bit of irrigation.

So how big was it overall did you say?

1100 acres all up.

That's a very nice size. It would keep you busy.

Yeah, it does keep us very busy.

And the length of time you've been there.

Ah, since I was five.

Yeah. So this is the family farm is it?

Yes it is.

And your reasons for living there?

Ah, work.

Yeah. And what is your distance from your nearest town or township?

Ah, we live within the town boundaries.

OK. In terms of the [town] boundary, how far out of actual [town] do you live?

Well in a direct line across the river it is about 100 metres.

Wow. OK. So you are like close in.

Yeah, yeah. [2:28] is on one side of the river and our farm is on the other side of the river.

Yep. Now in research with men living in urban areas, um people often refer to their local neighbourhood. And I was interested in what the term neighbourhood means to men who live in rural areas. And indeed if it is a relevant term. So what does the word neighbourhood mean to you?

Yeah, not much mate. You know, I mean I've got friends that live at the other end of the State. I've got friends that live 100 metres away. So I mean, yeah neighbourhood's I s'pose where you live. But I don't think the term really is that relevant to the country because –

Yeah, what about the word community? What would that mean to you?

Um –

And is that more relevant?

Yeah, community's definitely more relevant. Unfortunately um community's lost a bit of its cohesion, I s'pose is the right word for it. Um, because ah a lot of people have moved on. Um, farms have got bigger. Um, people have got more efficient, so there's less people about. So, I mean community events like the local show and that sort of thing are very important, just to bring the community back together because everyone is very busy and we don't get to see each other as often as we should.

Yeah. OK. So if you needed to put a distance around community, how big a distance would that be?

Oh I s'pose a 20k radius.

OK. OK. Now just going back to neighbourhood, what that be relevant at all?

No.

No. OK. That's fine. Now I'm just going to go onto physical activity and talk a bit about that. And as I was saying, it's a very broad term and it's really you know dependent on what the individual sees, sees activity as being. So what does the term physical activity mean for you?

I associate physical activity with work. Um, pretty much. Outside the workplace I do very little physical activity now. Ah, yeah I mean when you work in the winter, we don't do as much physical activity, but during summer um, you know I start work at 5 and sometimes don't knock off until 9. And most of the time I'm on foot and going.

Yes. Yes. So you are busy enough all the long hours of the day.

Pretty much.

So do you consider yourself to be physically active?

Yes.

Yeah, yeah. Because you are really on your feet most of the time.

Yes. Yeah.

And where does physical activity fit into your life? Is it of no interest? Or a priority? Or a high priority?

Oh it's a necessity more than anything. Um, if I got to a stage where I wasn't able to do physical activity, well I wouldn't be able to work.

Yep, yep. So in our research – we are going to talk a bit more about physical activity – in our research when we think about physical activity, we think of it as any activity that lasts for at least ten minutes. That's the evidence of health benefit effect. And it causes your body to work harder than normal. So your heart rate might go up. You might breathe a little heavier or you might warm up a bit. Especially in those hills in the [x] Highlands.

Yes.

Um, so just thinking about this type of physical activity during the past two weeks. So in the past two weeks - I'll just go through different parts of your life and not that I am

meaning to segment your life into bits, but mainly just to make sure that we cover everything –

Yeah, yeah. Alright. Yep.

OK? Yep. Yep. So in the past two weeks have you been active at work at all for at least ten minutes of the time? And obviously, you have. So can you tell me about what you did?

Ah, well, ah milking cows, drenching cows, um shifting flood irrigation, um loading calves, um yeah that would probably round it out.

Yep. Now thinking about those activities, could you tell me the intensity of them? Would they steady, moderate or vigorous?

Some steady, some vigorous.

Yep. And the duration? How long would you be doing those activities at any one time?

It varies. Some for three hours, can be.

Yep. And how often? That would be every day?

Every day.

Yep. And you're working on the farm how many days a week?

Um, between five and seven.

Yes. So it varies depending on the season. And I gather Spring would be a very busy one?

Yeah. That's exactly right. From September through till January it's seven days a week.

Yep. Yep. Um, and is this what you usually do? Or was it one off?

Usually.

Pardon?

Usually, usually.

Yep. And does it change depending on the season or time of year?

Yes.

Yep. And you've already said that in winter that, the, well all year round the tasks vary, but the intensity of how much you have to work certainly, there is certain times of the year that it is really a lot busier.

Yeah. Very –

Winter is probably the time that it would be slightly less busy?

Yep. That's it.

Yep. Yep.

It's busy September through till probably May - oh, May, June, July it's less.

Yep. And if you wanted to is there anything that would have helped you be more active at work? Have to ask the question, but you are very active at work.

Ah, no, not really.

Absolutely. Um, in the past two weeks have you walked or cycled for at least ten minutes at a time to get to or from a place?

Um, yes.

OK. Can you tell me about what you did?

Um, well golf was one. Um, and then the other is um walking behind cows.

Yes. Yes. So thinking about those two activities, what would be the intensity of them? Would they be steady, moderate or vigorous?

Um, steady.

Yep. And how long would you do those activities for?

Golf was for five hours and um the cows, behind the cows was probably 40 minutes.

Yep. And how often would you do those activities?

Golf, not as often as I'd like.

Yes.

Probably, golf was probably once a month. Um, and the other one, oh it can be up to five times a week. Could be.

Yep. Um, where do you play golf? Is there a golf course close to you?

Yeah, there's a golf club at [town]. It's ten minutes down the road.

Yeah. Yeah. Um, and when did you do those activities? Obviously the cows are during the day and during the week, and golf?

Ah on a Saturday.

Yep. Yep. And were you alone or with others doing those activities?

Alone with the cows and others doing golf.

Yep. And is that what you usually do? Or is it a one-off?

Oh, usually I s'pose, yeah.

Yeah? And does it change depending on the season or the time of year?

Yes.

Yep. So ah, the cows, yes, so it may change depending on the – you talked about two different activities. So, with golf um how does it change with the seasons?

I'm a fair weather player.

Fair comment. And the cows?

Ah, no I don't suppose the cows change too much. Um, I mean it does change a bit but not a lot.

Yep. OK. And if you wanted to is there anything that would have helped you walk more to get to or from places?

No.

OK. OK, in the past two weeks have you been active around the house or yard? i.e. at home but either inside or outside. I guess that's the closer outside boundary, the home boundary?

Ah, yes.

OK. And can you tell me about what you did?

Um, vacuuming, um hedge trimming. And that's about it.

OK. Now thinking about those activities, could you tell me the intensity? Whether they are steady, moderate or vigorous?

Oh vacuuming is moderate and hedge trimming would be moderate too.

And how long would you have done those activities for?

Oh vacuuming, probably twenty minutes and hedge trimming half an hour.

OK. And the frequency? How often would you have done that?

Hedge trimming is once a year. Vacuuming is once a week.

Yep. And when would you have done those activities?

Ah Mondays, vacuuming. And hedge trimming was Sunday.

And is there a particular reason why you would have done it on that time?

Ah Monday my wife is at work with the kids. And there's that spare half an hour on Sunday. So,

Fine. And would you have done those activities by yourself or with others?

By myself.

And is this what you usually do or is it one off?

Ah, well the hedge trimming is once a year, and yeah usually vacuum on Mondays.

Yep. And does it change depending on the season or time of the year?

Vacuuming no. Hedge trimming, well it's only done once a year.

Yep. And if you'd wanted to is there anything that would have helped you be more active around the home either inside or outside?

No.

OK. Now have you been physically active for at least ten minutes during your leisure or spare time? Now that's just to pick up anything we might have missed?

Um, only the golf.

Yep. Yep. And we've talked about that one. So that's fine. Now I want to go, the last bit is about the physical environment. Um we've talked a bit about where you live and your physical activity. And now we want to talk about the physical environments that you live, work and play in. Now, and when I talk about physical environment, I mean all the physical things that surround you. So it can be the roads, trees, paddocks, um, traffic. And some of these things are things that people in urban areas have said may be important for their physical activity. So we're interested in asking whether it is relevant for you?

Yep.

Yep. So first up is can you tell me a bit about how easy or difficult it is to be physically active in your area?

Ah, easy because it's work related and also, well the only activity I really do outside work nowadays is um, golf and there is a golf club which is 10 minutes away. So that makes it very easy.

Yeah. So that's ten minutes drive away. OK, so most of your activity would be on your property itself, or the golf at [town]. Yep. Now, thinking about this, I'll just go through a couple of areas – availability and accessibility of places to be active. And the comments here, they may be things that um, you may use yourself. Um, they may not be. But there are things that are within your area that you are aware of, OK? So if I ask you about places to be active in your area, what sorts of things would you tell me about?

Um, well Lake [x] [16:26?] is a place to be active I suppose. You can go skiing or lake boarding or swimming or whatever there. The golf club. [district] which is about an hour away I suppose by car, you can go skiing up there in the winter. And then I suppose you can go running or bike riding on the roads.

Yeah. And how far is the Lake from you?

Ah, two minutes.

Wow. OK. So that's very close. And all of these activities would be to get in your car and drive to?

Yeah, yeah.

Yep. OK. And the access to those places – are they convenient and easy to access in terms of cost or opening hours?

Ah, yes, yes.

Yep.

Very much so.

Does having places to be active influence whether you are active or not?

Yeah definitely.

Mmm. Like having access to the golf club is, does help, yep.

Yeah, well if the closest golf club was 40 minutes away, well I probably wouldn't go anywhere near as often as I do.

Yeah. Oh no that's a really good point. Mm. Thank you for that. And if you wanted to be more active, are there things or places that would help if they were available and more accessible?

Ah for me personally no.

Yep. Now next area is destinations. If I were to ask you about places that you could walk or cycle to from your home, what sorts of things would you tell me about?

To do?

Mm. Like you are actually in [town] itself, so…

Not much.

Yeah. Huh, not much. OK. OK.

Well, we walk to the park sometimes, the park that we did some fundraising for and built for the community. We take the kids to that sometimes. But apart from that – yeah, not a lot.

There's not a lot. Yes. So does having, or not having in this case, destinations influence whether you are active or not?

In that regard, yes.

So not having destinations does influence you?

Yeah, I'm very much a – my wife quite often asks, "let's go for a walk" and I say "where are we going to?"

Yeah.

I'm not a stroller. I don't mind walking as long as I've got somewhere to go to. But I'm not just going to go for a walk for the sake of it. I do enough of that during the day anyway.

Yeah. It's a really good point. The people we are obviously talking have also indicated that that does actually help – having a destination.

Yeah, yeah.

Or, it's a bit of multiple purpose. You don't just go for no reason. And if you wanted to be active, would it help if there were more destinations within easy walking or riding distance?

Yes.

Yep. Yep. OK. Now, going to talk about neighbourhood function and design. If I were to ask you about the built or the man-made features of your area, what sort of things

would you tell me about? And these are things like footpaths and the walkability. Those sort of things.

Oh look we've got to go across the bridge which is a bit hairy, cos there's no um, there's like a little space to the side which you can walk across, but there is no designated walking area on the bridge, which with a couple of young kids can be a bit daunting when there is log trucks circling past you.

Oh right. Yep.

As far as footpaths are concerned, I mean they're gravel footpaths – they're fine. Um, yeah, I mean throughout the township everything is fine. I know when I was a few years younger I used to go cycling after work. I put on a bit of weight, but I ended up stopping just for the simple fact that it was just too bloody hairy with the log trucks and everything, so, yeah.

Yes. And that's a comment that people have made, um not only in this study of men living in rural areas about the [region], but we also um talked to rural women earlier in the year and they were saying the same thing: that cycling particularly is a bit hairy because the roads are too narrow and there's trucks. OK. So do the built features in your area influence whether you are physically active or not?

Oh well I suppose they do, yeah.

And if you wanted to be more active are there any built features in your area that would help?

To be built? Or that are built?

Ah, that would be built. Yep, i.e. you don't have them now but it would be good to have.

Yeah, I s'pose just widening the roads a bit. You could do that for the 50 people that live round [town], of which probably one is going to use. I mean, yeah, the roads up round this way need widening for multiple reasons, not just exercise.

Ah yes, yes. I presume for safety, driving and – yep.

The log trucks need to be there because they bring a lot of advantages to the community, but the government just need to think of where the big traffic is and create

access for them, it would be better as well. Because they are only trying to make a living.

Yeah, no that's a really important point. There are obviously areas that are a bit trickier in terms of narrowness and bends and things like that. Yep. And there are sections that are OK. Yeah. OK. And that has got a lot to do with just the geography of the area.

Yeah, very much so.

OK. Now the other side of it is the natural environment. If I were to ask you about the aesthetic qualities or the attractiveness of your area, what sorts of things would you tell me about?

Well it's a pretty beautiful spot. There's a mountain range that you can look out at. At this time of year it's got snow on it and um, yeah, this time of year it's very beautiful. Everything is green and the hills look lovely. Um, but yeah, you get three years of drought and it doesn't look so beautiful. But yeah, it's a nice spot to live when the weather is like this.

Yeah, yeah. And um, do the aesthetics of your area influence whether you are physically active or not?

Well I suppose they do. Because if it's dry and horrible, then I work a lot more. Because more irrigation and that type of thing.

Hm. It's not so pleasant is it? Hmm. And then when you get that greenness and that lush country, mm, it's …

Yeah, it picks you up out of the doldrums very quickly when it's like this. Very nice.

Hm. And if you wanted to be more active are there features of your area that might help if they were more aesthetically pleasing or attractive?

No, not really.

No, because you live in a very beautiful area.

Yes.

Yeah. Now the last area is safety. And we have touched on it a bit. If I were to ask you about safety in your area, what sorts of things would you tell me about?

Yeah, well like we said before, like the roads that are a bit narrow and the bridge that sort of really hasn't got a walkway. But then again it's only our family and one other bloke that lives on this side of the river. So they're not going to spend $40,000 of walkway for us.

Yeah. Is that bridge the one on the main road through [town]?

Yep. It's good, I mean it's got a little elevated spot and a little bit of concrete there, but yeah, if there's two trucks passing on the bridge it doesn't leave them a hell of a lot of room.

No. So does road safety influence whether you are physically active or not?

Oh minimally. A small amount, yeah.

Yeah. Because most of your activity is either on the property or playing golf.

Yeah, that's it. Yep.

And does personal safety influence whether you are physically active or not?

Sorry what was that?

Personal safety.

No, not really.

Yeah, it's a safe community.

Yeah.

OK. And if you wanted to be more active, are there personal or road safety issues that if addressed might help you to be more active?

Oh I suppose the same issues again I suppose.

Yeah, the road safety ones, mm.

Yeah, pretty much.

Well that's it. But before we finish up, is there anything you would like to tell me about where you live, your um environment or your physical activity that we haven't covered today?

No, not really. I think we've gone over pretty much all of it.

That's great.

AUDIO RECORDING ENDS

Interviewer: OK. Right. So, I thought we might start by talking a bit about the place where you live, OK? And what it’s like to live there. So, if I, could you tell me a bit about, like, where it is that you do live, and what it’s like to live there? And the likes, dislikes?

Respondent: OK. So I live, [town] on a farming property.

Interviewer: Oh, right. OK.

Respondent: And for me, I absolutely love that. And I'm... but I've always lived on a farm. I've always lived in, on property. So, I don’t... yeah, I don’t have a problem with where I live at all.

Interviewer: Uh-huh. Uh-huh. And the, it’s a property, how big’s the property?

Respondent: 8,000 acres.

Interviewer: Wow. That’s big. (Laughing).

Respondent: (Laughing). So I've got lots of scope. (Laughing).

Interviewer: (Laughing). Wow. OK. And the length of time you’ve been living there?

Respondent: 26 years.

Interviewer: OK. And, you’ve mentioned a few reasons for living there, but, just your reasons for particularly living, maybe in [town]area itself?

Respondent: That wasn’t really by choice, necessarily. It was more of, that’s where my husband’s farming, family lived, and that’s where the farm was, and...

Interviewer: So you moved there?

Respondent: And so that’s where I am. (Laughing). And, yeah. And I don’t know whether you would, yes, I don’t know whether you can or choose, as a place to live, but it is where I live.

Interviewer: OK. OK. And your distance from your nearest town, which is presumably [town]?

Respondent: So I'm seven kilometres from [town].

Interviewer: Right. Which, which side?

Respondent: On the [city] side.

Interviewer: On the [city] side. OK.

Respondent: And we’re right on the highway. So our house is right on the highway, and the property’s split in half by the road.

Interviewer: Oh, really? That must be, probably, well, not easy...

Respondent: It has challenges.

Interviewer: ... it has challenges. Yes. Yeah. Do you run cattle, or?

Respondent: Sometimes, but mainly sheep.

Interviewer: Mainly sheep.

Respondent: Yep.

Interviewer: OK. Yes, because cattle seem to have to be, well, sheep do to, it has to be moved. (Laughing). OK.

Now, in research with women living in urban areas, which of course is where all, most of the research has been done, people often refer to their local neighbourhood. And I'm interested in understanding what the term ‘neighbourhood’ means to women who live in rural areas. OK? If it has relevance at all.

So what does the word ‘neighbourhood’ mean for you?

Respondent: It’s one I don’t suppose I would ever consider ‘neighbourhood’. Yes, I don’t think I would consider that I have a neighbourhood. I live in a community.

Interviewer: Ah. That’s the other question. What about the word ‘community’? What does it mean to you?

Respondent: Community is about where I live. And community isn’t just the nearest town, which is [town] community to me, because as a child, I grew up in [town].

Interviewer: Oh, right.

Respondent: And so I've lived in the [region] all my life. And so, there’s a lot of community for me. I, [town] would be a community that I like to go into for other reasons. So yes, I, and in my work, I work in, in all the communities.

Interviewer: OK. So, community is, is community about geography for you? Or...

Respondent: I think community’s about people.

Interviewer: OK.

Respondent: For me.

Interviewer: Yep. OK.

Respondent: More than, more than a place, or a...

Interviewer: Yep. So in that sense, it’s about the people. In that sense, does it have, if you needed to put a distance factor on it, what would it, what would it be? Would it be the whole of the [region], or would it be particular parts of it? Certainly more than...

Respondent: I, I think...

Interviewer: ... [town]?

Respondent: Yeah. I, I think you'd have to say... that my main community would extend... 45 minutes away from home. And...

Interviewer: Driving?

Respondent: ... around, yeah.

Interviewer: Yep.

Respondent: Yep.

Interviewer: Which would be roughly how many kilometres would that be? Don't know how far you can go in the [region]...

Respondent: No.

Interviewer: ... in 45 minutes. A fair way, can you?

Respondent: Yeah, 50, I suppose it'd be around 45, 50 kilometres.

Interviewer: OK.

Respondent: Yep.

Interviewer: OK. Now, I wanted to talk a bit about physical activity. And we've said that's a broad concept. So, what does the term 'physical activity' actually mean for you?

Respondent: Oh well, I... physical activity, I've always been very physically active. It's part, it's my absolute passion. So, for me, now, like, today, it would be walking and gardening and...

Interviewer: And it's very...

Respondent: ... bike riding.

Interviewer: ... a part of your work as well, so.

Respondent: And, and it's also taking exercise classes and doing Tai Chi and all those things. But, running. Yeah. So, I'm mentally am quite, well, yeah. I, I need to do physical activity to, to maintain me, my normality, I think. So, which is why I do it. But yeah, for me, it's just doing stuff.

Interviewer: Yep. Yep. Yep. And, do you consider yourself to be physically active?

Respondent: Yes, I do.

Interviewer: And why?

Respondent: Because I participate in some form of physical activity every day. Self moted, self-motivated physical activity every day.

Interviewer: Yes. Yes. And where does physical activity fit in to your life? Is it high priority? A priority? Of no interest?

Respondent: It’s a priority. I’d like to say it was a high priority. Yeah, well, I mean, I do it every day, so I suppose it’s just an everyday priority. So, but I don’t know whether I could say it’s high, because it’s just part of my life.

Interviewer: Yes, I understand that. Yes. Yes. OK. Absolutely. So, it’s important, it’s you know, an important part of your life, because it’s part of life. OK.

Now, in our research we often think about physical activity as any activity that lasts for at least ten minutes. You know, the evidence of health benefit, of course.

Respondent: Gee, I'm really active then. (Laughing).

Interviewer: (Laughing). And as you know, causes your body to work harder than normal, and your heart rate’s going up, and breathing a little heavier. And warming up. And so, thinking about this type of physical activity, during the last two weeks, just as a guide, now, in, so, I'll just ask questions about different parts of your life, just so we make sure we cover it all. It’s, it’s not just trying to segment your life into, into parts, so. In the past two weeks have you been active at all at work for at least ten minutes of the time?

Respondent: Yes.

Interviewer: Yes, and could you tell me about that?

Respondent: Well, I participate, I facilitate exercise classes, which is mainly [gentle exercise], so, that doesn't necessarily get your heart rate up, but it does get your heart rate up. But, and I do falls prevention gentle exercise classes as well. But I might be doing them, like today, I've done two lots of [gentle exercise], and yesterday I did, and a gentle exercise class And yesterday was the same. I did [gentle exercise] twice yesterday with two groups.

If I'm working up at the school, I always will walk to the school, which is, you know, a ten minute walk up and a ten minute walk back. So I, I take opportunity in every part of my day. And I'm quite often am running backwards and forwards across to the [workplace]. So I take those opportunities for incidental exercise in my work day.

Interviewer: Yes. Yes. And do you work in different communities here?

Respondent: Yep.

Interviewer: Whatever you want, like, prefer, areas, communities?

Respondent: Yep.

Interviewer: Well, how you would like to call them. You, you’ve obviously been working in [town] today. But you’ve mentioned [town]?

Respondent: So, I've been in [town] this week, and I'm, and then I'm quite often go to [town] as well.

Interviewer: OK.

Respondent: So I, I'm not, I mean, there's some awful days when I have to sit at the office, in the office. They're awful days. (Laughing).

Interviewer: OK. So, given those activities that you've talked about, in the last two weeks, OK? Quite a few. Could you give me an idea of the intensity of those activities? Whether they're steady, moderate or vigorous?

Respondent: Oh, for me, steady.

Interviewer: OK. Yes. Because of the nature of your work. You're... yep, yep. I understand. Because you're working with, maybe an older population?

Respondent: Yeah, really working with elderly and very unfit people. And physio clients that have got specific needs, so.

Interviewer: Yes. Yes. That they're trying to recover from. And duration? - how long would those activities have been... for?

Respondent: I would have think, well, Thursday mornings, it's generally an hour. …yesterday afternoon for [gentle exercise] as well, so that was another half an hour there. So, ranging from half an hour to an hour.

Interviewer: OK.

Respondent: But, it's teaching as well, so you've got that too though.

Interviewer: Yes. And how often would, during the week, would you have done that? In the last two weeks?

Respondent: Well, in the last two weeks, I've actually done it quite a bit. But yeah, so in the last two weeks, I probably would have done it every second day.

Interviewer: Right. OK.

Respondent: I seem to have been doing it quite frequently lately, which has been nice.

Interviewer: And it’s usually during the day?

Respondent: Yes.

Interviewer: OK. And this is what you usually do? And does it change depending on the season or time of year?

Respondent: It does change depending on the time of year.

Interviewer: OK. And in what way does it change?

Respondent: In the summer, if some of the groups aren’t going over the summer, because people go away or things like that. So, that would be one change. But other than that, it’s fairly steady, because we’ve generally, elderly people who are here. Or if I'm on holidays it changes.

Interviewer: Oh, yes, yes. And does the weather change or the coldness change it, for, you know, like for the walking?

Respondent: Well, it doesn’t change for me.

Interviewer: But does it change for them? I.E., so what you can do and what you can't do?

Respondent: Actually, that's really interesting you ask that, because I've got one Heart Foundation walking group that's been going for a year. I think they've missed one walk because of weather, which I think is fantastic.

Interviewer: That's amazing. That's great.

Respondent: They rug up and they just go.

Interviewer: Yep.

Respondent: And, and I think that comes down to, just educating, and motivating and keeping them going. And, and that's, so that they get to that point of loving it and needing to do it. (Laughing). Yep.

Interviewer: That's great. OK. And if you had wanted to, is there anything that would have helped you be more active at work? And it sounds to me like you're pretty active already. (Laughing).

Respondent: Yeah, no, I don't, yeah.

Interviewer: That's fine.

Respondent: I don't think it's very much, like, there are days and weeks when you're so committed to filling out reports or doing those things that, you really do have to make sure that you...

Interviewer: Get a balance of...

Respondent: ... get down on the floor and do some stretches. Or go outside and take those five minutes, ten minutes here and there. Otherwise, you know, I, physically, I would be a mess, sitting at the desk all day. I don’t know how people do it.

Interviewer: No, I have to get up and get around and do things. OK. Now, in the past two weeks, have you walked or cycled for at least ten minutes at a time, to get to or from places? Now, I don’t know if you cycle or walked?

Respondent: No, I, unfortunately I don’t cycle. I’d like to think I did, but I don’t. And walking, yes, I've walked, but not to get to places.

Interviewer: Uh-huh. OK.

Respondent: Oh, except up to the school, but that’s work. No, I haven’t been.

Interviewer: Yep. OK. That’s OK. Do you want to talk about walking to the school? I don’t know how far it is? It’s...

Respondent: Oh, it’s only, it’s a kilometre. (Laughing).

Interviewer: Oh, OK. OK. So do you want to count that, or, or not?

Respondent: No, not really, because that’s part of work, and that’s...

Interviewer: Alright.

Respondent: ... yeah, sort of covered that one.

Interviewer: OK. And is that usual for you, that you don’t tend to walk to or from places?

Respondent: It’s just that, I was just thinking about that last week. Like, last... if, if, if I'm, like, here it is usual for me not to walk to places, because of time.

Interviewer: Yep.

Respondent: That will be the sole reason. Just because for me to walk into [town], that’s seven kilometres. Well, I don't always have time to walk seven kilometres there and back. I might have time to walk one way but not both.

As much as I’d love to think I could walk to the shop and get my groceries, if I had a bike, I would definitely cycle. But the road limits me there enormously.

Interviewer: Because?

Respondent: Because it’s so scary.

Interviewer: The traffic?

Respondent: On a bike. And, and I, you know, I can go cross country, and I do go cross country, I just don’t have an operating bike at the moment. But the quicker route, if I'm just whizzing in to get the milk and the mail is, is on the road, and it’s, the last time it was petrifying. I remember I just

thought I can't do that again. So. And that's the first time I've really felt like that. But. So. Yep.

So, but you know, if I'm in town, I will always park my car in one place and walk the entire day. And so, if that means I'm parking in the city and need to go to [suburb], I will walk to [suburb] and walk back. And if I'm, yep, going down to [shopping centre], I'll walk from the city down to [shopping centre], rather than get my car and... so I do take those opportunities. But that's, yep.

Interviewer: OK. That's fine. And does the, does it change, depending on the season or time of year?

Respondent: Going from one place to the other, walking? Or just...

Interviewer: That's right. That particular question, yes. I'll, I'll look at different parts of your life, it's OK. Yep. Yep.

Respondent: No, if I'm going to do it, I'll do it anyway.

Interviewer: OK. That's fine. Now, in the past two weeks, have you been active around the house or yard? So that's at home, inside or outside, for at least ten minutes of the day, time?

Respondent: Yes.

Interviewer: OK. Can you tell me what, about what you did?

Respondent: As in, I've been gardening. Cleaning out the sheds, and housework and, I do, I stretch everyday for at least 20 minutes. What else have I done in my last couple of weeks? Yeah, I think that would be gardening and housework.

Interviewer: Yes.

Respondent: Cleaning out sheds.

Interviewer: OK. So thinking about those activities, could you tell me about the intensity of them? Are they steady or moderate or vigorous?

Respondent: Oh no, they'd just be steady, moderate. Yeah.

Interviewer: OK. And the duration? How long might you do these activities for?

Respondent: That's a hard one to answer. Like, if it was a Saturday, I wouldn't sit down. If it was a Sunday, I wouldn't sit down if I was at home. If I have a day at home, I, I don't stop.

Interviewer: Yes. So it's for several hours that you do things?

Respondent: Yeah.

Interviewer: And they become a continuous, you know...

Respondent: Absolutely.

Interviewer: ... you go from one activity to the next. Yes, I know what you mean (laughing).

Respondent: Yeah, so, where at work, I would spend a lot more time sitting down.

Interviewer: OK.

Respondent: So...

Interviewer: So at home, you know, it would be activities that'll last for at least a couple of hours, and you keep on going during the day?

Respondent: Yeah.

Interviewer: OK. And the frequency. How often... would you do...?

Respondent: In the last two weeks? Or...

Interviewer: Yes. Yes.

Respondent: Well, I worked seven days a week, a fortnight.

Interviewer: Yep. Yep.

Respondent: I would spend... one day a week in [city], because I also have children in [city]. A child in [city]. And... so, the other days, I'm fairly frantic at home. Which isn't that many days, really. When you look at...

Interviewer: It's a couple of days. Yes. Yes. So that would be, you know, every...

Respondent: So, maybe...

Interviewer: ... a couple of times a week? Yeah.

Respondent: Yeah.

Interviewer: OK. And, when, what time of the day did you do these activities? During the day, or in the evening?

Respondent: Both.

Interviewer: Both. OK. OK. Any reason why you did, did the activities at a particular time of the day?

Respondent: Because that was like, when, when it was outside activities, that was the time that suited, and so it was planning around what I could do when, I suppose. Yes.

Interviewer: And who did you do the activities with? Did you do them, yep.

Respondent: Family. Family and by myself.

Interviewer: Yep.

Respondent: So.

Interviewer: And is that what you usually do, or is one off?

Respondent: Yes.

Interviewer: Yep. Yep. And does it change, depending on the season or the time of the year?

Respondent: The gardening does.

Interviewer: Yes.

Respondent: (Laughing).

Interviewer: In what way does it?

Respondent: Well, in winter I probably don't get as physically active in the garden. Just because there's not that much to do and the ground's generally frosted. So (laughing).

Interviewer: It's not exactly a growing time.

Respondent: Yeah. So, I suppose, I don't do a lot then, in the garden. But I would do other things. Like, we've gone out and get wood, or, you know, so it's all very...

Interviewer: There's always something to do on properties, isn't it?

Respondent: ... similar, yeah.

Interviewer: When you live on land, there's always something to do. OK. If you wanted to, is there anything that would have helped you be more active around

that, you know, home, inside or outside? Sounds like you're pretty active already.

Respondent: Yeah, no. I don't.

Interviewer: OK. That's fine.

Respondent: Once again, I think that comes back to your own self-motivation. You can choose to sit and do nothing, or you can choose to do stuff.

Interviewer: Now, the last part of this one is, have you been physically active for at least ten minutes during your leisure, or spare time? OK. And if so, can you tell me about what you did?

Respondent: Yes. No, I've, I've walked most days, for half an hour or more, at moderate to high intensity. So, and that's something I try and do at least every second day, if not every day, if I possibly can.

Interviewer: So when in the day might you do that?

Respondent: Well, my working days, I race home and do it...

Interviewer: After work?

Respondent: ... just before the sun goes down.

Interviewer: Yep.

Respondent: (Laughing). And, and then when I'm at home, that’s when I tend to do more lengthy walks, and, and do it...

Interviewer: During...

Respondent: ... in, in the morning. Yeah. I’d like to do it, much prefer to do it in the morning.

Interviewer: And where? Where did you do your walking?

Respondent: Well, that’s where I'm very fortunate. I have...

Interviewer: You have all these paddocks.

Respondent: ... lots of paddocks.

Interviewer: So you would generally do...

Respondent: Oh yeah, I, I walk at home. More often than not. And sometimes I might meet a girlfriend and we’ll go for a walk at her place, or, but it’s all on farmland. More that, yeah, tend to... yep.

Interviewer: And is that what you usually do?

Respondent: Uh-huh.

Interviewer: And does this change, depending on the season or the time of year?

Respondent: Yes. It does.

Interviewer: Yes.

Respondent: Simply, in the spring, it's a nightmare because of the grass seeds. So I have to choose very carefully, and we might go onto the more gravel tracks and the, the lanes and things. More then.

Interviewer: And they're quiet enough for you to walk on?

Respondent: Yeah. And the roads. And obviously in the winter, if it gets really wet, you might hit the...

Interviewer: It's not much fun.

Respondent: ... hit the quieter roads a bit more.

Interviewer: Yes. Yes. Boggy paddocks.

Respondent: But generally, it never rains up here that much, so. (Laughing). So generally it's not, not a huge problem. It's more, more the grass seeds and the snakes in the spring. Yeah.

Interviewer: And if you'd wanted to, is there anything that would have helped you be more active in your leisure or spare time?

Respondent: More time. (Laughing).

Interviewer: Yes. OK. Now, we'll talk now about physical environments. OK? So we've talked a bit about where you live and physical activities. And now, the

physical environment’s about where you live and work and play. And so when I talk about physical environment, I mean all the physical things that surround you. Roads, trees, parks. Streets, lighting, whatever. Because, some of these things people in urban areas have said, might influence their physical activity. So, obviously like to ask you if that’s relevant to you too.

Now, first of all, can you tell me a bit about how easy or difficult it is to be physically active in your, your area? We’ve talked a little bit about that.

Respondent: Yeah. I think we’ve talked a little bit about that one.

Interviewer: Because you’ve got, you mainly talk, walk on your property.

Respondent: Yeah.

Interviewer: Yes. And it’s a bit far to, to then walk into [town], and it’s on a main road.

Respondent: Yeah. I can walk cross country to [town]. So, what was that question again?

Interviewer: It says “Can you tell me a bit about how easy or difficult it is to be physically active in your neighbourhood?”

Respondent: Look, I haven’t really experienced, I've, set myself a goal to run a marathon. That was a few years back. Five, five or six years ago. And it was just one of those life-long wishes that I was going to do. And so I set out to do it. And, and I had to do a lot of that on the road, because the actual race was going to be a road race. So, and not over paddocks.

And people used to think I was mad, running up and down the road. And I ran for hours and I ran for kilometres. But I wore fluorescent tops, and...

Interviewer: Did you run along the main roads?

Respondent: Yep. And I didn’t wear, I didn’t wear an I-Pod or anything. You know, didn’t have the music things in my ears...

Interviewer: Yep, yep, yep.

Respondent: ... so that I was very aware of what was around me.

Interviewer: Yes, because, with them, you can't, you’ve got your senses are impaired that way.

Respondent: And, and I think, you know, I, not once did I feel unsafe running on the road.

Interviewer: And it was actually on the tarmac, the bitumen?

Respondent: Yep. And...

Interviewer: And you’d just be able to get off if there were traffic?

Respondent: I would generally just follow the white line, so I was to the side. The log trucks, I'm sure they started talking to each other and saying “She’s on the road again.” Because they’d actually go around me.

Interviewer: Oh, so you didn’t have to get off?

Respondent: Yeah. So, so they were quite good. I mean, obviously I knew when it was safe they went around. And, and I just got used to stopping if...

Interviewer: Oh, you’d stop and get off the road?

Respondent: ... if one car was coming one way and a truck was coming, I would actually stop so I wouldn’t get sucked into the... but, and so I never actually felt unsafe doing that. But on a bike, I feel very unsafe, because there is nowhere to go.

Interviewer: Yeah, you can't get off.

Respondent: Yes, you can get off but...

Interviewer: Yes. But not easily.

Respondent: And so, generally you’d have to ride a mountain bike, which on roads is not that easy. And, and the caravans that are now on our roads are actually worse than the log trucks. And so, that, that was my last experience, was the caravans. Just one after the other after the other, and it was a windy day. And with their wind tunnels plus the wind, I was actually finding it really hard to stay, to not be thrown in to the caravans. So, to physically stay on the road.

So... but... you see, I, it is difficult. It would be really lovely to have that slightly wider road, so you could encourage a lot more people to walk.

And, because a lot of people were really concerned for my safety, doing that. I felt quite safe, but it was people’s concern for my safety.

I have to say, when I, unfortunately was then diagnosed with a form of arthritis. And so I went through a period of very, very, extreme pain. And so, walking over paddocks was nigh on possible, because of, but, and it was really hard then, to find a nice walk that you could do, locally that wasn’t too, that just, yeah. But, but because it’s something I love to do and I want to do, like, it’s not difficult.

Interviewer: Yep. OK.

Respondent: But, but I would...

Interviewer: But you, like, for you, you’ve, you’ve resolved a way that you can access and be physically active, but you’re also aware that there are some difficulties?

Respondent: Yeah. And for, I'm self-motivated too. And I know with the walking groups that I've run, that’s, that’s been really interesting and particularly at [town]. That, that group has been a fantastic group. And it’s all hills at [town]. There is nothing but hills at [town]. And they’re quite, so when this group arrived, they were all 50 and over. And they, they walk, were all reasonably unfit, really.

But, I think it was about... the third walk, a lady came along to join the group, having been coerced by one of the group that had done the first three walks as something she should do. And she came and she was, she’s morbidly obese, five different chronic conditions, and the set walk

for that day was to drive about five kilometres up a hill and then walk the last two. That's what they decided they were going to do. And I thought this lady was going to die. I, and she was so determined that she was going to do this walk. And yet, from a health point of view, there's just no way that she should have been doing it. But no, she was insisting.

Anyway, we duly, she made it. And she was so pleased with herself that she'd made it. And she has maintained walking ever since. And now you go with that group, and they start at the beginning of the five kilometre drive and walk the seven up...

Interviewer: That's impressive.

Respondent: ... up the hill. And, and so I think, for me this, I didn't know how the Heart Foundation walk was going, I always thought it was a really good idea, which is why I took on being the coordinator, because I thought, well, it's something. But when I see that group, I think it's fantastic, and it's a model that actually does allow people to self motivate and motivate each other. And it's got that really good social interaction.

And, where I walk for solitude. I walk for all those other reasons. But for some people, it, it's that very social, and that's what I see in the Heart Foundation walking groups, is that social aspect that keeps you coming back. Keeps you motivated. And, and yeah, with this group achieve, they go bush walking now. You know, they don't just walk on the roads and things. They actually go bush walking.

Interviewer: Oh, good. And that's what, that's why I'm saying, I wanted to talk to both, both you and [colleague] because you've got that overview. Because the,

when I'm going to be asking you about specific features about the physical environment, there's not only your own, it's your own experience, and that means for you, but what you know from, from others as well. So that'd be really useful.

Now. OK. So the first one's availability and accessibility of places to be active. If I were to ask you about places to be active in your area, and you can, as I said, there've been, maybe some examples you want to give around the highlands that you know. What sorts of things would you tell me about? So first of all start with your own area.

So what, if I was to ask you about places to be active, what, what sorts of things would you tell me about?

Respondent: Places to be active in [town]?

Interviewer: Uh-huh. Like, recreational facilities, walking tracks. Parks and things.

Respondent: There's no specific walking tracks. But walking in [town] is quite nice. And there is what they call the [x] walk, which I did with the Heart Foundation Group.

Interviewer: Oh yeah.

Respondent: I had my Heart Foundation walking group there the other day, and I, we actually walked along there.

Interviewer: The [x] walk is what? Down to the river is it?

Respondent: Or the [x] walk? And it’s got, it’s been sort of maintained. It’s got all the potential in the world. But it needs some work. And so, whilst this group, they’re quite, they’ve been going for a little while, I felt that they were fine, but it’s not a, not something that I’d advise a lot of people to do, just because there’s...

Interviewer: So there’s a couple of walks in that area?

Respondent: So there’s that. There’s tennis court in [town]. There’s a recreation ground in [town] where you can play cricket or whatever.

There’s, there’s some quite good side roads in [town] that you can walk out on, gravel roads, which aren’t private.

Interviewer: That aren’t busy?

Respondent: And they’re definitely not busy. They’re basically farm roads, but they’re not private. So you can access those. But it’s hilly again. The terrain’s hilly. But, and then there’s a hall that you can hire out if you wanted to, you know, run some specific exercise.

Interviewer: And so, with access, you’re saying ‘yes’ that there is accessibility. Are there any, are they easy to access? Like, are they, are there...

Respondent: Well, they’re not well identified.

Interviewer: ... any facilities that... OK. Require cost or opening hours or anything like that?

Respondent: They, they're not, like, the walk and the tennis courts and all those things aren't easily identified.

Interviewer: OK. So you'd have to know about them?

Respondent: Yeah, I think you would. Yeah. Or you'd have to be wandering around [town] and say "Oh, we've got a tennis court." And there's, you know, there's a really nice kids playground there as well.

Interviewer: Oh yeah.

Respondent: Yep. And, and a park. Which is a nice, and that's all quite new. That's quite a good facility. But for the hall, if you wanted to use the hall for physical activity, then you would have to pay hall hire and get the key from the council and organise that).

Interviewer: And does having places to be active influence whether you're active or not?

Respondent: No.

Interviewer: And if you wanted to be more active, are there things or places that would help, if they were available or more accessible?

Respondent: Well, like, if was a gym person, there's a gym up at the [town] school, that's accessible. If that's what motivated me. And then half an hour down the road's [town] for me.

Interviewer: Oh yes. Yes.

Respondent: So there's, there's an indoor pool. There's aerobics classes and punch-fits and Zumba if I wanted to go and do that. There's quite good walking tracks [town] as well, if that was, and that is half an hour away. A lot of people travel half an hour to do exercise.

Interviewer: Yes. Yes. Yes. Yes, you know, that thing of, you, you most often get in your car to, to get somewhere. Yeah.

Respondent: And I have ridden my bike to [town], so. (Laughing). It sounds, not that far.

Interviewer: That's impressive.

Respondent: (Laughing).

Interviewer: I'm impressed. (Laughing).

Respondent: I've ridden, I've ridden it to work too.

Interviewer: Destinations. If I were to ask you about places that you could walk or cycle from, to from your home, what sorts of things would you tell me about? Things like shops or schools or services.

Respondent: Like, riding to [town] on my bike?

Interviewer: Yes. Those sorts of things.

Respondent: Riding to work on my bike to [town].

Interviewer: Yes. Yes, that’s right. Friends. You know.

Respondent: I would, I've got a girlfriend and we have occasionally gotten our bikes and ridden a few of the dirt, the, the, the little country roads around [town] and around, so we’ve done that.

Interviewer: So there are a range of rides.

Respondent: Yeah. I haven’t explored the [x] Trail much here. I’d like to see what, what that involves. I think it’s really, and that, that is something that runs throughout our community, the [x] Trail.

Interviewer: [colleague] was telling me about that. It seems very interesting.

Respondent: And it’s, the government has really let that go, I think, a little bit.

Interviewer: That’s her point too. That it needs the, the upkeep of it needs to be looked at.

Respondent: And so I think that’s really unfortunate, because I think that there’s maybe some enormous potential there, to, to engage the local community in sections of it. And encourage them to use sections of it, not necessarily...

Interviewer: And again, it’s sort of like being able to access. Like, is, is it easy to access, and, I understand it’s through private property as well.

Respondent: Yeah.

Interviewer: And that needs to be really clearly signed and, and people need to be encouraged because that's quite...

Respondent: As I say, I haven't explored it much to actually work out. And you see, we do have a National Park on our back doorstep as well. And I think, you know, that something that as a family we would go up there and have a picnic, or when it's snowing, we'd go up and play on the, in the snow.

Interviewer: Yep. So how, how long would it take you to...

Respondent: And that's only half an hour, 45 minutes away.

Interviewer: Yep. Yep. OK.

Respondent: And then there's [x] Lake. And we're fortunate enough that our property actually runs down onto [x] Lake.

Interviewer: Oh, OK.

Respondent: But as a recreation area, that, that's huge. And, and I think a lot of community people would use that for fishing and for putting boats on if, water skiers or whatever. And yeah, no, we, we, and I would frequently walk from my property to, seven k's down to the lake. And there's kayaking and canoes and, yeah, we'd spend quite a bit of time there. But, that's very seasonal, obviously.

Interviewer: So, does having destinations influence whether you're active or not? Having talked about them?

Respondent: Well, no. Not really, because I have my property.

Interviewer: Yep. OK.

Respondent: There's destinations on my property, which encourage me. (Laughing).

Interviewer: If you wanted to be more active, would it help to have more destinations within easy walking or riding distance?

Respondent: No.

Interviewer: OK. Now, let's look at neighbourhood design, which is the sort of non-natural features. The built or the man-made features. And if I was to ask you about those non-natural features, or built features of your neighbourhood area, of your area, what sorts of things might you tell me about? That's when we're getting to roads and, and footpaths and things like that.

Respondent: Well, yeah. Rightly or wrongly, I have always think, well, we choose to live in the country, and, and we walk over rough paddocks and country lanes and country roads. And, and then, they're not ideal of you've got someone who is challenged for whatever reason. But...

Interviewer: Yes. Yes.

Respondent: ... but then if you're able and, it's really, I don't think a problem. And it's like people always walking on treadmills. Doesn't actually necessarily

improve their, their fitness from a holistic point of view. It might get their aerobic fitness up, but it's not a natural...

Interviewer: And this is where I'd be interested in asking you about your experience with the walking groups, because you are walking with people who've got a range of different ability, and, and stamina. And, like, how, how easy, how have, in terms of the question around the, the built or, features of the environment, and we're not only talking about footpaths, but in most communities, there's often there's a main road that just goes straight through. And when you try to walk, it's about not only footpaths, but whether the streets connect anywhere.

Respondent: Yes. So, a lot of it's going out and coming back.

Interviewer: It's quality, yeah.

Respondent: And that, and that, that sometimes doesn't motivate. It, I, you know, I don't, I like to do circuits.

Interviewer: Do you have to walk on the main roads for instance, in those communities?

Respondent: Well, no. Yes, so you'd be...

Interviewer: Yes, that connectivity, there's, walking through a main road.

Respondent: So, for a lot of people, like in [town], say, the walking group there, we've got someone with a walking frame, and we've got people pushing prams, and.

Interviewer: Because it's often not a continuous footpath, there's just a shoulder.

Respondent: So that's not ideal.

Interviewer: No.

Respondent: Pram walking is not easy.

Interviewer: No.

Respondent: So there's a whole group of people that it's very hard to encourage. So, here in [town], you could take a group out along the [x] Road, but you still could be confronted with log trucks.

Interviewer: Yes.

Respondent: And other trucks. And, and there's been too many roads...

Interviewer: And you take a walk along and then come back?

Respondent: Uh-huh. Yes. So. And also, one, one thing that I hadn't really thought about until I had a diabetic on one of the walking groups, and he actually had to stop because, a lot of it was on gravel roads, and it hurt his feet too much. And his, he didn't have good feet anyway because of his diabetes, and he just found it too hard. And, and then you've got the

limitation of not being able to afford the right sort of shoes that are going to help. And, so they, they are issues that do prevent people walking, who have got some form of chronic condition, maybe, or whatever.

So there're limitations there. But, and, and I can see, I mean, for me personally, it's not an issue, but I can see that, the not-build safe, walking environment does limit people's movement. And particularly with the elderly. And, and our population is getting elderly. And, and they're getting lazier and less motivated to, to move out of their houses and walk. But, when they're on their walking frames and things, no, it's not a good environment that encourages. The, the built environment isn't encouraging to them, to walk people to the shop to get the milk if they live in the town, or.

Interviewer: OK. Thank you for that. Now, OK. Do you think the build features of your area influence whether you are physically active or not?

Respondent: No.

Interviewer: No. Not personally, because of your context of how you're active. And if you wanted to be more active, are there any built features in your area that would help? Now, you were saying the road being a bit wider for cycles, but.

Respondent: I, I always wonder why they don't just, and I know it's money, but, (laughing).

Interviewer: Yes, all the extra little bit of bitumen.

Respondent: But just that, you know, that little bit wider each side. And I think that urban areas get to have their bicycle tracks and their walking tracks and everything. Why can't rural areas just have this little extension to the sides of their roads to ensure that it's safe for people to, to walk?

And, and you know, there's a man in [town]who, everyday walks his greyhounds. And sometimes I think that must be so challenging for him. Because he walks up the hill and he goes for a decent walk.

Interviewer: He's constantly on... yes, anxiety alert on the, on the main road.

Respondent: But, but yeah, it wouldn't be easy doing what he does. But.

Interviewer: Now, aesthetics, the other side of it, which is the natural environment. If I were to ask you about the aesthetic qualities, or the attractiveness of your neighbourhood area, what sorts of things would you tell me about?

Respondent: Well, I, I mean, I don't think... I've spent quite a bit of time recently in, in you know, Victoria and around the [x] area, because my daughter's there. And I, and you go there and it is, it doesn't matter which small town you go into, or where you go, there's lovely open, interesting parks, which have paths and things that kids can climb on. And...

Interviewer: It's a nice, natural environment. Trees and you know, landscape.

Respondent: Yeah. And the creek is a natural part, it hasn't been fenced off, and I do find here a lot of our parks and, they're just, sitting in a paddock, type park, and they're very fenced. Which, I know for legal reasons I think they

have to do that for children. But gosh, it looks so closed in and limiting. I find that very off-putting.

But I do not understand why in Tasmania, we cannot have, and replicate the same beautiful, flowing parklands and, areas and seating for people to sit on as, as they're active.

Interviewer: And have walkways and, and things that sort of connect with...

Respondent: Yeah.

Interviewer: Well, the example I got given was the little park opposite here. You can walk in it, but you can't get to the river.

Respondent: Yeah, I mean.

Interviewer: There's no access path.

Respondent: No. That's, that's like, we have this fantastic golf course here, and, which I know is a golf course, but it could actually have a beautiful walking track around it that could be used, and, and it's...

Interviewer: That's actually happened in areas.

Respondent: Yeah, and, and that to me would just be...

Interviewer: It's not a problem.

Respondent: ... and it's, you know, I just went to a meeting this week, actually with the, the [x] Golf people to see if we could run our fun-run around the golf course this year. And they were really for that. So, out of small things, big things might grow.

Interviewer: Yes. Yes. Yes. A beginning.

Respondent: And it's like, even the health centre, you can now walk down, across the river, and to the park. There's no seating anywhere in that. And there's no toilets. And, I, I do think that's really limiting when our general population's quite elderly in [town], and so, that, whilst it's nice, it's only half way there. And then you get to the end of the path, and you've either got to cross over a main highway, it doesn't extend up to a, to a more open, safer area to cross, or. And so, yeah, it's not connected. It's not, it just falls short of that connection.

And, and it's very much the same in [town], really. The backstreet. You can walk along the back road, and look, it's not a busy road, and it's really quite safe, and it's, it's a nice road as a foot path, really. But it is a road. (Laughing).

Yeah, but, but tourists seem to happily wander around [town]. You'll quite often see them wandering around [town]. But you've got the main road going through the town as well. So.

Interviewer: OK. And do you think your local environment is aesthetically pleasing and attractive, near your home?

Respondent: Actually, my, my paddocks.

Interviewer: Yes, your paddocks.

Respondent: Oh, look, I do. I mean, I love where I live. But no, we could improve it, but that's up to us to improve it.

Interviewer: And do you think the aesthetics...

Respondent: I have put some lovely bush around there though.

Interviewer: Yeah. Oh, that's lovely. Do you, do the aesthetics of your area influence whether you're physically active or not? And it's an interesting question for people who live in rural, beautiful areas. Because it's sort of, well, part of living there. But...

Respondent: I, I mean I, it's very hilly. And so, you know, I, you know, I have certain walks that I love and, and I love doing, at certain times of the year, because the view, and I never tire of what I have.

Interviewer: So, it does have an influence for you?

Respondent: Yeah.

Interviewer: It calls you out to go and visit.

Respondent: Yeah.

Interviewer: OK. There's just one, and if you wanted to be more active, are there features of your area that might help, if they were more aesthetically

pleasing or attractive? I think you were saying you were very active as it is.

Respondent: Yeah, and I think if I wanted to go for a bush walk, at least I've, oh, I've only had to go as far as [x] to achieve that, or.

Interviewer: Yep. Yep.

Respondent: But we do have some lovely spots around here. I mean, Lake [x] not very far away.

Interviewer: Uh-huh.

Respondent: And we’ve got some beautiful areas to walk.

Interviewer: Yes. Yes. One last area is just on safety. If I was to ask you about safety in your, in your area, what sorts of things would you tell me about? Now, we’ve talked a bit about traffic. But...

Respondent: Well, on the main roads...

Interviewer: Yeah, main roads.

Respondent: ... it’s the caravans.

Interviewer: Yes.

Respondent: And the log trucks obviously. And, and because there is so much main roads, it's very limiting to be able to walk after dark. And I'm not a, adverse to walking after dark, but it would not be safe.

Interviewer: Yep. Yep. From a traffic point of view. Does personal safety influence whether you're physically active or not?

Respondent: It's funny you should say that. I, I went for a walk the other day. You know, I do falls prevention exercise programs all the time, and I, I fell over a fence. (Laughing). I thought...

Interviewer: So easy. It is very simple.

Respondent: ... "No one's at home. It's a good thing I'm OK." But, so, every now and then I think, oh, and like, when there's snakes, you know, and I think, oh. And so I do tend to now carry a mobile phone with me, or leave a note on the kitchen table to say this is where I've gone, if no one's, if I hadn't seen anyone to say I'm going for a walk. And, I mean, I'll more often than not have my dog with me as well, so.

Interviewer: Yep.

Respondent: But, safety for...

Interviewer: But other than that, it's a safe community in that sense?

Respondent: Yeah. I, I mean, I've never heard of people being harassed because they've been walking on the roads, or anything like that. But yep.

Interviewer: And does road safety influence whether you're physically active or not? You've already talked about it.

Respondent: Yeah, well, I mean...

Interviewer: It has had an in...

Respondent: ... I know when I'm talking with groups, and encouraging people to be active, I do encourage them to wear bright colours, obviously. Take a phone with them. And, and I try and do that myself. But when the, half of the reason for you walking is to be away from it all.

Interviewer: I know. (Laughing).

Respondent: (Laughing).

Interviewer: That's the, even living near a, a National Park, and, and you're walking around and it a very popular one, and accessible for people, that walking and mobile phones, yabbering along and you go, "Excuse me." And you're (laughing). What are you here for? Anyway.

So, if you wanted to be more active, are there personal or road safety issues that, if they were addressed might help you to be more active?

Respondent: Well, the only one would be the cycling. I would love to think I could cycle more. But I, I really don't feel safe. In fact...

Interviewer: And for the larger community, you know there's obviously some road safety issues.

Respondent: And because, and it’s now becoming sort of a thing where, because of my arthritis, it is something that really I should be doing more of, because it’s going to limit that impact on my body. And yet, it’s not something that I feel I can safely do.

Interviewer: OK. Now, before we finish, is there anything else that you’d like to tell me about today? About where you’re living, or your physical activity, or the features of your environment that we haven’t covered today?

Respondent: I don’t know. Probably lots. I'm just trying to think of some examples... in the area. And I, I don’t know how this compares to urban areas, and I can't say I've done a lot of research or questioning about it, but, because I'm working one day a week now as ….I'm seeing a lot of what’s coming through, and, and what’s limiting people in their daily, even just their daily living activities. And, and it’s that, it’s that wear and tear of, and hard living. And when I say ‘hard living’, it’s, it’s women living on their own, cutting their own wood. And dealing with all of that. And it’s men who just have pushed and pushed and pushed themselves physically, working by themselves, lifting things that they shouldn’t be lifting. Which is, at very young ages, limiting them in, to being not overly active, because it hurts too much. And more often than not, it’s beyond repair. The damage.

And... and I don’t know how, you know, I don’t know if that’s really anything to do with this, but it is something that limits physical activity enormously, and I don’t know whether that’s the same in urban areas as much as...

Interviewer: Well, I think that's important. I think it's the context of health and our wellbeing, in a broader sense, and so context, physical activity is part, part of our health and wellbeing. But yes, I think it's really quite concerning. I mean, I mean, that's me coming from a more broader public health perspective.

Respondent: Yeah. And because there's not those built facilities for people to...

Interviewer: Have some options...

Respondent: ... to have some options.

Interviewer: ... choices of what suits them at what point suits them.

Respondent: Yeah. And, and whether, would they use them anyway? And that's, you can see, you know, there's that real juggle of, do you invest? But I think, I think in the long run, that investment wins out for a community, whether it be that tourists are going to be coming in to it more and using those built facilities, and therefore that, alone would encourage local people to join in.

Interviewer: Well, if they're going to be into an economic argument, it's a lot cheaper than emergency departments.

Respondent: Yeah.

Interviewer: And one of the women was...

Respondent: But they still...

Interviewer: ... actually saying about that whole thing of, you know, reaching a point in her life of, of, it's almost like, and I don't do it justice, because I'm not using her words, but it's that stuff about having to find your, you know, like, some, we all have times in life where we learn about having to take care of our bodies or our broader health and wellbeing. Like, more preventative stuff.

She was saying, there just isn't the support or the information. You've got to find it yourself. You've got to find your own path in all of this. And it would be really helpful if there was more understanding about the, to me, it's about the complexities of, of our life, and our bodies. And...

Respondent: And how it works, and why they work and...

Interviewer: Yeah.

Respondent: Yeah. And, and I mean, I think we're failing. We're failing in our schools.

Interviewer: That's what she was saying too. It needs to happen a lot earlier.

Respondent: Oh, yeah. And, and I think the worst thing that could have ever happened was, that school nurses were taken out of schools. Because, you know, I mean, they absorb it, and they're intelligent and, but, because it's not happening generation after generation after generation, you've got this big gap now. And, and how do you break that? You know, like, today, I can go and talk to the children about salt and wrapper foods, and nothing's going to change. Because that's what mum and dad do. And how, how does this little voice say "But..."

Interviewer: But you've sown a seed.

Respondent: Yeah, I've sown a seed.

Interviewer: The information's there.

Respondent: Yeah.

Interviewer: But yes, that, I mean, that's what I'm saying. Yeah. That's interesting. OK.

Interviewer: OK. So I thought I'd start a bit by talking about the place in which you live…

Respondent: Yes.

Interviewer: … and can you tell me a bit about what it's like living around where you do…

Respondent: Uh-huh.

Interviewer: … and your likes and dislikes? Now you're living in…

Respondent: In, well, [town], out of [town], about half way between [town] and [town], and we live on property and it's a sheep farm, and we help out on the sheep farm, my partner more so than I as we have two young children at the moment. We're very blessed to live in the location that we do, by the lake, which is beautiful, on Lake [x].

Interviewer: I wondered if it was Lake [x].

Respondent: Yeah.

Interviewer: When I've talked with women they actually mentioned that as one of the features of environment…

Respondent: Yes.

Interviewer: … yes.

Respondent: So we're actually, yeah, we're very lucky to be able to live there, and we obviously in the warmer weather get to swim in the lake and that kind of thing. Excuse me.

(Interruption – baby).

And we, what else? So we live about ten minutes out of [town], and…

Interviewer: Yes, so you're closer to [town] than [town]?

Respondent: Well, we're about… we're about in the middle actually. So we'll go to [town] to use the park there, 'cause they don't have one in [town]. But we'll go to the shop in [town].

Interviewer: So what would that make it in terms of kilometres?

Respondent: So we're actually about eight k's from [town] and about ten k's from [town] so… he's got a nappy I think, but that's all right. We'll deal with

that. So obviously we're really too far to walk to the local shop to get the paper and things, so it's a drive in the car for us, to the local shop.

Interviewer: Yes. Absolutely.

Respondent: And…

(Side conversation – baby).

Interviewer: Do you want to take a break?

Respondent: I might just change his nappy.

(Part b)

Interviewer: OK. Now just, you were saying that you lived on a sheep property.

Respondent: Yes.

Interviewer: What sort of size is it?

Respondent: We've got about two and a half thousand hectares where we are, yeah.

Interviewer: And the length of time you've been living there?

Respondent: We've been living there for a couple of years now.

Interviewer: Any reasons for living there?

Respondent: To take an interest in the family business and to keep it going.

Interviewer: So that was the family farm was it?

Respondent: That's my partner's family farm, yeah.

Interviewer: Yeah. OK. Now in research with women in urban areas are saying most of it has been done in urban areas, they often refer to their local neighbourhood and I'm interested in understanding whether that's relevant for women who live in rural areas. So if I asked you about the word neighbourhood, what would it mean to you? Would it be relevant?

Respondent: I think in a rural setting, having lived in both, that neighbourhood hasn't got as strong sense as community.

Interviewer: Ah, now that was the other question…

Respondent: Yeah.

Interviewer: … I was going to ask you. So neighbourhood, no, but community?

Respondent: Yes.

Interviewer: So what would community mean for you?

Respondent: I guess, well, community I think, well, with people with children I guess it's also a different, you know, differentiating between the community sense that would be if you didn't have children, but there were quite good facilities, playgroup and things like that that you can get along to. It

is, I guess the farming community, there are some people that they might live across the road from you but you don't say boo to them. There are a lot of different groups that come down to family and extended family that might be in the area, and they would obviously have a very strong sense of community within themselves. And I guess sometimes often I guess that you would kind of get a sense of a bit of clickiness and sort of different groups and things, but when you get things like the school and the church and places like that, and playgroup and the local supermarket, they're the crossroads where these different groups come across one another's path, or meetings for irrigators and things like that.

Interviewer: Yeah, things that you're involved in, yes.

Respondent: Yeah. So that's where I guess you get quite a strong sense of community when decisions needs to be made about legislation that the government may be wanting to change and the loss of hospital and [laughs] things like that. So I guess…

Interviewer: Yeah. So that's another level of community isn't it?

Respondent: Yeah, yeah, definitely.

Interviewer: And so if you needed to put a distance around community, what sort of distance? Would it be the whole of the [region] or particular geographic areas?

Respondent: No, I think really, I mean because of the distance between different townships, I think you may know people in the next town, but I think on day to day living when you've going about your business, you really… you

sort of don't have a lot of affiliation with them unless you're sort of doing a social event or something like that.

Interviewer: So it's closer in.

Respondent: Yeah.

Interviewer: So would it be [town], [town] area?

Respondent: I'd say [town] [town] [town] would be, yeah.

Interviewer: So what sort of distance would that be?

Respondent: So you're sort of looking at a radius, I mean it's [town] as well to a certain degree, but sort of a radius of around I'm thinking about 30 or 40 k's, yeah.

Interviewer: Wondered (?) that. Yes. OK. Now I was wanting to talk a bit about physical activity…

Respondent: Yep.

Interviewer: … and what does… and I was saying that it's a very broad concept, it's different for each individual. So what does the term physical activity mean for you?

Respondent: Well, for me anything from a really good go at the housework [laughs], if you do a really good job, to just going for a half hour walk or even a break in to a bit of jog. I mean I wouldn't really class myself as going to the gym

regularly, but I wouldn't mind being able to do it once a week, but I probably wouldn't go, regular membership or anything. Yeah, so that's about it for me, yeah [laughs].

Interviewer: And do you consider yourself to be physically active?

Respondent: Well, it's been very stagnant for me. I mean obviously…

Interviewer: The children?

Respondent: Yeah, as a mother I have been active but it's a different sort of active.

Interviewer: It's a different sort of, yes, very different.

Respondent: Yeah, and I guess having a baby that doesn't sleep well, being very sleep deprived, any excess time that I do have has been to get a little bit of extra rest myself just to get through, so the time that I would like to go out and do some exercise I can't really. And I guess because of where we are, I can go for a little walk and things like that, but not having a destination sometimes to go to can, you know, sort of don't… happy just to do it as much as you would [laughs].

Interviewer: Yes. We're going to go on and talk about that…

Respondent: Yep.

Interviewer: … so I'm really interested in your physical environment. So just where does physical activity fit in your life? Is it not of particular interest and priority, high priority?

Respondent: Well, for me, I mean after having children I would like to get back to somewhere near where I was before I started the whole adventure. I was a size 12 I'm now a 16, so for me it's quite, you know, I'd like to get back in to some of those old clothes that are sitting there. And I would like it to be more part of my life, but at the moment I'm struggling to be able to do it and keep it going. So and I guess for me as well, I mean I do have family that can look after the children to a… a grandmother that can look after the children at times, but I mean she's very involved in the community…

Interviewer: Busy herself.

Respondent: … and she's got the rest of the family that she looks after as well. And so really, I mean she can't take the boys all the time for all the things that I would like to do, and I do have day care for my son, my three year old in [town] because there's nothing else available in the area for family day care. I think there's something in [town], but at the time that we were looking that was full. So for me…

Interviewer: That's a bit of a trek for you.

Respondent: … not having, you know, and my partner works so much that he can occasionally take the boys for maybe a half hour here and there, and I can go out and have a walk in the paddock or whatever, but I don't… I would like to have a regular sort of, you know, this is my time. I've got a four hour block, I can do some exercise, come back, do a bit of paying bills on the phone or whatever I do, as a regular part of my week, my routine, but

it just hasn't been possible. If I had somewhere nearby, some care that I could drop the boys in for three hours or whatever.

Interviewer: 'Cause [town] isn't exactly that close.

Respondent: Well, half an hour, and by the time, you know, an hour a bit round trip, that's really, yeah, not really doable.

Interviewer: Yeah. So in our research we're sort of thinking about physical activity, we sort of think of any activity that lasts for at least ten minutes…

Respondent: Yes.

Interviewer: … that's the evidence of health fact, and so it causes your body to work harder than normal. You might get your heart rate up.

Respondent: Yep.

Interviewer: You might feel a bit huffy and puffy or warmer.

Respondent: Uh-huh.

Interviewer: So thinking about this type of psychical activity over the last two weeks…

Respondent: Yep.

Interviewer: … and I'll just go through parts of your life, not to separate out each part but mainly just to check that we're covering everything that's all.

Respondent: Yeah.

Interviewer: So, now, I just need to check, are you currently working?

Respondent: Just a little casual position, yeah.

Interviewer: Yep. OK. So in the past two weeks, have you been active at all for the last… for at least ten minutes?

Respondent: Not as part of my work, no.

Interviewer: 'Cause it's more a sit down…

Respondent: Sit down, take minutes, a bit of computer, yep.

Interviewer: Yep. And so it's the sort of type of work that you wouldn't, yeah, would be.

Respondent: Yeah, that's right.

Interviewer: And that would be usual for you? It's not, you know, like it's not that it changes with the time of the year or season?

Respondent: No. I mean the only activity that I would have would be going to the local online printer to print out minutes for, and I'd have to drive there anyway [laughter], yeah.

Interviewer: So if you wanted to, is there anything that would have helped you be more active at work?

Respondent: No, not with the type of position I have…

Interviewer: Not with the type of position.

Respondent: No.

Interviewer: No. OK. Now in the past two weeks have you ever walked or cycled for at least ten minutes to get to or from places?

Respondent: Not to and from places, no.

Interviewer: OK. Can you tell me a bit about why not, 'cause I presume it's because you have to get in a car...

Respondent: Well, I mean the distance I guess. I mean I do know of one woman in the community that, or two actually, that would run on the highway for however many kilometres or cycle, both of them do that. I would like to…

Interviewer: Brave women.

Respondent: I would like to be brave enough to do that but I can't. Log trucks and just, you know, people driving in general…

Interviewer: That's the general comment I'm getting. People don't feel safe to…

Respondent: And in the summer, I mean not just in the summer, but there's road kill everywhere and the stench of that just, you know, not so much in the winter. And snakes and I mean quite often I'm driving from home to

[town] and I'll see huge big snakes in the middle of the road and I'd hate to come across one of [laughs] those when I was running.

Interviewer: Yeah. So you're just off the main road are you?

Respondent: We're probably about a k and a half off the [x] Highway.

Interviewer: Yeah, so you're on the side road.

Respondent: Yeah.

Interviewer: Side dirt road?

Respondent: Yeah. Sorry?

Interviewer: Dirt road?

Respondent: No, well it's a sealed road, [x] Road which will take you to Mount [x].

Interviewer: Oh, you're off the [x] Road, yeah.

Respondent: Yeah.

Interviewer: I know where the turn off is.

Respondent: So, yeah, it's, yeah, a little bit off the main stretch.

Interviewer: OK. Yep. And if you'd wanted to, is there anything that would have helped you like walk or cycle to or from places?

Respondent: I think that if there were [laughs] cycle lanes and I'm willing to brave it, and perhaps…

Interviewer: You've always talked about more time too.

Respondent: … if I had more [laughs] time. I mean if I had a four hour block to get my exercise in to as opposed to half an hour like I am now, because by the time you get back I have to be on the phone to this person or whatever [laughs].

Interviewer: That and a safe place to be to.

Respondent: Yes, yeah, exactly, yeah. That's right.

Interviewer: Fair enough. Yep. OK. And in the past two weeks have you been active around the house or yard, so inside or outside probably?

Respondent: Yeah, I have.

Interviewer: Yep. Can you tell me a bit about what you did?

Respondent: Well, I've been trying to but the idea is that I'll go every day but so far in the last two weeks I've probably had about four days out [laughs] of that time where I've actually gone out and had a bit of a jog with the dog just up in the top paddock [laughs], the flattest one we've got, just to try and get back in to doing a bit more exercise. And apart from that, it's just walking around the property with the children. I might walk down the

lake or, yeah, kick the ball with my three year old and just general things like that.

Interviewer: And of course the housework [laughs].

Respondent: Yeah, and the housework [laughs].

Interviewer: So thinking about those three activities, what would be the intensity of them, would they be steady, moderate or vigorous?

Respondent: I'd say that, or with the children, just very steady a majority of the time, and when I get to go out for a jog, it's, you know, a get a bit of a huff and a puff [laughs], so…

Interviewer: So that would be fairly moderate, vigorous…

Respondent: Fairly moderate, yeah, to vigorous, yeah.

Interviewer: And the housework?

Respondent: I'd say that was moderate, yeah.

Interviewer: And how long would you have done those activities for at a time?

Respondent: I would say, well the housework if I'd had a, you know, I mean I do bits every day but when I have a good go a sort of a three hour, all the vacuuming and mopping and different bits and pieces. So, yeah, about three hours plus bits during the week. The jogging probably about 20

minutes to half an hour would be the time that that would go for. And playing with children, you sort of do…

Interviewer: You do that every day, yeah.

Respondent: …well, 20 minutes, half an hour at a time, yeah.

Interviewer: And the frequency. Now you've told me how often you do one of your activities, and about the housework and…

Respondent: About the jogging and…

Interviewer: Jogging, yes.

Respondent: … yeah.

Interviewer: You've told me about the housework. It's the jogging and the playing with the children.

Interviewe3: Yes. So, yeah, jogging probably, I mean like I said I'd like to do it every day, but it's only occurred in the last two weeks probably about four times. I mean I go for a walk, as long as the weather isn't foul; I go for a walk or to the park with the children daily.

Interviewer: So which park would that be? Down at [town]?

Respondent: Yeah, generally go to [town], and occasionally…

(Interruption – settling baby 16.13)

…yeah, occasionally we'd go to [town] but not very often. Obviously petrol's of a bit of a concern, just an expensive trip to the park.

(Interruption – settling baby).

Respondent: Anyway, we occasionally go to the [town] Park as well, yeah.

Interviewer: OK. And how far's [town] from you?

Respondent: It's about a 15 minute drive, so, yeah, 20 kilometres?

Interviewer: OK. Now thinking about those activities were they mainly during the day?

Respondent: All of them, yeah.

Interviewer: And would they be during the week or the weekend?

Respondent: During the week mainly, yeah.

Interviewer: OK. And who would you have done those with, obviously the children for one of them?

Respondent: The dog, the children and then by myself.

Interviewer: Yes. OK. So by yourself for the, which one?

Respondent: Mainly the housework. Occasionally going out for a jog I won't take the dog if he's just eaten or something, but, yeah, generally I will. He'll pull me along.

Interviewer: Yeah, for walks and things, yep. OK. And this is what you usually do.

Respondent: Yes.

Interviewer: And does it change depending on the season or time of year?

Respondent: No. No. I mean as long as it's not pouring down, I'll go running in the fog or… because it's on the property I sort of feel pretty safe that I know where I am and I know the surrounds and, yeah.

Interviewer: And the one thing about [region] it might be chilly but it's not going to rain forever.

Respondent: No, no. I guess the only thing would be that in summer with snakes and things…

Interviewer: Snakes, is the concerning.

Respondent: … yeah, I'm much more cautious than I would be, yeah, for myself and the children, mainly the children.

Interviewer: And if you wanted to is there anything that would have helped you be more active around the home, inside or outside?

Respondent: I think again having more care for the kids would allow me time to do that. That's probably the main reason, yeah.

Interviewer: OK. And your leisure or spare time. So that's just to cover everything if we haven't…

Respondent: Yeah.

Interviewer: … covered everything.

Respondent: Yeah.

Interviewer: Is there anything that you've done in your leisure or spare time for at least ten minutes?

Respondent: I have to be honest and say I don't actually have any leisure time, although I did go to a party a couple of Saturday nights ago and I had a bit of a dance for the first time in I can't remember how long. Yeah, so occasionally things like that crop up but it's not very often.

Interviewer: Yes. OK. And if you wanted to is there anything that would have helped you be more active in your leisure or spare time and that's time you've talked about that.

Respondent: Yeah, time, I guess…

Interviewer: And the facility too, you've actually said that [laughter].

Respondent: Yeah, I mean we do have a little gym at the school that is accessible, but I haven't been able to get along because I haven't had anyone to take the boys and some mothers can take their children in there but mine would not allow me to do that [laughs] unfortunately, yeah. I would actually really like to get in there once a week and use that because it's a great thing to have and it would be awful to lose it through lack of interest, yeah.

Interviewer: OK. I want to talk a bit about the physical environments now, that's the last section, and when we talk about physical environment I meant the physical things that are surrounding you, so it's the roads, the trees, the houses, the shops, traffic, you know.

Respondent: Yep.

Interviewer: So it's the environment that you live, work and play in. And of course some of these things are things that people in urban areas have said can be important for their physical activity, so we're interested in finding out whether it's relevant in rural areas too.

Respondent: Sure. OK. Yep.

Interviewer: So can you tell me a bit about how easy or difficult it is to be physically active in your neighbourhood, or your area?

Respondent: I guess it is quite easy because, well, if you live on a property definitely…

Interviewer: 'Cause you can actually use your property.

Respondent: Yeah, you can just…

Interviewer: Walk.

Respondent: Yeah, that's right. And I mean living on the farm, once the boys get a little bit older I'll be doing more hands on work with sheep and that type of thing, so I'll be able to pick up on my physical activity through that way which will be good. It's, yeah, no I think if you've got the time and the space that it's fairly easily, but having said that, I mean if you don't have the motivation, then it would be equally as easy just to sit [laughs] there and not, you know, cook in the kitchen and sit by the [laughs] fire and read a book and all that kind of thing.

Interviewer: Yeah, absolutely. OK. So I'll just go through a couple of areas here, availability and accessibility of places to be active, if I were to ask you about places to be active in your area, what would you tell me about?

Respondent: I'd say that, well, like I mentioned the little gym area which…

Interviewer: In [town]?

Respondent: … yeah, which would be fabulous to be able to access that. We have what is called a recreation ground, but there's not really much recreational there that I know.

Interviewer: Is that [town] or [town]?

Respondent: [town], yeah. I mean there are playgrounds for the children, but there's not a great deal for, well, older children and adults as far as bike tracks and things like that, any facilities. Fairly non-existent…

Interviewer: And that would be [town] and [town]?

Respondent: Exactly, yeah.

Interviewer: And [town] for that matter.

Respondent: Exactly, yeah. So I mean [woman] quite often has little things going on, walking groups and things like that, and if I didn't have worries of babies sleeping at certain times etcetera etcetera, then I would be able to make an effort to go along to things like that. I guess also with those things in a community, you may go along and you may feel that it's just not worthwhile because sometimes you, I don't know how to put this nicely, but you may not gel with the people in the group and you may not have anything in common or be able to have much more of a conversation past, so where do you live, and what do you run there, and anything outside of that. Some people are more open and willing to conversation than [laughs] others, and you sort of think, 'oh, gosh, why do I bother coming?' And, yeah, that type of thing happens as well [laughs], yeah.

Interviewer: OK. So you've talked about availability and not availability…

Respondent: Yeah.

Interviewer: … and access, are places convenient? Are they easy to access in terms of cost or hours?

Respondent: Well, I know that the [town] gym is free, and it is open outside of school hours definitely until about six in the evening. So I'd say that's fairly accessible. There's never anyone in there. It's such a shame that, yeah, it doesn't get used more than it does. And I guess occasionally there are classes and things, you know, [woman] might offer to bring someone to visit to put on a Zumba class or something like that, and it would be… I mean it would be great if they could have a bit more of that more regularly because you might miss one for one reason or another and not be able to make the benefit of it. And, yeah, like it's a long way to go to [town] or town to do a gym class.

Interviewer: Absolutely. OK. And do you think having or not having places to be active then influences whether you're active or not?

Respondent: Well, to a certain degree, you know, walking with the dog or going for a jog, is one way of exercising. But I think depending on your motivation level, sometimes getting involved with a class and doing it with other people is a very motivating factor, and gives you a bit of sort of, oh, I could have gone along to that class type thing, as opposed to, should I go out and go for a run or should I stay inside and, you know, do something else?

Interviewer: Yeah. So it's something quite useful.

Respondent: Yeah, I think so.

Interviewer: And if you wanted to be more active, are there things or places that would help if they were available or more accessible? Now obviously childcare's one of those things.

Respondent: Yeah, I think childcare, and I'm not sure why it's deemed that it's not necessary to have a government funded childcare facility in the area, or a non-government for that reason because babies are popping out left, right and centre from what I can see. There are a lot of young families in the area, and they all, I guess they're all in the same situation. Some of them are lucky enough to have grandparents that will have enough time to look after children, you know, for large periods. But, yeah, I guess all of them are travelling to [town] for childcare basically, if they use it. So that's a big, well, for mothers anyway. And I mean I know other people go to do swimming in town and things like that, or to [town] with the swimming pool there which has been a good facility that's come about down there. Yeah, that's about it really.

Interviewer: Now destinations, you mentioned destinations before, earlier.

Respondent: Uh-huh.

Interviewer: If I were to ask you about places that you could walk or cycle to from home, what sorts of things would you tell me about, for instance shops, schools?

Respondent: Well…

Interviewer: And what you've been saying is that you wouldn't actually walk or cycle from home.

Respondent: Well, I wouldn't because of the, you know, there's barely enough room on the roads for vehicles let alone… and they're not hard, the edges are all gravel and broken off and, you know, you don't know what you're going to drive past, you know, dead carcasses and goodness knows what else gets thrown on the side of the highway, which is really appalling that people do leave junk and all sorts of things on the side of the ride. It does happen, and, yeah.

Interviewer: OK. And do you think not having destinations influences what you… or having destinations influences you to be active or not?

Respondent: Well, having lived in suburbia as well, I do know that with young children for me to be able to… I mean when I was living in [city] I would quite often pack a little bag and go down to the supermarket and get the things that I needed daily as opposed to now doing click and collect online shopping and collecting it on the way out of town once a fortnight. Yeah, it would be more of a, you know, what do we need for the next couple of days? I'll walk down to the shop, I'll purchase it and walk back again and then I could incorporate my exercise in to that, which I don't have that now, no.

Interviewer: No. OK. So it does influence…

Respondent: I think so to a degree, yeah.

Interviewer: And if you wanted to be more active, would it help if there were more destinations within easy walking or riding distance, or within easy distance locally really?

Respondent: Yes, it could, but then I think it's really the way to get there is the problem as opposed to the destinations. And I mean I can't see that there would be any likelihood of any sort of thing being put between where we are in the nearest town because there's just no need for it. But I think if the condition of the roads had improved and that there was some sort of allowance for cyclists, which I mean could only benefit the tourist side of things as well. I mean you look at Tasmania as one of the top ten cycling destinations in the world, and yet we don't have any facilities, and I know that there are just so many injuries and fatalities with cyclists every year. I mean you just have to talk to someone that works in Emergency to say that how many cyclists come in with, you know [laughs].

Interviewer: It's certainly something that's been raised when I've talked with women in this whole study let alone the specifically the [region]...

Respondent: Right. Oh, OK. Sure.

Interviewer: Yeah, is road safety and not being able to walk on the edge, and one suggestion was actually just making a bit more bitumen available so you can…

Respondent: Yeah.

Interviewer: … put a cycle lane in and it's not very much.

Respondent: The really crazy thing is that probably three times a year, the local task force are going along the length of the lower highway at [x] Road putting

nice little bits of bitumen just to patch up the bits that have broken off the edge of the road from the log trucks and whatever else. If they just…

Interviewer: So they could be doing that anyway.

Respondent: …if they just put half a metre extra either side of the white lines that they paint, then perhaps they wouldn't need to be doing that at all.

Interviewer: Yes, that's true [laughter]. So it would be long term and more useful as well.

Respondent: You see them doing it and you're just thinking, 'oh, my goodness, who's idea is this?' It's just ridiculous.

Interviewer: OK. Just a couple of more areas left. I want to talk about the built features of your area.

Respondent: Yeah.

Interviewer: What sorts of things would you tell me about if I asked you about those, the manmade features?

Respondent: The manmade features?

Interviewer: Non-natural features, you know, well, we've talked already about the lack of footpaths or…

Respondent: Yeah.

Interviewer: … or areas along the road that you could walk…

Respondent: Yep.

Interviewer: … or cycle.

Respondent: Yep.

Interviewer: The other one's sort of things like lighting or walkability or cyclability, often it's not flat.

Respondent: Well, it is, you know it's…

Interviewer: It's not too bad?

Respondent: … pretty flat. It depends which way you go, I mean, yeah, if I want towards [town] way, it's a little bit of sloping but it's relatively flat. [town] way is bit more of an effort, yeah, but still doable.

Interviewer: Yep. OK.

Respondent: Lighting is, you know, country roads, unless you've got a set of headlights at night, you really [laughs] wouldn't do it [laughs].

Interviewer: And the other one's street connectivity.

Respondent: Yeah.

Interviewer: So say for instance in [town]…

Respondent: Uh-huh.

Interviewer: … is a better example than [town]…

Respondent: Yep.

Interviewer: …that this is what people comment about, is that you can actually get off the main road and there a few streets that you can actually connect…

Respondent: Yeah.

Interviewer: … and walk around close to the river, whereas in [town] it tends to be more of a major highway still.

Respondent: Well, they do have some backstreets but it's not as pretty, and you're sort of walking straight past someone's window, so it's a bit different.

Interviewer: So there's not that…

Respondent: Yeah.

Interviewer: OK. So do these built features in your area influence whether you're physically active or not?

Respondent: Well, having said that, I mean…

Interviewer: Yes.

Respondent: (Interruption – baby).

I just think that once the boys…

Interviewer: Do you want to stop for a minute?

Respondent: Oh, have you got much more to go or…

Interviewer: No.

Respondent: If there was better lighting and the roads were safer, then I could put the boys to bed and go out for a little bit of exercise before dinner or after dinner or whatever, yeah.

Interviewer: And if you wanted to be more active, are there features that would help?

Respondent: Just the lighting, and, yeah, upgraded roads mainly, yeah.

Interviewer: Now the aesthetics, the other side. The natural features, now you've talked a bit about the road kill and all of that stuff…

Respondent: Yeah [laughs].

Interviewer: … which is about the graffiti and that sort of stuff about the natural environment.

Respondent: Yeah.

Interviewer: But do you think your local environment is aesthetically pleasing and attractive near your home?

Respondent: Parts of it are, yeah.

Interviewer: And do the aesthetics actually influence whether you're active or not?

Respondent: Oh, definitely, yeah, 'cause I mean there's nothing better than going for a walk at sunset and seeing the moon come down over the hills, and, yeah.

Interviewer: And if you wanted to be more active are there any features of the area that might be more aesthetically pleasing or attractive?

Respondent: No, I don't think so [laughs].

Interviewer: And safety? That's the last one.

Respondent: Well…

Interviewer: If I talked about safety, what would you tell me about? Now you've already talked about road safety…

Respondent: Yep.

Interviewer: …and that influences… does that influence whether you're physically active or not?

Respondent: Yes.

Interviewer: Yes. And personal safety?

Respondent: Well, you mean for people in the area or…

Interviewer: Yeah.

Respondent: Yeah, well, I guess we have a lot of campers that come and camp in the area, 'cause we've got a camping ground at the end of our driveway, we're right on the lake. So there's generally more often than not there's someone around, so you sort of feel fairly safe, yeah. I guess at night it would be a bit different though.

Interviewer: Right. And most people are very sort of… walk at night, but mainly 'cause, well, it's not being able to see as well.

Respondent: Yeah, that's right.

Interviewer: So…

Respondent: That's it.

Interviewer: … if there's anything else that you don't think we've covered…

Respondent: Well, I don't know if my brain's able to think of it right now…

Interviewer: … about your… that's absolutely fine.

Interviewer: OK. So I wanted to talk first of all a bit about the place where you live, and could you tell me a bit about what it's like to live around here, your likes and your dislikes?

Respondent: Well, we moved down here nine years ago from [city], and we chose to move to a rural area because we thought if we're making a big change in our lives why just go and live in another suburb? So although we knew there were going to be drawbacks with transport, and work, and so on, we decided that having this wonderful rural community was worth it.

And it's actually nine years yesterday, and we don't regret it at all because this is a fantastic community. And you have things like [community centre], and the Radio Station, people that know you, people help each other. So from that point of view it's very good.

Our physical location is actually not bad – we live on the main road, we live two kilometres out of [town], so it is a little bit of a walk if I want to come to the shops and don't have access to the car, but we are on the bus route to [city], so I can get around to [town], [city], [suburb], anywhere I want to go, fairly easily, within the constraints of the bus timetable.

Interviewer: [Laughs]. So which way are you? Sort of [town] side?

Respondent: [town] side.

Interviewer: Yep. Yep.

Respondent: Actually, technically we're the last house in [town], but we give our mailing address as [town] because that way we get it delivered. If we put [town] we have to go down to the [town] shop and get it.

Interviewer: Ah, right! Yep, fair enough. And so how long ago did you say that you'd moved here?

Respondent: Nine years ago, yesterday.

Interviewer: Nine years, yeah. And what's the place you live in, is it a house block without acreage around?

Respondent: Yes, it's just a house block without acreage. We've got a fairly sizeable yard, but no acreage.

Interviewer: Yep, OK. And you're actually just a couple of kilometres out from [town]?

Respondent: Yes.

Interviewer: OK. So in our research with women living in urban areas, people often refer to the word neighbourhood.

Respondent: Uh-huh.

Interviewer: Uh-huh. And I'm interested in understanding what the term neighbourhood might mean to women who live in rural areas, if it's relevant at all of course. So what does it mean to you?

Respondent: Well probably not in the same way – when we first moved here there was a little group of houses. There was our house, the house next door, and a couple of others a bit further down, and I considered that was our neighbourhood. And we didn't really... apart from the next door neighbours we didn't really see anybody a lot.

Since we've been here several transportable homes have been built up behind us and around the back, so it's now become a slightly larger neighbourhood. But I don't really know the neighbours. Most of them rent, and they come and they go. So I guess I think of [town] itself as being my neighbourhood.

Interviewer: OK. So that's the couple of kilometres that it takes you to get in, and probably a couple of kilometres on the other side, is that sort of right?

Respondent: I'd say the couple of kilometres it takes me to get into [town], and the area of [town], probably bounded by [x] Road and the [x] Highway. So sort of taking in [x] Street...

Interviewer: So would that be another kilometre?

Respondent: Yeah, about that.

Interviewer: Yep. Yep. OK. And what about the word community – what does that mean to you?

Respondent: Community to me means I guess the people that live here that participate in activities in the town.

Interviewer: Uh-huh, uh-huh. Hmm, hmm, so that would be the [town] area itself?

Respondent: Yeah.

Interviewer: The same sort of distance?

Respondent: Yes, the same sort of distance.

Interviewer: Yeah. OK. So I wanted to talk a bit about physical activity and what does the word/term physical activity mean to you?

Respondent: Well I'm a self confessed lazy person, I don't care for playing sports or organised physical activity. I do like to walk, which is lucky seeing as I often have to anyway. So I'd say that physical activity generally to me is something that I don't want to do unless it's for a practical reason, like to get somewhere I want to go.

Interviewer: Yes. Yes. That's really clear. No, that's good. Do you consider yourself to be physically active?

Respondent: Not as much as I should be.

Interviewer: OK. And why would that be?

Respondent: Probably partly laziness, because as I said I don't really like organised things. I use to walk a lot when I first moved here, and now I definitely walk less. And I guess part of the reason is motivation, and part of it is that I'm probably not forced to do it as much because I know people now, and sometimes I get rides home instead of having to walk and so on.

When I was working I worked in physical jobs, and I thought well, I was getting quite enough exercise doing that. So since I'm no longer working in physical jobs – I was a [occupation] – I have noticed that I'm less fit.

Interviewer: Yep. OK. And where does physical activity fit in your life? Is it of no interest, of a priority, or a high priority?

Respondent: Definitely not a priority. It's something I... I wouldn't say it's of no interest, I'd probably say it's of little interest.

Interviewer: OK. Good. Yep, yep. No, it's really important to hear your views, and get a range of different views. So this is great. In our research, when we're thinking about physical activity we think about any activity that lasts for a duration of ten minutes – that's the sort of health benefit effect – and causes the body to work harder, you might start huffing and puffing, feel the heart rate get up, or you might feel warmer, a certain advantage in winter. [Laughs].

Respondent: Yeah, sometimes on a cold day the best thing to do is to get out and do something.

Interviewer: Yeah, yeah. So thinking about this type of physical activity during the last two weeks, I'll just ask you about some areas in your life. Now, you're working at the [workplace]?

Respondent: As a volunteer, yes.

Interviewer: Yes. Yes. And that's your main source of your work outside your home?

Respondent: Yes, it is.

Interviewer: Yep. So in the past two weeks have you been active at all at work for at least ten minutes at a time?

Respondent: Probably when I've been doing cleaning in the building – that'd probably be about the main thing, I think, and getting to and from there. Oh, and sometimes I walk, sometimes I go around to do the banking, and do the shopping, so I walk from the [workplace] over to [x] Street, to the bank, Post Office, and so on. In fact I did that yesterday.

Interviewer: Oh, OK.

Respondent: So that's the main way I'd be active when I'm at the [workplace].

Interviewer: So thinking about those activities, like the three that you've just mentioned, what sort of intensity would you describe them as – steady, moderate, or vigorous?

Respondent: What's the difference between steady and moderate?

Interviewer: It's really up to you [laughs] what you want to...

Respondent: Well I suppose probab...

Interviewer: Steady sort of means just generally doing things at a reasonable pace. Moderate you feel like you're putting some effort into it, versus vigorous seems to be [laughs]...

Respondent: OK, so steady is probably the cleaning tasks at the [workplace], probably the walks over to the shops because there's no real timeframe for that, so I can walk over. Sometimes I have to walk back with heavy shopping, but that's not a big deal.

Walking to the [workplace] in the morning, that's probably a little bit more moderate because I'm conscious that I want to be there by a certain time, so I sort of try and put my back into it a bit more.

Interviewer: Yep, yep. Yep. No, that's good. And how long would those activities be for?

Respondent: Cleaning – ten to 15 minutes at a time. Shops – the shops would probably be about a ten minute walk from here to the shops, and then back again, 20 minutes, and up and down [x] Street five minutes. From my house to here about 35 to 40 minutes is what I generally do it in, that's two kilometres – well actually I think it's a little bit over two kilometres.

Interviewer: OK. And how often would you do those activities?

Respondent: Well normally I'm at the [workplace] twice a week, and so I would probably do the cleaning activities each time that I was there, so that would be once a day. Shopping – I don't have to go out every time I'm there, it's only if we need something, so maybe once a week.

Interviewer: Uh-huh. And that would be about a 20 minute all up?

Respondent: Yes. And the walking I usually get... I'd say probably I'd do it about one trip in four these days – in winter time. You know if it's raining you're more likely to ask someone to give you a ride, because I don't particularly want to start walking and get drenched on the way.

Interviewer: Hmm, hmm. So in the last fortnight it would have been one occasion?

Respondent: Yeah. Well for example this week, because I was at the [workplace] for an extra day yesterday, I got a ride there, but I walked home.

Interviewer: That's what I was just going to ask. Yes.

Respondent: And this morning I walked in, and I'll probably get a ride home.

Interviewer: Oh, OK. OK, so it sort of averages out that once a fortnight you would walk in and walk out?

Respondent: Yeah.

Interviewer: Yep. Yep. OK. And when would you do this activity?

Respondent: In the mornings usually because my reception shift is mornings, so generally I'll leave home about half past eight, planning to be at the [workplace] close to nine.

Interviewer: Yep. And is this what you usually do, or is it one off?

Respondent: No, that's what I usually do.

Interviewer: Uh-huh. And does it change depending on the season or the time of year? Like would you walk more often if it was summer versus winter?

Respondent: Yes, I probably would. It's partly weather conditions that mean that I don't walk as often as I use to, now that I've got the option of getting a ride from time to time. So yes, weather is definitely a consideration.

Interviewer: Hmm, hmm, especially as we're in the middle of winter at the present moment.

Respondent: Hmm.

Interviewer: And if you'd wanted to, is there anything that would have helped you be more active at work? It sounds like you're pretty active for the couple of days you're there. [Laughs].

Respondent: Yeah, not that I can think of. No. I mean a lot of it is sitting at the computer, but I try and get up and move around because I have some back issues, so I know that I need to get up and stretch from time to time.

Interviewer: Yep. OK. And in the past two weeks have you walked or cycled for at least ten minutes at a time to get to and from places?

Respondent: Walked yes, cycled no, because I can't ride a bike. [Laughs].

Interviewer: And we've talked about walking to the [workplace], but are there any other occasions where you've walked?

Respondent: In the last fortnight – let me think? I think I've been into [city] in the last fortnight, and if I go into town on the bus, which I frequently do, I'll walk around town, and quite often I'll work my way that I get off the bus at [building], and I'll catch it in [x] Street to go home. So I'll kind of walk a city block or two.

Interviewer: Uh-huh, uh-huh. OK. And what would you call the intensity of that exercise?

Respondent: Hmm, steady.

Interviewer: Uh-huh. And how long for?

Respondent: Hmm, when you're shopping it's always hard to say because you're stopping, and you're starting, you're standing, and browsing, but maybe for actual walking probably half an hour at least.

Interviewer: Uh-huh. Uh-huh. And how often would you have done that in the last week? Was that about once?

Respondent: About once I think. I don't go up to town that often these days.

Interviewer: And that was during the day?

Respondent: Yes.

Interviewer: Yep, yep, and during the week. And why did you do it at that time?

Respondent: I had some errands to do in the city.

Interviewer: Yep. And OK, were you alone or with others?

Respondent: No, I was alone.

Interviewer: Uh-huh. And is this what you usually do, or was it one off?

Respondent: You mean being in town, or do you mean being with people?

Interviewer: Hmm, hmm, being in town and walking.

Respondent: I do it on a fairly regular basis. Yeah, probably about every couple of weeks I would do it.

Interviewer: Uh-huh. And does this change depending on the season or the time of year?

Respondent: No, not really.

Interviewer: Uh-huh. OK. And if you'd wanted to, is there anything that would have helped you walk, or walk to or from places?

Respondent: No, I don't think there's anything that would have made a difference to that. I don't find it a problem walking around town, even though [city]'s quite a hilly city.

Interviewer: [Laughs]. Yes, it can be that.

Respondent: Not like [city] which is as flat as a tack.

Interviewer: [Laughs]. Yep. In the past two weeks have you been active around the house or yard, either inside or outside your home, for at least ten minutes?

Respondent: Inside yes, outside no.

Interviewer: OK. So could you tell me about what you did?

Respondent: A couple of big house cleaning days going all around vacuuming. We have pets, so we have to vacuum a lot. Cleaning the bathroom, scrubbing showers, and bathtubs, and so on, change sheets on the bed, those sort of things, hanging out washing.

Interviewer: And the intensity of that exercise, would you call it steady, or moderate, or vigorous?

Respondent: Vacuuming tends to be fairly vigorous, the rest of it probably steady. [Laughs].

Interviewer: It does, doesn't it? Yes. [Laughs]. That's where you really get warm. [Laughs]. And how long would you have done those activities for?

Respondent: On the days that I did them, usually over the weekend I'll have a big cleaning day, probably three or four hours of doing jobs, get them all out the way, and then you can sort of have a lazy day the next day.

Interviewer: Yep, yep. And how often would you have done them?

Respondent: Probably twice a week I have a really good cleanup.

Interviewer: Yep, yep. And OK, and do you usually do that during the week, rather than at weekends, or it could be weekends?

Respondent: It tends to be more weekends. I do it during the week if I haven't got a lot of commitments outside.

Interviewer: OK. Yeah. And who would you do it with? By yourself?

Respondent: Nobody usually. [Laughs].

Interviewer: [Laughs]. Yes. The dog supervises? [Laughs]. Yeah.

Respondent: And is helpful jumping on the chairs after I've just vacuumed them.

Interviewer: [Laughs]. And is this what you usually do, or is it one off?

Respondent: Yes, it is what I usually do. Yep.

Interviewer: Yep. And does it change depending on the time of year or the season?

Respondent: No, not really.

Interviewer: OK. And if you'd wanted to, is there anything that would have helped you be more active around your home, either inside or outside?

Respondent: Well one thing that would help me be more active outside the home is if I had more help inside the home. [Laughs].

Interviewer: [Laughs]. Yes.

Respondent: Other than that, probably if I was able to access high ceilings and things more easily I'd be able to do more. There are jobs that I'd like to do, but I'm not good with ladders, so that would probably help if I had a – I don't know, either had a better ladder, or a person to climb it for me would probably be best. [Laughs].

Interviewer: [Laughs]. Be handy, yes. OK. And thinking about your spare time or leisure time, have you been physically active for at least ten minutes during that time? Is there anything we haven't covered basically?

Respondent: Most of my leisure time I tend not to be physically active.

Interviewer: Hmm, hmm. Fine. OK. And if you haven't been, why is that?

Respondent: Personal preference.

Interviewer: [Laughs]. Hmm. Just stop and be. [Laughs].

Respondent: Yes. You've just walked back from the [workplace]; you don't sort of really feel like running around doing anything really active for the rest of the afternoon.

Interviewer: Fair enough. If you'd wanted to, is there anything that would have helped you be more active in your leisure or spare time?

Respondent: No, not that I can think of.

Interviewer: OK. Now, getting onto physical environments – we've talked about where you live and physical activity, I just want to talk about your physical environment where you live, and work, and play. And when I talk about physical environment I mean all the physical things that surround you – the roads, the trees, shops, traffic, those sorts of things.

So, and some of these things are things that people in urban areas have said are relevant to their physical activity, so we're just wanting to find out does that apply to people who live in rural areas. So can you tell me a bit about how easy or difficult it is to be physically active in your area?

Respondent: Probably because of my lack of transport at times it does mean that I'm confined to a smaller area, and weather is definitely an issue. For example as I've said, if I'm not confident that the weather will stay dry I don't like to walk up from – I don't mind so much walking home, but I don't particularly like to walk to the [workplace], because then you're going to be there in wet clothes, which is not ideal obviously.

The other thing is that when it's consistently wet here in the winter time, as it sometimes can be day after day, it gets very muddy, and because there are no footpaths between where I live and the town it's sometimes difficult to negotiate. It means that no, if you walk in the mud you might slip, which I have done once or twice, or you walk on the road but we have log trucks and whatnot coming down the road, so obviously I don't like to do that very much. So that's definitely a little bit of an issue.

Interviewer: So where do the footpaths stop in relation to where you live then?

Respondent: The footpaths in [town] come down to about where [x] Road meets the Highway, and all the way back to my house, which is down by the [building], basically it's just verge.

Interviewer: Oh, OK.

Respondent: Just mud.

Interviewer: OK. So that would be about how much of – you were saying you're two kilometres into [town]?

Respondent: That would be most of the two kilometres.

Interviewer: Oh! OK.

Respondent: Hmm. So you're either walking on the... there's some sections where you're walking on grass, that's not too bad, but there are some muddy patches, and once or twice I have been unfortunate enough to slip over, and not hurt myself, only my pride, and get mud all over me.

Interviewer: Hmm. No.

Respondent: But obviously something that you would rather not do in that kind of weather.

Interviewer: Absolutely. No. And is that a quieter road?

Respondent: No, not really, because it's the [x] Highway.

Interviewer: Oh, OK! So... alright. So most of it hasn't got a...

Respondent: Most of my walk is along the verge of the highway, and it's... there are lots of lovely places that you can walk around [town], but you have to negotiate the highway to get to them.

Interviewer: Yep, yep. OK. Good point. OK. So what makes it... getting back to it, what makes it difficult? Is the lack of access in terms of footpaths and stuff like that?

Respondent: Yeah. Uh-huh. Yeah.

Interviewer: Yeah, OK. And lighting – is there any lighting?

Respondent: Lighting would be an issue. Well I wouldn't do it at night because I don't have really good night vision anyway, and although there are a few lights I certainly wouldn't want to be walking beside the road at night, not with heavy vehicles going by.

Interviewer: No. OK. I just want to check a couple of features of the physical environment with you. First of all availability and accessibility of places to be active, and if I were to ask you about places to be active in your area what sorts of things would you tell me about?

Respondent: Right. Well there's the football ground which is about halfway between my house and [town], and I do know ladies who go and walk there, round and round, and round the oval, and I have been there a few times with the dog on the lead, but I'm not really a great fan of walking around in circles.

Interviewer: No, it's a bit limited, isn't it?

Respondent: Yeah, it is a bit limited. I mean it's fun if you're with someone, you can chat, but if you're just doing it on your own it's not very interesting. And as I say I like to have a destination. When we first moved to [town] and everything was new to me, I use to walk a lot more, and I often use to get my husband to drop me off somewhere with the dog – not the dog we have now, an older dog – and we use to walk all over the place exploring different streets, and different areas in [town] and [town].

And there was also a place down [x] Road – I don't know if you passed that on your way in today – which was accessible, it's on the road to the [x] farms in there, and it use to be fairly accessible to go and walk there. I could let the dog off the lead, she could trot round, I'd go for a walk.

But then the owners fenced it off, and about that time the dog died. And we got another dog, but she's bigger and more boisterous, and needs a harder run, so my husband takes her down to [x] Point to run with the dog at the leash-free park there. And that's three kilometres from my place, so that's a little bit further than what I want to walk.

Interviewer: Oh, OK. So it's three kilometres which way?

Respondent: That's towards [town]. That's down by the [town].

Interviewer: Yes. Yep, yep. OK.

Respondent: So that's just a little bit more than what I want to walk, there and back. So I don't tend to do that very much.

Interviewer: Yeah, and does that mean walking along the main road?

Respondent: Yes.

Interviewer: Hmm, hmm.

Respondent: Yeah, everything from our place means walking along the main road for at least a kilometre.

Interviewer: OK. So you talked about your neighbourhood area, and I just needed to check out are there any other parks or recreation facilities that...?

Respondent: There are. There is the park, [x] Park in [town]. There's the football ground as I said. There's a stretch of, well I suppose you'd call it picnic area at [town], near the [building]. And there's the Sports Centre there, which I know has got a pool. So there's a few facilities.

Interviewer: OK. And are they... is access to those places convenient? Are they easy to access in terms of opening hours, or safety, or cost?

Respondent: Fairly easy to access as far as I know for opening hours, and the park, you know you can pretty well go there... go to any time. It's only probably an issue of lighting with those – you wouldn't want to go there after dark. Walking and to get to them, it's about the same as any other place I'd go – I'd either walk or I'd say to my husband can you take me to such and such, and pick me up afterwards?

Cost – I think most of the things around [town] don't cost that much. I've never looked into the cost of using the facilities at the Sports Centre, so I don't really know about that. But I think it's probably not that much.

Interviewer: Hmm. OK. And does having, or not having, places to be active influence whether you're active or not?

Respondent: I'd say to a degree it does.

Interviewer: Yes.

Respondent: Because as I said when I use to walk with the old dog, I was doing a lot more walking than I am now, and now it's sort of different places, the accessibility of where I use to like to go is not there, and the dog is a different type of a dog, so probably not doing that as much has changed things.

Interviewer: Hmm, hmm. And if you wanted to be more active, are there things or places that would help if they were available or more accessible?

Respondent: That's a hard one to answer. I think that the places that are, the local places are accessible to me; that it's probably more a matter of motivation. There's a walking group here in [town] – you may have seen them this morning – the [walking group]?

Interviewer: I've heard of them. Yes. [Laughs].

Respondent: And I would actually quite like to go out with the [walking group], but they walk on Tuesday mornings when I'm at the [workplace]. So if I had access to that group by changing my day, or if they walked on different days, I would probably do it. I could go to [town] and do it, but I'd rather walk with a local group, because a lot of the people who are in the [walking group] are people that I know, so it would be more pleasant to walk with acquaintances.

Interviewer: Hmm, absolutely. Hmm. OK. Now destinations – you mentioned destinations before. If I were to ask you about places you could walk to from your home, what sorts of things would you tell me about?

Respondent: I can walk to the shops in [town]; I can walk to [x] Park; I can walk to the football ground; to the [town] Sports Centre and Marina complex; I can walk to [town], but I probably wouldn't want to walk back afterwards.

Interviewer: [Laughs]. Because that's three kilometres, isn't it?

Respondent: That's probably about as far as I'd go just walking. If I could get a ride to somewhere else, then there are probably other spots around [town] I'd go to.

Interviewer: Hmm. So does having destinations influence whether you're active or not?

Respondent: Yes.

Interviewer: Yeah, you like to have somewhere that you can actually walk to?

Respondent: Yes. I like to look at things, so I often think oh, I'd like to go into [city] and look at this suburb, or that suburb, and explore an interesting looking path that I've seen. But then it's sort of getting the motivation to go out of [town] to do it.

And I guess – I don't know if you'll come into this later – but companionship has a bit to do with it, because my husband has arthritis in both knees, and can't walk very far.

Interviewer: Oh, that's tricky.

Respondent: So it means it's sort of just me on my own a lot of the time.

Interviewer: Yeah. Yeah. Yep. OK. And if you wanted to be more active would it help if there were more destinations within easy walking distance?

Respondent: I'd say yes, it would.

Interviewer: Yep. OK. Just going to look at a couple of other things – one's neighbourhood design, so that's if I was to ask you about the built or manmade features, the non-natural features of your area, what sorts of things would you tell me about? And you've started talking to me about there's no footpaths into [town] from where you are.

Respondent: Yeah. Well, footpaths and lighting are definitely that something that make walking much easier, and realistically they're not going to put a footpath all the way down out of [town] because there's very, very few houses – you know, there's just a couple of pockets of them here and

there – but certainly having a flat easy surface to negotiate would make a lot of difference.

Interviewer: Oh OK. So it may not be a total sort of footpath, but at least having it a bit flatter and not pot-holey and all that sort of certain...

Respondent: Not pot-holey, and not muddy in winter time.

Interviewer: Yeah. So well maintained, the verges.

Respondent: And the road verges also on the side of the road, I usually try and walk facing the oncoming traffic because it's safer, the road – the verge has a bit of a camber, which means you're sort of walking like that [demonstrating], and that's quite tiring on your feet, which is I have to admit has put me off doing it a bit.

Interviewer: Yeah. That's been a comment from a lot of women that we've been talking to, is that it's that camber that makes it so difficult, so you actually literally have to walk on the road, and it's just too hard.

Respondent: You do, and you don't feel safe walking on the road because of the heavy traffic. It's uncomfortable to walk on the slope, and that means that you get tired more quickly, and you're also probably more likely to injure yourself.

Interviewer: Well exactly, because you're putting stress on in an unnatural way on the body.

Respondent: Yeah, so if you have knee issues, or back issues, that can't be good for you. So that's definitely an issue.

Interviewer: Yes, yes.

Respondent: You know, if they ever widen the road we may not have a verge at all, I don't know. [Laughs].

Interviewer: [Laughs].

Respondent: The stretch of road that I have to negotiate to get to [town], half of it is 100 kilometres an hour zone, and then you get to the [town] 80k zone.

Interviewer: Which does make you feel vulnerable.

Respondent: So yeah, it does make you feel a bit vulnerable, even though I know that people watch out for you when they come. But if a truck goes past you that close [demonstrating], you don't feel very comfortable. And if they have to veer into the middle of the road because they see you, that's not very safe for them either.

Interviewer: Hmm. No, that's a good point. And that's come out very loud and clear in the study, is about road safety.

Respondent: Yeah. And not ideal for dog walking either. My dog's used to the traffic now, but when she was a puppy she use to absolutely get into a panic at the sound of a big heavy truck going by. She'd try and throw herself into a ditch at first, and then she later on started trying to jump on the road

and attack them, and then she got to the stage where she ignored them. But it makes dog walking not that pleasant.

Interviewer: No. And it overall doesn't make it very pleasant at all.

Respondent: No.

Interviewer: No. No. It's not exactly your idea of walking is it, to have to go on the main highway.

Respondent: It isn't. So it's a shame... I mean this is such a great area, and I suppose it's my fault that I never learnt to drive – there are lots of lovely places you could walk around here, and if you go up I[x] Road there's some lovely little parks, pocket parks on the side of the road that you can go to. There's nice tracks up around the dams on the other side of [town], but you have to get to them first.

Interviewer: Hmm, hmm.

Respondent: And that's what puts me off, and that's why I say lack of motivation to do that two kilometres to get to the starting point to go somewhere else.

Interviewer: Yeah. Yeah. No, that's... yeah, it's hard. It's very tricky. OK. And the other question around that is street connectivity too – like you've been talking about you have to go along a main road, but there's also that stuff walkability is about whether you can actually get onto streets that connect with streets. Do you have that in [town]?

Respondent: I'd say that most of the streets that I have walked around in my explorations of the town are pretty accessible. There are some that are very, very steep, especially going back towards [town], and I like views, so I like to climb up a hill and see a view, but some of these are really steep.

Interviewer: Oh OK. Yes. Yes. So after you got over the obstacle of the main highway, there are some streets that do connect with each other, and you can walk to?

Respondent: Yes.

Interviewer: Yep, OK.

Respondent: Yeah, so that's not a problem.

Interviewer: OK. So, and do the built features in your neighbourhood or your area influence whether you're physically active or not?

Respondent: I think so, because if you live in pleasant surroundings you enjoy being out in them, more than say if you didn't have such pleasant things to look at. And if I can come up to [town] and go for a walk in [x] Park, walk by the river, maybe be lucky enough to see a Platypus, or at least see some green grass, and trees, or flowers...

Interviewer: OK. So getting back to it, now we were talking about the footpaths and lighting and things like that, so does that actually affect – because I'll get onto the natural elements of your environment next – but do they influence the footpaths or the lack of them, or a steady place to walk, do they affect whether you're physically active or not?

Respondent: Yes, I'd say they do.

Interviewer: Yep. And if you wanted to be more active, are there any built features in your area that would help?

Respondent: I think it would really help if they could level out the road verges a little bit, and probably do something about some of the potholes, maybe gravel or something like that, so that you wouldn't be worried about slipping over.

Interviewer: Yeah, yeah. And even if... like they've got to put sort of dolomite stuff on the side anyway, that actually sort of helps your traction anyway.

Respondent: Yes, it does.

Interviewer: Hmm, OK. That's good. That's a good comment to make. Now the other side, the natural part of your environment, which is what you were starting to talk about – if I were to ask you about the aesthetic qualities or the attractiveness of your area, what sorts of things would you tell me about? You told me about the parks.

Respondent: Well, the parks... aesthetics is probably very important to me. I enjoy looking at nice settings when you go into parks, the river. I like being able to see the mountain, and even though I've been here nine years, snow is still a bit of a novelty, so I always enjoy seeing a bit of snow on the hills, and I'd love to actually be able to go to the snow a little bit more often than what I'm able to. And it doesn't snow down here in [town], to sea level very often.

I also enjoy built environments. I enjoy walking around pleasant suburbs and looking at houses, and looking at people's gardens and things like that. And I do that a lot for fun if I'm walking.

Interviewer: Hmm, hmm. And do you think your local environment is aesthetically pleasing, like near your home?

Respondent: Yes, I do. Yes.

Interviewer: OK. And do the aesthetics of your area influence whether you're physically active or not?

Respondent: I believe so, yes.

Interviewer: Yeah. Yeah, they help you get out and about, and want to do it really.

Respondent: Yeah. I mean if it's ugly outside you're probably less... for me at least, I'm less likely to want to be there. I'm more likely to take my exercise somewhere else.

Interviewer: Hmm. Hmm. And if you wanted to be more active, are there features of your area that might help if they were more aesthetically pleasing or attractive?

Respondent: Oh, well I wouldn't be living here if I didn't think this was an attractive area. There are a lot of new houses going up which I don't think are that attractive, but you can't do much about that. [Laughs].

Interviewer: [Laughs]. Yep.

Respondent: So no, I'd say it wouldn't probably change things all that much.

Interviewer: No. And the last area is just on safety – if I were to ask you about safety in your area, what sorts of things would you tell me about? And you already have about (inaudible – over talking – 36:24) traffic.

Respondent: Yeah, lighting and traffic, definitely.

Interviewer: Yeah. So does road – and you've already said road safety does influence whether you're physically active or not – does personal safety influence whether you physically active or not?

Respondent: No. I don't really worry about the way people would in a town about somebody attacking me, or accosting me when I'm out.

Interviewer: No, it's a safe community though?

Respondent: I feel this is a safe community.

Interviewer: Yep. Yep. And if you wanted to be more active, are there personal or road safety issues that if addressed might help you be more active? Now, we've already talked about one of them, are there any others?

Respondent: I suppose that it's not a fix, but it would probably make me feel more secure if I was out in the evenings – and on a summer evenings, because you have the lovely long twilights, you might walk if maybe you had some high visibility clothing or something. But ultimately it doesn't really

change – you know the traffic is heavy, and the verges are not really adequate.

Interviewer: And I guess if it's starting to... more houses are built out there, they might have to review their road speed zones for...

Respondent: Yeah, I think so. Hmm. Well [town] is a 70 kilometre zone, but between [town] and [town] it is highway speed. If a lot more houses were built I could see where people would probably... the people along the roads might start to feel that they would like the speed limit dropped, but I can also see where a lot of people would complain because it's a highway that they shouldn't have to keep on slowing down.

Interviewer: Yeah. On the other hand it's actually quite confusing because it chops and changes all the time.

Respondent: It's very confusing. Yes, I know.

Interviewer: Hmm. And I mean that's part of the broader debate they're having at the present moment.

Respondent: Yeah, it is. And that's a whole different issue. But I would say that it will probably be some years before that will happen here anyway.

Interviewer: Yep. Yep. OK. Thank you for that.

Respondent: That's alright.

Interviewer: But before we finish up, is there anything else you'd like to tell me about where you live, your activity, or the physical environment, that we haven't covered today?

Respondent: There was something on my mind, and it's gone again.

Interviewer: Don't you hate that when it happens?

Respondent: Yeah, I do.

Interviewer: [Laughs].

Respondent: It seems to happen to us more often as we get older, doesn't it? I think things...

Interviewer: Oh, I put it down to there are too many things in the world to think of now. [Laughs].

Respondent: Well that's probably true. Things like the walking groups, and the other activities that are going on here around the town, and sort of based in [community centre] and some other places, are probably a good thing that at least they get you out of the house. So if you're out interacting with other people you're more like to be – even if not having very hard physical activity, at least you're more likely to perhaps meet somebody who's place you could walk to, or who might want to go for a walk with you, and that sort of thing. So I guess just being out in the community is an important part of it.

Interviewer: Yep. No, that's a good point. Thank you for that.

Respondent: That's alright. I'm glad I was able to contribute.

Interviewer: So I thought I'd start by talking a bit about the place where you live. And so can you tell me what it's like to live around your area and your likes and dislikes.

Respondent: In regard to exercise?

Interviewer: No just about living there.

Respondent: In [x] Road it's a quiet area.

Interviewer: And you're in...?

Respondent: [x] Road.

Interviewer: Which is in...?

Respondent: Central.

Interviewer: Central to [town].

Respondent: Yeah. And there's footpaths and there's quite a bit of a walk to the main streets but it's close to schools and things like that. And it's close for my daughter to walk if she wants to but she usually takes the car. Other than that I can't think of anything else.

Interviewer: So do you live in a house and house block?

Respondent: Yes.

Interviewer: The length of time you've been living there?

Respondent: 28 years.

Interviewer: You're well settled [laughter]. And your reasons for living there? Why did you pick where you're living…?

Respondent: Family home bring the children up in that home and then after divorce I stayed there.

Interviewer: So you've always live in [town] yourself?

Respondent: No I was born in [country] and I came out here when I was three, and my parents settled in [town] and that's where I've stayed.

Interviewer: OK nice spot. So you're fairly much in the township itself, how far are you from the actual town centre?

Respondent: To walk probably about half an hour, five minutes drive.

Interviewer: So that's sort of about a kilometre or something?

Respondent: Probably three or four I reckon.

Interviewer: Three or Four? So in the research with women living in urban areas people often refer to their local neighbourhood. And I'm interested in understanding… what the term neighbourhood means for women living in rural areas and whether it's relevant. What does the word neighbourhood mean to you?

Respondent: Just residential.

Interviewer: So if you needed to put a distance around that, what would you… is it the sort of next few streets around you what would your neighbourhood be?

Respondent: Probably a kilometre radius.

Interviewer: What about the word community what does that mean to you?

Respondent: The town. The town and all it encompasses.

Interviewer: And that would be how far if you had to put a distance on it?

Respondent: From my house?

Interviewer: Uh-huh how big an area are we talking about?

Respondent: A radius of probably five kilometres.

Interviewer: Talking about physical activity now, and as I said it's a very broad term and very individual in what it means. So what does the term physical activity mean for you?

Respondent: Keeping fit, aerobic exercise, and I actually deliver junk mail to this area actually [laughs], so I do that twice a fortnight…

Interviewer: Bit of walking around with that one…

Respondent: Yeah and quickly because this is not a great area to live in. We call it [nickname] I'm afraid.

Interviewer: Fair enough you get the sort of different sense of... this is the second time I've come up and there is a different feeling here than over the river, and even where I'm staying, I stayed in a local B&B on the outskirts. You're right I get the feeling [laughs].

Respondent: Well I parked my car down there and as soon as I left it I thought make sure I lock it, remember that I've locked in because in this area I mean you've got to be careful.

Interviewer: OK so we've just talked about what it means to you to be physically active. Do you consider yourself physically active?

Respondent: Yes.

Interviewer: And why?

Respondent: Because it feels good when you've got rid of all the aches and pains [laughs], I mean physical activity cures a lot of the aches and pains.

Interviewer: So you know when you've been active you start feeling literally better in yourself?

Respondent: Yeah. I wouldn't say I'm fit though [laughs].

Interviewer: Yeah well would we [laughter]! Now where does physical activity fit into your life is it no interest or a priority or a high priority?

Respondent: It is a priority I suppose I wouldn't say it's a high priority otherwise I would be doing a lot more like these ladies.

Interviewer: For that moment in time that they're doing it. OK. In research we think about physical activity as being any activity that lasts for at least ten minutes, that is the sort of recognised health benefits. So that's when you start huffing and puffing a bit, feeling warmer – an advantage in winter. Or you just feel like your body is working a bit harder than normal. So thinking about this type of exercise and activity over the last two weeks in your life, are you working at all at the present moment?

Respondent: For settling purposes no but yes.

Interviewer: Only because I want to ask about different parts of your life, not to segment your life but to make sure we cover things.

Respondent: Well I do have a segmented life because I have different jobs that I do.

Interviewer: So I will ask you in the past two weeks have you been active at all at work for at least ten minutes of the time?

Respondent: Yes.

Interviewer: And can you tell me what you did?

Respondent: Junk mail delivery which takes up an hour and I walk fast anyway but carrying weight and walking quickly, so that takes an hour. We've done it twice in the fortnight so that is far as work. And I also do housework for an elderly lady so that's another hour's work.

Interviewer: So all of your work activities are actually physically active activities.

Respondent: Yes.

Interviewer: And can you tell me what intensity those two activities would be, would you call them steady or moderate or vigorous?

Respondent: Probably moderate for me because I do everything quickly and I could do it faster. My heart races, so yeah it does have benefit put it that way.

Interviewer: And those two activities how long would you have done them for?

Respondent: An hour each so that's four hours in the last fortnight only for work.

Interviewer: That is what I mean the two activities of house cleaning and also junk mail is four hours. And when do you do those activities, like during the day?

Respondent: Yes.

Interviewer: Is this what you usually do or is it one off?

Respondent: This is what I usually do.

Interviewer: And does it change depending on the season or the time of the year?

Respondent: No.

Interviewer: If you wanted to is there anything that would have helped you be more active at work?

Respondent: No I am active.

Interviewer: That is the nature of your work that is fine. In the past two weeks have you walked or cycled for at least ten minutes at a time to get to or from places?

Respondent: Yes.

Interviewer: And can you tell me what you did?

Respondent: Walked the dog.

Interviewer: And what intensity would that have been – steady, moderate, vigorous?

Respondent: Vigorous, fast walking.

Interviewer: And the duration how long?

Respondent: Dog walk takes about 45 minutes.

Interviewer: And how often would you have done it in the last fortnight?

Respondent: Probably two or three times a week so four to six times in the last fortnight.

Interviewer: When would you have done it, what time of the day?

Respondent: Any time, whenever he looks at me and says I want to walk.

Interviewer: So that could be early in the morning or it could be somewhere during the day or it could be in the evening.

Respondent: Yeah anytime.

Interviewer: And weekends?

Respondent: Not usually evenings but yes weekends. Also walks up to the street to do shopping and stuff like that.

Interviewer: And the intensity of walking to go shopping?

Respondent: Moderate.

Interviewer: And how long might that have taken you?

Respondent: Ten minutes there and ten minutes back.

Interviewer: And how often might you have done it in the last two weeks?

Respondent: Probably twice.

Interviewer: What time of the day would you have done that?

Respondent: Afternoons.

Interviewer: Afternoon. During the week or weekends?

Respondent: During the week.

Interviewer: Where would you have done? Well you've told me where you do the shopping so obviously the shop [laughter] but where do you take the dog?

Respondent: Beach.

Interviewer: His/her?

Respondent: Him.

Interviewer: His [laughs] favourite place. I've got a saying at home I have a dog a [dog breed] and it's like my favourite thing, my favourite place, my favourite [laughs]...

Respondent: [dog] is a [dog breed] too.

Interviewer: Oh right active [laughs].

Respondent: Not mine actually it is my fiancée's. We live in separate houses so if I'm at his place we will take his dog. It's his dog.

Interviewer: Who enjoys going out and about [laughter]. So you usually go to the beach and why, why did you do it there and what attracted you to that place?

Respondent: To the beach?

Interviewer: Uh-huh.

Respondent: The freedom for the dog to be able to run off, favourite spot I suppose.

Interviewer: And you've got a dog access beach because I noticed I had a little walk this morning there is a prohibited area...

Respondent: There is and we choose the day when there is not many people anyway. This time of the year there is not many people down there And other people…

Interviewer: Unnecessary restriction isn't it?

Respondent: Uh-huh.

Interviewer: And were you alone or with others?

Respondent: Others.

Interviewer: Usually with your fiancée…?

Respondent: Yeah.

Interviewer: And is this what you usually do?

Respondent: Yes.

Interviewer: It’s not a one off. Does it change depending on the season or the time of the year?

Respondent: Yes I suppose it would. If it’s summer time then we avoid the crowds.

Interviewer: You’d have to go somewhere else to…?

Respondent: No we would go evenings or we would walk the streets.

Interviewer: It wouldn’t change the fact that you would go for a walk for instance.

Respondent: No.

Interviewer: If you wanted to is there anything that would have helped you walk more often?

Respondent: No.

Interviewer: In the past two weeks have you been active in the house or the yard like at home inside and outside?

Respondent: Yes.

Interviewer: For at least ten minutes.

Respondent: Yep.

Interviewer: Can you tell me what you did?

Respondent: Well inside I did some painting [laughs], painting the walls. Outside I did some gardening mainly because I had a birthday coming up and I wanted to spruce everything up before everyone arrived [laughter]. And lots of cleaning and changing beds and vacuuming and all that sort of stuff that encompasses with people coming to visit. So yes we had a busy fortnight [laughs].

Interviewer: It sounds like a fun time as well.

Respondent: Yeah.

Interviewer: And the intensity of that, those different forms of exercise we're talking about, painting and doing the whole cleaning up and gardening.

Respondent: Probably moderate, it wouldn't have been at full pelt because it was going to take awhile anyway, so it was just plodding along and enjoying it – pottering.

Interviewer: That's a sensible way of doing it. And how long would you have done it at any one time?

Respondent: The painting took all day. The gardening probably two or three hours in the garden because there's mowing and then there's cleaning up.

Interviewer: And how often might you have done that in the last fortnight?

Respondent: Once [laughter].

Interviewer: Once for the gardening and once for the painting – [laughter] that's enough. And when would you have done that?

Respondent: What time of day?

Interviewer: Yeah what time of day.

Respondent: Gardening would have been just after lunch because it's warmer, and the painting was from morning til night.

Interviewer: Was that during the week or at the weekend?

Respondent: During the week.

Interviewer: Who with?

Respondent: Gardening on my own, painting with my fiancée.

Interviewer: Is it what you usually do or was it one off?

Respondent: One off.

Interviewer: For the painting. The gardening?

Respondent: Yeah I get out there and do that by myself all the time.

Interviewer: And does it change depending on the season or time of year?

Respondent: Oh yeah. More in the summer but the winter you don't need to get out and garden that much.

Interviewer: The garden sort of takes care of itself a bit more.

Respondent: Yeah.

Interviewer: And if you'd wanted to was there anything that would have helped you be more active around home either inside or outside?

Respondent: No.

Interviewer: Have you been physically active for at least ten minutes during your leisure or spare time?

Respondent: Yes.

Interviewer: And can you tell me about what you did?

Respondent: Weightlifting.

Interviewer: What would be the intensity of that one?

Respondent: Depends what I chose to do but I try and do it morning and night, probably for about ten minutes until I'm huffing and puffing [laughs]. Then if it's at night time then I can just drop into bed.

Interviewer: So you do that at home the weightlifting?

Respondent: Yep.

Interviewer: And usually about ten minutes at a time and usually the frequency of usually a couple of times a day?

Respondent: Once at least. If I'm in a better mood morning and night.

Interviewer: I'm impressed in how you fit it in.

Respondent: It's only ten minutes but you do feel better for it.

Interviewer: That is interesting. When would you normally do that?

Respondent: In the morning first thing and at night the last thing.

Interviewer: So it's usually the morning one?

Respondent: No it's usually the night one. It just depends I get up and get ready, get my daughter ready for school and all that sort of thing.

Interviewer: And why do you do it at that time?

Respondent: Because I'm usually left alone to do it.

Interviewer: It does make a difference. And you've told me that you usually do it at home. Any reason why you prefer to do it at home?

Respondent: I don't like going to gyms or anything like that. I'm a private person I would rather be pulling funny faces on my own thank you.

Interviewer: Fair enough. Who would you have done it with is it usually by yourself?

Respondent: By myself.

Interviewer: And is that what you usually do?

Respondent: Yes.

Interviewer: And does it change depending on the season or the time of year?

Respondent: No.

Interviewer: If you wanted to is there anything that would have helped you be more active in your leisure or spare time if you wanted to?

Respondent: No.

Interviewer: The last bit of it is talking about physical environment that you work and live and play in. When we talk about physical environment I mean

all the physical things that surround you like roads, trees, houses, footpaths, traffic. And some of these things that people living in urban areas have said they are relevant for their physical activity, so we're interested in asking whether that's the case in rural areas as well. So can you tell me a bit first of all about how easy or difficult it is to be physically active in your area?

Respondent: It's very easy. There are footpaths and where I'm situated is near the overpass near the highway. So there is an underpass to go under. Just before you get there there is a ramp that goes down to it or there is the steps. And what I normally do is walk down, go down the ramp, run up the steps and do a circuit and then jog home. So that is great.

Interviewer: So that is something else you do.

Respondent: That is something else I do do but not in the last fortnight I haven't had time.

Interviewer: That is fair enough that is something that you don't usually do. You do that some of the time.

Respondent: I do. I do that probably twice a week when I've had time and the weather and it's harder to get out in the winter.

Interviewer: So you would more likely do that in summer.

Respondent: I do it about 7.00 o'clock at night and it's cooling off.

Interviewer: So you do that a couple of times and how long would you do it for?

Respondent: It probably takes me about ten to 15 minutes because I walk down but I try and jog back if I'm not too exhausted [laughter].

Interviewer: And what would be the intensity it sounds pretty vigorous?

Respondent: Yeah it is vigorous. 'Cause jogging up the steps there's quite a few steps. I did count them... I can't remember now 32 perhaps.

Interviewer: Oh wow that would certainly get your heart rate up. OK. So we're talking about it is pretty easy to be active in your area. OK Why, what makes it easy?

Respondent: Footpaths. The footpaths. Also there's not too many trees, and just to flip the coin there are enough trees so that people aren't watching me [laughter].

Interviewer: Fair comment. I'm going to ask you about a couple of areas about physical environment. The first one's availability and accessibility. If I were to ask you about places to be active in your area, what sorts of things would you tell me about?

Respondent: Cycling – there's a beautiful cycling path from [town] to [x] Beach. That is great because they've put some little rest points along the way, so if you're not used to it you can rest on the seats. It's either cycling or walking.

Interviewer: Does that start close to where you are?

Respondent: It actually starts down near [x] School, the beach, the [x] beach but we cycle down there, cycle to it and then cycle along that path and then back again.

Interviewer: So it's the continuation of… just takes you down. OK so there's some cycle tracks and that's a joint walking track as well isn't it?

Respondent: Yes.

Interviewer: Because a few women have mentioned it. It seems to be a real highlight here.

Respondent: Yeah. The parks are great here. The parks are good for even just to walk or just to cycle through.

Interviewer: Recreation I presume there's a whole range of recreation things.

Respondent: I'm not into sports so that doesn't interest me.

Interviewer: OK. So there are places to be active that you want to... in terms of what you want to do and it's accessible for the things that you want to do too… does having places to be active influence whether you're active or not?

Respondent: Yes. Yes it does, especially with cycling. As you get older you get a bit more scared to cycle in traffic. When we were children we did it all the time.

Interviewer: I must admit I'm with you there. I gave up when I first started living in rural areas I gave up riding, cycling because of the main roads and I'm just getting back into it but I've got to get my road sense...

Respondent: Confidence back. Yeah you do. Once this bridge is done too that'll make a difference too.

Interviewer: Oh is it going to be wide enough that it's....

Respondent: I wouldn't ride on bridges or the roads but the footpath is wide, it looks like it's wide enough.

Interviewer: OK so they have got footpaths on both...

Respondent: No only on one side. That'll be good too 'cause that's stopped our access from the [town] footpath which is where we used to take the bike. To actually do the bike we've got to go over the bridge and then onto the main road and then quickly try and get off again, and it's a little bit hairy if you're not used to it.

Interviewer: It's always busy it seems to be busy when I've come along there doesn't matter what time of the day…

Respondent: That will make a huge difference when that is done and it's safer to stay on the footpath. I know you're not supposed to but anyway.

Interviewer: Actually quite a few women have made that comment around cycling and I can understand that because of the safety feature. Unless you've

got a designated cycle lane it's their choice too because of the safety feature is the footpath...

Respondent: I would rather get booked than hit by a car [laughs].

Interviewer: Well exactly, that's the trouble with cars I mean you're the one that comes off worst all the time.

Respondent: That's right and you hold the traffic up too.

Interviewer: OK. And if you wanted to be more active are there things or places that would help you if they were available? That's what we were saying about some…

Respondent: I'm just thinking of horse riding that would be good but I don't know of anywhere that offers horse riding here. No can't think of anything else, no it's pretty much well covered here.

Interviewer: Destinations, if I were to ask you about places that you could walk or cycle from your home, what sorts of things would you tell me about? You've told me about a few things already.

Respondent: Yeah I would walk to the shops but I wouldn't cycle.

Interviewer: Is that because again the road safety?

Respondent: The roads but also you never know what you are going to bring home and whether you can put it in your backpack so I would rather walk.

Interviewer: And you've told me about the beach. And you can walk from home to the beach?

Respondent: Yes.

Interviewer: So it's just about whether there are friends, schools, post boxes all those sorts of things, so where you are you would have all of those within walking distance?

Respondent: Mostly but I wouldn't always walk time restraints usually.

Interviewer: Always a juggle. So you can when you try to and you can when you've got the time.

Respondent: Yes.

Interviewer: Does having a destination influence whether you're active or not?

Respondent: Yes. For instance, if you want to go to the beach well that is a destination or the park or where to ride and then you have got to plan your route.

Interviewer: And if you wanted to be more active would it help if there were more destinations within easy walking or cycling distance?

Respondent: Yeah probably but I can't think of anything that would be an attraction.

Interviewer: I think you've talked about that a bit in terms of the continuity with the bridge and over the other side that gives you access to [town] and the beach. Is it the beach that's along [town]?

Respondent: No [town] cycling. Because I would start at my fiancée's house which is just this side of the bridge and then we would go over the bridge.

Interviewer: I'm with you, and then it sort of links and there is a cycle path all the way along is it?

Respondent: Well you can go through all the parks, you can cycle through the parks and then you come to the cycling path to [x] Beach, so you can cycle all the way and stay off the roads.

Interviewer: Very nice. Neighbourhood design. Now if I were to ask you about the built or man-made or non natural features of your area what sorts of things would you tell me about? You've already started talking about footpaths.

Respondent: There is [x] Park that is a natural one though, been down there a few times but this is man-made.

Interviewer: So for instance lighting if there is adequate lighting when you are walking.

Respondent: There is adequate lighting.

Interviewer: And just about the walk-ability of the place?

Respondent: Yes the footpaths are quite well maintained really.

Interviewer: And whether the streets connect together like some people live in a long strip and there is nothing of that, whereas [town] is quite different.

Respondent: With [x] Road I suppose it is dead-end but there is [x] Park down the end. You used to be able to go along a little track next to the highway but since they have put up their sound barrier that's not there anymore. So really that is a dead-end you have got to go down and come back again.

Interviewer Oh you used to be able to get through.

Respondent: You used to be able to go around on a track.

Interviewer: So that sort of cut the connection.

Respondent: Yes.

Interviewer: Do the man-made features in your area influence whether you're physically active or not like having reasonable footpaths?

Respondent: Yes it would because if there is not that you don't want to walk on the road all the time or gravel.

Interviewer: If you wanted to be more active are there any built features in your area that would help?

Respondent: Sorry can't answer that one [laughter].

Interviewer: No because [town] done quite a bit of work on that.

Respondent: Yeah.

Interviewer: That's fine. The other side of it is the aesthetics which is the natural qualities in your environment. If I were to ask you about the aesthetic qualities or attractiveness of your area what sorts of things would you tell me about?

Respondent: About the trees and the environment and that sort of thing. Yeah they have done a lot of planting of trees when they upgraded the highway and that's nice.

Interviewer: And you've told me about the beautiful natural features of the coast [laughs].

Respondent: Maintained the parks and all that – no they do a good job. I guess you take things for advantage really because you see all these beautiful shrubs and you admire them you look at the different things – yeah it's taken for granted [laughs].

Interviewer: That's what living in a beautiful place is about. It's being part of living rurally in a lovely place but you don't sort of take out the beauty part because it's so much part of being there anyway. Yeah I understand. And remember some of these questions are obviously asked in urban areas which is often there is quite a difference between whether there are some nice natural environment around. Do you think your local

environment is aesthetically pleasing near your home, sort of attractive?

Respondent: Yeah.

Interviewer: Do the aesthetics of your area influence whether you are physically active or not?

Respondent: Yes.

Interviewer: Having some beautiful places really does make a difference...

Respondent: Uh-huh.

Interviewer: And if you wanted to be more active are there features of your area that might help if they were more aesthetically pleasing or attractive?

Respondent: No.

Interviewer: Because it’s there already [laughter] and they’re doing some really good things as well. OK. The last area is about safety. If I were to ask you about safety in your area what sorts of things would you tell me about?

Respondent: Probably about the tunnel because it’s a bit dark and dingy.

Interviewer: Why is that? What’s that about?

Respondent: It's near [company] and it's the walkway under from [x] Road that connects up to [x] Street and it goes across the double highway so it's quite a long tunnel, and the lighting isn't really adequate in there. So when I go for my little jogs around the circuit I always look down the tunnel to see if anyone's coming. And if they are then I'm consciously aware of where they are, how close they are to me and try and time it so that I'm going around the ramp while they're going away, depending on the time of the day too.

Interviewer: Does personal safety influence whether you're physically active or not?

Respondent: Yeah it would.

Interviewer: So you've just given me an example of where you may have some caution. There's always the sort of antenna out. But generally in [town] is personal safety an issue?

Respondent: Not in general but when I am delivering the junk mail in this area...

Interviewer: You've got your own antenna out.

Respondent: I have but [fiancé], he delivers with me on a different... similar but around different areas. So he's there somewhere.

Interviewer: And I presume you've got a phone with you [laughs].

Respondent: It's in the car but [laughter]...

Interviewer: But you're in the general area. But does it in general influence whether you're physically active or not?

Respondent: No not really no you can take precautions and you can take safeguards.

Interviewer: Does road safety influence whether you are physically active or not?

Respondent: Yes, especially with the bike riding because I don't want to ride on the roads and making sure there is footpaths because you don't want to walk too close to the roads

Interviewer: That's in [town] itself as well.

Respondent: Well there are a few places that don't have adequate footpaths and they do need it I suppose.

Interviewer: And for cycling you are talking about in [town] too and that's just about having to deal with traffic as well.

Respondent: Yes.

Interviewer: And if you wanted to be more active are there personal or road safety issues that if they were addressed might help you be more active?

Respondent: If they could put cycling lanes in, yeah that would encourage me to be more active on the bike but that would be the only thing I think.

Interviewer: A lot of the streets I've noticed in [town] are wide enough you know it is a possible option.

Respondent: Yeah but then you get cars parking either side and they are not very wide anymore.

Interviewer: No that is true. OK. Now before we finish up is there anything that you'd like to tell me about where you live, your physical activity or the environment that you live in that we haven't covered today.

Respondent: No I think we've pretty much covered it. We are close enough here in [town] to go to [town], like we could drive to [town] and go up Mount [x] and do those sort of walk too. So it is close enough to other areas to take advantage of too. That is about it.

Interviewer: Thank you.

Interviewer: OK. So I thought we'd start by talking a bit about the place where you live first up. So could you tell me what it's like to live round here, your likes and dislikes?

Respondent: OK. Well I live at [rural town]...

Interviewer: Yes.

Respondent: Which is quite isolated.

Interviewer: Ah yeah. It's the [rural region] area is it?

Respondent: It is, yes. So sort of this side of [rural region], sort of between here and [rural town]. My husband and I chose to live there so we actually moved there because we liked the lifestyle. We're both outdoor people so we like walking, and we like, love fly fishing. It's one of our passions, which is why we love living in the area we live. We did move there for employment reasons, or change of lifestyle I guess. We left our jobs and actually bought the [rural town business].

Interviewer: Oh right.

Respondent: So that's sort of what made us move into that area. But we grew to like it more and more. We've been there 17 years I guess now.

Interviewer: Oh wow.

Respondent: Yeah, so...

Interviewer: That's a long time. I'll just... so we don't compete with the air conditioner.

Respondent: I can... I'll just turn the fan down.

Interviewer: No, that's OK. Lovely. That's OK. Those Dakin's are wonderful. Always like to have those.

Respondent: Fan should turn off. It's got a mind of its own.

Interviewer: And what's the type of property you live on? Have you got... is it a house and...

Respondent: OK. We actually own land...

Interviewer: Oh OK.

Respondent: ... so we're on a 1000 acres.

Interviewer: Wow that's a big lot of acres.

Respondent: They weren't selling small blocks so it was either that or nothing. So, we own a 1000 acres and it's sort of bushland. We lease it out to a local farmer so they run sheep.

Interviewer: Oh OK. Yeah.

Respondent: We also have horses so we run our horses on it. And we built, actually built. We'd been there for seven years and we actually built, bought the land and built on it, so.

Interviewer: Oh right. So you have done a lot since you've been...

Respondent: We have, yes. And since then...

Interviewer: That's an achievement.

Respondent: ... I've got employment in this area, so we sold our business. So I work now for [x], my husband works for an organisation called [x] so we both do a lot of travelling for our work, but both work in the [x] municipality which is fantastic for us.

Interviewer: OK. It is, to be able to work and live in the same spot...

Respondent: Definitely.

Interviewer: Now it's just on that topic, what's your nearest centre and sort of town centre relatively [indistinct – over speaking - 02.50]...

Respondent: OK. Well the nearest small town would be [town] which is 65 kilometres away. [Town] is the closest bigger town that would have more facilities like supermarkets, banks all the rest. So [town] is an hour and a half drive and [city] about... [city] [city] and [city] are all about a two hour drive from [town]...

Interviewer: Oh OK. And how far... how long would it take you take you to get to [town] from where you live…?

Respondent: It takes me about 40, 45 minutes, and I travel every day to either [town] or...

Interviewer: [town] isn't it?

Respondent: ... [town] quite often. I've been them to [town] this morning and back, so you live in the [rural region] and you have to always...

Interviewer: In the car.

Respondent: ... got to travel somewhere. Yes.

Interviewer: OK. In research with women living in urban areas it's the same, most of this has all been done in city based research. People often refer to their local neighbourhood, and I was interested in understanding what the term neighbourhood means to women who live in rural areas. So what does the word neighbourhood mean to you?

Respondent: It's interesting because I never... I probably wouldn't relate neighbourhood as a word I would use. I would use the word community.

Interviewer: That's the other question I was going to ask you, does the word community. So neighbourhood doesn't... doesn't feel relevant but community does.

Respondent: It does…

Interviewer: So what does community mean to you…?

Respondent: OK. Well I was brought up in the city so to me, that... my neighbourhood there would have been the actual neighbourhood you live in. So community to me probably means a lot different than a lot of people. Community to me could be the [town] people that live in the immediate area form a community, because in the [rural region] each little town sort of has its own sort of ways, they're all different. So that... I see them as all different communities. But then there can be communities within communities, so like a group of people with a similar interest, like fly fishing for instance, you could say that there's a little community around people that liked or are interested in fishing because they tend to mix together, play together and do things together. So that's what I...

Interviewer: So communities about interest and also about geography?

Respondent: Uh-huh.

Interviewer: And so the one around geog... well OK, for both, is there a distance that would be associated with that? Like the area that you're talking about for geography?

Respondent: Yeah. For... I would say community would be within, where I live at [town] you'd probably be looking at within a ten kilometre radius. So we've got [town] and then we've got [x] Lagoon that has some residents, and then [x] Lake. So all those people sort of would come to the shop at [town], so that would... I would say that would be a community. And there's a couple of properties might be a little bit outside that area, but I would probably see it in that geographic area.

Interviewer: And the fly fishing community, would that be...

Respondent: That could be outside the state.

Interviewer: Yep. So that's far bigger...

Respondent: Because there's a lot of people that come regularly to our area to fish, but they might be from... well, for instance we have a good friend from [USA] who's actually bought a place at [town] and he spends three months of the year here fishing, and there's a lot of mainlanders that do that.

Interviewer: So is there a particular season that people come fishing…?

Respondent: Yes. The season is usually your summer months, six months, but it's within the warmer months. It's just closed now. Yeah, winter can be quite quiet; winter is very quiet and lonely up in those areas. There's not much happening over winter.

Interviewer: OK. Now I wanted to talk a bit next about physical activity, and as we were saying it's a really broad concept. So I'm interested what the term physical activity means to you?

Respondent: OK. Well to me... well it's anything that keeps you moving. Look I've always kept active, so if you're going to be physically active I think you should be doing something most days of the week if you can, which sometimes I go weeks and don't do very much because of various reasons, busy at work, weather, but I try to do some structured exercise at least three to four times a week and then weekends try to actually get out and about. I mean if I spend all day inside, I try to get out and do something. So I think physical activity, although you can be doing it in your daily life which is really important, that incidental stuff, I really think that you need to structure something in to keep active, uh-huh. Because I know I can. There are some people obviously for physical reasons that can't do that too much, but there's always something you can do.

Interviewer: Uh-huh. Do you consider yourself to be physically active?

Respondent: Yes.

Interviewer: And why?

Respondent: Because I do exercise regularly and I do various types of exercise, although opportunities are very limited, which I'm guessing we're going to get to.

Interviewer: Uh-huh we are. Absolutely right there.

Respondent: Uh-huh. So I guess it's just that being active is part of my life.

Interviewer: Uh-huh.

Respondent: And I guess I have pride in it, or I like to keep myself fit and active.

Interviewer: Uh-huh, so it's part of life. And where does physical activity fit in your life? Like is it a... is a high priority, a priority or of no interest?

Respondent: High priority.

Interviewer: OK. Now to go into more detail, in our research, when we think about physical activity we think of any activity that lasts for at least ten minutes, the evidence of health affect, and causes the body to work harder, so you might get increased heart rate, start huffing and puffing a bit, and/or you might warm up, it's generally the benefit for it, especially in winter.

Respondent: In this weather.

Interviewer: Uh-huh exactly. So thinking about this type of physical activity, during the past two weeks, I'll have a look at different parts of your life only... I appreciate that physical activity is part of your whole life, but it's just we don't want to miss out on aspects of it, so we're just asking it this way. So

in the past two weeks have you been active at all at work for at least ten minutes at a time?

Respondent: Yes.

Interviewer: Uh-huh. And can you tell me what you did?

Respondent: Be walking and jogging.

Interviewer: OK. And would you describe that as steady or moderate or vigorous?

Respondent: Some moderate, a little bit vigorous.

Interviewer: And how long would you have done it for?

Respondent: At a time?

Interviewer: At a time.

Respondent: Uh-huh. 45 minutes to an hour.

Interviewer: And frequency?

Respondent: The last three, two weeks I'd say three times a week.

Interviewer: OK.

Respondent: Yeah I've been... haven't been that active the last couple of weeks.

Interviewer: And is there a particular time of the day that you would do that?

Respondent: Week days after work.

Interviewer: Now we’re talking about during at work.

Respondent: Yeah. Sorry.

Interviewer: At work.

Respondent: While you’re at work you mean?

Interviewer: Yeah. While you’re at work.

Respondent: Oh sorry, I misunderstood.

Interviewer: Oh OK. OK...

Respondent: No, no, no.

Interviewer: So let’s start again. That’s fine. So...

Respondent: So while I’m actually working?

Interviewer: This is while you’re at work.

Respondent: Oh OK. Sorry. OK.

Interviewer: Yep. No, that's fine. So in the past two weeks have you been active at all at work for at least ten minutes at a time…?

Respondent: At work. I'd say yes.

Interviewer: Yeah. And if... and can you tell me about what you did?

Respondent: OK. So this is unusual because normally at work I don't exercise 'cause I'm office based. But last week I took a group of women away on a women's retreat.

Interviewer: Oh right.

Respondent: Which was absolutely fantastic.

Interviewer: Oh wow. Where did you go?

Respondent: So... to [x].

Interviewer: Oh right.

Respondent: Most people have never heard of it, it's fantastic. It's a big beach homestead, four, four and a half star. Fantastic.

Interviewer: Where is it? Where's [x]]

Respondent: [x] is down [x] way.

Interviewer: Uh-huh.

Respondent: Uh-huh. Down that way. Highly recommend it.

Interviewer: OK.

Respondent: So I took... it was... I took a group of community women. So we did some walking and I did a job one day down there, so yeah. So that's the only time at work I've actually been active.

Interviewer: OK. So would you describe that intensity as vigorous or moderate or steady?

Respondent: Moderate.

Interviewer: OK. And the duration?

Respondent: An hour.

Interviewer: And the frequency, in the...

Respondent: Oh twice during the... twice during the, yeah, two weeks.

Interviewer: And so was that at a particular time of the day?

Respondent: Morning.

Interviewer: Morning. OK. And so you were saying that it's maybe not what you usually do, was it one off?

Respondent: Uh-huh. One off. Uh-huh.

Interviewer: Uh-huh. OK. And does this change depending on the season or the time of the year?

Respondent: No, not really. Not during work.

Interviewer: OK. Not during work. So if you'd wanted to, is there anything that would have helped you be more active at work?

Respondent: Ooh...

Interviewer: 'Cause you were just saying you were involved in walking groups and things like that.

Respondent: Uh-huh, which I haven't, which the last two weeks I haven't because we went away... because we went away and took... that was the group that went away. But normally on a Thursday morning I participate in the [x] Heart Foundation walking group, so I actually coordinate that. So we go on probably a 45 minute walk.

Interviewer: Walk. Uh-huh.

Respondent: But that would be the only time. Occasionally at [x] maybe once a month I'll meet with them for a walk.

Interviewer: Uh-huh. So would that intensity be moderate or vigorous?

Respondent: Low moderate.

Interviewer: Oh OK. So it's steady to moderate.

Respondent: So it's an older... yeah, sort of steady moderate it is.

Interviewer: Uh-huh. OK. No, but that's wonderful to be able to do things like that.

Respondent: Oh it is within your work.

Interviewer: Uh-huh. Now... so is there... if you had wanted to, is there anything that would have helped you be more active at work? Like more time or someone to be active with, or somewhere.

Respondent: I can't really see an opportunity with my workload that I could do that at this stage.

Interviewer: Uh-huh. OK. In the past two weeks, have you walked or cycled for at least ten minutes at a time to get to or from places?

Respondent: No.

Interviewer: And if not, tell me about why?

Respondent: There's not many places that are ten minutes away from where I live that I could actually manage that and there's no paths, there's no... I don't own a bike.

Interviewer: Uh-huh.

Respondent: All of those reasons. But it's mainly the environment.

Interviewer: It's very much driving and getting in a car to be able to get somewhere.

Respondent: Uh-huh.

Interviewer: OK. And does that change depending on the season or the time of year?

Respondent: No.

Interviewer: No. Do that usually, uh-huh. And if you had wanted to, is there anything that would have helped you use walking or cycling to and from places?

Respondent: Again not really because of the distance that I...

Interviewer: But the distance.

Respondent: ... have to travel to get anywhere. I mean you couldn't really even park your car half a kilometre up the road and walk to work because it wouldn't be safe to leave your vehicle, so it's yeah, it's just not something that I would do.

Interviewer: Uh-huh. And you were saying there's no footpaths around where you live, but also the destination that you would go to would be too far.

Respondent: Too far. That's right.

Interviewer: Uh-huh. OK. So how far out of... you were saying in terms of [town]...

Respondent: How far is my house…?

Interviewer: Yeah, from [town] …

Respondent: Yeah. So my house would be 300 metres from where the shop and things are…

Interviewer: OK. Now in the past two weeks, have you been active around the house or yard? So inside or outside your home for at least ten minutes...

Respondent: Is this structured exercise or does housework count?

Interviewer: It can be anything.

Respondent: Yep. I'd say yes.

Interviewer: Yes. And can you tell me about what you did?

Respondent: Oh actually yes. I did do a half an hour on the exercise bike, that would have been last, but I don't really like bikes, but I did [laughter]. And Sunday I did, probably half a day I was doing housework, which I would say was quite steady at the time. Yep.

Interviewer: Anything outside? In the yard.

Respondent: Oh are you talking around the house, like if you're going for a walk?

Interviewer: No. I'm talking about...

Respondent: No, the weather hasn't....

Interviewer: ... your home.

Respondent: The weather hasn't been very conducive to outside stuff. I love to get out in the garden but not until we get a bit better day.

Interviewer: Oh OK. Yes. OK. Now in terms of the housework, you were saying it's probably about moderate.

Respondent: Yeah like vacuuming and like sort of doing the bathrooms.

Interviewer: Yep. You've told me about where and when and all that. And who with? Would you do that alone or with others?

Respondent: Alone.

Interviewer: Yep. This is what you usually do. And does this change depending on the season and time of year? And it's probably one of the things to look at the outside, in the garden...

Respondent: The outside, yes, I do more gardening during the summer months or spring months, yep. Definitely. Winter, yes, I mean I do get outdoors a lot during the winter but we tend to bushwalk and things like that. But the garden where we live tends to look after itself pretty well over the winter.

Interviewer: Self. Yeah. So in summer how often would you get out in the garden?

Respondent: A bit spasmodic because we fly fish most weekends, but maybe oh on average I guess a couple of hours every few weeks.

Interviewer: Yeah. You've got a fairly native look after itself garden have you?

Respondent: Yeah. I mean in spring we'll do a good weekend or a couple of full days that my husband and I both get out to trim back and... but generally I like the garden to [laughter] look after itself.

Interviewer: It's good to get out in it. OK. So if you had wanted to, is there anything that would have helped you be more active around the house or yard either inside or outside?

Respondent: Probably better weather. Probably would have got outside more. But other than that, no, not really.

Interviewer: OK.

Respondent: More time [laughs].

Interviewer: So the last part of life I'm going to ask you about is, have you been physically active in... for at least ten minutes during your leisure or spare time?

Respondent: Uh-huh. Yes.

Interviewer: Yes. And can you tell me about what you did?

Respondent: Either I go to the gym after work and do walking machine and weights, or I walk from home, bushwalk from home.

Interviewer: OK. So in terms of the gym, where would that be?

Respondent: The school at [x] have a great gym that the community can use…

Interviewer: Oh OK.

Respondent: Which has been fantastic because they've had that for 12 months or so and for me it's great because I can go straight after work before I go home. Especially this time of the year because it's dark when I leave work and I can't...

Interviewer: Yes. So you just get in the car and just go down to the school...

Respondent: Go there for an hour and then go home.

Interviewer: Yep. OK. Now the... we've been talking about two activities, the gym and bushwalking, the intensity of those?

Respondent: I'd say moderate to vigorous.

Interviewer: OK. Duration, how long?

Respondent: An hour average.

Interviewer: And the frequency?

Respondent: Is this over the last two weeks?

Interviewer: Uh-huh.

Respondent: Yeah. Three times a week.

Interviewer: OK. And when? Oh you're saying with the gym it would be evening…

Respondent: The gym it's usually of an evening straight after work, so six o' clockish. At home, walking's weekends, Saturday or Sunday.

Interviewer: Yep. And would you do those activities by yourself or with others?

Respondent: The gym by myself. I try to encourage other people to come but they keep dropping off. Weekends, sometimes by myself, sometimes with my husband.

Interviewer: OK. And is this what you usually do or is it one off?

Respondent: No. This is usual, quite regular.

Interviewer: And does it change depending on the season or time of the year?

Respondent: Yes. So summer months when the days are longer I tend to go home and then go for a walk or a jog at home. But over the winter I'd use a gym more during the week. Weekends I'm probably more active again over the summer months, but the activity's different. Like I'd be out fly fishing and walking the lakes and where... this time of the year when the fishing

season's not on then we'll just go for a bushwalk, yeah, or a walk on our property or...

Interviewer: Uh-huh. OK. So that's sort of fairly immediate for you, you can walk. If you're picking bushwalking sort of, how far would you normally go to...?

Respondent: We have a lot of bush around us that we have access to so most of our bushwalking would be within a 20 kilometre radius, 15 kilometre radius.

Interviewer: And you mentioned horses one...

Respondent: We have horses but we haven't ridden for a while.

Interviewer: OK.

Respondent: Only 'cause we're so busy doing everything else.

Interviewer: Uh-huh, you can't do everything can you, that's the thing, and horses require...

Respondent: So they're not getting very much exercise.

Interviewer: Oh they're probably having a ball.

Respondent: They do. They're all quite old now, so they're sort of in retirement.

Interviewer: Oh OK. Fair enough. OK. Now let's see, now if you had wanted to, is there anything that would have helped you be more active in your leisure or spare time?

Respondent: In the last two weeks I'd probably say yes. But it's been a busy couple of weeks for me at work.

Interviewer: With work, uh-huh.

Respondent: And I actually had a couple of days off sick so that slowed me down, and I never get sick, so, yeah, so I went probably four or five days when I couldn't.

Interviewer: Uh-huh. And it... so is there anything that might help in terms of helping you be more active in your spare time?

Respondent: Uh-huh. This time of the year would be work not be so busy.

Interviewer: Uh-huh. You'd have a bit more energy for... and time.

Respondent: Well it's the time, yeah. So if I'm, if I've got days at [city] I'm travelling and I don't get back to the office 'till 6:30 it's too late to go to the gym and I have to get home. So works has a big impact, especially this time of the year, and we tend to be really busy this time of the year.

Interviewer: OK.

Respondent: And weekends the weather has a bit to do with it, cause if it's wet obviously I can't get out and bushwalk so that really limits what I can do at home exercise wise, although we get out in the cold and drizzle but...

Interviewer: When it's sleeting rain it's not much fun.

Respondent: But if it's snow and windy I tend to do housework so I guess I still do something. Housework needs to get done sometimes.

Interviewer: Uh-huh. It's a funny thing about housework, it's always there.

Respondent: It is.

Interviewer: Ok. Now I'm going to talk a bit more about the physical environments now. That's the next part of it. We've talked about where you live and your physical activity and now we want to talk about the physical environment you live and work and play in. Now when I talk about physical environment I mean all the physical things that surround you. Now that could be roads, trees, houses, shops, traffic, whatever, roads. So some of these things are things that people in urban areas have actually said might be important for their physical activity. So I wanted to ask whether they were relevant to you. Now first of all, can you tell me a bit about how easy or difficult it will be to be physically active in your neigh... in your own area?

Respondent: I would say generally difficult. Not difficult for me because I like to bushwalk and I'm able to do it. But for a lot of people it's difficult because they either for some reason either can't get out in the bush and they need a stable place to walk. We don't have footpaths, we don't have any lighting, we have log trucks and we have gravel roads.

Interviewer: Oh right.

Respondent: So you can't just go for a...

Interviewer: You can't actually just...

Respondent: ... walk up and down the road.

Interviewer: No. So you've actually got to drive to another spot to...

Respondent: Well you'd have a long drive. I mean even [town] doesn't have... isn't really conducive to walking safely. So it would be...

Interviewer: Oh OK. So for...

Respondent: ... it's really difficult for people that...

Interviewer: Uh-huh. So if you're not... well you would have to get in a car, if you were bushwalking you'd have you were saying a 20 kilometre radius, but not everybody can bushwalk.

Respondent: No.

Interviewer: So... but there's...

Respondent: And there's no other facilities. There's no gyms, there's no halls, there's no exercise path...

Interviewer: There's no parks...

Respondent: There's no parks.

Interviewer: Recreation parks.

Respondent: No, there's nothing like that, so.

Interviewer: Is that something you've got to...

Respondent: No, they'll leave a message.

Interviewer: OK. So it's quite difficult.

Respondent: And I mean even for me, like I can't exercise in the dark at all.

Interviewer: No.

Respondent: Because there's nowhere that's got any light that you can even just go for a walk.

Interviewer: No. It's not so...

Respondent: And then there's no indoor facilities to do anything, so, except for like an... we've got an exercise bike at home, but I don't, I really don't like... I do that if I have to.

Interviewer: Uh-huh I know.

Respondent: My husband does, but yeah, that's why I like... that's why the gym has been so good to me at [town].

Interviewer: Uh-huh. It's a really good opportunity isn't it?

Respondent: It is, yeah.

Interviewer: It's good. OK. So I'm going to ask you about some key aspects of physical environments. The first one is availability and accessibility of places to be active. So if I were to ask you about places to be active in your area, what sorts of things would you tell me about?

Respondent: OK. I would say, again to go for a walk in the bush, but I have access to that and I have access to my property which if you're visiting the area you wouldn't have any of that. I mean you can walk along the road, but only in daylight, and you have to be careful of the traffic. We do have like...

Interviewer: So I presume there's no particular shoulder, it's just the dirt road.

Respondent: Not at all, it's just a dirt road. And like I said it's used by log trucks and yeah. We do have [x] National Park which is fantastic, which is about a 30 minute drive...

Interviewer: Oh yeah, indeed.

Respondent: ... from where we live, so quite often I'll... we'll go and do a walk at the park. So that's great if you like to do that sort of stuff. But other than that there's not really much, no.

Interviewer: Uh-huh. So in terms of recreation facilities, I mean you've mentioned [town] with the gym at the school, which is...

Respondent: The school gym, uh-huh.

Interviewer: ... a community gym.

Respondent: Which you have to sign forms and you have to after hours. It's not... you can't use it during school holidays because it's alarmed, so... which is another reason that I wasn't so active during the school holidays, I couldn't use the gym, so that really makes a difference to me when it's not accessible.

Interviewer: So I mean that's the other one is access, so the place is convenient. So what there is has some limitations on it.

Respondent: Has limitations, uh-huh.

Interviewer: OK.

Respondent: And the other this is there's no... we don't have sports teams or anything, like you can't play netball or badminton or, 'cause it just doesn't exist.

Interviewer: Uh-huh. OK. So there aren't green open spaces and parks for people to... and walking tracks, cycle paths?

Respondent: No.

Interviewer: No. OK. And you were talking about sports, sports clubs and things like that. Is there anywhere to... for people to play...

Respondent: Look, I mean there's some local halls...

Interviewer: ... activities.

Respondent: ... but there's no activities organised at those. [town]'s the closest that have a cricket team but there's no sports anywhere in the [rural region] I know for women like netball, basketball, none of that exists.

Interviewer: Uh-huh. And there's no indoor sort of activity too, no?

Respondent: No. I mean the only sort of structured exercise would be what we organise through work, like the Heart Foundation walking groups.

Interviewer: Uh-huh, the walking groups.

Respondent: Which we have now. Oh, and there is one at [town] near [town]...

Interviewer: OK.

Respondent: ... and they meet on a Monday morning, but I work, so I'm not available.

Interviewer: Uh-huh. So how long have the walking groups been going for?

Respondent: [town] and [town] over 12 months now.

Interviewer: Right.

Respondent: Uh-huh. [town] started about three months ago.

Interviewer: Uh-huh. So they are quite new and...

Respondent: They've been fantastic.

Interviewer: So it sounds like it's a really good opportunity and certainly...

Respondent: We've tried to get walking groups going over the years before and they just haven't quite taken off. Something about the Heart Foundation ones gives a bit of structure to it.

Interviewer: Now the interesting one then given the sort of availability of places to be active is is obviously something the walking groups had to come to terms with.

Respondent: That's right. Uh-huh.

Interviewer: Where you're going to be walking, particularly with a group of women with various abilities. It's all women's walking groups or is it mixed?

Respondent: They are open, but they're predominantly women.

Interviewer: Uh-huh, it's funny that.

Respondent: [town] one has a few men, yeah.

Interviewer: OK. So where do you go walking?

Respondent: That's [colleague]. So, I'll just close that door.

Interviewer: Uh-huh. Where do the walking groups go walking?

Respondent: OK. So at [town], that towns probably set up better than any other town in [rural region] as far as paths and things go, and safety, so there we just do a trek round the streets and usually have two groups, a slower group and a faster group and we meet, start somewhere and meet back.

Interviewer: So there's enough footpaths, decent sort of...

Respondent: There is too actually, yeah.

Interviewer: And connection between the streets and pathways.

Respondent: Uh-huh. I mean it's still not the best. The paths aren't the best, but they are there. You've got to be careful.

Interviewer: And parks and walking trails to go... that connect on that…?

Respondent: No. There's a little park but it's only quite small. Now the [town] group did have a day where they... we took a bus down to town and went to [x] Dam and had a picnic and actually did the walk round the dam.

Interviewer: Oh OK.

Respondent: So that's something that they can do as a group...

Interviewer: Sometimes you do that.

Respondent: ... so that happens sometimes. [town], look I don't go over to that group but [colleague] does. They don't really have many paths either but they tend to pick sort of some out of the way roads that are safer to walk on.

[town], they meet... [town] is actually off the main highway, it's like a loop. It goes into... round the tracks and passed the houses, so they just walk up that road 'cause there's not much traffic…

Interviewer: Oh OK…

Respondent: But again it's... oh it is sealed now, so it's not too bad there.

Interviewer: OK…

Respondent: But don't have many choices though…

Interviewer: Yeah. 'Cause it was obviously something you had to resolve to set up the walking groups, it's, yeah.

Respondent: And a couple of them have spoken about... having trips where they go somewhere for the day. We had the discussion... we couldn't walk this morning 'cause it was wet.

Interviewer: Yes.

Respondent: So we were talking about ways that if it's wet again can we get a video in and do a dance class or...

Interviewer: Yeah, have an alternative activity.

Respondent: ... or walk round the hall and chat and walk, so things like that, just to get people moving...

Interviewer: Yeah. ‘Cause you get used to it and you want to... you like the activity I imagine…

Respondent: That’s right…

Interviewer: Yeah. OK. Now does having, or not having places to be active influence whether you are active or not?

Respondent: Yes. To a degree yes.

Interviewer: Yep. OK. And if you wanted to be more active are there things or places that would help if they were available or more accessible?

Respondent: Look it’d be great to have like structured sports, like netball or badminton, would be really great. Which could happen in one of the halls if you... I guess you got the people motivated to do it. I’d definitely be interested in that. Lighting is a big thing for me where I live because you just can’t go anywhere after dark. And often just...

Interviewer: You can’t see let alone they can’t see... anybody can see you.

Respondent: ...just walking past, just somewhere safe to walk even. And that’s a big ask I guess in the more rural areas. But even some of the places like [town] if they had a nice track or paths that went down the river or something, like even in your lunch break you’d probably be more willing to actually just for ten minutes go for a walk.

Interviewer: Yeah.

Respondent: Yeah. But because we don't have those things at all you just can't do it.

Interviewer: Yeah. OK. Destinations. If I were to ask you about places you could walk or cycle for instance, i.e. not drive, from your home, what sorts of things would you tell me about?

Respondent: Well walking for me, I can sort of go out my back door and just walk. So we border the [x] River so that's a really nice walk to walk down the river, along the river and back home again…

Interviewer: That's on your property is it?

Respondent: That's on our property.

Interviewer: Yeah.

Respondent: I can walk, quite often I can walk on to the neighbouring property, so, which is again, I mean I could sort of walk forever if I wanted to. And we also have access to, well it was [x]'s but it's now owned by [x]...

Interviewer: Oh OK.

Respondent: ... all the land, 1000s of acres that we, my husband and I have access to. When I say access it's gated with locked gates, and the [x] Trail actually goes through that area.

Interviewer: Oh wow.

Respondent: And because it was all forestry… they have quite good, if you wanted to just walk on the roads they have quite good gravel roads which doesn't get any traffic so it's quite safe. So I'm lucky in that respect that if I couldn't walk on uneven ground I could still have access to the roads.

Interviewer: OK.

Respondent: So to walk to that would be... the other thing I have access to is there's a canal, because it's all inland fisheries hydro operated up there there's a canal that runs between a couple of the lakes that's got a hydro road next to it. And that's a quite... quite a good road for me to walk on. This might sound silly, but in the summer it's really difficult to walk in the bush 'cause of the grass seeds and buzzies you get on your shoes, so I'll walk the road that goes alongside the canal. Again it's flat, I can actually jog on that safely. And it's a nice view 'cause you've got the water trickling by. So they're the places I would go that would be walking distance...

Interviewer: So you've really discovered and your own... like in your own locality what's... what works for you really...

Respondent: Yeah. And I know if I've got half an hour I can do this walk, or if I've got two hours I can do this one, so.

Interviewer: Yeah. But you were saying too like you live in the vicinity of [town], like is there shops or something that you could walk to or post box or whatever?

Respondent: Oh there's a general store...

Interviewer: General store.

Respondent: ... that I can just walk across our paddock straight to, and that's a post office as well, so I can walk to there and back.

Interviewer: Yeah. OK. So... and friends and like... all sorts of... OK. So does having destinations influence whether you're active or not?

Respondent: Yes it would, yeah. 'Cause it's easy having it right there what I like to do.

Interviewer: Yeah.

Respondent: If you've got to drive half an hour, like to drive to Lake [x] I love to do, but I'd only do it three or four times a year because you've actually got to, it takes half a day to do it. Where if I was sitting at home now and I looked outside and I thought I'd go for a walk for half an hour...

Interviewer: You can just go.

Respondent: ... I can do with... I have the... I mean sometimes I can work from home, especially in the winter if it's... if the roads are really bad and it's actually quite nice in the middle of the day if I'm working from home to think oh I can just go for half an hour walk and come back and keep working, which you can't do in an office environment like this because you... normally I'm dressed in [laughter] office attire, so.

Interviewer: Yeah. Absolutely. And if you wanted to be more active, would it help to be... to have more destinations within easy walking or riding distance?

Respondent: Yes it would, yeah. Just to give more option I guess, variety…

Interviewer: Yeah. OK. Neighbourhood design. This is where we look at the built or the non natural features of your environment...

Respondent: Yes.

Interviewer: ... manmade or whatever we want to call them. What sorts of ... if I asked you about these built features, what sorts of things would you tell me about?

Respondent: That are there now?

Interviewer: Yes. Where you live.

Respondent: Where I live. Well there's not really much at all. Like I said the lightings minimal, there are no halls or no community meeting places at all, except for the local hotel which is not an appropriate place.

Interviewer: And we've said about the walkability because of the roads and the gravel roads...

Respondent: Yes. So there really isn't anything. There's no tennis courts, there's no...

Interviewer: Despite the fact that you're actually not very far from your, what do you call it, what would you call it, your village or...

Respondent: There's a shop and a hotel, that's it…

Interviewer: Yeah.

Respondent: There's not many people live at [town], it's a very small community, so yeah.

Interviewer: OK. So do the built or non natural features in your area influence whether you're physically active or not?

Respondent: I guess the lack of would, yes.

Interviewer: Yes. And the fact is that you've found other alternatives, but it's sort of like you still wouldn't sort of go walking into [town] or along the road or wherever...

Respondent: No, 'cause there's nothing, nothing there to go to anyway.

Interviewer: Uh-huh. OK. And if you wanted to be more active, are there built features in your area that would help? And if you want to just extend it to [town] and more of the [rural region] 'cause you know those areas.

Respondent: Uh-huh. I mean some of the other towns I think a lot better footpaths.

Interviewer: Uh-huh.

Respondent: Uh-huh. If there were some little walking tracks or paths somewhere it would be really good.

Interviewer: Uh-huh. It just extends the options.

Respondent: And with... and just with the halls and stuff set up for activities, so to make it comf... a comfortable environment to go and feel like, yeah. And I mean even the gym at [town] is great but it's limited. And [town] doesn't have anything like that at all. And the other thing is... one of them is the instructors. We don't have anyone in our area that could actually take a class of something at all. So expense wise, to get someone up to do that's really expensive, and it's not regular. So that would be a big thing that would...

Interviewer: And the other thing of not having an instructor is you can't get the full benefit.

Respondent: No.

Interviewer: And...

Respondent: If there was someone that did a... I mean Zumba's really popular these days, if someone did a class every Wednesday night at [town] at the hall then I would go. But we don't have that at all. And I think a lot of other people would too.

Interviewer: Uh-huh. Certainly what you're talking about's very much coming out when I'm talking to women who live in rural areas and that's all of the areas.

Respondent: The opportunity to do that. And a lot of people need to do the group stuff. If they had a dance class or an aerobics class two nights a week they would do, but they would never go for a walk by themselves or do what I do.

Interviewer: No. Uh-huh.

Respondent: So it's really good to be able to offer different things for different people.

Interviewer: And people would do it for different reasons that some women will say that I've talked to will say it's not only an opportunity for another option for activity, but it's a sense of doing it in your community.

Respondent: Social.

Interviewer: Uh-huh.

Respondent: Well I actually like to exercise alone. Like I prefer to go for a bushwalk on my own...

Interviewer: Uh-huh. 'Cause you can do...

Respondent: ... or with my husband.

Interviewer: ... what you want when you want to, how you want to do it.

Respondent: That's right.

Interviewer: Uh-huh.

Respondent: But I still enjoy team sport and I still enjoy like an aerobic type class and I would do as an extra, just for some variety.

Interviewer: Uh-huh. That's good. OK. Now let's look at the other side of it, which is the natural features of your environment or the aesthetic qualities. So if I were to ask you about the aesthetic qualities or the attractiveness of your area, what sorts of things would you tell me about?

Respondent: OK. Well I... I mean I love the outdoors, so I love the natural bush and...

Interviewer: You've got heaps of bush.

Respondent: ... so for me. Uh-huh. So for me I love the mountains, the lakes, the... so I mean everything I've got there is what I love, yeah. I'd much rather just head off out the door and go for a bushwalk than what I would walk along a path in the city that is set up that some people like that sort of thing. But I prefer the natural, yeah.

Interviewer: Uh-huh. And do you think your local environment is aesthetically pleasing and attractive near your home?

Respondent: Yes I do.

Interviewer: Absolutely. And do the aesthetics of your area influence whether you're physically active or not?

Respondent: Definitely.

Interviewer: Uh-huh. And if you... and I guess the other side of it is OK, we've looked at the beauty, but there's also the rubbish and litter and graffiti is there, is that an issue?

Respondent: Not really. Not where I go, no. No. I mean they do get it around the lakes sometimes, but I wouldn't say it was an issue for me.

Interviewer: OK.

Respondent: No.

Interviewer: OK. And if you wanted to be more active, are there features of your area that might help if they were more aesthetically pleasing or attractive? The natural part of it.

Respondent: Probably not for me at the moment. No.

Interviewer: 'Cause it's mainly the non natural part.

Respondent: I quite like it being... I like it being isolated a little bit because I've... I mean I've travelled quite a bit and a lot of the sort of scenic places around the world now are getting so popular that there's too many people there. Where I like to be the only one out there, and that's, for me that's what actually attracts me to get out and do it.

Interviewer: Uh-huh.

Respondent: Where if it was a... I mean even Lake [x], I love the walks there but I actually like the longer walks, so once you get the first 20 minutes out of the park you know you're only going to run into a couple of people. But you do the smaller walks and having to pass someone every two minutes, and the overland track is a good example of that. I mean it's a beautiful

track but it doesn't really do much for me because it's all boardwalk now and just seem too many people do it.

Interviewer: Uh-huh. OK. Now the last area is safety. If I were to ask you about safety in your area, what sorts of things would you tell me about? You've talked a little bit about traffic.

Respondent: Uh-huh. On the road is traffic is number one obviously. The other one is places that I'd go... going on my own is an issue about making sure I let someone know where I am, carrying a mobile phone, but our service area is very limited, so that's another safety issue for us. So it's really making sure that... well it's usually my husband or if he's not there someone else knows where I'm going and if I'm not back at a certain time. Because a lot of places I walk we go through locked gates, other people don't have access to those areas and wouldn't even know. 'Cause I tend to just wander without... I mean I know the area well where someone probably wouldn't find me. So that's an issue. The lighting again is a real safety issue of a night.

Interviewer: Uh-huh.

Respondent: Safety as in you might hurt yourself. I wouldn't say I was... I'd be worried about someone picking you up or something, but again you do have to keep everything in mind. So I wouldn't go out for a walk up the road in the dark, I just wouldn't do it.

Interviewer: Uh-huh. So does personal safety influence whether you're active or not? Physically active.

Respondent: Uh-huh, sometimes.

Interviewer: Uh-huh. Particularly when it's sort of dark and yeah. OK. Does road safety influence whether you're physically active or not?

Respondent: Uh-huh.

Interviewer: Uh-huh. And if you wanted to be more active, are there personal or road safety issues that if they were addressed might help you be more active?

Respondent: Stop the log trucks. Although they haven't stopped me to date, but the lighting, yeah, the lighting is one. It's mainly the same things I think.

Interviewer: Uh-huh. OK. Well so...

Respondent: I know we have addressed... a few years ago we lobbied the council to get road signs up to slow the traffic through [town] which we were successful doing and they did the same at [town] 'cause that was a real issue.

Interviewer: OK. yes, 'cause it's...

Respondent: Just cars just flying through flat out.

Interviewer: Yeah, it's slowing them down isn't it?

Respondent: Which they still do of a night or whatever, but it has worked to a certain degree.

Interviewer: So before we finish up, is there anything else you'd like to tell me about about where you live, your environment or your physical activity that we haven't covered today that you thinks important…?

Respondent: Well I mean you've probably gathered that I keep quite active.

Interviewer: Yeah...

Respondent: That's just because I love to, the way it makes you feel. Look there are opportunities probably up in my area. Like I said the [x] Trail does go through the property up there which is now the [x], whatever they call themselves actually manage it. So the [x] Trail is something that, I mean I only had this conversation I think earlier this week about how they're not putting much money into that now. That could actually be a good opportunity for people to use our area more, 'cause you can actually do small parts of the [x] Trail.

Interviewer: OK So...

Respondent: And some of it actually is quite good, like it's away from the traffic.

Interviewer: OK.

Respondent: But there's still good roads that, yeah.

Interviewer: So it's a link... it's a trail; tell me a bit more about it. It's a trail...

Respondent: [x] Trail is [city] to [town].

Interviewer: Yes.

Respondent: And you can walk it, horseback ride or cycle.

Interviewer: Oh OK.

Respondent: So you can do the three. It crosses a lot of private land, so there's agreements with land owners about access and gates and all the rest of it. But I think that's where some of the issues are now that some of it's not insured for people to use it. And the upkeep is huge because it actually goes up the tiers and up to [town] and to [town] and it comes through [town] and uses a lot of back roads, and there's a book that people can follow. So a great initiative, but it takes money and people and time to actually upkeep it. And...

Interviewer: So it has actually been completed?

Respondent: Oh yes.

Interviewer: It was completed. But it's about the upkeep of it.

Respondent: It's about the upkeep of it and making sure that...

Interviewer: So in this area...

Respondent: ... making sure it's sign posted well and people know exactly where to go when...

Interviewer: That's always the problem.

Respondent: Yeah. And all the rest of it…

Interviewer: So in this area, in terms of upkeep, you were saying one of the things is better signage, other issues around its upkeep of accessibility and...

Respondent: I think immediately where we are it's quite good because of the fact that it is on quite well made roads. But I have heard people say when you're crossing in other people's properties it's keeping the tracks up to date, making sure that people actually have keys to the right gates and...

Interviewer: Often hard to find your way.

Respondent: ... don't take a wrong turn.

Interviewer: Oh of course.

Respondent: All those things.

Interviewer: Yeah...

Respondent: And I know that people on horseback and even the cyclists a lot of times they'll have a back up vehicle, so when they get to their destination that evening they've got someone to set up their tent or got their food or, yeah. But it's a great initiative.

Interviewer: It is.

Respondent: For those sort of areas.

Interviewer: Yep. OK. No, that'd be good. Well even using some of it more locally.

Respondent: That's right, yeah. So you can have a short part of it, that you have a day and people can do a walk together and things like that. So that's sort of something. I can't think of anything else. Maybe, like I said around the structured classes, ways of actually getting people up here that can actually do classes, whether we train local people or whether we can get people to come up. It's all about money really, funding and travel time for those people.

Interviewer: OK.

Respondent: I think I've covered most else.

Interviewer: Thank you.

Respondent: What I haven't I'm sure [colleague] will.

Interviewer: [Laughs] yeah she will. Thank you very much for that. That's great.

Interviewer: So I thought that we might start with that, talking a bit about the place in which you live which is [town]. So can you tell me a bit about what it's like living around where you do and your likes and dislikes?

Respondent: What is it like living there? It's cold in winter, I stay inside I don't go out. It's community, people know each other so there's that sense when you go to the post office that you see people that you know. So the sense of community was really what attracted us to living there as opposed to a big urban centre.

Interviewer: And do you live in [town] itself?

Respondent: I live in town, I've got next door neighbours.

Interviewer: OK so is it a house block, house and block?

Respondent: Yes.

Interviewer: Or very large block or fairly standard.

Respondent: There's a sheep paddock up the top so it's a decent size.

Interviewer: Yes so it's an acre, two acres?

Respondent: Oh it's probably three actually.

Interviewer: Three? OK. And how long have you been living here?

Respondent: We've been here three years.

Interviewer: And your reasons for living there?

Respondent: Our reasons for living there, we moved from London to Tasmania...

Interviewer: Really?

Respondent: I'm originally from Victoria, had been in the U.K fifteen years, had met my husband, had the two girls who are about two and a half and four and we decided that the U.K couldn't offer what we wanted for the girls, we wanted a country upbringing, ability to go outside, outdoors and we felt that Australia offered that more.

Interviewer: And that England's so densely populated.

Respondent: And so extortionally expensive.

Interviewer: Very expensive, exactly.

Respondent: That you've got that many in your house and still be living in a flat. So essentially I came from a farm, Western Victoria farming so essentially I wanted them to have that which I had had.

Interviewer: And OK so you're nearest town is [town], so you're living in that. 'Cause it just asks the distance to your nearest town so you consider [town] as that. So this research has mainly been done with women in urban areas but people often refer to the term, local neighbourhood and I know we've already started talking about this and your reasons why and I was

interested in understanding if the term neighbourhood had relevance for you, living rurally or whether it...?

Respondent: I think it does. I think it would have more relevance than living in a city for us personally. We get on very well with our neighbours in terms of helping each other. I can't put up pictures so [x] will put pictures up for me, he'll look after my dog or I'll bake, if I'm baking extra on a weekend then I will go over and give it to them. So in terms of our next door neighbours we do work very closely. The lady across the road she will look after our house if we're away so we do have that sense of community. So not really, that's personal as opposed to anything else.

Interviewer: No that's the other question in terms of about the question about community. So there are two different terms, what's most relevant to you? Are they different neighbourhood and community?

Respondent: I see neighbourhood, I would possibly have a closer relationship with my neighbourhood, with my neighbours, something which I would perhaps make a decision in terms of forming that relationship. Whereas in terms of my community that's something which I feel that I have to be a part of because it is my community.

Interviewer: OK.

Respondent: Does that make sense?

Interviewer: Yeah no it does. So if you needed to put a distance around neighbourhood what sort of distance would you put? Because it sounds like it's close to neighbours.

Respondent: Close and immediate, that would be me. Bordering or two down the road.

Interviewer: So that would be a matter of one hundred or so metres.

Respondent: Yes. You're either in the circle or you're out of the circle.

Interviewer: That's fine, and community?

Respondent: Community, I think community would for me, like I just did a phone call now responding to a party. I think community where I live is very much socio-economic. Your community you slot into whatever, whether you have a large farm or a small farm, whether you are educated or whether you earn a good wage. If you are from like a working class background then that would be a different community. That's what I find, there may be some cross over but it's not that great.

Interviewer: OK. And how does it work with, often people who have children talk about the school community, your community and friends at school as well.

Respondent: Well you see this is why perhaps we're great for the research. We started, look we actually come into town for school, in terms our needs weren't being met locally...

Interviewer: Well people do, there's been a few responses to say that...

Respondent: So we commute, people think we've got two heads so essentially I was going into town most days, town being [city] so we just... the best decision we ever made. For us the issues were there were all behavioural issues and you know they weren't being addressed...

Interviewer: Yeah that's fair enough.

Respondent: So I don't have that school community.

Interviewer: It's interesting your description of community to and I've heard that in other ways about the [region]...

Respondent: Oh how interesting. The old money, the not old money, the workers, the employers...

Interviewer: Yes because we've interviewed people in [town] and the whole town from [town].

Respondent: Oh yeah, there you go.

Interviewer: Yes, interesting.

Respondent: Very interesting, the type of person that you would...

Interviewer: So if you need to put distance around community, what distance would you put around community?

Respondent: For me I guess it would be for [town] we would go to [town], that would be our community and I guess [town] wouldn't. I know people from [town] but I wouldn't go into any of their community activities.

Interviewer: They say that they don't come over either.

Respondent: So I'm sure there's something happened about 150 years ago that's... so for us it would be to [town] and I guess a couple of reasons for that is mum's with young children, I guess it's the show, the [town] district show, that sort of is a lynch pinch between [town] and [town] and I guess the church as well. That sort of church community.

Interviewer: Yes. No remind me, how far is [town] from...?

Respondent: Oh it's ten minutes. Just down the road, that's where you go for your grocery shop if you don't want to go to [town].

Interviewer: So it's only about ten – 15 kilometres?

Respondent: If that.

Interviewer: Alright now just to start talking about physical activity. Now we're really interested in obviously a whole range of how people do their activity and we know that changes in your life...

Respondent: We're not in a good period now...

Interviewer: And winter doesn't help either.

Respondent: No.

Interviewer: So what does the term physical activity mean for you?

Respondent: What it would mean for me would be playing tennis once a week or playing netball once a week. Would be involved in a regular routine sporting activity, however that doesn't happen. That would be an ideal life and that's just where I'm at, mother of two children and commuting quite a lot.

Interviewer: Yes, you're very busy.

Respondent: And I guess, it's all time it's all related to time so I think, I'll drop the girls off at school and I'll walk to [workplace]. Then I go, I haven't got time I have to be there by nine, so it doesn't...

Interviewer: It gets a bit relentless...

Respondent: Oh it's terrible.

Interviewer: Do you consider yourself physically active?

Respondent: No. Not anymore.

Interviewer: Would you like to tell me why?

Respondent: I think it's just because I have too much on my plate. Because I have other priorities and for me it's really just a question of getting home, getting dinner on the table and doing reading and doing all those other things. So I think it's being a mother and I guess not only that, a working mother, other mum's will drop their kids off and go play badminton for the morning, whereas I will drop the girls off and go up to [workplace].

Interviewer: And also with study that's another additional factor, is that you really are trying to study every moment and that's always something to rush...

Respondent: Every minute that does...

Interviewer: You know work at least starts and ends at the same time.

Respondent: You're right, study doesn't. It's protected time but actually it doesn't allow for any other time.

Interviewer: I know when I studied and worked, it catches you in the end you go 'I can't do anything else'. OK and where does physical activity fit into your life? Is it priority, a high priority, no interest?

Respondent: Look I would walk from [suburb] to work which is [x] Street, up the top. I would walk there twice a week and that's a concerted effort, that's a choice that I make I could park but look it's just so hard to fit in. That's where I sit. We've just come back from holidays and we went and got a walking machine and a rowing machine with my husband and I thought why are we doing that? That's...

Interviewer: OK. Now I wanted to talk a bit more about physical activity and in our research we think about physical activity as any activity that lasts for at least ten minutes, that's the evidence of a health benefit. So that means it causes the body to work a bit harder, you might feel a bit warmer, you might have a bit of huffing and puffing later, so thinking about that type of physical activity over the last two weeks, and I'll just go over different parts of your life not to separate your life out...

Respondent: No that's OK.

Interviewer: Just to cover things. Now you're saying that you are working part time, so thinking about that and you just started talking about walking to work, in the past two weeks have you been active at all at work for at least ten minutes at a time?

Respondent: At work?

Interviewer: Yep or getting to work.

Respondent: Getting to work is a 20 minute walk there and a 20 minute walk back. And then I would walk from there up to the [hospital] and walk around there for two hours and be exhausted just meeting and greeting, so the walk there speeds you up, the walk back. Work? [study] nothing. What did I do on the weekend? I can't think? Garden?

Interviewer: That's OK. We'll just look at work.

Respondent: Work? No that would be it.

Interviewer: And then I'll get onto the other parts of being at home and stuff. Can you tell me about what you did? So you've said that you've walked to work and then you walked from work to the [workplace]?

Respondent: Yes.

Interviewer: OK. Now looking at those two activities what would you consider the intensity of them? Steady, moderate or vigorous?

Respondent: I walk fast.

Interviewer: So...

Respondent: That's the only opportunity, so I would take my coat off, take my scarf off...

Interviewer: Yes you do that don't you? So pretty vigorous?

Respondent: And wear flat shoes.

Interviewer: And the duration? How long would that be for?

Respondent: So it's 20 minutes getting to work, 20 minutes going home or back to the car and look to the [hospital] it's probably not even quite ten minutes, it would push ten minutes.

Interviewer: And how often would you do that?

Respondent: The [hospital] visit?

Interviewer: And the...

Respondent: The work. So twice a week...

Interviewer: Both activities.

Respondent: I would walk to work on both days, Monday and a Tuesday and the [hospital] probably once a week.

Interviewer: OK. And is that during the day?

Respondent: During the day.

Interviewer: And is this what you would normally do?

Respondent: Yes.

Interviewer: And does it change depending on the season or the time of year?

Respondent: No, I put on a bigger coat and take an umbrella.

Interviewer: If you wanted to, is there anything that would have helped you be more active at work? Like more time or some of the activities...

Respondent: Probably not because it's a desk job and with the odd meeting so no.

Interviewer: In the past two weeks have you walked or cycled for at least ten minutes at a time to get to or from places?

Respondent: No...

Interviewer: Other than what we were talking about getting to work?

Respondent: No, nothing more.

Interviewer: If not, could you let me know why?

Respondent: Time, deadlines.

Interviewer: Yes. And if you'd wanted to, is there anything that would have helped you be more active? Either walking or cycling, is cycling relevant to you?

Respondent: Cycling isn't relevant for me at all. In my 20s it was but too many hills in Tasmania. No, it's shocking. Today I did think about walking, not because it was a physical activity study but I was thinking I could slot in that but then the getting back to [university], it's the time.

Interviewer: Yes, the time.

Respondent: For me it's time.

Interviewer: Yep to get back to [suburb].

Respondent: Yes, well to get up to school and to get in the car and go home, oh that's crazy.

Interviewer: Yes there's a limit isn't there? In the past two weeks have you been active around the house or yard? Either inside or outside your home?

Respondent: I don't stop all day. So I don't sit down, three floors I'll go up and down the floors. There's no lift.

Interviewer: This is at home?

Respondent: At home. So that's up and down, we've just come back from the U.K. so that's not helping is it? The last two weeks...

Interviewer: Just pick the few weeks that seem typical of you.

Respondent: And so we would probably go outside for a walk, take the dog for a walk that could be 15 – 20 minutes, sometimes fast sometimes slow depending on who is going with me and our washing line's up the top of a hill so that's quite a walk as well.

Interviewer: OK and I guess there's housework and all of that sort of stuff as well?

Respondent: Oh yeah.

Interviewer: So that's three activities you were talking about, walking the dog...

Respondent: I just think general daily, up and down and look sometimes I might at home run up and take them by two because I don't exercise and then literally going out for a walk or just going around the garden or going up and hang the washing out.

Interviewer: OK so thinking about those activities inside and out and the dog, what would you say the intensity of them being? Steady, moderate or vigorous?

Respondent: Probably, the dog I'd say moderate because I will try and run the last bit out the house and going to the washing look I might try and run it up the hill so that would be steady to moderate depending on how I was feeling.

Interviewer: And how long would you have done the activities for?

Respondent: Still takes about 20 minutes, I was thinking I hope the RSPCA don't ask me, I probably should go for an hour. 20 minutes the ongoing, is on going in the house up and down and the washing, I certainly could do three loads.

Interviewer: So that would be how often?

Respondent: I would just do that on a Saturday.

Interviewer: And how often for the dog?

Respondent: Two days, Saturday and Sunday.

Interviewer: And how often for the up and down?

Respondent: Saturday, Sunday.

Interviewer: And so when you did that your activities it was mainly the weekend?

Respondent: Yes.

Interviewer: Yes and why did you do it at that time?

Respondent: Because I wasn't home Monday through Friday. It's hysterical.

Interviewer: I know it's about how busy our lives are.

Respondent: Which is terrible.

Interviewer: And there are times in your life when you are far more busy when after all you've got kids settling into school...

Respondent: I do dream of retirement, I swear it would be lovely.

Interviewer: And who would you have done those activities with?

Respondent: Children, dog or husband, the family thing. Washing, myself and the girls if they want to come up and the up and down inside, me.

Interviewer: And is this what you'd usually do?

Respondent: Yes.

Interviewer: And does it change depending on the season or the time of the year?

Respondent: No.

Interviewer: If you'd wanted to, is there anything that would have helped you be more active around the home inside or outside?

Respondent: No not really. Just a different time of my life I would be doing different things.

Interviewer: Now given all of your busy life, the last area is do you have... have you been physically active for at least ten minutes during your leisure or spare time?

Respondent: That's interesting. No. My leisure or my spare time would be sitting down with my feet up reading the paper or something.

Interviewer: Sounds like a really good idea.

Respondent: Just trying to think what would I do? And it's about 1.30 on a Saturday and Sunday afternoon, time out. Yep so that would be me.

Interviewer: That's the kind of time women will talk about is that they are busy enough in the rest of their life. And if you'd wanted to, is there anything that would have helped you be more active in your leisure or spare time?

Respondent: No.

Interviewer: Like more time or...

Respondent: Yeah, just the standard ones. I think, no. In terms of access, do you get to access?

Interviewer: We are. We're just about to get on to environment now.

Respondent: OK. However if it's the lack of availability as well I think. If there was a netball court down the road that did it, two minutes down the road then I would probably play netball one night a week and if there was a tennis club or just people doing social tennis on a Monday night I would block that out in my diary and childcare wouldn't be an issue. I'd be home and it would be two minutes and I'd be back home again.

Interviewer: And availability and accessibility were the first ones we were going to talk about, so that's great. OK. We've talked a bit about where you live and physical activity, now I want to talk about the physical environments that you work and live and play in. And when we talk about physical environments we're talking about all the physical things that surround you so that might be the roads, the trees, the houses, shops, traffic? And some of these things are things that people in urban areas have said are relevant to their activities so that's why I want to ask you about all areas.

Respondent: OK as a comparison.

Interviewer: Now first of all can you tell me a bit about how easy or difficult it is to be physically active in your area?

Respondent: I guess in terms of what my preference would be to be physically active then that is difficult. In terms of accessing, it's not there. I would have to come into [city].

Interviewer: And why? What makes it difficult?

Respondent: I guess it's the lack of numbers.

Interviewer: Lack of activities available.

Respondent: Yep.

Interviewer: Now availability and accessibility in places to be active. So if I were to ask you about places to be active in your area, what sorts of things would you tell me about? Now that could be recreation facilities or if there are walking tracks and parks...

Respondent: So for us, what we would do, we would go down with the girls so this is more about them than us, would be to the tennis court to have a hit of tennis. That doesn't exert me. We would go to the playground, or if we wanted to go for a proper walk we would head up to Mount [x].

Interviewer: OK.

Respondent: Mount [x] is our get in the car to go somewhere to have an outing, to lets all go for a walk.

Interviewer: How long would it take you to drive over there?

Respondent: 30 minutes on the dot, so that's where we would do that.

Interviewer: And other than that you don't really have sporting things available from the sound of it?

Respondent: No.

Interviewer: And parks and walking tracks? No?

Respondent: No. We would walk around town in terms of walking the dog, maybe go up the back way and go through a little lane...

Interviewer: It's not bad that way is it?

Respondent: Yeah so it's easy, you can go off the main road do the back streets and you can vary it.

Interviewer: And access, are places convenient? Are they easy to access? OK, well you were saying that they're not available and so it's not... we haven't even got to the cost or opening hours, they're just not there.

Respondent: No. And the other is a gym. That's what I... I think hospital [town] has gone one at the school...

Interviewer: Yes they do.

Respondent: But there's so many things like yes no, get the key from this person, and you don't. So accessibility in terms of that, I didn't even go there, I remember asking and I just thought 'Oh this is all too much hard work'.

Interviewer: Because I think, I've talked to people they've said it's not open on the weekends which is a problem and it's open part of the evening that...

Respondent: But even then there's conditions and actually that's too difficult.

Interviewer: Yes because I was talking to someone that doesn't use it because of that either.

Respondent: I thought that would be quite handy and then you ask you around and everyone just goes yes but.

Interviewer: So does having or in this case not having places to be active influence whether you're active or not?

Respondent: I think it does. As I said if there was a netball match happening every Thursday night at seven thirty I would block that out. Definitely and I don't have to organise husbands home, that's alright, I can feed people. Not having to put in that hour, a friend from [town], she'll come into a gym in [city] and she's got four children, has to feed everyone, he's a dairy farmer and that type of commitment, you just think that has to be a number one priority that can't be done on the run.

Interviewer: Yeah that's a lot of work. So...

Respondent: And I guess for me as well when stuffs available in [town] it tends to be during the week...

Interviewer: Yes when you're not there.

Respondent: When I'm not there so I can't tap into that.

Interviewer: OK so you've told me about what things might help if they were available or more accessible to be more active. Is there any other things?

Respondent: No, let me just think. Look I think as well, I think that's very much having a structure and a framework in place. A model that could be adopted to each, as opposed to this adhoc, let's do this because this is the current fashion. So it needs to come from a strategic way of thinking and the community does well in trying to set these little bits and pieces but at the end of the day you think 'Let's start to finish'.

Interviewer: It feels very adhoc.

Respondent: It is adhoc.

Interviewer: Now destinations. If I were to ask you about places that you could walk to from your home what sorts of things would you tell me about?

Respondent: What I would see?

Interviewer: Yes, like shops and schools and recreation facilities.

Respondent: Across the road I'd see the park and then I would go down by the river onto the back streets and then I'd go down to an old ruin, that's half falling down.

Interviewer: Can you get down to the river?

Respondent: I wouldn't take my lot, no. Would I? No I've never been down there. I can't swim, it just wouldn't work.

Interviewer: No, there's no track along the river?

Respondent: No so we would go on the road and then we would just make our way up, go past the church, have a look there, we might go in there past a friend's house see if she's home.

Interviewer: So does having or not having destinations influence whether your active or not?

Respondent: No not for us. We have a set path kind of thing.

Interviewer: If you wanted to be more active would it help if there were more destinations within easy walking distance?

Respondent: I don't think for us, I don't think the destination is the reason that we would do that. We would go out to be outside, get exercise and engage with each other I think as opposed to a destination.

Interviewer: Sometimes it's about we do things for arranged purposes...

Respondent: Hourly quality time.

Interviewer: Yes. OK looking at the built environment now. Neighbourhood design. If I were to ask you about the built or the man made features of your area what sorts of things would you tell me about? And this is about the footpaths, lighting, roads and those sorts of things.

Respondent: OK, not the best. Footpaths not the best, you walk on a road generally.

Interviewer: OK so that might be... are there any footpaths in [town]?

Respondent: There are some. There are footpaths in [town] but I'm just thinking of a particular route that we would take and we would have to be quite careful and the footpaths are being renovated.

Interviewer: So there not consistent?

Respondent: No consistent.

Interviewer: And not necessarily in good quality order? So if you for instance had a pram or something it would...

Respondent: If we were walking to the park then we have to sort of be careful because...

Interviewer: So the walk that you would do would be on some areas that wouldn't have footpaths? So would they have bitumen roads with just gravel...?

Respondent: The grass...

Interviewer: Grass area. OK. And the other one is in terms of walking often having the ability to connect with other streets or roads is handy too, rather than just going along for instance the main road and back again. Are you able to do that in your area?

Respondent: Yes, so we go back streets, left right, walking in a straight line would be, no.

Interviewer: Do the built features in your area influence whether you're physically active or not?

Respondent: I don't think so.

Interviewer: You found your own marathon circuit. And if you wanted to be more active are there built features in your area that would help?

Respondent: Currently or potentially?

Interviewer: Potentially.

Respondent: That would help? I don't think. For me in a rural community that's not what I'm there for. That's... if my rubber boots aren't going to get muddy

then I may as well go live in the city, do you know what I mean? That said though, that's not a priority for me.

Interviewer: OK that's fine. There's enough streets that connect. Some areas you just can't get down to other areas or can't get to things.

Respondent: I don't feel that I'm limited.

Interviewer: [town]'s not so bad for that.

Respondent: No. I don't feel I am.

Interviewer: Now the aesthetics, the other side of it the natural environment. If I were to ask you about the aesthetic qualities or the attractiveness of your area, what sorts of things would you tell me about?

Respondent: [x], my favourite bit is going up a dirt track having to open the farmer's gate. It's not even the farmers, it's probably a legal gate and just walking [x]. There's a little dam and I'm like in the country, walking up a hill and then coming down the hill. Beautiful.

Interviewer: It's very lovely.

Respondent: It is. So that for me and the other aesthetic is the old buildings is for us. That would be us.

Interviewer: And of course you've talked about Mount [x] which is beautiful for its natural environment as well, the trees and things. Do you think your local environment is aesthetically pleasing or attractive near your home?

Respondent: For me? Yes.

Interviewer: Any issues about the other side of it which is rubbish and litter and graffiti?

Respondent: No graffiti. Rubbish? People would pick it up. You pick it up.

Interviewer: That's a different thing.

Respondent: So if we have rubbish in the front of our house, we pick it up. Or you phone the council and they would... our neighbour she rings them up if there's weeds growing too high and they'll spray them.

Interviewer: So that's not really an issue is it?

Respondent: No.

Interviewer: And do the aesthetics of your area influence whether you are physically active or not?

Respondent: No.

Interviewer: It's always a difficult question because particularly in rural areas, because remember this comes from urban areas where you may have a pleasant environment that you live or you may not but generally people who've moved into rural areas do it because of the beautiful environment...

Respondent: Where as I am you just do it. That's interesting isn't it? And the other interesting thing is people come to our environment because of the aesthetics to visit.

Interviewer: That's right, I'm sure yours was...

Respondent: Initially yes definitely. It had to be pretty, it wasn't going to be some rough looking...

Interviewer: OK and if you wanted to be more active are there features of your area that might help if they were more aesthetically pleasing?

Respondent: I don't think so. I think it's as it is.

Interviewer: One last area is about safety. If I were to ask you about safety in your area, what sorts of things would you tell me about?

Respondent: I feel extremely safe. That's not an issue that doesn't cross my mind.

Interviewer: So that's personally safe. And road traffic safety is the other one?

Respondent: I don't, no I don't think. Look I know it's a through town but that's never an issue and people tend to slow down and I don't ever feel unsafe.

Interviewer: It's a nice, the other thing I remember about [town] to, it's never ideal having through traffic but given the design of the town in which the main roads actually quite wide and gentle curves...

Respondent: That's slows the down.

Interviewer: That slows them down and there are footpaths on that... and then you've also got room to get off into the side...

Respondent: So look even the trucks you don't...

Interviewer: So does personal safety influence whether you're physically active or not?

Respondent: I don't think it impacts on us. It's not a factor we have to take into consideration.

Interviewer: Sometimes people say 'Going for a walk at night is pretty tricky just because lighting and things like that'.

Respondent: Look we would take a torch, we would go to the [pub] for dinner and so we're OK with that, yeah that's fine.

Interviewer: That's part of being a...

Respondent: The nice thing is you can skip home and you can go on the road and you feel safe, you know that it's OK.

Interviewer: And likewise does road safety influence whether your physically active or not?

Respondent: I don't think that impacts on us, no.

Interviewer: And if you wanted to be more active are there any personal or road safety issues that if they're addressed might help?

Respondent: No not applicable again.

Interviewer: And just before we finish up is there anything else you'd like to tell me that we haven't covered today? Living in [town] or physical activity or the physical environment?

Respondent: I guess I think that in terms of a community that there needs to be some type of government approach, that's what I think in terms of minimum standards. And perhaps for the community or the council to have some type of guidance that this is...

Interviewer: Rather than have it come in from the community...

Respondent: Yes, let's just build a new playground but then we'll fence it but then the fence isn't right so then someone else has to fix it, you know it's just

this... someone who loves processes, just to be a bit more structured. That would be my view...

Interviewer: Yes, it's a common view that's coming out of [region] that people saying the role of council would be good if they were a bit more...

Respondent: Yeah there needs to be funding in the job. That would be my suggestion. Look it needs to be... the ad-hoc, that winds me up and the adhocness generally comes from well meaning people in the community trying to set these things up but it needs to be a bit more focused, that would be my only feedback.

Interviewer: Thank you.

Respondent: Pleasure.

Interviewer: OK. So, I thought we might start with just talking about the place where you live, a bit first.

Respondent: Yep.

Interviewer: So can you tell me a bit about what it's like to live around [town], and your likes and your dislikes. You're actually in [town] itself?

Respondent: No, no. I'm on a property about 15 k north, so.

Interviewer: Oh, OK.

Respondent: Yeah. Yep. Yep. So. Yeah, I mean, I've, I guess I haven't been fulltime at home for a little while. I sort of lived in [city] while I was doing uni.

Interviewer: Yep, yep.

Respondent: Sort of, you know, on and off at home, in town. I moved home, will be home permanently now. Probably from, as of about a month ago, but, yeah. I mean, I've sort of always, had home as my base, so.

Interviewer: Yep. Yep.

Respondent: But yeah. It's, I, I love it, living up there. It's, yeah. I had my stint in town and it was just, yeah, not quite, I mean, I didn't want to settle there, so I'd always want to be in a rural area.

Interviewer: Yeah.

Respondent: Just space, and not, you know, just being able to do your own thing, and not being crammed in with hundreds of other people it's, yeah. And just...

Interviewer: It's beautiful space out there..

Respondent: Yeah, having your animals, and your, yeah.

Interviewer: Yeah.

Respondent: No, it's, yeah. It's really...

Interviewer: Oh, OK. Yeah. Yeah. So, can you tell me a bit about the, the property you live on? You said...

Respondent: Yep. Yes.

Interviewer: ... it was on land?

Respondent: Yes. It's a sheep and cattle farm. It's been, in the family for about five generations. So. Yeah, there's a lot of history there.

It's about six hundred metres past sea level.

Interviewer: How big is it?

Respondent: There's it's, yeah, very quiet. There's sort of a few different blocks, over land that make up the farm. So we've got the main farm in [town]and a block in the [town], near [town] and the [town] and then [town] and then [town] as well. So, do, you know, sort of spread out a fair bit. But...

Interviewer: So, a bit of a guess of how much it might be?

Respondent: Land?

Interviewer: Yeah.

Respondent: Well, there's [x] acres altogether.

Interviewer: Wow.

Respondent: So, yeah, I mean...

Interviewer: Yes. OK. That's, that's a big one.

Respondent: I mean, it's sort of not, yeah, something I like to broadcast obviously. But yeah...

Interviewer: Yeah, yeah. Yeah.

Respondent: ... so it’s relatively large.

Interviewer: It gives us a bit of a sense. Because, what’s interesting is that, women often, you know, like, part of their activity is about where they actually live.

Respondent: Uh-huh. Uh-huh.

Interviewer: Or the place they live. Either, the work on a farm or whatever they’re doing. So, it’s good to know. And again, as I said, we’re interested in people who live in out of townships, so...

Respondent: Yep, yep. Yeah.

Interviewer: ... that’s why we asked.

Respondent: Yeah, so it’s...

Interviewer: And the length of time you’re living there?

Respondent: How, how long will I live there?

Interviewer: Yeah. No, how long have you lived there?

Respondent: Have I? Well, I guess I was brought up there, so...

Interviewer: OK, so...

Respondent: ... we've been full on 25 years.

Interviewer: So you were born there?

Respondent: Well, almost, yeah.

Interviewer: Almost?

Respondent: Born in [city].

Interviewer: Yep, yep. And then you went...

Respondent: Yeah.

Interviewer: ... there when you were very wee.

Respondent: Yes, yes. Pretty much. And yeah, as I say, sort of on and off through school. I was, I was at boarding school but came home for weekends. And uni, I was sort of in town but came home as well. Yeah. As I say, I moved home fulltime, yeah, about a month ago, so, yeah.

Interviewer: OK. And your reasons for living there? You really grew up there.

Respondent: I just enjoy... yeah. Yeah. It’s just, that’s where I'm from, I guess.

Interviewer: Yep. Yep.

Respondent: Yep.

Interviewer: And you, the distant to your nearest centre, that's [town]?

Respondent: Well, [town]. So it's about 15 K's.

Interviewer: About 15 K?

Respondent: Yep.

Interviewer: And in driving time, how long would it take you?

Respondent: Oh, ten minutes. Ten or 15 minutes. Yeah.

Interviewer: OK. Now, in research with women living in urban areas, OK. People often refer to their local neighbourhood. And I was interested in understanding what the term 'neighbourhood' might mean to women who live in rural areas and if it does have relevance or not.

Respondent: Yep.

Interviewer: So what does the word 'neighbourhood' mean to you?

Respondent: I mean, it conjures up pictures of, you know, neighbours where they're all living in the street, and...

Interviewer: Yeah, yeah.

Respondent: ... they're all friends, like, obviously it's clearly not overly relevant for us. But I think...

Interviewer: No.

Respondent: I mean, I guess it's just on a broader scale. You know, there's, you know, your neighbourhood would be our local farmers and the people that you, you know, associate with, but obviously on a much sort of larger area...

Interviewer: Yeah.

Respondent: ... than your typical sort of urban neighbourhood obviously. But, yeah. I mean, it's not something that I really considered, to be honest.

I don't really think about me as belonging to a neighbourhood. Belonging to an area, yes. Or a region, but, not really a neighbourhood. So.

Interviewer: So, if you needed to put, like, you're saying it's not particularly relevant, but if you needed put a sort of distance on that, how far would it be, when you're saying that, well, if it was anything, it would be the people around?

Respondent: Oh. I guess, I mean, I guess in other sense, a neighbour, you know, neighbourhood refers to your mates and your friendship group as well. And if that's the case, then it's sort of, [town], [town], [city], [region]. It's...

Interviewer: Yeah. It's much bigger.

Respondent: Yeah. So it’s sort of, it’s hard to put, hard to really quantify that I guess. I mean...

Interviewer: So that...

Respondent: Neighbourhood... I don’t know. Yeah.

Interviewer: OK. Well, I'll ask you then, what about the word ‘community’? Does that...

Respondent: Uh-huh.

Interviewer: ... what does that mean?

Respondent: Yeah.

Interviewer: Is that more relevant for you?

Respondent: Yeah, it would be.

Interviewer: Yep. OK.

Respondent: Yeah. Community probably. I mean, obviously [town] Township itself. And I guess the farms within a radius of, I don’t know, 20 to 25 k, out of [town]. Something like that sort of radius, I guess.

Yeah. I mean, once you get further away, you sort of get closer to [town], or you’re getting up to [region], where there’s centre, so you’ve got

[town], [town], or [town] so it’s, yeah, I guess a sort of a 20 k radius, I think. (Laughing).

Interviewer: And, what does, what would, does the word ‘community’ mean for you then?

Respondent: Yeah. Again, it’s sort of nothing I've, I've not, yeah, not really considered that, you know, in, I guess, absolute terms, what it means.

Interviewer: Yeah. What might it...

Respondent: I guess it’s...

Interviewer: If it’s more relevant to you, what might it sort of mean for you?

Respondent: I guess it’s, yeah, just sort of, bit of, the support network, I suppose...

Interviewer: Where your friends and family...

Respondent: ... is, is probably the best yeah, best way of describing it. It’s, you know, someone, you know, a group of people that you can rely on, I guess, if something goes wrong. You know. Like, if there’s a bushfire you, your community will come and help fight it. You know, that sort of stuff. So.

Interviewer: Yep. Yep. OK. That’s fine. Now, I’m going to talk a bit about physical activity. And what the term, and we’ve said, it’s a really broad thing. It’s really, you know, up to the individual, what they think it is for that. So, what does the term ‘physical activity’ mean to you?

Respondent: Just doing stuff, I guess. So, being busy, and you know, just, yeah.

Interviewer: Doing stuff in your life...

Respondent: Yeah, yeah.

Interviewer: Your day to day.

Respondent: Yep.

Interviewer: OK. And where does physical activity fit into your life? Is it high priority, not particularly interested?

Respondent: I guess, well, the nature of my work; I do a lot of it. I'm working for [company] at the moment...

Interviewer: Oh, OK.

Respondent: ... as a [role], so I've got a lot of, you know, walking paddocks and you know, cruising around with farmers and stuff. I'm always doing a lot of walking.

And then weekends, riding horses or I'm working the farm, or you know, lifting stuff or doing, you know, a variety of things. So, it’s pretty, it features, but it’s not a conscious decision for it to feature. It just happens. Yeah.

Interviewer: Yeah. Yep. Yep.

Respondent: So, it’s not that I think I've got to; better get 30 minutes a day, sort of. It’s not something that I ever think about, but, I guess I'm pretty, you know, relatively active, so.

Interviewer: OK. Now, in our research, we think about physical activity as any activity that lasts for at least ten minutes. Like, that’s the evidence for health benefit. You know, like, causing the body to work harder, or you, you’ll feel warmer, or your, your heart rate goes up, or breathing, a bit of huffing and puffing.

So, I just want to talk to you about different parts of your life. Not that we want to divide your life into parts, but it’s just handy that we don’t miss things.

Respondent: Yeah, sure.

Interviewer: So, thinking about this type of physical activity during the last two weeks, just as an indicator, now, in the past two weeks, have you been active at all at work for at least ten minutes of time?

Respondent: Yep.

Interviewer: You mentioned that.

Respondent: Absolutely.

Interviewer: So, can you tell me about what you did?

Respondent: I... walked a lot of paddocks, taking soil samples, so.

Interviewer: Oh, OK.

Respondent: Yeah, so...

Interviewer: OK.

Respondent: ... that was a lot, you know, you obviously walk the paddocks to get a feel for what's going on, and if the ground's hard, you've got to jump up and down a fair bit. So it's, yeah, it can be quite, you know, be a lot handy if I was about 20 kilos heavier.

Interviewer: Absolutely.

Respondent: Yeah. So that, that's sort of the main thing and...

Interviewer: Because you've got to actually drill in.

Respondent: Yeah, that's right. I helped in the depo for a while, moving bags and yeah, so it was, yeah. They were probably the main...

Interviewer: Bags of fertilizer?

Respondent: Yes. About 25 kilo bags that sort of.

Interviewer: Yeah. But that's hefty enough.

Respondent: Yeah. Oh, it's, yep. What else? Yeah, they're probably the main ones.

Interviewer: OK.

Respondent: Well, as far as work goes, anyway. Yeah.

Interviewer: Yep, yep. So, thinking about those two activities, just to get a bit more information from you. Intensity? Would you call those activities moderate, or vigorous, or steady?

Respondent: Oh, probably steady, I think.

Interviewer: OK.

Respondent: I mean, if you're walking, you know, you're walking paddocks at a sort of, you know...

Interviewer: What about the drilling stuff?

Respondent: ... a few kilometres square sort of thing. Yeah, I mean, that's, that's not too bad.

Interviewer: That's, that's not too bad.

Respondent: If, if the ground's hard, it's, you know, probably moderate. But, if it's, yeah, like it has been, it's not, it's just, it's probably more it's not intense exercise, but it's over a long period of time that you're walking, so. Each paddock can take you anywhere from...

Interviewer: Are you actually sort of working in the area you live now?

Respondent: Yeah, yeah.

Interviewer: Oh. Fabulous.

Respondent: Yeah. I do it all, sort of, my area is the south, so from [town] and [town]. So.

Interviewer: OK. OK. So you do, do a lot of travelling in your job?

Respondent: I do. Yeah. So, on the flip side of it, I also am in the car a lot. So. Yeah, I do a lot of driving, which isn't overly active, but it's, yeah.

Interviewer: Oh. OK. So, it's good you get a bit of a balance.

Respondent: Yeah, that's right.

Interviewer: So, thinking of those two activities over the last two weeks, how long would you have done them for?

Respondent: Over the two week period?

Interviewer: Yeah.

Respondent: Trying to think back on the last couple of weeks. I'm not sure where I was.

Interviewer: Yeah. Or, how often might be an easier place to start.

Respondent: Oh. Say, three days a week.

Interviewer: OK. So, three days a week, say.

Respondent: And then, say, a day in the office and probably a day's worth of travelling.

Interviewer: Yep. So, given the three days a week, what sort of time would be spent travel, you know, with the paddocks and...

Respondent: That's, that's what I mean, like, of the five days of the week, probably...

Interviewer: Oh yes the whole day?

Respondent: ... three days worth of walking...

Interviewer: Yep, yep.

Respondent: ... in the paddocks. A day's worth in the office and a day's worth of travelling.

Interviewer: Yep, yep. So, the equivalent of a seven hour day, type thing?

Respondent: Yeah. Roughly. Yep.

Interviewer: And when? During the day?

Respondent: Yeah, during the day.

Interviewer: Yeah, yeah.

Respondent: Anywhere from, you know, sort of seven thirty til five thirty.

Interviewer: Long days.

Respondent: Yeah. It, I mean, it depends where I am and obviously, and what...

Interviewer: And travelling.

Respondent: Yes, that's right. (Laughing).

Interviewer: And is this what you usually do at work?

Respondent: Yeah. Yep.

Interviewer: Yep.

Respondent: Yep.

Interviewer: It's not a one-off.

Respondent: In this job, yeah. Yep.

Interviewer: And does it change depending on the season or the time of year?

Respondent: Yeah. Heaps. Heaps.

Interviewer: Oh, OK.

Respondent: Heaps and heaps. Yeah. I mean, at the moment, it's quite busy, so we're testing because everyone's wanting to get the paddocks ready for crops,

so. Everyone's wanting soil testing done. But, you know. Sort of the summer, summer and winter are probably the quietest times.

Interviewer: The quieter times...

Respondent: Being autumn, we're really busy.

Interviewer: ... is when people get ready for planting?

Respondent: That's right. Yeah. I mean, I suppose it's coming into winter now, but it's still, you know, quite busy, but it slackens off a lot sort of July. July, August, it'll be dead and probably January, February will be pretty slow as well, so.

Interviewer: OK. And if you wanted to, is there anything that would have helped you be more active at work?

Respondent: Oh, not really. No.

Interviewer: Sounds like you're pretty active.

Respondent: It's just the nature the job is, is relatively active so it's, yeah.

Interviewer: Yep. OK. And would you, like, would you normally do that by yourself, those activities, or...

Respondent: Yep.

Interviewer: ... by, with others? OK, by yourself.

Respondent: Yep.

Interviewer: So, in the past two weeks, have you walked or cycled for at least ten minutes of the day to get to or from a places?

Respondent: Cycle no. I haven’t gotten on a bike for ten years. (Laughing).

Interviewer: Likewise. (Laughing). That’s alright.

Respondent: So, to get somewhere, as in...

Interviewer: Yeah.

Respondent: Yeah, just.

Interviewer: You know...

Respondent: I guess it depends when you say getting somewhere. Because if I'm getting to the paddock, then I'll walk, so it’s...

Interviewer: Yeah.

Respondent: Yeah. So yes. If that’s the case, then yep.

Interviewer: Yep. Yep. Yep.

Respondent: But not sort of, you know, to walk to the shops or anything like that. Because it’s yeah, not really doable. But yeah.

Interviewer: So that's over on top, on top of...

Respondent: So on top of work, probably... yeah. Actually.

Interviewer: Yep.

Respondent: Yeah. Yep.

Interviewer: Yep. OK. So that would be walking in paddocks, you were saying?

Respondent: Yeah, paddocks, or sort of around, you know, walking to buildings on the farm, or feeding dogs or taking them for a walk or, yep. Yep. All that sort of stuff.

Interviewer: Yep. Yep. Has to be done.

Respondent: Yep. Always...

Interviewer: Yep. Always something to do.

Respondent: Yes, there is. (Laughing).

Interviewer: And, what would be the intensity of that exercise? Would that be, activity, would that be steady or moderate?

Respondent: Oh, steady to moderate.

Interviewer: OK. And duration? How long might you have done that for?

Respondent: In the one stint, probably ten to 15 minutes, I guess.

Interviewer: Yep.

Respondent: Yeah.

Interviewer: And how often might you do that in the...?

Respondent: Oh, most days. Yeah.

Interviewer: There's always something to do, isn't there?

Respondent: Yeah. (Laughing).

Interviewer: Yep.

Respondent: Yeah.

Interviewer: And when would you have done that?

Respondent: Oh, most days.

Interviewer: Early morning? Late evening?

Respondent: Oh, sorry. Yeah, mainly, mainly evening. Yeah.

Interviewer: Yep. When you get back?

Respondent: Yeah. When I get home.

Interviewer: OK. So it'd be after work?

Respondent: Yeah.

Interviewer: And weekends?

Respondent: Yeah, well, I do a lot of horse riding as well, so that's sort of fairly, probably call that fairly vigorous.

Interviewer: Absolutely.

Respondent: Yeah. And that's normally weekends. Sometime, sometimes after work. And sometimes before work as well. Well, not Impact work, but.

Interviewer: Yep. OK. We'll follow that up in a moment. We'll get back to it. Oh, walking. Where did you do that? On the old property...

Respondent: Yep.

Interviewer: ... and to the buildings. And why did you do it there?

Respondent: Just felt like a walk. (Laughing).

Interviewer: And there were things to, to do...

Respondent: Yeah, it's close.

Interviewer: ... farm work to do?

Respondent: Yeah. I mean, it's close enough to walk, so you just walk.

Interviewer: Yeah.

Respondent: Yep.

Interviewer: Yeah.

Respondent: Yep.

Interviewer: Yep. And this is, is this what you usually do, or a one-off?

Respondent: Yep.

Interviewer: Yep. OK. And does it change depending on the season or the time of the year?

Respondent: Oh, slightly less in winter, because it's fairly bitter and cold. But yeah.

Interviewer: Yep. That would be fairly what you did?

Respondent: Fairly, fairly regularly.

Interviewer: And if you wanted to, is there anything that would have helped you use... forms of, these forms of transport? Like walking and cycling to and from places?

Respondent: Not really. No.

Interviewer: No. You already do that.

Respondent: No. You just, either you do or you don’t. Yeah.

Interviewer: You do or you don’t. You’re fine. OK.

In the past two weeks, have you been active around the house or the yard? I.E. at home, inside or outside for at least ten minutes of the day?

Respondent: Yep. Yeah...

Interviewer: So, OK.

Respondent: Mowing lawns. Feeding dogs. Yep.

Interviewer: Yep. And some of those activities what we’ve just been talking about, too?

Respondent: Yeah. Yeah.

Interviewer: OK, and the intensity of that?

Respondent: Oh, steady to moderate again.

Interviewer: Yep. And duration? How long you would do it for?

Respondent: Oh, an hour or so, I suppose, mowing lawns. Yeah. An hour and a half, maybe. Depending how long they are. (Laughing).

Interviewer: It’s always, grass isn’t there.

Respondent: Yeah.

Interviewer: And how often might you do it?

Respondent: Oh, about once a month, maybe.

Interviewer: OK.

Respondent: Yeah. Mowing lawns anyway. But I guess other things you’d...

Interviewer: There are other things...

Respondent: So hard to quantify, when you sort of just, yeah.

Interviewer: It’s, it’s tricky.

Respondent: Yeah.

Interviewer: Any other things that you might do inside or outside?

Respondent: Oh, apart from just normal cleaning...

Interviewer: Yep, yep.

Respondent: ... like vacuuming and dishes and cooking and yeah.

Interviewer: Well, it has to be done every day, doesn't it?

Respondent: Yeah, yeah. You sort of do what you need to. Cooking every day and vacuuming every couple of days, I suppose.

Interviewer: Yep. Yep. And that's sort of fairly steady?

Respondent: Yeah, just...

Interviewer: Yep.

Respondent: Yeah. (Laughing).

Interviewer: And...

Respondent: I don't run around the house with a vacuum, put it that way. So.

Interviewer: And how long might you spend on those activities?

Respondent: Oh, including everything? Probably...

Interviewer: Oh, just doing some, perhaps.

Respondent: Inside the house?

Interviewer: Yeah.

Respondent: You'd be doing... having do that every day, it'd probably be 20 minutes, I guess. Roughly. Maybe. (Laughing).

Interviewer: Yep. That’s fine. And, when might you have done that?

Respondent: Whenever I get a spare moment.

Interviewer: Oh, yeah.

Respondent: Normally weekends probably, or after work. Yeah.

Interviewer: Weekends, or after work. Yep.

Respondent: Yep.

Interviewer: Yep. Yep.

Respondent: I'm not home during the days, so. Yeah.

Interviewer: Yep. Yep. And who with? Would you have done that by yourself, or with others?

Respondent: Yeah, me and my partner.

Interviewer: Yep. And is this what you usually do?

Respondent: Yep.

Interviewer: Yep. And does it change with the season or time of year?

Respondent: No. Not really.

Interviewer: OK.

Respondent: (Laughing).

Interviewer: No, housework doesn't...

Respondent: No. (Laughing).

Interviewer: ... ever go away, really.

Respondent: It's never ending. (Laughing).

Interviewer: And if you wanted to, is there anything that would have helped you be more active around the, the house, either inside or outside?

Respondent: Oh, don't think so. No.

Interviewer: OK.

Respondent: No.

Interviewer: OK. OK. Now, have you been physically active for at least ten minutes during your leisure or spare time?

Respondent: Uh-huh.

Interviewer: OK.

Respondent: Yep.

Interviewer: Can you tell me about that?

Respondent: Yep. Playing tennis. Or, yeah, ride horses.

Interviewer: Yep, yep.

Respondent: Yeah. They're probably the main things. Taking, yeah, taking the dogs for a run.

Interviewer: Yep.

Respondent: Yeah. Yep.

Interviewer: And, so those three activities, what, how would you describe the intensity of those?

Respondent: Some are vigorous, some are sort of moderate.

Interviewer: OK. So what would be more vigorous?

Respondent: Moderate to vigorous, I guess. Well, riding and the tennis are fairly vigorous. But the, you know, the dogs just go for a run, just walk, wonder along, so it's, yeah, probably it's, yeah, moderate, I guess.

Interviewer: How long might you have done those act, each of those activities?

Respondent: I don’t know. Maybe... yeah, in the last couple of weeks, I did them for probably an hour.

Interviewer: Yeah. Each?

Respondent: I guess.

Interviewer: So riding is usually the weekend, isn’t it?

Respondent: Yeah. Riding sort of goes for a lot longer than...

Interviewer: Yep. Yeah.

Respondent: ... than other things, I guess.

Interviewer: So how long might you have spent?

Respondent: Oh, a standard ride probably go for three hours, I guess.

Interviewer: Yeah?

Respondent: Yeah. Just poke around.

Interviewer: You’ve got some good spots to go riding where you are?

Respondent: Yeah, yep. Yep.

Interviewer: Yeah. Quieter spots as...

Respondent: Yeah.

Interviewer: ... the property, or do you...

Respondent: Yep.

Interviewer: ... quiet roads as well?

Respondent: Yeah. On the property I go, go riding.

Interviewer: Mainly on the property?

Respondent: Always on the property.

Interviewer: Yep. Yep.

Respondent: Yeah, or 99.9 percent of the time anyway.

Interviewer: Yep. Yep. Great.

Respondent: Yep.

Interviewer: So that's, that's for riding. Dog walking. How long might that have lasted for?

Respondent: Oh, 20 minutes maybe.

Interviewer: OK. And tennis? How long that, might that have lasted for?

Respondent: Oh, last time it was about... I actually, well, I guess 45 minutes to an hour is probably the, yeah. Roughly. On average it would be, be about that long.

Interviewer: And how often would you have done those activities in the last couple of weeks?

Respondent: Oh, dogs probably... five. Tennis, once, because the weather's been awful, and riding...

Interviewer: It has. We've had a really cold snap.

Respondent: Uh-huh. Uh-huh. And riding... not for the probably the last month. Yeah. Yep. Yeah.

Interviewer: And, when would you have done those activities?

Respondent: Tennis and horses on weekends, and dogs, weekends and during the week as well.

Interviewer: During the week. And, would that be, after work for the dogs?

Respondent: Yep. Yep.

Interviewer: OK. And, why did you do it at that time?

Respondent: It's when I've got the most time. (Laughing).

Interviewer: It's when you've got the time.

Respondent: Yeah. Yeah. Yep.

Interviewer: OK. And where?

Respondent: On the property.

Interviewer: Yep.

Respondent: Yep.

Interviewer: Yep. And tennis?

Respondent: On the property.

Interviewer: Oh, OK. You play at home?

Respondent: Yep. Yep.

Interviewer: OK. And who would you have done the activities with?

Respondent: My family. Yep.

Interviewer: OK. And is that what you usually do?

Respondent: Yep.

Interviewer: OK. And does it change depending on the season or time of year?

Respondent: Yes. Yeah.

Interviewer: OK.

Respondent: Certain times of the year, April and November is busy on the horses, because we've got work we do on them.

Interviewer: OK. So you do some...

Respondent: Yeah, do stock work on...

Interviewer: Cross country, or?

Respondent: No. We do stock work on, on...

Interviewer: Oh, stock work. OK.

Respondent: Yeah. Yep. Yeah, so it's on a sort of all revolving around the farm still.

Interviewer: Yeah. Yeah.

Respondent: So that's, that's pretty intense for, you know, a week or so, I guess. Yeah, and tennis, obviously more in summer, because it's warmer and you've got more daylight after work. And yeah, it's more conducive for being outside. Yeah.

Interviewer: And, the dogs, they always need to, they need exercise, it doesn't matter what time of the year, don't they? (Laughing).

Respondent: Yeah. That’s it. Yeah.

Interviewer: OK. And so, if you wanted to, is there anything that might have helped you be more active in your leisure or spare time?

Respondent: I guess, I mean, I guess as far as tennis goes, the more people that are around, the more likely you are to, you know get a bit of a, bit of a game going. So yeah. I mean, having access to other people, then yeah, for sure.

Interviewer: Yep.

Respondent: Yep.

Interviewer: OK.

Respondent: And riding.

Interviewer: Yeah, because you mentioned it’s usually with family...

Respondent: Yeah. Yeah.

Interviewer: ... so if there’s more family or...

Respondent: Or if you’ve got your mates around or whatever, like, it’s, yeah.

Interviewer: Yep. It’s more.

Respondent: Yeah, you’re sort of more likely to go outside and do something. Yep.

Interviewer: And riding. Is there anything to help that?

Respondent: Yeah. Maybe, I mean... again, probably having a few more people around. I mean I'm, you know, enjoy going by myself, and you know, with family as well.

Interviewer: Do you usually go by yourself?

Respondent: It depends.

Interviewer: It depends. So you mainly go with family?

Respondent: Yeah. You know, family, friends. Just being, yeah, just depends on when it is and...

Interviewer: Yep. OK.

Respondent: ... and, yeah.

Interviewer: Right. Now, we've talked a bit about where you've lived and your physical activity. And we're going to now talk about the, the last part of it, is really looking at your physical environment that you live in...

Respondent: Uh-huh. Uh-huh.

Interviewer: ... and what, what might help and hinder. So, it's the physical environment where you live and work and play. And when we talk about physical environment, we mean all those physical things that surround

you, like the roads, and trees, houses. Shops, traffic lights. Whatever. You won't have any traffic lights up that way.

Respondent: Nup. (Laughing).

Interviewer: You know, lighting, whatever.

Respondent: Yep. Yep.

Interviewer: And some of these things are, are things that people who live in urban areas say that might be beneficial for their physical activity. So, I wanted to ask whether they're relevant for you as well.

Respondent: Uh-huh.

Interviewer: So first up, because we'll look at a, a couple of aspects of it. But first of all to start, can you just tell me a bit about how easy or difficult it is to be physically active in your area?

Respondent: I guess it depends where you draw the distinction. I mean, to be physically active, it's very easy, because you've got a lot of space, and you've got lots to do. As far as sort of sporting activities and you know, gyms and all that sort of stuff, it's not very easy.

So. Yeah. I guess organised physical activity's hard, but physical activity, you know, as part of your daily routine's quite easy. So. Yeah. Yep.

Interviewer: OK. Yep. That's good. Now. OK. So, first of all, I just want to ask about, OK. And why? What makes that easy and difficult?

Respondent: Well, difficult to, to organise sport because there isn't any. (Laughing).

Interviewer: Yep.

Respondent: Either that or travel to [city]. Yeah. Which, you know, it's not, you could do it if you really, really wanted to. But it would be pretty...

Interviewer: That would be a long trip.

Respondent: ... fairly late nights, and, yeah. And it's easy at home, because, as I say, you've just got stuff to do and it's, it's enjoyable, so you just do it. (Laughing). Yeah.

Interviewer: OK. Availability and accessibility of places to be active, OK?

Respondent: Uh-huh.

Interviewer: Now, if I were to ask you about places to be active in your area, what sorts of things would you tell me about?

Respondent: Places to be active? There's the golf course, if you're into golf, which I'm not, really. (Laughing). But that's obviously an option in the area.

Interviewer: And where's the golf course?

Respondent: It's in [town].

Interviewer: Yep.

Respondent: Yep. Yep.

Interviewer: OK.

Respondent: There is... that's about it really. Yeah.

Interviewer: Yep. OK.

Respondent: Apart from, sort of, yeah. People's farms. They're always, that's really the only place that's, I guess easy to be active in, yeah.

Interviewer: OK. Parks and...

Respondent: Yeah, there's...

Interviewer: ... walking tracks? Or...

Respondent: Yeah, Well, there is a walking track, but, sort of don't really drive to [town] just to go on the walking track. So.

Interviewer: Oh, OK. There is a walking track?

Respondent: Oh, there's a little. Yeah, there's one that sort of goes down to the river, but...

Interviewer: Oh, OK. I get you, yes.

Respondent: ... it's not really, like, yeah. (Laughing). A little one in the town, but there's no, you know, National Parks or anything around that you can go, go to and go from. Sort of a day walk or anything. You just do it. Yeah. Around your own place where you drive elsewhere.

Interviewer: Yeah. Yeah, because that's...

Respondent: Yep.

Interviewer: ... it's, so if you wanted to be, do anything off the property would be really getting in a car and, and...

Respondent: Yeah, you'd have to drive for, you know, I mean, [town] probably the closest one, and that's a good, good hour away I guess, so.

Interviewer: Yeah. That's quite a trek, isn't it?

Respondent: Uh-huh.

Interviewer: OK. So, does not having places to be active, i.e., that, you know, available, influence whether you're active or not?

Respondent: It can be to an extent. Yeah. I mean, if, if the option was there to do, like, sport, I'd definitely take it up for sure. So yeah, I guess it does influence it, yeah. Yep.

Interviewer: OK. And if you wanted to be more active, are there things or places that would help if they were available or more accessible? This is a bit what you were saying.

Respondent: Yeah, well, yeah, obviously it would. Yeah. Yep.

Interviewer: And in particular sport?

Respondent: Oh, just anything.

Interviewer: Anything?

Respondent: Yeah. Yeah.

Interviewer: Yep.

Respondent: Just an option, you know, even if it's Zumba classes, or if it's, you know, anything like that at all, all help for sure. You know, not having to travel an hour. You know, having to drive for ten minutes is a lot more attractive than travelling an hour just for a, you know...

Interviewer: Absolutely.

Respondent: Yeah, game of sport or a class or a, you know, whatever. So.

Interviewer: OK. And are there any halls in [town] that...?

Respondent: There's a town hall, but they don't ever really have anything...

Interviewer: They haven't, never have anything?

Respondent: ... like that there, no.

Interviewer: No. OK.

Respondent: Not that I'm aware of, anyway. So. Yeah.

Interviewer: Now, destinations. If I were to ask you about places that you could walk, or cycle, from your home, what sorts of things would you tell me about?

Respondent: Walking from home is just, yeah, walking on the farm.

Interviewer: Does...

Respondent: I guess (inaudible – 28.11).

Interviewer: ... you couldn’t walk to, I don’t, shops in [town], or?

Respondent: No. No.

Interviewer: School, yeah?

Respondent: No. Well, you could but it would be a day walk, so. (Laughing).

Interviewer: Any friends or, live close by that you would walk to?

Respondent: No. Not that I’d walk to, no.

Interviewer: No. It’s all, yep. So you really do need to, if it’s not on the property, it’s in a car.

Respondent: You've got to, yeah. Yep. Absolutely.

Interviewer: And does not having destinations whether, influence whether you're active or not?

Respondent: No.

Interviewer: No.

Respondent: No.

Interviewer: And if you wanted to be more active, would it help to have more destinations within easy walking or riding distance?

Respondent: Possibly. Yeah. I mean, it's, I guess if you'd gone walk, walked somewhere and go out for breakfast or, you know, on a Sunday morning or something like that, then you, you may, more than you would, you would otherwise, but yeah.

Interviewer: Yeah. You could do it for a few, few reasons, rather than just...

Respondent: Yeah. That's right. Yeah. It's sort of hard, like you don't really; they're not there, so you don't really ever consider it.

Interviewer: No.

Respondent: So you just...

Interviewer: And, and for you, your closest...

Respondent: ... and you get...

Interviewer: ... centre's [town], and that's 15 k, and...

Respondent: Yeah.

Interviewer: ... Yeah.

Respondent: And you sort of get used to just driving places. It doesn't, you know, like, an hour's drive is a long time, but it's, you know...

Interviewer: Yeah. It's unlikely that you would end up in a, a cafe just down the road (laughing), two kilometres or something.

Respondent: Yeah, exactly. Yeah. Yep.

Interviewer: OK. Now, I want to talk a bit about neighbour, well, neighbourhood design is what it's called. It's the sort of built features, or non-natural features, man-made features in, in your area. So if were to ask you about those built features, what sorts of things would you tell me about?

Respondent: Well, there's the town. So, you know, obviously houses and shop and servo. Pub. That's about it. Yeah. There's obviously the buildings on the farm. There's the other, you know, farm buildings on the neighbouring properties.

Interviewer: How close would your neighbouring property be, just out of interest?

Respondent: Oh, about, as the crow flies, probably four or five k, I guess.

Interviewer: Yeah, so it's big properties, aren't they?

Respondent: Yeah.

Interviewer: OK. And then you've got the main highway? So, there, are there any footpaths in your area?

Respondent: No.

Interviewer: No. In [town]?

Respondent: Yes.

Interviewer: There would be?

Respondent: Yes. But...

Interviewer: OK. OK. Would you ever walk along the roads at all? No? There would be no reason, you use the property.

Respondent: No, not really. Yeah.

Interviewer: OK. So, just about walkability in your area, it's really your property, but what's the walkability like off the property?

Respondent: The walkability? In what sense?

Interviewer: Yeah, like...

Respondent: Is it easy to go for a walk, do you mean?

Interviewer: Yeah.

Respondent: Oh, yeah. I suppose it would be. If you, yeah.

Interviewer: Is there any problems with traffic? Or?

Respondent: No. No. No trouble like that.

Interviewer: It's quiet.

Respondent: Yeah, no. I mean yeah, in that sense, no, there's no, it's, it's very easy...

Interviewer: OK. If you wanted to ride...

Respondent: If you wanted to go for a walk.

Interviewer: ... off the property, for instance. If, if for instance you wanted to ride off the property?

Respondent: Yeah. Well, you probably wouldn't ride off the property because; unless you're on someone else's, because you don't really want to be riding up the highway or through the town, so.

Interviewer: No. Because of? It's that, because of the traffic, or?

Respondent: Yeah...

Interviewer: It’s just not pleasant?

Respondent: You just don’t. You don’t. You, you wouldn’t choose to ride through town, over riding through a paddock, so. (Laughing).

Interviewer: No it’s more interesting.

Respondent: Yeah, I guess it’s for the interest type thing, and the, I mean, traffic, I guess is a, is a factor, but it’s not, I mean, the traffic’s not excessive through [town]. It’s, you know.

Interviewer: No. It’s quite quiet. OK. Do the non-natural features, or built features in your area influence whether you’re physically active or not?

Respondent: No.

Interviewer: No.

Respondent: No.

Interviewer: Because that’s not where you do most of your activity...

Respondent: Yeah, that’s right.

Interviewer: ... basically. And if you wanted to be more active, are there any built features in your area that would help? Given that, you know, you don’t...

Respondent: Yeah.

Interviewer: ... don't use, you know...

Respondent: If it was feasible, you know, squash courts or gym, or anything like that. I mean. It's probably not going to happen, but it's, if they were there, then they'd get used for sure. Well, I'd probably use them, yeah.

Interviewer: Yep. Yep. OK. Now, the other side of it is the natural environment. The aesthetics. If I were to ask you about the aesthetic qualities or the attractiveness of your area, what sorts of things would you tell me about?

Respondent: Yeah, it's beautiful. Yeah, it's very easy to, to be a part of. Yeah.

Interviewer: Hilly and...

Respondent: Yeah, it's undulating.

Interviewer: Undulating. So...

Respondent: Bit of bush, bit of, you know, scrub, bit of...

Interviewer: Bit of bush, bit of trees.

Respondent: Bit of pasture.

Interviewer: Yep.

Respondent: Yeah, it's quite varied.

Interviewer: OK. And so, sort of, yeah. So you've got a, a combination of sort of open paddocks with sort of bushland and...

Respondent: Yep, yep, that's right.

Interviewer: Yep. Yep. OK. Do you think your local environment is aesthetically pleasing?

Respondent: Yes, I do. (Laughing).

Interviewer: You do. And near your home?

Respondent: Beg your pardon?

Interviewer: Near your home?

Respondent: Yeah. Yep.

Interviewer: Absolutely.

Respondent: Yep.

Interviewer: OK. And on the other side of it, is there sort of any rubbish, litter, graffiti in the area?

Respondent: No graffiti as such. No. You get rubbish up the highway every now and again, but it's pretty clean really.

Interviewer: Yep.

Respondent: Yeah.

Interviewer: OK. Now, do the aesthetics of your area influence whether you're physically active or not?

Respondent: Oh, not...

Interviewer: It's a bit...

Respondent: Yeah.

Interviewer: It's an interesting one to ask.

Respondent: Yeah.

Interviewer: Because, often these questions are asked in urban environments, where people don't necessarily...

Respondent: Yeah.

Interviewer: ... have a lovely environment.

Respondent: Yeah.

Interviewer: In rural areas, it's sort of taken as part of why you're living there. (Laughing).

Respondent: Yeah. That's right. So...

Interviewer: So to ask and separate it out is a bit, bit hard to do.

Respondent: Yeah. I think... yeah. I mean, you're more likely to, to want to be outside...

Interviewer: Yeah. Yep.

Respondent: ... in a beautiful place, because, we just are. (Laughing).

Interviewer: Yeah.

Respondent: But whether it actually makes me more active or not, it's hard to quantify really. It's...

Interviewer: Other than you really enjoy being out, outdoors...

Respondent: Yeah. Yeah.

Interviewer: ... and doing outdoor activities.

Respondent: Yeah. And I think you either enjoy being outside or you don't. You know, you either like going out and doing stuff, or you like, you know, staying inside and reading a book, or watching telly or something. And I guess, you know, the former category that I do enjoy being outside. So it, I guess, you know, regardless of where I was, I'd always probably be outside. But whether you know, the fact that it's a nice place makes me more likely to be outside, I don't know.

Interviewer: Makes it a bit easier.

Respondent: Probably not, I don't think.

Interviewer: Yeah, because you would do that anyway.

Respondent: Yeah, yep.

Interviewer: But it's certainly a bonus having a nice...

Respondent: It is, yep. Absolutely.

Interviewer: ... place.

Respondent: Yep. Absolutely.

Interviewer: Yep. Absolutely. And, and being able to, actually be on your own land to do, to exercise.

Respondent: Yeah. And you sort of know that you don't have to see people you don't want to see. (Laughing).

Interviewer: And if you wanted to be more active, are there any features of your, natural features of your area that might help, that were more, if it was, if they were more aesthetically pleasing or attractive?

Respondent: No, I don't think so. No.

Interviewer: Yeah. So, it’s pretty beautiful?

Respondent: Yeah.

Interviewer: Now, the last area’s just about safety in your neighbourhood. If I was to ask you about safety, what things would you tell me about?

Respondent: Oh, it’s pretty safe.

Interviewer: Yeah. Yeah. So, physic...

Respondent: Nice. Yeah.

Interviewer: ... so personally...

Respondent: I mean I could walk down the street in [town] at 12 o’clock at night and not feel worried. Put it that way, so yeah. By myself. So, that’s not, yeah. I'm not, not too concerned about safety.

Interviewer: And road safety?

Respondent: It’s pretty good. Yeah. There’s probably more hazards. You know, you’ve got to look out for livestock or, you know, live animals and stuff occasionally, so, but yeah.

Interviewer: It’s pretty good.

Respondent: No, it’s pretty good. Yeah.

Interviewer: OK. So, OK. So, personal safety, that doesn't influence whether you're physically active or not?

Respondent: No.

Interviewer: And not so for road safety...

Respondent: No.

Interviewer: So if you wanted to be more active, are there any personal or road safety issues that if they were addressed might help you be more active?

Respondent: No, I don't think so. No.

Interviewer: OK.

Respondent: Because there's no, I don't really have any issues, anyway. So it's, yeah.

Interviewer: Yep.

Respondent: I don't think so.

Interviewer: And just before we finish up, is there anything else you'd like to tell me about where you live? Physical activity, or the environment before we finish today, that we haven't, don't think...

Respondent: We've covered...

Interviewer: Yeah.

Respondent: ... covered most things, I think. Yeah. In a sort of work and personal and leisure times sort of main, you know, obviously the main times you're going to be active. Yeah, no, I think we've covered them pretty well.

Interviewer: OK. That's great. Thank you very, very much.

Respondent: No worries.
