# Supplementary material for: A Qualitative Study of Environmental Factors Important for Physical Activity in Rural Adults
Source: PLoS One. 2015 Nov 10;10(11):e0140659. doi: 10.1371/journal.pone.0140659 (PMC4640845; doi:10.1371/journal.pone.0140659)
Supplement: S5 File — (PDF) [file pone.0140659.s005.pdf]

Interviewer: Right, so I thought I might start by just talking a bit about…what is it like, sort of, living here; and what your likes and dislikes are? Now, you're living in [town] itself?

Respondent: Yeah, I live up the road here, so I'm probably not like ultra-rural.

I think that I'm in the city.

Respondent: I like it. We all walk to school. We all walk around to the skate park. So we live fairly close in that we don't have to drive everywhere, which is, I think, sort of good.

Interviewer: Yeah.

Respondent: But I only rent as well.

Interviewer: So how far are you say from the centre of [town] here? Is it…in terms of walking?

Respondent: Probably five, ten minutes.

Interviewer: Five or ten minutes. So, that's sort of under a kilometre for you?

Respondent: Yep, yep. I wouldn't necessarily walk around at night time.

Interviewer: No.

Respondent: But in the day, like we'll walk around. And if it's raining we don't walk, which it rains a fair bit down here.

Interviewer: Yes, I've noticed it does.

Respondent: It’s one thing we can’t do (speaking to child)…

Interviewer: OK. So…and what’s…do you live in a house with a house block?

(Interruption – dealing with child)

Respondent: Yeah, we’ve got like 15 acres but the…

Interviewer: Oh, have you?

Respondent: But someone agists that but we can still walk on it…

Interviewer: Oh, OK. Oh, that’s good. You’ve got access to, to…

Respondent: Yeah, we’ve got a paddock area.

Interviewer: [Laughter]. Right. So it’s agisting for horses is it?

Respondent: They put cattle on it.

Interviewer: Oh, cattle. OK, OK. And the length of time you’ve lived here?

Respondent: A year.

Interviewer: A year. And what were your reasons for coming to live here?

Respondent: ‘Cause it was nice. I was in the city for a while with my sister. We shared a house. Then, because I’m a single parent, I couldn’t really afford to live in that same area. This was a better area, I thought, than all the areas that I could live in up there, so.

Interviewer: Yeah, OK. And one of the things about this sort of research is that because it's been mainly done with women in cities…

(Interruption – discussion with child)

People often refer to their local neighbourhood. And I was sort of interested, particularly as you have lived in the city, whether that has any meaning or relevance to you now, living rurally. So if I asked you, what does the word neighbourhood mean, would it mean anything here?

Respondent: Not really.

Interviewer: No.

Respondent: It's like…the town.

Interviewer: Yes, OK.

Respondent: It would be like the whole of [town] town.

Interviewer: OK. So that's what a neighbourhood would be for you?

Respondent: Yeah. Here yeah.

Interviewer: OK. And how big's [town] in that way?

Respondent: To get around the…

Interviewer: A couple of kilometres?

Respondent: …yeah, you can sort of walk around it.

Interviewer: Yeah.

Respondent: But, I actually went to [x]'s Hill with a friend of mine, we went through some gates, I'm not sure if you were supposed to, but…you could see right out.

Interviewer: Oh, OK…

Respondent: And it actually stretches really far. There's lots of houses up behind the hills and things as well...

Interviewer: Ah, that's not down here on the flats…

Respondent: So there'd be lots of people living out there that wouldn't get…that would have to drive everywhere.

Interviewer: Yep. And you more see the immediate sort of, probably kilometre of people, or so, both ways; that would be more your local area. OK. And what about the word community? What does that mean to you? Is that a relevant thing here?

Respondent. I think there's a fair few people that work in [town] e, and there's a fair few people from [town] that work here, maybe. So it's like, sort of local, yeah. I don't work at the moment.

Interviewer: So community's about the people that sort of live here?

Respondent: Yeah. Actually there's a fair few mothers from playgroup that work at school. So…

Interviewer: So it’s the people you know through playgroup and…

Respondent: Yeah.

Interviewer: Yeah, yep.

Respondent: Yeah, it’s pretty…community, yeah, definitely.

Interviewer: Now, I just wanted to talk to you a bit about physical activity; because that’s a really broad term, and it just means different things to different…everyone. What does it mean to you?

Respondent: Like getting out and stretching out, I suppose. Because, you can sort of dawdle when you’ve got kids. But you never really get to walk; or you never get to run or do anything at your own pace. But that’s what I would consider it to be, because that’s what I remember.

Interviewer: Yep.

Respondent: So something that’s ongoing at your own pace.

Interviewer: Yep, that’s a really good way of looking at it.

Respondent: Yeah.

Interviewer: Do you consider yourself physically active?

Respondent: Yeah, a little bit, yeah.

Interviewer: And why do you say that?

Respondent: Well, I come down here and do yoga twice a week, and we walk around a fair bit because we can. I probably could be more active, but sort of not as a rule. I tire, too, really…I do what I can, I suppose, yeah.

Interviewer: Yeah, in your life – because there's lots of things to do.

Respondent: And they go away with their dad on the fortnight, once a fortnight. And I find those times are when I can do a walk, like a proper walk, or something like that.

Interviewer: And so where does physical activity fit into your life? Is it a priority?

Respondent: It is, but you just do what you can.

Interviewer: Yep, yep. Because how old are your children?

Respondent: Four and six.

(Interruption – talking with child)

Interviewer: So I just want to have a look a bit more about that. In our research when we think about physical activity, we think of any activity that lasts for at least ten minutes. So anything that raises your heart rate, as you've been saying, and to feel a bit warmer, or you're huffing and puffing a bit…breathing a little heavier. So thinking about this type of physical activity during the past two weeks; we're just going to go to different parts of your life, just so we don't miss things out. Now you currently are parenting full time so you're not actually working at the present?

Respondent: No.

Interviewer: So in the past two weeks have you walked or cycled for at least ten minutes at a time to get to or from places?

Respondent: Yep.

Interviewer: You have. Could you tell me about what you did?

Respondent: What did we do? We went to school and then we came down to Yoga and did yoga and then we went home again. And then other days we have gone into the city…into the main street, done a couple of things there, stopped at the skate park, run around a little bit, gone home. All that…

Interviewer: Yep, so you tend to do a few things together.

Respondent: We'll go down to the creek while [daughter] at school with the dog and…yep. And sometimes we've gone to get wood, we go and get wood.

Interviewer: So there's quite a few things to do here and enjoy?

Respondent: Yeah, yeah. And there's a fair few playgroups, so you do that. They're like an hour. So you go…the mothers don't – we don't move around that much. But they all do.

Interviewer: That's fair enough. And having to think about those activities that you did…and walking, if you had a look at whether that was sort of steady exercise or moderate or intense, what would you say?

Respondent: Steady.

Interviewer: Probably a pretty steady, yep? And just a bit of guesswork. How long would you have spent doing those activities?

Respondent: Sometimes hours, yeah. Last year I played netball, but now that we don't have teams…we…

Interviewer: Oh, OK. So they don't have one here yet?

Respondent: Well, a lot of the girls from down here, they're having babies this year.

Interviewer: Oh, OK.

Respondent: So it's a year off this year. Yeah, so that was a good hour of intense…once a week.

Interviewer: And how often would you have done those activities that you were talking about doing during the fortnight?

Respondent: A fair few. Like…

Interviewer: Would you do them every day or…?

Respondent: Oh, well every day we do something. Got to look after this one.

Interviewer: Yep, yep, so…

Respondent: So the creek, probably like once or twice a week. Same as Skate Park. Yoga we do on Monday. And then there's one at night, but we'll probably drive to that one.

Interviewer: Oh, I see. And where's that?

Respondent: Here.

Interviewer: Oh is it? So there's something going at night? Oh, good. OK.

(Interruption – child talking)

Interviewer: OK, so when did you do it, was it during the day?

Respondent: Yep.

Interviewer: OK, and weekdays or weekends?

Respondent: Mainly weekdays and then weekends we do something else.

Interviewer: OK. And you've told me about where you do it. And why did you do it at those places? What attracted you to pick…to do those activities?

Respondent: They're free.

Interviewer: Yes. [Laughter]. That's a very good point.

Respondent: They're free and there's like no time, so you just do it.

Interviewer: And they're also things that your little one likes to do?

Respondent: Yep. He rides his bike sometimes.

(Interruption – Talking to child – asking name)

Interviewer: And so…OK. Were you alone or with others?

Respondent: Mostly alone.

Interviewer: OK. Or with [son]?

Respondent: With [son], yep.

Interviewer: And is this something you usually do, or was it one-off?

Respondent: No, normally.

Interviewer: Yep, and does it change with the season or time of year?

Respondent: Yeah, because summer… well summer you're sort of out a lot more.

Interviewer: Yeah.

Respondent: But in winter you stay at home a lot more.

Interviewer: Yep, yep.

Respondent: And it gets dark pretty early, too. So you don't…from about 4:30 you don't really go anywhere.

Interviewer: No, because it's dark.

Respondent: You watch TV [laughter]. Watch TV, hey?

Interviewer: And if you'd wanted to, is there anything that would have helped you be more active, walking to and from places?

Respondent: Personally, I think it would be good if there were tennis courts here. Because that's something you can do with the kids.

Interviewer: Yeah, yeah. Community…

Respondent: Because it's free, you can just come down and play with a friend. But you don't need many people for that.

Interviewer: No.

Respondent: Yeah, lights around the place. Like there's lights on my street until about half way. Or not even… a quarter of the way up. And then there's the nothing - the lights.

Interviewer: Oh, OK. So it's fairly dimly lit. It's only the center part that's… light...

Respondent: Yep. And then there's… I don't know I don't really come down here, but…to the park at night time. Because it's for the young people.

Interviewer: Oh, right [laughs].

(Interruption – talking to the child)

Respondent: So, yeah. You can hear cars down here sometimes so I stay where you can see them.

Interviewer: So that's about some personal safety, too?

Respondent: Yeah.

Interviewer: Yeah.

Respondent: There's some good walks up our road as well, that we've done. But we do that but we do that here and there. Just for something different.

(Interruption – talking to the child)

Interviewer: So the past two weeks, have you been active at home, inside or outside in the yard?

Respondent: Yeah, sort of.

Interviewer: Yeah? Can you tell me what you did? There's always the dreaded housework [laughter].

Respondent: Well, yeah, there's housework. What's that… is that moderate?

Interviewer: Yeah. I think it well could be. Can we put… yes?

Respondent: Yeah, a bit of…like we go and get us some wood and it's small pieces, but we get it for free. So we do that once a week probably. So we get loads of wood.

Interviewer: That would be moderate or...

Respondent: Sometimes we do some gardening, but not very often. But, yeah, not a great deal at home. A little bit. I move around a lot, but I don't actually do a lot of heavy lifting or anything.

Interviewer: So how long would those activities that you've talked about, how long might you have done them for?

Respondent: On and off.

Interviewer: And how often?

Respondent: Fairly often. Every day.

Interviewer: Yeah, every day. Yep. Now, who with?

Respondent: Myself.

Interviewer: Yeah, OK. And is what you usually do?

Respondent: Yeah.

Interviewer: OK. And does it change during the season?

Respondent: Some of the time. I would do more gardening in summer.

Interviewer: Yeah, less wood carting.

Respondent: Less inside stuff.

Interviewer: Yeah. And if you wanted to, is there anything that would help you be more active around the home, inside or out?

Respondent: Sometimes I think I'd like to have a…like this year I'd like to buy them a trampoline. But something that was like maybe a bike or a like one of those things.I've noticed from living in the city, I used to just walk around a lot more. You do smaller trips here and there, you can do that late at night if you wanted, and yeah.

Interviewer: Yeah.

Respondent: So you don't do things late at night here.

Interviewer: So you're thinking about bikes for them and how about you as well?

Respondent: Yeah, a bike would be good, yeah.

Interviewer: Is it fairly flat around here?

Respondent: It's not too bad. But he's started two wheels.

(Interruption – child talking)

Interviewer: And have you been physically active for at least ten minutes in your spare time in the last two weeks?

Respondent: Yep.

Interviewer: Yeah, OK, so can you tell me about what you did

Respondent: Just like walking. I walk a lot.

Interviewer: You were saying that you were doing some other walks, further around the area.

Respondent: Yep.

Interviewer: OK. And what would be the intensity of those?

Respondent: They'd be like, brisk. Sort of being pretty vigorous.

Interviewer: Yep. And on the flat or up and down?

Respondent: Up and down.

Interviewer: Yeah, yep. Because there'd be some beautiful walks around here.

Respondent: Yeah.

Interviewer: And how long would you have done them for?

Respondent: An hour, hour and a half.

Interviewer: And how often?

Respondent: Like if they're away for two days I do it every day.

Interviewer: Yes.

Respondent: And sometimes you do two a day. Like you might go down to…and come around here and then you might take the dog for a walk later. Yeah, not heaps though. I read a lot of books.

(Interruption – discussion with child)

Interviewer: We won't be too much longer. And if you wanted to, is there anything that would have helped you be more active in your spare time?

Respondent: Well, one of the ladies I'm friends with down here, I haven't been done here for years and years, but I mean, they've all got children as well, so they're usually spending time with their husbands and…so I don't really know actually. Sometimes you think that if the Sports Centre up there had childcare, you might maybe do a class or two. Like, I'd definitely do a class or two up there if they…

Interviewer: So where's the Sports Centre? That's in [town]?

Respondent: No, it's at [town].

Interviewer: Oh, [town].

Respondent: So you could ride your bike there, have your class, and ride home again. It's only, what…five minutes.

Interviewer: Five k or so?

Respondent: Yeah, yep

Interviewer: And they've got various classes there?

Respondent: Well, I think they've got an aerobics type thing, they've got a pool there, things like that. But then there's no one to watch them. Like, I used to take them up to netball but that was because there were usually a lot of people around. So they would play while we were on the court, so that was OK. But I think if you were doing something with a class, like they come to yoga here, but I know they're annoying [laughter]. But what do you do?

Interviewer: Yeah, well that's the thing. If you need…

(Interruption – child talking)

Interviewer: Now, ah yes. So when you were saying doing longer walks, do you usually do that in the daytime?

Respondent: Yep.

Interviewer: Yep. Weekends or weekdays?

Respondent: The other walks? Probably the weekend.

Interviewer: OK. And how far away would they be? There'd be somewhere that you'd have to get in the car and actually drive?

Respondent: Yeah.

Interviewer: Yeah, so…

Respondent: Half an hour.

Interviewer: And who with?

Respondent: Whoever's around. Sometimes a sister or brother who's come down, maybe to visit, or…sometimes take these guys.

(Interruption – talking with child)

Interviewer: And is it what you would usually do?

Respondent: Yeah, we usually do something. Try to anyway. But, yeah, there's not a lot to do down here. And once you've done everything, you do it all again [laughter].

(Interruption – dealing with child)

Interviewer: So the last part is just talking about physical environment. And that's where you work and live and play in. OK? And when I talk about

physical environment, it means it's anywhere from roads to trees, shops, traffic lights, anything – parks…yep. And some of these things are things that people have actually, in the city, have actually said it might be important to their physical activity. So we're interested in knowing whether they're relevant to you.

Respondent: Yep.

Interviewer: So can you tell me about how easy or difficult it is to be physically active in your area?

(Interruption – child talking)

Respondent: It's actually very easy not to be physical because you're asking me now when it's sort of cold and everything's wet a lot of the time. A lot of the parks, all the stuff's wet. All the play equipment wet, all the grass wet, and everywhere that you want to sit down and watch the children is [laughter] cold. Like, you get some nice days, but a lot…like last year when we came down…it was wet a lot. And then, yeah, so…and you don't grow as many vegetables so you don't do as much gardening. Yeah. Maybe that's why. I don't know. But, yeah, even when I lived in the city I didn't do a lot of paid for things either. There was a lot more sort of free sort of stuff to do

Interviewer: Yeah, and there's less down here? And that's what I was going to ask you about to – the availability and accessibility. If I was to ask you about places to be active, what are the sorts of thing that you would tell me about? You've told me a bit about there's the park, the creek, and…?

Respondent: They go swimming once a week.

Interviewer: OK, and is that at [town]?

Respondent: Yep.

Interviewer: Yep.

Respondent: And that's a lesson.

Interviewer: Yep, yep.

Respondent: I mean I could swim when they're in the pool, but that's like half an hour and sometimes that just don't fit in....

Interviewer: That doesn't give you much time does it?

Respondent: No, and there's… it's only a small pool. So there's…sometimes it's packed and sometimes it's not.

Interviewer: And I'll presume there's a cost attached to it?

Respondent: Yep.

Interviewer: And sometimes it's just really too busy?

Respondent: Well, if they both have a lesson, and then I go for a swim, it'd be like 30 dollars a week, which is sort of pretty hefty. So at the minute it's about 22 just for them. Which, you know, that's OK because it's a lesson. But if it was a leisure thing, if it were leisurely swimming once a week, then we might give that a miss here and there. Yeah, yoga's pretty good, yoga's five dollars a go.

Interviewer: Yep, and that's here is it.

Respondent: Yep.

Interviewer: And once a week and I can bring him..

Respondent: Yep, a night every week. That's twice a week, sorry.

Interviewer: That's twice a week?

Respondent: Well, it is now. It used to be once and just doing two now.

Interviewer: So that's five dollars a session, is it?

(Interruption – talking to child)

Interviewer: And any other things that are available in terms of recreation or walking tracks or green spaces or…?

Respondent: Well, yeah there's like the track the [x] Walk and that goes across the road and past the RSL.

Interviewer: Yep.

Respondent: And so that's sort of nice. But then it turns back into housing. But yeah a really long walking track into town would be good. We could take the dog along there and they could ride their bike.

Interviewer: So when you're saying the [x] track,how long would it take you to walk?

Respondent: They've got ten minutes on there, but I reckon it's five.

Interviewer: Oh, OK. It's a short, short one. Yeah, this is a beautiful area here.

Respondent: Especially along the highway. Yeah. It'd be good to have something that followed the river.

Interviewer: Yeah.

Respondent: Because they only…well when you've got little children, too, they're only going to walk for how long? So if you've got something that you can either push the pram or they can ride their bike, you've got more of a chance of…

Interviewer: To have a go at a large track. And at the present moment there isn't one.

Respondent: Not really.

Interviewer: Is there any way you can get a longer ride than that?

Respondent: Well, the (inaudible) walk, really. Like that's grass, so you wouldn't take a pram or anything. But you could.

(Interruption – talking to child)

Interviewer: So does having, or not having in this case, places to be active influence whether you're active or not?

(Interruption – child talking)

Respondent: Yeah, definitely.

Interviewer: Yeah?

Respondent: Because you have to make up your own thing.

Interviewer: Yeah. Dependent on you and what you can find your way around with and what [son] is able to do, too. Yep, OK. And if you wanted to be more active and there's thing or places that would help, if they were available or more accessible?

Respondent: Yep.

Interviewer: I think you've been talking about that, you were saying that child care at the Sports Centre and…

Respondent: Child care at Sports Centre and probably a walking track or something. Something that was concrete, something like that.

Interviewer: Some sort of decent hard surface, yeah.

Respondent: Where a bike and a pram could go on. It makes a difference.

Interviewer: And can a bike and pram go happily around [town] itself?

Respondent: Yeah on the footpath is fine.

Interviewer: Yeah, that's OK.

Respondent: But then the footpath only goes this way. It stops. So you're on the side of the road which is actually the highway to [town].

Interviewer: Oh right, that doesn't sound real safe.

Respondent: In actual, for me, you've got things…like I said, if I lived further out I would drive, I wouldn't really do anything.

Interviewer: OK. So just a couple more questions. Destinations, if I was to ask you about places you could walk or cycle to from home, what sorts of things would you tell me about? For instance, shops, schools, recreational facilities, friends, places…

Respondent: Yeah, that's it.

Interviewer: So there's all of those. And does having or not having destinations influence whether you are active or not?

Respondent: Yep.

Interviewer: It does. And why?

Respondent: Well, when you're by yourself, it's good if you have somewhere that you're going to, because it makes you want to go somewhere and then part of going there is getting there.

Interviewer: So you don't just, you know, go for a walk. There's a few things you can do and it makes sense in terms of…

Respondent: If you say to the children, let's go for a walk, it's not fun. If you tell them let's go to somewhere, that's exciting.

Interviewer: Yeah, I'm with you. And if you wanted to be more active, would it help if you had more destinations? With your need to walking or riding distance.

Respondent: Probably.

Interviewer: Yeah.

Respondent: I think it's too, the age of children, has got a lot to do with it.

Interviewer: Yeah.

Respondent: Plus, too, I'm single. There'd be other people that have partners that might do things on their property.

Interviewer: Yes.

Respondent: Which would be exercise, but I mean I don't have that, so…I think a fair few people down here would have partners that might go fishing or they might ride motorbikes or, you know, they might do things like that.

Interviewer: OK. Now, I just thought I'd ask you a bit about the built environment or the man-made bits – the things that are like footpaths and lighting and walkability. And you have said a bit about that. Are there any other things that you'd like to tell me about?

Respondent: No. It was just useful having them… especially in a place where you don't know everybody. So you don't know where one person's property ends, so you can't go for a walk wherever you feel like. Because even the walking tracks, they're tracks. So they're man-made. So everything that you use for free is pretty much man-made.

Interviewer: Yep. But certain of these things around tracks and better lighting and about better…how walks connect together around the place would help.

Respondent: Yep.

Interviewer: But if you wanted to be more active, are there any built features of your area that would help, and we've just been talking about those things.

Respondent: Yeah.

Interviewer: The other side of the natural part of your environment, if I was to ask you about the aesthetic qualities or the attractiveness of your area, what sorts of things would you tell me about?

Respondent: That are nice?

Interviewer: Which are natural.

Respondent: The creek.

(Interruption – child talking)

Respondent: Is that what?

Interviewer: Is the creek the river?

Respondent: Yeah, yep. We sort of only access it from certain points because there's a lot of properties backing onto it.

Interviewer: Oh, OK. So there's no sort of public right of way that…

Respondent: Well, there is just here and there is further up our road.

Interviewer: It's limited, is it? And do you think your local environment is aesthetically pleasing or attractive?

Respondent: Yep.

Interviewer: And near your home?

Respondent: Yep.

Interviewer: Yep, OK. And do the aesthetics of your area actually influence whether you're physically active or not? The beauty of it?

Respondent: Yeah, maybe. Yeah.

Interviewer: Because it's sort of part of living in a rural area where it's beautiful and you enjoy it. It's one of the benefits of being here [laughs]. Have you got trees on your property, or…?

Respondent: Yeah.

Interviewer: Sounds like it's a rather beautiful sort of spot.

Respondent: It is very beautiful.

Interviewer: And you were saying, what, you're down by the river, or not far from it?

Respondent: We come in. So we cross a creek to get to school, over the bridge. But we can reach that bit just down the side of the bridge and along a tiny bit. That's not very far, that's not a walk.

Interviewer: Yep.

Respondent: And then you've got people's properties backing onto that. And that's what I find a lot around here, you can't just decide to go for a walk anywhere you want. You know, it's private property, which is fair enough [laughter].

Interviewer: But there hasn't been any…so it's like when places grow up but it hasn't really thought about people being able to move around them.

Respondent: No, there's that one here, that's just across the park but…

Interviewer: And if you wanted to be more active are there features of your area that might help if they were more aesthetically pleasing?

Respondent: You could have a…I think there's one undercover barbeque area next to a small park. But, yeah, like have a bigger park with a more undercover area for the rains.

Interviewer: Yeah.

Respondent: Like an undercover area would be good.

Interviewer: OK, yeah.

Respondent: Something like that. And like the walking track with concrete and maybe a tennis court [laughter]. But, yeah, just a couple of things like that, that are weatherproof...

Interviewer: Yes, that makes a lot of sense, yeah. Give you some options. Now the last area is about safety and we have touched on this one. If I were to ask you about safety in your area, what sort of things would you tell me about?

Respondent: No footpaths near the road. It's a bit scary for them on their bikes.

Interviewer: That's the main road?

Respondent: Yeah, well, any road. We don't have footpaths.

Interviewer: Oh, you don't have footpaths in your area?

Respondent: No. So we're just on the side of the road. So we go on the opposite way of the car. But they're riding bikes or skateboards sometimes, and it's a 60 zone, but that's still quite fast I find, for a lot of people.

Interviewer: Absolutely.

Respondent: But I think that's a big safety thing. And it being dark, I suppose.

Interviewer: Lack of lighting, and dark early.

Respondent: Yep.

Interviewer: OK. Does the safety influence whether you're physically active or not?

Respondent: Yep.

Interviewer: Yep, because you were saying that there are some areas that you won't walk into at night, you know. Does road safety influence whether you're physically active or not?

Respondent: Yep.

Interviewer: Yep, so you stay off the main roads and try and find sort of more back routes that…?

Respondent: Definitely. We've got a lot of trucks.

Interviewer: There's not much room when there's big trucks. If you wanted to be more active, are there personal road safety issues that, if they were addressed, would help you be more active?

Respondent: Yep.

Interviewer: Yeah. Which is basically just…be the footpaths.

Respondent: Well, the speed not so much if you've got footpaths, you know? Because then everyone's not sharing the same bit of road.

Interviewer: Yeah, that's right. And so where you are, you really need to have some restrictions. Well, ideally you shouldn't have to compete with cars in that way. OK, so just before we finish, is there anything else you'd like to say about where you live, or being active, or your physical environment that you haven't had a chance to, today?

Respondent: No not really.

Interviewer: Thank you very much.

Date: 1 November 2011

Duration: 45 minutes, 4 seconds

Interviewer: [interviewer]

Transcriber: [transcriber]

*OK. I think we're on. OK, so today what I'm really interested in is hearing your thoughts on a range of issues related to what it's like to live where you live and how this might relate to your physical activity. So keep in mind there is no right or wrong answers. I'm just really interested in your experiences, your thoughts, your opinions. Um, so if there is anything you're unsure about, just yell out. Anything you don't want to answer you don't have to, and also you can stop the interview at any time if you need to. OK. So what I thought I might start off by asking about is if you could tell me a little bit about where you live. So for example, what's it's like to live there, what your property is like, how long you've lived there. That kind of thing.*

Right. I live on a farm 3km from the nearest town, which is [town]. Um, my home is a [year] building, it's got an old house which is rambling, um needs a repair – like a lot of farms. The garden needs a lot of work. The farm itself is a mixture of hills and flatland. And the farm is virtually in the middle of it.

*Yeah, OK. And so roughly how much property do you have?*

About six and a half thousand acres.

*Wow. OK. That's a big one.*

Not really.

*It is when you live on a small suburban block like I do. OK. And so how long have you been there XXX?*

I was born in [city] and I've lived here all my life. Other than that I've been away studying.

*Yeah. OK. OK. And so the reason you sort of live in [town] is because you've grown up there?*

No, my family business is here. And I was sort of induced by my family in becoming involved in that and now run it for them.

*Yeah. Sure, OK. And so roughly how far is it for you to get to the places that you need to go regularly? So to get into town for example.*

Oh 3 km. Five minutes. But I don't go to [town] township very often. I go to the city usually once a week.

*Yep, so that being [city].*

[city]. That's my nearest city. That's about an hour and ten minutes.

*How long does that take you sorry?*

An hour and ten minutes.

*Yep. OK.*

And I travel interstate a lot.

*OK. That's for work or pleasure?*

Work. I'm away up to three or four months of the year.

*OK. Righto. OK. And so what's it like to live around where you live? Do you enjoy living there? What, if someone came and asked you "what's it like to live here in [town], what would you say?"*

Um, what would I say? I'd say it would be much nicer if it were not for work pressures. The area is um very cold in winter and I find that a bit of a chore. And in the summer it can get pretty hot and dry, and not so pleasant to be working outside, which is what I do for a living.

*Yeah, of course.*

So it isn't the best climate. It's probably one of the more difficult climates in [country] to be farming, with the type of business that we are trying to run.

*And what do you farm?*

We've got a mixture of things. We've got sheep and cattle and deer and we've got a lot of intensive crops.

*Yeah, OK. What's probably your main one? Have you got one that is sort of …*

Yeah, sheep are probably our main enterprise. But poppies represent probably about 40% of my income.

*Yeah. OK. Alright.*

There's a very intense period of the year – for about six months it's all poppies. The rest of the year it's all livestock.

*Right. Yeah. OK. No that makes sense. Alright. So when we've been talking to men that live in cities, urban areas, what they often refer to is sort of their local neighbourhood. And so what I'm interested in sort of talking to men like yourself is*

*asking what the term "neighbourhood" sort of means to you? Does it mean something in terms of where you live?*

To me it doesn't, really. It used to. But I feel that the sense of local identity has been diminished with council amalgamations and things like that. And also in the disputes that have occurred in the last twenty years in the district. And so I don't have a sense of neighbourhood or community anymore.

*Sure. OK.*

I used to have, but I don't feel that now.

*OK. So it was there once but it's not anymore.*

And it's becoming more and more diminished as you get transient populations, as you lose your local facilities. The points of meeting or where people congregate have really gone for me. Um, some people, they would see a hotel as an area where they might meet and congregate, but I don't drink much, so that doesn't suit me. So really I look for my friends outside the district now rather than within it.

*Yeah, OK, that makes sense. So the next question I was about to ask was about sort of the word "community", which also sort of gets used a bit interchangeably with "neighbourhood". And I just wondered you know if that was different to neighbourhood or does that sort of mean the same thing to you?*

Well I've lived in the city as well, and no, it's quite different. And while country communities can be closer, they can also be very distant. And when they're not working, if they become dysfunctional they become quite ah, distant.

*Mm. So you mean distant sort of socially?*

Yes, socially.

*Not just geographically.*

Yeah. The actual travelling times are – for instance, I see my neighbours more socially in the city than I do in the country. Like I see them at a party or a function or something in the city regularly. But on the actual, in the district, I would not see them more than once a fortnight. And I don't think it's an unusual position for people living in the bush.

*No. I think we've actually heard that from someone else up your way as well. So it's um, yeah I think it is a common theme. OK. Alright. So now we are going to talk a little bit about physical activity. And what that means to you? So can you tell me*

*what the word or the term "physical activity" actually means to you? What do you interpret that as being?*

Well I think of it in terms of the effect it has, rather than just what is physical activity. I think in terms of you know work fitness as opposed to cardiac fitness.

*Sorry, you cut out a little bit there – as opposed to ?*

Cardiac fitness.

*Cardiac fitness.*

I think they are quite different.

*Yep. OK.*

I consider myself to be quite work fit, but not cardiac fit. Um, for instance many people couldn't do the jobs that I do all day for the protracted periods that I do. But I could not play tennis without getting pretty worn out, that my friends in the city could. I think they're different levels of fitness and different types of fitness. And the physical activity that I have, um it comes in bursts. I'll be walking around hills mustering livestock, so that's quite active. And then I'll be sedentary. I'll be sitting in vehicles supervising people or driving tractors and things, which is quite sedentary. And so it's quite a contrast from one thing or the other.

*Mm. Interesting. Alright.*

It's much more sedentary than it would have been ten years ago, twenty years ago, thirty years ago, because of time pressures.

*OK. So what way is that? So it's mean that you're busier?*

It means that you haven't got time to walk livestock. You need to use a motor bike or drive. And with the irrigation equipment, whereas in the past I would walk along with a spade and irrigate with the old conventional bays, now you've got travelling irrigators that need you to get up all night and service them run around.

*Yeah. OK.*

So it's just, it's like all people, we are all becoming time poor even though we've got more time saving devices.

*So it's some of these technological advances have sort of changed the way you do things?*

Absolutely. They've changed the way – we are much more efficient. But that comes at a price of less physical activity.

*Yeah. OK. Alright. Well we are going to talk a little bit more about physical activity at work and those kinds of things in a minute. But I will just ask sort of in terms of physical activity, I think you've touched on this a little bit, but how do you see it fitting into your life? Is it sort of something that you know, you make a priority? Is it just something you do? Um, something you do at work? Is that …*

I don't really know how to interpret what you are asking me? Um,

*This might be a little bit of a – maybe more of an urban concept, in that often um –*

People set time aside for activity. Do I do that? No I don't. I used to. But I don't so much now. In the winter I tend to more than the summer. In the summer I'm just so time poor. Daylight savings has been the death of physical activity for [job].

*Really, why is that?*

Well we get up so much earlier and we have just got to, we work from daylight to dusk. And so the time is not available. And it just means the day starts an hour earlier. In the past you might have had times to do other things, but with daylight saving that's all disappeared now because we just work those extra hours. And of a night when other people decide "well look it's 5.30 or 6 o'clock or whatever, I'm going home", well [job] like me work until you can't physically see any longer.

*Yeah. Till the sun goes.*

That's right. And then you head home. So it's half an hour to get home or an hour or how ever far you have to travel. And so I'm rarely inside before 8 or 8.30 in the summer. Usually more like 9 o'clock.

*Yeah right, long days.*

They are long days.

*Yeah. OK. Alright. So we're going to talk a bit more about physical activity in a bit more detail. So in sort of um the work that we do around physical activity, we often think of it as any activity that you are doing that lasts for about at least ten minutes, and um that's related to the evidence around health benefits, and it also sort of causes your body to work a bit harder than normal. You know, your heart rate goes up, you might huff and puff a little bit, you might get a bit warmer. So thinking about this sort of, this type of physical activity in the last two weeks or so, um again you've touched on this a little bit but I'd like to talk about it a little bit more, have you been um active at all at work for at least ten minutes at a time?*

Oh yes, frequently.

*Yep. OK. Tell me a little bit about, you sort of touched on that it sort of comes on and off, and sometimes you are sitting, and sometimes you are doing harder work. Do you tend to, when you are being active does it tend to be sort of moderate intensity or is it a bit harder, a bit more vigorous?*

It can be either. Usually it's moderate to hard. If you crutch sheep, then you'll have an 8 hour day where you don't get a break. It's hard physical activity that whole day. Then at other times you might be just walking up a hill and moving, mustering livestock. Moderate activity. And that might be for an hour or so at a time. And then you'll have periods when you won't have much activity at all. It isn't regular or structured.

*Yeah. OK. So over the course of a week or so it could vary from day to day, week to week.*

Oh absolutely. From hour to hour.

*OK. Yep. Um, and you mentioned before it changes a bit depending on the season and the time of the year. Can you tell me a bit more about that, is that related to what you are growing or what you're focussing on at that time?*

That's right. In the winter, the days are shorter. It's very difficult to do very much before quarter to seven in the morning because it's just not daylight. And at five o'clock at night it is starting to get pretty cold and unpleasant outside.

*Yeah. Yeah.*

So you tend to, your day gets compressed into that time frame. But in the summer you don't have those restrictions and so you just work whilst it's daylight.

*Yep. You just keep on going. OK.*

And your whole year's income is really determined by the work that you do in the spring. And so this is a big financial incentive to get in and work really hard in the spring and the summer and autumn because that's when you make the money.

*Yeah, that makes sense. That makes sense. OK, alright. Well, now some of these other questions I'm going to ask you may not be relevant because you are clearly doing so much work, but I'll go through them anyway and if the answer is no, that's more than fine and we can leave it there. In the past week or two have you walked*

*or cycled to get to and from places? So have you sort of walked to get to the local shop or cycled to somewhere?*

Yes.

*Yeah, do you want to tell me a bit about that? Where you've been and what you do?*

Oh just moving livestock. Walking, taking sheep from you know, I would probably walk an average of three to five kilometres a day, moving stock. And in other days I'm so busy that I sit in a vehicle and do it rather than walk. Like as I've said I've actually been walking more in the last couple of weeks just because it suited our enterprises better.

*Say that last bit again sorry?*

It suited our enterprise better, walking rather than driving.

*Yeah. OK. Alright. That makes sense. OK, so the majority of that is on your property – are you doing that on your own? Or have you got colleagues and things with you?*

Always by myself.

*Yep. Always by yourself. OK. And does that walking sort of change, again depending on the time of year?*

Yes, it does. In fact I do probably as much in the winter as in the summer, the actual walking. Because at that time of year you've got ewes lambing. And so you are walking around those. Because usually it's too wet to drive anyway.

*Yeah. Right.*

But it's cold and it's actually quite nice to walk on those days.

*Yeah. OK. Are they nice crisp days?*

That's right. Whereas in the summer it's just too jolly hot.

*OK. Thinking around the house, so maybe inside the home, is there anything, any sort of activity that you do there that is sort of you know at least ten minutes? Do you tend to do sort of chores and …*

I've got an exercise bike and I use that intermittently.

*OK. Yep. Sort of what, sort of inspired you to do that? Or when do you do it?*

I sort of get too fat. I give myself a workout for a month or two and then I seem to lose interest again.

*Yeah. OK. Do you sort of tend to have a bit of a go at it for a couple of weeks and then sort of lose it?*

Yeah, it's usually probably up to a month or six weeks, just til I get back to a weight that I feel more comfortable with. I just eat and drink too much again.

*Yeah, those things are um, very appealing. Um, OK. And you normally do that on your own? It's not with someone?*

No, never with anyone.

*Yep. Doing it on your own. Yep. And is there any sort of thing else say in your, I guess in your leisure or in your discretionary time if you have any that you do? Any physical activity?*

I don't have very much leisure time at all. But I like to go, my landlord [14:42] has got a swimming pool. I like to go there and swim when I can. And I used to um, er, play tennis with friends nearly once a week. That, over the last several years that's just been, you know work pressures have stopped me doing that.

*Yeah. OK. No problem. Yep. Alright. I guess if there is anything that, you know if you wanted to do more in your leisure time..*

Oh the thing that would make an enormous difference to me – and I've tried to arrange it is if it's possible to swim in the morning. There' s a swimming pool locally, and that's the case in most towns, but it really doesn't suit me to be doing it during the day or of an evening. But from sort of 5.30 in the morning until 6.30 I've got an hour that I could go and swim. But that, I think that would be a wonderful opportunity if that were made available to not just men, but women, who work. And it wouldn't really take a lot of organising.

*And it's not open that early? Is that …*

No, it's not open or it's not heated enough. In other words it's cold and no-one wants to swim there anyway. And I think that's one thing that could really significantly improve the health of sort of middle aged men anyway in this district.

*Yep. You think there would be a bit of interest?*

I think there would be uptake. I know people that would like to do it. But they just can't be bothered with all the kids of a night, and at 6 o'clock in the morning you're not going to get many kids there.

*No. Not at all.*

And it's a time of day when people can set time aside to do some exercising. And I think people would get a lot of benefit from it.

*Yeah. So it's probably what's stopping you though is that it's not open and ..*

It's not open at the hours that you would like it to be. And it's not user friendly. My daughter actually works for the [workplace], and does that pool. Over the uni holidays she looks after the pool for the local town. But the worst thing is that they don't open until 11 o'clock in the morning and usually till 9 or 10 at night. Which is really good for kids after school and it's good for people who have got time during the day, don't work. But they are not really user friendly hours to people in the workforce.

*No, no, thats…*

Particularly people that work the hours that most farmers and people who work on farms, or work in country areas work.

*Yeah. Sure. Is it an outdoor pool or an indoor pool up there.*

An outdoor pool and provision was left to heat it and cover it. But council have decided not to pursue that because – anyway, that's another matter. But my view is they should.

*Yeah, do you know why they've decided not to?*

It wouldn't cost a lot for the health outcomes. Beg your pardon?

*Do you know why they've decided not to heat it?*

Oh it's [17:10] – he'd rather spend the money on a man looking after a rubbish tip.

*Yep. On something else. Yeah. OK. That's really interesting. Alright. So we have sort of covered off your physical activity. I feel we've sort of talked a fair bit about that. But now we are sort of going to talk a little bit about, a bit more about where you live and how it might influence what you do. Um, so I guess when I talk about the physical environment, what I'm getting at are all the, you know the things around you: roads, trees, houses, shops, traffic lights if they're around, that kind of thing. And some of these things, people that live in urban areas have sort of told us these things are important for their physical activity, and what I really want to sort of talk to you guys about is, is that also relevant to you? Or are there other things in your environment that sort of affect your activity or not? So I mean, I guess the question here is sort of can you tell me a bit about how easy or difficult it is to be active in your neighbourhood, or your area? And you've talked a little bit about that in terms of the pool. But are there other things out there in your sort of local community that um,*

*would sort of make it easy or difficult to be physically active if you wanted to be more active?*

I think if you wanted to be more active, then the options would be there for you. Like bikes, people run. The country is nice and flat, so it lends itself to getting more exercise. The things that detract from it are that on the country roads you got log trucks there that are pretty jolly dangerous. And I wouldn't let my kids ride bikes and things like that. Trucks I guess are just not aware of cyclists and don't want to be aware. And so there are some traffic issues on country roads that make them less friendly than they might be. But other than that, um really it's a lack of time, and a lack of willingness. Like you can decide to do anything if you make your mind up to it. But it's um motivation I guess.

*Yeah. OK. As in for you personally, or for people in general?*

People in general. I'm thinking about all my friends and peers. I can't think of any of them that really go to much trouble to exercise, other than when they retire and move to the city. And feel as though they need to.

*Yeah. OK. And is that generally because they've stopped doing their physical work?*

I think because the time is available. I just know with my mother, I mean once time was available she was able to do things she had always wanted to be able to do. But I think the lack of time is an issue. I think there are facilities that could be made available. There are things like gymnasiums at schools and things like that. But there aren't the social support networks, particularly for men in the country. How well they'd be used, I don't know. But I know that – I really, most of the health system has been very anti-male. I think it's actually structured, and I feel very strongly about it, I've written to the paper about it I feel so strongly about it. I think that the health system, you can always mend health issues in general, they're not well catered for the way the medical system operates.

*OK. So in what way? Just out of interest.*

Out of interest?

*Yep.*

It's nothing to do with this survey, but basically if you ring the Men's Health Line, 9 times out of 10 it's a woman that picks the phone up. You know, there's an immediate um, and you are asked "what is it you'd like to discuss?" which is a barrier

– men don't like to talk about personal issues generally. Um, there are social stigma attached to most mental health issues, particularly amongst country people. They are still like twenty years behind urban areas in terms of acceptance of you know, the bell curve of people's activities. I think that's an issue. Also if you look at the protocols for – men don't fit well into a structured health system. They need longer consultations if they see GPs, and most of my friends don't. Um, they need to be um spoken to differently and treated differently. And it just doesn't happen because the system is under pressure. And also there's not an appreciation of the buttons that need to be ticked for men, who aren't familiar with the health system.

*Yeah. OK. Yes, it's really interesting.*

I could write a book about this, eh?

*Yeah. You could. Look I could talk to you about it all night. Yeah, it's quite, yeah, it's fascinating.*

Well, I had trouble with melanomas. Go along to a, I had them removed, and yet there are no male practitioners in the clinic that I go to for skin checks. That's a bit bizarre. 72 medical students now are all women. So it's not going to improve. I don't feel my needs are being met at all with the GP that see. They don't respond to the questions I ask them, I get very poor answers. And I'm not unusual.

*Yeah, OK. Interesting. So I mean yeah, we could talk about this all night.*

We could, couldn't we?

*I guess it's a side track to hear what you, you know…*

You've got all the boxes to tick. You've got to keep ticking boxes.

*Yeah, I'll do my best. Alright. So we might, I'll get back to my sort of questions and we'll keep chatting and see how we go. But um, so I was going to talk to you a little bit about availability and accessibility of places to be active in your community. And you've touched on this a little bit, but I'll just ask it in case there is anything else you think of. So I guess if I was to ask you about the places in your neighbourhood that are available to be active, what sorts of things would you tell me about? So you've told me a bit about there's a pool. Are there other sort of recreational facilities or …*

There are but they're not structured towards community use. They're for like a football game, or there is a court that can be used for playing school tennis. So they're aren't those sort of facilities that are generally publically available. But that

doesn't mean that they couldn't be if people were of a mind to try and structure it so that they could be. It's just that there isn't the infrastructure there and there isn't the mindset to, to um, - like I know the local women's group where they go for walks every Friday afternoon sort of thing. And I think that's a terrific idea. So that's well catered for. I think women are better catered for in terms of structured activities and get fit classes. All that sort of stuff. There is actually nothing like that for men. That I'm aware of. They probably wouldn't go anyway. But if they were there, then it's very hard to argue that the opportunity is not there.

*Yeah. OK. What sort of things do you think just out of interest would men be interested in? You know you are saying the women's walking group and that kind of thing, but you know if you offered the same thing for men they wouldn't go. What would men, what would get a bloke in?*

Well I think it needs to be a very different thought process. I find the thought process of the men's shed and where men's health is – there's an analogy drawn with motor vehicles – is really condescending. If there were those analogies drawn for women's health where you've got things like kitchen appliances there would be an outcry.

*Yeah, OK. There would be.*

Dreadful, dreadful. You couldn't get anything more sexist. Tell me something that's more sexist than you know an analogy to a motor vehicle. And so I don't know whether that's unusual, but I find it offensive.

*I hadn't heard that before. But yeah, I think you've got a really good point.*

And a lot of other men do too. I go to a luncheon group once a month. And we get a few beers into the men, get a few wines into them and you start to actually hear what they think, rather than the façade. I find that very interesting, because I actually don't drink much because I've got to drive home.

*Yes. Occupational hazard.*

So it's very, I almost feel like an observer, hearing the way that people within sort of twenty years of age think about health issues. It's been quite fascinating really.

*I wish you had a little recorder that you had been taking with you!*

I'd never be asked again.

*That's true.  Alright. Um, so getting back to these sort of places in your neighbourhood that you know you've mentioned the pool …*

Well the topography lends itself – because there are nice places you can go for a walk, there are rivers, all those sort of things.

*Are there walking tracks and cycle paths and things?  Or is it really just sort of walking on the side of the road?*

No there aren't.  My farm is big enough that I could do that anyway if I chose to. And we have people that come here just for exercise.  I've got fellas who come here from [city] every weekend just to go, they play hunting but they never shoot anything.  It's just for a walk in the bush.  And there are old couples who come here to ride the pushbikes you know.  One couple comes once a fortnight from [suburb] – they've kept their bike and they come up here and have a Sunday afternoon ride.

*Oh there you go.*

And is doesn't hurt anyone and they love their day out.  It doesn't really matter what the weather is like.  If it's raining they still do it.  So I think it's not that the opportunities aren't there.  That calls for direction [26;09] – and I think it's so easy to become bogged in the day to day minutiae, that you don't address the big picture issues in life.

*Yeah.  OK. Yep. OK. Alright.*

I haven't been much help have I?

*No, it's fascinating, it's really interesting. We talk to all different people with all different perspectives so you know, it's certainly contributing plenty of um, really thoughtful contribution I think. Um, but I will keep moving along this thing because I don't want to take too much of your time.  I know you are a busy man. So I've just got a few more questions to go.  Something I wanted to talk to you a bit about was um, this idea of um destinations, so often we are getting men that live in cities and things talk to us about this idea of you know, having somewhere to um, walk or cycle to or from. You know, it can sometimes inspire them to be active. So instead of driving they might go for a walk or whatever. So I guess I'm interested to know whether having …*

I think that's really good. In my case, I go bushwalking, a lot of my friends two or three times a year we just go for a wander and go camping for a couple of nights.

And I think that's a really useful thing.  I think that there could be opportunities for that to be more organised, for some people that like to do things like that in a group. And there aren't any opportunities like that. And I think that it's a shame. Because we live within very close distance to some wonderful wilderness areas.

*World class. Yeah.*

And yet, so much of it is wasted. So I think, yes certainly there are opportunities that if it could be organised.  And I think certainly [27:43] – I've got friends that ride from [city] up here on their bikes about once a month.  And they do it individually. And I've always wondered why they don't sort of do it together.

*Yeah, why don't they?*

Well I think it's just that they are, again a bit like me. They are time poor. I've got six hours, I'm going to ride to [town] on my bike, have a nice lunch and coast home.

*Go home again. And they go when they can go and go at their own pace.*

That's right.  And when they're in the city, they live in [city], they'll go for a ride up the mountain or ride down to [town].  They understand that they need to get the exercise. But they don't do it in a structured form and they don't do it in groups.

*Yeah. OK. And it sounds like, I guess you know some of the messages that are out there around physical activity that we should be doing a little bit everyday.  It sounds to me like these people it's more like a weekly or a monthly thing. It's a bit more sporadic.*

That's right. It's almost a weekly [28:32] Or they might do it a couple of times a week, but usually just weekly.  Or even fortnightly.

*Every couple of weeks. But when they do do it it's a really big you know, like you say a big six hour ride or a three day walk or whatever.*

Exactly. And they [28:48] for a fortnight afterwards.

*Yeah.*

I do anyway.

*I don't think I'd make it. OK. So um, thinking about sort of, I guess, you know places to go.  So thinking about you and your physical activity, I know we've talked a lot about your work activity but I guess thinking about you and where you live and [town] and whether, I guess, does having or not having sort of places to go influence whether you're active or not?*

No.

*No. OK.*

No, because you can make them. It's all your mind. You can decide where to go. And I also realise that sometimes, I gosh I need to get a bit more exercise than I've been getting, so I'll leave the vehicle and walk rather than take the ute somewhere. It's just a conscious decision that it's what I need to do.

*Yep. That makes sense. Alright. OK. Now I'm going to ask you a little about the um sort of built or man-made features of your neighbourhood and your area. So we talked a bit about the topography and the landscape. But um, I guess you know things that are you know like your paths and your footpaths and your lighting and that kind of thing, um do those kinds of things influence whether you're physically active or not?*

Well I don't think they do because I don't live near enough. They are 3 or 4 km away from where I live. I really don't have a park to play, I kind of avoid the roads, traffic.

*When you are walking or driving?*

Yes, yeah. When I'm walking or riding a pushbike. Again I try and do it quietly rather than – the main roads are just um, really not so friendly. Particularly because they are narrow and gravel.

*I was going to say what's not friendly about them? Is it that they're – so they're narrow?*

Well that is one of the things. And also that there is not an appreciation by the – log trucks start early in the morning, finish late at night and drive too close to people. Just really have a poor sense of social responsibility. Um, not all of them, but a significant number. Enough to make it so that it's not really safe.

*Yeah. OK. And is there a like a, is there a shoulder?*

And I'm someone who is actively involved in forestry but I think these fellas really need a wake up, but …

*Yeah, OK. That's a really interesting thought. Are there um, are shoulders or anything like that on your roads?*

Oh yes, because they're all gravel roads. It's not an option to walk beside the road because they are very poorly drained, and there are ditches and long grass and snakes.

*Yeah, not very nice.*

Well no. Not conducive to – not for walking on the road.

*No, fair enough. Alright. And so um, moving up to the next one, wanted to ask you about the sort of aesthetic qualities of your neighbourhood. Or the attractiveness of your neighbourhood. So I guess you know if I asked you about that, what sort of things would you tell me about? Is it an attractive area or, in your opinion?*

Um, depends on the time of year. I think all [state] is very attractive in the autumn, but I think it looks pretty bleak in the middle of winter and pretty ordinary in the middle of summer.

*Is that because it's so dry in the summer?*

That's right, it's very dry. And exercising then can be pretty unpleasant because it's, we don't get sea breezes until late in the day. Um, and it gets hot early and in the winter time it's pretty bloody cold.

*Yeah, OK. So does, I guess does the aesthetics of your sort of neighbourhood or area influence whether you are active or not?*

Not really. Because even if I'm in the city I'll get up in the morning and go for a walk just to get some exercise and it doesn't really matter to me what the countryside is like. You just almost turn off.

*Yep. Yep. It's all the same.*

Mm.

*OK. Um, and just following on from that, if you did want to be more active, which it sounds like you are plenty active enough already, but are there features of your neighbourhood that might help, or if they were a bit more attractive or aesthetically pleasing, or are you quite happy with it all the way it is?*

I don't think I'd fit in terribly well. And I don't know that I'm a good representation of how other people think, because I'm just not 'urban', as a person who lives out of a town, it doesn't influence whether I exercise or not.

*Yep. That's more than fine. And like I said we have sort have taken a lot of these ideas from people that do live in urban areas, and I guess we are just trying to get a sense of you know, are they relevant for people in rural areas? Which, my suspicion is no. And that's coming through loud and clear –*

Really? Oh right.

*Well not so much no, but there are a lot – a lot of the issues are very different.*

Are they? Right.

*Yep. Well I think you've sort of highlighted that just by saying things like whether it's attractive or not sort of doesn't really influence you, whereas people –*

It doesn't at all, I mean it's all in your mind isn't it?

*Yeah, whereas we do hear different things from people in the city. Alright. So one last thing I wanted to cover off was this issue of safety – so if I was going to ask you about safety in your area, what sorts of things would you tell me about?*

Safety in exercise you mean?

*Um, I guess just in your general area. I mean you could think about either road safety or personal safety. Um, either one of those things?*

We are very badly served in terms of opportunities for people who have hassles exercising. There aren't physiotherapists nearby, there aren't good access to GPs, those sort of issues. So if people have things, I think if we can find an excuse not to exercise, generally you will. And yet minor improvements in health can make exercise not just possible, but quite enjoyable. And people in the country don't have access easily to those services. I think that would make the difference. But in terms of safety, um certainly things like there are no – like the swimming pool. There is only a pool, the public pool in the town, and if people want to swim in rivers and things, they are pretty dangerous places. There's a lot snakes, there's shallow water, there's deep water, there's cold water, there's [35:05] obstacles. There's a certain transient population, so I don't think we would have the same degree of issues around feeling safe, running at night for instance. There's unlikely to be muggings and things in country areas. It could happen but not so frequently. And so no, those sorts of issues aren't really, I don't think, a barrier to exercise.

*Yep. So some of those things that you were talking about before, the creeks and the rivers and the snakes and that kind of thing, do you think that influences you or people that you know in terms of being active?*

Oh absolutely. I know a lot of people, particularly women here, don't like walking along the river bank um, in the summer much at all, simply because of coming across snakes. And I know that is certainly like a barrier to a lot of people walking. And long grass, snakes, that sort of thing. And also some of the better walks, if you are

doing it alone, can be pretty hazardous. Because people tend not to, if you are only going to go for a walk for two or three hours, to take any food or something. And yet it wouldn't be that hard to come unstuck.

*Yeah. OK. Yep. OK. So do any of these sort of things influence you, and what you do.*

No. No, not at all. I'm just lazy.

*No, but you walk around all day.*

Yeah, that's right. I've been thinking about this since this survey you know was in the newspaper you know. What reasons do I, do I not, and I often bounced it off my friends, "why are we not exercising? We're just lazy bastards." Enough! We're just too bloody tired to exercise. And so it's, I think it's a combination of all of the above. And unfortunately, the exercise we get doesn't give us good cardiac fitness. It gives us good work fitness. I can [36:36] things around all day. But, and so cardiac fitness I think is very important as well to our long term health options.

*Yes. Certainly. Um, although you know, it's probably better than, what you are doing is certainly better than ..*

Oh than being sedentary.

*Than being sedentary, so..*

Oh absolutely. Yes, I agree with that. But it's only half the story. And that's where a lot of men become unstuck. They think they're fit because they can work all day. But in fact, it's um, it's not the whole story.

*Yeah. OK. Alright. Well that's about the end of my sort of formal questions. But I guess before finishing up, is there sort of anything else you'd like to tell me about um, you know I guess in thinking of how and where you live and the physical environment, and whether it affects your physical activity, anything we haven't covered in those at all?*

No. I think the great strength in what you are doing here, you are actually asking people. And I think what it will do is it will make people first of all think about it and then a lot of good can only come of that. It's a wonderful thought process to actually ask people for their views, rather than saying this is what we think is happening.

*Yeah, yeah.*

And if go through the process that you are going now, that's absolutely terrific. What it will do is, first of all I'm sure you will develop a picture very quickly when you speak to thirty people. And from that only positive things can happen. I think it's a great thing you're doing. Because you're asking rather than telling.

*Oh thank you. That's good to hear.*

And I think that augers really well for getting positive outcomes.

*Yeah, that's really nice to hear that. Because I guess what I really hope that is that by talking to people we, you know, we are hearing what the needs are and then we can, you know, make recommendations and that kind of thing, about perhaps things that the – you know whether it be the local council or whoever may be able to do to help the guys in your area get more active.*

Certainly, for some people that will be a really positive thing. I think some men like the Men's Shed concept. That's perfect for them. They like the sense of community, they like the being close to other people. And so I think great – there might be 15% or there might be 60% that [39:08] to fill that need. Would it fulfil mine? No. I've got different needs, but that doesn't mean I don't have them. And it doesn't mean that I recognise them. Now they're quite different aren't they?

*Yeah, they certainly are. Yeah, that's a real, really key question isn't it? How do you recognise you know what your needs are? You know, how do you get to that point where you say "I need to do X"?*

Well I think many of us are aware of what we should be doing. It's the encouragement to actually do them, and to stick with them is the hassle. People talk about sinking into their art, you know health coaching. Some of my friends go to a boot camp thing and find it really, regularly. And find it really useful for them. Because they've got personal trainers, they've got people that can actually encourage them and keep them at exercise thing. None of those options in the country.

*No. Is that because there is not, not sort of the infrastructure and the programs to run them. Is it a supply or is it a demand or is it a bit of both?*

I think it's both. I think it's an education thing. When you do see how well other people respond to having a personal trainer and getting fitter and feel better about themselves and more motivated, it motivates those people around them. They think

"oh yeah, perhaps I should be doing" – it's the leading by example thing that, which is very positive.

*Yep. Certainly.*

There are none of those stereotypes in the country. The country stereotype is the overweight um, you know, sedentary um, living sport through the TV stereotype. And that's not always true but there is a large element of that.

*Yeah. I guess that is something we haven't talked a lot about today isn't it, is sort of more organised sport? I mean is that something in your community that's big or not particularly?*

It's not at all. But it could be.

*OK. In what …*

There seems to be the kids – I don't know how, it seems to become uncool about 17 or 18 years old. And kids stop active involvement in sport other than football or other than something else. Now, amongst some parts of society that doesn't happen, but it does amongst a fair little slab of young people. But not everyone. And some, the really good thing about stereotypes is that they are really true –

*Yeah, OK.*

And usually you can get through to people if you paint the right picture and you set good examples. So I think there is some really positive things that could occur, but there needs to be the will and there needs to be a sense of support for them.

*Yeah. OK. And what about for um, sort of not so much young people but you know for adults and people, you know in your age bracket for example?*

Well that's exactly who I'm thinking of. You know I think of friends who have gone to the trouble to get fitter and they are all much better for it. All happier. Yes they are more time poor; they are often doing it at the expense of sleep, usually early in the morning. Or they don't get any time to themselves at the weekend. Most of us don't anyway. And they are so much better for it. And I think there is a flow on effect from that. But it needs to be um, it needs to be handled the right way. And I think so much of the language is important in getting the right outcome. And that's where I think a lot of the Men's Shed stuff has been not as good as it might have been. And where there's you know analogies with motor vehicles and men's health is a really bad one.

*Yeah, that's interesting.*

A very poor stereotype. And if you looked at it, this is –oh probably 30% of my mates are GPs or my brother-in-law is a surgeon, and I've got a lot of medical friends – and one of my friends highlighted to me that men's health in [country] comes from a paper written by two women in [country].

*Right. Seriously?*

Now this is not good. This is not good. It does ah – there's a lack of empathy in the way the system works I think towards men's health at the moment. And it can easily be fixed. It's just a matter of a bit of communication.

*A bit of reorientation.*

Yeah that's right.

*Yeah. Well we need to ..*

But thanks very much. What I'll do is I'll send you a um list of people that you can contact with their phone numbers.

*Are you sure they're OK for me to contact them? I just sort of ..*

Yes. They are all expecting you to contact them. They'll be most disappointed if you don't. And some of them like – [name] – I'll just put a note that he has got an 18 year old staff member who he thinks would be involved. [name] is a fellow that works for me and he is 27 and he would like to. My son [name] is 19.

*Oh brilliant.*

I've badgered [43:42] him into it.

*That'd be great.*

When you talk to these people and you get their ages – what I've tried to do is deliberately get a like a 10 year age spread in them.

*Thank you.*

So 70, 40, 50, 30, 20, 19 ..

*Excellent. Look [name] that's been…*

Thanks for taking the trouble to respond to ..

*Oh thanks so much for your time. We are incredibly appreciative. I know it's um, yeah, it's just, it amazes me the generosity of people like yourself who will give up an hour of their time to have a chat to us. But um, look hopefully -*

It's in my interest isn't it?

*Yes.  Well hopefully. What we'll do is in a few months we'll probably send you a little summary of our findings.*

Oh really.

*Yes. If you are interested.*

I would be very, most interested.

*Yes. Just sort of the general things that we're hearing from men in your area. And also we are talking to men in [town] and up in sort of around [town] / [town] as well.*

Oh really? Good, now I'll badger the GP in my household and make her think about it too.

*Yes, please do.  I think that's where we've got a fair way to go there. So I reckon um, yeah get in that one's ear as well, and have a good chat to them about physical activity and men's health.  That'd be great.*

Give my regards to [name].

*Yeah, I will do.  Nice to talk to you [name].*

Oh that's fine.

*Take care.*

AUDIO RECORDING ENDS

Interviewer: I'll just start by talking a bit about the place where you live.  OK?  So can you tell me what it's like to live around where you do and your likes and your dislikes?

Respondent: OK.  I live over in [town], near [x] Creek.  And it's very flat.  We're about half a minute from the beach.  So it's, I reckon one of the most beautiful spots in the world to live.  So I love living there.

Interviewer: It's very beautiful.

Respondent: Yeah.  There's lots of places to walk and to ride and we're right near [x] Park as well, which is a great big park that's great for kids and for adults too, to just head off down there.  So we're very fortunate.

Interviewer: And do you live in a house with a house block, or is it...

Respondent: Yes. Yes.

Interviewer: Sort of slightly bigger. Yep OK.

Respondent: Yes it's a house block.

Interviewer: And the length of time that you've lived there?

Respondent: Three years.

Interviewer: OK. And your reasons for living here?

Respondent: Because I like the area so much and I like the fact that it was flat and thinking, my husband's a bit older than I am, so thinking about our older years. Thinking about living somewhere close to town where we can walk to town or ride, we've both got bikes to ride up into town or walk. Just to go and get whatever we need or to... whatever we want to do.

Interviewer: It's very convenient.

Respondent: Yeah. I really liked that idea and I also liked being near the sea. That's really important to me.

Interviewer: So you actually picked to move to [town] and that part of [town]?

Respondent: Yeah, yeah very deliberately chose that.

Interviewer: For those reasons. And you mentioned about the distance into the town centre that was rideable, what sort of distance is it?

Respondent: We're talking about probably four blocks, three to four blocks.

Interviewer: Oh.

Respondent: So it's about a 15 minute walk or a five minute ride.

Interviewer: Very handleable. Very nice.

Respondent: It's lovely.

Interviewer: So in research with women and living in urban areas, they often refer to the local neighbourhood, and I'm interested in understanding what the term neighbourhood might mean for women who live in rural areas and if it's relevant, OK? So what does the word neighbourhood mean to you?

Respondent: I guess for me it means probably the houses or the people that live in the houses, or the people that live in the houses, sort of fairly close to us, some of which we've never met. People in [town] tend to be very quiet people, you don't see them out and about much. They tend to be very, well seem to me to be fairly home based and, yeah so some of them I have never met. So I guess for me that's what neighbourhood means.

Interviewer: OK so if you needed to put a distance around it, thinking about that, what would it be?

Respondent: Yeah probably the people in the next two streets from our house would be our neighbourhood.

Interviewer: OK and what about the word community, what does that mean to you?

Respondent: I think that means any group of people that see themselves in a... with a common purpose, and act together as a group with some sort of common purpose. And I think that's probably fairly well and alive here, from what I've noticed. People tend to look out for each other and if somebody's doing it tough, there are people that will help. So that's good. I think we've got some very good service clubs and organisations who also look out for people and try and help them. So I guess that's what community means.

Interviewer: Yeah and likewise if I got you to put a distance around it what would it be? Would it be the whole of [town]?

Respondent: For community?

Interviewer: 'Cause you're talking bigger than the neighbourhood here.

Respondent: Yes I'd say the whole of [town].

Interviewer: Yep, OK. Now I wanted to talk a bit about physical activity and what it meant to you and of course it's a [laughter] really broad one and it's all... each individual's different how they view it. So what does the term physical activity mean for you?

Respondent: Well I guess it means just ordinary everyday moving around your home, getting things done. But I think it needs also to mean, any purposeful physical activity you take for... or undergo or do, so that you can keep your body moving and well and, yeah a very difficult thing for me to fit into my lifestyle sometimes.

Interviewer: Yeah, yes. So I guess that then the question is do you consider yourself physically active?

Respondent: I'm certainly physically active but I probably don't push myself to the limit where I think I should.

Interviewer: OK so why do you say that?

Respondent: Well I'd like to be one of those people that gets up at six o'clock every morning and goes for a two kilometre walk, but I'm just not that sort of person. Or fits it in somewhere into the day. But I've never been one of those sporty types. So, physical activity for me has always been more of an effort rather than a pleasure.

Interviewer: That's really clear, thank you. Thank you for that. I mean I think you know that's a range of things to a range of different people and we often need to recognise what's within our capacities and what we want to do. Yep that's fine. And where does physical activity fit into your life. Is it of no interest? Is it a priority? High priority?

Respondent: It certainly is a priority 'cause I've seen with different family members, what it means to lose the ability to move and the loss of facility of being able to care for yourself and that sort of thing. So it certainly is a priority and I understand how important it is to keep moving and keep active.

Interviewer: OK so in research when we think about physical activity we think about any activity that lasts for at least ten minutes, that's where the evidence of health benefits are, and causes your body to work harder than normal, so you might be warming up a bit, you might be huffing and puffing a bit or you might feel your heart racing a bit more. So thinking about that type of physical activity in the past two weeks, and I'll go through just different parts of your life, not to segment your life but to just make sure we don't leave things out...

Respondent: Yeah.

Interviewer: So in the past two weeks have you been active at all at work for at least ten minutes at a time.

Respondent: Yes.

Interviewer: And could you tell me about that?

Respondent: Yes, well I'm a [job] so for me we... there's often quite a bit of walking involved in our job and often there's a lot of lifting. So there's certainly a fair bit of physical...

Interviewer: Physical, yes.

Respondent: Component to that particular job.

Interviewer: OK. Now just thinking about the intensity of that exertion, would you call it steady, moderate or vigorous, those two activities?

Respondent: Probably all of the above. But probably steady to moderate.

Interviewer: OK.

Respondent: I mean we don't want to be seen like we're racing around like...

Interviewer: No.

Respondent: As if we have forgotten things. We need to present a very calm appearance.

Interviewer: Yes. Yes.

Respondent: So it’s not, yeah... but...

Interviewer: It’s very important for your clients isn’t it? Yeah. OK so how long would you have done those two activities over the last two weeks, just a rough guess would be good? Like whether it’s every day or... at work or not.

Respondent: Yeah. I’d say every day, certainly every second day.

Interviewer: Yeah.

Respondent: Some days are office based.

Interviewer: Yes. Yes. So for sort of roughly how much of the time of that day, might it be?

Respondent: Depends what sort of [job] it is too. But it’s probably half to three quarters of an hour.

Interviewer: Yep. OK. And that’s during the day?

Respondent: Yep.

Interviewer: And is this what you usually do or is it one off?

Respondent: Yes.

Interviewer: OK. And does it change depending on the season or time of year?

Respondent: Work wise, no. But my own personal exercise is greater in summer.

Interviewer: OK, we'll come to that. But... and that's really important and what women are all saying too, it's a lot easier in summer. So at work if you had wanted to, is there anything that would have helped you be more active at work? It sounds like you're pretty active anyway...

Respondent: No. No. No. It's a good place to be active.

Interviewer: Yep. OK so in the past two weeks have you walked or cycled for at least ten minutes at a time to get to and from places?

Respondent: Yes we've walked.

Interviewer: Yes.

Respondent: Walked down to the beach. But it's not vigorous walking, it's just ambling along 'cause my husband's unwell at the moment.

Interviewer: OK so it's... it wouldn't... well would you rate it as sort of steady?

Respondent: Very leisurely.

Interviewer: Leisurely.

Respondent: Just taking our time.

Interviewer: OK a bit less than... Yep. OK.

Respondent: Yeah, it's not vigorous at all.

Interviewer: Yep. OK and how long would you have gone walking for?

Respondent: Probably an hour. Yeah.

Interviewer: And how often in the last couple of weeks?

Respondent: Probably only about twice.

Interviewer: Yes. And when would you have done it? During the day, on the weekend?

Respondent: Yeah during the day.

Interviewer: Weekend? Weekday?

Respondent: Yeah, weekends, yep.

Interviewer: And why did you do it at that time?

Respondent: Partly to get out of the house. Partly because we just love going... walking along the beach, watching the waves, just seagulls, just...

Interviewer: It's very accessible for you.

Respondent: Yeah, it's very accessible.

Interviewer: And is this what you usually do or was it one off?

Respondent: No we often do that.

Interviewer: And does it change depending on the season or time of year?

Respondent: Yes. Summer. Summer and warm weather, it's much easier to get out. As soon as it's windy and wet I'm, you [laughs] know... stay home, light the fire.

Interviewer: Yeah it's not much fun. Yeah [laughs]. And if you'd wanted to, is there anything that would have helped you use, to walk or to cycle, to and from places?

Respondent: I think it's always important to have good paths or roads to cycle on and to walk on. And we've certainly got them. I know they've put in a cycling track from [town] out to [x] Beach and we've loved using that.

Interviewer: Oh OK. For walking or cycling?

Respondent: For cycling.

Interviewer: OK so you have been cycling.

Respondent: It's a great ride. And you can pop your thermos in your bike basket and some morning tea and ride out there, again sit by beach, tie your bike up and sit by beach. And then ride back. So we're really grateful to the very kind people that made that track for us, 'cause it's brilliant.

Interviewer: 'Cause it's a couple of, it's a few kilometres isn't it.

Respondent: Yeah it is. But it's a lovely ride. It's an easy ride. There's no hills and it's lovely wide paths, so if you met a group of walkers you're not having to stop and get off your bike. It's very well done.

Interviewer: Excellent. We've been, I've been hearing a lot about that particular walk and cycle path…

Respondent: It's good…

Interviewer: It’s very, very popular. OK and in the past two weeks have you been active around the house or yard, so at home, inside and outside…?

Respondent: Yeah, yep. I do a lot of gardening.

Interviewer: OK and I guess the usual housework things as well.

Respondent: Oh yeah. I do my own housework I don’t have a cleaner come in. Much as I’d love one [laughter].

Interviewer: I always think though you always end up having to do a lot of work so they can actually get on with it. ‘Cause you’ve gotta clean up and all sorts of things [laughs]. So thinking about those two activities, could you tell me what you thought the intensity of that was? Steady or moderate or vigorous?

Respondent: Moderate to vigorous when I’m in the garden. Usually there’s a fair bit to be done and...

Interviewer: Yeah, you’ve got a good size garden by the sound of it.

Respondent: It’s not a huge garden but I like it to look nice.

Interviewer: And so for how long would you have done those activities?

Respondent: Oh sometimes three or four hours at a time. If I have a gardening day I really get into it.

Interviewer: And over the last two weeks, how often would you have done that?

Respondent: Probably for two days.

Interviewer: And that's for gardening and for the housework side of it?

Respondent: Oh I do housework all the time.

Interviewer: So that's every day...

Respondent: Yeah but mainly it's confined to Saturdays if I'm working a lot. So, Saturday morning probably three to four hours cleaning.

Interviewer: OK. And when did you do your gardening?

Respondent: Well because my husband's sick, we're both working part time at the moment, so would probably have been through the week.

Interviewer: And why did you do it at that time?

Respondent: I pick a nice day.

Interviewer: Fair comment.

Respondent: When it's not too cold. If it's really cold I just stay inside.

Interviewer: There's not much point is there. And who would you have done those activities with?

Respondent: Just usually myself. On my own.

Interviewer: And is this what you usually do?

Respondent: Yeah.

Interviewer: Yep. And does it change depending on the season and time of year?

Respondent: Yes. Winter always keeps me indoors a bit more. But...

Interviewer: But the housework doesn't change.

Respondent: The housework always has to be done.

Interviewer: And so if you'd wanted to, is there anything that would have helped you be more active inside or outside your home?

Respondent: Not really. No. I think you... it's always personal motivation.

Interviewer: It sounds like you're busy enough doing these. OK and have you been physically active for at least ten minutes during your leisure or spare time? Now we might have already covered this with your walking, but is there anything else that you want to mention? Oh and the cycling.

Respondent: Not really. We've got an art room upstairs, it's a little bit of a studio and I seem to be racing up and down the stairs a lot. It's a fairly steep staircase. It certainly isn't ten minutes worth but you know you might be up and down three or four times...

Interviewer: It's regular enough. Yes.

Respondent: And we've got two lots of stairs in our home that we're always up and down. Which I like 'cause I think stairs are a good way to keep fit.

Interviewer: They are. Thank you for that. Now the last bit's about your physical environment. Now this is about the physical environment where you work and live and play and when we talk about physical environment I mean all the physical things that surround you, so it might be roads, trees, houses, parks, beach. And some of these things are things that

people living in urban areas have said might actually be important for their physical activity. So I wanted to ask whether they're relevant to you. So can you tell me a bit about how easy or difficult it is to be physically active in your area?

Respondent: It's very easy.

Interviewer: And what makes it easy?

Respondent: Because it's flat.

Interviewer: Yes.

Respondent: Because there are good... there's a very good park near us and there are good footpaths.

Interviewer: Yes.

Respondent: I think they're important.

Interviewer: And how about your lighting?

Respondent: Yeah, yeah, that's important 'cause I know that a lot of people do walk of an evening. I tend not to but... unless it's in summer then we're out til...

Interviewer: You're out anyway.

Respondent: Til nine or ten o'clock at night sometimes, out on our bikes.

Interviewer: Oh right.

Respondent: Yeah. Good fun.

Interviewer: Oh OK.

Respondent: But lighting is important. Good access down onto the beach, that sort of thing I think is important. It's... after the storms we had last year I know a lot of our foreshores washed away and so there's quite a drop there now...

Interviewer: Oh really.

Respondent: And so that doesn't make it very easy to actually get onto the beach in certain places. But there are parts that you can get on...

Interviewer: And the other one is that it's very walkable for... your streets connect with each other. It's not like your living on a main highway and it's a strip and that's it.

Respondent: Yep. Yep.

Interviewer: You virtually can walk through...

Respondent: Well even if you're walking up into town there are a couple of main roads to cross but you can cross at lights in once place and it's quite safe to cross because [town] a little bit smaller town, the volume of traffic isn't as bad.

Interviewer: Yep. Yep.

Respondent: On [x] Street, it is in, right in the centre of town. Yeah so it certainly is accessible.

Interviewer: Excellent. OK. I'm going to ask you a couple of areas, and we've probably touched on some of them as we've been talking, availability and accessibility of places to be active, if I were to ask you about places to be active in your area, what sorts of things would you tell me about? And you've already told me about walking tracks, cycle paths, your parks...

Respondent: Beach.

Interviewer: Beach. Anything else?

Respondent: Well there's a river...

Interviewer: Yes.

Respondent: There's a river and that's great for kayaking and boating. So... and we do use the river, we've got kayaks as well. So...

Interviewer: Oh you're well set up [laughter]. Good on you.

Respondent: Yeah, we... before...

Interviewer: And it's easy access into the river?

Respondent: Yeah it's easy access and there's a number of boat ramps that you can access the river on. And even access for, way up the river, you can loads your kayaks in there and paddle down the river which we did in summer. So there are lots of things to do for people who want to do them. This area's excellent really.

Interviewer: And access? Are places convenient? Are they easy to access…?

Respondent: Yes, yes they are very easy.

Interviewer: And does having places to be active influence whether you're active or not?

Respondent: I think so. I think because, because it is easy and because it's not far away, you're not travelling for half an hour to get anywhere, you can just...

Interviewer: Enjoy it.

Respondent: Get straight on and get into it. Yeah definitely.

Interviewer: And if you wanted to be more active, are there things or places that would help if they were available or more accessible?

Respondent: I'd be very interested in doing some things like some more Pilate's classes and things like that. And I have heard, somebody told me that they are here in [town] but I've not seen any advertising or...

Interviewer: You haven't found them.

Respondent: Or found them yet.

Interviewer: It's having something that's available.

Respondent: Yeah. Yeah.

Interviewer: Like sometimes things like this appear and disappear very quickly...

Respondent: They do. Or there are times that you can't get there if you're working all the time. And if you feel you can only get to the odd one or two...

Interviewer: You don’t think it’s...

Respondent: You think “Oh I don’t know if I can manage that”.

Interviewer: Yep. OK. Now destinations. If I was to ask you about places that you would walk and cycle from your home, what sorts of things would you tell me about? And you’ve already talked about that, the shops and the beach, any other places that you might walk or cycle to?

Respondent: We’ve got a bit of a funny thing in our family where, ‘cause we all get together and do these bike rides in summer and...

Interviewer: Oh OK so a bit more of the blood...

Respondent: Oh yes, well my sisters and that and we’ve all got bikes and their husbands and kids...

Interviewer: And they’re all in this area?

Respondent: Yeah and well they’ve got caravans in the caravan park that’s just over the road from us. So we all get together in the summer. And we always have this big ride one evening where we ride round to the cemetery and we [laughter] visit our grandparents’ graves. It’s just something that we’ve done.

Interviewer: Oh wow. OK.

Respondent: Just as a commemorative thing I suppose. Just a...

Interviewer: Yeah, so you’ve got a longer link with [town] area?

Respondent: Oh yeah. Yeah. So that's...

Interviewer: That sounds a great thing to do.

Respondent: And we often stop off at [restaurant] and have an ice cream and then keep going and we do... we call it the big ride [laughter].

Interviewer: So does having destinations influence whether your active or not?

Respondent: Well I think this funny little ritual affects us because we say "Oh we've gotta do the big ride yet" and we plan a night where we're going to go and do that.

Interviewer: Doing that and that's a good example of yes it does.

Respondent: And we just... I think it's just really another excuse to get out and do something together and...

Interviewer: It's nice to have some...

Respondent: But usually every night while we're all camping in the park, we do big rides, we ride all over town, and all... yeah big rides.

Interviewer: Oh OK.

Respondent: We're not sort of, you know dressed in lycra or anything like [laughter] that, but it's just fun...

Interviewer: Yeah.

Respondent: It's fun to be with a group and us girls always go and do our shopping with our bikes, we all ride up in town, all these middle aged women on

their bikes [laughs], wobbling up into town and [laughs] get our groceries and ride home. It's much more fun than taking a car.

Interviewer: Yes absolutely. And if you wanted to be more active, would it help if there were more destinations within easy walking or riding distance?

Respondent: Well probably except that I think you make your own destination. And you can always say "Well let's go and see such and such" and we do that.

Interviewer: As well as overall it sounds like things are fairly well, not too far in Ulverstone.

Respondent: No it's good here in [town]. Yeah…

Interviewer: Yeah. OK, now going on from what you were just talking about, about neighbourhood design, like the built or the man-made features of your area, now we've talked quite a bit about these and I just want to check if there are any others. If I were to ask you about these built features, what sorts of things would you tell me about? And you've already said about...

Respondent: Really good wide paths that are smooth and not pitted and, yeah, they're really important. Good access. I think that one thing I'd like to see is good access down onto the beach, near where we live because it's been eroded and I just think it would be better if we could access some of that a little bit more easily. It'd be really nice if there was a nice boardwalk right along the beach too. I mean it's not probably so important for me but I think there are people who are probably more disabled than I, who would find it easier to walk on a boardwalk or push someone in a wheelchair in a boardwalk rather than... I mean obviously the beach is very difficult for those people. But the need to be near the

sea is still the same or the desire to, so that would be really good. But for me personally, I can still access everything that I want to access…

Interviewer: OK. So do the built features of your area influence whether you're physically active or not?

Respondent: Yes definitely. Definitely.

Interviewer: And if you wanted to be more active are there any built features in your area that would help and you've already talked about that.

Respondent: Yeah, yeah.

Interviewer: Now the other side of it is the natural environment, if I were to ask you about the aesthetic qualities or the attractiveness of your area, what sorts of things would you tell me about, and you have actually told me about a lot of things. So it's a beautiful place, beautiful scenery, beaches, trees, bush.

Respondent: Yeah. Even just the galahs. [town] well known for a fairly big galah population, but they're lovely if you're walking through [x] Park and you see a big cluster of galahs and you can just stop and... 'cause I always carry a camera with me and take photos of them and I think that's, they're lovely. It's lovely to have some wildlife around.

Interviewer: Do you think your local environment is aesthetically pleasing and attractive near your home?

Respondent: Yes definitely.

Interviewer: And do the aesthetics of your area influence whether you're physically active or not?

Respondent: Yes definitely. I'm a person that's drawn to beautiful things, whether it's just the shape of a tree or a patch of flowers or just seeing what people are growing in their gardens and so I'm very drawn to that, when I'm out walking and riding.

Interviewer: It's sort of magical being out in the environment isn't it? It's just like mmm.

Respondent: It's just beautiful. There's one man who's got a gorgeous garden just up the street from where we live, so often I find myself walking or cycling past his house, just so that I can stop at his fence and just see what he's growing and that sort of thing.

Interviewer: And as you say there's always something to look at. Like you were saying the galahs, I was on the beach last night and it was the oyster catchers, I love those.

Respondent: Oh yes.

Interviewer: Yes the pied oyster catchers. So OK, so if you wanted to be more active are there features of your area that might help if they were more aesthetically pleasing or attractive?

Respondent: No not really. I think, I think it's a very good town for being very accessible to people...

Interviewer: And very aesthetically pleasing.

Respondent: Yeah and...

Interviewer: Like there's parks, there's gardens...

Respondent: Yes, there's lovely parks.

Interviewer: There's trees, there's bush...

Respondent: And there's barbecue areas, there's swings for kids and there's lots of...

Interviewer: And I noticed even on the cycle path and various different other places, there's actually re-plantings as well.

Respondent: Yeah, yep, and there are seats for those people who are walking who maybe need to just sit and gather a bit more strength before they go on. So...

Interviewer: OK. The last area is safety. And if I were to ask you about safety in your area, what sorts of things would you tell me about?

Respondent: Well we live very close to a train line. So that's... when I have my grandson that's always a concern 'cause he loves to go and watch the trains so... but I guess that's just educating him about always stopping and stopping well back from the train line. There's always the fear of stranger danger I guess and that might be a social issue rather than something that we can build to protect kids from. But I know he loves to go down to the skate park on his bike. And I can't always go with him and he's eight years old now, so he probably is quite OK to go on his own, but...

Interviewer: You've always got that antenna out of...

Respondent: It does worry me. But it's wonderful that that skate park's there for him to take his bike down and ride with other kids and learn new tricks and new stunts and it's safe and off the road.

Interviewer: Yep. But overall [town] would be a fairly safe community?

Respondent: I think it's very safe and I think, there's a bit of an intersection but they're putting a roundabout in that now that's not known to be a safe intersection, there's been quite a few...

Interviewer: So that's the other side of it. Road traffic.

Respondent: Yeah and so that impacts you as a walker or as a biker. But, no I'd say overall it's safe.

Interviewer: So does personal safety influence whether you're physically active or not?

Respondent: Yes, yes I'm very careful not to be out alone after dark. There are a number of [laughs] flashers around [town] and my sister has been flashed at a number of times, she's a great beach walker and she goes off walking at nine o'clock at night and men step out of the bushes and... so that's something that really is of great concern...

Interviewer: You can do without that.

Respondent: And so I just wouldn't go and normally everywhere I go my husband comes with me too 'cause he loves getting out just as much as I do, so there's that aspect of safety. But I just think after dark you don't tend to be as visually aware as what you are normally too.

Interviewer: And does road safety influence whether you're physically active or not?

Respondent: It would always be something that I'd be aware of and that I would be monitoring.

Interviewer: Particularly on your bikes, you were saying that your often...

Respondent: Particularly on bikes. Yeah very careful crossing roads and I never just ride over I always stop and listen for traffic as well as look for traffic before I cross.

Interviewer: But where you're riding around the town, is there a problem with road traffic?

Respondent: The main street can be very busy. Even for a small town it can be really busy. You need to be careful to cross at the lights and do the right things there. But in terms of major city traffic like [city] or [city], it doesn't even rate a mention. But I'm always conscious of road safety and just having my wits about me.

Interviewer: Yep, OK. Thank you. And if you wanted to be more active, are there any personal or road safety issues that if they're addressed, might actually help you be more active?

Respondent: Not really, no. I think it's really safe where we are. We live near a school so there's a lot of traffic to and from the school at certain times of the day, but then over the summer, when everyone's on school holidays that traffic's not there. No I think it's good.

Interviewer: OK great, OK well before we finish up, is there anything else that you'd like to mention about where you live, your physical activity or the environment that you live in, that you haven't had a chance to talk about today?

Respondent: No I think I've covered it all.

Interviewer: OK.

Interviewer: Now just to start, I'll start by talking a bit about the place where you live OK. And so can you tell me a bit about what it's like to live around here, your likes, your dislikes?

Respondent: OK I'm an unusual study in that respect because we've sold a house in Victoria, with no residence to live in, in Tasmania, returning to Tasmania after 22 years away, [town] presented itself as an opportunity for employment and there happened to be a house available with it. So my experience of [rural town]itself is limited to walking up and down, as far as the golf course, occasionally walking through the back streets to lengthen the walk to get back home, walking down the street to buy some eggs from the local produce person. So my experience of [town] is yeah, not a long term experience. We've only been here for 14 months now or 15 months now. Yeah, not very long.

Interviewer: OK thank you. … what's the property, is it a house and do you have a block of land with that?

Respondent: It is a house; it's a very small three bedroom house, brick residence … It does have a yard out the back. There's two separate houses that are, they are joined by a carport, a double carport. OK and then behind that there is a yard for both houses, so it's a double backyard.

Interviewer: So is someone in the other house?

Respondent: … Other than that, no there's not but the rest of the …are my backyard as well so I've got an oval out the back, there's a veggie patch to work in, there's a hothouse to work in.

Interviewer: OK so now you're actually right in [rural town] itself? I'm just trying to get a sense of…

Respondent: Yes.

Interviewer: Where you're placed in terms of distance to your nearest town is [rural town]?

Respondent: Yes.

Interviewer: Yeah so you're just down the road there?

Respondent: Well I walked so yeah.

Interviewer: OK yes, yes.

Respondent: …So it's just on the rise of the hill really, it's just, it's not…

Interviewer: So in terms of distance, how…

Respondent: 500 metres.

Interviewer: Long is [rural town] itself?

Respondent: [rural town] itself might be a kilometre and a half.

Interviewer: Kilometre and a half and?

Respondent: Along the highway and then a little bit.

Interviewer: So where's the …placed in all of that? About halfway?

Respondent: It's on the western boundary of it.

Interviewer: OK. Now in research with women living in urban areas, because most of the research has been done in cities, people often refer to their local neighbourhood and I'm interested in understanding whether the term neighbourhood is relevant to women who live in rural areas. So what does the word neighbourhood mean to you?

Respondent: Neighbourhood means to me a cluster of houses probably that within calling out distance, to be able to wave and say yahoo and catch someone's attention.

Interviewer: Does that have relevance here for you?

Respondent: It does here, there are houses that are like a suburban proximity to each other and then there are ones that are a long way away that are just on farms where obviously it's a different sort of community but I don't so much equate that with the term neighbourhood, in my own mind.

Interviewer: OK that's fine. And so now you were saying it's people who are close by, can that be, if you had to put a distance on that, what would that be?

Respondent: From one residence to another do you mean? I don't know, how far can you call out away?

Interviewer: Because it depends on how big the blocks are.

Respondent: Yes it does a bit.

Interviewer: So if the house blocks are slightly bigger.

Respondent: Well look I've come off a two acre block elsewhere and I would have said that we were in a neighbourhood, but I don't know.

Interviewer: So about two acre blocks are you talking about?

Respondent: Yeah I suppose so.

Interviewer: So they've, so if it's not…

Respondent: So I suppose that's 150 metres or something, I don't know.

Interviewer: OK no that's fine because we don't all think about it in that way.

Respondent: No, no.

Interviewer: But it's sort of handy to just sort of get a, it gives a bit of perspective on block sizes.

Respondent: I think if you're talking about a community I'd answer differently, but the term neighbourhood to me, yeah that idea of neighbour.

Interviewer: Well I'm about to ask you about the word community.

Respondent: OK.

Interviewer: And what does it mean to you?

Respondent: Alright a collection of people living in an area that could be quite large, it could be, yes so it could be a 20 kilometre radius even that would be a community of people, who have a common focus point that there is some reason to collect as a community as a sense of being togetherness that might just be a local shop or it might be that once a month we go and meet at Joe Blow's farm to have a discussion about sustainability and agriculture or something, but there's a reason to get together.

Interviewer: And so is that relevant for you here in [rural town]?

Respondent: We came to [rural town]with the intention of throwing ourselves into the community, saying yes to everything, and we have found that there is no community to throw one's self into. When you walk down the street and there's someone out in their garden and you lean over the fence and say "G'day, hello I'm [name]", they say, and there's nothing coming back on the other end. And if you say "Yes, now what was your name?" "Who wants to know?" There's this very defensive, yeah very anxious about people who are new to the community, there's a little bit of reluctance to make friends with people …. So community here…

Interviewer: Does it take a while for that to be overcome?

Respondent: It does and we recognise that but even for the people who are long term inhabitants, when we've been talking to them they say no, there isn't a sense of community.

Interviewer: Right OK.

Respondent: So I just, I think that's really interesting. And as it gets smaller and contracts, the pubs closed, there's two shops that are run by the same people so there's not even any friendly competition there, and there just doesn't seem to be very much. There's no, [community centre] runs things from time to time and there's a bit of Land Care bits and pieces, but if that's not your cup of tea then there's very little else to be involved in.

Interviewer: OK. Now I'm going to talk a bit about physical activity OK, now that's saying that's a very, very broad term. And I'm really interested in what the term physical activity means for you, for you personally?

Respondent: OK anything I do that requires the use of muscles I guess, physical activity.

Interviewer: Yeah, yeah so that could be anything in your life?

Respondent: Yes, yeah.

Interviewer: Do you consider yourself to be physically active?

Respondent: Yes I do.

Interviewer: And could you tell me why?

Respondent: OK it's not on a consistent or regular basis, it tends to be spasmodic, but I do enjoy hiking and camping. And so this year we've walked through the [bushwalking track], we've been camping over Easter and done some walking around north of the [x] area and around the… beaches and into [x] Bay and so we had a swim, and those bits and pieces. I do enjoy going to [x] National Park any time, any time that I've got an excuse, anyone says do you want to go and go up onto the [x], yes please. And next weekend we're going walking with some friends who live in [x] and going up around Mt [x].

Interviewer: That will be beautiful.

Respondent: So physically active in bursts and at other times completely slovenly.

Interviewer: It has a balance in your life.

Respondent: Yeah.

Interviewer: OK. And where does physical activity fit in your life, for instance no interest or a priority or a high priority?

Respondent: Yeah it's not a priority. I like physical activity during my week that is for a reason, like coming to [community centre] today, I didn't even consider taking the car. I thought even if it's raining I'll just get the brolly out and do this, because it's a reason to get out and walk with a destination in purpose. I have difficulty thinking right I'll just go for a walk, but if I've got something to do or I need to go and buy the milk or I need to go and check the mail, then I work, but in terms of making exercise a priority for exercise sake, I don't have that if that makes sense.

Interviewer: Yeah no it makes a lot of sense and actually some of these things around destinations we'll be talking more about so we're interested in your thoughts about whether that helps.

Respondent: Yes.

Interviewer: Yep OK now we're going to talk a bit more about physical activity and the different parts of your life, mainly because we don't want to leave out parts of your life. So in our research when we think about physical activity we think of any activity that lasts for at least 10

minutes, that's the evidence of health benefit and it causes the body to work a bit harder, you might feel a bit warmer, a bit of heart rate or feel a bit of huffing and puffing.

Respondent: Yeah.

Interviewer: So thinking about this type of physical activity over the last two weeks. Now first of all, in the past two weeks have you been active at all at work for at least ten minutes at a time?

Respondent: Yes.

Interviewer: OK could you tell me what you did?

Respondent: Well out on duty I'm walking for 20 minutes and it's constantly walking around the [workplace] doing bits and pieces. Yeah that's a sustained 20 minutes.

Interviewer: And would you call, now in terms of the intensity of that exercise, would it be steady or moderate or vigorous?

Respondent: Just steady.

Interviewer: Yep OK and you've just told me how long you do it for, but how often?

Respondent: Twenty minutes every morning. And then incidental bits and pieces. Our corridor is amazingly long, by the time I walk up and back. And I do that probably ten, 15 times a day so, but it's not that ten minute sustained.

Interviewer: Yes OK. And is this what you usually do?

Respondent: Yes that's usual but there are other unusual things like working in the garden … which would be sustained also.

Interviewer: Yes, as part of your activities.

Respondent: But that hasn't been at all regular.

Interviewer: OK does that depend, and does it depend on the season or the time of the year?

Respondent: Yes it does, yeah and the weather.

Interviewer: So I imagine in the [workplace] you would have to do that sort of regularly anyway?

Respondent: Yes, yeah it happens every single day.

Interviewer: But other activities like working in the garden… and things like that, would that depend on season?

Respondent: It does definitely, yes if we're doing lots of planting or weeding and bits and pieces then that depends.

Interviewer: So more in spring and summer?

Respondent: Yes although I've handed over to [name] who I think is having the next interview.

Interviewer: Yes so I get to meet her next.

Respondent: Yeah she's much more active. She's actually taken over the garden because I don't have time to do it.

Interviewer: Yes it's a bit of a juggle doing everything, yeah.

Respondent: Yeah.

Interviewer: OK and if you wanted to, is there anything that would have helped you be more active at work? More time or someone to be active with or somewhere, different activities?

Respondent: Well time, yes there just isn't time at work to do it. And like I don't need to work to make time to do it because I just think that the work, if the nature of the work is that I'm there to work … and if that includes being active, then that's fine. But it's not their responsibility to make that for me.

Interviewer: Yep.

Respondent: In terms of making use of … lunch times then I've got a partner who is at home all the time at the moment and part of keeping ourselves happy at [town] is that I have some interaction with him during the day so I go home at lunch time ….. often. So I'm not looking for activity either.

Interviewer: Yep, no that's really clear. Now in the past two weeks have you walked or cycled for at least ten minutes at a time to get to or from places?

Respondent: Yes, today. Yes, to buy things from the shop. No cycling. I'm just trying to think with a fortnight. No, I hadn't done this before then, I did [x] Bay.

Interviewer: OK yep. And the intensity of that exercise, steady, moderate?

Respondent: Maybe moderate.

Interviewer: OK and the duration?

Respondent: I suppose its 15 minutes.

Interviewer: Walking to the shops and walking to here?

Respondent: Yeah.

Interviewer: And the frequency?

Respondent: The frequency, twice a week perhaps.

Interviewer: OK now would that be in the day time?

Respondent: Yes.

Interviewer: Yes OK. For after work or weekends?

Respondent: After work and maybe weekends but it's less likely on the weekend.

Interviewer: Yep and where do you do it, it would be in the village?

Respondent: Yes.

Interviewer: Yeah is it called a village here?

Respondent: No.

Interviewer: What's it called?

Respondent: [town]. I don't know what it's called…

Interviewer: I just wondered if there was a term that people used.

Respondent: No, I just go down to the shop or walk to the post office or, yeah it's not a case of going into town or into, no it's interesting.

Interviewer: And is that something you usually do or one off?

Respondent: No it's usual.

Interviewer: And does it change depending on the season or the time of the year?

Respondent: No, no.

Interviewer: OK. And if you'd wanted to, is there anything that would have helped you use more active forms of transport, like walking or cycling, to and from places?

Respondent: Only perhaps if I lived a bit further away I might.

Interviewer: You'd have…

Respondent: I'd have a longer…

Interviewer: You'd have a longer walk.

Respondent: Walk.

Interviewer: Yeah.

Respondent: In terms of other opportunities, maybe if there was a walking group of like minded souls perhaps that I thought I could engage with that was at an appropriate time of the day or something, maybe yeah.

Interviewer: OK. Now in the past two weeks have you been active around the house and yard, and so that's at home inside and outside, for at least 10 minutes at a time?

Respondent: Only if you count housework.

Interviewer: Yes I do.

Respondent: I suppose, yeah.

Interviewer: Yep that's fine, yep as I said we're talking about broad, broadly looking at activity here, all the sorts of things that people do in their lives.

Respondent: Yeah the house is a disaster so it's probably about two hours of constant activity last Friday afternoon. It's a big job.

Interviewer: Yep OK. So what would you call the intensity of that?

Respondent: Just steady.

Interviewer: Steady OK, and you've just told me how long, and how often do you have to do that? Regularly enough.

Respondent: Once a fortnight I suppose.

Interviewer: Yep OK. And when did you do it?

Respondent: Friday afternoon, immediately after work.

Interviewer: OK and why did you do it at that time?

Respondent: Free, my partner was not in the house and that makes it easier if I have to the house to myself I guess.

Interviewer: OK and who did you do it with, alone or with others?

Respondent: Alone.

Interviewer: Yep OK. And is that what you usually do?

Respondent: Yes.

Interviewer: Or is it one off? And does this change depending on the season or the time of year?

Respondent: No. It all needs doing all the time.

Interviewer: Unfortunately. OK and if you'd wanted to, is there anything that would have helped you be more active around the home, and that's inside and outside?

Respondent: Yeah if the house was my own house, yes. There's no incentive to do anything extra or to establish a veggie patch of ….. Yeah it's sort of, yeah. It would be very different if it was my own, I'd want to be doing things and improving bits and pieces.

Interviewer: Now have you been physically active for at least ten minutes during your leisure or spare time?

Respondent: Well yes because that's, spare time. Yes.

Interviewer: And so…

Respondent: Actually I did go to the gym one day last week so that was in my spare time, must be a time after work.

Interviewer: OK and sometimes you go for walks in your spare time?

Respondent: Yeah.

Interviewer: May not have been exactly in the last couple of weeks but it's sort of trying to get a bit of a picture of what you would usually do, so yep OK. Now, the intensity of that activity?

Respondent: Look it really gets beyond moderate for me. I just don't like getting hot and sweaty. I like to enjoy it and I don't mind huffing and puffing but I just don't like getting to the stage where I just feel like I need to go and have a shower and everything else. So unless you're putting me in a swimming pool where actually I will go quite hard then, but there's no swimming pool here so that doesn't happen. But that's the only place that I will go to the really extreme level of probably getting that heart rate up and the breathing going quite ferociously because I'm wet anyway I suppose.

Interviewer: Yeah but I notice, I've been noticing too, it doesn't take much in terms of getting warmed up.

Respondent: Yes.

Interviewer: It doesn't, just going for a walk, it usually sort of starts to really warm up.

Respondent: Yeah.

Interviewer: So it's just about the constant and the length of time. OK so thinking about that exercise you were talking about, which was the bushwalking and the gym, the duration, how long would that have been for, those activities?

Respondent: The bushwalking was ten hours of walking, it was five hours and five hours, we had an hour in the middle for, no we had… It was 10 hours total so it was, yeah an hour for lunch in the middle of it and a good rest in the middle. It was slow walking. And that was, it was 21 kilometres on the first day and 14 on the second, so it was, yeah. And I wasn't particularly fit at the time so it was a stretch that was.

Interviewer: That's a long walk.

Respondent: It was a long walk but 21 on the first day, it was pretty amazing, yeah.

Interviewer: OK and how long with the gym?

Respondent: The gym, dear look I can manage to stretch it out to about 40 minutes and I just get bored. I just don't like being in a room. It's better here. I've been twice to a commercial gym years ago and I just can't stand the smell and the closeness and there's no fresh air and I'm surrounded by walls and I just don't like it. So here I can manage to go for about half an hour and then I just get sick of it.

Interviewer: Because that's at [workplace] isn't it?

Respondent: It's at [workplace] so there's running machines and rowing machines and weights, and yeah.

Interviewer: And the frequency, how often for those activities?

Respondent: About once every two months for the gym.

Interviewer: Yes, yes.

Respondent: And probably the bushwalking, it's probably five times a year. It's not with any great frequency but yeah, it's something that I enjoy but it takes a bit of planning and organising if it's overnight and carrying everything.

Interviewer: Yep OK. Let's see, now when would you have done these activities?

Respondent: Long weekends, Easter holiday, school holidays, the gym is sort of after work. Never on a weekend because on a weekend it means I have to go down to the [workplace] first, turn all the security system off, lock it all back up, go right round the other side of the [workplace], use the gym, walk back around again and put the alarm system back on and then go back home. I just, in my brain it just seems, it's not particularly difficult but in my brain it's difficult.

Interviewer: Yes especially when you're there five days a week and you do all of that.

Respondent: So if I do it on a [week] night, then the [workplace] is still off system until 10:00 so that's it.

Interviewer: And those activities, who would you do them with?

Respondent: The hiking is with my partner. Gym is usually with my partner, I think I've gone once by myself.

Interviewer: Yep and does this change depending on the season and time of year?

Respondent: No.

Interviewer: Yep OK. And if you'd wanted to, is there anything that would have helped you be more active in your leisure or spare time? You were mentioning about access to a pool for instance.

Respondent: Yes I think if there was a swimming pool then I might use that.

Interviewer: Is there one that's anywhere in the [rural region]?

Respondent: There's one at [nearby town] but it's closed through winter and during the rest of the year, it's on school grounds too so I guess you'd be fighting your way through school kids after school to get into it. But there is one, a tiny one at [nearby town] too but I'd prefer a larger one that I could do some laps in I think probably. Yeah but I'm not here long term so that's alright.

Interviewer: OK. Now I want to talk now about physical environments, so those, we've talked a bit about where you live and the physical activity.

Respondent: Yeah.

Interviewer: Now the physical environments where you live, work and play. And so when I talk about physical environment I mean the physical things that surround you, the roads, the trees, houses, shops, traffic lights, roads, parks, those sorts of things.

Respondent: Yeah.

Interviewer: And some of these things are people living in urban areas have said may be important for their physical activity, so we're interested in seeing whether they're relevant for you too. Can you tell me a bit about how easy or difficult it is to be physically active in your neighbourhood or your area?

Respondent: It's, yeah it's quite easy.

Interviewer: Yep.

Respondent: The footpaths are not well formed and uneven surfaces and gravelling bits and broken, but other than that around [town] itself it's OK to be physically active. One of the things, well the physical environment that I would like to see here is some access to the water, that you could actually…

Interviewer: Yes, yes.

Respondent: It just seems like there's this… I know there's this river here and I can sort of see it from the bridge and I can sort of walk a little bit, but I can't actually get to the river.

Interviewer: Yeah you're not the only one; I've heard that one too. I was going go ask you that too is that whole sort of stuff, especially when you've got these sorts of smaller communities, can you actually sort of connect with parts of it rather than just the walk along the main road, and you've just said the river?

Respondent: No it's very difficult.

Interviewer: OK yeah that's very tempting if you can't, you can see it but you can't get to it.

Respondent: Yes, yes and you can sort of get through the park over there into the muddy bits, but it's not…

Interviewer: There's a park over there?

Respondent: There is a park.

Interviewer: Yep, there is a park.

Respondent: The [x] Park or something.

Interviewer: OK.

Respondent: I don't know what it's called. And it's just very difficult to get down to the water, you just can't.

Interviewer: There's just no track?

Respondent: No, no like there's a grassed area…

Interviewer: Is it private property?

Respondent: No there's a grassed area and there's a barbeque there, and it's lovely and shady trees but then, and the river is next to it, but it's all mud and there's no formed, there's no little spot even that's logical for you to just sit and just contemplate.

Interviewer: Yep let alone walk along a while.

Respondent: Yeah, no.

Interviewer: OK.

Respondent: And the rest of it does seem to be private, that you can't get to.

Interviewer: Yeah you can't get to.

Respondent: But having said that and the physical environment, you can go through up the hill a bit and go along x] Road and that's a nearby physical environment that's quite pleasant and you can get to the water there and you can dabble your toes in it and that's lovely.

Interviewer: So how far would that be? Would you need to get in a car to do that?

Respondent: You do need to get into a car.

Interviewer: Yep OK.

Respondent: But one Friday afternoon that's something that my husband and I did, we went down.

Interviewer: Yep is that what, a two minute drive or 10 minute drive?

Respondent: Yeah a five minute drive even, yeah it's not far.

Interviewer: Yep OK and you can actually access and walk around there can you?

Respondent: Yes, yeah. And walk up the river on the rocks and do things at the [x] River.

Interviewer: Because it's very hard with the beautiful environment and you want to get to it and enjoy it.

Respondent: Yes.

Interviewer: OK now I'm going to look at some features of physical environment, and availability and accessibility is the first one of places to be active, but if I want to ask you about places to be active in your neighbourhood, your area, what sorts of things would you tell me about?

Respondent: Paths, so footpath areas, the little park, the oval at the school. There's also hard courts there that you could put a net up and play tennis on, there's a netball court. Then the [x] River, you could canoe and fish and do things.

Interviewer: You can get into it, is there access to it?

Respondent: Yes, yes.

Interviewer: There are access points?

Respondent: Yes.

Interviewer: But not through the park.

Respondent: But not through here, no you'd need to… This is for the [x] Dam and [x] Lake which runs out of it as well, that goes down, so.

Interviewer: So where would you access, like if you wanted to put your canoe in the river?

Respondent: Well I don't know where you'd do it at [town] it would just be really difficult. You'd have to go, yeah around the back. I wouldn't even know how to start.

Interviewer: Yeah, yeah.

Respondent: We took the dog for a walk trying to find a spot to get down to the river and it was just too difficult.

Interviewer: OK and so, OK there are a few facilities. And access, are these places convenient? Are they easy to access?

Respondent: Yes, yeah, apart from the actual local river.

Interviewer: Yeah that's, to get to it OK. And for instance like the gym and the cost or opening hours with that?

Respondent: Yeah well I believe it's a community gym that's free, I don't think there's any cost at all.

Interviewer: That's very good.

Respondent: And it is only accessible while the school is open for the general community, so Monday to Friday, and then in school holidays it isn't so suddenly it would be withdrawn. But for me, I've got [ongoing access] so it's accessible, there's no excuse really if it was something that I liked doing.

Interviewer: If it's something you want to do.

Respondent: Perhaps they could, yeah build in a reward at the end.

Interviewer: Fair enough. OK so does having places to be active influence whether you're active or not do you think?

Respondent: Hugely I think, yes I think if there's no incentive to do anything, then it would be really difficult.

Interviewer: Or if it's difficult to get to.

Respondent: Yeah, yeah.

Interviewer: It just gets frustrating doesn't it?

Respondent: Yeah it does, yes.

Interviewer: If you wanted to be more active, are there things or places that would help if you, if they were available or more accessible?

Respondent: Yeah the swimming pool would definitely be a positive for me.

Interviewer: So how far away would the pool that you mentioned be, at the school, even though given that it wouldn't be exactly desirable the one that you mentioned, but how far away would it be?

Respondent: It would be 35 minutes drive.

Interviewer: Yep OK.

Respondent: The other thing would be community classes that were active, so maybe some of the Zumba dance ones or I don't know, Latin dancing is something that I still have yet to do that I will do once we're in a close enough to some classes that will do that. So, organised activity that is fee for service even is fine, but yeah.

Interviewer: That's certainly something that's been talked about by other women in rural areas, if there was an activity or something that you could do, and also have a benefit of feeling like being part of the community as well.

Respondent: Yes. A few years ago I did a couple of terms of belly dancing and the woman who was running that was very good at nurturing women and feeling, building that confidence and sense of a group but also nurturing the inner goddess if you like, I don't like much of the new age terminology but just to honour yourself as a woman, to love your hips and your belly.

Interviewer: Which is really important, the whole difference in culture when it comes to belly dancing is a very different, comes from a very different culture.

Respondent: Yeah.

Interviewer: So it's good to have someone who can…

Respondent: Yes rather than just focus on the physical activity, there's so much more to be gained from that joint experience.

Interviewer: Yes, yes.

Respondent: If you can get people there to start with, which is always the problem.

Interviewer: Now destinations, we started talking about that at one stage. If I were to ask you about places that you could walk or cycle, i.e. it's a shorter distance to and from your home, what sorts of things would you tell me about?

Respondent: So existing things?

Interviewer: Yes, yes, … you mentioned shops.

Respondent: Yep, a library, bookshops maybe. What else would I walk to? Meeting places or a group that I needed to walk to. Yeah I'm not sure.

Interviewer: OK that's fine. Does having destinations influence whether you're active or not?

Respondent: Yes unfortunately for me it does, some people have that intrinsic motivation to just go and do it for the sake of exercise but for me, I really like to have a purpose at the end of it.

Interviewer: That's fair enough.

Respondent: If someone invites me around for tea and it's within walking distance, then I'll walk, so all of those sorts of things, a reason to do it.

Interviewer: Yes, yes and it's about looking at a number of things you can do rather than just one thing, going to tea, it's like well what else can I…

Respondent: But then thinking about like next week we're going hiking with these friends, it's about, it's not because I want to get to the destination that they're taking me to, so it's about then the social interaction of having someone to do it with that is just… Like I don't have anyone to do things with because I'm just not reliable, I'm either here or I'm not here and it's just confusing for everyone. So it's part of the, where we're at.

Interviewer: And the nature of your work as well.

Respondent: Yeah.

Interviewer: Yeah. Now if you wanted to be more active, would it help if there were more destinations within an easy walking distance?

Respondent: Yes probably but it's pretty close here.

Interviewer: I want to talk a bit about neighbourhood design, which is the non natural features of your area, and that's manmade or built.

Respondent: Sure.

Interviewer: Now if I were to ask you about those features, the non natural features, what things would you tell me about?

Respondent: The online access centre in the little house, the hospital, the doctor's surgery, [community centre] here, the park that's actually designed and for the purpose for people to go to.

Interviewer: That's not that one?

Respondent: Yeah that one there.

Interviewer: That one, OK. And what's the house that's got online access?

Respondent: The online access, it's just along the main street, there's just a little house that's been made into the access centre that also houses the library.

Interviewer: Footpaths, lighting?

Respondent: Yes there is footpaths but not in good repair.

Interviewer: Are they continuous?

Respondent: No, no.

Interviewer: So through this area they're not continuous?

Respondent: Some parts they are but when you, as soon as you leave the main street, some of them have footpaths and others don't, it's just a bit of up and down.

Interviewer: So you'll be walking on the edge of the road?

Respondent: Yes, yeah.

Interviewer: And do the streets connect?

Respondent: Yes but they sort of stop at properties at the end.

Interviewer: So they don't connect together as a community.

Respondent: No.

Interviewer: Like if you wanted to go for a walk around?

Respondent: Yes, there is sort of one street that's a block around and that's continuous so I like that, I can do that, but in terms of going further, no.

Interviewer: And lighting is there enough lighting if you want to go for a walk in an evening or to different destinations?

Respondent: Probably for what is around there's enough lighting yes, but you'd want to carry a torch anyway for the bumpy bits.

Interviewer: Now do these built features of your area influence whether you're physically active or not?

Respondent: In the sense that they create a destination, yes they do. And the design of [town], yes if it was laid out differently there would be greater range of places to walk I suppose but it just not.

Interviewer: It sounds like it's a bit linear.

Respondent: It is a bit linear, yeah.

Interviewer: OK and if you wanted to be more active, are there built features in your area that would help? Like that connection down to the river.

Respondent: Yes that's true, yes that too. Yeah that's hard to predict, yeah. If I was a golfer I'd be very well set up.

Interviewer: Yeah I noticed the golf course as soon as I came in.

Respondent: Wonderful golf course, my partner uses it quite a lot.

Interviewer: Fair enough. So is there any other features for you?

Respondent: No.

Interviewer: Now the other side of it is the natural features of your environment or the aesthetics. So if I were to ask you about the aesthetic qualities or the attractiveness of your area, what sorts of things would you tell me about?

Respondent: The hills, the changing colours on the sunrise on the hills out my back window are just gorgeous, it changes and it's, and when it has snow on it and its lovely. I love trees, I love water, and that's why it's sad not to be able to get to the water. It's just a very pretty place. It's not flat and uninteresting and it's not like the wheat fields out in North West Victoria or somewhere, it is like lots of hills and changing scenery depending on the season and the time of day. I appreciate all that.

Interviewer: It's a pretty special spot really isn't it, the [region], they're gorgeous.

Respondent: Yeah and it's really quiet and isolated too and it's got its own sense of appeal if you can relax into that and not feel frustrated by the quietness, yeah it's nice.

Interviewer: OK and do you think your local environment is aesthetically pleasing and attractive near your home?

Respondent: Not, the natural environment is yes I think but the physical environment, the manmade environment is just lacking something altogether.

Interviewer: Yep and that's the home you live in.

Respondent: Yes.

Interviewer: The house.

Respondent: Yeah. But the natural beauty around is evident, yeah it's nice this place.

Interviewer: And do you think the aesthetics of your area and neighbourhood influence whether you're physically active or not?

Respondent: No probably not.

Interviewer: It's a hard one to sort of talk about isn't it, because you enjoy it and you obviously go out walking and looking for walks.

Respondent: Yes, yes. But whether I would be any more active if it was any different to what it was is in my mind, I just honestly not sure that I would be.

Interviewer: No but do you think it affects you being active, live having a pleasanter surroundings?

Respondent: Affects my ability to be active do you mean?

Interviewer: Yeah or your interest in being active.

Respondent: OK yes possibly. If it was a really, really pleasant place to walk around in, to be involved in, then I might do it more often or the frequency of it.

Interviewer: Or as it is now, is it one of the things that you appreciate about living here is the beauty?

Respondent: Yes.

Interviewer: Because you're obviously saying that you're walking and find beautiful spots to go walking.

Respondent: Yes.

Interviewer: So it's sort of like, in a way, one of the givens.

Respondent: Yes, yes.

Interviewer: Like of course we want to, but I guess that's what we're trying to get at too is, is the fact that you live in a beautiful area something that you enjoy because, and you enjoy by being active, going for a walk.

Respondent: Yes and I'm including [x] and [x] as my area because if I was living in [city] I wouldn't make the effort to go there, but the fact that it's just down the road is, yes we can do that. And so it is something that I think yes we could go for a walk there that would be really pleasant, whereas if I was in [city] then it wouldn't even occur to me. So in that sense it does affect the way that the natural beauty, yeah

Interviewer: And sometimes I think some of these questions have been asked in urban areas, whereas people who live outside in beautiful surrounds…

That's some of the reasons why people choose to live out in rural areas so it's sort of like a given.

Respondent: Yeah.

Interviewer: Yeah OK. Alright now the last area is just about safety.

Respondent: OK.

Interviewer: And if I was to ask you about safety in your area, what sorts of things would you tell me about?

Respondent: Well physical safety, I mean for someone who's got balance issues or something, there's no incentive to be out and about because the footpaths are not in good repair at all. There's a gentleman who rides around in an electric wheelchair and I fear for him all the time because it's just…

Interviewer: How on earth he does I think he must ride on the road sometimes.

Respondent: I think he must come up on the roads and do things sometimes because it's just so appalling.

Interviewer: And is it safe to be on the roads?

Respondent: No because there are log trucks going up and down so that is a real concern. If people had strollers, and I don't think I've ever seen a stroller around [town] actually or someone pushing a child around because, you know up in the school grounds they do but. And I think that would be one of the reasons, for safety, physical safety there. In terms of personal safety, feeling safe on the street to walk around, absolutely safe.

Interviewer: And that's all the time and dark?

Respondent: At dark as well. I live on the… but I'm frequently there by myself for weeks at a time and there is no-one within half a kilometre almost that I think if I had a problem I could step outside and yell my little lungs out there would be nobody who would even hear me. So fortunately, yes I feel quite safe personally.

Interviewer: And does personal safety influence whether you're physically active or not?

Respondent: Hugely. If I didn't feel safe then I wouldn't. If I needed to come down the street of a night time, I wouldn't feel anxious about that, that would be fine. Yeah but I wouldn't otherwise.

Interviewer: And does road safety influence whether you're physically active or not?

Respondent: Yes, if I was a cyclist I don't think I'd be cycling around here. But being on the road, the log trucks are frequent and difficult. And there's a lot of tourists too who are driving cars that they're unfamiliar with and that's a little bit anxiety producing sometimes.

Interviewer: Yes and with areas that haven't got footpaths and you're left to the shoulder of the road and there's not really anywhere to go.

Respondent: Yes. But up and down the main street here, that's pretty well taken care of.

Interviewer: That's OK?

Respondent: Yes.

Interviewer: So for that… What did you say about a kilometre or so?

Respondent: I reckon it's only a kilometre and a half from the front, the first speed sign to the last one I reckon.

Interviewer: Well that's like I was going to ask you, will it slow people down.

Respondent: Yeah. Might be two kilometres on the outside.

Interviewer: Yeah OK. And if you wanted to be more active, are there any personal or road safety issues that if they were addressed might help you be more active?

Respondent: For myself, no I don't think so. But for other people in the community, yes a good footpath would be essential.

Interviewer: Thank you for that. Just one last question, before we finish up is there anything else that you'd like to tell me about where you live, your environment, your physical activity, that you haven't covered today?

Respondent: Only that I live a bit of a split life. I have the life in [town] and I have a life in [city] too, and so that… And I suspect that there would be quite a few people in the community who have a similar split life.

Interviewer: Yes so you have responsibilities…

Respondent: Elsewhere.

Interviewer: The family responsibilities. Do you go down on a regular basis to [city]?

Respondent: Yes. Yes once or twice a week even.

Interviewer: Yes.

Respondent: Sometimes more than that, sometimes every day for three or four days in a row. So it just depends, and so the physical nature of what I do in [city] might be very different to what I do here or vice versa, like it can, yeah be a bit topsy-turvy.

Interviewer: So it's hard to sort of settle into your life in that way.

Respondent: Yeah, but then large blocks of my time would be here too, so that's all.

Interviewer: Thank you.

Respondent: That's alright.

Interviewer: OK. So, I thought we'd start a bit about, talking a bit about the place you live.

Respondent: Yep.

Interviewer: Yep. And can you tell me about what it's like to live where you do, and what's your likes and dislikes are?

Respondent: I've lived here for nearly 20 years.

Interviewer: Uh-huh.

Respondent: Things have, a lot have changed. We've lost almost our hospital. So, that was one disappointment. The high school we lost.

So... it's quiet up here. That's what I like. And... and there's nothing really... I can't say, really say anything else.

Interviewer: OK. And tell us a bit about where you live? You're saying that you live a bit, a bit out of town?

Respondent: Yeah.

Interviewer: About 30 minutes walk.

Respondent: Yeah.

Interviewer: That's how many K's?

Respondent: I think probably not, just under three k's.

Interviewer: Under three k's.

Respondent: Under three k's.

Interviewer: Yep. Yep. And you live, is it on a house block, or is it with land?

Respondent: We live on our property.

Interviewer: Uh-huh. And how big's the property?

Respondent: I'm not quite sure, but that's only the small part of it where we are. The rest is just up [X] Road, so it's a bit area.

Interviewer: Uh-huh. So it would be thousands of, 1000 acres?

Respondent: Probably a bit more than that, I reckon. Yeah.

Interviewer: OK. That's fine. And, the, the length of time you've been living there?

Respondent: In that house, it's been nearly six years, and about two months.

Interviewer: Uh-huh. And then you've lived in this area for...?

Respondent: For nearly 20 years...

Interviewer: Yes.

Respondent: ... in, in different houses.

Interviewer: In different houses.

Respondent: Yes.

Interviewer: OK. And so the reasons for living here?

Respondent: My husband... well, what, he wasn't my husband when we first moved in.

Interviewer: Uh-huh.

Respondent: Moved here, he got a job here, and his parents moved here as well, from [town]. So there's, we've just stayed.

Interviewer: OK. Yep. OK. And, so, in the research that's been done, I was saying that like, most of it's all been done in urban areas.

Respondent: Yep.

Interviewer: Yep. And people often refer to their local neighbourhood. Yep? And I was interested in understanding what the term 'neighbourhood' means to women who live in rural areas, whether it's relevant or not.

So, what does the word ‘neighbourhood’ mean to you? Do you think it’s relevant?

Respondent: Yeah. In some way, it can be, yeah.

Interviewer: Uh-huh.

Respondent: I don’t have many neighbours. I just have my mother-in-law just down the road, and my son up the road. So, I don’t get out to see a lot of people.

Interviewer: Uh-huh. Uh-huh. So they’re fairly close proximity, are they?

Respondent: Uh-huh.

Interviewer: A few kilometres?

Respondent: About ten minute walks.

Interviewer: Oh wow. OK. Very close.

Respondent: Yes.

Interviewer: And so, what would, is that your neighbourhood, or?

Respondent: Yeah.

Interviewer: Yep. Yep.

Respondent: I used to be really active.

Interviewer: Yep.

Respondent: When my kids were up at the school, and since then, since they've left, I haven't. I've just been at home.

Interviewer: So your neighbourhood's sort of a bit smaller than what it used to be?

Respondent: Yep.

Interviewer: With the kids, and, and at school. Now it's sort of closer with family being close by?

Respondent: Yep.

Interviewer: Yeah. OK. So, neighbourhood means, to you, what? People that are quite close in proximity?

Respondent: Yeah, and I would like to have a little bit more. But... yeah.

Interviewer: Uh-huh.

Respondent: It's hard because everyone's got their own...

Interviewer: What they're doing, and...?

Respondent: Yeah.

Interviewer: Yeah. If I was to talk about the word 'community', what would that mean to you? Is that a relevant term? That would be relevant?

Respondent: Yeah.

Interviewer: It would be?

Respondent: Yeah.

Interviewer: Yep. So what would it mean to you, ‘community’?

Respondent: In what way?

Interviewer: In living in here. What would the word ‘community’ mean? Is, is there a community that you would say “Oh yep. OK. Live in [town], and yep, this is my community.”

Respondent: Yeah. I can't explain.

Interviewer: Uh-huh. It has a sense to you. Is it different to ‘neighbourhood’?

Respondent: (Pause). No, I think it would be.

Interviewer: It would be different?

Respondent: Yeah.

Interviewer: So different in what way?

Respondent: I don’t know how to answer.

Interviewer: Yeah. Sometimes communities, sometimes the difference is about... a distance, or, you know, a literally a geography, whereas community’s sometimes about like-interested, minded people. And it can be a different, different area in that way. And sometimes they’re the same.

Respondent: Similar.

Interviewer: Uh-huh. But, so they're more similar than they are different?

Respondent: Yeah. I think so. Yeah.

Interviewer: Yep. Yep. Yep. Yeah. OK. Yep. And so, I mean, you, you've got, indicated a bit of a, a distance with the neighbourhood, that's a couple of minutes walk. Is there a similar sort of distance with community?

Respondent: Uh-huh.

Interviewer: Yep. OK. And that sounds like it changes depend, depending on what sort of...

Respondent: Yeah.

Interviewer: ... like you were saying. With school it was a bigger community, and now the kids have grown up. Yep. Yep.

OK. So I wanted to talk about physical activity, OK. And that's a really broad term.

Respondent: It is.

Interviewer: It can mean all sorts of different things. And it's different for everyone. So that's why we want to find out what it means for you.

Respondent: Uh-huh.

Interviewer: OK. So what, what does the word 'physical activity' mean for you?

Respondent: Health.

Interviewer: Uh-huh.

Respondent: Mostly. And being active.

Interviewer: Yeah. So being active in your life?

Respondent: Yeah.

Interviewer: Yeah. Yep. Yeah. OK. Do you consider yourself physically active?

Respondent: No.

Interviewer: OK. Why not?

Respondent: I don’t have the motivation.

Interviewer: Uh-huh.

Respondent: I need a kick-start. I was active. Because I used to do a lot of things with the school. And I used to work. But now, just, everything’s just... slowing down.

Interviewer: So when you were saying you did activities with the school, what sort of things... did you do?

Respondent: Fundraising.

Interviewer: Oh, right. OK.

Respondent: And they, I used to go to school up there, like a college thing. Do courses. And then, I went to work. So.

Interviewer: So that was when you had the high school here?

Respondent: Uh-huh.

Interviewer: Yeah. That's a big difference isn't it?

Respondent: It is.

Interviewer: So, where does physical activity fit in your life? Is it, not of particular interest, or, is it...?

Respondent: I'm interested of getting active. It's just... I think the motivation's just not there. But yeah. I do me housework, but that's, I know that's getting, getting active with it. But anything else I just can't get into it.

Interviewer: OK. Well, doing housework's certainly active. (Laughing).

Respondent: Yes.

Interviewer: Never ending.

Respondent: No, well, that's it.

Interviewer: Yeah. So, in our research, when we think about physical activity, we think of any activity that lasts for at least ten minutes, OK? And that's causes the body to work harder. Your heart rate to go up or you might be breathing a little heavier. You know. Huffing and puffing.

So, thinking about the type of physical activity during the past two weeks, and I'll go through different parts of your life, mainly so we can just make sure we cover off things.

Now, you were saying that in, not currently working?

Respondent: No, I got let, laid off.

Interviewer: Oh. Things closed down here, did they?

Respondent: No. It was when the recession was in.

Interviewer: Oh, OK.

Respondent: And he didn’t have the work. And I was the last person to go on, so I had to be the first one to go off. So.

Interviewer: That’s...

Respondent: And I haven’t worked since.

Interviewer: So that’s hard. Yeah. It’s hard to find the work that you want to. Yeah.

So, have, in the past two weeks, have you walked or cycled for at least ten minutes at a time to get to and from places?

Respondent: I have walked.

Interviewer: Can you tell me a bit about what you did?

Respondent: I usually just walk down, probably about half an hour walk down the road from where I am. And then I walk back again. But that, that's not every day.

Sometimes I walk, my husband and I walk over to our, to his mother's. But that's only about a five minute walk. But yeah. Not every day.

Interviewer: Uh-huh. Uh-huh. And so, the half hour walks you were talking about, what would be the intensity of those? Would they be pretty steady, or moderate?

Respondent: No, just steady going along.

Interviewer: Yep.

Respondent: Because if I walk too fast, I get puffed out too quickly.

Interviewer: And it's sort of about thirty minutes in and thirty minutes back?

Respondent: Yeah.

Interviewer: Yep. Yep.

Respondent: I try and make it thirty minutes anyway.

Interviewer: Yeah. And how often might, would you do that in the last couple of weeks?

Respondent: I've probably done it about once.

Interviewer: Uh-huh. Uh-huh. Uh-huh. And when did you do it? Is there a time of the day? During the day that you did it?

Respondent: It was mainly before tea.

Interviewer: Yep. OK. And that's during the week, is it?

Respondent: Yes.

Interviewer: Yep. And that was sort of walking in from your place, along the, the road here, into...

Respondent: Yeah, going towards, [x] way.

Interviewer: Oh. OK.

Respondent: I head down that way.

Interviewer: Oh, OK. You walk the other way. OK. Alright. And, why did you do it there?

Respondent: Health problems. For my health.

Interviewer: Oh, OK.

Respondent: Yes.

Interviewer: And why did you walk the, where you did walk?

Respondent: Because I used to walk it... about a couple of months ago, I used to walk it every day. And, about a week, I walked the same distance, and then another week, I walk a bit further.

Interviewer: A bit further.

Respondent: Yes. And then, yeah. And then all of a sudden, I stopped.

Interviewer: OK. Right. So, that, that's a usual walk that you would, you would do?

Respondent: Yes.

Interviewer: Is you tend to go out, not this, this way, into [town]? You go...?

Respondent: Oh, sometimes I come, one, probably once a year if I need anything...

Interviewer: Yep. OK.

Respondent: ... from the shop. But yeah.

Interviewer: Yep. Oh, OK. And what attracted you to mainly walk the [town] side then?

Respondent: I don't really know. Just, it's... because this way, you got, I suppose it's more quieter going down that way. And you've hardly got any traffic.

Interviewer: Yeah. Because, are you having to walk alongside the road?

Respondent: Yes.

Interviewer: Yeah. Yeah. OK.

Respondent: Oh, sometimes I can walk on the property, if I wanted to. But I think it was easier just to walk down the road then back again.

Interviewer: Uh-huh. Yep. Yep. And were you alone or with others when you walked?

Respondent: Before I walked I was, I had a walking partner. And then she left. And now, just walk on my own.

Interviewer: So when, in the last couple of weeks that would have been by yourself?

Respondent: Yes.

Interviewer: OK. And is there a reason why you walk by yourself?

Respondent: Because my husband won't walk with me. Because he doesn't need to lose the weight.

Interviewer: (Laughing). Right. And is this what you usually do, or is, was it a one-off?

Respondent: It's...

Interviewer: Like, you said you walked once in the, the last couple of weeks. Is that usual?

Respondent: Just the usual walk.

Interviewer: Is that usual, that you walk once in a fortnight?

Respondent: No, it's just got a, had a fad that I wanted to walk. And I have to walk for health reasons. So, I haven't had a walk... for a while. Til today.

Interviewer: Oh, well done. (Laughing). OK. Does this change depending on the season or the time of the year?

Respondent: It’s a bit cold of, of a night. But, walking shouldn’t stop you, no matter what kind of weather it is.

Interviewer: So it doesn’t tend to. And if you wanted to, is there anything that would have helped you to, to walk to and from places? Like...

Respondent: It would be nice if I had someone else to walk with. Because I wouldn’t be so lonely.

Interviewer: Yeah. Yep.

Respondent: And I would have the motivation.

Interviewer: Yes. It always helps when there’s somebody else, isn’t it?

Respondent: And yeah, it does.

Interviewer: Fair enough. In the past two weeks, have you been active around the home? Either inside or outside? You were talking about doing housework, I know.

Respondent: Yeah, housework’s mainly. And sometimes gardening.

Interviewer: Oh yep. OK.

Respondent: Getting my garden going.

Interviewer: And was, and was that for at least ten minutes at a time?

Respondent: I usually do it a full day.

Interviewer: Wow. I'm impressed.

Respondent: When I get started, I can't stop.

Interviewer: Yeah, you do. OK. OK. OK, so can you, so you’re doing housework and you’re doing gardening?

Respondent: Yep.

Interviewer: So let’s have a look at those two activities. What would be the intensity of them? Would it be steady or moderate or vigorous?

Respondent: Well, housework would probably be steady.

Interviewer: Uh-huh.

Respondent: But gardening is more... more intense.

Interviewer: Yeah. Would it be moderate or vigorous?

Respondent: Oh, in between.

Interviewer: Yep. OK. And how long would you have done those activities? The housework and the gardening? You were saying all day, for the housework?

Respondent: Yeah.

Interviewer: Yep. And the gardening?

Respondent: Gardening, it depends on the weather.

Interviewer: (Laughing). Fair enough.

Respondent: Yeah.

Interviewer: So...

Respondent: Well, probably...

Interviewer: In the last two weeks, what would it have been?

Respondent: About three days for gardening. It depends.

Interviewer: Yeah. Yep. Yep. And how often in the last two weeks would you have done those two activities?

Respondent: I've only done a little bit of gardening in the last two weeks. But housework's just full on. It's every day.

Interviewer: OK. So every day you'd do... most of the day housework?

Respondent: Yep. Vacuuming, dusting. But just the usual things that you do.

Interviewer: Uh-huh. And in the garden? You... in the last two weeks? Bearing in mind it was cold for the first week of it.

Respondent: Yes. But this weekend wasn't too bad.

Interviewer: No.

Respondent: Probably a little bit of weeding, but.

Interviewer: Uh-huh. So how long do you think you might have spent?

Respondent: About an hour or so.

Interviewer: Uh-huh. OK. And, your, let's see... now, would you have done this by yourself, or with others?

Respondent: I usually do gardening with my husband. He usually does the pruning, because I don't touch that. I just do the weeding and, and cleaning up and what not.

Interviewer: Uh-huh. And this is what you usually do, or is that a one-off?

Respondent: Yes.

Interviewer: Yep. So...

Respondent: We always do it.

Interviewer: ... does it change during, with the seasons or the time of the year?

Respondent: No, not really.

Interviewer: Yep. Yep. OK. And if you'd wanted to, is there anything that would have helped you be more active around the house, inside or outside?

Respondent: What do you mean by that?

Interviewer: Well, things like more time, or someone to be active with, or some...

Respondent: Oh, I don't like anyone else doing my housework, because they don't do it right.

Interviewer: Yes. (Laughing). Fair enough.

Respondent: And I think the same goes for the gardening.

Interviewer: Yep. So there wouldn’t have been anything?

Respondent: No.

Interviewer: OK. And have you been physically active for at least ten minutes during your spare time, in the last two weeks?

Respondent: Does scrapbooking count?

Interviewer: You tell me. (Laughing). Is it more than ten minutes?

Respondent: No. Hanging out the washing.

Interviewer: Uh-huh.

Respondent: I don’t know, that’s, that wouldn’t take ten minutes though.

Interviewer: And you’ve talked about your walking, so.

Respondent: Yeah. Well, when I am at home, I'm always, I'm always on the go, kind of thing, but not as much as I want to. Wood cutting. Doing the wood. Yeah. Filling the wheelbarrow up. Things, little things like that. Probably about ten minutes, doing things like that.

Interviewer: Yep. Yep. And they’re fairly moderate exercise, aren’t they?

Respondent: They are when you’re swinging an axe.

Interviewer: Yes. Yes. And is that, how often would you have to do that?

Respondent: I usually do it through the week, because my husband works, so. And by the time he gets home it's dark and he's buggered. So, I usually do it through the week. And the weekend is just, he does it.

Interviewer: So you live on a property, but you don't work on the property?

Respondent: He does.

Interviewer: He does?

Respondent: Yes. We live in one of the houses.

Interviewer: OK.

Respondent: I used to work for them. In, the guy had a Bed and Breakfast.

Interviewer: Oh yeah.

Respondent: I used to clean there.

Interviewer: Oh yeah.

Respondent: Until I got another job. And yeah. So.

Interviewer: OK. So... OK. So, you've told me, you, you've done it during the day.

Respondent: Uh-huh.

Interviewer: And how long would you do the wood cutting and collecting? How long would it be for?

Respondent: Oh, about five minutes.

Interviewer: Yep. Uh-huh.

Respondent: I usually do it in the late afternoon.

Interviewer: Uh-huh. And you were saying that you usually do it during the week, when you...

Respondent: Yeah.

Interviewer: ... your husband’s not at, not home. OK. And if these, if you wanted to, is there anything that would have helped you be more active in your leisure or spare time?

Respondent: I would have liked to have, to do a lot more things. It’s hard when you don’t have a licence. I, I'm on my learners at the moment, so that’s...

Interviewer: You really need to have a car and...

Respondent: I've got a car and all that. Just, the only time I do get to do hours is when my husband’s got time off. Or we go away on holiday. I usually do the driving. I hardly get out and do anything, because you just need transport.

Interviewer: Yes. Yes. Yep. It’s a big one.

Respondent: It is.

Interviewer: It is. Yeah. Yeah. So that certainly would have helped you.

Respondent: Yes.

Interviewer: And are there any other things that might have helped you? Like, in terms of, you were saying that things have changed here in terms of...

Respondent: Yeah.

Interviewer: ... the sorts of things you used to do, because things have closed down. Are they things that you would like to do that... if they were, if they were available?

Respondent: Well, the only thing I would like to do is find work. But it's hard up in this area. Because there isn't any. At the moment, everything gets on hold. So my health has to get on track first. And then I can look and, and we can, if anything comes up, well we can move. But at the moment, it's just, yeah.

Interviewer: Uh-huh. OK. Thank you. I wanted to talk a bit more about the physical environment that you live in.

Respondent: Yeah.

Interviewer: We've talked about where you live, and physical activity. And so, the physical active, environment that you work and live and play in, and when I talk about physical environment, I mean all the physical things that surround you. The roads, the trees, the houses, the shops, traffic. Those sorts of things.

Respondent: Uh-huh.

Interviewer: So some of these things are people in, living in urban areas have said might be relevant to their physical activity. So we're interested in finding out whether they're relevant to, to you, who living in rural

area. So, can you tell me a bit about how easy or difficult it is to be physically active in your area? Is it easy or is it difficult?

Respondent: For me, it’s probably hard, like I said, with transport. Other people, it would probably be easy for them to get out and get about. But, if, if anything was walking distance, it’d be fine.

Interviewer: But the, is there much in walking distance?

Respondent: No.

Interviewer: No.

Respondent: Not really.

Interviewer: OK. So that, yeah. So it’s a combination of not being able to drive...

Respondent: Yes.

Interviewer: ... and, because you really need to be in a car to, to be able to drive to places.

Respondent: Yep.

Interviewer: And also the fact that close-by, there really isn’t very much for you.

Respondent: There is a gym at the school.

Interviewer: Oh yep.

Respondent: Yep. But I don’t go to that.

Interviewer: Oh. OK. Now, that's what I was going to ask you, what's available and accessible in your community too. So, what sorts of things are here? There's a gym?

Respondent: Yeah, in the school area, but it's not usually open at weekends. So that's in, if people wanted to go to it through the week, they work. But I could go to it, but it's just, I like to have a, you know, someone else with me to, yeah.

Interviewer: And also it would be, if it's during the week and it's after school hours...

Respondent: You can't, I don't think so, if you can or not. It's got to be in between the school hours.

Interviewer: In between?

Respondent: In like, nine to...

Interviewer: Oh, it has to be in school hours, does it?

Respondent: Because it's inside the school.

Interviewer: Oh, OK. OK. Because I was thinking then that it's probably... would it be that the children need to use it during the, the day?

Respondent: Oh, they, they can use it, because it's in the...

Interviewer: OK. So it's during the day?

Respondent: Yeah.

Interviewer: OK. Right. And are there any other things available?

Respondent: Got a golf club. Or golf course.

Interviewer: Yeah. Any parks?

Respondent: We've got, we do have a park, but there's no swings or anything there.

Interviewer: OK.

Respondent: In this area, there isn't. But in [town] there is.

Interviewer: Uh-huh. And sport, sporting clubs, or recreation facilities?

Respondent: I'm not quite sure what... I don't think there is.

Interviewer: Uh-huh. Any walking treks?

Respondent: Not in this area.

Interviewer: Uh-huh. OK. So, there's not very, there's not really much in the way of what's available. No. But, given that, they're not very accessible either, are they?

Respondent: No.

Interviewer: And as you said, like, the gym isn't available weekends either, so.

Respondent: No.

Interviewer: So does having or not having places to be active in influence whether you're active or not? Would it help if you had more place, more places to be active in?

Respondent: Yeah. It would be.

Interviewer: Yep. Yep. Yep. And obviously if they were accessible as well?

Respondent: Yeah.

Interviewer: OK. And if you wanted to be more active, are there things or places that would help if they were available and accessible?

Respondent: Uh-huh.

Interviewer: What sort of things would you like to have? This is a bit of like a wish-list, isn't it? (Laughing).

Respondent: It would be nice if there was, if the gym was open, like, seven days a week. Somewhere you can go and, and meet friends and, you know, have a drink and, or a cup of coffee, say.

A park. You know, for the kids and things like that. Even though there's hardly any little ones here. Just more... I don't know. Probably... pool or, yeah, something like that.

Interviewer: Uh-huh.

Respondent: Oh, there's a pool at [town]. But I don't know if you're allowed.

Interviewer: How far is that?

Respondent: Be probably about... 20 minutes drive.

Interviewer: Uh-huh.

Respondent: But I don't know if you're allowed to use, if outer people are allowed to use it, or it's just for the, the community up there, or. Or, I'm not quite sure. But yeah.

Interviewer: And I guess the other one is if it's open all year or not.

Respondent: I don't think it is open all year. It's only through summer.

Interviewer: Yeah. Yep. OK. Now, another one's to look at destinations. Like...

Respondent: Uh-huh.

Interviewer: ... if I was to ask you about places you could walk or cycle, but you, you're walking from your home, what sorts of things would you tell me about? Like, shops or schools, or friends and, you told me about your family that you went to.

Respondent: Other destinations?

Interviewer: Yeah. Is there, like, is there a shop along here?

Respondent: Yes, there's a supermarket and a take-away shop. Used to be a pub here, but it closed down. There's a, the golf club.

Interviewer: And those are all places you can walk to?

Respondent: Yes.

Interviewer: Yep. OK. And does having destinations influence whether you're active or not?

Respondent: Oh, not really.

Interviewer: And if you wanted to be more active, would it help if there were more destinations within easy...

Respondent: Yes. Access. Yeah.

Interviewer: ... walking. Yeah. There would be?

Respondent: Uh-huh.

Interviewer: Yep. OK. Just sorts of things we've been talking about here. OK. Now, a bit about, it's called 'neighbourhood design', or the built environment that you live in, yeah? The non-natural features of your area. If I was to ask you about those, what sorts of things would you tell me about that were man-made? Things...

Respondent: That were man-made?

Interviewer: Things like footpaths and, if there are any. (Laughing). Roads.

Respondent: Only in the shop area.

Interviewer: Yep. Which is...?

Respondent: Just up, there's the walk way just over there.

Interviewer: OK. So there's a footpath that starts over there, is there?

Respondent: Yeah, and a walk, and you’ve got a little walk bridge.

Interviewer: Oh, OK.

Respondent: Yep. And it walks through the park and into the shopping, the centre of Ouse.

Interviewer: Oh, OK. So is the shopping centre on the main road as well, or just off?

Respondent: Yes.

Interviewer: It is?

Respondent: Yeah.

Interviewer: OK. Yep. So that’s, what, just down the road there?

Respondent: Yeah.

Interviewer: So, so there’s a footpath that starts and goes to the shop...

Respondent: Yeah.

Interviewer: Does it go beyond the shop?

Respondent: Yep.

Interviewer: It does?

Respondent: Yeah.

Interviewer: So it goes through...

Respondent: Right to the school.

Interviewer: Right up to school?

Respondent: Yes.

Interviewer: OK. So that would be, what? A couple, how? Distance-wise, would it be half a kilometre, or?

Respondent: I think so, yeah.

Interviewer: Yep. OK. Alright. What about lighting or streets that you know, that connect? Like, you can actually sort of walk down one street and into another one? You've got a few of those, have you?

Respondent: Yes. In, only in the...

Interviewer: Only in the township?

Respondent: Yeah, that's it.

Interviewer: Yep. And lighting?

Respondent: Yes.

Interviewer: Yep. So, if you wanted to walk from your place say, at night?

Respondent: Oh. No.

Interviewer: No. Because you're outside, you don't have lights where you are?

Respondent: No.

Interviewer: OK.

Respondent: Only in the township.

Interviewer: Yep. So you're fairly well, your house fairly well on the road? It's set, just set back from it?

Respondent: Yep.

Interviewer: Yep. OK. OK. So, does having, or not having, built features in your area influence whether you're active or not? Like, having, not having a footpath from your place?

Respondent: Uh-huh.

Interviewer: It does?

Respondent: It does. Yeah. Because you have to walk on the, on the side of the road, and it can be dangerous.

Interviewer: Yep. Yep. For, in terms of road traffic and stuff?

Respondent: Yes. Through the day, it's not too bad. But of a night, if you want to go somewhere, and just a bit, you know, for a walk or anything, I don't think you'd be able to.

Interviewer: Yeah. Yeah. It's too dark?

Respondent: Too dark.

Interviewer: You wouldn’t be seen, and you can't see.

Respondent: No.

Interviewer: OK. And if you wanted to be more active, are there certain features, man-made features that in your area that would help?

Respondent: No.

Interviewer: Other than extending the footpath out to your place? (Laughing). And lighting?

Respondent: Yep.

Interviewer: OK. Now, the other side of it is the aesthetic qualities of where you live, or the natural features, yeah? So if was to ask you about the aesthetic qualities, or the attractiveness of your area, what sorts of things would you tell me about?

Respondent: In what way?

Interviewer: Well, for instance, you know, the trees, or scenery. Or parks that you might have.

Respondent: I've got nothing.

Interviewer: Oh, OK.

Respondent: Yeah. Been here for so long, it’s just.

Interviewer: Yeah, it’s a bit bare out your way?

Respondent: Uh-huh.

Interviewer: Do you think your own local environment is aesthetically pleasing?

Respondent: No.

Interviewer: Your home? No. OK. And why would you say that?

Respondent: Because there's nothing there.

Interviewer: No trees?

Respondent: Got a few. That's all you can see. It's just trees and paddocks.

Interviewer: Yep.

Respondent: And it gets a bit boring after a while.

Interviewer: OK. So there's not sort of, more scenery and parks and things like that? OK. So do you think the aesthetics of your area influence whether you're physically active or not?

Respondent: Uh-huh.

Interviewer: OK. And if you wanted to be more active, are there features of your area that might help if they were more aesthetically pleasing or attractive?

Respondent: Uh-huh.

Interviewer: OK. So what might those be?

Respondent: I don’t know. In what way?

Interviewer: So, if you had for instance, if you had more trees, or you had sort of, more pleasant surroundings, walk, being able to walk rather than on the road, or. More parks, or.

Respondent: Probably more parks and more things to see and...

Interviewer: Uh-huh. Because I was told that, yes, there’s a park there, but you can't actually get down to the river there, so you can't even see the...

Respondent: No.

Interviewer: ... the river that comes through. (Laughing).

Respondent: No.

Interviewer: Yeah. So those sorts of things...

Respondent: Yep.

Interviewer: ... would help. Uh-huh. And, just one other area to ask you about. If I was to ask about safety in your area, what sorts of things would you tell me about?

Respondent: I think we’re pretty safe.

Interviewer: Uh-huh. Uh-huh.

Respondent: We hardly have any trouble.

Interviewer: You feel personal, personally safe here?

Respondent: Yeah.

Interviewer: And the, the other one would be about road safety. Do you feel safe on the roads?

Respondent: Yeah. I think so.

Interviewer: Uh-huh. And you sort of tend to walk on a fairly quiet patch of the road.

Respondent: Yeah.

Interviewer: OK. So does personal safety influence whether you're physically active or not?

Respondent: It does, yeah.

Interviewer: Yeah. So feeling safe, you feel OK to...

Respondent: Yeah.

Interviewer: Yep. And does road safety influence whether you're physically active or not?

Respondent: Yes.

Interviewer: Uh-huh. OK. So, yes. Because, it's fairly safe, the walks that you do. Then you feel like OK about it.

Respondent: Yep. Uh-huh.

Interviewer: And if you wanted to be more active, are there any personal or road safety issues that, if addressed might help you be more active?

Respondent: (Pause). Not that I know of. I can't think of anything.

Interviewer: Uh-huh. OK. Well, before we finish, is there anything else that we haven't covered about where you live, or your physical activity or the environment you live in that we haven't mentioned that you'd like to just talk about?

Respondent: No, not really. I just hope I helped.

Interviewer: Yes, absolutely. Absolutely. That was great. Thank you very much.

Interviewer: OK, so I thought we might actually start by talking a bit about the place where you live and what it's like to live there. So a bit about what it's like, your likes and dislikes.

Respondent: So the town, or our actual farm, or…

Interviewer: Yeah, so where are you exactly?

Respondent: So we're in [town] and our farm is basically just outside the town boundaries. So we're very close to the town. So we can actually walk to everything.

Interviewer: Oh, right...

Respondent: And [x]'s unique in that a lot of the farms run off a circle, so they sort of branch out a bit like the sun. So there's a lot of farms very close to the township.

Interviewer: Oh, that's how it was originally designed is it, interesting…?

Respondent: Was just designed… the town was put there and the farms sort of, yeah, settled off that.

Interviewer: So what's the sort of distance that you are from the town? You say walking distance…

Respondent: Oh, we're less than one kilometre.

Interviewer: Wow. So it only takes you…?

Respondent: Yeah, no, I could have walked today except that I was running late, so I needed to get here quickly [laughter]. I was doing other things, yeah.

Interviewer: So how long might it take you to walk to [town], if that's what… if you chose to…?

Respondent: Five minutes. Five minutes if I need to get something in a hurry.

Interviewer: Now the property. You're saying it's a farm. What sort of size is it?

Respondent: It's about 5000 acres. But it's in two parts. There's a part down here at [town] and a part up towards the [town] which is our summer run where we run our sheep in the Summer time. And then they get…we drove them down the road on foot.

Interviewer: Wow.

Respondent: So that's a bit different. And we also have poppies and cattle that graze down here. And we harvest… grow poppies down here at [town].

Interviewer: So a bit of mixed…?

Respondent: A bit of everything....

Interviewer: Yeah. That's good. So what's the length of time you've lived here?

Respondent: I've been married and lived here since…well I've worked here from 1993, so I don't do the sums on that one. That's about 17 or 18 years, yeah.

Interviewer: And your reasons for living here?

Respondents: I'm married. My husband. And he's been here…our children are seventh generation [town]. So he's been here for a long…he's been here forever.

Interviewer: And OK, so [town]'s your nearest centre and we've just had a look at how far it is away. So, as I was saying, with this sort of research, most of its been done in urban areas, OK. And people often refer to their local neighbourhood in an urban and city context. And I'm interested in actually understanding what the term neighbourhood means to women who live in rural areas, if it's relevant or not?

Respondent: Yeah, I think people that live, even if they're ten or 15 k's out on a farm, I still think they think the place where they go to get their mail or their children might go to school is still their neighbourhood.

Interviewer: So do you…does the word neighbourhood, what does it mean to you?

Respondent: Probably…well, I grew up in the city, so it's different to the neighbourhood in the city. So, I guess people up here don't call it neighbourhood, they call it community.

Interviewer: Ah, now that was the other question I had.

Respondent: So it's probably termed a bit differently....

Interviewer: Is it more relevant?

Respondent: I think community means that it's more widespread. So, for instance, community may extend over from [town] right through to [town], to [town], up to [town]. It's, yeah, it's the interaction of people where those people come from that make the community.

Interviewer: Right.

Respondent: Even as far as [town], even though it's in a different municipality, it's still kind of community because we have all the same industries, I guess. Farming makes a community in this area.

Interviewer: And so, neighbourhood isn't relevant, and we've talked about community. If you needed to actually put a distance around that area that you've talked about, what sort of distance, or you know…kilometers are we talking about?

Respondent: Oh, now I know that someone has actually, for the [region], they'd actually measured it, and I don't know what that distance is. It could be found out.

Interviewer: Just roughly.

Respondent: For us, I would say 60 kilometre radius. Approximately. Possibly more, though, 'cause [town] is a lot further away. And it's still part of our community; we'd consider it our community. You know, children come from [town] to the school at [town], so they're still part of the community, if that makes sense.

Interviewer: Yeah, no, that makes perfect sense. So it's about farming and schools and activities that bring you together.

Respondent: Uh-huh.

Interviewer: OK. Now I wanted to talk a bit about physical activity and what that term means to you, as I was saying it could be a very broad term, that's why we're asking people. So, if I said physical activity, what does it mean to you?

Respondent: For me personally, I always go for a walk most days, you know, go for a purposeful walk. But it also means filling up a wheelbarrow with wood, and gardening and maybe helping my husband with the farm work. So that's all physical activity. Yeah, that sort of thing.

Interviewer: And do you consider yourself physically active?

Respondent: Yes, definitely.

Interviewer: Uh-huh. And why?

Respondent: I guess I do try and purposely exercise each day for a four or five kilometre walk. Most days, not every day. But most days. If something else happens and I can't go, well I can't go. But I don't play team sport or do any specific sport. But walking, and just, yeah, getting the wood in and all that sort of thing.

Interviewer: And where does physical activity fit in your life? Is it an important priority, or just a priority, or not at all?

Respondent: Oh, I think it is important, because you've got children and you want to set a good example to children. And they're super fit and into all their team sports and they probably set an example to us, as well.

Interviewer: That's good. Because for, you know, the society we're living with these around, people often sit around these days. So it's very much a temptation.

Respondent: I think my husband has become fitter since we've had the kids because they play team sports and they do their cross country and their fun run so we do it with them.

Interviewer: Oh, that's great. So in our research, when we think about physical activity, we think of it as any activity that's lasted for at least ten minutes. So that's the sort of health benefit time. And it causes the body to work harder, you might be sort of huffing and puffing a bit, might make you feel a bit warmer, your heart rate comes up. And so thinking about this type of physical activity during the last two weeks, we're going to talk about some different parts of your life, just because it's easier to sort of make sure that we cover all of your life, basically. So in the past two weeks, have you been active at all at work for at least ten minutes at a time?

Respondent: Ah, yes.

Interviewer: Can you tell me about what you did?

Respondent: Oh, just filling up the wheelbarrow three or four times, sometimes the stockpiling of wood.

Interviewer: So that's at work?

Respondent: Oh, for work. Well, it's on the farm. So at work, when I'm [occupation], physically well I'm a [occupation… three quarters of an hour. Yes, but yep, so it's sort of two different works.

Interviewer: Yes, that's fine. That's good.

Respondent: Is that alright?

Interviewer: That's quite clear. No, no, no, that's quite clear.

Respondent: And taking the stairs at work at the [workplace] and that sort of thing.

Interviewer: Yeah, no that's good. OK, so you've told me about what you've done, the activities, the two different activities. Can you tell me about the intensity of it; whether you'd call those activities moderate or steady or vigorous?

Respondent: Oh, the [workplace] was steady. Work on the farm is moderate, probably slightly more than the hospital work. But not vigorous.

Interviewer: And the duration? How long might you have done those activities?

Respondent: [workplace] work was probably… And then filling up the wheelbarrow, stockpiling it for a rainy day was probably three quarters, to an hour. Yeah.

Interviewer: And the frequency, how often would you have done that?

Respondent: Well, I only work at the [workplace] once a fortnight, so, that was just that. And oh, the wheelbarrow job, because I consider that quite good for the muscles in the [laughs] arms; that's every day.

Interviewer: Yeah, I was just going to say [laughs].

Respondent: I mean I do two or three loads; it's usually only once or twice a week.

Interviewer: And when would this have been, during the day that you would have done these activities?

Respondent: Yes.

Interviewer: OK. And this is what you usually do, or is it one-off?

Respondent: No, it's pretty much in the…yep.

Interviewer: Uh-huh.

Respondent: The wheelbarrow one is a winter job, but…

Interviewer: I was going to say, does it change depending on the seasons?

Respondent: Changes a bit in summer. So it's probably more of a gardening physical thing or you know, helping out on the farm, doing something heavy.

Interviewer: There's always something?

Respondent: Yeah.

Interviewer: OK. And if you'd wanted to, is there anything that would have helped you be more active at work?

Respondent: No, not probably at the [workplace].

Interviewer: You were saying that, yes, 'cause you said you take the stairs.

Respondent: Take the stairs and…most people at work take the stairs. No, I probably…no not on the farm either.

Interviewer: In the past two weeks, have you walked or cycled at least ten minutes at a time to get to or from places?

Respondent: Yes, yes. As I said, I walk… I try to walk at least four times a week for four to six kilometers.

Interviewer: And so, what would be the intensity of that walking?

Respondent: It's a brisk walk, very brisk walk.

Interviewer: That'd be pretty vigorous?

Respondent: Yeah, so it usually takes me, I have timed it, about 55 minutes to walk about eight kilometers if I do the full circuit that I've got. But that's not, [laughter] yeah, I don't always get to do the full eight kilometers, but I figure any walk's better than no walk.

Interviewer: Yeah, yeah. So when you do your eight kilometers, you've got a…you were saying…

Respondent: I've measured it when I go out, sort of on the road on the farm and then back down and then around the town and then home again.

Interviewer: OK.

Respondent: So a bit of everything.

Interviewer: Right.

Respondent: Not a lot of hills at [town], so it's a pretty flat walk, but anyway.

Interviewer: And how…yeah, you've told me how often you do that, about three or four times…

Respondent: Yep.

Interviewer: And when did you do it?

Respondent: Do you mean the time of the day? In the mornings I try and go after I've taken the youngest one to school.

Interviewer: OK. And looking at where you do your walk, why did you do it there?

Respondent: Well, I go out the road 'cause it goes along the farm and I can look at the farm and see what's going on there and keep an eye on it and there's not as much traffic. But then I'll come back 'round and I'll go round the town and it's nice to bump into people, to see people on the town, the locals and…it's a bit of everything really.

Interviewer: A little bit of both. A little bit more of the social side of it, but with the road that you're walking on isn't too…hasn't got too much traffic on it.

Respondent: Uh-huh.

Interviewer: OK. And why did you do it there? What attracted you to that place?

Respondent: It was just convenient. Yep, convenient really, more than anything.

Interviewer: Would you have had any other options?

Respondent: Oh, yeah, there's lots of options. Anywhere. Yeah, lots of places to go walking around...

Interviewer: You've got a favourite…?

Respondent: I've measured it [laughter] and I know that that's how far I've walked, so it's a creature of habit.

Interviewer: [Laughs] fair enough. And were you alone or with others?

Respondent: No, I prefer to walk on my own.

Interviewer: And why's that?

Respondent: I can do it at my pace that I want to do it at. And often I sort of just plan the week or plan the day, think about things and switch off. Or I might have my phone with me and I might make a couple of phone calls while I'm walking so I don't have to do it when I get back. Yeah, all sorts of reasons, but I do prefer to walk on my own.

Interviewer: And does this change depending on the season or the time of the year?

Respondent: Yes. If it's really wet and cold I don't go for a walk. And if it's really, really hot I might go earlier in the day or it might be a shorter walk. So, yes it does. But most days…it has to be really extreme to not do it.

Interviewer: And you've got the option, I guess, of if it's a few times a week of which days…?

Respondent: Yep.

Interviewer: And if you'd wanted to, is there anything that would have helped you use walking from places, what…if there is anything that would help you with walking?

Respondent: Not really, no. Good shoes, you need good sneakers.

Interviewer: Now in the past two weeks, have you been active around the house or yard – so at home, inside or outside for at least ten minutes of the time? Now you've already told me about the wood activity; are there any other activities that you want to tell me?

Respondent: I've had a lot of cooking and a lot of…yeah, more than usual cooking and running around doing some things like that, that was physical.

Interviewer: And the intensity of that?

Respondent: Fairly…more than average, yeah. Constantly on your feet and I was tired at the end of the day.

Interviewer: Yep. And the duration – how long you might have done it for?

Respondent: Probably, it was a few days, Thursday, Friday, Saturday; probably six or more hours each day.

Interviewer: Big days.

Respondent: Yes.

Interviewer: And how often over the last two weeks would you have done it?

Respondent: Probably the week before there was a day that was like that. So probably four days out of the fortnight that I was normally busier than I would be.

Interviewer: OK. And was that during the day?

Respondent: Yes.

Interviewer: And during the week or the weekend?

Respondent: Mainly to the weekend, it was on the Saturday.

Interviewer: And did you do that activity with others or by yourself?

Respondent: I was helped with other. It was a…basically I was asked to cater for a very large funeral and provide all the food and do that. So other people helped me. But it was quite busy because we had to gather all the catering equipment and do all the cooking.

Interviewer: That's a very big job.

Respondent: So it was a big job. It doesn't sound big, but it was big. It was for the community, so…Is that the sort of thing you mean? That sort of activity?

Interviewer: Yep.

Respondent: OK.

Interviewer: Yep, anything that you consider as…

Respondent: It was an out of the norm activity.

Interviewer: And is this what you would usually do, or is it…?

Respondent: Well, I would do other…helping the community with other things, but it was pretty much a one off this time.

Interviewer: Yep. And does this change depending on the season or the time of the year?

Respondent: Yeah, I guess it does, yep.

Interviewer: In what way? Is it more or less?

Respondent: It's something that I don't get asked to do very often, but, yeah it just depends.

Interviewer: Just depends.

Respondent: Outside influences I guess you'd say.

Interviewer: OK. And if you wanted to, so just having a think about being at home and being inside and outside; is there anything that would have helped you be more active around the home? Sounds like you're pretty busy.

Respondent: Yeah, I am pretty busy. My dad came to stay with us a few weeks ago and he said, you never sit down! So, yeah, no I don't think anything else. Sometimes I think it would be nice to have a bit more time to sit down and do something else.

Interviewer: Yes, well that's the last question as part of this – have you been physically active for at least ten minutes during your leisure or your spare time.

Respondent: Yeah, don't get a lot of that.

Interviewer: Yeah, exactly.

Respondent: So what was the question – have you been physically active…?

Interviewer: … active for at least ten minutes during your leisure or spare time.

Respondent: No, my leisure and spare time I do try and sit down – is that OK?

Interviewer: Absolutely (laughing).

Respondent: No, my spare time is sit down time.

Interviewer: As I said, these questions are just to make sure that we capture every part of your life. This is very usual for women being busy doing lots and lots of things. So when it comes to whatever small amount of

spare time you have, it's not something you want to go rushing and doing something.

Respondent: At the same time, I guess, when I go for my walk, that to me is also leisure time, so, two things in one.

Interviewer: Yes. And that's the other thing that comes out is that…

Respondent: I enjoy my activity and I enjoy my exercise, yeah.

Interviewer: Yeah. But often women are saying that, you know, you don't do one thing for one reason, it's sort of…yep. So that's fine. Now I want to talk a bit about physical environments now. We've talked about where you live and your physical activity; now physical environment is about where you work and play and live. And when I talk about physical environment I mean the physical things that surround you: the roads, the trees, the houses, shops, traffic. And some of these things are things that people in urban areas have said that they might be relevant to their physical activities. So that's why we're interested in asking, whether they're relevant for you. So can you tell me a bit about how easy or difficult it is to be physically active in your area, your community?

Respondent: Oh, look. Our area's really good place to go for walks. I think anyone that's able to walk, there's no excuse for them not to be able to go for a walk and be active in that way. And actually a lot of people in our town do, you know. They're all trying to be a little bit more active.

Interviewer: It's a beautiful town…

Respondent: Yeah…

Interviewer: It looks like it's well organised for…

Respondent: It is quite well organised. I can give you an example; there's one lady who used to be extremely large and she made up her mind that she wanted to change that, and bit by bit she's just slowly walking, walking, walking and slowly changing her eating patterns and you can see a difference. And she just walks around the town. So there's…yeah, anyone that wants to walk, can walk.

Interviewer: Yep.

Respondent: There's not a lot of other options for sport or physical activity, although there are there's golf and different things. If you want to go for a walk, you can go for a walk very easily in [town]...

Interviewer: So what would make it easy? Like, what makes it easy?

Respondent: What makes it easy? It's very flat. And there's roads, you can walk up the middle of the road, there's very little traffic, you know, most people walk right down the middle of the road when they go for a walk. There's not…a lot of the places there aren't actually footpaths, but you can walk on the road and feel safe. You can even go for a walk at night, there's street lights and I guess the safety thing makes it easy. Most people know people so you feel safe out walking.

Interviewer: And what I want to do know is talk about different aspects of physical environment, OK?

Respondent: Uh-huh.

Interviewer: First up is availability and accessibility of places to be active. So if I were to ask you about places to be active in your area, what sorts of things would you tell me about?

Respondent: I can tell you there's a golf course so that people can play golf. I know that just a few people go up together and organise an indoor bowls afternoon once a week – they can do that. I'm not sure if it's still going, but there was a lady taking Tai Chi.

Interviewer: Oh, was there? OK.

Respondent: I don't think that's going at the moment.

Interviewer: So there's a hall here that she can…?

Respondent: There's the hall, there's the school gymnasium. There's also the town hall and that sort of thing. And I know some people on farms that have tennis courts. They have social tennis just for a bit of fun. And of course there's other things that people would consider to be called sports, there's fishing, shooting. Just bush walking. There's lots of options for people.

Interviewer: And I think I noticed I don't know if it's open or not, there's a sign to a swimming pool…?

Respondent: A swimming pool. Yes, there is a swimming pool. In the summer it's opened.

Interviewer: Ah.

Respondent: So that's accessible to people within the community or visitors to the town. Usually from late November to the end of April. So it's only just

shut. So yes, I forgot about the swimming pool. There's two ovals – the school oval and the football oval, that you can go and just kick a footy there or have a hit of cricket, or…

Interviewer: Any other parks, or…?

Respondent: There's the main park in [town], there's swings and play equipment for kids and things like that.

Interviewer: And are there any particular walking tracks or paths?

Respondent: There's not actually any particular walking tracks as such but lots of farms will let you onto their farm to walk. For instance we've got a waterfall that runs on the river and people often ring up and say can they walk through the paddocks to the waterfall. So that's OK. It's not a set track. As long as they let us know they're going. There's all sorts of different... And that would be unique to our place. There's other things on other farms that people can go and do.

Interviewer: So it's a rural community that want to…

Respondent: There's all sorts of different things. So people have horse-riding and yeah. So it's probably not the run of the mill. But in [town], it's unique.

Interviewer: No, it's an interesting little place, absolutely.

Respondent: And in fact, the cleaner at the local school is just starting to teach the kids boxing, so, different again – but it's something, a different option.

Interviewer: There's lots of things happening. And access – are these places convenient, are they easy to access?

Respondent: Yeah, I'd say they are fairly easy to access. And even the golf club's probably the most expensive thing, if you wanted to get set up.

Interviewer: I was going to ask about cost or…

Respondent: But to join, it's not very expensive. I think they'd lend you…I think people would lend golf clubs. I think most things are fairly accessible to most people if they wanted to get involved.

Interviewer: And does having places to be active influence whether you're active or not?

Respondent: Doesn't influence me. But I think there's people that, yeah, because it's an option they choose to do it.

Interviewer: Yep, yep. And if you wanted to be more active, are there things or places that would help if they were available or accessible?

Respondent: Yeah. I think if someone wasn't playing golf and they wanted to play golf they'd be made to feel welcome and people would be there to help, or…yeah. That sort of thing.

Interviewer: Are there any other activities in the…for you?

Respondent: I can't think…personally there's nothing like…I would quite like to do Zumba but there's nothing in [town]for that. But I do know there are people in [town] that travel to [town] once a week and go to Zumba. So there are other options that are outside of [town] that are available and if you choose to travel a bit further…

Interviewer: And that would take, what is that, about 20 minutes?

Respondent: Yeah, 20 minutes to [town].

Interviewer: Yeah.

Respondent: This is for my age group. There's also football for younger people.

Interviewer: Any activities that you want to tell us about is really, really handy to know.

Respondent: So there's two local football clubs, one that is called the [x] which encompasses [town], [town], [town]; and there's also the [x] Football Club. And there are children from age seven right through to adults that play for both those clubs. And they travel to them and travel doesn't seem to worry anyone. And that means that kids, if they wish to - girls and boys, can participate in a team sport. And there's also [town] Soccer Club for juniors which a lot of children from [town] have participated in over the years. And dancing, there's a dancing troop in [town] and children travel, so…I don't think the travel and the distance is a huge discouragement for people. If children wanted to participate, or adults and it's there, they will travel a certain distance to get to things. Which is good. Probably there's other things, but I can't think of them off the top of my head.

Interviewer: That's fine. Destinations – if you were to ask about places you could walk to from your home, what sorts of things would you tell me about? You've already told me a bit about…

Respondent: Yeah, well, there's a nice café and the park and you can walk to all those. Could even walk to the golf club. There's a little picnic spot down by the river, you can walk down to that. You can walk to the

school. There's a little cottage nursery in [town], you could walk to that.

Interviewer: OK. So you've talked a lot about the services and things like, and the recreational facilities. And I guess it's the way it's organised too, with the farms as friends as well, that they're in close proximity as well.

Respondent: They are.

Interviewer: And does having destinations influence whether you're active or not?

Respondent: It doesn't really influence me, because I'll probably find a way of doing something active anyway. But, yeah, so that would be my answer to that.

Interviewer: Yeah, it doesn't stop you, but it sort of, it's sometimes nice to incorporate it as well, yeah. OK. And if you wanted to be more active would it help if there were more destinations within easy walking distance?

Respondent: Probably if there was something, for instance, like Zumba or something like that a bit closer. And tied in with a day that I was free, yeah, I would probably do something else as well.

Interviewer: OK. And I want to talk a bit about the actual sort of neighbourhood design, OK, so that's the built or the non-natural features of your environment...

Respondent: OK.

Interviewer: A bit like the roads and things like that. So if we were to ask you about these… what were the non-natural features, the built, man-

made features in your environment, what would you actually tell me about?

Respondent: Oh, well, the swimming pool? Is that the sort of thing you were…? Yep, the swimming pool and the school gymnasium. And the town hall I guess has things there. And the park. The [town] recreation ground with the football stadium.

Interviewer: And you were saying there weren't footpaths out where you were, but there's footpaths in the town?

Respondent: There's footpaths, I'm still not quite sure why…there's gravel footpaths up both sides of the roads of the main street for most part. And then for one street back from the main street there's footpaths for part of that. For [x] Street there's part footpath. But sort of stops and starts.

Interviewer: And then, you're on a… are you on a dirt road or…?

Respondent: We're on a sealed road. We're on a sealed road from [town] through to [town], and that's a sealed road, but the laneway into our actual farm is a gravel road.

Interviewer: OK. And you were saying that you can actually walk on both sides, so there's enough room?

Respondent: Yep, there's plenty of room.

Interviewer: And there's a…?

Respondent: Yeah, two big stock trucks come along fully laden I can get out far enough off the road onto the grass side.

Interviewer: And in [town] itself, you know, like when you do your routine – what about the streets and the connectivity how do they connect in terms of, can you actually do a walk through the town versus, you know, some towns it's really the main road and that's it…what about [town]? Does that have streets that connect?

Respondent: Yeah, it's really well set out and there's lots of streets that connect. Lot's of streets. It's a very well laid out town. The streets are pretty much all in one area, I guess, and there are other areas, but they can pretty much all be connected.

Interviewer: Just looking at different things that affect people's walkability and that's one of the things that people have said, you know, like you've got a main drag basically and there's nowhere else to walk from.

Respondent: Yeah, no there's even the backstreet which is, people just call it the backstreets of [town]. Even there you could go for a walk.

Interviewer: Yep, OK. And lighting and things like that?

Respondent: Yeah, for most part there's street lights, so yeah, you could go for a walk at night and feel safe.

Interviewer: OK. And what about for you to your farm, it's about a five minute walk, is there adequate enough lighting for most of the way?

Respondent: I couldn't walk out the road at night, there's no lights on the road at the [x] Road, but if I wanted to go for a walk at night I could walk around the town and there'd be lights.

Interviewer: OK. And do these features which are part of the built environment actually influence whether you're physically active or not, do you think?

Respondent: They don't influence me, but they probably influence other people, for instance, especially the swimming pool in the Summer, that influences lots of the younger people to be active in the Summer. And if there are events at the gymnasium at the school, it influences…it probably doesn't influence me.

Interviewer: And then the other side of it of the being able to…the way that the roads and the streets are organised, sort of does help for you when you're walking…?

Respondent: Yes, it definitely helps, yep, definitely helps.

Interviewer: OK, so the other side of it is the natural environment and if I were to ask you about the aesthetic qualities or the attractiveness of your area, what sorts of things would you tell me about?

Respondent: For the most part it's nice and quiet. It's bush and paddocks around and it's just quite peaceful.

Interviewer: It's a very beautiful spot.

Respondent: Yeah. Sometimes you notice them and sometimes you don't notice them. Some days I don't absorb and some days you do. At the same time, if it's raining, the smell is amazing.

Interviewer: And do you think your local environment is aesthetically pleasing and attractive?

Respondent: Yes, I do.

Interviewer: And near your home?

Respondent: Yes. But even if you walk around the town there's lots and lots of gardens you walk past and yeah…

Interviewer: And do the aesthetics of your area influence whether you are physically active or not?

Respondent: I guess they do without thinking about it. I guess, yeah, it makes it more pleasant if I walk and not see a heap of rubbish.

Interviewer: So things like rubbish and graffiti aren't a problem here?

Respondent: No, no.

Interviewer: And if you wanted to be more active, are there features of your area that might help if they were more aesthetically pleasing?

Respondent: Not for me, but maybe if there were better footpaths some of the older people possibly could; but I don't think at the moment there's anything that stops them. But maybe that's something in the future they can look at improving is some better footpaths.

Interviewer: OK. Now the last area is about safety. If I was to ask you about safety in your area, what sorts of things would you tell me about? You've already mentioned…

Respondent: I mean the lighting's good and the footpaths are reasonable and the roads are reasonable. Yeah, I don't think there's…I guess…no I don't think there's anything they could really do to improve the safety. The

children ride pushbikes around the town after school, ride their bikes to school. Yeah, I guess, maybe in that instance for pushbike riding, there could be maybe more signs for people coming from outside of the area that don't know the area. So, for instance, truck drivers, there may be a sign: please be careful of children on bikes. Something like that. That's the only thing I can think of that would make it safer for physical activity.

Interviewer: And does personal safety actually influence whether you're physically active or not?

Respondent: I guess it does. You're not going to do something if it's not safe. So yes.

Interviewer: And in this context and in your community you're feeling safe, so that's fine.

Respondent: Yes.

Interviewer: And does road safety influence whether you're physically active or not?

Respondent: Yes, but that's…road safety is good, so I feel safe.

Interviewer: And you've already told me if you wanted…well you were talking more about the community, about whether they want kids on bikes. But for you, if you wanted to be more active, are there any personal road safety issues that if they were addressed might help you be more active?

Respondent: No, I don't think so.

Interviewer: OK. It sounds a very nice place to be.

Respondent: It is a nice place, it's got its downfalls, but yeah.

Interviewer: So there's only one final question before we finish: is there anything else you'd like to tell me about of where you live, physical activity or your environment, that we haven't covered today.

Respondent: I don't think so, but I think if anyone's going to be able to do something to improve the general physical activity in a community, I think the best place to start is at the school. And I've still got children at this school, and I still think there's lots to be done to help and not just physical activity but their general wellbeing, eating habit and all that sort of thing.

Interviewer: Set them up early.

Respondent: Set them up early, because I think that's where we all need to start.

Interviewer: Absolutely.

Respondent: None of us are perfect, but sometimes you…

Interviewer: But it makes it a lot easier if it's a way of life, doesn't it?

Respondent: Yeah, and if people are healthy, there's less strain on services.

Interviewer: Absolutely, you're the [occupation] [laughter]. I come from health too, it's very important. You think, well, you don't want people getting into that hospital end. It's far better just to focus on staying well.

Respondent: And you see kids up at the school and the PE teacher does his bit, but they need to be taught not just a little bit of PE, it's all about looking out for what you put in your body. There's a whole lot of things, but yeah. It's everything. So that's probably the only other thing I'd add, if I can add something, that's…yeah.

Interviewer: Yeah, no that's really important. Again, so you think of things in much more of a life context of…

Respondent: And those children can then go home and hopefully teach their families something they may not have been taught. Teach their mums and dads how to cook something healthy, or…saying let's go for a walk after tea, or…yep. Little things can make a difference.

Interviewer: Absolutely. Yeah, it's a good point. Thank you.

Respondent: That's alright.

Interviewer: OK. I thought I'd start by talking a bit about the place where you live and what's it's like to live there. The likes and dislikes for instance...

Respondent: What sorry?

Interviewer: Your likes and dislikes.

Respondent: Oh right, yes OK. I've only recently moved here which was last October...

Interviewer: Oh OK.

Respondent: So that last 18 years I've been living on an island off of the [region] called [x] Island.

Interviewer: Oh right.

Respondent: So many changes for me in the respect of from living right on the water and a [x] island opposed to living now on a rural property and

having my own house as well and having actual dirt with worms in it. I mean, it's a totally different environment obviously...

Interviewer: Can you just tell me where you are? Are you in the broader [town] area?

Respondent: I'm in [town], our road...four kilometres off of, out of [town] and our road [x] Road. And then you drive up to the top of that and then I've got a property of 27 acres up there so...

Interviewer: OK. So, wow, that's very lovely.

Respondent: It is very lovely. And my biggest likes are one I've got my own property, it's deep up in the forest and I have electricity where I was on generator power before which is a bit... many conveniences only being four kilometres from an actual town instead of a boat ride to the mainland for a town so and I can't find any dislikes, I'm really quite happy here. Yes I wouldn't think of any dislikes at this point put it that way.

Interviewer: Oh great. And what were your reasons for picking [town]?

Respondent: Oh it had probably to do a bit financially, that properties and housing was a little bit less expensive than more coastal and I wanted land, was my big thing so I wanted no neighbours sort of environment.

Interviewer: Have you got actually sort of farm land or a mixture of bush and...

Respondent: Oh no, it's a tiny bit of pasture and the rest is all rainforests, the nuclear forests so yes.

Interviewer: Beautiful.

Respondent: It is.

Interviewer: In research with women living in urban areas, people often refer to their local neighbourhood. And I'm interested in understanding what the term neighbourhood means to women who live in rural areas and if indeed it's relevant? So what does the word neighbourhood mean to you?

Respondent: Well I suppose being part of a community and only four kilometres from the actual town of [town] I feel very part of that community. I've joined a couple of volunteer groups, actually one's in [town] and one's

in [town] but that's still the greater community area. Just before I had a job, just to get to know people so there land care groups and the [town] community centre I'm part of that and go to the men shed so I guess it is that community... you can be as isolated as you want to be or you can be as involved as you want to be as well because there's that many opportunities volunteer wise that you can get to know people by coming here and not knowing anyone. And then my partner went to [country] so I was, for about six months I've been on my own which has been fine, I'm used to that so...

Interviewer: It's been a change in terms of it's now you having to get to know your community...

Respondent: Yeah that's exactly right. And I guess it's a luxury, I usually move to a place and have a job and then you're working full time and that becomes your community, your full time work and you don't get to venture out into your surrounding areas, but I had the luxury of this time coming here and deciding 'Oh well I'm not going to so much work right away, I'm not going to focus on getting a job right straight up and get to know the people and the surrounding area first'. So that was a bit of a luxury to be able to do that the opposite way around.

Interviewer: So I was actually going to ask you about the word community as well. So does the word community mean anything different than neighbourhood?

Respondent: Oh well, I worked out at [workplace] which is roughly about a 45 minute drive further south which is even more rural and the people down there say 'Oh I haven't been to [city] for two or three months' so it's actually working with people who are even more isolated in an area and so that community we're a great bunch of people but we're making our own fun, so to speak. So we get together, go for hikes and we're discovering new caves and spend time doing various things like separate communities I suppose.

Interviewer: Yes so just going back then does the word community, is that relevant or would you prefer to community having relevance?

Respondent: Well I suppose it's just work colleagues, yeah I suppose that's a community, that's separation yes. It would be separate to obviously what's here.

Interviewer: Yes. So is around [town] neighbourhood and is that what you're saying is neighbourhood or community?

Respondent: Community.

Interviewer: OK so it's not that neighbourhood has that bit that is relevant...

Respondent: No I mean neighbour, well we've met our neighbours and they had a big barbeque to introduce us around but we don't have a great deal to do with neighbours per say but more parts of the community. People here are active in the community, put it that way.

Interviewer: Oh OK. So neighbourhood you don't see as such a relevant term?

Respondent: No.

Interviewer: No, it's community. And community is... there's a number of definitions for you about community. One is [town] itself and the community of like minded people and the other you're talking about is further afield as well like the work community at [workplace] which...

Respondent: Yeah and then it goes the other direction as well so the community that I do Landcare with is [town] and [town] so...

Interviewer: So if you had to put a distance on those activities which are a part of community what on average would you say, how many kilometres would that be?

Respondent: Well its 45 kilometres to [town] and to [town] its 13 so the in total opposite directions.

Interviewer: OK. So that's about 50 – 60 kilometres. OK. That's great. Thank you. Now we get onto physical activity and I'll actually talk about physical activity and ask you what the term means for you.

Respondent: What it means to me? Well I've always remained physically fit for pretty much my whole life. I had a back injury about two years ago so

I slowed down a little bit and have altered my type of activity but saying that I've been lucky enough that the more active I am the better I feel. So like I said just alter, slow down a little bit but now I'm back up to speed again so when I think of physical activity on a daily basis, I'll get out for at least an hour and I'm building trails on my property so we're making a loop and we hooked up to forestry and now my partner and I we've got a loop that goes for an hour and a half so we make ourselves do that at least once a day and we've got mountain bikes so we go biking, we've got kayaks as well so on our off time we'll go biking or hiking most generally or kayaking.

Interviewer: That's great. It sounds ideal. I'm intrigued on how you've got your trails on your property that's great. That's a really good idea.

Respondent: We keep physically fit slashing through all the blackberry to get them and then when you finally get them you can walk them.

Interviewer: Yes and what a good idea. You don't do it just for the sake of it. So do you consider yourself physically active?

Respondent: Yes absolutely.

Interviewer: And why's that so?

Respondent: Because it's a mental, I mentally make sure that it happens every day. It's part of what you do on a daily basis, drink water or have a coffee you know that's something I consciously make an effort to include in my daily regime.

Interviewer: And that's what women are actually saying to me when I'm talking to them which is actually a new concept about physical activity which is really interesting rather than physical activity is a once a week thing that I do and I go specifically to a certain place for example a gym, where all women are actually saying it's something we do every day. And where does physical activity fit into your life? Is it interest, no interest, high priority or priority?

Respondent: Oh it's high priority.

Interviewer: Great. Now going to talk a bit more about physical activity and in our research when we think about physical activity we think of it as any

activity that lasts for at least ten minutes. That is the evidence of health benefit and causes the body to work harder than normal so you might find your heart rates up, you might breath a little bit heavier, you might feel a little bit warmer and so thinking about that activity over the last two weeks, I'm just going to talk about different parts of your life not to actually segment your life but just to actually make sure we cover all the different activities that you're involved in. So I'm just going to start with work first. So in the past two weeks have you been active at all at work for at least ten minutes for a time?

Respondent: Absolutely yes.

Interviewer: OK and could you tell me what you did?
[text removed because of identifying information]

Respondent: Oh two weeks, oh on average four days a week.

Interviewer: And when? Would this be during the day?

Respondent: Yes.

Interviewer: And is that what you usually do or is it a one off?

Respondent: No it's what I usually do.

Interviewer: And does it change depending on the season or the time of year?

Respondent: Yes it does...

Interviewer: OK.

Respondent: The [x] season.

Interviewer: OK and which season's that? More summer?

Respondent: Summer is definitely more busy.

Interviewer: Yes OK. And if you wanted to is there anything that would help you be more active at work? Sounds like you're pretty active as it is.

Respondent: [text removed because of identifying information]

Interviewer: OK so that's another activity that not only you would do but it would offered?

Respondent: If it could be offered I would love to do it yes.

Interviewer: If it could be offered, yes OK. Now in the past two weeks have you walked or cycled for at least ten minutes at a time to get to or from places.

Respondent: Walked yes.

Interviewer: Yes? And could you tell me about what you did?

Respondent: Trails, although I hiked part of the [x] Trail and then trails around my house.

Interviewer: OK. And how would you describe the intensity of that activity? Steady, moderate or vigorous?

Respondent: Vigorous.

Interviewer: How long would you have done it for?

Respondent: Well with the [x] trail and then we hiked up parts of Mountain [x] and that was four hours for that one, [x] Trail that was four or five hours on that and then even just our look that we've made around here is an hour and fifteen minutes.

Interviewer: Wow. OK and how often would you have done those, you mentioned three activities there?

Respondent: Well the two we did one hike with the [x] Mountain that was one week and [x] Trail was the next week and then this trail around the house per week about three, four times.

Interviewer: When did you do it?

Respondent: During the day.

Interviewer: During the day? And would that be at weekends, before or after work?

Respondent: Probably it would be the morning but I can't remember my roster but actually the [x] Peak and the [x] were on the weekend.

Interviewer: Why did you choose to do it at those locations?

Respondent: Hobby or enjoyment.

Interviewer: Yes. And were you alone or with others?

Respondent: Oh no with my partner.

Interviewer: Is that what you usually do?

Respondent: Yes.

Interviewer: Does it change depending on the seasonal time of the year?

Respondent: It doesn't really change. It's more of a conscious effort when it's cold out but we'd still... you'd wake up and the house is cold and you'd go

‘OK let’s go for a hike, just to warm up’ sort of thing and that’s what we’re finding that living here is, you can stay inside and it’s raining and it’s cold and do nothing all day but you have to ignore it and just get out because it does clear up. And sometimes it’s to warm up even.

Interviewer: Yeah I understand that one. I haven’t lived here that long myself and it’s certainly activity every morning taking the dog for a walk around Mt [x] is great because you come back really warm. If you wanted to is there anything that would have helped you walk or cycle to or from places?

Respondent: Anything that would have helped me? Like what? What do you mean?

Interviewer: Given that you’ve been very active, it’s saying well is there anything else given those activities that would have helped?

Respondent: No.

Interviewer: In the past two weeks have you been active around the home? Inside or outside? And could you tell me about what you did?

Respondent: Well clearing those trails, those are right on my property so that’s one and then we’re clearing a fire break around the house so we’re cutting trees and then we’re working on the house itself. Renovations...

Interviewer: A lot of work.

Respondent: Yes.

Interviewer: So what would be the intensity and of course there’s the usual sort of house cleaning and stuff like that but what would be the intensity of those activities?

Respondent: What’s the middle one?

Interviewer: Moderate.

Respondent: Yeah I’d just say moderate.

Interviewer: Though sort of bashing your way through blackberries can get a bit vigorous.

Respondent: Yeah it can’t but not vigorous the whole time.

Interviewer: OK. Now the duration in the last couple of weeks, how long might you have done those activities?

Respondent: Well Sunday we worked outside all day, we worked inside when it was raining and went outside, so we were outside for a period of four hours working in the bush and then we went for a big hike after that. Well a big hike, on our trail so an hour and a half. Yes steady work throughout the day but that's normal for us.

Interviewer: So how often do you that then?

Respondent: Well it's so dependent on work because my job I drive an hour there and an hour back so if I can get out if I'm not working early we'll go out for a hike in the morning so for a two week period I would say on average about four times a week.

Interviewer: And thinking about these activities around your home inside and out, you've told me some are obviously during the weekend and some are dependent on what time of the day when you're working, I just need to check why would you do them at those times of the day other than the other times?

Respondent: That's the only time I've got.

Interviewer: That's the available time that you've got.

Respondent: Yes exactly right.

Interviewer: And this is what you usually do?

Respondent: Yes.

Interviewer: Does it change depending on the seasonal time of the year?

Respondent: For the last six months when [husband] been gone I've been consistently doing that so no it seems... you just work even more during summer 'cause it's lighter longer and early, I probably would be spending more time outside really.

Interviewer: If you'd wanted to is there anything that would have helped you be more active around the home either inside or outside and it sounds like you already are?

Respondent: I can't think of anything else.

Interviewer: I'll just tell you to make sure we catch everything that you're active about, have you been physically active for at least 10 minutes during your leisure or spare time?

Respondent: Oh yes.

Interviewer: Can you tell me about what you did?

Respondent: It sounds so boring but we go hiking, but even spending the day in [city] and we'll put that many things on our list. We'll park at a central parking, a dollar parking and then we'll walk everywhere. So we'll walk from up [x] Street down [x] markets, we just walk everywhere so that's part of our day.

Interviewer: Yes it's a very walkable city that way.

Respondent: Oh it is, it's lovely.

Interviewer: So now you've told me in your leisure time about the walks that you've done but you also mentioned at the beginning you did some cycling to? So was that during the last two weeks?

Respondent: No. I haven't been cycling lately. Last time was probably about a month and a half ago I went to cycle from here to... I went to the markets in [town] so I just put my panniers on my bike and off I went. And I've got a back pack for my [x] so I take her with me as well.

Interviewer: Oh right. So that would take you on the main road, the main highway?

Respondent: Oh yes that was the whole road.

Interviewer: How was that in regard to cycling with traffic?

Respondent: No I won't do that again. And then I helped with clean up Australia Day and at [town] and then I hiked or I cycled over from [town] bend there was a short cut, a road that comes over the mountain, it comes over [x] road, I don't know if you know that area?

Interviewer: I've been told that there's some back trails that you can ride?

Respondent: You can't ride, I'm telling you right now. I walked and pushed my bike the whole way but it was lovely coming down the other side.

Interviewer: That's the thing. Often women are saying about cycling is that if they used to do it when they move rurally they don't do it because of the traffic but the other thing is that the only thing that's flat is the main road which has got all the big trucks and things, anywhere else it's really quite steep.

Respondent: Well I was hoping that with this job that I got, 40 kilometres is not that... it's a bike ride but when I drove down and had a closer look and looking at the shoulder of the road, there's no way you would ride a bike on that road. It's just too dangerous, the way people drive as well. If I could change anything as far as that, it would be fantastic camp bike riding trails but that's a farfetched dream...

Interviewer: It's worth contemplating because it's ways and means, often things come with those sorts of idea but it is also something that women have been saying about, you actually need safe ways to be able to ride or even work.

Respondent: Exactly. Even walking from my house down into [town], even the road on our road is not the best. There's areas that you quickly go, well you go to the other side or there's steep ditch but if there's two vehicles coming you could quite easily get hit by a truck or something...

Interviewer: But aren't roads in terms of [town], it's one of your main roads isn't it?

Respondent: Yes it is.

Interviewer: Is it bitumen where you are as well, it's all covered?

Respondent: On [x] that's only a side road so that's only about a kilometre that's gravel road, a dirt road and that's quite fine but the trip into town is... we've been, where we hike behind our house goes into forestry so we're actually I suppose trespassing in the forestry but anyway we go cut through there and we did see little signs for the [x] Trails so we're slowly discovering where that goes behind and hooking up but it's so vague, I mean if they've got a [x] Trail I got online and am trying to get the guide book and if that was clearly maintained and sign posted, that would be fantastic for men, women, kids to ride a bicycle or hike but its, you ask people and they say what's that? You don't know about the [x] Trail but you can see why they don't because it's difficult to find.

Interviewer: That's the question I was wanting to ask you, with your leisure or spare time is there things that would help you be more active? And those are the sorts of things that you've already told me. Now the last bit is the physical environments. I've talked a bit about where you live and your physical activity but now I want to talk about physical environments about where you live and work and play. By physical environment I mean all the physical things around you, road, trees, houses, shops, traffic and some of these things are things that people living in urban areas have actually said are important for their physical activity. So we want to ask whether it was relevant to for rural people. Can you tell me first of all a bit about how easy or difficult it is to be physically active in your area and why?

Respondent: I don't think it's difficult at all really. There are plenty of books on different trails but then if you don't like hiking, I suppose that can be an issue, but there's bush walking clubs so you can join up with other people if you're a single person.

Interviewer: Would you normally drive to get to these walks?

Respondent: I looked into all the information about this bush walking club and while my partner was gone and you meet and you go to Mount [x] so you drive a fair distance but they're all over the place. But it would give you a better insight of what's around here just to join up for a year and then do it on your own after that so it does take a bit of an effort but they give you a three month little booklet on everything that's upcoming and who to contact and carpooling and so that's really well organised. I plan to join up on that one.

Interviewer: OK. There's just a couple of areas I wanted to ask about in particular, availability and accessibility of places to be active. If I were to ask you about places to be active in your area what sorts of things would you tell me about?

Respondent: Well we do have the [town] aquatic centre and they've got a good span of activity there whether it be a Zumba class or aerobics class. They've got boot camp so they've got a range of activities and it's,

well I thought it was affordable, I mean it is but then it could be maybe have winter rates that would encourage... because once I figured out how much it is per day because I said to [husband] 'Oh you should join this' because I joined it when he was gone and he was like 'Well how much is it?' and it comes out to be about $380 per six months and so OK I thought it was cheaper for a year but it's not cheaper to go for a year and then if you times that by two that can be quite expensive for a year. So then we went out and bought gym equipment and so now we're set up for here. We bought a little brace for our bike so we can bring the bike into the house and set it up on stand and we got a workout machine. So it's actually cheaper to buy your own but then of course it takes more of a conscious effort because sometimes when you walk into a gym you may not feel like working out but once you get there your OK and you feel like it then because people are around you but I guess I would say that maybe surrounding rural places would have better rates I suppose, certainly in the winter months. I don't know if they can afford that or not...

Interviewer: Encourage community. That's a very good point. So you've got the aquatic centre but what other recreational walking tracks have you got in your area?

Respondent: You really need to go and get a map and find them yourself, so that's what we've got hiking books and it tells you where places are. But some of the places like [x] Falls which is outside of [town], we haven't been there because the gates been locked so it's not always accessible. So I think once you're a hiker you know where to look for things but maybe for people that are new into hiking or finding activities they might find it a little bit more difficult. I suppose better information would be...

Interviewer: Would be helpful. Yes I must admit being a new person from interstate to, you find your way and you have a certain amount when you're used to it but it takes a bit of finding out sometimes.

Respondent: Well I want to join an orienteering or rogaining. Have you heard of that before?

Interviewer: Yes.

Respondent: That's quite a fun activity and you're learning navigation, how to read a compass and you find different points and it can be quite physically challenging against teams and I think that would be a fantastic activity to take place down here but...

Interviewer: And you're usually finding different areas than the common trails too.

Respondent: So once I've started my new job in the last few months so once I sort of catch up and have extra time maybe I can find a source that helps to implement that around here.

Interviewer: Yes. So having places to be active influence whether you're active or not?

Respondent: Yes, definitely I would think so.

Interviewer: And the other question which you've already answered is if you wanted to be more active are there things or places that would help if they were available or more accessible? And we've talked about that. Destinations. If I were to ask you about places you could walk or cycle to from your home what sorts of things would you tell me about?

Respondent: Like I said cycling is out. I have found going down forestry roads cycling would be the better idea. So that's what we'll do instead of main roads obviously and hiking there's quite a few trails and that around down here but saying that I was not too... what's the national park which is over by [town], only 10 kilometres north of [town]. There are some fantastic, the [region] and I hiked that during the day and I camped the night and then hiked a little more the next day and then went home. So everything is pretty close proximity so nothing's that far away.

Interviewer: So obviously you'd have to get into your car to do that. Are there anywhere that you can walk to, like would you walk into [town] to shops or...

Respondent: Of course. There's a couple of cafes and if you need to pick up something from the hardware store or the [supermarket].

Interviewer: So does having destinations influence whether you're active or not?

Respondent: Absolutely.

Interviewer: And if you wanted to be more active locally, for example be able to walk or cycle from home, would it help to have more destinations within easy walking or riding distance?

Respondent: Yes.

Interviewer: There's a couple of more areas. Neighbourhood design which is the built or manmade features of an area. If I was to ask you about them what sorts of things would you tell me about?

Respondent: What features there are?

Interviewer: Footpaths, lighting, you know are there?

Respondent: Oh yes. Well there's everything I think you would need in a community on a smaller scale as in this community so you've got your groceries, your bank, your butcher, lolly shop...

Interviewer: And the general thing women have said about [town] is that within [town]on itself the main area, there's footpaths and things like that and it's OK...

Respondent: Oh yeah it's well set up. And it's safe.

Interviewer: Which is actually unusual in terms of other rural areas, they don't have that...

Respondent: Well that's true.

Interviewer: It's small and it gets out of the town. And so do the built features of your area influence whether you are physically active or not?

Respondent: Yes it gives you, if you want to walk into town and join up with somebody for a coffee you can do that, so yes.

Interviewer: And if you wanted to be more active are there any built features in your area that would help?

Respondent: You could put up more restaurants at night but apparently there's a couple that are... there's a cafe that's going to open so that would be

nice to be able to walk in for a dinner, or you could put a little cinema or that sort of thing.

Interviewer: That would be nice. So there destinations and activities that would attract people. That's great. The other side of it is the aesthetics or the natural environment which of course [town] very beautiful. If I were to ask you about the aesthetic qualities or the attractiveness of your area what sorts of things would you tell me about?

Respondent: It's got a nice big park behind it so you do see families with picnics and sporting, so that's always nice to see and people walking their dogs. Probably the footpaths do go a little bit more toward [town] on their way out of town that way but certainly not down our road too far and they cut across that park area to go to the community centre and that southern design. And they've got a [x] walk or something...

Interviewer: So it's not just...

Respondent: No it's nicely...

Interviewer: Because it is a big park it's not just a park people actually use it to navigate different parts of [town] as well.

Respondent: Yes.

Interviewer: Again destination stuff. Do you think your local environment has aesthetically pleasing and attractive?

Respondent: For the most part, yes.

Interviewer: And near your home what's it like?

Respondent: Oh it's very rural so...

Interviewer: Very beautiful...

Respondent: Pastures up [x] Road it's pastures with cows and horse so then you get further in and it's all forested. So very aesthetically pleasing.

Interviewer: Do the aesthetics of your area influence whether you are physically active or not?

Respondent: Oh yes.

Interviewer: And if you wanted to be more active are there features of your area that might help? If they were more aesthetically pleasing or attractive?

Respondent: I can't think of how...

Interviewer: Yes it's beautiful as it is.

Respondent: Yes and you could have a...

Interviewer: As you were saying before, it draws you out to go and have a hike or a walk.

Respondent: Well you could have bigger mountains or more... the rivers right there so no I think everything is right here.

Interviewer: Can you actually walk along the river at [town]?

Respondent: No, you'd have to go [town] is basically the closest area.

Interviewer: OK so the river goes through but there's...

Respondent: Well it goes... what is it called [x] Road so where does it skirt out? It's, I don't even know. You drive, it must skirt out somewhere, I'm not quite sure where, I'm going to have to walk along the river one day.

Interviewer: One woman mentioned that it would be actually nice to have a bit better access to the river in [town].

Respondent: Yes, true. Behind the aquatic centre and it's coming toward [town], obviously it gets smaller and smaller but it does thin down quite a bit but there is a beautiful place and I've said 'God they should have that accessible to the public' where you could have a picnic or a walk area so they could make a walk area from even the aquatic centre at the [x] hotel pub thing right down into [town]. That's what Landcares' trying to do from the middle of [town] to make a footpath, that's what we've started on and it goes for about maybe a kilometre so far but they want to make a footpath all the way to [town].

Interviewer: So where does that go from now?

Respondent: Down by the [x] centre. You can see it, there's a picnic table down there but it goes along the river.

Interviewer: So along the to... in the direction, now which direction [town] or the other?

Respondent: No it's going to [town]. It would be good to eventually go the other way as well.

Interviewer: Indeed.

Respondent: They need to make that into a biking walking trail would be fantastic.

Interviewer: I've been talking to women up in [town] and not only in that regional centre but the local outlying areas, more rural areas and they are all very keen on there's a bike and walking track from [town] to [x] Beach...

Respondent: Oh? That's good.

Interviewer: Yes, that seems to be very well used. The idea is the council are interested in extending it along so that would be even better. OK one last area is safety. If I was to ask you about safety in your area what sorts of things would you tell me about?

Respondent: I would feel quite confident to walk into town on my own at night, to and from in the dark by myself. So I have no fear at all of, there's kids that ride around but they're nice kids so no problem at all.

Interviewer: So it's a safe community in that way so it's so... if I were to ask you does personal safety influence whether your physically active or not?

Respondent: Well it would but it doesn't in this case. I even walk through the forest and there's nothing, I come from a place in [state] where you make noise 'cause there's bears there...

Interviewer: I was going to say you come from big mountain country.

Respondent: We were talking about that the other day on the trail, it's like, there's not much here. But saying that you do run into the snakes in summer so building trails as soon as February, March when the snakes are breeding at that particular time I saw a couple and being on my own, well that's a safety issue so you stay out of the forest then...

Interviewer: And that's another thing that people, one they have actually mentioned to. That one. Does road safety influence whether you're physically active or not?

Respondent: Absolutely. That's...

Interviewer: You already told me about the cycling but I imagine even walking it's a bit tricky too in places. And so if you wanted to be more active are there any personal or road safety issues that if addressed might help you to be more active?

Respondent: Oh clearly not bike trails that are on the road because I don't think people have a wider road they're not going to care if you're there or not, but separate trails from the road adjacent to the road like the trail that's already started from [town] to [town] that are totally separate would be ideal. And the [x]Trail as well.

Interviewer: It's sort of building on the existing trails as well isn't it? It's just sort of being a bit mindful about how they can be better used. And just to, before we finish up is there anything else that you'd like to tell me about where you live, your environment or physical activity that we haven't covered today?

Respondent: It's definitely with the, especially the winter months it's obviously more difficult with the shorter days to stay in that keeping fit mode. It's so easy to come home from work at four or five o'clock and then just sit in front of the TV and get some warm soup and do a big shut down and then plus you're wearing that many more clothes so OK well I could cover up my fat if I have to, nobody's going to notice but you can't do that. It all catches up with you. So just to, you wake up in the morning and it looks a bit rubbishy outside but just to get out there anyway and not just be, I've got a couple friends that they say they just get into this depression. It's like play cards, let's do something so it's definitively more of an effort in winter but you just have to combat that. Even at the community centre and I could bring that up to [woman] there she's very good. Even to have although it sounds goofy but trivia nights or bingo nights in [town] community centre, that's fun stuff.

Interviewer: It's a way people can get together.

Respondent: Just to play in winter. That's about it.

Interviewer: Thank you very much that's great.

Interviewer: So I thought we might actually start by talking a bit about the place that you live. OK? And what it's like to live there. So can you tell me a bit about what it is like living in [town]? What you like, what you dislike?

Respondent: I was attracted to [town] for its location in terms of it being in the middle of two larger centres, so you don't actually have to be in the larger centres, but you can have easy access to either or, and within 20 minutes of each other. And so in that regard you can have all the things you actually need, but you don't have to live right next to it, so industry, and services, retail, all that kind of thing. But for me, [town], the main attraction was the [x] River.

Interviewer: Oh, OK.

Respondent: Yep. Or the River [x] as it's known on maps and so forth. The history, the natural asset that it is was the main attraction, and where I live in [town] is only 250 metres from the river. So I kayak, so I can just drag it down there and hop on, and off I go, and go and explore.

Interviewer: Wow. That's beautiful.

Respondent: Yeah, so it's mainly that. [town] is also relatively flat in [state] terms; although you look all around the window here you'll find hills everywhere. But I cycle, so I can cycle with relative ease, without having to climb up hills, which really does... is a challenge if you've not done it before.

Interviewer: Hmm. It is a challenge. Hmm.

Respondent: So there's that. And being relatively close to a beach, I have dogs, and being mindful that some beaches have restrictions, and rightly so they should, because we need to protect wildlife – we all have to share these things. But I've got access to ones that are designated for dogs, and I have dogs, so that made it easier for me.

Interviewer: So these were all things that...

Respondent: That I considered. And I wanted to live in an older home, which I do, close to the centre of town. It's five minutes' walk into the centre.

Interviewer: OK. I was going to ask you. So you're on a house and house block?

Respondent: Yes. Yes.

Interviewer: But it backs onto... not far from the river. It's a...

Respondent: Oh, you can... yeah, you walk into the river.

Interviewer: OK.

Respondent: And you can go upstream and you're in rural area within 20 minutes of paddling. I mean everywhere you look around here it's a rural setting; we are in a rural town.

Interviewer: Yep. So you're five minutes away in terms of walking to the centre, is it?

Respondent: Yeah. Walking, yeah.

Interviewer: Wow.

Respondent: So footpaths and... that access was important to me. I mean in outer areas, to walk pets, or walk yourself even, if you're... you know, the roads a little bit too hazardous for that, because a lot of country folk can't walk their dogs where they want to if they... unless they walk in a paddock. It might not be their paddock, so they can't...

Interviewer: Hmm, because of the traffic and there's no actual footpath. The road just drops off.

Respondent: The traffic's... there isn't, yeah. And you can't expect that, and you haven't got enough money to put that infrastructure in, but that's one of the things... I work in natural areas all day. I don't need to go and own one myself. I can come here and have access to it whenever I want, so that was part of the choice.

Interviewer: Hmm. OK. And the length of time you've been here?

Respondent: I've been in [town] since February this year, although been in [town], which is nine kilometres west of here, for over 12 months, looking for the right place to buy.

Interviewer: Yeah, so you took your time.

Respondent: Yeah. Yeah, well I wanted to weigh up location, services, all sorts of natural attributes, social attributes, all those things – I wanted to weigh it up before I stuck my neck out.

Interviewer: Hmm. Fair enough. Now, in research with women living in urban areas, in cities, and like most of this research has been done around urban areas, people often refer to their local neighbourhood. And I was interested in understanding what the term neighbourhood might mean for women who live in rural areas. So what does the word neighbourhood mean for you?

Respondent: Right at this very minute it's the immediate neighbours, one on each side, but you get to know others who are close by that are in the same locality if you like. And that might be [town] just up the road, [town] just up the road – or it's actually south, even though it's uphill. I refer to most of [town] as a neighbourhood. It's only a small town. There's not a lot of people here.

Interviewer: Hmm. And how far would... you mentioned [town], how far would that be up the road?

Respondent: Oh, three minutes, four minutes.

Interviewer: Drive?

Respondent: Yeah. Yeah, you can cycle there, you can walk.

Interviewer: OK.

Respondent: It's fairly close. It's all close. Even [town], I know people in [town], it's kind of my neighbourhood, it's my neck of the woods.

Interviewer: Hmm. OK. So OK, so in terms of actually sort of getting a distance around that, is that your neighbourhood within kilometres, or can you give me a...

Respondent: Yeah.

Interviewer: OK, so sort of... what sort of... how far would it be?

Respondent: Well, I've lived in rural areas all my life, and your immediate neighbourhood is anything up to ten k's. You know, it's much the same here.

Interviewer: Yeah. Yeah, so it's much the same?

Respondent: Yeah.

Interviewer: Yep. Fine. OK. And what about the word community, what does that mean to you?

Respondent: Lots of people busy doing things for nothing. [Laughter].

Interviewer: [Laughter].

Respondent: Community – it's... yeah, there's a... I look at a community as... well, as a whole bunch of people doing different things, not necessarily being paid money. I get involved in community activities, so we all kind of look out for each other, always do. And that's quite a powerful thing in a small community, because it's generally the size that brings people together. It has its downfalls, because a lot of people know your business – and it doesn't bother me because I've got really nothing to... not bothered with that, but some people find that quite off-putting. But there is a sense of community in [town]. The history of [town] as it started out in the [x] days is testimony to that, and it's still strong. Yeah.

Interviewer: Hmm. And community – does that have a distance with it as well?

Respondent: Not a lot. Not a lot. I mean I've found living here that most people don't travel a lot. And they don't like going any more than five or ten minutes from home to work.

Interviewer: OK. So community might be smaller than neighbourhood in that sense for you?

Respondent: Yeah, possibly. Possibly. You know, much the same thing.

Interviewer: Or are we talking about community being [town]?

Respondent: Yeah. [town]. Yep.

Interviewer: Yep. No, that's fine. Yep, OK. So I want to talk a bit about physical activity now, and that can be really broad. So it's actually... I wanted to ask you what the term physical activity meant for you in your life.

Respondent: Generally exercise, which I don't think a lot of people do enough of. I do – that's just the way I am. Although in some areas that you choose to live, some of those options are a little bit harder, and therefore probably people are less likely to get involved in those kind of activities. Whereas that's part of the reason I've chosen the location that I have, is because I can very quickly access all the things that I would do out of hours. But then again, as I mentioned before, I've

chosen the work that I do so that I am physical in my work day, rather than then come home, now I've got to go and find 20, or 30, or an hour really, what people should be doing – this 20 business is not much at all – but that's my choice. I've elected to do that, on purpose – I've specifically done that so that I can.

Interviewer: OK. And you've talked about some of the activities that you enjoy. Do you consider yourself physically active?

Respondent: Yeah, definitely.

Interviewer: Yeah. And why?

Respondent: You just feel healthier. I don't smoke, I don't drink excessively – just what's known as a social drinker. And even then you just know that you haven't... if you haven't done your regular routine for a couple of days you feel it.

Interviewer: You can feel it.

Respondent: You feel it. And you need to make up for lost time, and I do. So yeah, I'm not... I don't think I'm the norm. I'm not a gym junkie as they can be referred to. I don't go to the gym. I've got an outdoor gym that I utilise, so... yeah, and that requires motivation. It doesn't just happen. People look at me and say, "Well aren't you lucky." Well, yes I am, but it's my choice to be active, and it's your choice not to be, so don't kind of have a crack at me. [Laughter]. Yes, I am active. Yeah.

Interviewer: Hmm. OK. And where does physical activity fit into your life? Is it... obviously high priority? Very interested in...

Respondent: It is. It is. It starts for me in the morning when I do walk my dogs for about 20 minutes, half hour, and that's how it starts. I sometimes cycle before work. I'll take my bike with me, and I can use a circuit in [city].

Interviewer: Oh, OK.

Respondent: Or there's bike paths there that are relatively safe, so I can do... I don't do that every day. I don't actually do it every week, but I do do it, weather dependent. Then go to work. And sometimes, I'd say half

of my work is outdoors, and that will be property assessments where I'll go onto farms, and we might walk for a kilometre or two.

Yes, so I do... half of my time is spent doing that. And then at home, I get home, I may take the dogs for another run, I may not. But on a weekend I'll either go kayaking, or more cycling, walking – because there's the [x] Range not far from here, and there's walk trails all round.

Interviewer: Hmm, how far is it?

Respondent: Well, [x] Range, you can do a 12, 13 kilometres. You go to [town] and then go through it.

Interviewer: Oh, OK. So it's not far at all.

Respondent: No.

Interviewer: No, no. OK.

Respondent: No, it's good. And the beach, you can walk a couple of kilometres on a stretch of beach here, and so I do that on a week... I take the dogs on a long walk on the beach at least once a week, sometimes twice.

Interviewer: Yeah, yeah. They are... dogs are always great. I've got a [dog] myself.

Respondent: Right. Yeah, they're busy. Busy.

Interviewer: And every morning, every morning... [Laughter].

Respondent: Yeah, busy.

Interviewer: Yeah. Yeah. So that's great, because now I'm going to ask you a bit more detail about the physical activity, and so in our research, when we think about physical activity, and we think of any activity that lasts for at least ten minutes and causes the body to work harder than normal, heart rate up, breathing, a bit more puff – feeling warmed up. So with that definition in mind, I just wanted to have a look at just different parts of your life over the last couple of weeks. OK?

Respondent: Uh-huh.

Interviewer: So thinking about over the last couple of weeks, have you been active at work for at least ten minutes of the time?

Respondent: Yeah.

Interviewer: So, definitely so?

Respondent: Definitely. Definitely.

Interviewer: Would you call that moderate or vigorous in intensity at work?

Respondent: In the last couple of weeks it's been vigorous because I've been on this project site, where I've been walking in wellie boots in mud and slush, carrying things for... you know, like one day I did about nine kilometres walking doing that, and then hiking through river systems, climbing over things, doing survey work, and that's pretty hard going because you're...

Interviewer: It's all fairly vigorous exercise isn't it?

Respondent: Your body's hot, you've got to take stuff off so you don't overheat, and then the moment you stop put it back on.

Interviewer: Yeah, because you don't want to cool down.

Respondent: Yeah, exactly. So the last couple of weeks definitely has been quite intense really.

Interviewer: Hmm. Yep. And duration?

Respondent: Hmm, all day.

Interviewer: All day.

Respondent: Hmm.

Interviewer: And how frequently would that be?

Respondent: Well, two or three of the days starting at about eight, you're vigorous until morning tea and you have your 15 minute whatever, and you carry on until lunch time, have another 15 minutes, and that finishes at four o'clock. So it was consistent.

Interviewer: So it's consistent over...

Respondent: Yeah, it wasn't a ten minute burst. It was like a three hour burst, and a two hour burst, and a...

Interviewer: Yeah. And that's fairly typical of the sort of work that you do?

Respondent: Yeah. Yeah.

Interviewer: Yep. Yep. And does it change depending on the season, or time of year?

Respondent: Yes. Yes, it does.

Interviewer: OK.

Respondent: Yeah. We're just moving into the [x] season, where we do a lot of... all our [x] projects that have been planned, are starting to become implemented on the ground. And that'll only go for a month or two, and then that'll fade out and you'll start doing other things. And there are times in the office, January, where we've just come back off holidays and things are just starting to build momentum, and we don't do much at all. Do a lot of thinking, but not a lot of work physical stuff.

Interviewer: Hmm, hmm. So there's a bit of a balance.

Respondent: There is, hmm.

Interviewer: Depending. Yep. Fortunately. I think there is this business that there needs to be a time at work where you actually have a chance to have a look at what's happened for the year, clean it up, before you jump into the next year. And certainly with environment you've got...

Respondent: Oh, it does.

Interviewer: It's very driven by seasons. Yep.

Respondent: Yes. It's also driven by funding rounds, so...

Interviewer: Oh, yes. [Laughter]. Yes.

Respondent: Yeah. That's...

Interviewer: That's the hard one.

Respondent: You know we're funded by the [funding body], so...

Interviewer: Does all of your funding come via...

Respondent: Most of it.

Interviewer: Most of it. Yeah.

Respondent: Most of it. And there's criteria that we have to respond to in regard to acquitting that, and that is a cycle.

Interviewer: Yeah, that's a cycle, and it's a cycle of collecting the data, and all of that sort of stuff I bet.

Respondent: It is. Yep.

Interviewer: Hmm. OK. So if you had wanted to, is there anything that could have helped you be more active at work? If you had wanted to. It sounds like you're quite active.

Respondent: Yeah, I don't... I probably don't need to. Although if we had access to certain, I suppose, equipment, or a facility close by, that would be really, really helpful, and it would get others involved as well. It's just this... there's a lot of effort involved in finding facilities and actually going to facilities, and then using them.

Like here, we're coming into the winter months where there's less light, so you finish work in the dark, and you get to work in the dark, in most cases. And so to fit some sort of a regime in, it's very discouraging for those who are less motivated to go and do these things. I can't cycle in the dark. I'm not going to, it's too dangerous. But if there was a facility that was under lights, or cover, I'd be into that like a rat up a drainpipe. And I reckon others would as well.

Interviewer: Hmm, hmm. It's a common thing that's starting to come out, is this sort of seasonal difference and for cycling and even walking, how visible... no, actually for cycling particularly actually.

Respondent: Yeah. There's a lot more people wanting to get involved in cycling around, and they do, and there's an imminent risk in the roads here, and that's not a criticism because we don't have the population, we don't have the taxable income, we don't have the funding available to go and put cycleways everywhere.

Interviewer: Hmm, hmm.

Respondent: Open chequebook we could do some nice circuits around here.

Interviewer: Yeah, wouldn't it be nice. Hmm, hmm.

Respondent: Yeah. And it would get people healthier. I don't know how much money goes into the health system that gets pumped in there for no reason at all, because people are lazy, they get fat, they get sick, and taxpayers have got to pay for it. That's my beef. I'll stop now. [Laughter].

Interviewer: Uh-huh. No, fair comment. I think it's important to... wellbeing is much, much, much more important.

Respondent: Of course it is.

Interviewer: It's a lot easier too.

Respondent: It is. It's cheaper. If we start right, it's cheaper.

Interviewer: Absolutely.

Respondent: Anyway. Hmm.

Interviewer: Hmm. OK.

Respondent: Yes, a facility – I would use a facility like that.

Interviewer: Yes, a facility. So in the last two weeks, have you walked or cycled at least ten minutes at a time to get to and from places?

Respondent: Yeah.

Interviewer: Yeah, OK.

Respondent: Yep, yep, yep.

Interviewer: And can you tell me about what you did? You've talked about cycling, but was its intensity moderate, or vigorous?

Respondent: Vigorous. Yeah. No, I push myself. I'm getting the heart rate going.

Interviewer: Yep, yep. And duration? How long?

Respondent: Oh, cycling would have been maybe an hour, with sort of bursts of speed where you just... you feel your heart, your pulse go up, and you get hot, then you slow down, you get hot, then you slow down. So sort of every five minute bursts I guess, and then slow down.

Interviewer: Hmm. And how often would you have done that in last couple of weeks?

Respondent: In the hour? That's been twice. Yeah.

Interviewer: Uh-huh, uh-huh.

Respondent: But the walking has been... been a bit more involved in the walking this last couple of weeks.

Interviewer: Hmm, because you've been describe... well it's a bit hard; you've been over on [x] Island.

Respondent: That's correct. Yes. And no cycling.

Interviewer: But a sort of typical week, couple of weeks for you, because you were saying that you would usually take the dogs for a walk before you go to work.

Respondent: Yes. Yeah, and because I didn't have that, I didn't need to at the end of the day with the walking that I was doing, and under strain – I was

doing that under strain, because we were walking up steep embankments with equipment, walking in mud, muddy terrain, with boots which weigh a lot, and when they've got lots of mud on there's more stuff you're carrying. I lost a kilo and a half in two weeks, so... hmm.

Interviewer: Hmm, hmm. OK. Now, where would you have... OK, so when and where would you have cycled and walked?

Respondent: Cycled had been here around [town], around the flatter trails, and the walking was all on [x]... oh, even walking here, because I walked to the river. Before I went to [x] Island I was still walking to the river. And then took the dogs to the beach, walked along there. And then on [x] Island, I was just walking on farms, and along rivers.

Interviewer: Oh, OK.

Respondent: Yeah. So that was... you know the site that I was working on was four kilometres long, so I did that a couple of times in the day.
[Someone enters the room].

Interviewer: Oh, hi. [Laughter].

Respondent: So I was doing that a couple of times during the day, delivering bits and pieces of product from one end to the other, and surveying that as well.

Interviewer: Yeah. OK. And with the cycling, you mentioned a... was that cycle track that you were talking about?

Respondent: No, this is just on the roads here in [town].

Interviewer: Just on the roads.

Respondent: Yeah.

Interviewer: OK. Because...

Respondent: I try and keep off the busy ones, and just... there's a circuit that I can do at a certain time in the morning that's relatively quiet.

Interviewer: Yeah, that's a bit quieter.

Respondent: And you do go over busy roads. You go through a traffic light, two traffic lights and one roundabout, which is a bit of a challenge. But if

you do it early enough you don't get too many people there, so it's not so bad.

Interviewer: OK.

Respondent: It's a bit hard riding when you've got to go this way, and then that way.

Interviewer: Yeah. [Laughter].

Respondent: You haven't got enough arms.

Interviewer: I know. [Laughter]. You're always constantly... I've just started. I haven't ridden a bike for a long time, because when I moved out into a rural area the traffic was just [expressive noises].

Respondent: Yeah. And it's too risky. Too risky.

Interviewer: Yep. So I've only just started getting back into riding, but in [city], where it's like [expressive noises], it's a challenge.

Respondent: Yeah. Well it is. I've gone to [city], and I've actually taken my bike there.

Interviewer: Oh!

Respondent: And I've... there's a nice cycleway from...

Interviewer: Hmm, yes. It's finding...

Respondent: Near the bridge.

Interviewer: That's right.

Respondent: Going north?

Interviewer: Yeah. That's right.

Respondent: Yeah, like I'd been doing it... yeah, love that. So I've done that.

Interviewer: It goes for quite way It's nice. So it's just finding those sorts of places, and as you say, that's something that you don't have to be watching traffic as well.

Respondent: True. Well that one, you had... obviously to cross roads, but its well sign posted, and you can get halfway across safely.

Interviewer: Yep.

Respondent: Stop, and get over the rest of the way. And you can get a good stretch done before you get to the next crossing, and so it's quite... I really

enjoyed that. And I've... surf – learning to surf – so I've taken my board down there, down to...

Interviewer: Oh, wow, its good surfing?

Respondent: Well, it's... I like it here because we're protected by the [sea], and we don't have massive fronts like you have... on the west coast we do, because they have the [event], and all the rest of it. That's full on intense over there, but on the north it's easy. Hmm, it's nice. And that's really good for getting your heart rate up as well. People think surfing's a doddle, but it's not.

Interviewer: No. Yeah, I agree with you. OK. So just where you've... we were just talking about where you've cycled and walked, why did you do it there? What attracted you to that place?

Respondent: Well the walking, it was all work, so I was attracted there because I developed the project, had no choice. Like to have a choice, but that was my choice. The cycling, again I chose to live in [town], at the site that I do, because...

Interviewer: So you could do all those things.

Respondent: So I could do that with ease. You know, I have my pushbike mounted on the wall inside the house in a wide passage, so I look at it all the time. So it's encouraging me to just grab it and walk out the door, rather than oh, I've got to go and unlock the shed; oh, I've got to take the cover of; oh, I've got to do this, I've got to do that.

Interviewer: Yes, yes. Yes. I know.

Respondent: It's all those sorts of things, if you don't have them handy and accessible, you're less likely to utilise them.

Interviewer: Yeah, you're absolutely right.

Respondent: So I've got my house set up with all that sort of stuff.

Interviewer: Hmm, hmm. And does this change, the cycling and the walking, depending on the season or the time of year?

Respondent: Yes. Yeah, yeah. Winter is a little bit tricky if you don't have facilities, because it's... it's not even... it's not so much the cold. I mean when

it's wet you can't go out – well you can, and I do, but it's not safe. It's a lot safer if the roads are dry.

Interviewer: Hmm, dry conditions. Yeah.

Respondent: Yeah. Yeah. And inherently [state] is wet. But again, if there was a facility, or even if it's a cycle path, I'd still... I've cycled in the rain, I've surfed in the rain, I've kayaked in the rain – you still go and do things.

Interviewer: Hmm. And the other one of course is if you're doing it before – like walking before work, it's got to do with the light as well.

Respondent: Exactly. So winter you are less likely to do it.

Interviewer: Hmm.

Respondent: And that's where people here, I find, get caught out, and get lost. They might have momentum during spring, summer, autumn, and then in winter they just...

Interviewer: Hmm, it's finding your way through that. Hmm, hmm.

Respondent: Yeah. Yeah. And it doesn't bother me a great deal, I'll always.... I'm a fidget, and carry on, so I'm always relatively active anyway. But I can see that it would be very, very difficult for a lot of people who aren't as motivated, or aren't as up with it, and see the benefits of being persistent with it.

Interviewer: Hmm. Yeah.

Respondent: Yeah. It's easy to go home and knock back a couple of beers, and I do that.

Interviewer: [Laughter].

Respondent: I do.

Interviewer: Yeah, yeah.

Respondent: But I... because I do stuff, so I can have my beer. I don't not spoil myself with those sorts of things. You know you can... it's all in balance.

Interviewer: Yeah. Absolutely.

Respondent: Yeah.

Interviewer: Yeah. Absolutely. Oh, and if there's... if you'd wanted to, is there anything that would have helped you to be cycling and...

Respondent: I would do a lot more of it if there was a cycle path. In fact there's one being constructed between [town] and [town], I think. Hmm, the [x] project. That is exciting, because there'll be kids there, there'll be families can do short walks, they can do long walks, you can go from one end to the other. You can just peg a section out that you want to do, and just do it. And there'll be other cyclists there. It's just fantastic. Looking forward to that.

Interviewer: Great. Good. It sounds like there's quite a bit of activity around sort of pathways and... for walking and cycling.

Respondent: Yes. Yeah. The Local Governments are recognising I think, that we haven't got the healthiest community around.

Interviewer: No, [state] hasn't actually.

Respondent: It's pretty shocking.

Interviewer: Hmm.

Respondent: The statistics, as I read...

Interviewer: And rural communities too.

Respondent: Yeah. Well, that's because they work so hard tending the potatoes and the cattle, and all the rest of it, and forget about...

Interviewer: Hmm, and don't have time for anything else.

Respondent: And don't have time for themselves. Whilst that's they're busy, they're busy, they're always attentive, they're always attentive, but it's not physical over a period of... you know, short burst to get the heart rate up. They're just consistently plodding, and a lot of people can't keep up with them because they're just... they work long hours, but it's not healthy, it's not healthy for their mind, it's not healthy with their family, it's not... when it's dark then it's worse. So yes, we have the utmost unhealthy people probably in [country], and that's more so in the northwest I believe, which is sort of a little bit east of here, to [expressive noise] all the way down to [town]. Yeah, so it's a bit sad.

Interviewer: It is.

Respondent: Hmm. And we don't have to be. Don't have to be. And it's not all about facilities, but to some extent if there were some that would improve...

Interviewer: It's a bit of a complex venture isn't it?

Respondent: It is. It is. Yeah, definitely.

Interviewer: Complex world we live in now, it gets to be a bit more complex.

Respondent: Well it's fast paced, regardless of whether we're here in [state] and people think it's a doddle, it still has a pace. And...

Interviewer: Yep, it does.

Respondent: Yeah, it's difficult to... it's... when everything comes through... any bit of information that you can extract that you need for your day-to-day life comes through on an iPhone, or an iPad, or your computer screen, no-one gets off their bum. So, [expressive noise] it's says we've got so much technology that's kept us inside now.

Interviewer: Hmm. Exactly. Now, talking about inside, in the past two weeks have you been active around the house and yard, inside or outside?

Respondent: Uh-huh.

Interviewer: Yep?

Respondent: Yep.

Interviewer: So this is focused on home.

Respondent: Yeah, my little mess. Yeah.

Interviewer: Yep. So can you tell me what you did?

Respondent: Lawns, edges, twice in the last couple of weeks. Did it before I left, did it when I came back. Having not been there all that long, [month] I moved in – I mean within a week it looked like I'd been there for years, but you still...

Interviewer: Yeah, you're doing a lot of stuff aren't you?

Respondent: ...putting things where they're supposed to go – no, it doesn't go there, it goes over there – and you're still... I'm still arranging things, and rearranging things. And I'm naturally anal anyway, so my housework's up to date. [Laughter].

Interviewer: [Laughter]. So the intensity of that, would you call that vigorous or moderate?

Respondent: No, that's moderate. I'd do that sort of stuff in my sleep.

Interviewer: Yep. Yep, yep. And how long would you have done it for?

Respondent: Lawns and garden takes about half an hour, and the other faffing around the house, a couple of hours here, a couple of hours there.

Interviewer: Hmm, hmm. And you've already described how often you'd do that, but any particular time of the day that you did these things?

Respondent: The gardens in the morning. I'm more alert in the morning. By two o'clock I'm buggered, I've had enough. Well not so much physically, but mentally – if anybody wants me to make too much sense, it has to be before two. So I don't know if this is going to make much sense. [Laughter].

Interviewer: [Laughter]. No, you're making sense. [Laughter]. That's fine.

Respondent: [Laughter]. Yeah. Yeah, mostly in the morning.

Interviewer: Mostly in the morning?

Respondent: Yeah.

Interviewer: OK. Now, and OK, does this change at all during the season or time of year?

Respondent: No.

Interviewer: Yep. No. It has to be done. And if you had wanted to, is there anything that would have helped you be more active around the house or yard?

Respondent: No. I'm active enough. I don't need to do any more.

Interviewer: OK. Now, OK so now we'll look at have you been physically active for at least ten minutes during your leisure or spare time? So that's at home.

Respondent: Yep. Yep.

Interviewer: So can you tell me about what you did? And I think you've already sort of described a few things that you've done in the last couple of weeks.

Respondent: Hmm. Yeah. A lot of... I'm still nesting; I'm still doing things around the house, which is my leisure time. I don't know, I mean I...

Interviewer: You mentioned canoeing before.

Respondent: Yeah, yeah, kayaking. Yeah.

Interviewer: Yeah, kayaking. How often... would you have done that in the last couple of weeks?

Respondent: Once. Yeah.

Interviewer: Hmm.

Respondent: Yeah. Yeah. I like... I do that probably once a fortnight.

Interviewer: OK.

Respondent: Yeah. Just before going to [x] Island, I did it out in the ocean.

Interviewer: Wow.

Respondent: Yeah. There's some islands not far from here that you can go offshore. It's only half a kilometre, so went out there in amongst... there's three of them – [island], and [x] Island, just out of [town].

Interviewer: Beautiful.

Respondent: Yeah, it was. And the water wasn't flat either, it was... there was a little bit of a swell, but it was just a lovely, lovely thing to do.

Interviewer: So it can be protected enough that you can canoe out?

Respondent: Oh yeah, yeah. I mean sometimes there's currents and rips, so you don't go out there when the weather's not conducive. But it was a lovely thing to do, and that was for about, I don't know, two hours.

Interviewer: Hmm. So thinking back about all the things that you did in your spare time – like we've talked about canoeing, walking, and cycling – the intensity of that, how would you describe it?

Respondent: The walking moderate. The kayaking, yeah that was pretty... that was a bit of a doddle as well. There was a couple of times there where we – because I went with some friends, and we had a bit of a race for about 100 metres, and then we thought what are we doing this for?

Interviewer: [Laughter].

Respondent: Should be relaxing.

Interviewer: Enjoy it. Yeah. [Laughter].

Respondent: So pretty moderate. Yeah.

Interviewer: Yeah. OK. And... OK. And how long... you've talked about how long you've cycled and walked, but how long did you canoe for?

Respondent: Two hours.

Interviewer: OK. You told me how, and when, and where.

Respondent: Uh-huh.

Interviewer: OK. Right. And who would you do your exercise with, in your leisure time and spare time? Would it be a mixture of doing it by yourself and with others?

Respondent: Mostly by myself. I'm separated, so... and that's fine, that's just... that's life. So I just carry on, and just do most of it myself. Every... probably 90% of it is on my own. Yep.

Interviewer: Uh-huh. And does it change depending on the season and time of year?

Respondent: No. Not really.

Interviewer: No? OK. And if you wanted to, is there anything that would have helped you be more active in your leisure or spare time?

Respondent: If I wanted to? No.

Interviewer: No?

Respondent: No.

Interviewer: OK. We'll move on now to physical environments, and having talked about where you've lived and your physical activity, I'd just like to talk a bit about the physical environments where you live, work, and play. When I talk about physical environment, I'm meaning all the physical things that surround you – roads, trees, parks, open spaces, river, shops, traffic, lights, all those sorts of things – and some of these things are things that people living in urban areas have said are important, or they might be important to their physical activity, so I wanted to ask whether they're relevant to you. So can you tell me a bit about how easy or difficult it is to be physically active in your neighbourhood?

Respondent: My neighbourhood.

Interviewer: Hmm. The area you live. Hmm.

Respondent: Yep. Yep. Easy.

Interviewer: Yeah?

Respondent: Yeah. Easy.

Interviewer: Yeah. And what makes it easy?

Respondent: The location that I've chosen. It's easy, accessible to various places that I like to go, and can get there fairly quickly.

Interviewer: Hmm. And that's what you'd actually considered and planned for. Yeah.

Respondent: Yeah, yeah.

Interviewer: Now, looking at availability and accessibility of places to be active, if I was... were to ask you about places to be active in your neighbourhood area, what sorts of things would you tell me about?

Respondent: Well, close to where I live are a number of Reserves within walking distance, lawned areas on the riverfront, a few parks, less than a kilometre is the beach, there's a big Reserve grass lawned area there for families, for pets on a lead. Then you can go to the beach and you can have your pet off the lead, under control, on the beach. It's all accessible, the whole lot.

Interviewer: Uh-huh. So there's a lot of opportunities there.

Respondent: Hmm.

Interviewer: Now, so there's definitely the availability of places to be active, and there's definitely access – the places are convenient. Are they easy to access?

Respondent: Yep.

Interviewer: Yeah. For instance they don't have specific costs, or opening hours to them at all?

Respondent: No. No, you can walk there, you can drive there, you can cycle there.

Interviewer: Hmm. OK. And does having places to be active actually influence whether you're active or not?

Respondent: Yeah, it does. Yep.

Interviewer: Hmm. And if you wanted to be more active, are there things or places that would help you?

Respondent: Not necessarily, because they're all...

Interviewer: In terms of availability and accessibility, because it's there...

Respondent: Yeah, it's already there, just got to use it. Again, something under cover for the wet season would be handy, but again that's that cycling thing. Or even a cycle path that's not part of the road system. Or if it is part of the road system, it's actually designated and marked so that other traffic users – because I know you drive, you're aware of other things on the road, if there's a place there that's set aside for cyclists, you then OK, well that's... you're aware, so it becomes more safe. Without that there, its user beware. You know, just use it at your own risk kind of thing. And motorists can be quite frustrated with cyclists, especially if some of them are riding two abreast and they shouldn't. So it's... you've got to share.

Interviewer: Hmm, hmm. Yeah, it's tricky, isn't it?

Respondent: It is tricky.

Interviewer: Hmm. Now destinations – if I were to ask you about places you could walk or cycle to from your home, what sort of things would you tell me about?

Respondent: Destinations? I would cycle to [town], which is nine, ten kilometres away, so it's a 20 kilometre circuit. And that's probably all. I wouldn't cycle anywhere from here. I'd cycle around [town], and then to [town], and that's about it. But I...

Interviewer: Hmm, so it's around the actual...

Respondent: The immediate area.

Interviewer: ...sort of immediate area. Hmm.

Respondent: Yep. Yeah. But I have got a rack to put the bike on, so I can go, like I have done to [city]. I've gone to [city] and used the cycle paths there.

Interviewer: Hmm. So does having or not having destinations influence whether you're active or not?

Respondent: Not really, no.

Interviewer: If you wanted to be more active, would it help if there were more destinations within easy walking or riding distance?

Respondent: I don't think so, about destinations, because you do it... yes, you want to go and have a nice view when you're cycling, you don't... but you do it for a purpose. It is a form of exercise. I'd be just as happy going around in a circuit, in a rollerdrome, doing my ten k's. I mean it's nice to go and have a look at the view and whatever, and have a bit of a doddle and a cup of tea halfway. You can still do... you can still get your physical activity done, whether you're going round in circles, or on a road.

Interviewer: Yeah. Fair enough. Now, I was going to ask you about neighbourhood design, i.e. the non-natural, or built, or manmade features of your neighbourhood. What sort of things would you tell me about?

Respondent: I'm lucky, in the area that I live is... that there are natural areas that haven't been altered to any extent, or if they have it's very, very slight. So there's natural areas along the river frontage, and some of them that have been altered have been rehabilitated back to natural anyway, so that's within eye shot.

Their design of the streetscape is very old, because it's one of the closest streets to the centre of [town] when it was first established in the late 1800s.

Interviewer: So footpaths, and lighting, and all of that.

Respondent: Yeah, so there's wide footpaths... yeah, that's all there.

Interviewer: Uh-huh. And streets that connect with each other?

Respondent: Yeah, in that particular precinct they're all square, as was the design back then. So there's not a lot of interesting bits in it, but there's some interesting old homes.

Interviewer: Hmm. OK.

Respondent: Hmm. So it's quite aesthetically pleasing in that most people who live in that particular section own their homes, and take care. So there's a sense of pride in that region.

Interviewer: Hmm. OK. And do the built or non-natural features in your neighbourhood influence whether you're physically active or not?

Respondent: No.

Interviewer: OK. And if you wanted to be more active, are there any features that are non-natural, or built, or manmade, in your neighbourhood that would help?

Respondent: Yeah, it's the cycle... the cycle path... the designated yellow strip, or the dotted line that says that metre and a half is for cycles. I would use it more.

Interviewer: Yeah. Fair enough. Now you mentioned aesthetics, that's the next set of questions, the aesthetic qualities, the natural qualities, or attractiveness of your neighbourhood area. What sorts of things would you tell me about?

Respondent: People enjoy their gardens, and it's relatively... they're maintained and fresh looking. You know it's not...

Interviewer: You've talked about the river, the parks, and the natural scenery, the trees, and the coast.

Respondent: Yeah, well the river's... yeah, and from the park near the river you can see the [x] Range, you can see...

Interviewer: Oh, really?

Respondent: Yeah.

Interviewer: Yeah.

Respondent: You know, you've got the water, and in the background the Range. So to have that within three minutes of my front door...

Interviewer: That's pretty impressive isn't it?

Respondent: It is. It's... I find it again, for the soul it's really quite... it's a powerful thing to have access to that. To live in a shoebox in a city would be the destruction of me. I could do it if I had access to all these other things, but I've got that here.

Interviewer: Hmm, hmm. Yeah. And if you wanted to be more active, are there features in the natural features in your neighbourhood that might help?

Respondent: No.

Interviewer: No. OK. Because they're already there?

Respondent: Hmm.

Interviewer: Hmm. And the last area is about safety. If I asked you about safety in your neighbourhood, what would be the sorts of things you'd tell me about?

Respondent: You can walk around anywhere, anytime, there's no risk. [Laughter].

Interviewer: Hmm.

Respondent: Not that I have ever sensed it, or felt it, or seen it. It's a... I mean we all do it, and probably shouldn't, but I just go down to the river in the morning with the dogs, and I don't lock anything up. [Laughter]. You just go.

Interviewer: It's a nice feeling, isn't it?

Respondent: It is a nice feeling. And being on [x] Island, I've just come back from there, after having spent a couple of weeks there, and no-one locks cars, and no-one takes the keys out of them.

Interviewer: Hmm? [Laughter].

Respondent: You just don't.

Interviewer: Hmm.

Respondent: Yeah, it's... it is a different feeling.

Interviewer: Very different. Hmm.

Respondent: Hmm. Yeah.

Interviewer: Hmm. And OK, so are there any personal safety... does personal safety influence whether you're physically active or not?

Respondent: Yes, because I'm single, and I do exercise on my own, I choose not to do it of an evening. It'll be in daylight. And if it's really early I'll have the dogs with me, and that would ideally put anyone off if there was anything untoward potentially that could happen.

Interviewer: Hmm, hmm. That's something you're aware of?

Respondent: I'm aware of it. Yeah, I'm tuned into it, I'm aware of it. And I have lived in cities before to know how awkward that can be, and how much stress that can put on you.

Interviewer: Hmm. Uh-huh. And does road safety influence whether you're physically active or not?

Respondent: Not really. The areas that I choose to do my thing doesn't really involve walking along busy roads. I avoid them. Hmm.

Interviewer: Yeah. Yeah.

Respondent: I can easily... where I live I don't have to go on busy roads.

Interviewer: Yep. Yep, you've got those... the facilities. Yeah. Which is wonderful.

Respondent: Hmm. Hmm.

Interviewer: And if you wanted to be more active, are there any personal or road safety issues that could be addressed that might help?

Respondent: No. I think the streetscape lighting and all that kind of stuff is as good as it should be, and as good as it can get. No, I'm happy with that. There's just certain things that you just don't fight it, because there's no point in changing. It's going to cost too much. Adjust.

Interviewer: And it seems like there's been a lot of stuff actually done, even if it's more recently, but it's still... it's being done here to...

Respondent: There is. Yeah.

Interviewer: Yeah.

Respondent: The [x] Council is pretty forward thinking in that regard.

Interviewer: Hmm. And just an ending question before we finish up, if there's anything else you'd like to tell me about where you live, your environment, or any of the physical activity that we haven't covered today?

Respondent: I don't think... I don't think we have any extra. I think I've covered it fairly well. I mean just suffice to say that people will and could become more active if there's more pathways around. Even just to connect smaller areas. It doesn't have to be from one end of bloody [state] to the other, just smaller areas where mums and dads, and the kids, can actually get out.

Interviewer: More like the cycleway here that's local...

Respondent: Yeah, yeah. Yeah. You know a cycleway in particular, because little kids love their bikes. And if their mums and dads, either one, or both,

have an opportunity to get into as well without too much hassle, because see quite... it's a lot of hassle getting your family onto a pathway, and looking after them.

Interviewer: Hmm. You've got to organise yourself to get there.

Respondent: You do. You do.

Interviewer: And have all the gear and facilities to do it.

Respondent: Well, if this coastal pathway thing... it's just very exciting, because there's going to... there's little seats along the way where if you're puffed you stop.

Interviewer: Hmm.

Respondent: Picnic facilities. If you only want to do three kilometres and go and have a picnic, well let's go. It's just...

Interviewer: Yeah, you've got choices. That's good too.

Respondent: There's a lot of choices involved in that. And I think that'll be just the... such a good thing for the region – not so much just [town]. Again, farming... farmers who are just five minutes away, a bit hard if you're a farmer, but then they've got more space that their kids run around in anyway.

Interviewer: Yeah, that's true. Yeah, because they have to actually travel over to get there.

Respondent: Yeah. Yeah.

Interviewer: Yeah.

Respondent: I mean you've got to come into town to do shopping, whatever, anyway, most likely, so if there's an easy access, right whilst we're going shopping, let's go and ride the bikes between here and there as a family unit, and promote that. It would work.

Interviewer: Yeah. No, I think that's a really good idea. I think what you're saying about sort of linking activities.

Respondent: Linking, hmm.

Interviewer: You know that's one of the things coming up too, is that you don't just do one thing for one thing. If you can sort of link it to a few things that it's part of your life...

Respondent: Yep, it becomes... it is, it's a lifestyle.

Interviewer: Yeah.

Respondent: It's a lifestyle as a choice.

Interviewer: Yeah.

Respondent: I mean I go shopping, I go cycle, then go shopping, and then I go to the cafe after for lunch. So you do a whole heap of things in the one hit, and get your fix in every area.

Interviewer: Hmm. Hmm. Very important. [Laughter].

Respondent: It is. It is.

Interviewer: Oh, yeah.

Respondent: It is. I don't always get it right, but that's the aim. Hmm. But just being busy, focused on one thing, and becoming mundane, and some people get depressed with all of that.

Interviewer: Hmm. Hmm.

Respondent: And sleep – if you get lots of sleep it's good too.

Interviewer: [Laughter]. Thank you very much for that.

Interviewer: So I thought we might actually start by talking a bit about the place where you live.

Respondent: Uh-huh.

Interviewer: And what it's like to live there. So if... could you tell me about what it's like living around where you do, your likes and dislikes of it?

Respondent: We live in a coastal position situated between [town] and [town]. It's a gorgeous place to live, there's a lot of privacy, we've got access to beaches close by and rocks and fishing and within walking distance. It's very private and quiet and it's... I think it's a pretty good central place, and you get the opportunity to shop and that sort of thing at the larger centres, but you can still feel like you're a little bit out of the rat race so to speak.

Interviewer: So you're a bit... are you closer to [town] or [town] or somewhere...

Respondent: We're pretty much sort of right in between really.

Interviewer: OK.

Respondent: Uh-huh. So if we chose to drive to either then it's probably about the same amount of time.

Interviewer: OK. And having just gone along that road, it's quite a narrow little road. Are you back from the coast or...

Respondent: Uh-huh. We're sort of...

Interviewer: Which part are you?

Respondent: We're sort of on the [x] side...

Interviewer: Oh right.

Respondent: ... but we'd be [distance] down to the water I suppose. So we're fairly close.

Interviewer: 'Cause it's quite narrow, so that's quite opportune... lucky finding a little spot that's on the coast. It's beautiful.

Respondent: Uh-huh. Well it used to be... [partner] place was an old shack and we've just sort of gradually done that up.

Interviewer: Ah right.

Respondent: Uh-huh. It's actually a nature reserve, so we have seagulls nesting on the islands.

Interviewer: So it's a designated reserve is it?

Respondent: Uh-huh. Nature reserve. Although that doesn't give it a lot of protection, but it does give it some sort of protection, but. So it's actually a large nesting spot for the fairy penguins...

Interviewer: Oh right.

Respondent: ... they're little penguins, and also the seagulls, cormorants sea eagles and yeah, we get a lot of bird life around there which is really lovely. So that's also really nice for your wellbeing as well, to sort of be surrounded by nature like that.

Interviewer: And so do you live in a house with a house block or is it bigger?

Respondent: Uh-huh. It's actually a house block, I'm not sure of how big, about 800 square metres I suppose.

Interviewer: So a reasonable size one?

Respondent: Uh-huh. Just three bedrooms, only a fairly small house.

Interviewer: And the length of time that you've been living there?

Respondent: I've been there about four years.

Interviewer: And your reasons for living there?

Respondent: I moved in with [partner] who I'm in a relationship with. Yeah, so that's why I moved up there, which was very fortunate really.

Interviewer: Absolutely.

Respondent: That's right. It's like...

Interviewer: 'Cause it's a pretty nice place... well it's obviously a place that you were happy to move into?

Respondent: Uh-huh. Lovely. We've done a... as I said we've done a few bits and pieces to it to, so to make it a bit more liveable, so it's lovely.

Interviewer: And OK. So, and your nearest towns are... it's mainly [town] that's the biggest?

Respondent: Uh-huh. That's got the sort of market and stuff like that.

Interviewer: OK. All right. Now in research with women living in urban areas, people often sort of refer to their local neighbourhood. So I'm interested in understanding what the term neighbourhood means to women who live in rural areas. So does... what does the word neighbourhood mean to you?

Respondent: Uh-huh it's... oh probably I don't... not sure if I see it as a neighbourhood where we live or not really. I probably haven't really thought about it. I probably don't in some ways because there's not a lot of people around. We only really have one lot of neighbours that are across the fence.

Interviewer: Sort of fairly well next door?

Respondent: Uh-huh. And then some other people a bit further on which you wouldn't have daily contact with 'cause they're far enough away, so. No I don't really see it as a neighbourhood particularly. I'm not sure what I see it as really, but yeah, it's not like other places I've been where you actually kind of feel you're in a neighbourhood with lots of neighbours and stuff like that.

Interviewer: Around the place, yeah. And what about the word community? Does that... what does that mean to you?

Respondent: Community means to me probably the whole of the [x] coast in a way.

Interviewer: Uh-huh. OK.

Respondent: 'Cause we work around different areas, so you'd go to [city] and you'd go to [city] and so that to me feels like sort of the whole of the...

Interviewer: You've got a bit broader sense of...

Respondent: Uh-huh.

Interviewer: And communities more a geographic thing of, or is it more than geographic?

Respondent: It's probably more geographic to me in some ways. Yeah.

Interviewer: OK. And so in terms of distance, so roughly what would that be?

Respondent: Into... for my sense of...

Interviewer: Uh-huh.

Respondent: Oh I reckon it would be 30 sort of K radius I suppose, something like that.

Interviewer: OK. And I want to talk a bit about physical activity and as we've sort of said it's a very broad term, it means different things to different people. So what does the term physical activity mean to you?

Respondent: To me it's anything that gets your body moving really. So it can be a wide variety of things from actual deliberate setting about doing some exercise or gardening, vacuuming, anything.

Interviewer: Anything in your day to day life. And where does physical activity, oh sorry, do you consider yourself physically active?

Respondent: Uh-huh.

Interviewer: OK. And why?

Respondent: Well we deliberately set ourselves to exercise and stay fit. And we also, and this is as far as general activities, with gardening and mowing the lawns and yeah, so definitely do plenty of physical activity in that respect, and actually sort of try to make that effort to do at least half an hour up to an hour a day sort of thing.

Interviewer: We'll go into a bit more detail about what that might be in a moment. And where does physical activity fit in your life? Is it sort of important, high priority, not particularly...

Respondent: To me it's a very high priority. I've had some issues with post traumatic stress and some depression issues over the years and so physical activity is extremely important to me because of the feel good factor and, so I wouldn't be sitting here if I don't do it and make

a deliberate effort to do that, so very important. So, oh right up there with everything I think. Yeah. And, plus I've always played so much sport that yeah, from a very early age played in quite a few state teams in different variations of sport, so yeah, it's been a really...

Interviewer: It's always been an important part of your life.

Respondent: Very. Very important.

Interviewer: OK. Now in research we often think about physical activity as any activity that lasts for at least ten minutes, that the definition of health benefit.

Respondent: Uh-huh. Sure.

Interviewer: And causes the body to work harder, your heart... feel your heart rate go up, you feel a bit warmer, and maybe a bit of huffing and puffing. So I want to think... get you to think about this time for physical activity.

Respondent: Uh-huh.

Interviewer: Over the last two weeks. So in your work, have you been active at work for at least ten minutes of the time in the last two weeks?

Respondent: Uh-huh. It's funny, 'cause our work is so sort of... we work from home so it's... we don't actually sort of sit down at nine and then leave work at five, so it's not sort of like an office environment or something like that. So what we generally do is do a couple of hours in the morning, just thinking over the last couple of weeks, and then we've actually just started going to the gym. So we go to the gym for an hour or so and then we do a bit more work, and then we might go for a walk. So

yeah, I suppose during the working day we would do exercise fairly regularly.

Interviewer: OK. Uh-huh.

Respondent: And that would generally be how it is. I mean, oh sometimes when you've got a bit of a deadline, you're getting a bit slack and you might sit there for hours...

Interviewer: I think that's pretty much our day.

Respondent: Uh-huh it's like sort of...

Interviewer: It's like oh where did that time go?

Respondent: Uh-huh. That's right.

Interviewer: But you try and...

Respondent: Uh-huh. We've got some stationery bikes at home so often I'll just sit and watch something on TV and pedal away as well, so yeah, we sort of try and make provision for it. See I find I start to go and get a bit of cabin fever if I don't get some exercise during the day. Yeah, just can't sit at the computer the whole time, so yeah.

Interviewer: OK. And thinking back about what you did at work, like I look at different parts of your life and we may overlap here, that's absolutely fine. But the only reason for doing it is to make sure we don't miss out things, 'cause often you don't compartmentally live quite like this, but anyway. OK. So thinking back over the last couple of weeks, what would you say the intensity of that exercise was? Would it be vigorous or moderate or fairly steady?

Respondent: Range from steady to vigorous. Yeah. So if we were at the gym and we're running on the treadmill then it's quite vigorous. And then with the walking I suppose not so vigorous. So all of the above.

Interviewer: And how long would you have... do those activities for?

Respondent: I'd say a minimum of half an hour.

Interviewer: Uh-huh. OK. And how often in the last couple of weeks?

Respondent: Most days I'd say. I'd say pretty much every day in the last two weeks.

Interviewer: OK. As you were saying you get to feel it if you haven't.

Respondent: Uh-huh. That's right. I mean so yeah, like saying you sort of make provisions. So if it is raining or something there's things we can do.

Interviewer: Uh-huh. With such a really interesting idea is that you sort of plan that so you can... depending on the weather there are things you can do as well.

Respondent: Absolutely. Uh-huh.

Interviewer: So generally during the day is the activity?

Respondent: Uh-huh.

Interviewer: Uh-huh. And that was... the other question I was going to ask, does it change depending on the season or the time of the year?

Respondent: Uh-huh. It just changes just by the day. There's not... we don't have a set routine or anything like that.

Interviewer: And in winter would it change?

Respondent: I don't think it'd vary a lot. I mean the only thing with summer you probably spend more time outside maybe, but...

Interviewer: You still try and keep your range of activity.

Respondent: Uh-huh. We still try and get out even if it's cloudy or not that pleasant.

Interviewer: OK. And if you had wanted to, is there anything that would have helped you being more active at work?

Respondent: No.

Interviewer: Uh-huh. You're happy with how you're being active. Uh-huh. Now in the past two weeks have you walked or cycled for at least minutes of the time to get to or from places?

Respondent: Uh-huh.

Interviewer: OK. Can you tell me about it?

Respondent: Well yesterday I went for a ride out to [attraction] from town, from in central [city]. That was probably about 45 minutes there and 45 minutes back, so that was a good ride.

Interviewer: And was that fairly vigorous or...

Respondent: Uh-huh.

Interviewer: Uh-huh.

Respondent: Pretty much pedalled the whole time. So it was actually a good ride, it's flat so you've got to keep moving.

Interviewer: You keep going.

Respondent: Very lucky as it were. Very jealous.

Interviewer: Uh-huh

Respondent: Uh-huh. We've walked to... we walk to a lot of places, so just in the last few days we've walked from where we're staying down to town, down town and that would generally be the case. The only thing is we do live on a road that's not very conducive to walking or riding, so that's a bit of an issue.

Interviewer: So... OK. So can you, at home, walk or cycle from your home?

Respondent: Uh-huh we can. And it is actually a... it's known as a bit of a cycling training route, so a lot of the cyclists there in the large groups cycle. But it's not so friendly when it's just one or two people, the motorists can be a little bit impatient and...

Interviewer: So would it be something you would do?

Respondent: Not that regularly because it's not, yeah. But we do, we still do. And it's... if... as long as there's two... I think if you don't pick the peak traffic times it's quite... so if we choose to go during the day and ride to [town] or something like that, then it's not so bad.

Interviewer: 'Cause it's a pretty narrow road if I remember.

Respondent: It is a very narrow road. Uh-huh. So the vehicles virtually... they have to overtake you to get past you on that road, so that's a bit difficult.

Interviewer: And it's twisty and turny and there's no way to get off.

Respondent: No.

Interviewer: Very pretty. So sometimes you might cycle from home. Do you walk from home?

Respondent: No.

Interviewer: OK.

Respondent: Well it's not really a sort of road you'd walk on, and the other option is the railway track, but that's not very good for walking on either, probably not that safe either, so.

Interviewer: It's in use is it? It's still being used?

Respondent: Uh-huh. It's four or five times a day but it's still in use, yeah.

Interviewer: And so does that mean that you would generally drive to where you wanted to walk?

Respondent: Uh-huh. So say for example we walk... if we got for a walk that we do on the beach which takes about 45 minutes, we probably drive for three minutes to get there. So it's not very far, but it's still virtually you've got to get in your car to go.

Interviewer: OK. All right. So, just going back on the last couple of weeks, we're talking about cycling and that being vigorous and the duration. And what about walking. How... have you done some walking... you obviously have done walking in the last couple of weeks as well. So how often would you have done that?

Respondent: We try and walk every day for at least half an hour, so.

Interviewer: OK. And that would be to get in the car and do the three minutes and go down to...

Respondent: Uh-huh. Walk down to the beach. Yeah. Or if we go somewhere we do actually try and park a distance away and walk because it's just easier to park and that sort of thing. So yeah, when we, we sort of walk for half an hour each day.

Interviewer: Uh-huh. OK. And would you describe that as moderate or vigorous exercise?

Respondent: Depends what we do. It would only be moderate if we do the walk on the beach. But then we do fairly regularly use the [x] Range which is a walking trail not far from us.

Interviewer: 'Cause it's just behind you isn't it?

Respondent: Uh-huh. It's just behind [town] so that's probably a ten minute drive. And when we walk up through there it's vigorous. But... and we would probably average doing that every fortnight, once a fortnight if you averaged it out. We probably do that walk, so sometimes might go a month and we don't do it and then other times we might do it more.

Interviewer: OK. And if... and would this change depending on the season and time of year?

Respondent: No, not really.

Interviewer: 'Cause you already said you try and get out anyway. And if you wanted to, is there anything that would have helped you use walking or cycling to and from places?

Respondent: It would really help if we had a bike track that went round the coast, because I would ride to [city], we'd use it all the time, so it would really help us if there was the cycle track nearby.

Interviewer: 'Cause at the present moment when I've been talking with people in [town] they're talking about the cycle and walk track that goes to [x] Beach, so it only goes from [town] to [x] Beach at this moment?

Respondent: Uh-huh.

Interviewer: So it doesn't go along from your section of the coast?

Respondent: No. And our section would probably be the last because of the nature of the cliff and there's just not enough room to.

Interviewer: Uh-huh. I was wondering where they were going to put it?

Respondent: Uh-huh well...

Interviewer: I was wondering if the railway was still in use?

Respondent: Uh-huh it is. Otherwise that would be the ideal spot.

Interviewer: Uh-huh 'cause freights.

Respondent: So that would be...

Interviewer: That would be the ideal.

Respondent: Uh-huh. But they will work something out eventually when they... 'cause that'll sort of be the last probably a bit of link, everything else will sort of probably get done in the next few years I reckon, so yeah. [partner] actually did the study on the cycle way stuff, so...

Interviewer: Uh-huh she was mentioning that. So I was quizzing here about what was happening.

Respondent: Uh-huh. So I reckon yeah, it won't... as long as they get the funding and stuff I think that it will get done, 'cause I think it's important for the [x] coast to have that done.

Interviewer: Absolutely.

Respondent: And our public transport's so poor too, but it does give people another option of getting around.

Interviewer: Uh-huh. And my very brief... so going up to [town] yesterday was, and being in [town] where the community house is, when I went out to [town] I realised those roads actually then back on to the coast. So it's actually not very far. And people in [town], the other side of the river, have actually said they're very close to the coast, so it would seem like it would certainly open up some opportunities for people.

Respondent: It really would. I think it would make a big difference to the health of the people on the coast really. I think they would really embrace it and tourists and a lot of different reasons to have it.

Interviewer: Oh it'd be great.

Respondent: Because there's a good chance they might be able to get the railway from [town] to [town] it's not... no longer used. So you can imagine having a bike track that went from Port [x] down to [town] [inaudible 19.31]

Interviewer: Uh-huh. And that would be fairly flat 'cause of the grade of the track.

Respondent: That's right. Uh-huh. So it'd be fantastic.

Interviewer: That'd be good.

Respondent: Uh-huh. It'd be really good.

Interviewer: Get it before they have any other ideas.

Respondent: They're talking about putting a tourist train on it and I think...

Interviewer: No.

Respondent: ... no, we don't want more people sitting around.

Interviewer: Uh-huh. Ok. Now where were we? We were talking about... oh yes, things that would help.

Respondent: Uh-huh.

Interviewer: So we've done that. OK. And in the past couple of weeks, have you been active around the house or the yard, like inside and outside of your home?

Respondent: Uh-huh.

Interviewer: Uh-huh. OK.

Respondent: So general stuff like vacuuming, cleaning and which I think vacuuming actually would be quite physical.

Interviewer: Yeah certainly, you'd get warm.

Respondent: You might get a a bit of a sweat up. Uh-huh. Things like cleaning cobwebs and stuff like that. And also mowing, we've got a big lawn so that's a bit of a... it's takes about an hour to mow, so it's a bit of a physical job.

Interviewer: Here it's been, people have been generally saying they've got to mow it quite frequently at this time of the year.

Respondent: Uh-huh. Well normally it's... going in to the summer we normally don't have to mow hardly at all, but it was so much rain that it was just raining the whole time. It's probably the same down here I would imagine, but yeah. So yeah, we are physical around the house.

Interviewer: OK. And what... the intensity of that activity?

Respondent: Moderate. So it'd be, what was the lowest one?

Interviewer: I call it steady for want of a better word.

Respondent: Uh-huh. So steady to moderate.

Interviewer: And what be the duration?

Respondent: Anywhere from ten minutes to an hour.

Interviewer: And the frequency? How often you would have done that?

Respondent: I'd say a couple of times a week I suppose.

Interviewer: OK. And did you do it in any particular time of the day?

Respondent: No there isn't.

Interviewer: Uh-huh. OK. And do you do it alone or with others?

Respondent: Sometimes with [partner], just depending on what we've got on and how we separate the chores out.

Interviewer: And does it change at all with the season?

Respondent: No.

Interviewer: No. OK. And if you had wanted to, is there anything that would have helped you being more active around the home and inside or out?

Respondent: No.

Interviewer: OK. Fine. Now the last part of this one is, have you been physically active for at least ten minutes during your leisure or spare time. OK.

Respondent: That's when you mainly be physical.

Interviewer: OK. Can you tell me what you did in the last couple of weeks?

Respondent: Oh the last couple of weeks we probably done... oh well we've been to the gym. So we started doing some weights. Recognise we're getting a bit older and if you don't use it you lose it and yeah. I had a bit of a nasty injury about 18 months ago and snapped my Achilles tendon.

Interviewer: Ah.

Respondent: Uh-huh. So that...

Interviewer: That takes a long time.

Respondent: Well my leg and my actual calf muscle in this leg's never sort of recovered...

Interviewer: Which affects the whole...

Respondent: Uh-huh. So I sort of thought from that it wasn't getting better. I was actually, I was walking with a bit of a limp and I thought I think I really need to start to work with doing some weights, some leg stuff, so that's what took us back there. So we've done the gym. We walked up the [x] Range, one of the mountains, a couple... a few days ago. Uh-huh, we did the cycling yesterday out to [attraction], walked along the beach. Uh-huh we've done lots of stuff.

Interviewer: And that sort of fits in with you were saying that you tend to do something every day. And what would be the intensity of those?

Respondent: We try to do something vigorous every day.

Interviewer: OK.

Respondent: So I suppose you'd say it's some... like I said some of the activities like the walking might be moderate, but that we're doing...

Interviewer: The bush walks.

Respondent: Uh-huh. And the bush walks would be vigorous. Uh-huh.

Interviewer: OK. And the duration?

Respondent: Ranging from half an hour to an hour and a half, two hours probably.

Interviewer: A bush walk would sometimes be longer.

Respondent: Uh-huh. Up to... oh actually yeah we did do a reasonable walk some friends, that was three hours on the weekend, so yeah.

Interviewer: And OK. So let's see... so do you usually do your activity alone or with others?

Respondent: Usually [partner].

Interviewer: OK. And does it change depending on the season?

Respondent: No.

Interviewer: No. OK. Right. And if you'd wanted to, is there anything that would have helped you be more active in your leisure or spare time?

Respondent: No. I mean up until recently I thought... I still do think that [town] lacks exercise facilities, gyms and stuff. We've only just got onto this guy that he's moved to a different gym and he's got a reasonable amount of equipment, but he's got... he does have limited opening hours and doesn't have wonderful equipment 'cause he's sort of done it on a bit of a shoestring sort of a budget. So definitely in [town] we don't have good gyms and that's... I think that's been a bit of an issue for some time. And I'm surprised that a proper operator hasn't come in and put one of those 24 hour gyms or something in, because I think it would actually do quite well. And it's a little bit far to get motivated to travel to [city] or [city] 'cause you know you've got to jump in the car and drive about 30 K's so yeah, so it puts you off, so. But there is somewhere we can go now at least. That has been an issue in the past. And also I think there's a lack of aquatic facilities as well.

Interviewer: Uh-huh, so if you want to go swimming.

Respondent: Uh-huh, well it's just got the body if you do work out and that sort of stuff to be able to do something that's now weight gearing.

Interviewer: Is there anywhere?

Respondent: No, there's... [city] got a pool, and [city], but they're not open during the winter anyway.

Interviewer: Oh OK.

Respondent: And there's a small pool in [town] at one of the schools, but so many people use it that it's... you sort of have to get in, you have to do the laps. And there is a... also a physio pool up at one of the old people's homes which you can use if you have an injury. So I use that one when I was recovering from my leg.

Interviewer: OK.

Respondent: But yeah, it's pretty limited.

Interviewer: OK. And where... I'll go on a bit more in a moment about availability and accessibility. But just one question, we actually want to go on to physical environments and looking at the environment that you want to... that you live and work and play in. And I'm interested in... and talking about physical environment and by that I mean physical things that surround you like the roads and trees, the coast, the houses... you know shops, recreation facilities. Because when research is being done with people in urban areas they often say that that might actually contribute to helping with their physical activities, so we're interested in finding out whether that's rural areas too. So can you tell me a bit about how easy or difficult it is to be physically active in your area?

Respondent: I think it's very easy. We're pretty blessed really with having things in the natural environment that are so close by like the walking tracks and those sorts of things. So the [x] Range is terrific. We've also got great beaches. [town] got a lot of really lovely parks, very, probably one of the best park towns I've come across really, it's just absolutely superb. Uh-huh, so I don't think there's any barriers particularly to physical exercise. It might be necessarily a structured environment, you know some people would think you need to have a track or you need to have it, but if you can find things in your natural environment...

Interviewer: You can go.

Respondent: ... there's plenty of stuff to do.

Interviewer: And for you where you live, it just means anything from a few moments drive to a few minutes' drive?

Respondent: Uh-huh. I mean it would be great if we could just jump on the bike on a safe road and we could do that.

Interviewer: Uh-huh. Or that track.

Respondent: Uh-huh. That's right.

Interviewer: The coastal track.

Respondent: I know. Right.

Interviewer: That'd be great. OK. So I want to talk about availability and accessibility which you've actually talked a lot about, but just wanted to check whether you wanted to add some things about that. If I were to ask you about a place to be active in your neighbourhood area, you've actually told me quite a bit about that, the recreation facilities, the track, cycle tracks, sports clubs, parks. And you've just told me that they are quite available. I was just interested in access 'cause you actually were talking about particularly the gym and the... how easy they are to access, and whether you wanted to say anything more about that sort of cost and opening hours and things like that.

Respondent: Uh-huh.

Interviewer: And now the pool was the other one.

Respondent: Uh-huh. I mean I could... I understand that people that are on lower incomes or when we have times when we haven't got contracts

coming in and stuff that the gym's about $60 each per month, so it could be a factor that if things were a little bit tight money wise. And yeah, but most things we do don't cost money, so it's not really an issue.

Interviewer: Uh-huh. So that's fine. And does having places to be active influence whether you're active or not?

Respondent: Uh-huh, I think, yeah, definitely. But I think everywhere's got somewhere where you can...

Interviewer: so that's fine.

Respondent: Uh-huh.

Interviewer: OK. And if you wanted to be more active, are there things or places that would help if they were available or more accessible?

Respondent: Uh-huh, only what I was saying about possibly some more aquatic facilities and bike tracks and those things. Because I think incorporated into your daily life as part of getting to and from work is important, because for some people it's the only bit... that might be a limited opportunity for them to take up, so.

Interviewer: And that seems to be one of the themes that's coming through, is having a look at your opportunities in your everyday life.

Respondent: Uh-huh.

Interviewer: And looking at options of how you mow rather than necessarily having to think about a specific activity then save up and have to go particularly...

Respondent: That's right.

Interviewer: ... to to do. Destinations. If I were to ask you about places that you could cycle or walk to from your home, what sorts of things would you tell me about? For instance any shops or recreation facilities, friends.

Respondent: If we were going to walk or cycle from home?

Interviewer: Uh-huh, though you actually have said that you don't on the whole...

Respondent: Uh-huh. I'm not crazy about getting on that road, so, and walking that's... so it's not conducive to walking either because there's no actual sort of verge at all.

Interviewer: No.

Respondent: Uh-huh. So we only get on there when we really... when we know it's going to be quieter and we want a bit of a ride.

Interviewer: Fair enough. So having destinations, does that influence you to be active? Places to go?

Respondent: Uh-huh well it does. Say for example, just yesterday with the opportunity to ride out to the museum. So yeah, I like that. Plus like the opportunity we know we can walk into town and walk home, so yeah, it does. I mean if you can use that as part of your exercise then it's really important.

Interviewer: Uh-huh. And of course the question rurally is whether you can because of the nature of... and it's often what's coming up is ..

Respondent: Uh-huh. Absolutely.

Interviewer: Traffic.

Respondent: Uh-huh.

Interviewer: So if you wanted to be more active, would it help if there were more destinations within easy, easy walking and riding distance?

Respondent: Uh-huh.

Interviewer: OK. Now, neighbourhood function or design, which is looking at the non natural or built or manmade features of your environment. If I were to ask you about that, what would you tell me about the non natural features, the built features of the area that you live in?

Respondent: In relation to physical activity or just generally?

Interviewer: Just generally. I mean do you have footpaths and lighting and things like that?

Respondent: Uh-huh. That... look I would say that generally the coast is very limited, where we live in [town] there's not many footpaths. The lightings ordinary. It's not a very well designed town for that reason, I don't think they've ever thought about walk ability or anything like that, particularly up until now. So yeah, I'd say it's fairly, yeah, very... I'd say it was sort of an hoc sort of design.

Interviewer: Uh-huh. And certainly when we talked about roads, they're a big impact.

Respondent: Absolutely. Uh-huh.

Interviewer: So do the built features of your area influence whether you are physically active or not?

Respondent: Well they do for us because of the fact the road is so narrow and trying to get... if you want to ride or walk. It's... yeah, it's not safe really more than anything.

Interviewer: And you can't go coastward where you are because it's quite rocky isn't it?

Respondent: Uh-huh. That's right.

Interviewer: They're rocky sections.

Respondent: Uh-huh. So the only thing we, like I said we've got is the railway track. That's it. I'm sure I'd not recommend it.

Interviewer: That's a bit unsafe too.

Respondent: I want you off the railway tracks not getting on them.

Interviewer: No. OK. So if you wanted to be more active, are there built features in your area that would help you? You've certainly talked about that more coast, walkway, cycle track.

Respondent: Uh-huh. Well they... if yeah. Cycle track and the walk track that would be... make a massive difference.

Interviewer: If they're going to take the issue of the road traffic away then you're going to have to think about footpaths 'cause you'd have them.

Respondent: And then everyone if they want to could commute to work and you know.

Interviewer: Now the other side is the aesthetics, the natural part of your environment. If I were to ask you about the aesthetic qualities or the attractiveness of your neighbourhood, what sort of things would you tell me about?

Respondent: I mean we live in a lovely area, it's probably been a bit degraded over the years in relation to sort of some farming practices and that sort of stuff in the area, 'cause I know there's a lot of weeds, blackberries and that sort of thing which is a shame.

Interviewer: Is that in the coastal reserve section itself?

Respondent: There is, yeah, there's little bits of it down that area. There's actually a sort of like a bit of farmland that's between us and so that's... there's yeah, it's a bit of sea spurge and a few things that we try and keep on top of. But apart from that it's a beautiful area here.

Interviewer: And do you think the... your local environment is aesthetically pleasing?

Respondent: Uh-huh.

Interviewer: Uh-huh. Do you think the aesthetics of your neigh... of your area influence whether you're physically active or not?

Respondent: Uh-huh. I think they do because we look out of our windows and you see the ocean and you see, and it does... I think it just... it's good for your wellbeing and how you feel about the natural world really if you can have a nice place to look out on to.

Interviewer: And if you wanted to be more active, are there features of your area that might help if they were aesthetically pleasing or attractive?

Respondent: No.

Interviewer: I think you've got a pretty special place...

Respondent: Uh-huh.

Interviewer: ... from the limited little look I had...

Respondent: That's right.

Interviewer: ... along the coast, that coast yesterday.

Respondent: Absolutely.

Interviewer: It's gorgeous. 'Cause you've got those... the little islands off the coast as well.

Respondent: Uh-huh, well that's where we are. Just where those islands are.

Interviewer: Oh OK. Oh right. It's very pretty.

Respondent: Uh-huh it is. Gorgeous. So we can actually take the kayak and go round those and that's how, we just drag, we just drive it down the back on the ute and plop it in and it's absolutely beautiful.

Interviewer: So that's another activity?

Respondent: Uh-huh. Absolutely. We haven't done heaps of it yet, but we are lucky in [town] because we've got the river, you can kayak on that really easily and it's lovely, and you've got the islands as well. So it's a really good kayaking.

Interviewer: So you've got beautiful water coast and coastal vegetation, and then you've got the ranges behind.

Respondent: Uh-huh, that's right. So you've got everything there. I sometimes wonder too that at how well it's marketed to people in that, the [x] Range just doesn't really seem to be used very much by people, but I mean it's perfect really. So whether that's part of it as well, is just letting people know more about...

Interviewer: So does it have a main access point? It's not from [town] is it?

Respondent: No. [town].

Interviewer: From [town]. So, and that's another feature for [town] isn't it?

Respondent: Oh absolutely. Uh-huh. It's surprising that... I mean I see the people that live and have you been up there, no I've never been up there. And it's like ten minutes away. So it's only ten minutes from [town] and close to [city] and so it could be used quite a lot for people if they wanted. 'Cause probably sometimes the maintenance of it, parks are pretty stretched and...

Interviewer: Uh-huh. They are always stretched.

Respondent: So if a big storm comes through and, it is.

Interviewer: Environment - they always seem to have less resources than anyone else.

Respondent: Uh-huh. It's a real shame.

Interviewer: Uh-huh. OK. And if you... OK. I've sort of covered this. If there aren't any more features in your area that would help you've got it all there. There's only one other area to talk about, and that's safety. And if I were to talk to you about safety in your area, what sorts of things would you tell me about?

Respondent: I'd say, like we've talked about the road thing, but as far as if we wanted to walk around the parks or anything like that it... I feel it feels like a really safe place. We don't have a lot of crime in [town] or crimes against the person so to speak. So yeah, I mean I think it has a nice safe feel.

Interviewer: So because you feel safe, personal safety and does that influence whether you're physically active or not?

Respondent: I suppose it does because you don't have to think about it particularly. But, so yeah, and the fact that when we do have to think about to ride the bikes oh yeah it does, it can effect whether you're physically active.

Interviewer: OK.

Respondent: ... whether you're physically active or not.

Interviewer: And does road safety influence what you're physical at?

Respondent: Uh-huh. It does.

Interviewer: Underline. Underline. And if you wanted to be more active, are there are... personal or road safety issues that if they were addressed might help you become more...

Respondent: Uh-huh. I think I would like to see the speed limit reduced.

Interviewer: Uh-huh.

Respondent: There's no need to have an 80 kilometre an hour through there, it should be 60 and... because it's a known cycle, riding, training area and local councils need to be looking at those sorts of things, or at least having some signage. I mean there's not one sign there that says beware of cyclists or something like that. So something as basic as that could make a big difference. And maybe a little sign that said be patient, cyclists training in the area or something like that. So it's simple solutions sometimes.

Interviewer: Uh-huh. That are easy to implement.

Respondent: No, not costly or anything. But it could make... certainly make a difference around our area.

Interviewer: It certainly would help add to the whole like as you're saying, the idea of utilising its natural beauty far more.

Respondent: Uh-huh. Absolutely.

Interviewer: OK. Well before we finish up, I just wanted to check with you, if there's anything else that you'd like to tell us about where you live, or your environment or your physical activity that we haven't covered today?

Respondent: No, I think we've pretty much covered everything.

Interviewer: I think so. Thank you very, very much.

Respondent: It's a pleasure.

Interviewer: That's much appreciated.

Respondent: Great study that you're doing.
